# Quantifying the fatal and non-fatal burden of disease associated with child growth failure, 2000–2023: a systematic analysis from the Global Burden of Disease Study 2023

**DOI:** 10.1016/S2352-4642(25)00303-7

**Published:** 2026-01

**Authors:** GBD 2023 Child Growth Failure Collaborators, Christopher E Troeger, Michael Benjamin Arndt, Hasan Aalruz, Meriem Abdoun, Auwal Abdullahi, Mesfin Abebe, Armita Abedi, Alemwork Abie, Richard Gyan Aboagye, Hassan Abolhassani, Yonas Derso Abtew, Ahmed Abu-Zaid, Lawan Hassan Adamu, Mesafint Molla Adane, Isaac Yeboah Addo, Oyelola A Adegboye, Victor Adekanmbi, Juliana Bunmi Adetunji, Qorinah Estiningtyas Sakilah Adnani, Leticia Akua Adzigbli, Muhammad Sohail Afzal, Saira Afzal, Navidha Aggarwal, Aqeel Ahmad, Muayyad M Ahmad, Sajjad Ahmad, Elham Ahmadi, Ayman Ahmed, Haroon Ahmed, Mehrunnisha Sharif Ahmed, Mushood Ahmed, Marjan Ajami, Budi Aji, Syed Mahfuz Al Hasan, Omar Al Omari, Mohammad Khursheed Alam, Mohammed Albashtawy, Fentahun Alemnew, Ayman Al-Eyadhy, Mohammed Usman Ali, Rafat Ali, Syed Shujait Ali, Waad Ali, Joseph Uy Almazan, Hesham M Al-Mekhlafi, Mohammed A Alsabri, Najim Z. Alshahrani, Awais Altaf, Nelson Alvis-Guzman, Mohammad Al-Wardat, Hany Aly, Dickson A Amugsi, Abhishek Anil, Zelalem Alamrew Anteneh, Boluwatife Stephen Anuoluwa, Saeid Anvari, Anayochukwu Edward Anyasodor, Jalal Arabloo, Aleksandr Y Aravkin, Demelash Areda, Mahwish Arooj, Anton A Artamonov, Ashokan Arumugam, Nurila Aryntayeva, Bernard Kwadwo Yeboah Asiamah-Asare, Seyyed Shamsadin Athari, Maha Moh'd Wahbi Atout, Amlaku Mulat Aweke, Adedapo Wasiu Awotidebe, Asteray Assmie Ayenew, Melkalem Mamuye Azanaw, Shahkaar Aziz, Giridhara Rathnaiah Babu, Ruhai Bai, Jennifer L Baker, Wondu Feyisa Balcha, Palash Chandra Banik, Mainak Bardhan, Amadou Barrow, Shahid Bashir, Afisu Basiru, Quique Bassat, Mohammad-Mahdi Bastan, Priyamadhaba Behera, Michelle L Bell, Maryam Bemanalizadeh, Ajeet Singh Bhadoria, Sonu Bhaskar, Priyadarshini Bhattacharjee, Jasvinder Singh Bhatti, Catherine Bisignano, Bijit Biswas, Trupti Bodhare, Srinivasa Rao Bolla, Sri Harsha Boppana, Angelo Capodici, Rama Mohan Chandika, Vijay Kumar Chattu, Anis Ahmad Chaudhary, Moges Sisay Chekole, Hana Chen, Daniel Youngwhan Cho, Sonali Gajanan Choudhari, Isaac Sunday Chukwu, Erin Chung, Natalia Cruz-Martins, Alanna Gomes da Silva, Tukur Dahiru, Xiaochen Dai, Lalit Dandona, Rakhi Dandona, Samuel Demissie Darcho, Amira Hamed Darwish, Fernando Pio De la Hoz, Edgar Denova-Gutiérrez, Vinoth Gnana Chellaiyan Devanbu, Devananda Devegowda, Adriana Dima, Thanh Chi Do, Robert Kokou Dowou, Angel Belle Cheng Dy, Ibrahim Farahat El Bayoumy, Marwa Eldegwi, Muhammed Elhadi, Legesse Tesfaye Elilo, Iman El Sayed, Abidemi Omolara Fasanmi, Marta Figueiredo, Florian Fischer, Artem Alekseevich Fomenkov, Amin Fraij, Amanuel Tesfay Gebremedhin, Lemma Getacher, Genanew K Getahun, Maryam Gholamalizadeh, Nora M Gilbertson, Alem Abera Girmay, Mahaveer Golechha, Dinorah Gonzalez-Castell, Michal Grivna, Shi-Yang Guan, Mohammed Ibrahim Mohialdeen Gubari, Damitha Asanga Gunawardane, Zhifeng Guo, Bhawna Gupta, Rajat Das Gupta, Demewoz Haile, Nadia M Hamdy, Alexis J Handal, Nasrin Hanifi, Habtamu Endashaw Hareru, Eka Mishbahatul Marah Has, Ahmed I Hasaballah, Ikrama Hassan, Simon I Hay, Khezar Hayat, Jiawei He, Behzad Heibati, Austin Heuer, Kamal Hezam, Ramesh Holla, Md Sabbir Hossain, Hassan Hosseinzadeh, Sorin Hostiuc, Tanvir M Huda, Javid Hussain, Dursa Hussein, Hong-Han Huynh, Bing-Fang Hwang, Segun Emmanuel Ibitoye, Mustapha Immurana, Teresa R Iskander, Md Rabiul Islam, Md Sahidul Islam, Sheikh Mohammed Shariful Islam, Louis Jacob, Mihajlo Jakovljevic, Shubha Jayaram, Achala Upendra Jayatilleke, Wenyi Jin, Alex Joseph, Nitin Joseph, Ali Kabir, Vidya Kadashetti, Dler H. Hussein Kadir, Sanjay Kalra, Arun Kamireddy, Kehinde Kazeem Kanmodi, Rami S Kantar, Faizan Zaffar Kashoo, Gbenga A Kayode, Shemsu Kedir, Tibebeselassie S Keflie, Ajmal Khan, Maseer Khan, Vishnu Khanal, Shaghayegh Khanmohammadi, Khaled Khatab, Moawiah Mohammad Khatatbeh, Mahalaqua Nazli Khatib, Feriha Fatima Khidri, Kwanghyun Kim, Min Seo Kim, Adnan Kisa, Farzad Kompani, Isaac Koomson, Kewal Krishan, Mukhtar Kulimbet, Dewesh Kumar, G Anil Kumar, Nithin Kumar, Vijay Kumar, Almagul Kurmanova, Maria Dyah Kurniasari, Dian Kusuma, Chandrakant Lahariya, Kamaluddin Latief, Minh Huu Nhat Le, Nhi Huu Hanh Le, Sang-woong Lee, Seung Won Lee, Yo Han Lee, Virendra S Ligade, Stephen S Lim, Jialing Lin, Jue Liu, Xuefeng Liu, Rakesh Lodha, José Francisco López-Gil, Surbala Devi Lourembam, Zheng Feei Ma, Mahmoud Mabrok, Kashish Malhotra, Ahmad Azam Malik, Vahid Mansouri, Emmanuel Manu, Melvin Barrientos Marzan, Roy Rillera Marzo, Sammer Marzouk, Medha Mathur, Rita Mattiello, Rishi P Mediratta, Riffat Mehboob, Kala M Mehta, Tesfahun Mekene Meto, Tomislav Mestrovic, Sachith Mettananda, Tomasz Miazgowski, Giuseppe Minervini, Mojgan Mirghafourvand, Andreea Mirica, Jama Mohamed, Nouh Saad Mohamed, Sakineh Mohammad-Alizadeh-Charandabi, Abdollah Mohammadian-Hafshejani, Shafiu Mohammed, Ali H Mokdad, Lorenzo Monasta, Mohammad Ali Moni, Sumaira Mubarik, Sumoni Mukherjee, Sileshi Mulatu, Francesk Mulita, Christopher J L Murray, Ghulam Mustafa, Ayoub Nafei, Ganesh R Naik, Zuhair S Natto, Javaid Nauman, Samidi Nirasha Kumari Navaratna, Biswa Prakash Nayak, Amanuel Tebabal Nega, Samata Nepal, Henok Biresaw Netsere, Georges Nguefack-Tsague, Dang Nguyen, The Phuong Nguyen, Robina Khan Niazi, Ali Nikoobar, Lawrence Achilles Nnyanzi, Shuhei Nomura, Mehran Nouri, Chisom Adaobi Nri-Ezedi, Dieta Nurrika, Sylvester Dodzi Nyadanu, Chimezie Igwegbe Nzoputam, Ogochukwu Janet Nzoputam, Ismail A Odetokun, Hassan Okati-Aliabad, Akinkunmi Paul Okekunle, Osaretin Christabel Okonji, Bolajoko Olubukunola Olusanya, Jacob Olusegun Olusanya, Uchechukwu Levi Osuagwu, Amel Ouyahia, Mahesh P, Jagadish Rao Padubidri, Anca Pantea Stoian, Romil R Parikh, Jay Patel, Shankargouda Patil, Shrikant Pawar, Gavin Pereira, Arokiasamy Perianayagam, Fanny Emily Petermann-Rocha, Hoang Nhat Pham, Hoang Tran Pham, My Kieu Phan, Jalandhar Pradhan, Pranil Man Singh Pradhan, Akila Prashant, Jagadeesh Puvvula, Ibrahim Qattea, Pankaja Raghav, Md. Mosfequr Rahman, Mosiur Rahman, Muhammad Aziz Rahman, Amir Masoud Rahmani, Masoud Rahmati, Rajesh Kumar Rai, Ivano Raimondo, Sathish Rajaa, Rayan Rajabi, Mahmoud Mohammed Ramadan, Chitra Ramasamy, Shakthi Kumaran Ramasamy, Chhabi Lal Ranabhat, Chythra R Rao, Sowmya J Rao, Davide Rasella, Mamunur Rashid, Devarajan Rathish, Santosh Kumar Rauniyar, David Laith Rawaf, Salman Rawaf, Nazila Rezaei, Mohsen Rezaeian, Thales Philipe Rodrigues da Silva, Jefferson Antonio Buendia Rodriguez, Peter Rohloff, Debby Syahru Romadlon, Bedanta Roy, Shubhanjali Roy, Cameron John Sabet, Kabir P Sadarangani, Basema Ahmad Saddik, Umar Saeed, Amene Saghazadeh, Dominic Sagoe, Narjes Saheb Sharif-Askari, Amirhossein Sahebkar, Pragyan Monalisa Sahoo, Yoseph Leonardo Samodra, Abdallah M Samy, Rama Krishna Sanjeev, Senthilkumar Sankararaman, Milena M Santric-Milicevic, Jacob Owusu Sarfo, Yaser Sarikhani, Tanmay Sarkar, Gargi Sachin Sarode, Sachin C Sarode, Benn Sartorius, Jennifer Saulam, Monika Sawhney, Ganesh Kumar Saya, Christophe Schinckus, Art Schuermans, Ashenafi Kibret Sendekie, Subramanian Senthilkumaran, Yashendra Sethi, Allen Seylani, Shazlin Shaharudin, Samiah Shahid, Masood Ali Shaikh, Sunder Sham, Muhammad Aaqib Shamim, Mohd Shanawaz, Mohammed Shannawaz, Nigussie Tadesse Sharew, Vishal Sharma, Pavanchand H Shetty, Aminu Shittu, Ivy Shiue, Seyed Afshin Shorofi, Emmanuel Edwar Siddig, Mithun Sikdar, Luís Manuel Lopes Rodrigues Silva, Harmanjit Singh, Jasvinder A Singh, Kalpana Singh, Surjit Singh, Shipra Solanki, Mansi Soni, Reed J D Sorensen, Muhammad Suleman, Desy Sulistiyorini, Chandan Kumar Swain, Seyyed Mohammad Tabatabaei, Seyed-Amir Tabatabaeizadeh, Mohammad Tabish, Jacques Lukenze Tamuzi, Birhan Tsegaw Taye, Wegayehu Zeneb Teklehaimanot, Abainash Tekola, Mohamad-Hani Temsah, Rekha Thapar, Jansje Henny Vera Ticoalu, Tenaw Yimer Tiruye, Mariya Vladimirovna Titova, Sojit Tomo, Marcos Roberto Tovani-Palone, Quynh Thuy Huong Tran, Thang Huu Tran, Nguyen Tran Minh Duc, Aristidis Tsatsakis, Abdul Rohim Tualeka, Saeed Ullah, Shahid Ullah, Muhammad Umair, Bhaskaran Unnikrishnan, Era Upadhyay, Jibrin Sammani Usman, Jef Van den Eynde, Siavash Vaziri, Balachandar Vellingiri, Vasily Vlassov, Gebeyaw Biset Wagaw, Yanzhong Wang, Felicia Wu, Hong Xiao, Vikas Yadav, Galal Yahya, Dong Keon Yon, Naohiro Yonemoto, Chuanhua Yu, Sojib Bin Zaman, Iman Zare, Michael Zastrozhin, Mohammed G M Zeariya, Salih M Mustafa Salih Zebari, Claire Chenwen Zhong, Nicholas J Kassebaum, Robert C Reiner

**Affiliations:** A Institute for Health Metrics and Evaluation, University of Washington, Seattle, WA, USA; B Department of Health Metrics Sciences, School of Medicine, University of Washington, Seattle, WA, USA; C Department of Global Health, University of Washington, Seattle, WA, USA; D Department of Nursing, Al Zaytoonah University of Jordan, Amman, Jordan; E Department of Medicine, University of Sétif Algeria, Sétif, Algeria; F Department of Health, University of Sétif Algeria, Sétif, Algeria; G Department of Physiotherapy, Bayero University Kano, Kano, Nigeria; H Department of Physiotherapy, Federal University Wukari, Wukari, Nigeria; I Department of Midwifery, Dilla University, Dilla, Ethiopia; J Department of Emergency Medicine, Zanjan University of Medical Sciences, Zanjan, Iran; K Department of Midwifery, Bahir Dar University, Bahir Dar, Ethiopia; L Department of Family and Community Health, University of Health and Allied Sciences, Ho, Ghana; M School of Population Health, University of New South Wales, Sydney, NSW, Australia; N Research Center for Immunodeficiencies, Tehran University of Medical Sciences, Tehran, Iran; O Department of Medical Biochemistry and Biophysics, Karolinska Institute, Stockholm, Sweden; P Department of Biomedical Science, Arba Minch University, Arba Minch, Ethiopia; Q Department of Biochemistry and Molecular Medicine, Alfaisal University, Riyadh, Saudi Arabia; R College of Graduate Health Sciences, University of Tennessee, Memphis, TN, USA; S Department of Human Anatomy, Federal University Dutse, Dutse, Nigeria; T Department of Anatomy, Bayero University Kano, Kano, Nigeria; U College of Medicine and Health Sciences, Bahir Dar University, Bahir Dar, Ethiopia; V School of Medicine, University of Sydney, Sydney, NSW, Australia; W Centre for Social Research in Health, University of New South Wales, Sydney, NSW, Australia; X Menzies School of Health Research, Charles Darwin University, Darwin, NT, Australia; Y Department of Obstetrics and Gynecology, University of Texas Medical Branch, Galveston, TX, USA; Z Department of Biochemistry, Osun State University, Osogbo, Nigeria; AA Department of Public Health, Universitas Padjadjaran (Padjadjaran University), Bandung, Indonesia; AB Department of Epidemiology and Biostatistics, University of Health and Allied Sciences, Ho, Ghana; AC Department of Life Sciences, University of Management and Technology, Lahore, Pakistan; AD Department of Community Medicine, King Edward Memorial Hospital, Lahore, Pakistan; AE Department of Public Health, Public Health Institute, Lahore, Pakistan; AF MM College of Pharmacy, Maharishi Markandeshwar (Deemed to be University), Ambala, India; AG College of Medicine, Shaqra University, Shaqra, Saudi Arabia; AH School of Nursing, University of Jordan, Amman, Jordan; AI Department of Health and Biological Sciences, Abasyn University, Peshawar, Pakistan; AJ Department of Natural Sciences, Lebanese American University, Beirut, Lebanon; AK School of Medicine, Tehran University of Medical Sciences, Tehran, Iran; AL Institute of Endemic Diseases, University of Khartoum, Khartoum, Sudan; AM Swiss Tropical and Public Health Institute, University of Basel, Basel, Switzerland; AN Department of Biosciences, COMSATS Institute of Information Technology, Islamabad, Pakistan; AO College of Nursing, Majmaah University, Al Majmaah, Saudi Arabia; AP Department of Medicine, Rawalpindi Medical University, Rawalpindi, Pakistan; AQ National Nutrition and Food Technology Research Institute, Shahid Beheshti University of Medical Sciences, Tehran, Iran; AR Faculty of Medicine and Public Health, Jenderal Soedirman University, Purwokerto, Indonesia; AS Division of Public Health Sciences, Washington University in St. Louis, St. Louis, MO, USA; AT Fundamentals and Administration Department, Sultan Qaboos University, Muscat, Oman; AU Preventive Dentistry Department, Jouf University, Sakaka, Saudi Arabia; AV Department of Community and Mental Health, Al al-Bayt University, Mafraq, Jordan; AW Pediatric Intensive Care Unit, King Saud University, Riyadh, Saudi Arabia; AX Department of Medical Rehabilitation (Physiotherapy), University of Maiduguri, Maiduguri, Nigeria; AY Nethersole School of Nursing, The Chinese University of Hong Kong, Hong Kong, China; AZ Department of Biosciences, Jamia Millia Islamia, New Delhi, India; BA Center for Biotechnology and Microbiology, University of Swat, Swat, Pakistan; BB Department of Geography, Sultan Qaboos University, Muscat, Oman; BC Department of Medicine, Nazarbayev University, Astana, Kazakhstan; BD Department of Parasitology, University of Malaya, Kuala Lumpur, Malaysia; BE Department of Parasitology, Sana'a University, Sana'a, Yemen; BF Department of Emergency Medicine, Sana'a University, Sanaa, Yemen; BG Pediatric Emergency Medicine Department, Drexel University, Philadelphia, PA, USA; BH Department of Family and Community Medicine, University of Jeddah, Jeddah, Saudi Arabia; BI Institute of Molecular Biology and Biotechnology, The University of Lahore, Lahore, Pakistan; BJ Faculty of Health Sciences, Equator University of Science and Technology, Uganda, Masaka, Uganda; BK Research Group in Health Economics, Universidad de Cartagena (University of Cartagena), Cartagena, Colombia; BL Research Group in Hospital Management and Health Policies, Universidad de la Costa (University of the Coast), Barranquilla, Colombia; BM Department of Rehabilitation Sciences, Jordan University of Science and Technology, Irbid, Jordan; BN Department of Pediatrics, Cleveland Clinic, Cleveland, OH, USA; BO Department of Health and Wellbeing, African Population and Health Research Center, Nairobi, Kenya; BP Department of Pharmacology, All India Institute of Medical Sciences, Bhubaneswar, India; BQ Department of Epidemiology, Bahir Dar University, Bahir Dar, Ethiopia; BR Department of Environmental and Occupational Health, University of Medical Sciences, Ondo, Ondo, Nigeria; BS Regenerative Medicine, Organ Procurement and Transplantation Multi-disciplinary Center, Guilan University of Medical Sciences, Rasht, Iran; BT Rural Health Research Institute, Charles Sturt University, Orange, NSW, Australia; BU Health Management and Economics Research Center, Iran University of Medical Sciences, Tehran, Iran; BV Department of Applied Mathematics, University of Washington, Seattle, WA, USA; BW College of Art and Science, Ottawa University, Surprise, AZ, USA; BX School of Life Sciences, Arizona State University, Tempe, AZ, USA; BY University College of Medicine & Dentistry, The University of Lahore, Lahore, Pakistan; BZ Institute for Biomedical Problems, Russian Academy of Sciences, Moscow, Russia; CA Department of Physiotherapy, University of Sharjah, Sharjah, United Arab Emirates; CB Department of Physiotherapy, Manipal Academy of Higher Education, Manipal, India; CC Department of Public Health, Kazakh National Medical University, Almaty, Kazakhstan; CD Department of Clinical Disciplines, Al Farabi Kazakh National University, Almaty, Kazakhstan; CE School of Health and Social Development, Deakin University, Melbourne, VIC, Australia; CF Department of Immunology, Zanjan University of Medical Sciences, Zanjan, Iran; CG Faculty of Nursing, Philadelphia University, Amman, Jordan; CH School of Nursing and Public Health, University of KwaZulu-Natal, Durban, South Africa; CI Flinders University, Adelaide, SA, Australia; CJ Institute of Biotechnology and Genetic Engineering, The University of Agriculture, Peshawar, Pakistan; CK Department of Population Medicine, Qatar University, Doha, Qatar; CL Clinical Research Center, Nanjing Children's Hospital, Nanjing, China; CM Center for Clinical Research and Prevention, Bispebjerg University Hospital, Frederiksberg, Denmark; CN Department of Non-communicable Diseases, Bangladesh University of Health Sciences, Dhaka, Bangladesh; CO Miller School of Medicine, University of Miami, Miami, FL, USA; CP Department of Public and Environmental Health, University of The Gambia, Banjul, The Gambia; CQ Department of Epidemiology, University of Florida, Gainesville, FL, USA; CR University Institute of Food Science and Technology, The University of Lahore, Lahore, Pakistan; CS Department of Veterinary Physiology and Biochemistry, University of Ilorin, Ilorin, Nigeria; CT Barcelona Institute for Global Health, ISGlobal Instituto de Salud Global de Barcelona, Barcelona, Spain; CU Catalan Institution for Research and Advanced Studies (ICREA), Barcelona, Spain; CV Non-communicable Diseases Research Center, Tehran University of Medical Sciences, Tehran, Iran; CW School of Medicine, Iran University of Medical Sciences, Tehran, Iran; CX Department of Community Medicine and Family Medicine, All India Institute of Medical Sciences, Bhubaneswar, India; CY School of the Environment, Yale University, New Haven, CT, USA; CZ School of Health Policy and Management, Korea University, Seoul, South Korea; DA Department of Pediatrics, Isfahan University of Medical Sciences, Isfahan, Iran; DB Department of Pediatric Neurology, Tehran University of Medical Sciences, Tehran, Iran; DC Department of Community and Family Medicine, All India Institute of Medical Sciences, Rishikesh, India; DD Community Health Department, University of South Wales, South Wales, UK; DE Global Health Neurology Lab, NSW Brain Clot Bank, Sydney, NSW, Australia; DF Division of Cerebrovascular Medicine and Neurology, National Cerebral and Cardiovascular Center, Suita, Japan; DG Translational and Clinical Research Institute, Newcastle University, Newcastle upon Tyne, UK; DH Laboratory of Translational Medicine and Nanotherapeutics, Central University of Punjab, Bathinda, India; DI Department of Community and Family Medicine, All India Institute of Medical Sciences, Deoghar, India; DJ Department of Community and Family Medicine, All India Institute of Medical Sciences, Ramanathapuram, India; DK Department of Biomedical Sciences, Nazarbayev University, Astana, Kazakhstan; DL Department of Anesthesia and Critical Care Medicine, Johns Hopkins University, Baltimore, MD, USA; DM Unit of Hygiene and Public Health, Romagna Local Health Authority, Forlì-Cesena, Italy; DN Interdisciplinary Research Center for Health Science, Sant'Anna School of Advanced Studies, Pisa, Italy; DO Clinical Nutrition Department, Jazan University, Jazan, Saudi Arabia; DP Department of Epidemiology and Biostatistics, Semey Medical University (SMU), Semey, Kazakhstan; DQ Department of Community Medicine, Datta Meghe Institute of Medical Sciences, Sawangi, India; DR Department of Biology, Imam Mohammad Ibn Saud Islamic University, Riyadh, Saudi Arabia; DS Midwifery Department, Debre Berhan University, Debre Berhan, Ethiopia; DT Faculty of Humanities and Health Sciences, Curtin University, Miri, Malaysia; DU Division of Plastic Surgery, University of Wisconsin, Madison, WI, USA; DV Department of Community Medicine, Jawaharlal Nehru Medical College, Wardha, India; DW Department of Paediatric Surgery, Federal Medical Centre, Umuahia, Nigeria; DX Department of Pediatrics, University of Washington, Seattle, WA, USA; DY Life and Health Sciences Research Institute (ICVS), University of Minho, Braga, Portugal; DZ Institute for Research and Innovation in Health (i3S), University of Porto, Porto, Portugal; EA School of Nursing, Federal University of Minas Gerais, Belo Horizonte, Brazil; EB Department of Community Medicine, Ahmadu Bello University, Zaria, Nigeria; EC Public Health Foundation of India, Gurugram, India; ED Department of Public Health, Haramaya University, Harar, Ethiopia; EE Department of Pediatrics, Tanta University, Tanta, Egypt; EF Department of Public Health, National University of Colombia, Bogota, Colombia; EG Dirección de Nutrición, Salvador Zubiran National Institute of Medical Sciences and Nutrition, Mexico City, Mexico; EH Chettinad Hospital & Research Institute, Chettinad Academy of Research and Education, Chennai, India; EI JSS Medical College Department of Biochemistry, Jagadguru Sri Shivarathreeswara Academy of Health Education and Research, Mysuru, India; EJ Faculty of Management, Bucharest University of Economic Studies, Bucharest, Romania; EK Department of Medicine, Pham Ngoc Thach University of Medicine, Ho Chi Minh City, Viet Nam; EL Ateneo Center for Research and Innovation, Ateneo De Manila University, Pasig City, Philippines; EM Demography and Health, London School of Hygiene & Tropical Medicine, London, UK; EN Department of Public Health and Community Medicine, Tanta University, Tanta city, Egypt; EO School of Public Health, Texila American University, Guyana, Guyana; EP Pediatrics and Neonatology Department, Kafr Elshiekh University, Kafr Elshiekh, Egypt; EQ College of Medicine, Korea University, Seoul, South Korea; ER Houston Methodist Hospital, Houston, TX, USA; ES Department of Public Health, Wachemo University, Hossana, Ethiopia; ET Biomedical Informatics and Medical Statistics Department, Alexandria University, Alexandria, Egypt; EU Satcher Health Leadership Institute, Morehouse School of Medicine, Atlanta, GA, USA; EV School of Medicine, Emory University, Atlanta, GA, USA; EW Department of Social Policy and Action, Escola Superior de Saúde do Alcoitão, Alcabideche, Portugal; EX European Network of Occupational Therapy in Higher Education, Vienna, Austria; EY Institute of Public Health, Charité Universitätsmedizin Berlin (Charité Medical University Berlin), Berlin, Germany; EZ Department of Cell Biology and Biotechnology, K.A. Timiryazev Institute of Plant Physiology, Moscow, Russia; FA MBBS, University of Sharjah, Sharjah, United Arab Emirates; FB School of Nursing and Midwifery, Edith Cowan University, Perth, WA, Australia; FC School of Population Health, Curtin University, Perth, WA, Australia; FD Department of Public Health, Debre Berhan University, Debre Berhan, Ethiopia; FE Department of Public Health, Menelik II Medical and Health Science College, Addis Ababa, Ethiopia; FF Cancer Research Center, Shahid Beheshti University of Medical Sciences, Tehran, Iran; FG Department of Nursing, Aksum University, Aksum, Ethiopia; FH Department of Health Systems and Policy Research, Indian Institute of Public Health, Gandhinagar, India; FI Maternal, Child and Adolescent Nutrition Department, National Institute of Public Health, Cuernavaca, Mexico; FJ Institute of Public Health, United Arab Emirates University, Al Ain, United Arab Emirates; FK Department of Public Health and Preventive Medicine, Charles University, Prague, Czech Republic; FL Department of Epidemiology and Biostatistics, Anhui Medical University, Hefei, China; FM Department of Clinical Science, University Of Sulaimani, Sulaimani, Iraq; FN Department of Community Medicine, University of Peradeniya, Kandy, Sri Lanka; FO Nanyang Maternal and Child Health Care Hospital, Nanyang Central Hospital, Nanyang, China; FP Department of Public Health, Torrens University Australia, Melbourne, VIC, Australia; FQ Department of Epidemiology and Biostatistics, University of South Carolina, Columbia, SC, USA; FR Centre for Noncommunicable Diseases and Nutrition, BRAC University, Dhaka, Bangladesh; FS Biochemistry Department, Ain Shams University, Cairo, Egypt; FT Department of Epidemiology, University of Michigan School of Public Health, Ann Arbor, MI, USA; FU Department of Critical Care and Emergency Nursing, Zanjan University of Medical Sciences, Zanjan, Iran; FV School of Public Health, Dilla University, Dilla, Ethiopia; FW Department of Advanced Nursing, Universitas Airlangga (Airlangga University), Surabaya, Indonesia; FX School of Nursing and Midwivery, La Trobe University, Bundoora, VIC, Australia; FY Department of Zoology and Entomology, Al-Azhar University, Cairo, Egypt; FZ Department of Community Medicine, Federal University Teaching Hospital, Lafia, Nigeria; GA Department of Epidemiology and Community Medicine, Federal University of Lafia, Lafia, Nigeria; GB Institute of Pharmaceutical Sciences, University of Veterinary and Animal Sciences, Lahore, Pakistan; GC Department of Pharmacy Administration and Clinical Pharmacy, Xian Jiaotong University, Xian, China; GD Department of Medicine, University of Alberta, Edmonton, AB, Canada; GE Department of Microbiology, Taiz University, Taiz, Yemen; GF School of Medicine, Nankai University, Tianjin, China; GG Kasturba Medical College, Mangalore, Manipal Academy of Higher Education, Manipal, India; GH Department of Statistics, Shahjalal University of Science and Technology, Sylhet, Bangladesh; GI School of Health and Society, University of Wollongong, Wollongong, NSW, Australia; GJ Department of Legal Medicine and Bioethics, Carol Davila University of Medicine and Pharmacy, Bucharest, Romania; GK Department of Clinical Legal Medicine, National Institute of Legal Medicine Mina Minovici, Bucharest, Romania; GL School of Public Health, University of Sydney, Sydney, NSW, Australia; GM Maternal and Child Health Division, International Centre for Diarrhoeal Disease Research, Bangladesh, Dhaka, Bangladesh; GN Department of Biological Sciences and Chemistry (DBSC), University of Nizwa, Nizwa, Oman; GO Clinical Governance and Quality Improvement Head, Salale University, Gerba Guracha, Ethiopia; GP International Master Program for Translational Science, Taipei Medical University, Taipei, Taiwan; GQ Department of Occupational Safety and Health, China Medical University, Taiwan, Taichung, Taiwan; GR Department of Occupational Therapy, Asia University, Taiwan, Taichung, Taiwan; GS Department of Health Promotion and Education, University of Ibadan, Ibadan, Nigeria; GT Institute of Health Research, University of Health and Allied Sciences, Ho, Ghana; GU Independent Researcher, Cairo, Egypt; GV School of Pharmacy, BRAC University, Dhaka, Bangladesh; GW Research and Publication Department, World Health Organization (WHO), Dhaka, Bangladesh; GX Institute for Physical Activity and Nutrition, Deakin University, Burwood, VIC, Australia; GY Department of Physical Medicine and Rehabilitation, Université Paris Cité, Paris, France; GZ Research and Development Unit, Biomedical Research Networking Center for Mental Health Network (CiberSAM), Barcelona, Spain; HA UNESCO-TWAS Section of Economic & Social Sciences, Humanities & Arts, The World Academy of Sciences UNESCO-TWAS, Trieste, Italy; HB Shaanxi University of Technology, Hanzhong, China; HC Department of Biochemistry, Government Medical College, Mysuru, India; HD Postgraduate Institute of Medicine, University of Colombo, Colombo, Sri Lanka; HE Faculty of Graduate Studies, Institute for Violence and Injury Prevention, Colombo, Sri Lanka; HF Department of Orthopedics, Wuhan University, Wuhan, China; HG Department of Biomedical Sciences, City University of Hong Kong, Hong Kong, China; HH School of Public Health, SRMIST, Sri Ramaswamy Memorial Institute of Science and Technology, Chennai, India; HI Department of Community Medicine, Manipal Academy of Higher Education, Mangalore, India; HJ Minimally Invasive Surgery Research Center, Iran University of Medical Sciences, Tehran, Iran; HK Department of Oral and Maxillofacial Pathology, Krishna Vishwa Vidyapeeth Deemed to be University, Karad, India; HL Department of Statistics, Salahaddin University, Erbil, Iraq; HM Department of Business Administrations, Cihan University-Erbil, Erbil, Iraq; HN Department of Endocrinology, Bharti Hospital Karnal, Karnal, India; HO University Centre for Research and Development, Chandigarh University, Mohali, India; HP Russell H. Morgan Department of Radiology and Radiological Science, Johns Hopkins University, Baltimore, MD, USA; HQ Office of the Executive Director, Cephas Health Research Initiative Inc, Ibadan, Nigeria; HR The Hansjörg Wyss Department of Plastic and Reconstructive Surgery, NYU Langone Health, New York, NY, USA; HS Cleft Lip and Palate Surgery Division, Global Smile Foundation, Norwood, MA, USA; HT Department of Physical Therapy and Health Rehabilitation, Majmaah University, Majmaah, Saudi Arabia; HU International Research Center of Excellence, Institute of Human Virology Nigeria, Abuja, Nigeria; HV Julius Centre for Health Sciences and Primary Care, Utrecht University, Utrecht, Netherlands; HW Department of Public Health, Werabe University, Werabe, Ethiopia; HX Institute of Biological Chemistry and Nutrition, University Hohenheim, Stuttgart, Germany; HY Natural and Medical Sciences Research Center, University of Nizwa, Nizwa, Oman; HZ Epidemiology Program, Jazan University, Jazan, Saudi Arabia; IA Department of Health, Nepal Development Society, Chitwan, Nepal; IB Department of Preventable Non Communicable Disease, Menzies School of Health Research, Alice Springs, NT, Australia; IC Department of Epidemiology, Non-Communicable Diseases Research Center (NCDRC), Tehran, Iran; ID College of Health, Wellbeing and Life Sciences, Sheffield Hallam University, Sheffield, UK; IE College of Arts and Sciences, Ohio University, Zanesville, OH, USA; IF Department of Basic Medical Sciences, Yarmouk University, Irbid, Jordan; IG Global Consortium for Public Health Research, Datta Meghe Institute of Higher Education and Research, Wardha, India; IH Department of Biochemistry, Liaquat University Of Medical and Health Sciences, Jamshoro, Pakistan; II School of Medicine, Creighton University, Omaha, NE, USA; IJ Cardiovascular Disease Initiative, Broad Institute of MIT and Harvard, Cambridge, MA, USA; IK Massachusetts General Hospital, Boston, MA, USA; IL School of Health Sciences, Kristiania University College, Oslo, Norway; IM Department of International Health and Sustainable Development, Tulane University, New Orleans, LA, USA; IN Children's Medical Center, Tehran University of Medical Sciences, Tehran, Iran; IO Centre for the Business and Economics of Health, The University of Queensland, Brisbane, QLD, Australia; IP Department of Anthropology, Panjab University, Chandigarh, India; IQ Research and Publication Activity Division, Kazakh National Medical University, Almaty, Kazakhstan; IR Center of Medicine and Public Health, Asfendiyarov Kazakh National Medical University, Almaty, Kazakhstan; IS Department of Community Medicine, Rajendra Institute of Medical Sciences, Ranchi, India; IT Department of Economics, Manipal University, Jaipur, Jaipur, India; IU Department of Clinical Subjects, Al Farabi Kazakh National University, Almaty, Kazakhstan; IV Faculty of Medicine and Health Science, Universitas Kristen Satya Wacana (Satya Wacana Christian University), Salatiga, Indonesia; IW School of Nursing, Taipei Medical University, Taipei, Taiwan; IX Department of Public Health and Epidemiology, Khalifa University of Science and Technology, Abu Dhabi, United Arab Emirates; IY Faculty of Public Health, University of Indonesia, Depok, Indonesia; IZ Division of Evidence Synthesis, Foundation for People-centric Health Systems, New Delhi, India; JA Division of Lifestyle Medicine, Centre for Health: The Specialty Practice, New Delhi, India; JB Centre for Family Welfare, University of Indonesia, Depok, Indonesia; JC Department of Global Health and Health Security, Taipei Medical University, Taipei, Taiwan; JD International Ph.D. Program in Medicine, Taipei Medical University, Taipei, Taiwan; JE Research Center for Artificial Intelligence in Medicine, Taipei Medical University, Taipei, Taiwan; JF Faculty of Medicine, University of Medicine and Pharmacy at Ho Chi Minh City, Ho Chi Minh City, Viet Nam; JG Department of Cardiovascular Research, Methodist Hospital, Merrillville, IN, USA; JH Pattern Recognition and Machine Learning Lab, Gachon University, Seongnam, South Korea; JI Department of Precision Medicine, Sungkyunkwan University, Suwon-si, South Korea; JJ Department of Preventive Medicine, Korea University, Seoul, South Korea; JK Department of Pharmaceutical Regulatory Affairs and Management, Manipal Academy of Higher Education, Manipal, India; JL International Centre for Future Health Systems, University of New South Wales, Sydney, NSW, Australia; JM Department of Epidemiology and Biostatistics, Peking University, Beijing, China; JN Lerner Research Institute, Cleveland Clinic, Cleveland, OH, USA; JO Department of Quantitative Health Science, Case Western Reserve University, Cleveland, OH, USA; JP Department of Paediatrics, All India Institute of Medical Sciences, New Delhi, India; JQ School of Medicine, Universidad Espíritu Santo, Samborondón, Ecuador; JR Vicerrectoría de Investigación y Postgrado, Universidad de Los Lagos, Osorno, Chile; JS Ashok & Rita Patel Institute of Physiotherapy, Charotar University of Science and Technology, Anand, India; JT Centre for Public Health and Wellbeing, University of the West of England, Bristol, UK; JU Faculty of Veterinary Medicine, Suez Canal University, Ismailia, Egypt; JV Department of Microbiology and Parasitology, King Salman International University, South of Sinai, Egypt; JW Rama Medical College Hospital and Research Centre, Uttar Pradesh, India; JX Institute of Applied Health Research, University of Birmingham, Birmingham, UK; JY Rabigh Faculty of Medicine, King Abdulaziz University, Jeddah, Saudi Arabia; JZ Digestive Diseases Research Institute, Tehran University of Medical Sciences, Tehran, Iran; KA Department of Population and Behavioural Sciences, University of Health and Allied Sciences, Ho, Ghana; KB Centre for Alcohol Policy Research (CAPR), La Trobe University, Melbourne, VIC, Australia; KC Faculty of Humanities and Health Sciences, Curtin University, Sarawak, Malaysia; KD Jeffrey Cheah School of Medicine and Health Sciences, Monash University, Subang Jaya, Malaysia; KE Medical Scientist Training Program, Northwestern University, Chicago, IL, USA; KF Department of Community Medicine, Geetanjali Medical College and Hospital in Udaipur India, Udaipur, India; KG Department of Social Medicine, Federal University of Rio Grande do Sul, Porto Alegre, Brazil; KH Division of Pediatric Hospital Medicine, Stanford University, Palo Alto, CA, USA; KI National Heart, Lung and Blood Institute, Bethesda, MD, USA; KJ Research and Development Department, Lahore Medical Research Center, Lahore, Pakistan; KK Department of Epidemiology and Biostatistics, University of California San Francisco, San Francisco, CA, USA; KL Department of Public Health, Arba Minch University, Arba Minch, Ethiopia; KM University Centre Varazdin, University North, Varazdin, Croatia; KN Department of Paediatrics, University of Kelaniya, Ragama, Sri Lanka; KO University Paediatrics Unit, Colombo North Teaching Hospital, Ragama, Sri Lanka; KP Department of Propedeutics of Internal Diseases & Arterial Hypertension, Pomeranian Medical University, Szczecin, Poland; KQ Multidisciplinary Department of Medical-Surgical and Dental Specialties, University of Campania Luigi Vanvitelli, Naples, Italy; KR Saveetha Dental College and Hospitals, Saveetha University, Chennai, India; KS Faculty of Nursing and Midwifery, Tabriz University of Medical Sciences, Tabriz, Iran; KT Department of Statistics and Econometrics, Bucharest University of Economic Studies, Bucharest, Romania; KU College of Applied and Natural Science, University of Hargeisa, Hargeisa, Somalia; KV Molecular Biology Unit, Sirius Training and Research Centre, Khartoum, Sudan; KW Bio-Statistical and Molecular Biology Department, Sirius Training and Research Centre, Khartoum, Sudan; KX Social Determinants of Health Research Center, Tabriz University of Medical Sciences, Tabriz, Iran; KY Midwifery Department, Tabriz University of Medical Sciences, Tabriz, Iran; KZ Modeling in Health Research Center, Shahrekord University of Medical Sciences, Shahrekord, Iran; LA Health Systems and Policy Research Unit, Ahmadu Bello University, Zaria, Nigeria; LB Heidelberg Institute of Global Health (HIGH), Heidelberg University, Heidelberg, Germany; LC Clinical Epidemiology and Public Health Research Unit, Burlo Garofolo Institute for Maternal and Child Health, Trieste, Italy; LD AI & Cyber Futures Institute, Charles Sturt University, Bathurst, NSW, Australia; LE The University of Queensland, Brisbane, QLD, Australia; LF Unit of Pharmacotherapy, Epidemiology and Economics, University of Groningen (Rijksuniversiteit Groningen), Groningen, Netherlands; LG Department of Epidemiology and Biostatistics, Wuhan University, Wuhan, China; LH Knowledge Management Department, Prahlad Omkarwati Foundation (POF), Mumbai, India; LI Changescape Consulting, Independent Consultant, New Delhi, India; LJ Department of Pediatrics and Child Health Nursing, Bahir Dar University, Bahir Dar, Ethiopia; LK Department of Surgery, General University Hospital of Patras, Patras, Greece; LL Faculty of Medicine, University of Thessaly, Larissa, Greece; LM Department of Pediatrics & Pediatric Pulmonology, Institute of Mother & Child Care, Multan, Pakistan; LN Elderly Health Research Center, Research and Academic Institution, Tehran, Iran; LO College of Medicine and Public Health, Flinders University, Adelaide, SA, Australia; LP Department of Computer Science and IT, Torrens University, Adelaide, SA, Australia; LQ Department of Dental Public Health, King Abdulaziz University, Jeddah, Saudi Arabia; LR Department of Health Policy and Oral Epidemiology, Harvard University, Boston, MA, USA; LS College of Medicine and Health Sciences, United Arab Emirates University, Al Ain, United Arab Emirates; LT Department of Circulation and Medical Imaging, Norwegian University of Science and Technology, Trondheim, Norway; LU Amity Institute of Forensic Sciences, Amity University, Noida, India; LV Department of Community Medicine, Lumbini Medical College, Palpa, Nepal; LW School of Nursing, University of Gondar, Gondar, Ethiopia; LX Department of Public Health, University of Yaoundé I, Yaoundé, Cameroon; LY Harvard T.H. Chan School of Public Health, Harvard University, Cambridge, MA, USA; LZ Department of Medical Engineering, University of South Florida, Tampa, FL, USA; MA Hitotsubashi Institute for Advanced Study (HIAS), Hitotsubashi University, Tokyo, Japan; MB Institute for Cancer Control, National Cancer Center, Chuo-ku, Japan; MC International Islamic University Islamabad, Islamabad, Pakistan; MD Social Determinants of Health Research Center, Shahid Beheshti University of Medical Sciences, Tehran, Iran; ME Center for Public Health, Teesside University, Middlesbrough, UK; MF International Research Institute of Disaster Science (IRIDeS), Tohoku University, Miyagi, Japan; MG Global Research Institute, Keio University, Tokyo, Japan; MH Health Policy Research Center, Shiraz University of Medical Sciences, Shiraz, Iran; MI Health Research Institute, Babol University of Medical Sciences, Babol, Iran; MJ Department of Paediatrics, Nnamdi Azikiwe University, Awka, Nigeria; MK Department of Public Health, Banten School of Health Science, South Tangerang, Indonesia; ML Ministry of Research, Technology and Higher Education, Higher Education Service Institutions (LL-DIKTI) Region IV, Bandung, Indonesia; MM Center of Excellence in Reproductive Health Innovation (CERHI), University of Benin, Benin City, Nigeria; MN Department of Physiology, University of Benin, Edo, Nigeria; MO Department of Physiology, Benson Idahosa University, Benin City, Nigeria; MP Department of Veterinary Public Health and Preventive Medicine, University of Ilorin, Ilorin, Nigeria; MQ Health Promotion Research Center, Zahedan University of Medical Sciences, Zahedan, Iran; MR Department of Food and Nutrition, Seoul National University, Seoul, South Korea; MS College of Medicine, University of Ibadan, Ibadan, Nigeria; MT School of Pharmacy, University of the Western Cape, Cape Town, South Africa; MU Centre for Healthy Start Initiative, Lagos, Nigeria; MV Research Policy & Administration, Centre for Healthy Start Initiative, Lagos, Nigeria; MW School of Medicine, Western Sydney University, Bathurst, NSW, Australia; MX Department of Optometry and Vision Science, University of KwaZulu-Natal, KwaZulu-Natal, South Africa; MY Faculty of Medicine, University Ferhat Abbas of Setif, Sétif, Algeria; MZ Division of Infectious Diseases, University Hospital of Setif, Sétif, Algeria; NA Department of Respiratory Medicine, Jagadguru Sri Shivarathreeswara University, Mysore, India; NB Department of Forensic Medicine and Toxicology, Manipal Academy of Higher Education, Mangalore, India; NC Department of Diabetes, Nutrition and Metabolic Diseases, Carol Davila University of Medicine and Pharmacy, Bucharest, Romania; ND Division of Health Policy and Management, University of Minnesota, Minneapolis, MN, USA; NE Faculty of Medicine and Health, University of Leeds, Leeds, UK; NF College of Dental Medicine, Roseman University of Health Sciences, South Jordan, UT, USA; NG Department of Genetics, Yale University, New Haven, CT, USA; NH School of Population Health, Curtin University, Bentley, WA, Australia; NI Centre for Fertility and Health, Norwegian Institute of Public Health, Oslo, Norway; NJ Social and Economic Survey Research Institute (SESRI), Qatar University, Doha, Qatar; NK Facultad de Medicina (Faculty of Medicine), Universidad Diego Portales (Diego Portales University), Santiago, Chile; NL School of Cardiovascular and Metabolic Health, University of Glasgow, Glasgow, UK; NM Department of Internal Medicine, University of Arizona, Tucson, AZ, USA; NN Department of Cardiovascular Medicine, Mayo Clinic, Rochester, MN, USA; NO Department of Internal Medicine, Weiss Memorial Hospital, Chicago, IL, USA; NP Faculty of Medicine of Nam Can Tho University, University of Medicine, Nam Can Tho University, Viet Nam; NQ Department of Humanities and Social Sciences, National Institute of Technology Rourkela, Rourkela, India; NR Department of Community Medicine and Public Health, Tribhuvan University, Kathmandu, Nepal; NS T.H. Chan School of Public Health, Harvard University, Boston, MA, USA; NT Department of Biochemistry, JSS Academy of Higher Education and Research, Mysuru, India; NU Department of Biostatistics, Epidemiology, and Informatics, University of Pennsylvania, Philadelphia, PA, USA; NV Department of Neonatology, Case Western Reserve University, Akron, OH, USA; NW Department of Community Medicine and Family Medicine, All India Institute of Medical Sciences, Jodhpur, India; NX Department of Population Science and Human Resource Development, University of Rajshahi, Rajshahi, Bangladesh; NY Institute of Health and Wellbeing, Federation University Australia, Berwick, VIC, Australia; NZ School of Nursing and Midwifery, La Trobe University, Melbourne, VIC, Australia; OA Future Technology Research Center, National Yunlin University of Science and Technology, Yunlin, Taiwan; OB Health Service Research and Quality of Life Center (CEReSS), Aix-Marseille University, Marseille, France; OC Society for Health and Demographic Surveillance, Suri, India; OD Institute of Nutrition, Mahidol University, Salaya, Thailand; OE Department of Medical, Surgical and Experimental Sciences, University of Sassari, Sassari, Italy; OF Gynecology and Breast Care Center, Mater Olbia Hospital, Olbia, Italy; OG Department of Community Medicine, Employees' State Insurance Model Hospital, Chennai, India; OH Department of Medicine, Iran University of Medical Sciences, Tehran, Iran; OI Department of Clinical Sciences, University of Sharjah, Sharjah, United Arab Emirates; OJ Department of Cardiology, Mansoura University, Mansoura, Egypt; OK Department of Anatomy, Govt. Siddhartha Medical College, Vijayawada, India; OL Department of Radiology, Stanford University, Stanford, CA, USA; OM Department of Research, Eastern Scientific LLC, Richmond, KY, USA; ON Planetary Health Research Centre (PHRC), Kathmandu, Nepal; OO Department of Community Medicine, Manipal Academy of Higher Education, Manipal, India; OP Department of Oral Pathology, Microbiology and Forensic Odontology, Sharavathi Dental College and Hospital, Shimogga, India; OQ Institute of Collective Health, Federal University of Bahia, Salvador, Brazil; OR Barcelona Institute for Global Health, Barcelona, Spain; OS Unit for Public Health Science, University of Gävle, Sweden, Stockholm, Sweden; OT Department of Family Medicine, Rajarata University of Sri Lanka, Anuradhapura, Sri Lanka; OU Department of Global Health Policy, University of Tokyo, Tokyo, Japan; OV WHO Collaborating Centre for Public Health Education and Training, Imperial College London, London, UK; OW Inovus Medical, St Helens, UK; OX Department of Primary Care and Public Health, Imperial College London, London, UK; OY Academic Public Health England, Public Health England, London, UK; OZ Department of Epidemiology and Biostatistics, Rafsanjan University of Medical Sciences, Rafsanjan, Iran; PA Department of Nursing in Women's Health, Federal University of São Paulo, São Paulo, Brazil; PB Vaccination Research Observatory, Federal University of Minas Gerais, Belo Horizonte, Brazil; PC Department of Pharmacology and Toxicology, University of Antioquia, Medellin, Colombia; PD Warwick Medical School, University of Warwick, Coventry, UK; PE Division of Global Health Equity, Harvard University, Boston, MA, USA; PF Center for Indigenous Health Research, Wuqu' Kawoq Maya Health Alliance, Tecpan, Guatemala; PG Faculty of Nursing, Chulalongkorn University, Bangkok, Thailand; PH Faculty of Medicine, Quest International University Perak, Ipoh, Malaysia; PI Research Department, Indian Institute of Public Health, Delhi, India; PJ Department of Medicine, Georgetown University, Washington, DC, USA; PK Escuela de Kinesiología, Diego Portales University, Santiago de Chile, Chile; PL Universidad Autónoma de Chile, Santiago de Chile, Chile; PM Department of Public Health and Epidemiology, Khalifa University, Abu Dhabi, United Arab Emirates; PN Operational Research Center in Healthcare, Near East University, Nicosia, Turkiye; PO International Center of Medical Sciences Research, Islamabad, Pakistan; PP Department of Psychosocial Science, University of Bergen, Bergen, Norway; PQ Clinical Sciences Department, University of Sharjah, Sharjah, United Arab Emirates; PR Center for Global Health Research, Saveetha University, Chennai, India; PS Biotechnology Research Center, Mashhad University of Medical Sciences, Mashhad, Iran; PT Department of Analytical and Applied Economics, Utkal University, Bhubaneswar, India; PU Institute of Epidemiology and Preventive Medicine, National Taiwan University, Taipei, Taiwan; PV Benang Merah Research Center, Benang Merah Research Center (BMRC), Minahasa Utara, Indonesia; PW Department of Entomology, Ain Shams University, Cairo, Egypt; PX Medical Ain Shams Research Institute (MASRI), Ain Shams University, Cairo, Egypt; PY Department of Pediatrics, SRM Medical College Hospital And Research Centre, Kattankulathur, India; PZ Department of Pediatrics, University Hospitals Rainbow Babies & Children's Hospital, Cleveland, OH, USA; QA Department of Pediatrics, Case Western Reserve University, Cleveland, OH, USA; QB Faculty of Medicine, University of Belgrade, Belgrade, Serbia; QC School of Public Health and Health Management, University of Belgrade, Belgrade, Serbia; QD Department of Health, Physical Education and Recreation, University of Cape Coast, Cape Coast, Ghana; QE Department of Public Health, Jahrom University of Medical Sciences, Jahrom, Iran; QF Department of Food Processing Technology, West Bengal State Council of Technical Education, Malda, India; QG Department of Oral Pathology and Microbiology, Dr. D. Y. Patil Vidyapeeth, Pune (Deemed to be University), Pune, India; QH Faculty of Medicine, The University of Queensland, Brisbane, QLD, Australia; QI Nuffield Department of Medicine, University of Oxford, Oxford, UK; QJ Department of Medical Informatics, Kagawa University, Miki-cho, Japan; QK Food Processing and Nutrition, Karnataka State Akkamahadevi Women's University, Vijayapura, India; QL Department of Public Health Sciences, University of North Carolina at Charlotte, Charlotte, NC, USA; QM Department of Preventive and Social Medicine, Jawaharlal Institute of Postgraduate Medical Education and Research, Puducherry, India; QN Faculty of Business and Computing, University of the Fraser Valley, Abbotsford, BC, Canada; QO Graduate School of Business, ESAN University, Lima, Peru; QP Faculty of Medicine, Katholieke Universiteit Leuven, Leuven, Belgium; QQ Department of Cardiovascular Sciences, Katholieke Universiteit Leuven, Leuven, Belgium; QR Department of Clinical Pharmacy, University of Gondar, Gondar, Ethiopia; QS School of Pharmacy, Curtin University, Perth, WA, Australia; QT Emergency Department, Manian Medical Centre, Erode, India; QU Department of Medicine, Swami Vivekanand Subharti University, Meerut, India; QV National Heart, Lung, and Blood Institute, National Institutes of Health, Rockville, MD, USA; QW School of Health Sciences, Universiti Sains Malaysia, Kota Bharu, Malaysia; QX Research Centre for Health Sciences (RCHS), The University of Lahore, Lahore, Pakistan; QY Independent Consultant, Karachi, Pakistan; QZ Department of Pathology and Laboratory Medicine, Northwell Health, New York, NY, USA; RA Department of Pharmacology, All India Institute of Medical Sciences, Jodhpur, India; RB College of Nursing and Health Sciences, Jazan University, Jazan, Saudi Arabia; RC Amity Institute of Public Health, Amity University, Noida, India; RD Department of Nursing, Debre Berhan University, Debre Berhan, Ethiopia; RE Interdisciplinary Center Psychopathology and Emotion Regulation (ICPE), University of Groningen, Groningen, Netherlands; RF Institute of Forensic Science & Criminology, Panjab University, Chandigarh, India; RG Department of Veterinary Public Health and Preventive Medicine, Usmanu Danfodiyo University, Sokoto, Sokoto, Nigeria; RH Oulu Business School, University of Oulu, Oulu, Finland; RI Martti Ahtisaari Institute, University of Oulu, Oulu, Finland; RJ Department of Medical-Surgical Nursing, Mazandaran University of Medical Sciences, Sari, Iran; RK Department of Nursing and Health Sciences, Flinders University, Adelaide, SA, Australia; RL Unit of Basic Medical Sciences, University of Khartoum, Khartoum, Sudan; RM Department of Medical Microbiology and Infectious Diseases, Erasmus University, Rotterdam, Netherlands; RN Anthropological Survey of India, Anthropological Survey of India, Mysore, India; RO Sport Physical Activity and Health Research & Innovation Center (SPRINT), Polytechnic Institute of Guarda, Guarda, Portugal; RP RISE Health, University of Beira Interior, Covilhã, Portugal; RQ Department of Pharmacology, Government Medical College and Hospital, Chandigarh, India; RR School of Medicine, Baylor College of Medicine, Houston, TX, USA; RS Department of Medicine Service, US Department of Veterans Affairs (VA), Houston, TX, USA; RT Research Department, Hamad Medical Corporation, Doha, Qatar; RU Department of Biochemistry, American University of Integrative Sciences, Bridgetown, Barbados; RV Department of Neuro Physiotherapy, Charotar University of Science and Technology, Anand, India; RW School of Life Sciences, Xiamen University, Xiamen, China; RX Faculty of Health Science, Universitas Indonesia Maju, Jakarta, Indonesia; RY Department of Medical Informatics, Mashhad University of Medical Sciences, Mashhad, Iran; RZ Applied Biomedical Research Center, Mashhad University of Medical Sciences, Mashhad, Iran; SA Department of Basic Medical Sciences, Islamic Azad University, Mashhad, Iran; SB Department of Internal Medicine, Islamic Azad University, Mashhad, Iran; SC Saveetha Medical College and Hospital, Saveetha Institute of Medical and Technical Sciences, Chennai, India; SD Department of Epidemiology, Stellenbosch University, Cape Town, South Africa; SE Department of Medicine, Northlands Medical Group, Omuthiya, Namibia; SF School of Nursing and Midwifery, Debre Berhan University, Debre Berhan, Ethiopia; SG Department of Pediatrics and Child Health Nursing, Debre Berhan University, Debre Berhan, Ethiopia; SH School of Public Health, Haramaya University, Harar, Ethiopia; SI Research Chair for Evidence-Based Health Care and Knowledge Translation, King Saud University, Riyadh, Saudi Arabia; SJ Faculty of Public Health, Universitas Sam Ratulangi (Sam Ratulangi University), Manado, Indonesia; SK Department of Allied Health and Human Performance, University of South Australia, Adelaide, SA, Australia; SL Public Health Department, Debre Markos University, Debre Markos, Ethiopia; SM K.A. Timiryazev Institute of Plant Physiology, Russian Academy of Sciences, Moscow, Russia; SN Department of Biochemistry, All India Institute of Medical Sciences, Jodhpur, India; SO Second Department of Internal Medicine, Kansai Medical University, Hirakata, Japan; SP Department of Internal Medicine, University of Medicine and Pharmacy at Ho Chi Minh City, Ho Chi Minh City, Viet Nam; SQ Department of Business Analytics, University of Massachusetts Dartmouth, Dartmouth, MA, USA; SR Research and Advocacy Initiative, ALS Vietnam, Quang Ngai, Viet Nam; SS Department of Medicine, University of Crete, Heraklion, Greece; ST Department of Occupational Health and Safety, University of Development, Surabaya, Indonesia; SU International Center for Chemical and Biological Sciences, University of Karachi, Karachi, Pakistan; SV Department of Biology and Biochemistry, University of Houston, Houston, TX, USA; SW Medical Genomics Research Department, King Abdullah International Medical Research Center, Riyadh, Saudi Arabia; SX Kasturba Medical College Mangalore, Manipal Academy of Higher Education, Manipal, India; SY Amity Institute of Biotechnology, Amity University Rajasthan, Jaipur, India; SZ Department of Rehabilitation Sciences, Hong Kong Polytechnic University, Hong Kong, China; TA Department of Infectious Disease, Kermanshah University of Medical Sciences, Kermanshah, Iran; TB Department of Zoology, Central University of Punjab, Bathinda, India; TC Department of Human Genetics & Molecular Biology, Bharathiar University, Coimbatore, India; TD Department of Health Care Administration and Economics, National Research University Higher School of Economics, Moscow, Russia; TE College of Medicine and Health Sciences, Wollo University, Dessie, Ethiopia; TF School of Life Course and Population Sciences, King's College London, London, UK; TG Department of Food Science and Human Nutrition, Michigan State University, East Lansing, MI, USA; TH School of Public Health, Zhejiang University, Zhejiang, China; TI Department of Public Health Science, Fred Hutchinson Cancer Research Center, Seattle, WA, USA; TJ Department of Environmental Health and Epidemiology, National Institute for Research in Environmental Health, Bhopal, India; TK Department of Microbiology and Immunology, Zagazig University, Zagazig, Egypt; TL Department of Cells and Tissues, Molecular Biology Institute of Barcelona, Barcelona, Spain; TM Department of Pediatrics, Kyung Hee University, Seoul, South Korea; TN Department of Biostatistics, University of Toyama, Toyama, Japan; TO Department of Public Health, Juntendo University, Tokyo, Japan; TP Department of Health Sciences, James Madison University, Harrisonburg, VA, USA; TQ Research and Development Department, Sina Medical Biochemistry Technologies, Shiraz, Iran; TR Department of Bioengineering and Therapeutical Sciences, University of California San Francisco, San Francisco, CA, USA; TS Department of Administration, PGxAI, San Francisco, CA, USA; TT Department of Public Health, University of Hail, Hail, Saudi Arabia; TU Department of Animal Resources, Salahaddin University-Erbil, Erbil, Iraq; TV Department of Nutrition and Dietetics, Cihan University-Erbil, Erbil, Iraq; TW Jockey Club School of Public Health and Primary Care, The Chinese University of Hong Kong, Hong Kong, China; TX Department of Anesthesiology & Pain Medicine, University of Washington, Seattle, WA, USA

## Abstract

**Background:**

Child growth failure (CGF), which includes underweight, wasting, and stunting, is among the factors most strongly associated with mortality and morbidity in children younger than 5 years worldwide. Poor height and bodyweight gain arise from a variety of biological and sociodemographic factors and are associated with increased vulnerability to infectious diseases. We used data from the Global Burden of Diseases, Injuries, and Risk Factors Study (GBD) 2023 to estimate CGF prevalence, the risk of infectious diseases associated with CGF, and the disease mortality, morbidity, and overall burden associated with CGF.

**Methods:**

In this analysis we estimated the all-cause and cause-specific (diarrhoea, lower respiratory tract infections, malaria, and measles) disability-adjusted life-years (DALYs) lost and mortality associated with stunting, wasting, underweight, and CGF in aggregate. We combined the burden associated with mild, moderate, and severe forms of CGF: stunting was defined as height-for-age Z scores (HAZ) less than –1, underweight was defined as weight-for-age Z scores (WAZ) less than –1, and wasting was defined as weight-for-height Z scores (WHZ) less than –1, according to WHO Child Growth Standards. Population-level continuous distributions of HAZ, WAZ, and WHZ were estimated for 2000 to 2023 using data from surveys, literature, and individual-level study data. The risk of incidence of, and mortality due to, diarrhoea, lower respiratory infections, malaria, and measles was separately estimated in a meta-regression framework from longitudinal cohort data for Z scores less than –1. Finally, fatal outcomes associated with these diseases were estimated with vital registration, verbal autopsy, and case-fatality data, while non-fatal outcomes were estimated with surveys as well as health-care utilisation and case reporting data. The exposure prevalence and relative risk estimates were from continuous distributions, allowing for direct assessment of the attributable fractions for mild, moderate, and severe stunting, underweight, wasting, and the combined impact of child growth failure within populations. All estimates were age-specific, sex-specific, geography-specific, and year-specific.

**Findings:**

We estimated that, in children younger than 5 years in 2023, CGF was associated with 79·4 million (95% uncertainty interval [UI] 47·0–106) DALYs lost and 880 000 (517 000–1 170 000) deaths. This represented 17·9% (10·6–23·8) of 444 million (434–457) total under-5 DALYs and 18·8% (11·1–25·0) of all 4·67 million (4·59–4·75) under-5 deaths. Compared to stunting (33·0 million [24·1–42·2] DALYs, 373 000 [272 000–477 000] deaths) and wasting (39·2 million [23·8–53·0] DALYs, 428 000 [256 000–583 000] deaths), childhood underweight was associated with the largest share of CGF-related disease burden: 52·2 million (21·9–75·1) DALYs and 573 000 (236 000–824 000) deaths in children younger than 5 years in 2023.

**Interpretation:**

CGF remains a leading factor associated with death and disability in children younger than 5 years, despite global attention and focused interventions to reduce the prevalence of associated CGF indicators. Our findings underscore the need for policies, strategies, and interventions that focus on all indicators of CGF to reduce its associated health burden.

**Funding:**

Gates Foundation.

## Introduction

Child growth failure (CGF), characterised by poor linear height and bodyweight gain, represents a complex interplay of nutritional, maternal, developmental, socioeconomic, environmental, and health-care factors.^1^ Children who experience growth failure have a greater infectious disease burden, including risk of mortality, than those who do not.^2^, ^3^, ^4^, ^5^ CGF is associated with lifelong consequences such as cognitive and metabolic impairment, which might contribute to poorer educational performance and lost opportunities.^1^, ^6^ Therefore, quantifying its prevalence and associated disease burden on a global scale is of crucial importance for devising targeted interventions and policies aimed at alleviating its associated burden and generating advocacy for funding prevention and treatment.

**Research in context**

**Evidence before this study**

Even with decades of substantial global investment, child growth failure (CGF) remains a leading risk factor associated with death and disability in children younger than 5 years. In the most recent global estimates of CGF burden, the Global Burden of Diseases, Injuries, and Risk Factors Study (GBD) 2021 estimated that all CGF (mild, moderate, and severe) was associated with 774 000 deaths and 70·2 million DALYs in children younger than 5 years in 2021. Despite the crucial importance of understanding the causes and relative risks of negative health outcomes associated with CGF, global estimates of the increased relative risks of negative health outcomes associated with CGF typically rely on a synthetic review of 11 studies that were conducted between 1977 and 1997.

**Added value of this study**

Using updated estimates from GBD 2023 of both the relative risks of CGF associated with health outcomes as well as the proportion of children experiencing CGF, we present estimates of the global burden associated with CGF aggregated across mild, moderate, and severe forms for each of three CGF indicators: stunting (low height-for-age), underweight (low weight-for-age), and wasting (low weight-for-height). For each indicator, our estimates represent burden estimates for children that are at least 1 SD below the WHO Child Growth Standards. The length and quality of the studies included in the re-estimation of relative risks allowed for distinct quantification of the morbidity and mortality risk associated with each CGF indicator for lower respiratory infections, diarrhoea, measles, and malaria. In particular, using relative risks based on increased mortality alone, we found that more than 18% of all under-5 deaths were associated with CGF, with almost as many deaths associated with stunting as with wasting.

**Implications of all the available evidence**

Previous estimates of CGF identified wasting as the primary CGF risk factor associated with disease burden. We found that stunting is associated with almost as much of the global CGF burden as wasting and that stunting prevalence has decreased at a far slower rate than wasting over the past decades. Given the previously observed difficulty in a child recovering from stunting after their third month of life, our estimates should serve as a call to refocus efforts to avert CGF at the earliest stages of onset.

Since 1990, substantial strides have been made in reducing childhood mortality and improving overall child growth and health,^7^ despite some setbacks caused by disruptions in health-care services and treatment of acute malnutrition due to the COVID-19 pandemic.^8^ However, many countries are not on track to meet the UN Sustainable Development Goals for reducing child mortality and the prevalence of CGF, and there is substantial overlap in the countries that have high child mortality and CGF.^7^, ^9^ In 2019, more than half of all under-5 deaths globally were attributable to malnutrition, including CGF, low birthweight and short gestation, suboptimal breastfeeding, vitamin A deficiency, and zinc deficiency.^2^ This is in part because growth failure is a leading risk factor associated with primary infectious causes of death among children younger than 5 years.^3^

Numerous interventions exist that can prevent CGF, such as increased rates of exclusive breastfeeding^10^ and improved micronutrient uptake,^11^ but the effectiveness of these interventions varies across CGF indicators^11^ as well as by the age of the child.^12^ As outlined in the most recent WHO guidelines, severe acute malnutrition in children from 0 to 59 months of age can frequently be managed by community health workers and increased monitoring of the child's health.^13^ Although community case management can also help reduce stunting, the likelihood of successfully reversing stunting decreases as the infant grows older,^14^ indicating that identifying children whose growth is faltering as early as possible is critical. Substantial evidence suggests that short gestation, low birthweight, and factors influencing a fetus's development in utero drive many growth outcomes in the early years of life and that the ideal time to intervene on growth failure might be before the child is born^15^ or even conceived.^16^ Finally, although CGF is a risk factor associated with more severe outcomes for various infectious diseases, enteric infections can also alter intestinal absorption rates, increasing the likelihood of CGF and creating a so-called vicious cycle.^17^ Improved integrated case management and immediate access to treatments such as oral rehydration solution can reduce the severity of disease and in turn decrease the likelihood of growth failure.^17^ Proximal and effective interventions with strong evidence of impact are described in appendix 1 (table S7). A concerted effort by the global community to increase detection of CGF and access to treatment has led to substantial declines in CGF over the past few decades.

Many existing estimates of CGF focus primarily on the prevalence of moderate and severe stunting, underweight, and wasting, defined by the proportions of children less than two Z scores from the median age-specific and sex-specific global growth standards for height-for-age (HAZ) for stunting, weight-for-age (WAZ) for underweight, and weight-for-height (WHZ) for wasting.^4^, ^18^ Measuring the prevalence of these indicators is convenient for comparing populations between geographical regions and over time and for defining and monitoring progress towards global goals.^19^ However, focusing exclusively on these prevalence estimates might obscure meaningful changes in population distributions and does not connect CGF with disease burden and mortality.^20^

This analysis extends on previous estimates of the prevalence of CGF by including all forms of CGF (mild, moderate, and severe; panel), quantifying infectious-disease-specific attributable fractions from previously estimated continuous distributions of growth indicators and newly estimated continuous relative risks for incidence and mortality due to diarrhoea, lower respiratory infections, measles, and malaria.^4^, ^18^, ^20^ In this study, based on the Global Burden of Diseases, Injuries, and Risk Factors Study (GBD) 2023, we present comprehensive estimates of disability-adjusted life-years (DALYs) and deaths associated with CGF at the global, regional, and national level, including changes over time. Additionally, we describe updated cause-specific risks of incidence and mortality associated with each CGF indicator and the associated attributable burden. The resulting estimates of the disease burden associated with CGF are available for 204 countries and territories, five age groups younger than 5 years, by sex, from 1990 to 2023. This manuscript was produced as part of the GBD Collaborator Network and in accordance with the GBD Protocol.^21^

Panel **Rationale for modelling all child growth failure (mild, moderate, and severe)**

We modelled all child growth failure (mild, moderate, and severe) at height-for-age Z scores (HAZ), weight-for-age Z scores (WAZ), and weight-for-height Z scores (WHZ) less than –1 to capture a broader distribution of risk than the conventional Z score less than –2 threshold. Evidence suggests that the risks of infectious morbidity and mortality increase even with mild growth deficits (Z score <–1), and continuous risk curves allow more accurate estimation of the attributable burden. This approach aligns with Global Burden of Diseases, Injuries, and Risk Factors Study (GBD) methodology, which emphasises modelling the full distribution of exposure rather than categorical cut-offs.

## Methods

### Overview

We used four main methodological steps to estimate the burden of all CGF (mild, moderate, and severe). To estimate the attributable fractions for each of the three risk factor components of CGF modelled in GBD—stunting, underweight, and wasting—for diarrhoea, lower respiratory infections, malaria, and measles, we produced estimates of exposure and estimates of the risk of disease given varying levels of exposure. Finally, we multiplied those attributable fractions by cause-specific cases, deaths, and DALYs to produce our results for CGF burden. Each step is described briefly here with specific references for further information. The analyses in this study follow the methods published by the GBD 2023 Disease and Injury and Risk Factor Collaborators.^22^ They have been described in full elsewhere^22^ and are summarised below.

### Estimation of the population-level distributions of HAZ, WAZ, and WHZ

A detailed description of the methods used to estimate continuous distributions for each of the CGF indicators is provided in the study by Fitzgerald and colleagues.^20^ We included data from various sources, such as population-representative surveys, administrative data, and published scientific literature. More than 1700 sources were used in this model; citations and metadata for all sources used in the analysis are available for download from the GBD 2023 Sources Tool. A spatiotemporal Gaussian process regression (ST-GPR) model, a methodology used across many models in GBD that leverages evidence across time and space to produce estimates for each indicator, age group, sex, year, and location, was used to make predictions of the mean HAZ, WAZ, and WHZ and the prevalence of moderate (<–2 Z scores) and severe (<–3 Z scores) growth failure by age, sex, year, and location. This model includes linear effects on maternal care and immunisation, Healthcare Access and Quality (HAQ) Index, prevalence of severe anaemia, the Socio-demographic Index (SDI), age-specific and sex-specific unsafe sanitation summary exposure value, and all-age energy (kilocalories per person per day) as covariates.

Separately, we fit an ensemble model of distribution families to describe each CGF Z score distribution from individual-level data. Ten distributions were fit simultaneously (normal, log-normal, log-logistic, exponential, gamma, mirrored gamma, inverse gamma, Gumbel, mirrored Gumbel, and Weibull). We derived weights for each distribution based on minimised error in predicting CGF prevalence of less than –1 Z score, less than –2 Z scores, and less than –3 Z scores. Finally, we synthesised the results from these steps to estimate continuous HAZ, WAZ, and WHZ curves to estimate a weighted probability density function based on the ensemble distributions and ST-GPR mean and SD values.

### Estimation of the relative risk of cause-specific incidence and mortality at varying levels of CGF indicators

We created new estimates of the relative risk of cause-specific incidence and mortality for continuous distributions of CGF exposure for GBD 2021 and used these estimates for GBD 2023. Estimates of CGF burden in previous GBD publications used categorical exposures (–3, –2, and –1 Z scores) matched with relative risks of cause-specific disease burden at each of those levels of exposure.^22^, ^23^ A longer description of the methods is available elsewhere,^22^ and we have summarised the methods below.

For cause-specific mortality, we included 22 870 children and more than 137 000 anthropometric measurements from eight studies, representing 1029 cause-specific deaths (283 due to diarrhoea, 737 due to acute lower respiratory infections, and nine due to malaria). We also included hazard ratios for cause-specific mortality from a pooled analysis by Olofin and colleagues^3^ in our meta-regression. For cause-specific incidence, we included 60 436 children and more than 286 000 anthropometric measurements from 19 studies, representing more than 100 000 unique study-defined infectious disease episodes (95 950 diarrhoea episodes, 12 947 acute lower respiratory infections, 4896 malaria episodes, and 376 measles episodes). These data were from longitudinal studies that reported multiple measurements of height and bodyweight over time. We collapsed these observations into bins of Z scores for each CGF indicator and by age and study while summing the number of child-days of infectious disease episodes based on a standardised one-week recall, and cause-specific deaths within each bin.

We used a Bayesian meta-regression tool and risk factor assessment framework built for GBD and used across risk factors to quantify a continuous risk curve for each CGF exposure category and outcome.^24^ Each risk exposure indicator (HAZ, WAZ, and WHZ) across the Z score range –6 to 1 was paired with each outcome (incidence and mortality for each infectious disease) to create a risk–outcome pair. In other words, we built separate models for the incidence and mortality rates for each infectious disease outcome, and for each metric of growth failure (24 models). We used logit-transformed mortality ratio and log-transformed incidence as the continuous independent variables in our models. Age was included as a fixed effect in the model. Studies were included as random intercepts in the model, and a flexible regularised spline fitting algorithm determined the shape of the curves. Age was chosen as a fixed effect since we wished to estimate the specific impact of age on the outcomes while our random effects were chosen as such due to our interest in estimating both the differences by study as well as the general variation one would expect from study to study. The meta-regression model incorporates both within-study and between-study uncertainty measured by standard errors and simulates Bayesian uncertainty intervals including and excluding between-study variation. More details on the meta-regression tool can be found in the publication associated with the method^24^ as well as the publication from which the relative risk estimates are taken.^23^

Because of the high degree of correlation between stunting, underweight, and wasting, we adjusted our relative risk values by simulating a joint distribution of the three indicators using extracted data from Demographic and Health Surveys. Based on an analysis done by McDonald and colleagues,^5^ we fit an interaction term between the three indicators and calculated adjusted relative risks by minimising the error between the crude relative risks from our meta-regression and the expected relative risk derived from the joint estimate with the interaction term. More details of the specific approach taken are provided in appendix 1 of the report by the GBD 2023 Disease and Injury and Risk Factor Collaborators.^22^

### Calculation of a cause-age-sex-location-specific attributable fraction and multiplication by cause burden

Given that there were two continuous distributions, we produced estimates of the attributable fraction of disease incidence and mortality. The attributable fraction represents the proportion of disease burden that was attributable to a given risk factor (ie, the proportion of disease burden that would not exist in the absence of the risk factor). As CGF does not directly cause infections or deaths, the interpretation of the percentage attributable fraction is the proportion of disease burden that would not exist in the absence of the risk factor due to the linked reduction in associated direct biological drivers that cause increased burden such as immune system dysregulation.

The attributable fraction for continuous population-level exposure estimates and relative risks is:

$$PAF_{i,m,c}=\frac{\int_{0}^{\infty }RR_{i,m,c}\left(x\right)P_{i}\left(x\right)dx-1}{\int_{0}^{\infty }RR_{i,m,c}\left(x\right)P_{i}\left(x\right)dx}$$

where *i* is the CGF indicator (HAZ, WAZ, or WHZ), *m* is the disease outcome (incidence or death), and *c* is the cause (diarrhoea, lower respiratory infections, malaria, or measles). An attributable fraction for CGF overall was estimated with a multiplicative aggregation of the individual indicator attributable fractions:

$$PAF_{m,c}=1-\prod_{i-1}^{n}\left(1-PAF_{i,m,c}\right)$$

Notably, due to a scarcity of data, GBD does not estimate attributable burden for stunting in the early neonatal (0–6 days) and late neonatal (7–27 days) age groups. Attribution is estimated for the following age groups: 1 to less than 6 months, 6 to less than 12 months, 12 to less than 24 months, 24 to less than 60 months.

### Cause-specific incidence and mortality modelling

A full description of the modelling methods for the incidence and mortality of diarrhoea, lower respiratory infections, malaria, and measles has been published previously.^25^, ^26^

In short, mortality due to diarrhoea, lower respiratory infections, and measles was modelled with a Bayesian ensemble hierarchical tool called the Cause of Death Ensemble model (CODEm).^27^ Measles used CODEm for settings with vital registration systems and used a separate model based on incidence and case-fatality ratio for other settings.^27^ Malaria mortality was modelled with spatially defined incidence and case-fatality estimates.^27^, ^28^ Input data included vital registration, verbal autopsy, administrative records, and surveys. Covariates informed the models, and the final set of ensemble models was selected on the basis of out-of-sample predictive validity. Non-fatal incidence of diarrhoea and lower respiratory infections was modelled in a meta-regression tool with data from population-representative surveys, clinical data, administrative data, and scientific literature.^27^ This meta-regression tool includes a compartmental component that enforces consistency between disease incidence and mortality, making it an internally consistent estimate with mortality. Malaria incidence was based on estimates from the Malaria Atlas Project and on administrative, routine surveillance, and other geolocated and community-representative observations of infection prevalence for *Plasmodium falciparum.*^28^ Measles incidence was estimated in a mixed-effects regression model by use of Joint Reporting Form case notifications and 5-year rolling lagged routine measles vaccination rates.^27^

Cause-specific cases and deaths were multiplied by the population attributable fraction (PAF) values to produce our final estimates of the disease burden associated with each CGF indicator. All steps in the estimation process included 500 iterations of each age-sex-geography-year-specific value. 95% uncertainty intervals (UIs) were calculated as the 2·5th and 97·5th percentiles across all draws. This uncertainty was carried through the entire process to maintain the variation in the estimates. Count data are presented to three significant figures, while rates and percentages are presented to one decimal place.

The code and input data are available on the Global Health Data Exchange (GHDx) website. This study complies with the GATHER requirements for burden estimation and reporting.^29^

### Role of the funding source

The funder of this study had no role in study design, data collection, data analysis, data interpretation, or the writing of the report.

## Results

**Figure 1:**
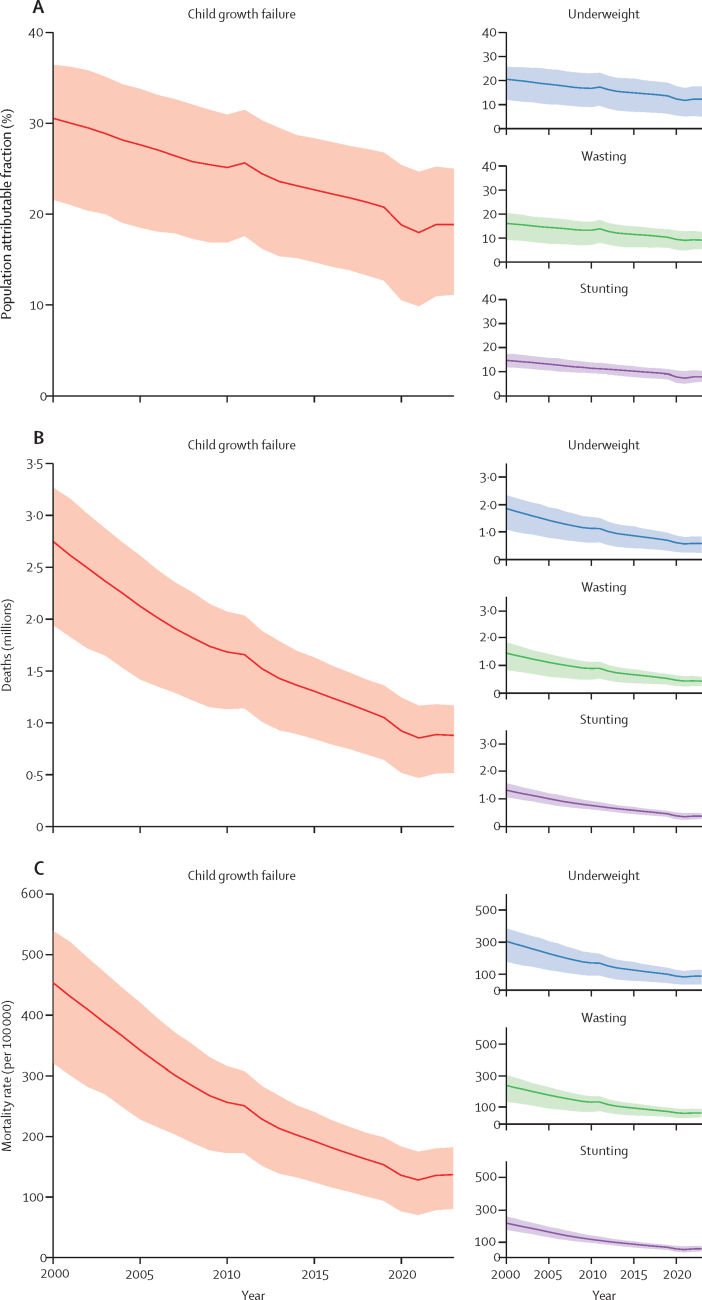
Global burden associated with child growth failure for children younger than 5 years as a fraction of all deaths (A), number of attributable deaths (B), and attributable mortality rate per 100 000 (C) Separate panels show the attributable burden for underweight, wasting, and stunting separately, as well as child growth failure as a whole. Shaded regions represent 95% uncertainty intervals. Estimates combine burden associated with mild, moderate, and severe forms of child growth failure: stunting was defined as height-for-age Z score (HAZ) less than –1; underweight was defined as weight-for-age Z score (WAZ) less than –1; and wasting was defined as weight-for-height Z score (WHZ) less than –1, according to WHO Child Growth Standards.

**Figure 2:**
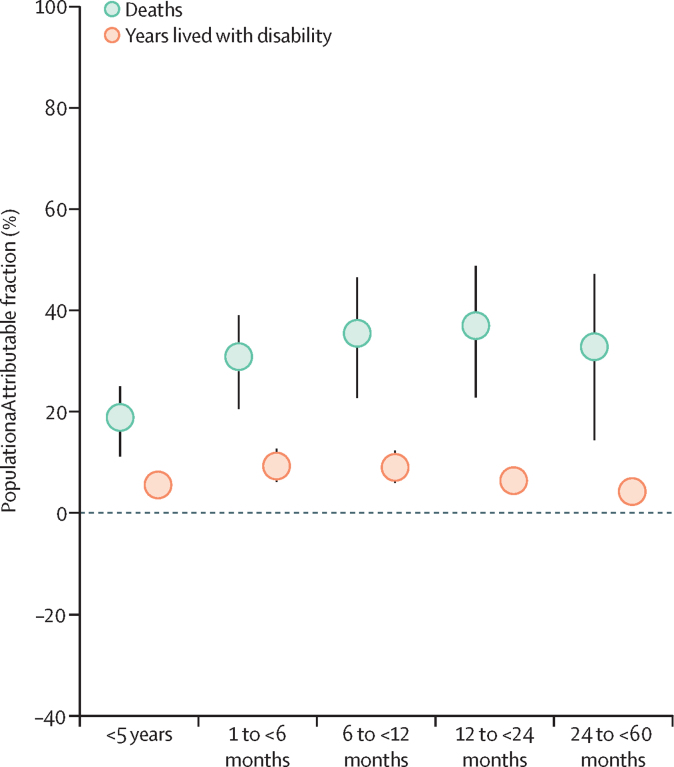
Percentage of all-cause deaths and years lived with disability associated with all child growth failure (mild, moderate, and severe) in children younger than 5 years, globally, in 2023 Population attributable fraction of all-cause deaths and years lived with disability for all children younger than 5 years, as well as age-specific values (1 to <6 months, 6 to <12 months, 12 to <24 months, and 24 to<60 months) are plotted. Dots represent mean population attributable fractions and whiskers represent 95% uncertainty intervals.

Globally, in 2023, in children younger than 5 years, all CGF (mild, moderate, and severe) was associated with 79·4 million (95% UI 47·0–106) DALYs, ranking only behind low birthweight and short gestation (133 million [125–143] DALYs) as the leading risk factors associated with health burden among all children younger than 5 years (table 1; appendix 1 table S1). CGF was associated with 17·9% (10·6–23·8) of 444 million (434–457) total under-5 DALYs (table 2; appendix table S2). In 2023, CGF was associated with 880 000 (517 000–1 170 000) deaths (Figure 1, Figure 2; table 3; appendix 1 table S3), representing 18·8% (11·1–25·0) of all 4·67 million (4·59–4·75) under-5 deaths (figure 1; table 4; appendix 1 table S4) and 1·54 million (0·804–2·41) years lived with disability (YLDs; appendix 1 table S5), representing 5·5% (3·2–7·9) of all 28·0 million (20·4–37·1) under-5 YLDs (figure 2; appendix 1 table S6). Due to limited space and similar trends between DALYs and mortality through time and across countries by indicator and cause, we primarily focus the results presented below on mortality. Full estimates produced in association with these analyses across all health metrics are available via the GBD Results Tool.

**Table 1:** All-cause and cause-specific DALYs associated with child growth failure among children younger than 5 years at the global and GBD super-regional levels, 2023

	**Child growth failure**	**Child underweight**	**Child wasting**	**Child stunting**
**Global**				
All causes	79 400 000 (47 000 000 to 106 000 000)	52 200 000 (21 900 000 to 75 100 000)	39 200 000 (23 800 000 to 53 000 000)	33 000 000 (24 100 000 to 42 200 000)
Diarrhoeal diseases	21 800 000 (13 000 000 to 32 200 000)	10 600 000 (5 870 000 to 17 400 000)	15 100 000 (−1 090 000 to 29 200 000)	7 580 000 (4 290 000 to 11 700 000)
Lower respiratory infections	32 100 000 (22 400 000 to 41 200 000)	22 100 000 (1 690 000 to 38 000 000)	12 500 000 (7 900 000 to 18 100 000)	17 300 000 (12 200 000 to 23 400 000)
Malaria	9 600 000 (−6 360 000 to 30 400 000)	6 980 000 (−4 070 000 to 20 500 000)	..	4 460 000 (−2 350 000 to 19 700 000)
Measles	6 930 000 (2 700 000 to 13 300 000)	3 560 000 (1 330 000 to 6 880 000)	2 590 000 (782 000 to 5 530 000)	3 690 000 (1 320 000 to 7 100 000)
**Central Europe, eastern Europe, and central Asia**				
All causes	792 000 (577 000 to 1 030 000)	442 000 (54 500 to 876 000)	314 000 (246 000 to 393 000)	303 000 (198 000 to 406 000)
Diarrhoeal diseases	70 500 (32 900 to 105 000)	22 000 (12 500 to 34 800)	51 000 (−2120 to 97 000)	17 900 (11 800 to 25 600)
Lower respiratory infections	706 000 (526 000 to 920 000)	405 000 (15 000 to 841 000)	248 000 (158 000 to 342 000)	285 000 (184 000 to 385 000)
Malaria	0 (0 to 0)	0 (0 to 0)	..	0 (0 to 0)
Measles	1040 (501 to 1450)	351 (163 to 510)	374 (140 to 751)	510 (233 to 737)
**High income**				
All causes	57 900 (37 100 to 92 300)	30 100 (9950 to 64 800)	24 800 (18 200 to 32 900)	17 700 (13 900 to 23 500)
Diarrhoeal diseases	11 500 (2420 to 23 200)	2750 (1840 to 4370)	7450 (−436 to 19 100)	2450 (1130 to 3840)
Lower respiratory infections	38 800 (26 600 to 64 000)	19 800 (514 to 54 600)	9830 (5620 to 14 400)	15 200 (10 900 to 21 100)
Malaria	0·0753 (−0·0302 to 0·312)	0·0753 (−0·0302 to 0·312)	..	<0·001 (>–0·001 to <0·001)
Measles	4·48 (1·66 to 6·86)	1·25 (0·478 to 2·01)	1·77 (0·561 to 3·58)	1·73 (0·654 to 2·83)
**Latin America and Caribbean**				
All causes	1 470 000 (1 110 000 to 1 850 000)	920 000 (427 000 to 1 430 000)	624 000 (450 000 to 788 000)	566 000 (417 000 to 728 000)
Diarrhoeal diseases	322 000 (202 000 to 460 000)	118 000 (67 800 to 188 000)	183 000 (−6980 to 415 000)	115 000 (76 600 to 159 000)
Lower respiratory infections	878 000 (641 000 to 1 180 000)	528 000 (22 700 to 1 070 000)	168 000 (97 600 to 242 000)	451 000 (318 000 to 578 000)
Malaria	1810 (−892 to 6850)	1320 (−665 to 4290)	..	684 (−239 to 3520)
Measles	3·5 (1·53 to 5·11)	1·09 (0·494 to 1·64)	0·849 (0·322 to 1·76)	1·95 (0·797 to 2·86)
**North Africa and Middle East**				
All causes	2 800 000 (2 020 000 to 3 590 000)	1 790 000 (884 000 to 2 690 000)	1 490 000 (1 070 000 to 1 920 000)	1 120 000 (816 000 to 1 470 000)
Diarrhoeal diseases	582 000 (284 000 to 1 010 000)	254 000 (128 000 to 458 000)	403 000 (−20 700 to 873 000)	203 000 (107 000 to 345 000)
Lower respiratory infections	1 480 000 (1 010 000 to 2 050 000)	947 000 (61 500 to 1 830 000)	565 000 (328 000 to 848 000)	749 000 (486 000 to 1 060 000)
Malaria	43 800 (−33 200 to 126 000)	34 500 (−23 800 to 98 100)	..	17 600 (−7240 to 72 500)
Measles	282 000 (99 700 to 585 000)	138 000 (50 100 to 288 000)	103 000 (33 100 to 217 000)	154 000 (50 100 to 321 000)
**South Asia**				
All causes	15 400 000 (12 500 000 to 18 100 000)	10 800 000 (5 050 000 to 14 600 000)	9 500 000 (7 040 000 to 11 500 000)	6 210 000 (4 460 000 to 8 120 000)
Diarrhoeal diseases	4 020 000 (2 660 000 to 5 630 000)	2 180 000 (1 400 000 to 3 290 000)	2 930 000 (−263 000 to 5 150 000)	1 390 000 (875 000 to 2 060 000)
Lower respiratory infections	8 790 000 (6 880 000 to 10 700 000)	6 310 000 (526 000 to 9 610 000)	4 430 000 (3 020 000 to 6 100 000)	4 430 000 (2 850 000 to 6 110 000)
Malaria	179 000 (−145 000 to 598 000)	140 000 (−97 700 to 450 000)	..	76 400 (−37 100 to 360 000)
Measles	602 000 (231 000 to 1 270 000)	336 000 (119 000 to 717 000)	285 000 (84 700 to 623 000)	305 000 (111 000 to 653 000)
**Southeast Asia, east Asia, and Oceania**				
All causes	3 730 000 (2 810 000 to 4 660 000)	2 380 000 (1 020 000 to 3 520 000)	1 840 000 (1 320 000 to 2 370 000)	1 520 000 (1 110 000 to 1 990 000)
Diarrhoeal diseases	924 000 (472 000 to 1 530 000)	417 000 (208 000 to 751 000)	611 000 (−33 600 to 1 340 000)	322 000 (168 000 to 552 000)
Lower respiratory infections	2 130 000 (1 500 000 to 2 800 000)	1 430 000 (96 800 to 2 580 000)	737 000 (439 000 to 1 070 000)	1 060 000 (703 000 to 1 480 000)
Malaria	12 700 (−8810 to 44 300)	9450 (−5840 to 30 700)	..	5450 (−2700 to 26 400)
Measles	246 000 (91 200 to 486 000)	118 000 (41 700 to 245 000)	82 000 (24 600 to 172 000)	133 000 (49 500 to 273 000)
**Sub-Saharan Africa**				
All causes	55 200 000 (26 700 000 to 77 100 000)	35 800 000 (13 400 000 to 53 100 000)	25 400 000 (14 400 000 to 36 800 000)	23 300 000 (14 600 000 to 34 000 000)
Diarrhoeal diseases	15 900 000 (9 100 000 to 24 400 000)	7 600 000 (4 100 000 to 12 600 000)	10 900 000 (−762 000 to 21 600 000)	5 530 000 (2 950 000 to 8 790 000)
Lower respiratory infections	18 100 000 (11 100 000 to 25 400 000)	12 400 000 (969 000 to 22 400 000)	6 360 000 (3 670 000 to 10 200 000)	10 300 000 (6 660 000 to 14 400 000)
Malaria	9 360 000 (−6 160 000 to 29 600 000)	6 790 000 (−3 940 000 to 20 000 000)	..	4 360 000 (−2 290 000 to 19 200 000)
Measles	5 800 000 (2 210 000 to 11 100 000)	2 960 000 (1 090 000 to 5 690 000)	2 120 000 (628 000 to 4 460 000)	3 100 000 (1 070 000 to 6 070 000)

Estimates combine the burden associated with mild, moderate, and severe forms of child growth failure: stunting was defined as height-for-age Z score (HAZ) less than −1; underweight was defined as weight-for-age Z score (WAZ) less than −1; and wasting was defined as weight-for-height Z score (WHZ) less than −1, according to WHO Child Growth Standards. The meta-regression model used to estimate relative risks for various levels of risk-factor exposure incorporates both within-study and between-study uncertainty measured by standard errors. The results presented herein incorporate between-study variation, meaning the uncertainty intervals here represent both uncertainty in the relative risks as well as predicted variation due to between-study variation, and as such some uncertainty intervals' lower bounds fall below zero. As described in the publication associated with the method,^24^ a risk factor, outcome, or cause combination would not be included in the table if the uncertainty without between-study variation was not statistically significantly different from zero. Data in parentheses are 95% uncertainty intervals. Count data are presented to three significant figures. GBD=Global Burden of Diseases, Injuries, and Risk Factors Study.

**Table 2:** All-cause and cause-specific population attributable fraction of DALYs among children younger than 5 years for child growth failure at the global and GBD super-regional levels, 2023

	**Child growth failure**	**Child underweight**	**Child wasting**	**Child stunting**
**Global**				
All causes	17·9% (10·6 to 23·8)	11·7% (5·0 to 16·8)	8·8% (5·4 to 11·9)	7·4% (5·6 to 9·5)
Diarrhoeal diseases	75·2% (53·1 to 88·1)	36·6% (24·9 to 47·3)	51·9% (−3·1 to 84·2)	26·2% (18·6 to 31·9)
Lower respiratory infections	59·1% (48·5 to 66·5)	40·5% (2·7 to 63·0)	23·0% (16·0 to 29·3)	31·8% (24·3 to 37·9)
Malaria	26·3% (−17·8 to 69·4)	19·1% (−11·6 to 48·9)	..	12·1% (−6·2 to 44·7)
Measles	66·4% (39·9 to 75·8)	34·0% (18·3 to 42·4)	24·9% (10·8 to 40·9)	35·3% (17·3 to 43·5)
**Central Europe, eastern Europe, and central Asia**				
All causes	11·2% (8·2 to 14·7)	6·2% (0·76 to 12·6)	4·4% (3·5 to 5·5)	4·3% (2·8 to 5·7)
Diarrhoeal diseases	56·4% (27·8 to 78·1)	17·7% (10·5 to 24·7)	40·7% (−1·8 to 74·2)	14·4% (10·0 to 18·3)
Lower respiratory infections	43·5% (32·8 to 57·2)	24·9% (0·93 to 52·3)	15·3% (10·0 to 20·4)	17·5% (11·4 to 23·5)
Malaria	0% (0 to 0)	0% (0 to 0)	..	0% (0 to 0)
Measles	38·9% (18·9 to 48·4)	13·1% (6·2 to 17·5)	14·0% (5·3 to 25·6)	19·0% (8·7 to 25·4)
**High income**				
All causes	0·98% (0·62 to 1·6)	0·51% (0·17 to 1·1)	0·42% (0·30 to 0·57)	0·30% (0·23 to 0·41)
Diarrhoeal diseases	18·4% (3·9 to 35·5)	4·4% (2·8 to 6·9)	11·9% (−0·69 to 29·1)	3·9% (1·7 to 6·1)
Lower respiratory infections	34·6% (24·4 to 54·5)	17·6% (0·44 to 47·2)	8·8% (5·1 to 12·2)	13·6% (9·9 to 17·7)
Malaria	8·3% (−3·4 to 29·8)	8·3% (−3·4 to 29·8)	..	<0·1% (>–0·1 to <0·1)
Measles	13·6% (6·0 to 17·7)	3·8% (1·8 to 5·2)	5·4% (1·9 to 10·0)	5·2% (2·4 to 7·2)
**Latin America and Caribbean**				
All causes	9·2% (6·9 to 11·6)	5·8% (2·7 to 9·1)	3·9% (2·8 to 5·0)	3·5% (2·6 to 4·5)
Diarrhoeal diseases	55·0% (36·4 to 73·7)	20·2% (12·1 to 28·7)	31·3% (−1·1 to 66·5)	19·6% (13·7 to 24·7)
Lower respiratory infections	52·2% (38·7 to 68·7)	31·3% (1·3 to 63·2)	10·0% (6·0 to 13·6)	26·8% (18·7 to 33·5)
Malaria	14·5% (−7·2 to 45·5)	10·8% (−5·3 to 31·2)	..	5·1% (−2·2 to 23·4)
Measles	25·7% (12·3 to 32·4)	8·0% (3·8 to 10·6)	6·2% (2·4 to 11·7)	14·3% (6·5 to 19·7)
**North Africa and Middle East**				
All causes	10·1% (7·3 to 12·8)	6·5% (3·2 to 9·6)	5·4% (3·9 to 6·9)	4·1% (3·0 to 5·3)
Diarrhoeal diseases	64·7% (39·9 to 80·9)	28·2% (19·2 to 37·9)	44·7% (−2·1 to 77·1)	22·5% (16·2 to 27·9)
Lower respiratory infections	54·0% (43·8 to 63·1)	34·5% (2·0 to 58·7)	20·7% (13·9 to 27·0)	27·5% (18·9 to 34·8)
Malaria	31·3% (−23·3 to 78·1)	24·7% (−17·2 to 60·2)	..	12·3% (−6·3 to 49·0)
Measles	61·1% (34·4 to 70·9)	29·8% (15·1 to 38·1)	22·4% (9·3 to 38·7)	33·3% (15·5 to 41·2)
**South Asia**				
All causes	14·1% (11·4 to 16·3)	9·9% (4·6 to 12·8)	8·7% (6·4 to 10·2)	5·7% (4·1 to 7·3)
Diarrhoeal diseases	77·1% (55·2 to 86·8)	41·8% (30·2 to 51·9)	56·2% (−3·9 to 83·6)	26·7% (19·0 to 32·4)
Lower respiratory infections	53·1% (45·1 to 58·8)	38·2% (2·9 to 55·9)	26·8% (19·4 to 33·3)	26·8% (17·3 to 33·8)
Malaria	32·1% (−24·4 to 78·9)	25·2% (−17·7 to 60·4)	..	13·4% (−7·2 to 50·9)
Measles	75·2% (48·7 to 84·8)	42·0% (23·7 to 51·0)	35·8% (16·4 to 55·2)	38·0% (19·0 to 46·4)
**Southeast Asia, east Asia, and Oceania**				
All causes	11·2% (8·4 to 13·7)	7·1% (3·0 to 10·4)	5·5% (4·0 to 7·1)	4·6% (3·4 to 5·9)
Diarrhoeal diseases	68·5% (44·7 to 83·9)	31·0% (21·3 to 40·3)	45·1% (−2·3 to 78·0)	23·8% (17·2 to 29·2)
Lower respiratory infections	56·5% (43·5 to 66·0)	37·8% (2·2 to 62·6)	19·5% (13·0 to 25·3)	28·0% (19·3 to 34·9)
Malaria	26·1% (−18·5 to 69·2)	19·6% (−12·5 to 51·3)	..	10·6% (−5·5 to 42·1)
Measles	60·9% (35·4 to 70·2)	29·3% (15·6 to 36·7)	20·5% (8·5 to 35·1)	32·9% (16·3 to 40·7)
**Sub-Saharan Africa**				
All causes	22·5% (10·9 to 31·7)	14·6% (5·5 to 21·7)	10·4% (5·9 to 15·0)	9·5% (6·0 to 13·9)
Diarrhoeal diseases	76·5% (54·2 to 89·5)	36·6% (24·5 to 47·9)	52·4% (−3·0 to 86·0)	26·6% (18·9 to 32·7)
Lower respiratory infections	64·8% (52·2 to 73·6)	44·4% (2·9 to 69·3)	22·8% (15·1 to 30·0)	36·9% (30·2 to 42·9)
Malaria	26·2% (−17·7 to 69·3)	19·0% (−11·5 to 48·7)	..	12·1% (−6·2 to 44·6)
Measles	66·1% (39·4 to 75·7)	33·8% (18·1 to 42·1)	24·3% (10·4 to 40·2)	35·3% (17·3 to 43·6)

Estimates combine the burden associated with mild, moderate, and severe forms of child growth failure: stunting was defined as height-for-age Z score (HAZ) less than −1; underweight was defined as weight-for-age Z score (WAZ) less than −1; and wasting was defined as weight-for-height Z score (WHZ) less than −1, according to WHO Child Growth Standards. The results presented herein incorporate between-study variation, meaning the uncertainty intervals here represent both uncertainty in the relative risks as well as predicted variation due to between-study variation, and as such some uncertainty intervals' lower bounds fall below zero. As described in the publication associated with the method,^24^ a risk factor, outcome, or cause combination would not be included in the table if the uncertainty without between-study variation was not statistically significantly different from zero. Data in parentheses are 95% uncertainty intervals. Population attributable fractions are presented to one decimal place. DALY=disability-adjusted life-year. GBD=Global Burden of Diseases, Injuries, and Risk Factors Study.

**Table 3:** All-cause and cause-specific deaths associated with child growth failure among children younger than 5 years at the global and GBD super-regional levels, 2023

	**Child growth failure**	**Child underweight**	**Child wasting**	**Child stunting**
**Global**				
All causes	880 000 (517 000 to 1 170 000)	573 000 (236 000 to 824 000)	428 000 (256 000 to 583 000)	373 000 (272 000 to 477 000)
Diarrhoeal diseases	243 000 (146 000 to 360 000)	118 000 (63 600 to 195 000)	169 000 (−11 900 to 327 000)	84 800 (49 900 to 132 000)
Lower respiratory infections	362 000 (253 000 to 465 000)	249 000 (19 200 to 428 000)	141 000 (88 700 to 204 000)	196 000 (138 000 to 264 000)
Malaria	107 000 (−71 300 to 341 000)	77 400 (−44 700 to 229 000)	..	50 800 (−26 800 to 224 000)
Measles	78 600 (30 700 to 150 000)	40 400 (15 200 to 78 100)	29 300 (8850 to 62 600)	42 000 (15 000 to 80 600)
**Central Europe, eastern Europe, and central Asia**				
All causes	8790 (6390 to 11 400)	4860 (512 to 9720)	3420 (2670 to 4320)	3410 (2230 to 4560)
Diarrhoeal diseases	774 (380 to 1140)	239 (123 to 386)	568 (−21·4 to 1080)	195 (117 to 284)
Lower respiratory infections	7920 (5900 to 10 300)	4540 (169 to 9430)	2770 (1770 to 3830)	3200 (2090 to 4320)
Malaria	0 (0 to 0)	0 (0 to 0)	..	0 (0 to 0)
Measles	11·7 (5·63 to 16·4)	3·96 (1·84 to 5·75)	4·18 (1·55 to 8·42)	5·76 (2·63 to 8·34)
**High income**				
All causes	623 (422 to 964)	322 (111 to 697)	269 (201 to 352)	193 (138 to 264)
Diarrhoeal diseases	107 (39·1 to 210)	19·6 (9·8 to 30·3)	78·2 (−1·7 to 202)	20·8 (12·5 to 29·5)
Lower respiratory infections	436 (299 to 718)	222 (5·93 to 612)	110 (63·1 to 162)	172 (124 to 238)
Malaria	<0·001 (>–0·001 to <0·001)	<0·001 (>–0·001 to <0·001)	..	<0·001 (>–0·001 to <0·001)
Measles	0·0505 (0·0185 to 0·0775)	0·0142 (0·00544 to 0·0228)	0·0198 (0·00615 to 0·0404)	0·0197 (0·00742 to 0·0321)
**Latin America and Caribbean**				
All causes	16 500 (12 500 to 20 800)	10 300 (4780 to 16 000)	6980 (5030 to 8820)	6370 (4660 to 8200)
Diarrhoeal diseases	3580 (2290 to 5100)	1310 (730 to 2110)	2060 (−75·7 to 4660)	1280 (824 to 1790)
Lower respiratory infections	9870 (7210 to 13 300)	5930 (256 to 12 000)	1890 (1090 to 2710)	5080 (3600 to 6500)
Malaria	19·2 (−9·6 to 74·3)	13·6 (−6·94 to 46·2)	..	7·78 (−2·72 to 40·0)
Measles	0·0394 (0·0172 to 0·0574)	0·0123 (0·0056 to 0·0186)	0·00933 (0·00331 to 0·0195)	0·022 (0·00896 to 0·0325)
**North Africa and Middle East**				
All causes	30 600 (21 900 to 39 400)	19 300 (9290 to 29 400)	15 900 (11 400 to 20 700)	12 600 (9160 to 16 600)
Diarrhoeal diseases	6410 (3260 to 11 100)	2780 (1340 to 5090)	4500 (−216 to 9740)	2240 (1150 to 3860)
Lower respiratory infections	16 600 (11 400 to 23 000)	10 600 (698 to 20 600)	6330 (3670 to 9520)	8440 (5490 to 11 900)
Malaria	472 (−357 to 1400)	366 (−253 to 1070)	..	200 (−82·7 to 827)
Measles	3200 (1130 to 6630)	1570 (568 to 3260)	1160 (373 to 2460)	1750 (568 to 3640)
**South Asia**				
All causes	165 000 (134 000 to 194 000)	113 000 (49 000 to 155 000)	98 900 (71 200 to 120 000)	69 600 (49 700 to 91 300)
Diarrhoeal diseases	44 200 (30 000 to 61 900)	23 800 (14 900 to 36 600)	32 600 (−2800 to 57 400)	15 400 (9690 to 23 100)
Lower respiratory infections	98 600 (77 200 to 120 000)	70 800 (5950 to 108 000)	49 600 (33 800 to 68 300)	49 900 (32 100 to 68 600)
Malaria	1990 (−1620 to 6700)	1540 (−1090 to 5020)	..	864 (−421 to 4070)
Measles	6810 (2620 to 14 300)	3810 (1350 to 8140)	3220 (956 to 7040)	3450 (1250 to 7410)
**Southeast Asia, east Asia, and Oceania**				
All causes	40 400 (30 300 to 50 500)	25 300 (10 200 to 37 400)	19 400 (13 800 to 25 400)	17 000 (12 400 to 22 400)
Diarrhoeal diseases	10 100 (5410 to 16 800)	4480 (2060 to 8360)	6800 (−339 to 14 900)	3520 (1690 to 6200)
Lower respiratory infections	24 000 (16 900 to 31 500)	16 000 (1100 to 28 900)	8260 (4920 to 12 100)	11 900 (7940 to 16 600)
Malaria	128 (−82·9 to 477)	90·6 (−51·0 to 317)	..	61·9 (−30·7 to 299)
Measles	2790 (1040 to 5520)	1350 (474 to 2790)	926 (277 to 1950)	1510 (562 to 3100)
**Sub-Saharan Africa**				
All causes	618 000 (299 000 to 862 000)	399 000 (149 000 to 594 000)	283 000 (160 000 to 412 000)	264 000 (165 000 to 386 000)
Diarrhoeal diseases	178 000 (104 000 to 274 000)	85 100 (45 200 to 143 000)	123 000 (−8480 to 243 000)	62 200 (33 400 to 99 100)
Lower respiratory infections	205 000 (126 000 to 288 000)	141 000 (11 000 to 253 000)	71 800 (41 300 to 115 000)	117 000 (75 700 to 163 000)
Malaria	105 000 (−69 000 to 333 000)	75 400 (−43 300 to 223 000)	..	49 700 (−26 200 to 219 000)
Measles	65 800 (25 100 to 127 000)	33 700 (12 400 to 64 700)	24 000 (7110 to 50 600)	35 200 (12 200 to 69 000)

Estimates combine burden associated with mild, moderate, and severe forms of child growth failure: stunting was defined as height-for-age Z score (HAZ) less than −1; underweight was defined as weight-for-age Z score (WAZ) less than −1; and wasting was defined as weight-for-height Z score (WHZ) less than −1, according to WHO Child Growth Standards. The results presented herein incorporate between-study variation, meaning the uncertainty intervals here represent both uncertainty in the relative risks as well as predicted variation due to between-study variation, and as such some uncertainty intervals' lower bounds fall below zero. As described in the publication associated with the method,^24^ a risk factor, outcome, or cause combination would not be included in the table if the uncertainty without between-study variation was not statistically significantly different from zero. Data in parentheses are 95% uncertainty intervals. Count data are presented to three significant figures. GBD=Global Burden of Diseases, Injuries, and Risk Factors Study.

**Table 4:** All-cause and cause-specific population attributable fraction of deaths among children younger than 5 years for child growth failure at the global and GBD super-regional levels, 2023

	**Child growth failure**	**Child underweight**	**Child wasting**	**Child stunting**
**Global**				
All causes	18·8% (11·1 to 25·0)	12·3% (5·1 to 17·6)	9·2% (5·5 to 12·5)	8·0% (6·0 to 10·3)
Diarrhoeal diseases	76·7% (54·9 to 89·1)	37·2% (24·7 to 48·4)	53·2% (−3·1 to 86·1)	26·8% (19·2 to 33·0)
Lower respiratory infections	59·4% (48·9 to 66·8)	40·8% (2·7 to 63·3)	23·1% (16·0 to 29·4)	32·1% (24·7 to 38·1)
Malaria	26·4% (−17·8 to 69·5)	19·0% (−11·3 to 48·2)	..	12·4% (−6·4 to 45·9)
Measles	66·6% (40·0 to 76·0)	34·2% (18·4 to 42·5)	24·9% (10·8 to 41·0)	35·5% (17·4 to 43·7)
**Central Europe, eastern Europe, and central Asia**				
All causes	12·4% (9·0 to 16·2)	6·9% (0·72 to 13·8)	4·8% (3·8 to 6·0)	4·8% (3·2 to 6·4)
Diarrhoeal diseases	61·2% (31·8 to 83·2)	18·9% (10·3 to 27·1)	44·8% (−1·8 to 81·1)	15·5% (9·9 to 20·1)
Lower respiratory infections	43·6% (33·0 to 57·4)	25·0% (0·94 to 52·4)	15·3% (10·0 to 20·5)	17·6% (11·5 to 23·7)
Malaria	0·0% (0 to 0)	0·0% (0 to 0)	..	0·0% (0 to 0)
Measles	40·2% (19·5 to 49·8)	13·6% (6·4 to 18·0)	14·4% (5·5 to 26·4)	19·7% (9·0 to 26·4)
**High income**				
All causes	1·2% (0·82 to 1·9)	0·6% (0·2 to 1·4)	0·5% (0·4 to 0·7)	0·4% (0·3 to 0·5)
Diarrhoeal diseases	32·7% (12·1 to 60·1)	6·0% (3·0 to 9·0)	23·9% (−0·52 to 58·1)	6·4% (3·8 to 8·4)
Lower respiratory infections	34·8% (24·6 to 54·8)	17·7% (0·5 to 47·4)	8·8% (5·2 to 12·3)	13·7% (10·1 to 17·9)
Malaria	4·2% (−1·8 to 13·4)	3·8% (−1·5 to 11·7)	..	0·5% (−0·2 to 2·0)
Measles	14·2% (6·2 to 18·4)	4·0% (1·8 to 5·4)	5·5% (2·0 to 10·3)	5·5% (2·4 to 7·6)
**Latin America and Caribbean**				
All causes	10·3% (7·8 to 12·9)	6·4% (3·0 to 10·0)	4·4% (3·1 to 5·5)	4·0% (2·9 to 5·1)
Diarrhoeal diseases	56·8% (38·6 to 75·5)	20·7% (11·9 to 29·7)	32·6% (−1·1 to 69·0)	20·3% (13·7 to 26·0)
Lower respiratory infections	52·4% (38·9 to 68·9)	31·5% (1·3 to 63·4)	10·0% (6·0 to 13·6)	27·0% (19·0 to 33·7)
Malaria	14·4% (−7·1 to 45·4)	10·3% (−5·0 to 29·4)	..	5·6% (−2·4 to 24·8)
Measles	27·3% (12·9 to 34·5)	8·5% (4·0 to 11·2)	6·5% (2·5 to 12·3)	15·3% (6·9 to 21·6)
**North Africa and Middle East**				
All causes	10·9% (7·9 to 13·8)	6·9% (3·3 to 10·3)	5·7% (4·0 to 7·4)	4·5% (3·3 to 5·9)
Diarrhoeal diseases	68·4% (43·9 to 83·7)	29·6% (19·1 to 40·0)	47·8% (−2·1 to 81·2)	23·9% (16·4 to 29·7)
Lower respiratory infections	54·3% (44·1 to 63·4)	34·7% (2·0 to 58·9)	20·8% (13·9 to 27·1)	27·7% (19·2 to 35·0)
Malaria	31·3% (−22·7 to 78·3)	24·3% (−16·2 to 58·1)	..	13·1% (−6·7 to 50·9)
Measles	61·4% (34·5 to 71·2)	30·0% (15·2 to 38·3)	22·4% (9·3 to 38·9)	33·5% (15·6 to 41·5)
**South Asia**				
All causes	14·6% (11·8 to 16·8)	10·0% (4·3 to 13·2)	8·7% (6·3 to 10·4)	6·1% (4·4 to 7·9)
Diarrhoeal diseases	79·4% (58·3 to 88·3)	42·8% (29·6 to 54·0)	58·6% (−3·9 to 86·6)	27·7% (19·6 to 34·2)
Lower respiratory infections	53·4% (45·4 to 59·0)	38·4% (2·9 to 56·2)	26·9% (19·5 to 33·4)	27·0% (17·4 to 34·0)
Malaria	32·1% (−24·3 to 78·9)	25·0% (−17·4 to 60·1)	..	13·8% (−7·3 to 51·8)
Measles	75·9% (49·1 to 85·7)	42·5% (23·9 to 51·6)	36·0% (16·5 to 55·4)	38·4% (19·2 to 46·8)
**Southeast Asia, east Asia, and Oceania**				
All causes	12·3% (9·1 to 15·2)	7·7% (3·1 to 11·2)	5·9% (4·1 to 7·7)	5·2% (3·8 to 6·8)
Diarrhoeal diseases	73·0% (50·6 to 86·9)	32·5% (20·3 to 43·5)	49·1% (−2·2 to 84·1)	25·5% (17·3 to 31·7)
Lower respiratory infections	56·8% (43·9 to 66·3)	38·0% (2·2 to 62·9)	19·6% (13·0 to 25·3)	28·2% (19·6 to 35·1)
Malaria	25·7% (−17·2 to 68·9)	18·1% (−10·6 to 47·0)	..	12·3% (−6·1 to 46·3)
Measles	61·5% (35·8 to 70·9)	29·7% (15·8 to 37·2)	20·6% (8·6 to 35·3)	33·3% (16·4 to 41·2)
**Sub-Saharan Africa**				
All causes	23·4% (11·3 to 32·8)	15·1% (5·6 to 22·3)	10·7% (6·1 to 15·6)	10·0% (6·3 to 14·6)
Diarrhoeal diseases	77·3% (55·2 to 90·2)	37·0% (24·5 to 48·6)	53·1% (−3·0 to 87·0)	27·0% (19·4 to 33·3)
Lower respiratory infections	65·2% (52·5 to 73·9)	44·7% (2·9 to 69·7)	22·9% (15·1 to 30·1)	37·2% (30·6 to 43·2)
Malaria	26·3% (−17·7 to 69·4)	18·9% (−11·2 to 48·0)	..	12·4% (−6·3 to 45·8)
Measles	66·2% (39·5 to 75·8)	33·9% (18·1 to 42·2)	24·3% (10·4 to 40·2)	35·4% (17·4 to 43·7)

Estimates combine burden associated with mild, moderate, and severe forms of child growth failure: stunting was defined as height-for-age Z score (HAZ) less than −1; underweight was defined as weight-for-age Z score (WAZ) less than −1; and wasting was defined as weight-for-height Z score (WHZ) less than −1, according to WHO Child Growth Standards. The results presented herein incorporate between-study variation, meaning the uncertainty intervals here represent both uncertainty in the relative risks as well as predicted variation due to between-study variation, and as such some uncertainty intervals' lower bounds fall below zero. As described in the publication associated with the method,^24^ a risk factor, outcome, or cause combination would not be included in the table if the uncertainty without between-study variation was not statistically significantly different from zero. Data in parentheses are 95% uncertainty intervals. Population attributable fractions are presented to one decimal place. GBD=Global Burden of Diseases, Injuries, and Risk Factors Study.

The number of deaths associated with CGF was 2·75 million (95% UI 1·95–3·27) in 2000, decreasing by an average of 5·0% (4·2–5·9) per year to 880 000 (517 000–1 170 000) in 2023 (figure 1; GBD Results Tool). Some of these decreases were due to secular trends in disease burden not related to CGF. The attributable fraction of CGF decreased at a slower rate, by 2·1% (1·4–3·0) per year since 2000, from 30·5% (21·5–36·3) of deaths in children younger than 5 years in 2000 to 18·8% (11·1–25·0) in 2023 (figure 1; table 4). However, the attributable fraction of CGF among all causes of death for which it contributes as a risk factor decreased less rapidly between 2000, from 73·7% (53·9–86·3) in 2000 to 61·2% (33·6–79·2) in 2023, representing a 0·9% (0·3–2·0) average yearly reduction (GBD Results Tool).^22^

Among the different indicators of CGF, underweight was associated with the greatest disease burden: 12·3% (95% UI 5·1–17·6) of all deaths in children younger than 5 years in 2023, followed by child wasting (9·2% [5·5–12·5]) and child stunting (8·0% [6·0–10·3]; figure 1, table 4; appendix 1 table S4). The fastest decline in attributable burden from 2000 was in child stunting (2·6% [1·8–3·6] decline per year), followed by child wasting (2·4% [1·5–3·2] decline per year) and underweight (2·3% [1·5–3·2] decline per year; figure 1).

**Figure 3:**
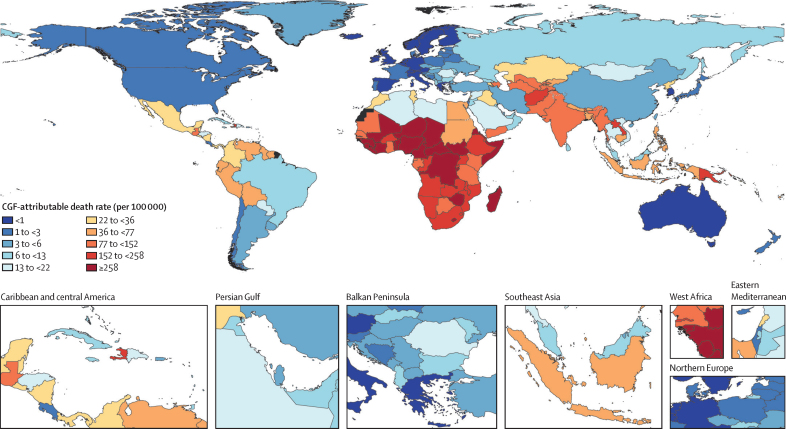
Deaths from all child growth failure (mild, moderate, and severe) in children younger than 5 years per 100 000 in 2023 CGF=child growth failure.

In 2023, at the GBD super-regional level, the highest percentage of total under-5 deaths associated with CGF occurred in sub-Saharan Africa (23·4% [95% UI 11·3 to 32·8]) and in south Asia (14·6% [11·8 to 16·8]; table 4; appendix 1 table S4). In 2023, CGF was associated with 618 000 (299 000 to 862 000) deaths in sub-Saharan Africa and 165 000 (134 000 to 194 000) deaths in south Asia (table 3, appendix 1 table S3). At the national level, in 2023, the largest numbers of deaths among children younger than 5 years associated with CGF occurred in Nigeria (188 000 [70 200 to 270 000]), India (112 000 [88 300 to 131 000]), and DR Congo (50 800 [17 000 to 87 800]; appendix 1 table S3). The highest proportions of under-5 deaths associated with CGF in 2023 were in Chad (40·2% [27·9 to 48·8] of all under-5 deaths), Niger (34·9% [17·3 to 45·5]), and South Sudan (32·8% [18·9 to 43·3]; appendix 1 table S4). Globally, in 2023, CGF was associated with 362 000 (253 000 to 465 000) deaths due to lower respiratory infections in children younger than 5 years (59·4% [48·9 to 66·8] of 610 000 [486 000 to 749 000] deaths due to lower respiratory infection in children <5 years), followed by diarrhoeal diseases (243 000 [146 000 to 360 000]; 76·7% [54·9 to 89·1] of 317 000 [213 000 to 462 000] under-5 diarrhoea deaths), malaria (107 000 [–71 300 to 341 000]; 26·4% [–17·8 to 69·5] of 409 000 [158 000 to 735 000] under-5 malaria deaths), and measles (78 600 [30 700 to 150 000] deaths; 66·6% [40·0 to 76·0] of 118 000 [47 700 to 211 000] under-5 measles deaths; tables 3, 4; appendix 1 figures S1–S5; tables S3, S4). CGF was also associated with the burden of cause-specific infectious disease incidence, including 27·7% (–24·4 to 70·7) of 605 000 (387 000 to 884 000) under-5 malaria YLDs, 24·7% (–18·1 to 56·9) of 767 000 (527 000 to 1 070 000) under-5 diarrhoeal YLDs, 13·4% (–32·7 to 50·4) of 60 000 (40 100 to 85 900) under-5 lower respiratory infection YLDs, and 11·7% (–4·9 to 30·3) of 38 400 (15 000 to 80 000) under-5 measles YLDs (appendix 1 figures S1–S4, table S6). The CGF-attributable burden by cause varied substantially between countries and regions (figure 3; appendix 1 figure S5). The attributable fraction by cause was lowest among countries in the high-income super-region, but the range of deaths due to diarrhoea and lower respiratory infection associated with CGF still varied from 6·0% to 51·2% between countries in this super-region (appendix 1 table S2).

## Discussion

In 2023, CGF was a leading factor associated with mortality in children younger than 5 years globally, falling only behind short gestation and low birthweight, and was associated with approximately 791 000 deaths due to diarrhoea, lower respiratory infections, malaria, and measles. The burden associated with CGF decreased between 2000 and 2023 and has been an important correlate for tracking reductions in childhood mortality.^30^, ^31^ Multifaceted strategies are needed to address the intersectional contribution of environmental, socioeconomic, biological, and behavioural factors that affect childhood growth.

No single intervention is likely to improve childhood growth for all children.^1^ Recent reviews of effective interventions for maternal and childhood malnutrition describe interventions (appendix 1 table S7) according to direct and indirect relationships between the health sector or other macro-level sectors and undernutrition and provide a useful framework to consider different interventions at different points in the life cycle.^32^, ^33^ Although societal and indirect interventions, such as family planning and reproductive health services, or poverty alleviation and women's empowerment strategies, might have important effects on CGF, several reviews have focused on the more proximal interventions that affect childhood environment and biology.^32^, ^34^

Across the CGF indicators, childhood underweight was associated with the largest proportion of the under-5 disease burden. Due to the calculations for underweight, wasting, and stunting, WAZ scores are depressed by both wasting and stunting, making WAZ lowest when both substantial wasting and stunting are present. Moreover, there is evidence that wasting and stunting reinforce each other, meaning that stunting increases the risk of future wasting and vice versa—a relationship that gets stronger as a child grows older.^12^ As such, locations with a high estimated burden associated with all three CGF indicators are likely to require disproportionately more intense interventions than those where stunting and wasting are reinforcing each other.

Recovery from stunting is rare: most children who experience stunting do not recover towards the global mean for length or height, and those who do gain a small amount on their peers.^35^ Although there is some evidence that stunting can be addressed after the first 1000 days of life,^36^ early detection and intervention are critical for successful reversal of stunting.^4^ Our work found that a larger proportion of the CGF burden was associated with stunting than in previous estimates; given the difficulty in reversing stunting, our estimates should be used to identify locations in need of increased early detection and intervention. In a pooled analysis by Mertens and colleagues,^12^ comprising 19 studies and about 60 000 infants, the cumulative incidence, including at birth, of stunting was 25% in the first 3 months of life.^12^ This study also found that the incidence of wasting peaked in the first 3 months of life.^12^ Recovery from wasting is more common than from stunting. In the pooled analysis,^12^ about 65% of infants recovered from wasting within 60 days in the first 3 months of life, and about 50% of infants who experienced wasting at older ages recovered in 60 days. This suggests that changes in lifestyle or interventions have a larger impact in younger compared to older children and for wasting compared to stunting.

Early detection must begin before birth. On average, children in low-income and middle-income settings tend to grow more slowly than the WHO global reference standard, moving further away from the global mean as they age up to 5 years. In part, this appears to be because they are born small, and children born with low birthweight or of short gestation are more likely to experience growth failure.^14^ Other reviews support the importance of intervening in the pre-conception, pregnancy, or neonatal periods to prevent the burden of CGF.^14^, ^15^, ^32^

There are several potential mechanisms that explain how suboptimal growth is associated with an increased risk of infectious disease burden, including an altered microbiome, chronic inflammation, immune system dysregulation, changes in endothelial barrier function, and others.^37^ A review of the literature concluded that although improvements in nutrition can reverse susceptibility to infection, this effect is not well characterised.^1^ Reversing acute malnutrition might help make children more resilient to infection.

Our study includes meaningful updates to previously reported estimates of the CGF burden in GBD 2019.^2^ We used continuous estimates of height and bodyweight by age, sex, geography, and year instead of modelled estimates of the prevalence of CGF indicators. This approach reflects the continuous nature of deviations in growth from an expected mean. We also used continuous estimates of the relative risks of incidence and mortality due to infectious causes for each CGF indicator by age group. Together, these improvements have produced estimates of a higher specificity and with a better reflection of the true, underlying relationship between population-level exposures and risk factors.

Since GBD 2021, updated relative risk estimates have been used to quantify the burden of disease that is associated with CGF.^22^ Previous iterations of GBD (eg, GBD 2017 and GBD 2019)^2^ used the cause-specific mortality hazard ratios from the study by Olofin and colleagues^3^ for both incidence and mortality. In addition to estimating the risk of cause-specific disease incidence, there are two main ways in which these estimates differ from those values. First, the relative risk of lower respiratory infection due to low HAZ and low WAZ are meaningfully higher than in the study by Olofin and colleagues^3^ and in comparison with a separate systematic review and network meta-analysis.^22^, ^38^ Second, we identified statistically significant relationships between low HAZ and low WAZ and malaria mortality, which were not observed in the smaller sample size and discrete categorical analysis by Olofin and colleagues.^3^ These CGF indicators were only included as risk factors associated with malaria mortality in GBD 2021 based on these findings and might therefore represent a gap in previous estimates of the malaria burden.

Although our updated CGF burden estimates highlight changes in the proportions of the under-5 health burden associated with wasting, stunting, and underweight compared to previous iterations of GBD, the total CGF burden remains comparable to that of GBD 2021, GBD 2019, and GBD 2017. However, even with increased relative risks and estimates of the stunting burden and after adjusting for reductions in the CGF burden in the past decade, our stunting estimates remain considerably lower than those of the Maternal and Child Nutrition Study Group (MCNS).^4^ The MCNS group used CGF indicator prevalence estimated by the UN^39^ and the Nutrition Impact Model Study^40^ as well as cause-specific relative risks from a pooled analysis of ten longitudinal studies^3^ to estimate CGF-attributable deaths in children younger than 5 years in 2011 (appendix 1 table S8). Our estimates are based on aggregating the burden associated with mild, moderate, and severe forms of each CGF indicator (eg, Z scores <–1) while the MCNS group focused only on moderate and severe forms (eg, Z scores <–2). In spite of this, our underweight and wasting burden estimates are somewhat comparable with those of the MCNS group. A comprehensive comparison between estimates would require comparing the burden using identical definitions of stunting, wasting, and underweight, which is difficult considering that it depends on exposure prevalence, risk of cause-specific death, and the total number of under-5 and cause-specific deaths. Importantly, no other group, to date, has used the updated relative risk estimates for CGF indicators used in the current study, which probably explains some of the differences in estimates.

There are several limitations to this analysis. First, our estimates depend on numerous sources of data for childhood growth, infectious disease incidence, and infectious disease mortality, each with various potential gaps and biases, which we attempted to resolve using expert opinion and sophisticated statistical models. One common data gap for each of the inputs into this study is that the burden of CGF is highest in countries in the lowest SDI quintiles that do not have robust vital registration and disease surveillance systems. We attempted to account for uncertainty throughout our modelling process by including measured error when possible and producing estimates incorporating uncertainty in each step. We strived to report our findings both as the mean from the posterior distributions as well as the 2·5th and 97·5th percentiles of those distributions. Capturing and reporting uncertainty is crucial in modelling burden of disease, including this report. Second, many unanswered questions remain about the impact of the COVID-19 pandemic and the associated disruptions in maternal and child health services on child nutrition and mortality.^41^ We have included modelled impacts on cause-specific child mortality,^42^ but strong evidence of changes in vaccine coverage or birth size and gestational age, two confounding risks for infectious disease mortality in children, could change our understanding of the current and forecasted burden of CGF. Third, although we believe that our analysis strengthens previous burden estimates by using estimated continuous distributions for CGF prevalence and for the risk of infections associated with CGF, our approach is cross-sectional in time and might not reflect the longitudinal nature of childhood growth. For example, a child who has been experiencing growth failure for several months might have a different risk of infection or death than a child with a newly incident case of growth failure—a nuance that is not captured in our analysis. Such an analysis would need to account for repeated measurements of growth and potentially repeated measures of infection events.^43^ It is likely that a cross-sectional measurement of growth does not accurately represent an individual child's risk of disease, especially given the importance of birth and early-life exposures.^35^ Fourth, as mentioned earlier, low birthweight and short gestation have been found to be correlated with CGF outcomes at birth, during the critical first 1000 days of life, and throughout childhood. Better data and a deeper analysis are needed to carefully link health status at and before birth to health outcomes throughout childhood to parse the relative risks of wasting, stunting, and underweight from low birthweight and short gestation. Finally, we used an adjustment after modelling our relative risk curves to account for the correlation between different growth failure indicators. For example, children who have a low height-for-age are more likely to have low weight-for-age.^12^ Future work to simultaneously quantify such correlations in a single statistical model might have an important impact on risk estimates.

The global burden of disease associated with all CGF (mild, moderate, and severe) is substantial and concentrated in south Asia and sub-Saharan Africa. Children experiencing growth failure are at increased risk of mortality and incidence of infectious diseases. Although the burden associated with CGF has decreased in the past 20 years, more must be done to prevent children from being born small or preterm and to prevent children's growth from faltering. All children deserve an opportunity to have a healthy and productive life, but too many are being denied that chance because of poor growth. By accurately parsing the relative associations of the three main CGF indicators with childhood mortality and morbidity, our estimates provide an unprecedented opportunity to target interventions specifically for the dominant indicator, outcome, and cause of burden in each location.

### GBD 2023 Child Growth Failure Collaborators

### Affiliations

### Contributors

### Data sharing

To download the citations and metadata for the input data used in these analyses, please visit the Global Health Data Exchange GBD 2023 Sources Tool: https://ghdx.healthdata.org/gbd-2023/sources. To download estimates produced in these analyses, please visit the GBD Results Tool: https://vizhub.healthdata.org/gbd-results/.

## Declaration of interests

S Afzal reports support for the present manuscript from the Institute of Public Health Lahore; grants or contracts from the Dean office Institute of Public Health Lahore; honoraria for experts, lectures, visiting speakers, and educational seminars provided by the Dean Institute of Public Health Lahore; support for attending meetings and travel provided by the Dean Institute of Public Health, Lahore Pakistan; leadership or fiduciary roles in board, society, committee, or advocacy groups, paid or unpaid as a Member of the Pakistan Higher Education Commission Research Committee, Member of Pakistan Medical and Dental Commission Research and Journals Committee, Member of Pakistan National Bioethics Committee, Member of Pakistan Society of Internal Medicine, Member of Pakistan Association of Medical Editors, Member of Medical Microbiology and Infectious Diseases Society, a Fellow of LEADS International, Fellow of Faculty of Public Health UK, and as a Fellow of College of Physicians and Surgeons Pakistan; receipt of equipment, materials, drugs, and services including computer software and equipment from Bergen University Norway for research writing; and other financial or non-financial support from the Dean Public Health Institute of Public Health Birdwood Lahore, outside the submitted work. J Baker reports grants or contracts paid to her institution from Novo Nordisk Foundation, World Cancer Research Fund, Independent Research Council Denmark, and European Union Horizon; consulting fees from Novo Nordisk; payment or honoraria for lectures, presentations, speakers bureaus, manuscript writing, or educational events from Novo Nordisk; support for attending meetings or travel, or both, from the European Association for the Study of Obesity; participation on a data safety monitoring board or advisory board from Novo Nordisk; and leadership or fiduciary roles in other board, society, committee, or advocacy groups, paid or unpaid with the European Association for the Study of Obesity, outside the submitted work. M L Bell reports grants or contracts paid to her institution from the US Environmental Protection Agency, National Institutes of Health, Hutchinson Postdoctoral Fellowship, Health Effects Institute, Robert Wood Johnson Foundation, Wellcome Trust Foundation, and Internal Yale funding for the Yale Institute for Biospheric Studies; consulting fees from Clinique and Toximap; honoraria for speaking from the Colorado School of Public Health, Duke University, University of Texas, Data4Justice, Korea University, UPenn, Brown University, and Northeastern University; honorarium for editorial duties from IOP Publishing; honorarium for grant review from the NIH, Health Canada, Environment, Health & Safety, Program Advisory Committee, UK Research and Innovation, AXA Research Fund Fellowship, and University of Texas; honorarium for research from Korea University; honoraria for participation on an external advisory committee from Harvard University and University of Montana; honorarium for online survey or workshop from SciQuest; support for attending meetings or travel, or both, from the Colorado School of Public Health, University of Texas, Duke University, Harvard University, American Journal of Public Health, Columbia University, Harvard, Community Modeling and Analysis System conference, Nature Conference, Boston University, and Northeastern University; leadership or fiduciary roles in other board, society, committee, or advocacy groups, paid or unpaid from the Fifth National Climate Assessment, *Lancet* Countdown, US EPA Clean Air Scientific Advisory Committee (CASAC), Johns Hopkins Department of Environmental Health And Engineering Advisory Board, Harvard external advisory committee for training grant, WHO Global Air Pollution and Health Technical Advisory group, and the National Academies Panels and Committees, outside the submitted work. S Bhaskar reports grants or contracts from the Japan Society for the Promotion of Science (JSPS), Japanese Ministry of Education, Culture, Sports, Science and Technology (MEXT), Grant-in-Aid for Scientific Research (KAKENHI; Grant ID 23KF0126), JSPS and the Australian Academy of Science, JSPS International Fellowship (Grant ID P23712); leadership or fiduciary roles in other board, society, committee, or advocacy groups, paid or unpaid as District Chair, Diversity, Equity, Inclusion & Belonging of Rotary District 9675 (Sydney, Australia); as Chair, Founding Member, and Manager of the Global Health & Migration Hub Community, Global Health Hub Germany (Berlin, Germany); as Editorial Board Member of *PLOS One*, *BMC Neurology*, *Frontiers in Neurology*, *Frontiers in Stroke*, *Frontiers in Public Health*, *Journal of Aging Research*, *Neurology International*, *Diagnostics*, and *BMC Medical Research Methodology*; as a member of the College of Reviewers, Canadian Institutes of Health Research (CIHR), Government of Canada; as the Director of Research of World Headache Society (Bengaluru, India), as Expert Adviser/Reviewer of Cariplo Foundation (Milan, Italy), as Visiting Director of National Cerebral and Cardiovascular Center, Department of Neurology, Division of Cerebrovascular Medicine and Neurology, Suita (Osaka, Japan); as Member, Scientific Review Committee of Cardiff University Biobank (Cardiff, UK), as Chair of Rotary Reconciliation Action Plan, and Healthcare and Medical Adviser at Japan Connect (Osaka, Japan), outside the submitted work. A A Fomenkov reports support for the present manuscript from the Ministry of Science and Higher Education of the Russian Federation (theme number 122042600086-7 and number 122042700043-9). N J Kassebaum reports support for the present manuscript from the Gates Foundation (funding for Global Burden of Disease Study); consulting fees from Fujifilm Sonosite (product development consultation unrelated to present work) and Philips (product development consultation unrelated to present work); payment or honoraria for lectures, presentations, speakers bureaus, manuscript writing, or educational events from Bristol Myers Squibb (travel expenses plus consulting fees for presenting GBD results at international meeting). K Krishan reports other financial or non-financial interests from the UGC Centre of Advanced Study, CAS II, awarded to the Department of Anthropology, Panjab University (Chandigarh, India), outside the submitted work. J Liu reports support for the present manuscript and grants from the National Natural Science Foundation (72474005) and Beijing Natural Science Foundation (L222027). L Monasta reports support for the present manuscript from the Italian Ministry of Health (Ricerca Corrente 34/2017), payments made to the Institute for Maternal and Child Health IRCCS Burlo Garofolo. S Nomura reports support for the present manuscript from the Ministry of Education, Culture, Sports, Science and Technology of Japan (24H00663) Grant and the Precursory Research for Embryonic Science and Technology from the Japan Science and Technology Agency (JPMJPR22R8) Grant. Y L Samodra reports grants or contracts from Taipei Medical University (Taiwan), Type A Doctoral Scholarship, Institute of Epidemiology and Preventive Medicine, National Taiwan University, National Science and Technology Council Taiwan, Post-doctoral Fellow contract; payment or honoraria for lectures, presentations, speakers bureaus, manuscript writing, or educational events from Generasi Peneliti, Indonesia; leadership or fiduciary roles in board, society, committee, or advocacy groups, paid or unpaid as the co-founder of Benang Merah Research Center, Indonesia (https://www.benangmerah.net); and other financial or non-financial interests as a scholarship mentor with Jago Beasiswa (https://www.idebeasiswa.com), outside the submitted work. V Sharma acknowledges support from Directorate of Forensic Science, Ministry of Home Affairs research project (DFSS28(1)2019/EMR/6) at the Institute of Forensic Science & Criminology, Panjab University (Chandigarh, India), outside the submitted work. L M L R da Silva reports grants or contracts from SPRINT (Sport Physical Activity and Health Research & Innovation Center), Polytechnic of Guarda (6300-559 Guarda, Portugal) and RISE-Health, Faculty of Health Sciences, University of Beira Interior (6201-506 Covilhã, Portugal), outside the submitted work. J Singh reports consulting fees from ROMTech, Atheneum, Clearview Healthcare Partners, American College of Rheumatology, Yale, Hulio, Horizon Pharmaceuticals, DINORA, ANI/Exeltis, USA Inc, Frictionless Solutions, Schipher, Crealta/Horizon, Medisys, Fidia, PK Med, Two labs, Adept Field Solutions, Clinical Care Options, Putnam Associates, Focus Forward, Navigant Consulting, Spherix, MedIQ, Jupiter Life Science, UBM LLC, Trio Health, Medscape, WebMD, and Practice Point Communications; and the NIH; payment or honoraria for lectures, presentations, speakers bureaus, manuscript writing, or educational events from Simply Speaking; support for attending meetings or travel, or both, from Simply Speaking; leadership or fiduciary roles in other board, society, committee, or advocacy groups, paid or unpaid as a past steering committee member of OMERACT, an international organization that develops measures for clinical trials and receives arm's length funding from 12 pharmaceutical companies, and as Chair of the Veterans Affairs Rheumatology Field Advisory Committee, and as editor and Director of the UAB Cochrane Musculoskeletal Group Satellite Center on Network Meta-analysis; stock or stock options in Atai Life Sciences, Kintara Therapeutics, Intelligent Biosolutions, Acumen Pharmaceutical, TPT Global Tech, Vaxart Pharmaceuticals, Atyu Biopharma, Adaptimmune Therapeutics, GeoVax Labs, Pieris Pharmaceuticals, Enzolytics, Seres Therapeutics, Tonix Pharmaceuticals Holding Corp, Aebona Pharmaceuticals, and Charlotte's Web Holdings; and previously owned stock options in Amarin, Viking, and Moderna Pharmaceuticals, outside the submitted work. J H V Ticoalu reports leadership or fiduciary roles in board, society, committee, or advocacy groups, paid or unpaid as Co-founder of Benang Merah Research Center, Indonesia (https://www.benangmerah.net), outside the submitted work. M Titova reports support for the present manuscript from the state assignment of the Ministry of Science and Higher Education of the Russian Federation (theme number 122042600086-7 and number 122042700043-9). E Upadhyay reports the following patents planned, issued or pending with The Office of the Controller General of Patents, Designs & Trade Marks (CGPDTM; https://iprsearch.ipindia.gov.in/PublicSearch/PublicationSearch/ApplicationStatus): “Eco-friendly bio-shoe polish from banana and turmeric” (filed 202511021382), “Honey-based polyherbal syrup composition to treat air pollution-induced inflammation and preparation method thereof” (filed 202511035171), “Process for preparing a caffeine free, antioxidant and nutrient rich beverage” (filed 202511042794), “A system and method of reusable filters for anti-pollution mask” (published 202011003559), “A system and method for electricity generation through crop stubble by using microbial fuel cells” (published 202011008531), “A system for disposed personal protection equipment (PPE) into biofuel through pyrolysis and method” (published 202111005659), “A novel herbal pharmaceutical aid for formulation of gel and method thereof” (published 202111023333), “Herbal drug formulation for treating lung tissue degenerated by particulate matter exposure” (published 202311035276), “A method to transform cow dung into the wall paint by using natural materials and composition thereof” (filed 202311085452), “Biodegradable packaging composition and method of preparation thereof” (filed 202511017848); and leadership or fiduciary roles in other board, society, committee, or advocacy groups as an Executive Council Member for the Indian Meteorological Society, Jaipur Chapter (India) and a Member Secretary for the Department of Science & Technology (DST), Promotion of University Research and Scientific Excellence (PURSE) Program, outside the submitted work.
