# Supplementary material for: Quantifying the fatal and non-fatal burden of disease associated with child growth failure, 2000–2023: a systematic analysis from the Global Burden of Disease Study 2023
Source: Lancet Child Adolesc Health. 2026 Jan;10(1):22–38. doi: 10.1016/S2352-4642(25)00303-7 (PMC12674951; doi:10.1016/S2352-4642(25)00303-7)
Supplement: Supplementary appendix 1 [file mmc1.pdf]

THE LANCET
Child & Adolescent Health

Supplementary appendix 1

This appendix formed part of the original submission and has been peer reviewed. We post it as supplied by the authors.

Supplement to: GBD 2023 Child Growth Failure Collaborators. Quantifying the fatal and non-fatal burden of disease associated with child growth failure, 2000–2023: a systematic analysis from the Global Burden of Disease Study 2023. *Lancet Child Adolesc Health* 2026; **10:** 22–38.

Appendix 1: Figure and table appendix to "Quantifying the fatal and non-fatal burden of disease associated with child growth failure, 2000–2023: a systematic analysis from the Global Burden of Disease Study 2023"

This appendix provides additional figures and tables for "Quantifying the fatal and non-fatal burden of disease associated with child growth failure, 2000–2023: a systematic analysis from the Global Burden of Disease Study 2023"

Table of Contents

Figures 1

Figure S1. The percentage of diarrheal disease deaths and years lived with disability among children under 5 years associated with child growth failure, globally, in 2023 3

Figure S2. The percentage of lower respiratory infection deaths and years lived with disability among children under 5 years associated with child growth failure, globally, in 2023 4

Figure S3. The percentage of malaria deaths and years lived with disability among children under 5 years associated with child growth failure, globally, in 2023 5

Figure S4. The percentage of measles deaths and years lived with disability among children under 5 years associated with child growth failure, globally, in 2023.. 6

Figure S5. The attributable fraction of infectious disease deaths by cause for child growth failure among children under 5 by country and GBD super-region in 2023 7

Tables 8

Table S1. All-cause and cause-specific DALYs associated with attributable to child growth failure at the global, super-regional, regional, and national levels among children under 5 years, 2023 ..8

Table S2. All-cause and cause-specific population attributable fraction (PAF, %) of DALYs among children under 5 years for child growth failure at the global and super-regional, regional, and national levels, 2023.. 49

Table S3. All-cause and cause-specific deaths associated with attributable to child growth failure at the global, super-regional, regional, and national levels among children under 5 years, 2023 90

Table S4. All-cause and cause-specific population attributable fraction (PAF, %) of deaths among children under 5 years for child growth failure at the global and super-regional, regional, and national levels, 2023 131

Table S5. All-cause and cause-specific YLDs associated with attributable to child growth failure at the global, super-regional, regional, and national levels among children under 5 years, 2023 172

Table S6. All-cause and cause-specific population attributable fraction (PAF, %) of YLDs among children under 5 years for child growth failure at the global and super-regional, regional, and national levels, 2023 213

Table S7. Interventions and strategies to reduce the burden of childhood growth failure 254

Table S8. Comparison of all-cause population attributable fractions for stunting, underweight, and wasting deaths among children younger than 5 years between current and previous iterations of the Global Burden of Disease study and with the Maternal and Child Nutrition Study Group estimates published in 2013 255

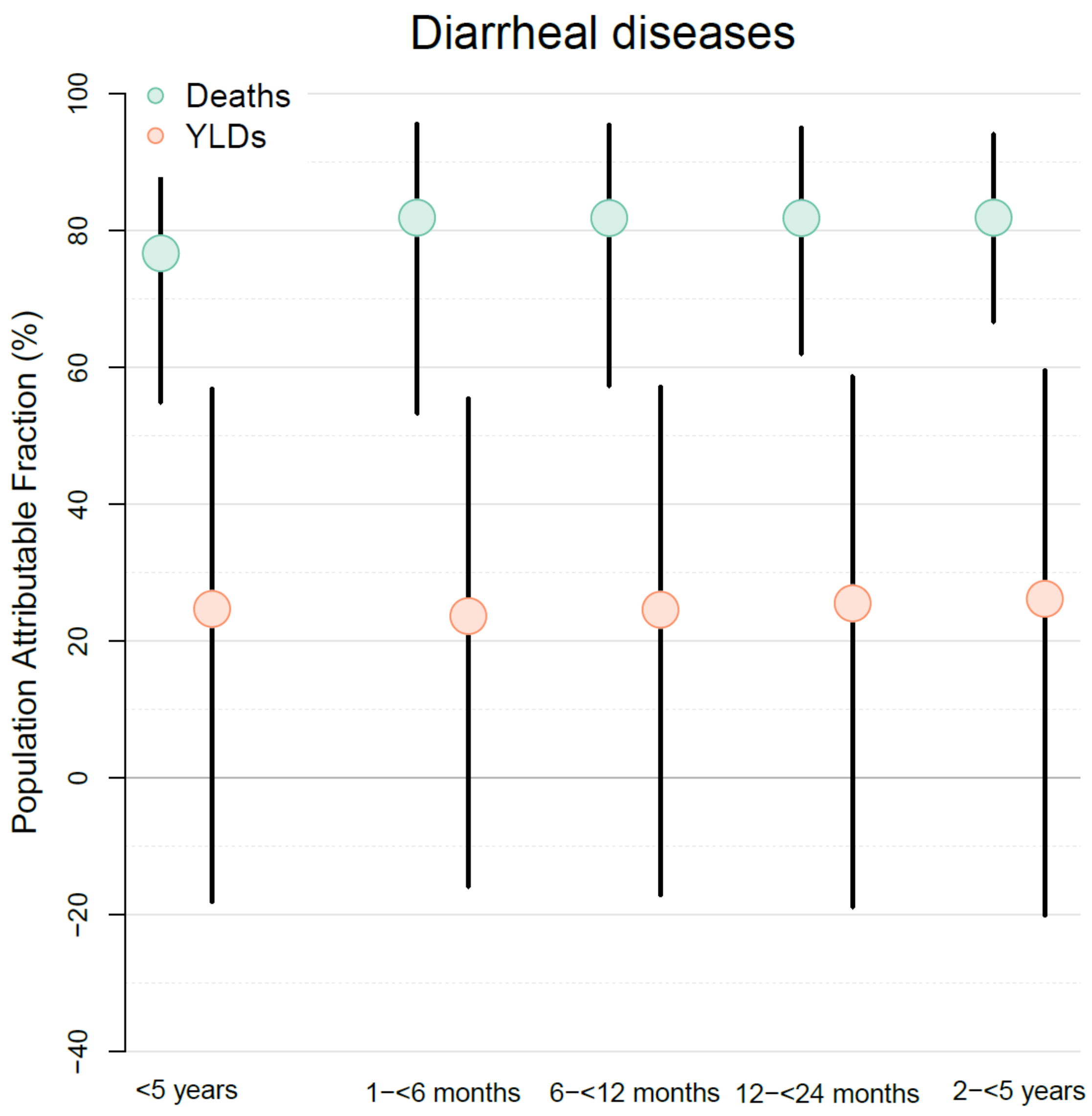

Figure S1. The percentage of diarrheal disease deaths and years lived with disability among children under 5 years associated with child growth failure, globally, in 2023

Population attributable fraction of cause-specific deaths and years lived with disability for all children under 5, as well as age specific (1–<6 months, 6–<12 months, 12–<24 months, and 2–<5 years) are plotted for diarrheal diseases. Dots represent mean PAFs and whiskers represent 95% uncertainty intervals.

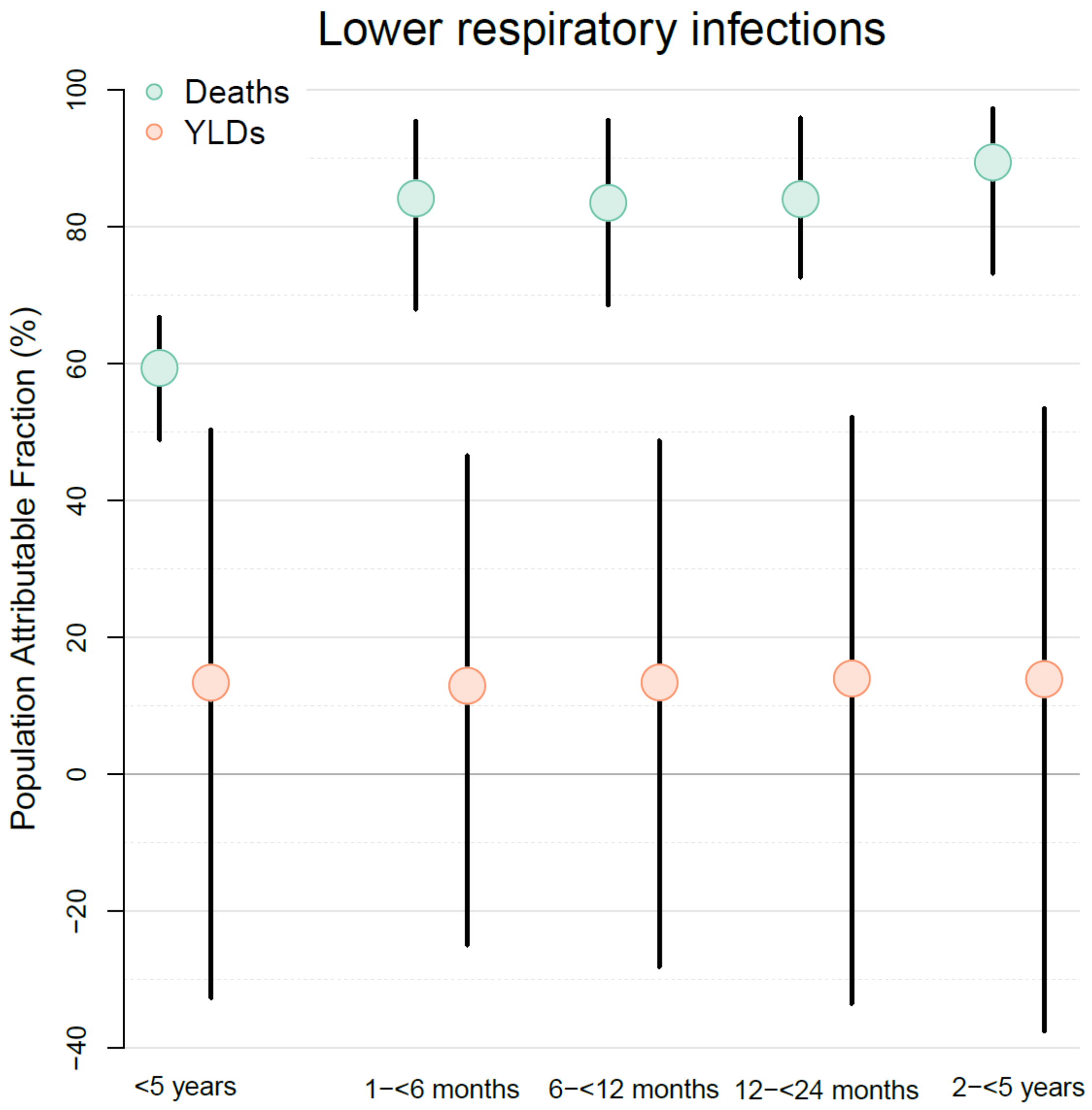

Figure S2. The percentage of lower respiratory infection deaths and years lived with disability among children under 5 years associated with child growth failure, globally, in 2023

Population attributable fraction of cause-specific deaths and years lived with disability for all children under 5, as well as age specific (1–<6 months, 6–<12 months, 12–<24 months, and 2–<5 years) are plotted for lower respiratory infections. Dots represent mean PAFs and whiskers represent 95% uncertainty intervals.

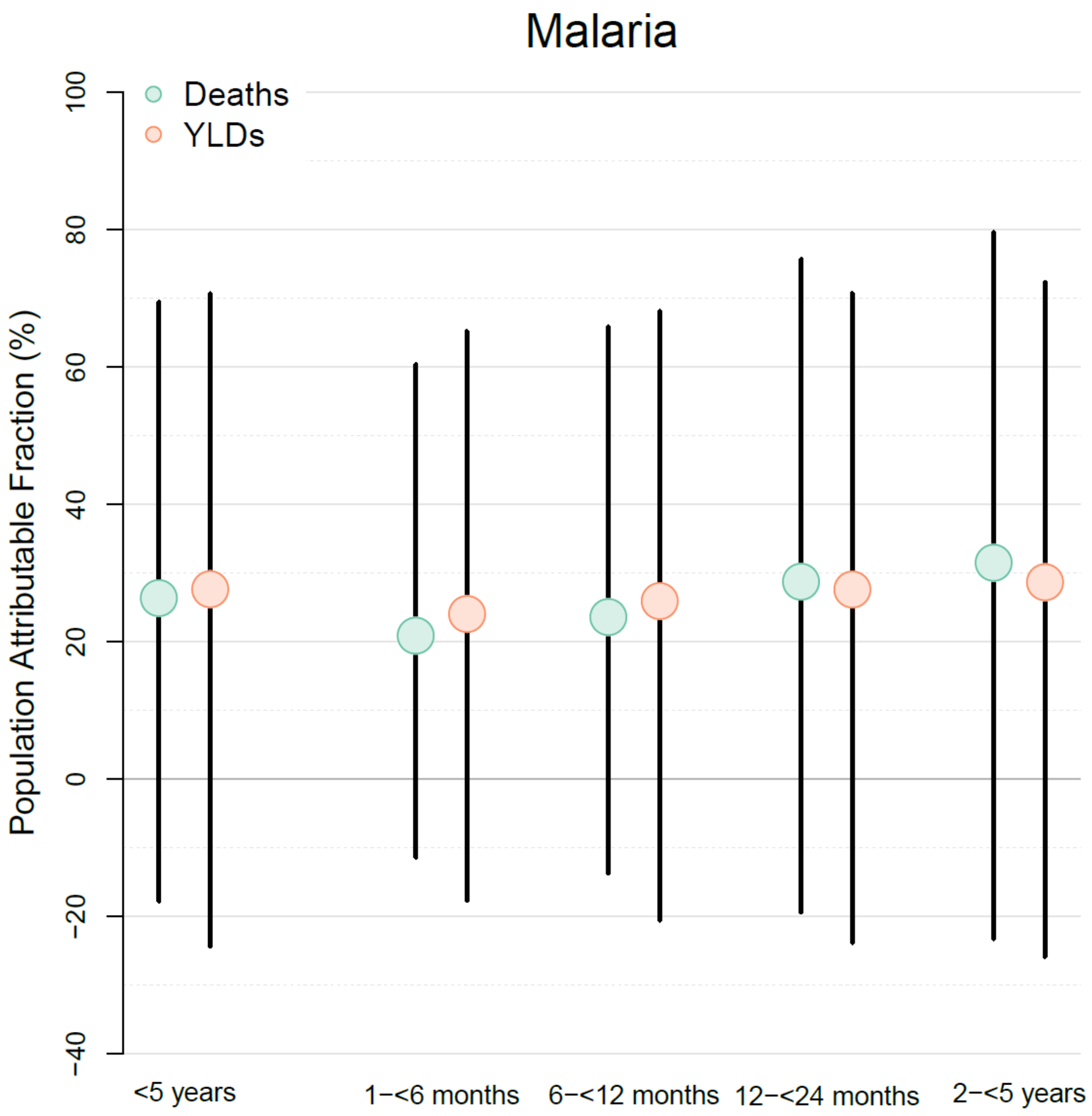

Figure S3. The percentage of malaria deaths and years lived with disability among children under 5 years associated with child growth failure, globally, in 2023

Population attributable fraction of cause-specific deaths and years lived with disability for all children under 5, as well as age specific (1–<6 months, 6–<12 months, 12–<24 months, and 2–<5 years) are plotted for malaria. Dots represent mean PAFs and whiskers represent 95% uncertainty intervals.

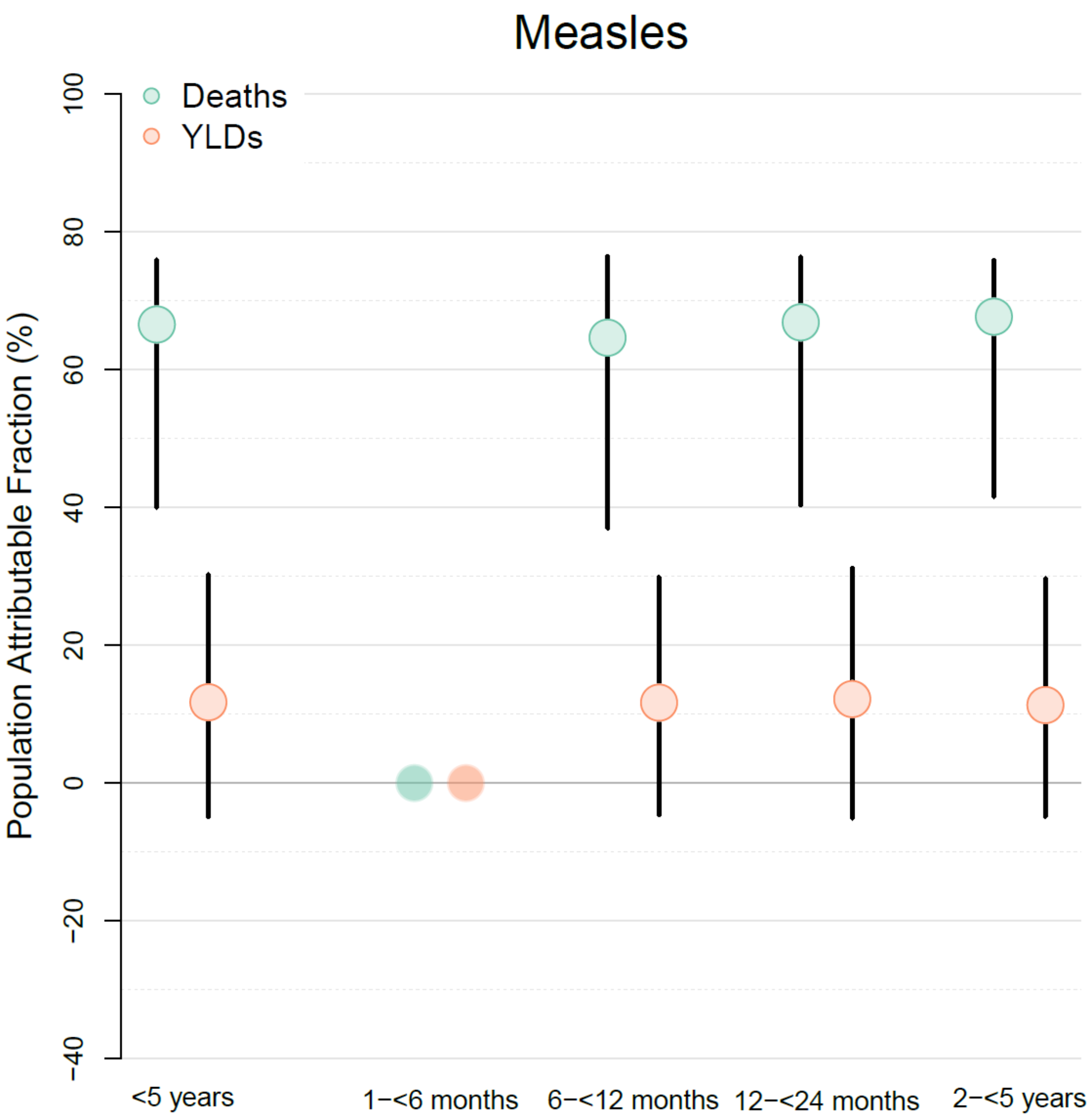

Figure S4. The percentage of measles deaths and years lived with disability among children under 5 years associated with child growth failure, globally, in 2023

Population attributable fraction of cause-specific deaths and years lived with disability for all children under 5, as well as age specific (1–<6 months, 6–<12 months, 12–<24 months, and 2–<5 years) are plotted for measles. Dots represent mean PAFs and whiskers represent 95% uncertainty intervals.

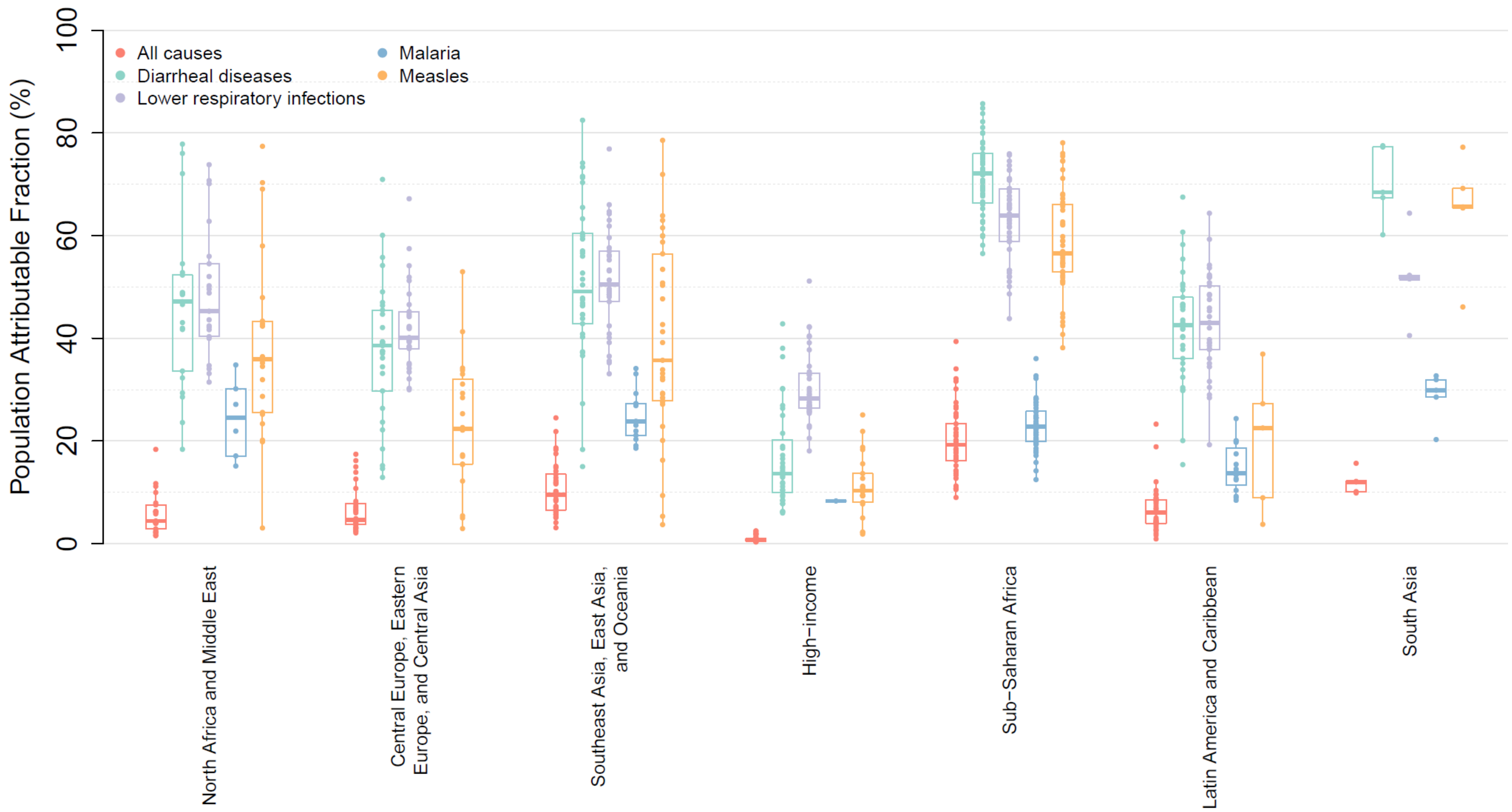

Figure S5. The attributable fraction of infectious disease deaths by cause for child growth failure among children under 5 by country and GBD super-region in 2023

All-cause and cause-specific population attributable fractions are plotted for every country, grouped by super-region. Super-region-level boxplot summaries are overlaid over the country-level mean PAF estimates for each cause/super-region group

Table S1. All-cause and cause-specific DALYs associated with child growth failure at the global, super-regional, regional, and national levels among children under 5 years, 2023 Estimates combine burden associated with mild, moderate, and severe forms of CGF: stunting was defined as height-for-age z-score (HAZ) < –1; underweight as weight-for-age z-score (WAZ) < –1; wasting as weight-for-height z-score (WHZ) < –1, according to WHO Child Growth Standards.

Location	Cause name	Child growth failure	Child underweight	Child wasting	Child stunting
**Global**	**All causes**	**79 400 000 (47 000 000–106 000 000)**	**52 200 000 (21 900 000–75 100 000)**	**39 200 000 (23 800 000–53 000 000)**	**33 000 000 (24 100 000–42 200 000)**
**Global**	**Diarrheal diseases**	**21 800 000 (13 000 000–32 200 000)**	**10 600 000 (5 870 000–17 400 000)**	**15 100 000 (-1 090 000–29 200 000)**	**7 580 000 (4 290 000–11 700 000)**
**Global**	**Lower respiratory infections**	**32 100 000 (22 400 000–41 200 000)**	**22 100 000 (1 690 000–38 000 000)**	**12 500 000 (7 900 000–18 100 000)**	**17 300 000 (12 200 000–23 400 000)**
**Global**	**Malaria**	**9 600 000 (-6 360 000–30 400 000)**	**6 980 000 (-4 070 000–20 500 000)**	**--**	**4 460 000 (-2 350 000–19 700 000)**
**Global**	**Measles**	**6 930 000 (2 700 000–13 300 000)**	**3 560 000 (1 330 000–6 880 000)**	**2 590 000 (782 000–5 530 000)**	**3 690 000 (1 320 000–7 100 000)**
**Central Europe, Eastern Europe, and Central Asia**	**All causes**	**792 000 (577 000–1 030 000)**	**442 000 (54 500–876 000)**	**314 000 (246 000–393 000)**	**303 000 (198 000–406 000)**
**Central Europe, Eastern Europe, and Central Asia**	**Diarrheal diseases**	**70 500 (32 900–105 000)**	**22 000 (12 500–34 800)**	**51 000 (-2 120–97 000)**	**17 900 (11 800–25 600)**
**Central Europe, Eastern Europe, and Central Asia**	**Lower respiratory infections**	**706 000 (526 000–920 000)**	**405 000 (15 000–841 000)**	**248 000 (158 000–342 000)**	**285 000 (184 000–385 000)**
**Central Europe, Eastern Europe, and Central Asia**	**Malaria**	**0 (0–0)**	**0 (0–0)**	**--**	**0 (0–0)**
**Central Europe, Eastern Europe, and Central Asia**	**Measles**	**1 040 (501–1 450)**	**351 (163–510)**	**374 (140–751)**	**510 (233–737)**
Central Asia	All causes	710 000 (517 000–917 000)	400 000 (44 700–789 000)	282 000 (216 000–358 000)	270 000 (176 000–365 000)
Central Asia	Diarrheal diseases	58 700 (28 400–87 600)	19 400 (10 500–31 600)	42 800 (-1 790–82 000)	14 800 (9 100–21 900)
Central Asia	Lower respiratory infections	641 000 (477 000–834 000)	371 000 (13 900–761 000)	229 000 (146 000–316 000)	254 000 (165 000–347 000)
Central Asia	Malaria	0 (0–0)	0 (0–0)	--	0 (0–0)
Central Asia	Measles	1 010 (487–1 420)	341 (160–496)	365 (138–728)	493 (226–716)
Armenia	All causes	5 060 (3 760–6 760)	2 590 (192–5 650)	1 560 (1 080–2 150)	2 440 (1 600–3 370)
Armenia	Diarrheal diseases	246 (107–372)	59·3 (36·1–91·4)	165 (-5·55–343)	78·4 (54·5–111)
Armenia	Lower respiratory infections	4 780 (3 600–6 390)	2 500 (89·1–5 570)	1 360 (806–2 020)	2 360 (1 510–3 270)
Armenia	Malaria	0 (0–0)	0 (0–0)	--	0 (0–0)
Armenia	Measles	0·0488 (-0·00799–0·14)	0·00104 (<0.001–0·00225)	0·0363 (-0·0098–0·101)	0·0127 (>-0.001–0·038)
Azerbaijan	All causes	84 600 (59 000–112 000)	48 800 (8 770–90 600)	35 400 (25 200–45 500)	32 500 (18 900–47 600)
Azerbaijan	Diarrheal diseases	12 500 (5 730–22 800)	4 230 (1 980–7 680)	8 920 (-365–21 700)	3 330 (1 710–5 640)
Azerbaijan	Lower respiratory infections	69 900 (48 000–95 100)	42 900 (1 660–86 300)	24 800 (14 600–35 400)	28 800 (15 800–42 400)
Azerbaijan	Malaria	0 (0–0)	0 (0–0)	--	0 (0–0)
Azerbaijan	Measles	798 (380–1 140)	277 (125–404)	283 (105–594)	391 (182–580)

Georgia	All causes	1 950 (1 310–3 040)	976 (56·5–2 450)	452 (296–653)	892 (675–1 190)
Georgia	Diarrheal diseases	76·6 (16·0–152)	19·8 (12·7–29·6)	43·8 (-2·22–112)	22·5 (5·98–40·2)
Georgia	Lower respiratory infections	1 860 (1 270–2 900)	938 (25·8–2 390)	389 (215–593)	870 (638–1 180)
Georgia	Malaria	0 (0–0)	0 (0–0)	--	0 (0–0)
Georgia	Measles	0·00157 (>-0.001–0·00425)	<0.001 (<0.001–<0.001)	0·00107 (>-0.001–0·003)	<0.001 (<0.001–0·00121)
Kazakhstan	All causes	40 900 (28 600–56 700)	23 000 (3 960–48 600)	15 900 (11 500–21 300)	15 000 (9 400–21 900)
Kazakhstan	Diarrheal diseases	2 150 (840–3 400)	521 (322–779)	1 530 (-54·6–3 130)	526 (359–711)
Kazakhstan	Lower respiratory infections	36 100 (25 500–50 500)	19 800 (705–45 300)	11 700 (6 860–16 900)	14 500 (8 810–21 200)
Kazakhstan	Malaria	0 (0–0)	0 (0–0)	--	0 (0–0)
Kazakhstan	Measles	28·4 (5·79–60·9)	7·43 (1·21–18·5)	9·98 (2·44–22·6)	14·3 (2·6–32·5)
Kyrgyzstan	All causes	25 700 (17 600–36 400)	13 300 (1 660–30 300)	8 010 (6 250–10 200)	11 100 (7 670–15 700)
Kyrgyzstan	Diarrheal diseases	2 920 (1 300–4 680)	682 (371–1 050)	1 950 (-46·8–4 430)	841 (545–1 190)
Kyrgyzstan	Lower respiratory infections	22 200 (15 500–32 300)	12 000 (391–28 900)	5 490 (3 240–8 090)	10 300 (6 970–14 600)
Kyrgyzstan	Malaria	0 (0–0)	0 (0–0)	--	0 (0–0)
Kyrgyzstan	Measles	39·1 (12·0–83·6)	10·9 (3·27–23·1)	10·4 (2·82–23·0)	22·9 (6·8–51·2)
Mongolia	All causes	7 430 (4 980–11 300)	3 780 (263–9 470)	1 450 (918–2 070)	3 690 (2 470–5 330)
Mongolia	Diarrheal diseases	247 (102–496)	53·3 (23·5–112)	152 (-3·15–459)	76·0 (37·5–140)
Mongolia	Lower respiratory infections	7 090 (4 660–10 900)	3 630 (120–9 330)	1 200 (614–1 910)	3 610 (2 430–5 250)
Mongolia	Malaria	0 (0–0)	0 (0–0)	--	0 (0–0)
Mongolia	Measles	0 (0–0)	0 (0–0)	0 (0–0)	0 (0–0)
Tajikistan	All causes	117 000 (80 300–149 000)	65 900 (17 000–106 000)	58 400 (38 200–73 900)	44 400 (30 400–59 800)
Tajikistan	Diarrheal diseases	31 300 (14 700–51 500)	11 300 (5 760–19 000)	23 100 (-1 090–47 000)	7 900 (4 570–12 200)
Tajikistan	Lower respiratory infections	83 600 (60 300–109 000)	52 600 (3 020–96 600)	33 300 (19 300–50 000)	36 400 (24 400–50 200)
Tajikistan	Malaria	0 (0–0)	0 (0–0)	--	0 (0–0)
Tajikistan	Measles	46·7 (24·0–68·6)	18·5 (9–29·3)	18·5 (7·35–35·7)	22·2 (10·1–33·4)
Turkmenistan	All causes	52 700 (35 900–69 700)	30 400 (1 830–59 100)	20 100 (13 500–27 100)	19 200 (13 700–26 100)
Turkmenistan	Diarrheal diseases	2 240 (974–3 340)	636 (344–1 040)	1 680 (-46–3 240)	446 (270–665)
Turkmenistan	Lower respiratory infections	50 000 (34 400–66 500)	29 300 (947–57 900)	18 000 (10 600–25 400)	18 700 (13 400–25 400)
Turkmenistan	Malaria	0 (0–0)	0 (0–0)	--	0 (0–0)

Turkmenistan	Measles	0<br>(0–0)	0<br>(0–0)	0<br>(0–0)	0<br>(0–0)
Uzbekistan	All causes	375 000<br>(265 000–497 000)	212 000<br>(10 700–450 000)	140 000<br>(91 800–193 000)	140 000<br>(87 700–194 000)
Uzbekistan	Diarrheal diseases	7 020<br>(2 900–10 700)	1 850<br>(1 080–2 860)	5 230<br>(-185–10 100)	1 550<br>(1 050–2 140)
Uzbekistan	Lower respiratory infections	366 000<br>(261 000–484 000)	208 000<br>(6 910–447 000)	133 000<br>(81 200–188 000)	139 000<br>(86 200–192 000)
Uzbekistan	Malaria	0<br>(0–0)	0<br>(0–0)	--	0<br>(0–0)
Uzbekistan	Measles	99·3<br>(32·5–246)	27·9<br>(8·77–70·4)	43·1<br>(12·0–123)	42·3<br>(13·6–99·4)
Central Europe	All causes	33 600<br>(22 400–47 600)	16 400<br>(3 210–33 800)	13 700<br>(9 190–17 400)	12 400<br>(8 460–16 800)
Central Europe	Diarrheal diseases	7 450<br>(2 620–12 200)	1 620<br>(1 060–2 290)	5 380<br>(-199–11 200)	1 740<br>(1 220–2 300)
Central Europe	Lower respiratory infections	25 100<br>(18 700–34 700)	13 700<br>(488–31 100)	7 310<br>(4 460–10 100)	10 600<br>(6 600–14 800)
Central Europe	Malaria	0<br>(0–0)	0<br>(0–0)	--	0<br>(0–0)
Central Europe	Measles	28·7<br>(10·8–61·4)	8·87<br>(3·05–19·8)	7·93<br>(2·4–21·6)	16·0<br>(5·87–34·7)
Albania	All causes	1 290<br>(793–1 930)	681<br>(138–1 510)	601<br>(394–836)	549<br>(286–868)
Albania	Diarrheal diseases	121<br>(33·8–246)	29·4<br>(15·8–53·9)	85·5<br>(-3·99–217)	39·0<br>(17·2–68·8)
Albania	Lower respiratory infections	1 060<br>(610–1 640)	537<br>(17·4–1 350)	401<br>(182–657)	510<br>(236–832)
Albania	Malaria	0<br>(0–0)	0<br>(0–0)	--	0<br>(0–0)
Albania	Measles	0·0314<br>(0–0·105)	0·00686<br>(0–0·022)	0·0142<br>(0–0·0491)	0·0167<br>(0–0·0543)
Bosnia and Herzegovina	All causes	409<br>(243–604)	168<br>(46·4–367)	187<br>(103–264)	166<br>(114–229)
Bosnia and Herzegovina	Diarrheal diseases	125<br>(33·5–213)	26·3<br>(15·8–43·2)	87·4<br>(-4·08–187)	36·9<br>(18·9–56·1)
Bosnia and Herzegovina	Lower respiratory infections	264<br>(184–392)	123<br>(3·79–330)	80·5<br>(41·3–118)	129<br>(82·2–183)
Bosnia and Herzegovina	Malaria	0<br>(0–0)	0<br>(0–0)	--	0<br>(0–0)
Bosnia and Herzegovina	Measles	0·0306<br>(0–0·092)	0·00509<br>(0–0·0158)	0·0102<br>(0–0·0349)	0·0183<br>(0–0·057)
Bulgaria	All causes	2 780<br>(1 760–4 350)	1 390<br>(216–3 370)	1 120<br>(833–1 370)	846<br>(575–1 200)
Bulgaria	Diarrheal diseases	426<br>(134–745)	86·9<br>(52·3–135)	321<br>(-11·1–706)	74·9<br>(49·7–109)
Bulgaria	Lower respiratory infections	2 270<br>(1 510–3 620)	1 220<br>(34·8–3 210)	716<br>(407–1 020)	771<br>(517–1 100)
Bulgaria	Malaria	0<br>(0–0)	0<br>(0–0)	--	0<br>(0–0)
Bulgaria	Measles	0<br>(0–0)	0<br>(0–0)	0<br>(0–0)	0<br>(0–0)
Croatia	All causes	685<br>(424–1 010)	291<br>(67·6–624)	309<br>(181–417)	243<br>(178–324)
Croatia	Diarrheal diseases	198<br>(58·7–333)	40·9<br>(27·9–59·2)	144<br>(-6·56–300)	45·7<br>(31·4–62·1)
Croatia	Lower respiratory infections	471<br>(324–675)	234<br>(6·98–577)	149<br>(84·0–219)	197<br>(140–268)

Croatia	Malaria	0 (0–0)	0 (0–0)	--	0 (0–0)
Croatia	Measles	0 (0–0)	0 (0–0)	0 (0–0)	0 (0–0)
Czechia	All causes	2 510 (1 370–3 830)	1 090 (431–2 080)	1 490 (650–2 210)	533 (385–733)
Czechia	Diarrheal diseases	1 110 (321–1 800)	254 (159–380)	861 (-36·2–1 680)	162 (109–228)
Czechia	Lower respiratory infections	1 260 (844–1 920)	686 (21·7–1 670)	475 (270–684)	371 (258–533)
Czechia	Malaria	0 (0–0)	0 (0–0)	--	0 (0–0)
Czechia	Measles	0·002 (<0.001–0·00399)	<0.001 (<0.001–<0.001)	0·00106 (<0.001–0·00246)	<0.001 (<0.001–0·00133)
Hungary	All causes	1 990 (1 020–3 170)	608 (294–1 110)	1 170 (258–2 230)	605 (401–833)
Hungary	Diarrheal diseases	1 240 (402–2 180)	226 (145–340)	908 (-34·3–2 020)	300 (199–415)
Hungary	Lower respiratory infections	703 (490–1 060)	335 (9·39–885)	217 (119–328)	306 (201–431)
Hungary	Malaria	0 (0–0)	0 (0–0)	--	0 (0–0)
Hungary	Measles	0 (0–0)	0 (0–0)	0 (0–0)	0 (0–0)
Montenegro	All causes	150 (94·1–233)	77·2 (13·0–183)	51·1 (37·9–68·2)	61·9 (36·6–97·4)
Montenegro	Diarrheal diseases	16·4 (4·55–33·4)	3·86 (2·46–6·68)	10·4 (-0·479–27·7)	4·94 (2·43–8·09)
Montenegro	Lower respiratory infections	126 (79·0–197)	65·3 (2·3–170)	32·8 (17·9–54·2)	57·0 (30·9–91·5)
Montenegro	Malaria	0 (0–0)	0 (0–0)	--	0 (0–0)
Montenegro	Measles	0 (0–0)	0 (0–0)	0 (0–0)	0 (0–0)
North Macedonia	All causes	475 (270–691)	157 (54·1–333)	275 (120–421)	151 (97·3–226)
North Macedonia	Diarrheal diseases	207 (45·9–362)	30·5 (18·7–48·9)	161 (-5·66–330)	46·8 (27·6–69·0)
North Macedonia	Lower respiratory infections	246 (175–350)	105 (2·95–278)	91·9 (52·8–140)	104 (58·9–163)
North Macedonia	Malaria	0 (0–0)	0 (0–0)	--	0 (0–0)
North Macedonia	Measles	0·239 (0·08–0·425)	0·0457 (0·015–0·0823)	0·0847 (0·0237–0·191)	0·131 (0·0401–0·25)
Poland	All causes	4 820 (2 960–7 160)	1 930 (483–4 310)	2 270 (1 160–3 410)	1 680 (1 190–2 330)
Poland	Diarrheal diseases	1 610 (567–2 770)	294 (174–451)	1 200 (-37·5–2 640)	362 (247–503)
Poland	Lower respiratory infections	3 100 (2 100–4 500)	1 520 (45·0–3 900)	950 (549–1 390)	1 320 (917–1 860)
Poland	Malaria	0 (0–0)	0 (0–0)	--	0 (0–0)
Poland	Measles	0·0141 (0·00574–0·0276)	0·00296 (0·0011–0·0057)	0·00564 (0·00186–0·0118)	0·00679 (0·00252–0·0127)
Romania	All causes	13 900 (9 750–19 600)	7 870 (994–16 000)	4 320 (3 270–5 490)	5 890 (3 530–8 350)
Romania	Diarrheal diseases	1 390 (636–2 260)	401 (270–583)	868 (-31·3–2 010)	419 (306–558)

Romania	Lower respiratory infections	12 200 (8 760–17 000)	7 140 (282–15 300)	3 120 (1 790–4 530)	5 460 (3 100–7 840)
Romania	Malaria	0 (0–0)	0 (0–0)	--	0 (0–0)
Romania	Measles	27·4 (10·1–59·5)	8·51 (2·92–19·2)	7·52 (2·2–20·8)	15·2 (5·5–33·6)
Serbia	All causes	1 190 (756–1 720)	575 (151–1 160)	538 (326–755)	444 (318–618)
Serbia	Diarrheal diseases	324 (89·5–575)	82·1 (52·3–137)	225 (-11–506)	86·5 (43·7–140)
Serbia	Lower respiratory infections	794 (539–1 170)	426 (17·4–1 000)	246 (143–378)	357 (235–527)
Serbia	Malaria	0 (0–0)	0 (0–0)	--	0 (0–0)
Serbia	Measles	0·613 (0·23–0·978)	0·159 (0·0599–0·266)	0·175 (0·0531–0·367)	0·349 (0·119–0·61)
Slovakia	All causes	2 560 (1 680–3 620)	1 160 (182–2 660)	1 090 (764–1 390)	932 (607–1 320)
Slovakia	Diarrheal diseases	546 (166–943)	111 (65·4–183)	406 (-12·5–877)	127 (79·4–191)
Slovakia	Lower respiratory infections	1 970 (1 460–2 840)	1 010 (31·3–2 500)	642 (377–920)	806 (488–1 160)
Slovakia	Malaria	0 (0–0)	0 (0–0)	--	0 (0–0)
Slovakia	Measles	0 (0–0)	0 (0–0)	0 (0–0)	0 (0–0)
Slovenia	All causes	274 (175–421)	127 (12·5–324)	106 (76·7–137)	99·3 (71·1–136)
Slovenia	Diarrheal diseases	31·2 (4·88–58·9)	6·62 (3·94–10·9)	21·4 (-1·46–45·3)	7·15 (2·33–12·5)
Slovenia	Lower respiratory infections	238 (165–369)	115 (2·94–310)	79·6 (45·5–115)	92·1 (62·9–129)
Slovenia	Malaria	0 (0–0)	0 (0–0)	--	0 (0–0)
Slovenia	Measles	0 (0–0)	0 (0–0)	0 (0–0)	0 (0–0)
Eastern Europe	All causes	48 300 (36 500–64 700)	25 400 (6 400–52 200)	18 500 (14 700–23 200)	21 000 (13 900–28 500)
Eastern Europe	Diarrheal diseases	4 380 (1 620–7 000)	1 040 (747–1 480)	2 850 (-125–6 100)	1 420 (962–1 910)
Eastern Europe	Lower respiratory infections	39 400 (29 700–53 900)	19 900 (629–47 100)	11 200 (6 650–16 100)	19 600 (12 400–27 100)
Eastern Europe	Malaria	0 (0–0)	0 (0–0)	--	0 (0–0)
Eastern Europe	Measles	2·1 (0·617–4·48)	0·367 (0·146–0·783)	1·04 (0·0238–2·49)	0·826 (0·329–1·8)
Belarus	All causes	920 (498–1 510)	571 (66·9–1 290)	282 (206–373)	271 (192–378)
Belarus	Diarrheal diseases	123 (0·591–282)	54·6 (13·9–118)	54·8 (-7·59–133)	27·1 (-4·12–64·2)
Belarus	Lower respiratory infections	760 (465–1 210)	481 (15·9–1 120)	191 (109–289)	244 (150–361)
Belarus	Malaria	0 (0–0)	0 (0–0)	--	0 (0–0)
Belarus	Measles	0·497 (0·148–1·19)	0·214 (0·0598–0·544)	0·131 (0·0346–0·348)	0·187 (0·0607–0·444)
Estonia	All causes	146 (78·7–232)	74·3 (5·06–181)	45·1 (32·0–57·8)	56·0 (42·7–72·2)

Estonia	Diarrheal diseases	20·5 (-3·37–50·5)	7·21 (0·965–17·1)	9·38 (-1·89–22·4)	5·83 (-2·16–15·6)
Estonia	Lower respiratory infections	123 (78·1–189)	64·8 (1·93–164)	33·3 (18·3–49·9)	50·1 (35·0–69·9)
Estonia	Malaria	0 (0–0)	0 (0–0)	--	0 (0–0)
Estonia	Measles	0·00245 (<0.001–0·00528)	<0.001 (<0.001–0·00152)	0·00101 (<0.001–0·00258)	0·00102 (<0.001–0·002)
Latvia	All causes	244 (153–385)	130 (7·84–311)	70·3 (47·8–96·6)	96·1 (70·7–132)
Latvia	Diarrheal diseases	20·7 (-1·98–49·1)	7·43 (1·52–16·6)	9·66 (-1·62–23·0)	5·89 (-1·48–14·8)
Latvia	Lower respiratory infections	220 (149–337)	119 (3·55–291)	57·6 (32·4–88·1)	90·2 (61·4–127)
Latvia	Malaria	0 (0–0)	0 (0–0)	--	0 (0–0)
Latvia	Measles	0 (0–0)	0 (0–0)	0 (0–0)	0 (0–0)
Lithuania	All causes	254 (134–407)	123 (12·1–297)	86·4 (54·3–112)	93·6 (69·1–126)
Lithuania	Diarrheal diseases	53·0 (3·63–108)	15·5 (6·62–28·8)	30·2 (-3·2–70·1)	13·7 (1·11–27·0)
Lithuania	Lower respiratory infections	197 (123–302)	104 (2·76–267)	52·8 (29·5–79·6)	80·0 (53·6–115)
Lithuania	Malaria	0 (0–0)	0 (0–0)	--	0 (0–0)
Lithuania	Measles	<0.001 (<0.001–<0.001)	<0.001 (<0.001–<0.001)	<0.001 (<0.001–<0.001)	<0.001 (<0.001–<0.001)
Republic of Moldova	All causes	1 560 (1 030–2 360)	830 (75·8–1 880)	514 (383–680)	568 (396–796)
Republic of Moldova	Diarrheal diseases	151 (37·8–270)	39·5 (28·0–52·7)	99·5 (-5·84–222)	34·1 (16·7–46·8)
Republic of Moldova	Lower respiratory infections	1 390 (976–2 090)	771 (25·3–1 810)	395 (232–584)	534 (352–758)
Republic of Moldova	Malaria	0 (0–0)	0 (0–0)	--	0 (0–0)
Republic of Moldova	Measles	0·044 (0·0107–0·115)	0·0123 (0·00281–0·0307)	0·0148 (0·00287–0·0438)	0·0208 (0·00529–0·052)
Russian Federation	All causes	37 200 (27 300–50 400)	18 100 (2 560–40 100)	13 300 (10 000–17 200)	17 400 (11 300–24 200)
Russian Federation	Diarrheal diseases	3 590 (1 400–5 690)	791 (519–1 160)	2 410 (-93·6–5 060)	1 180 (844–1 570)
Russian Federation	Lower respiratory infections	32 200 (24 000–43 700)	15 900 (493–38 000)	9 450 (5 560–13 900)	16 200 (10 100–22 800)
Russian Federation	Malaria	0 (0–0)	0 (0–0)	--	0 (0–0)
Russian Federation	Measles	1·15 (-0·134–3·01)	0·0438 (0·0169–0·0767)	0·802 (-0·16–2·12)	0·349 (0·0219–1·06)
Ukraine	All causes	7 930 (5 990–10 600)	5 570 (2 840–9 120)	4 230 (3 110–5 520)	2 520 (1 760–3 470)
Ukraine	Diarrheal diseases	418 (177–692)	127 (87·6–188)	241 (-11·5–570)	152 (89·2–217)
Ukraine	Lower respiratory infections	4 510 (3 350–6 360)	2 450 (86·7–5 550)	991 (552–1 460)	2 370 (1 600–3 280)
Ukraine	Malaria	0 (0–0)	0 (0–0)	--	0 (0–0)
Ukraine	Measles	0·403 (0·139–1·18)	0·0959 (0·0296–0·286)	0·0927 (0·0275–0·315)	0·268 (0·0903–0·815)

**High-income**	**All causes**	**57 900 (37 100–92 300)**	**30 100 (9 950–64 800)**	**24 800 (18 200–32 900)**	**17 700 (13 900–23 500)**
**High-income**	**Diarrheal diseases**	**11 500 (2 420–23 200)**	**2 750 (1 840–4 370)**	**7 450 (-436–19 100)**	**2 450 (1 130–3 840)**
**High-income**	**Lower respiratory infections**	**38 800 (26 600–64 000)**	**19 800 (514–54 600)**	**9 830 (5 620–14 400)**	**15 200 (10 900–21 100)**
**High-income**	**Malaria**	**0·0753 (-0·0302–0·312)**	**0·0753 (-0·0302–0·312)**	**--**	**<0.001 (>-0.001–<0.001)**
**High-income**	**Measles**	**4·48 (1·66–6·86)**	**1·25 (0·478–2·01)**	**1·77 (0·561–3·58)**	**1·73 (0·654–2·83)**
Australasia	All causes	752 (414–1 370)	259 (60·8–716)	361 (204–595)	234 (181–309)
Australasia	Diarrheal diseases	233 (-10·5–577)	33·1 (11·2–68·4)	160 (-18·6–442)	51·9 (-1·74–119)
Australasia	Lower respiratory infections	474 (336–770)	181 (3·58–607)	156 (81·4–237)	182 (121–268)
Australasia	Malaria	0 (0–0)	0 (0–0)	--	0 (0–0)
Australasia	Measles	0·311 (0·0842–0·666)	0·039 (0·0115–0·0758)	0·147 (0·0343–0·41)	0·134 (0·0379–0·299)
Australia	All causes	517 (276–941)	166 (49·9–451)	271 (151–449)	155 (119–204)
Australia	Diarrheal diseases	171 (-18·1–446)	22·7 (4·89–53·7)	117 (-15·8–330)	38·2 (-6·6–97·1)
Australia	Lower respiratory infections	300 (212–491)	98·5 (1·74–363)	109 (54·9–166)	117 (80·7–170)
Australia	Malaria	0 (0–0)	0 (0–0)	--	0 (0–0)
Australia	Measles	0·288 (0·0766–0·639)	0·0348 (0·00976–0·0715)	0·138 (0·032–0·4)	0·124 (0·0324–0·276)
New Zealand	All causes	236 (135–419)	92·9 (8·94–270)	89·5 (50·2–147)	79·2 (56·4–111)
New Zealand	Diarrheal diseases	62·1 (9·25–138)	10·4 (6·66–16·6)	42·6 (-2·78–114)	13·7 (5·13–23·4)
New Zealand	Lower respiratory infections	173 (118–293)	82·1 (1·85–257)	46·5 (25·6–70·5)	65·4 (37·6–97·8)
New Zealand	Malaria	0 (0–0)	0 (0–0)	--	0 (0–0)
New Zealand	Measles	0·0229 (0·00735–0·048)	0·00416 (0·00127–0·00863)	0·00915 (0·0028–0·02)	0·0105 (0·00297–0·022)
High-income Asia Pacific	All causes	4 830 (2 720–7 840)	2 630 (709–5 600)	2 050 (1 380–2 840)	1 320 (1 030–1 660)
High-income Asia Pacific	Diarrheal diseases	1 310 (107–2 760)	427 (161–843)	756 (-77·8–1 800)	260 (34·3–531)
High-income Asia Pacific	Lower respiratory infections	3 030 (1 950–5 030)	1 720 (46·9–4 460)	807 (444–1 200)	1 060 (754–1 470)
High-income Asia Pacific	Malaria	0·0753 (-0·0302–0·312)	0·0753 (-0·0302–0·312)	--	<0.001 (>-0.001–<0.001)
High-income Asia Pacific	Measles	1·03 (0·384–1·72)	0·346 (0·129–0·6)	0·394 (0·124–0·861)	0·383 (0·142–0·694)
Brunei Darussalam	All causes	96·6 (63·4–138)	47·8 (7·7–104)	28·4 (20·4–37·8)	51·5 (33·0–75·8)
Brunei Darussalam	Diarrheal diseases	12·0 (5·16–21·5)	2·95 (1·61–5·32)	7·17 (-0·266–17·8)	4·93 (2·68–8·3)
Brunei Darussalam	Lower respiratory infections	81·6 (54·0–119)	41·8 (1·64–98·1)	18·2 (9·15–30·1)	46·6 (28·2–71·0)
Brunei Darussalam	Malaria	0 (0–0)	0 (0–0)	--	0 (0–0)

Brunei Darussalam	Measles	0 (0–0)	0 (0–0)	0 (0–0)	0 (0–0)
Japan	All causes	3 740 (2 080–6 100)	2 090 (612–4 450)	1 590 (1 080–2 190)	1 030 (794–1 310)
Japan	Diarrheal diseases	1 030 (27·6–2 300)	360 (106–771)	549 (-68·2–1 350)	220 (8·54–484)
Japan	Lower respiratory infections	2 270 (1 430–3 730)	1 280 (33·9–3 320)	586 (319–881)	809 (563–1 140)
Japan	Malaria	0 (0–0)	0 (0–0)	--	0 (0–0)
Japan	Measles	0·965 (0·351–1·64)	0·325 (0·119–0·565)	0·364 (0·116–0·788)	0·361 (0·134–0·659)
Republic of Korea	All causes	684 (352–1 220)	322 (79·4–739)	323 (160–546)	144 (108–194)
Republic of Korea	Diarrheal diseases	239 (55·0–494)	55·8 (36·6–84·8)	175 (-8·45–435)	29·8 (16·7–43·6)
Republic of Korea	Lower respiratory infections	419 (263–740)	240 (6·79–652)	122 (63·9–188)	115 (81·9–158)
Republic of Korea	Malaria	0·0753 (-0·0302–0·312)	0·0753 (-0·0302–0·312)	--	<0.001 (>-0.001–<0.001)
Republic of Korea	Measles	0·017 (0·00322–0·0393)	0·00523 (0·00108–0·0115)	0·00812 (0·00141–0·0212)	0·00468 (<0.001–0·00928)
Singapore	All causes	305 (169–504)	169 (16·9–416)	109 (70·2–146)	91·3 (53·4–143)
Singapore	Diarrheal diseases	33·9 (11·9–60·7)	8·53 (5·1–13·3)	24·6 (-0·884–56·7)	5·97 (3·44–8·59)
Singapore	Lower respiratory infections	268 (155–444)	157 (4·49–407)	81·1 (41·1–127)	85·3 (47·7–136)
Singapore	Malaria	0 (0–0)	0 (0–0)	--	0 (0–0)
Singapore	Measles	0·049 (0·0163–0·0971)	0·0161 (0·0056–0·0325)	0·0212 (0·00641–0·0519)	0·0172 (0·00613–0·037)
High-income North America	All causes	25 100 (16 900–38 900)	12 900 (4 010–28 800)	11 400 (8 960–13 600)	7 240 (5 420–9 940)
High-income North America	Diarrheal diseases	3 500 (955–6 830)	604 (369–880)	2 670 (-74·9–6 530)	595 (409–832)
High-income North America	Lower respiratory infections	18 400 (12 800–30 000)	9 080 (225–25 400)	5 510 (3 140–8 110)	6 650 (4 820–9 190)
High-income North America	Malaria	0 (0–0)	0 (0–0)	--	0 (0–0)
High-income North America	Measles	1·4 (0·465–2·67)	0·344 (0·114–0·718)	0·644 (0·178–1·39)	0·506 (0·165–1·04)
Canada	All causes	1 390 (648–2 500)	688 (207–1 610)	764 (406–1 240)	150 (112–214)
Canada	Diarrheal diseases	487 (108–1 000)	97·1 (56·7–148)	392 (-11·2–960)	31·8 (19·9–46·8)
Canada	Lower respiratory infections	806 (436–1 590)	490 (11·3–1 440)	271 (150–404)	119 (86·7–171)
Canada	Malaria	0 (0–0)	0 (0–0)	--	0 (0–0)
Canada	Measles	0·11 (0·039–0·226)	0·0271 (0·0107–0·0518)	0·0702 (0·0224–0·173)	0·0164 (0·00631–0·0313)
Greenland	All causes	19·1 (11·0–31·6)	9·68 (2·21–22·9)	6·98 (4·74–9·55)	6·06 (4·1–9·08)
Greenland	Diarrheal diseases	3·11 (1·04–6·56)	0·688 (0·305–1·35)	2·2 (-0·0511–6·16)	0·624 (0·306–1·13)
Greenland	Lower respiratory infections	14·0 (8·04–23·9)	7·84 (0·262–21·1)	3·46 (1·83–5·63)	5·04 (3·21–7·86)

Greenland	Malaria	0 (0–0)	0 (0–0)	--	0 (0–0)
Greenland	Measles	1·02 (0·274–2·19)	0·266 (0·0739–0·615)	0·434 (0·101–1·06)	0·393 (0·119–0·914)
United States of America	All causes	23 700 (16 300–36 100)	12 200 (3 800–27 200)	10 600 (8 420–12 500)	7 090 (5 300–9 740)
United States of America	Diarrheal diseases	3 010 (850–5 810)	507 (310–739)	2 270 (-63·7–5 550)	563 (385–788)
United States of America	Lower respiratory infections	17 600 (12 400–28 300)	8 580 (214–24 000)	5 240 (2 980–7 690)	6 520 (4 720–9 030)
United States of America	Malaria	0 (0–0)	0 (0–0)	--	0 (0–0)
United States of America	Measles	0·268 (0·0933–0·452)	0·0508 (0·0186–0·0825)	0·139 (0·0431–0·296)	0·0961 (0·0344–0·162)
Southern Latin America	All causes	15 800 (11 200–23 400)	8 720 (3 510–17 100)	6 010 (4 770–7 570)	6 090 (4 620–8 170)
Southern Latin America	Diarrheal diseases	2 400 (944–4 400)	593 (439–839)	1 370 (-61–3 620)	745 (507–1 020)
Southern Latin America	Lower respiratory infections	10 600 (7 350–16 500)	5 330 (156–14 000)	1 840 (1 010–2 810)	5 340 (3 860–7 390)
Southern Latin America	Malaria	0 (0–0)	0 (0–0)	--	0 (0–0)
Southern Latin America	Measles	0·0215 (0·00524–0·0481)	0·00895 (0·00234–0·0199)	0·00662 (0·00142–0·018)	0·00699 (0·00149–0·015)
Argentina	All causes	12 600 (8 920–18 400)	6 590 (2 260–13 600)	4 390 (3 560–5 370)	5 390 (4 010–7 330)
Argentina	Diarrheal diseases	1 730 (653–3 110)	388 (281–573)	975 (-42·9–2 550)	604 (399–844)
Argentina	Lower respiratory infections	8 990 (6 400–13 700)	4 340 (125–11 500)	1 560 (847–2 410)	4 790 (3 400–6 660)
Argentina	Malaria	0 (0–0)	0 (0–0)	--	0 (0–0)
Argentina	Measles	0 (0–0)	0 (0–0)	0 (0–0)	0 (0–0)
Chile	All causes	2 630 (1 690–4 100)	1 810 (973–3 030)	1 390 (1 030–1 840)	484 (376–626)
Chile	Diarrheal diseases	511 (166–993)	156 (110–229)	315 (-14·8–828)	84·7 (53·0–129)
Chile	Lower respiratory infections	1 270 (744–2 270)	806 (25·7–2 010)	233 (123–356)	399 (303–533)
Chile	Malaria	0 (0–0)	0 (0–0)	--	0 (0–0)
Chile	Measles	0·0215 (0·00524–0·0481)	0·00895 (0·00234–0·0199)	0·00662 (0·00142–0·018)	0·00699 (0·00149–0·015)
Uruguay	All causes	572 (386–826)	322 (145–574)	225 (142–334)	211 (153–287)
Uruguay	Diarrheal diseases	163 (70·9–270)	49·6 (36·0–69·9)	84·1 (-3·29–217)	56·6 (35·7–74·5)
Uruguay	Lower respiratory infections	318 (218–497)	182 (5·5–435)	49·5 (26·5–74·5)	155 (99·0–223)
Uruguay	Malaria	0 (0–0)	0 (0–0)	--	0 (0–0)
Uruguay	Measles	0 (0–0)	0 (0–0)	0 (0–0)	0 (0–0)
Western Europe	All causes	11 400 (5 530–20 700)	5 590 (1 650–13 000)	5 010 (2 680–8 720)	2 820 (2 200–3 700)
Western Europe	Diarrheal diseases	4 090 (472–8 810)	1 090 (546–1 990)	2 500 (-204–6 650)	799 (140–1 560)

Western Europe	Lower respiratory infections	6 320 (3 900–11 600)	3 500 (82·0–10 400)	1 510 (825–2 230)	2 020 (1 380–2 880)
Western Europe	Malaria	0 (0–0)	0 (0–0)	--	0 (0–0)
Western Europe	Measles	1·72 (0·6–2·76)	0·513 (0·178–0·879)	0·58 (0·181–1·31)	0·703 (0·264–1·21)
Andorra	All causes	1·09 (0·473–2·15)	0·568 (0·0345–1·67)	0·309 (0·198–0·453)	0·335 (0·219–0·54)
Andorra	Diarrheal diseases	0·171 (-0·0413–0·469)	0·0608 (>-0.001–0·155)	0·0748 (-0·0174–0·214)	0·0434 (-0·0231–0·134)
Andorra	Lower respiratory infections	0·896 (0·461–1·73)	0·488 (0·0131–1·47)	0·214 (0·0992–0·382)	0·291 (0·175–0·498)
Andorra	Malaria	0 (0–0)	0 (0–0)	--	0 (0–0)
Andorra	Measles	0 (0–0)	0 (0–0)	0 (0–0)	0 (0–0)
Austria	All causes	104 (23·0–219)	45·1 (4·01–114)	38·1 (9·32–77·2)	30·5 (12·9–52·7)
Austria	Diarrheal diseases	54·7 (-9·1–140)	18·4 (2·36–44·5)	25·9 (-4·96–67·5)	13·6 (-5·93–38·1)
Austria	Lower respiratory infections	47·8 (27·6–84·8)	25·4 (0·557–71·2)	10·9 (5·55–17·4)	17·0 (12·3–24·3)
Austria	Malaria	0 (0–0)	0 (0–0)	--	0 (0–0)
Austria	Measles	0·00531 (-0·0012–0·0153)	<0.001 (<0.001–<0.001)	0·00477 (-0·00151–0·0142)	<0.001 (<0.001–0·0012)
Belgium	All causes	368 (166–667)	158 (42·3–393)	165 (60·9–324)	94·3 (73·3–128)
Belgium	Diarrheal diseases	161 (29·4–341)	38·0 (22·5–60·9)	105 (-6·48–278)	31·1 (11·0–53·6)
Belgium	Lower respiratory infections	193 (117–354)	106 (2·41–322)	46·2 (24·7–68·4)	63·2 (42·5–93·6)
Belgium	Malaria	0 (0–0)	0 (0–0)	--	0 (0–0)
Belgium	Measles	0·00305 (<0.001–0·00746)	<0.001 (<0.001–<0.001)	0·00239 (>-0.001–0·00656)	<0.001 (<0.001–<0.001)
Cyprus	All causes	60·3 (21·2–133)	23·9 (8·18–51·6)	30·5 (6·29–81·4)	14·5 (8·85–20·8)
Cyprus	Diarrheal diseases	36·8 (6·42–90·9)	9·63 (5·12–16·7)	23·5 (-1·3–75·4)	7·37 (2·28–12·9)
Cyprus	Lower respiratory infections	21·9 (11·0–42·7)	12·6 (0·408–38·3)	5·3 (2·55–9·55)	7·15 (3·71–12·7)
Cyprus	Malaria	0 (0–0)	0 (0–0)	--	0 (0–0)
Cyprus	Measles	0 (0–0)	0 (0–0)	0 (0–0)	0 (0–0)
Denmark	All causes	277 (118–490)	114 (33·9–266)	136 (42·3–270)	65·4 (46·1–92·4)
Denmark	Diarrheal diseases	141 (30·8–282)	32·8 (22·0–50·1)	94·6 (-4·64–239)	26·4 (11·4–41·5)
Denmark	Lower respiratory infections	127 (76·8–230)	72·7 (1·76–212)	32·7 (17·1–51·8)	39·0 (20·3–63·9)
Denmark	Malaria	0 (0–0)	0 (0–0)	--	0 (0–0)
Denmark	Measles	0 (0–0)	0 (0–0)	0 (0–0)	0 (0–0)
Finland	All causes	86·9 (37·1–160)	42·2 (5·51–108)	30·8 (15·4–50·3)	25·2 (18·0–34·0)

Finland	Diarrheal diseases	28·0 (-0·114–64·7)	8·68 (2·5–18·7)	15·1 (-1·89–38·1)	6·26 (-0·694–15·4)
Finland	Lower respiratory infections	56·8 (32·1–103)	31·4 (0·79–89·3)	13·7 (7·21–21·5)	18·9 (12·8–27·7)
Finland	Malaria	0 (0–0)	0 (0–0)	--	0 (0–0)
Finland	Measles	0 (0–0)	0 (0–0)	0 (0–0)	0 (0–0)
France	All causes	2 550 (1 470–4 300)	1 470 (740–2 830)	1 370 (880–2 100)	534 (407–697)
France	Diarrheal diseases	778 (55·3–1 700)	209 (99·5–397)	461 (-47·2–1 250)	163 (10·4–340)
France	Lower respiratory infections	1 140 (706–2 060)	621 (14·6–1 830)	274 (150–420)	371 (247–552)
France	Malaria	0 (0–0)	0 (0–0)	--	0 (0–0)
France	Measles	0·0471 (0·0126–0·104)	0·013 (0·00288–0·0301)	0·017 (0·0049–0·0416)	0·0188 (0·004–0·0467)
Germany	All causes	1 670 (540–3 160)	708 (204–1 580)	838 (184–1 750)	347 (245–444)
Germany	Diarrheal diseases	1 000 (136–2 110)	293 (143–539)	623 (-49·3–1 600)	172 (40·6–329)
Germany	Lower respiratory infections	612 (359–1 100)	359 (8–1 010)	159 (88·3–234)	175 (112–262)
Germany	Malaria	0 (0–0)	0 (0–0)	--	0 (0–0)
Germany	Measles	0·123 (0·0456–0·259)	0·0418 (0·0154–0·0874)	0·0453 (0·0146–0·108)	0·0418 (0·016–0·0916)
Greece	All causes	262 (135–466)	138 (5·16–382)	70·4 (51·2–92·4)	85·0 (63·0–120)
Greece	Diarrheal diseases	37·0 (-6·84–93·1)	13·1 (1·49–31·1)	16·0 (-3·27–40·3)	10·2 (-4·52–28·1)
Greece	Lower respiratory infections	224 (137–388)	124 (2·96–353)	53·7 (28·7–82·1)	74·8 (41·7–115)
Greece	Malaria	0 (0–0)	0 (0–0)	--	0 (0–0)
Greece	Measles	0 (0–0)	0 (0–0)	0 (0–0)	0 (0–0)
Iceland	All causes	14·6 (7·08–27·6)	6·98 (1·11–18·4)	5·52 (2·91–8·77)	3·98 (3·06–5·39)
Iceland	Diarrheal diseases	4·26 (0·299–9·37)	1·11 (0·467–2·12)	2·58 (-0·243–6·79)	0·873 (0·0727–1·82)
Iceland	Lower respiratory infections	9·87 (5·91–18·0)	5·42 (0·139–16·1)	2·49 (1·35–3·7)	3·11 (1·9–4·68)
Iceland	Malaria	0 (0–0)	0 (0–0)	--	0 (0–0)
Iceland	Measles	0 (0–0)	0 (0–0)	0 (0–0)	0 (0–0)
Ireland	All causes	168 (77·1–321)	83·8 (6·62–241)	55·3 (34·5–77·9)	47·7 (34·2–66·7)
Ireland	Diarrheal diseases	36·4 (-2·23–86·0)	10·5 (2·74–23·0)	19·9 (-2·89–51·9)	8·16 (-1·84–20·0)
Ireland	Lower respiratory infections	129 (76·4–243)	71·0 (1·51–216)	33·1 (17·5–51·4)	39·5 (23·3–61·4)
Ireland	Malaria	0 (0–0)	0 (0–0)	--	0 (0–0)
Ireland	Measles	0 (0–0)	0 (0–0)	0 (0–0)	0 (0–0)

Israel	All causes	718<br>(289–1 360)	284<br>(87·3–659)	345<br>(99·0–709)	177<br>(135–234)
Israel	Diarrheal diseases	378<br>(74·3–790)	86·8<br>(55·7–145)	245<br>(-14·9–637)	74·1<br>(27·5–131)
Israel	Lower respiratory infections	316<br>(189–577)	174<br>(3·88–515)	75·4<br>(40·4–116)	103<br>(63·8–156)
Israel	Malaria	0<br>(0–0)	0<br>(0–0)	--	0<br>(0–0)
Israel	Measles	0·109<br>(0·0328–0·237)	0·0288<br>(0·00856–0·0637)	0·0356<br>(0·00897–0·0898)	0·0491<br>(0·0159–0·115)
Italy	All causes	1 070<br>(573–1 950)	534<br>(109–1 420)	410<br>(250–617)	297<br>(213–415)
Italy	Diarrheal diseases	270<br>(33·9–575)	65·5<br>(36·0–116)	170<br>(-12·1–446)	54·0<br>(12·1–102)
Italy	Lower respiratory infections	743<br>(442–1 420)	408<br>(9·62–1 270)	180<br>(97·3–274)	243<br>(150–368)
Italy	Malaria	0<br>(0–0)	0<br>(0–0)	--	0<br>(0–0)
Italy	Measles	0·0425<br>(0·0117–0·0959)	0·0123<br>(0·00309–0·0302)	0·0143<br>(0·00361–0·0391)	0·0176<br>(0·00402–0·0416)
Luxembourg	All causes	14·8<br>(6·46–27·9)	6·71<br>(2·59–14·1)	7·41<br>(2·85–14·4)	3·35<br>(2·53–4·38)
Luxembourg	Diarrheal diseases	7·41<br>(1·07–16·0)	1·96<br>(1·08–3·46)	4·59<br>(-0·345–12·0)	1·46<br>(0·336–2·79)
Luxembourg	Lower respiratory infections	6<br>(3·54–10·9)	3·4<br>(0·088–9·84)	1·47<br>(0·785–2·27)	1·89<br>(1·16–2·82)
Luxembourg	Malaria	0<br>(0–0)	0<br>(0–0)	--	0<br>(0–0)
Luxembourg	Measles	0<br>(0–0)	0<br>(0–0)	0<br>(0–0)	0<br>(0–0)
Malta	All causes	15·2<br>(7·48–28·3)	8·13<br>(0·501–22·3)	4·44<br>(3·26–5·83)	4·59<br>(3·62–6·14)
Malta	Diarrheal diseases	2·69<br>(-0·31–6·66)	0·959<br>(0·147–2·24)	1·25<br>(-0·242–3·11)	0·662<br>(-0·235–1·84)
Malta	Lower respiratory infections	12·3<br>(7·51–21·6)	7·01<br>(0·177–19·7)	3·02<br>(1·7–4·49)	3·93<br>(2·34–5·8)
Malta	Malaria	0<br>(0–0)	0<br>(0–0)	--	0<br>(0–0)
Malta	Measles	0<br>(0–0)	0<br>(0–0)	0<br>(0–0)	0<br>(0–0)
Monaco	All causes	0·918<br>(0·382–1·85)	0·475<br>(0·0256–1·5)	0·261<br>(0·165–0·399)	0·278<br>(0·177–0·455)
Monaco	Diarrheal diseases	0·121<br>(-0·0244–0·33)	0·0394<br>(0·0015–0·0974)	0·0556<br>(-0·012–0·168)	0·0314<br>(-0·0148–0·0944)
Monaco	Lower respiratory infections	0·788<br>(0·386–1·63)	0·427<br>(0·0126–1·42)	0·196<br>(0·0904–0·349)	0·247<br>(0·117–0·45)
Monaco	Malaria	0<br>(0–0)	0<br>(0–0)	--	0<br>(0–0)
Monaco	Measles	0<br>(0–0)	0<br>(0–0)	0<br>(0–0)	0<br>(0–0)
Netherlands	All causes	436<br>(212–794)	278<br>(86·1–609)	151<br>(90·9–255)	92·6<br>(67·0–132)
Netherlands	Diarrheal diseases	106<br>(31·9–205)	36·7<br>(25·0–53·9)	58·5<br>(-2·88–157)	18·0<br>(8·72–26·3)
Netherlands	Lower respiratory infections	277<br>(133–552)	188<br>(5·11–508)	39·7<br>(19·5–64·8)	74·5<br>(49·8–111)
Netherlands	Malaria	0<br>(0–0)	0<br>(0–0)	--	0<br>(0–0)

Netherlands	Measles	0·224 (0·0805–0·398)	0·0774 (0·0287–0·142)	0·0674 (0·0205–0·156)	0·0865 (0·0305–0·163)
Norway	All causes	69·9 (30·8–134)	34·2 (8·34–83·7)	29·9 (15·3–52·2)	17·6 (12·2–23·9)
Norway	Diarrheal diseases	24·0 (0·698–59·1)	6·41 (1·95–13·7)	13·9 (-1·51–39·0)	4·97 (-0·233–11·8)
Norway	Lower respiratory infections	39·8 (23·5–71·9)	21·7 (0·474–64·3)	9·84 (4·98–15·3)	12·7 (7·95–19·8)
Norway	Malaria	0 (0–0)	0 (0–0)	--	0 (0–0)
Norway	Measles	0·00297 (<0.001–0·00645)	<0.001 (<0.001–0·002)	<0.001 (<0.001–0·00269)	0·0012 (<0.001–0·00269)
Portugal	All causes	313 (175–555)	165 (31·4–411)	107 (71·3–150)	91·8 (67·7–127)
Portugal	Diarrheal diseases	62·7 (9·19–131)	17·6 (9·18–30·2)	37·2 (-3·07–97·4)	13·3 (2·95–24·7)
Portugal	Lower respiratory infections	235 (142–414)	133 (3·38–367)	54·8 (28·7–82·2)	78·4 (52·6–117)
Portugal	Malaria	0 (0–0)	0 (0–0)	--	0 (0–0)
Portugal	Measles	0·513 (0·103–1·24)	0·168 (0·0334–0·414)	0·154 (0·0256–0·465)	0·216 (0·0384–0·482)
San Marino	All causes	0·491 (0·205–0·95)	0·257 (0·0149–0·744)	0·143 (0·0841–0·211)	0·149 (0·0949–0·249)
San Marino	Diarrheal diseases	0·0933 (-0·0229–0·257)	0·0338 (<0.001–0·0902)	0·0394 (-0·00948–0·115)	0·0247 (-0·0135–0·0773)
San Marino	Lower respiratory infections	0·391 (0·192–0·776)	0·215 (0·00606–0·666)	0·0964 (0·0452–0·177)	0·124 (0·0644–0·235)
San Marino	Malaria	0 (0–0)	0 (0–0)	--	0 (0–0)
San Marino	Measles	0 (0–0)	0 (0–0)	0 (0–0)	0 (0–0)
Spain	All causes	994 (443–1 880)	466 (90·1–1 160)	399 (186–724)	265 (196–356)
Spain	Diarrheal diseases	358 (40·1–774)	94·4 (43·6–176)	218 (-17·3–585)	72·9 (12·4–148)
Spain	Lower respiratory infections	603 (357–1 130)	339 (8·18–996)	148 (78·1–226)	192 (118–289)
Spain	Malaria	0 (0–0)	0 (0–0)	--	0 (0–0)
Spain	Measles	0·0015 (<0.001–0·00276)	<0.001 (<0.001–<0.001)	<0.001 (<0.001–0·0016)	<0.001 (<0.001–<0.001)
Sweden	All causes	261 (109–506)	110 (29·2–266)	122 (41·4–246)	63·9 (48·4–81·7)
Sweden	Diarrheal diseases	123 (16·8–270)	29·0 (15·5–50·3)	79·8 (-5·51–211)	23·2 (6·21–43·1)
Sweden	Lower respiratory infections	126 (74·3–236)	69·2 (1·44–209)	30·8 (16·3–46·6)	40·7 (28·4–59·4)
Sweden	Malaria	0 (0–0)	0 (0–0)	--	0 (0–0)
Sweden	Measles	0·00581 (0·0019–0·0105)	0·00164 (<0.001–0·00305)	0·00196 (<0.001–0·00456)	0·00243 (<0.001–0·00481)
Switzerland	All causes	180 (66·5–356)	80·3 (21·0–192)	82·2 (29·9–158)	44·2 (30·7–59·8)
Switzerland	Diarrheal diseases	85·2 (4·01–200)	23·1 (8·7–44·2)	50·3 (-5·15–134)	17·5 (0·48–37·6)
Switzerland	Lower respiratory infections	84·1 (50·4–159)	46·2 (1–141)	20·9 (10·8–32·5)	26·7 (17·7–40·4)

Switzerland	Malaria	0 (0–0)	0 (0–0)	--	0 (0–0)
Switzerland	Measles	0·00158 (>-0.001–0·00425)	<0.001 (<0.001–<0.001)	0·00127 (>-0.001–0·00375)	<0.001 (<0.001–<0.001)
United Kingdom	All causes	1 750 (874–3 350)	832 (94·5–2 320)	600 (402–895)	512 (391–662)
United Kingdom	Diarrheal diseases	383 (8·08–895)	95·5 (30·4–195)	228 (-18·5–651)	78·4 (-1·12–177)
United Kingdom	Lower respiratory infections	1 310 (805–2 440)	680 (15·4–2 080)	316 (167–467)	433 (313–612)
United Kingdom	Malaria	0 (0–0)	0 (0–0)	--	0 (0–0)
United Kingdom	Measles	0·642 (0·25–1·02)	0·168 (0·0672–0·273)	0·234 (0·0818–0·481)	0·268 (0·113–0·452)
**Latin America and Caribbean**	**All causes**	**1 470 000 (1 110 000–1 850 000)**	**920 000 (427 000–1 430 000)**	**624 000 (450 000–788 000)**	**566 000 (417 000–728 000)**
**Latin America and Caribbean**	**Diarrheal diseases**	**322 000 (202 000–460 000)**	**118 000 (67 800–188 000)**	**183 000 (-6 980–415 000)**	**115 000 (76 600–159 000)**
**Latin America and Caribbean**	**Lower respiratory infections**	**878 000 (641 000–1 180 000)**	**528 000 (22 700–1 070 000)**	**168 000 (97 600–242 000)**	**451 000 (318 000–578 000)**
**Latin America and Caribbean**	**Malaria**	**1 810 (-892–6 850)**	**1 320 (-665–4 290)**	**--**	**684 (-239–3 520)**
**Latin America and Caribbean**	**Measles**	**3·5 (1·53–5·11)**	**1·09 (0·494–1·64)**	**0·849 (0·322–1·76)**	**1·95 (0·797–2·86)**
Andean Latin America	All causes	257 000 (195 000–337 000)	165 000 (61 000–284 000)	93 000 (80 100–106 000)	103 000 (72 700–134 000)
Andean Latin America	Diarrheal diseases	22 900 (13 700–36 600)	7 300 (4 170–11 900)	11 900 (-421–30 900)	8 370 (5 160–11 900)
Andean Latin America	Lower respiratory infections	184 000 (132 000–254 000)	107 000 (3 720–229 000)	30 800 (18 500–43 500)	94 500 (64 600–123 000)
Andean Latin America	Malaria	38·7 (-15·5–133)	33·8 (-13·9–111)	--	5·99 (-2·28–36·6)
Andean Latin America	Measles	0·0276 (0·0101–0·0482)	0·00933 (0·00329–0·0168)	0·00608 (0·00186–0·015)	0·0164 (0·00559–0·0318)
Bolivia (Plurinational State of)	All causes	78 100 (52 400–109 000)	49 800 (14 900–93 800)	28 600 (21 300–35 700)	31 000 (18 600–46 300)
Bolivia (Plurinational State of)	Diarrheal diseases	5 990 (2 270–12 300)	1 790 (689–3 950)	3 430 (-121–9 900)	2 120 (875–4 260)
Bolivia (Plurinational State of)	Lower respiratory infections	57 600 (34 200–90 700)	33 500 (1 370–81 000)	10 700 (5 520–17 900)	28 900 (16 700–44 000)
Bolivia (Plurinational State of)	Malaria	11·9 (-4·66–44·3)	10·3 (-4·12–35·3)	--	1·96 (-0·752–11·2)
Bolivia (Plurinational State of)	Measles	0 (0–0)	0 (0–0)	0 (0–0)	0 (0–0)
Ecuador	All causes	76 700 (58 400–97 600)	49 400 (18 500–83 300)	33 400 (28 400–39 000)	27 500 (19 400–37 100)
Ecuador	Diarrheal diseases	6 950 (3 850–10 300)	2 100 (1 230–3 160)	4 450 (-159–9 440)	2 070 (1 420–2 790)
Ecuador	Lower respiratory infections	54 900 (39 600–74 500)	32 400 (1 230–67 700)	14 000 (8 410–19 600)	25 400 (17 300–34 600)
Ecuador	Malaria	2·53 (-0·987–9·13)	2·26 (-0·899–7·59)	--	0·328 (-0·132–1·97)
Ecuador	Measles	0·0276 (0·0101–0·0482)	0·00933 (0·00329–0·0168)	0·00608 (0·00186–0·015)	0·0164 (0·00559–0·0318)
Peru	All causes	103 000 (74 200–139 000)	65 500 (27 800–116 000)	31 000 (25 200–38 800)	44 400 (30 800–58 700)
Peru	Diarrheal diseases	9 990 (6 120–16 200)	3 400 (1 960–5 950)	4 070 (-141–12 600)	4 180 (2 690–6 450)

Peru	Lower respiratory infections	71 700<br>(47 500–107 000)	41 200<br>(1 120–92 700)	6 070<br>(2 990–9 370)	40 200<br>(27 000–53 400)
Peru	Malaria	24·3<br>(-10·1–80·0)	21·2<br>(-8·79–69·0)	--	3·7<br>(-1·38–22·6)
Peru	Measles	0<br>(0–0)	0<br>(0–0)	0<br>(0–0)	0<br>(0–0)
Caribbean	All causes	402 000<br>(268 000–528 000)	258 000<br>(134 000–363 000)	225 000<br>(132 000–310 000)	129 000<br>(84 700–178 000)
Caribbean	Diarrheal diseases	134 000<br>(66 400–225 000)	55 600<br>(26 500–102 000)	89 300<br>(-4 130–191 000)	39 200<br>(20 400–70 100)
Caribbean	Lower respiratory infections	189 000<br>(116 000–261 000)	124 000<br>(7 440–234 000)	58 600<br>(31 400–93 800)	89 100<br>(52 600–133 000)
Caribbean	Malaria	1 090<br>(-587–3 910)	798<br>(-419–2 870)	--	449<br>(-154–2 030)
Caribbean	Measles	0·0534<br>(0·0199–0·103)	0·0136<br>(0·00482–0·0279)	0·0257<br>(0·00761–0·0593)	0·0181<br>(0·00667–0·0379)
Antigua and Barbuda	All causes	65·6<br>(47·2–89·8)	42·6<br>(17·9–75·3)	34·0<br>(27·2–41·3)	16·8<br>(11·5–24·2)
Antigua and Barbuda	Diarrheal diseases	4·96<br>(1·75–8·16)	1·3<br>(0·858–1·91)	3·6<br>(-0·176–7·36)	0·932<br>(0·635–1·28)
Antigua and Barbuda	Lower respiratory infections	45·2<br>(30·7–64·9)	25·9<br>(0·914–58·2)	14·9<br>(8·24–22·0)	15·9<br>(10·7–23·2)
Antigua and Barbuda	Malaria	0<br>(0–0)	0<br>(0–0)	--	0<br>(0–0)
Antigua and Barbuda	Measles	0<br>(0–0)	0<br>(0–0)	0<br>(0–0)	0<br>(0–0)
Bahamas	All causes	183<br>(126–259)	117<br>(38·0–228)	80·8<br>(64·7–100)	53·6<br>(36·5–75·0)
Bahamas	Diarrheal diseases	15·5<br>(6·55–25·0)	4·44<br>(2·83–6·52)	10·6<br>(-0·422–22·4)	3·34<br>(2·26–4·65)
Bahamas	Lower respiratory infections	137<br>(91·0–208)	81·3<br>(2·66–193)	38·8<br>(22·2–58·0)	50·2<br>(33·4–71·4)
Bahamas	Malaria	0<br>(0–0)	0<br>(0–0)	--	0<br>(0–0)
Bahamas	Measles	0<br>(0–0)	0<br>(0–0)	0<br>(0–0)	0<br>(0–0)
Barbados	All causes	70·4<br>(47·4–101)	43·1<br>(16·0–83·6)	33·4<br>(27·6–40·4)	19·1<br>(12·5–27·8)
Barbados	Diarrheal diseases	8·15<br>(1·99–14·6)	2·16<br>(1·51–3·18)	5·53<br>(-0·311–12·7)	1·73<br>(0·879–2·47)
Barbados	Lower respiratory infections	49·5<br>(32·8–75·3)	28·2<br>(0·892–69·0)	15·1<br>(8·48–22·0)	17·4<br>(10·5–25·8)
Barbados	Malaria	0<br>(0–0)	0<br>(0–0)	--	0<br>(0–0)
Barbados	Measles	0<br>(0–0)	0<br>(0–0)	0<br>(0–0)	0<br>(0–0)
Belize	All causes	745<br>(550–956)	489<br>(217–775)	328<br>(267–391)	259<br>(169–361)
Belize	Diarrheal diseases	99·5<br>(54·7–153)	30·7<br>(17·4–47·4)	58·0<br>(-1·54–137)	32·8<br>(20·5–47·9)
Belize	Lower respiratory infections	464<br>(313–639)	276<br>(9·62–572)	88·7<br>(49·6–133)	226<br>(144–321)
Belize	Malaria	0<br>(0–0)	0<br>(0–0)	--	0<br>(0–0)
Belize	Measles	0<br>(0–0)	0<br>(0–0)	0<br>(0–0)	0<br>(0–0)
Bermuda	All causes	2·46<br>(1·42–3·99)	1·39<br>(0·328–3·03)	1·06<br>(0·814–1·33)	0·615<br>(0·452–0·847)

Bermuda	Diarrheal diseases	0·439 (-0·0235–1·01)	0·153 (0·0451–0·335)	0·237 (-0·0362–0·554)	0·0925 (-0·025–0·233)
Bermuda	Lower respiratory infections	1·76 (1·14–2·83)	0·992 (0·0283–2·46)	0·562 (0·311–0·869)	0·521 (0·316–0·805)
Bermuda	Malaria	0 (0–0)	0 (0–0)	--	0 (0–0)
Bermuda	Measles	0·00982 (-0·00364–0·0357)	<0.001 (<0.001–<0.001)	0·00903 (-0·00337–0·0314)	<0.001 (>-0.001–0·00386)
Cuba	All causes	3 010 (1 900–4 640)	1 610 (293–3 650)	913 (671–1 160)	1 170 (843–1 570)
Cuba	Diarrheal diseases	328 (109–602)	81·5 (57·2–124)	209 (-8·61–506)	79·2 (51·2–112)
Cuba	Lower respiratory infections	2 500 (1 590–3 890)	1 360 (41·3–3 380)	527 (274–813)	1 090 (777–1 480)
Cuba	Malaria	0 (0–0)	0 (0–0)	--	0 (0–0)
Cuba	Measles	0 (0–0)	0 (0–0)	0 (0–0)	0 (0–0)
Dominica	All causes	26·7 (15·5–40·1)	17·1 (7·28–32·9)	13·5 (9·45–17·8)	6·96 (4·14–11·1)
Dominica	Diarrheal diseases	4·09 (1·41–7·91)	1·14 (0·506–2·22)	2·83 (-0·101–7·09)	0·874 (0·366–1·56)
Dominica	Lower respiratory infections	16·7 (8·88–28·9)	10·0 (0·247–26·1)	4·72 (2·34–8·01)	6·09 (3·39–9·95)
Dominica	Malaria	0 (0–0)	0 (0–0)	--	0 (0–0)
Dominica	Measles	0 (0–0)	0 (0–0)	0 (0–0)	0 (0–0)
Dominican Republic	All causes	18 400 (12 600–26 500)	11 600 (5 980–19 600)	9 480 (6 830–13 600)	4 950 (2 660–7 730)
Dominican Republic	Diarrheal diseases	4 100 (1 760–7 220)	1 180 (613–2 010)	2 670 (-84·8–6 640)	1 020 (598–1 650)
Dominican Republic	Lower respiratory infections	9 940 (6 000–14 800)	6 010 (207–13 600)	2 420 (1 120–4 110)	3 930 (1 860–6 240)
Dominican Republic	Malaria	0·119 (-0·0469–0·492)	0·0972 (-0·0376–0·413)	--	0·0262 (-0·00795–0·146)
Dominican Republic	Measles	0 (0–0)	0 (0–0)	0 (0–0)	0 (0–0)
Grenada	All causes	94·1 (66·4–133)	60·5 (16·5–116)	36·5 (27·6–46·7)	31·3 (21·4–44·4)
Grenada	Diarrheal diseases	4·57 (1·76–7·3)	1·49 (1·07–2·12)	2·9 (-0·163–6·1)	1·07 (0·683–1·53)
Grenada	Lower respiratory infections	76·8 (51·4–112)	46·3 (1·71–101)	20·9 (11·8–31·2)	30·3 (20·2–43·3)
Grenada	Malaria	0 (0–0)	0 (0–0)	--	0 (0–0)
Grenada	Measles	0 (0–0)	0 (0–0)	0 (0–0)	0 (0–0)
Guyana	All causes	3 470 (2 520–4 480)	2 500 (1 460–3 570)	2 270 (1 750–2 760)	722 (501–987)
Guyana	Diarrheal diseases	640 (355–940)	229 (120–375)	462 (-20·2–893)	140 (77·6–221)
Guyana	Lower respiratory infections	1 540 (1 060–2 060)	988 (42·5–1 930)	560 (327–819)	570 (366–807)
Guyana	Malaria	47·5 (-25·9–182)	38·8 (-20·4–136)	--	12·0 (-4·1–74·3)
Guyana	Measles	0 (0–0)	0 (0–0)	0 (0–0)	0 (0–0)

Haiti	All causes	358 000 (238 000–475 000)	230 000 (122 000–322 000)	202 000 (116 000–283 000)	116 000 (76 800–163 000)
Haiti	Diarrheal diseases	124 000 (61 100–210 000)	51 900 (24 500–96 300)	82 100 (-3 850–178 000)	36 400 (18 700–65 700)
Haiti	Lower respiratory infections	166 000 (95 200–233 000)	110 000 (6 850–208 000)	52 300 (27 000–85 000)	79 500 (46 500–120 000)
Haiti	Malaria	1 010 (-540–3 620)	732 (-386–2 660)	--	423 (-145–1 890)
Haiti	Measles	0 (0–0)	0 (0–0)	0 (0–0)	0 (0–0)
Jamaica	All causes	1 220 (851–1 700)	764 (444–1 170)	698 (489–949)	292 (182–422)
Jamaica	Diarrheal diseases	304 (128–516)	79·6 (42·5–127)	203 (-6·79–469)	72·0 (44·4–103)
Jamaica	Lower respiratory infections	561 (358–849)	325 (8·23–761)	137 (75·0–209)	220 (132–336)
Jamaica	Malaria	0 (0–0)	0 (0–0)	--	0 (0–0)
Jamaica	Measles	0 (0–0)	0 (0–0)	0 (0–0)	0 (0–0)
Puerto Rico	All causes	376 (242–554)	226 (113–398)	212 (158–269)	79·8 (57·6–110)
Puerto Rico	Diarrheal diseases	79·2 (20·8–149)	17·8 (12·4–25·9)	57·4 (-2·45–132)	13·5 (7·82–19·0)
Puerto Rico	Lower respiratory infections	200 (127–328)	112 (3·04–289)	58·6 (32·8–87·5)	66·3 (46·9–95·3)
Puerto Rico	Malaria	0 (0–0)	0 (0–0)	--	0 (0–0)
Puerto Rico	Measles	0·0418 (0·0143–0·0888)	0·0131 (0·00465–0·0269)	0·0159 (0·00478–0·0388)	0·0166 (0·00595–0·0366)
Saint Kitts and Nevis	All causes	36·4 (24·9–48·9)	24·4 (13·6–38·0)	21·5 (16·6–25·5)	7·73 (5·09–10·8)
Saint Kitts and Nevis	Diarrheal diseases	6·45 (2·74–10·6)	1·78 (1·04–2·7)	4·51 (-0·161–9·84)	1·34 (0·864–1·89)
Saint Kitts and Nevis	Lower respiratory infections	18·1 (11·5–26·3)	10·9 (0·376–24·1)	5·16 (2·97–7·54)	6·39 (4·04–9·06)
Saint Kitts and Nevis	Malaria	0 (0–0)	0 (0–0)	--	0 (0–0)
Saint Kitts and Nevis	Measles	0 (0–0)	0 (0–0)	0 (0–0)	0 (0–0)
Saint Lucia	All causes	91·4 (63·7–130)	62·6 (33·1–103)	54·4 (42·8–67·4)	15·5 (10·8–22·2)
Saint Lucia	Diarrheal diseases	15·2 (5·49–26·5)	4·24 (2·69–6·28)	11·2 (-0·408–24·6)	2·21 (1·38–3·2)
Saint Lucia	Lower respiratory infections	47·8 (30·1–74·0)	30·0 (1·14–67·5)	14·8 (8·37–22·7)	13·3 (9·03–19·2)
Saint Lucia	Malaria	0 (0–0)	0 (0–0)	--	0 (0–0)
Saint Lucia	Measles	0 (0–0)	0 (0–0)	0 (0–0)	0 (0–0)
Saint Vincent and the Grenadines	All causes	130 (94·1–173)	93·3 (51·2–148)	75·6 (61·9–93·5)	29·2 (19·0–41·1)
Saint Vincent and the Grenadines	Diarrheal diseases	13·2 (5·83–22·2)	3·73 (2·14–5·63)	9·14 (-0·274–20·6)	2·85 (1·83–4·15)
Saint Vincent and the Grenadines	Lower respiratory infections	69·4 (44·2–102)	42·0 (1·47–91·1)	18·9 (9·86–29·1)	26·3 (16·2–38·3)
Saint Vincent and the Grenadines	Malaria	0 (0–0)	0 (0–0)	--	0 (0–0)

Saint Vincent and the Grenadines	Measles	0<br>(0–0)	0<br>(0–0)	0<br>(0–0)	0<br>(0–0)
Suriname	All causes	1 710<br>(1 000–2 360)	1 040<br>(508–1 570)	996<br>(562–1 480)	384<br>(243–557)
Suriname	Diarrheal diseases	563<br>(240–969)	187<br>(88·8–352)	408<br>(-14·6–927)	106<br>(52·7–187)
Suriname	Lower respiratory infections	831<br>(503–1 250)	541<br>(18·6–1 100)	276<br>(147–433)	278<br>(155–410)
Suriname	Malaria	0·0952<br>(-0·053–0·386)	0·0952<br>(-0·053–0·386)	--	<0.001<br>(>-0.001–<0.001)
Suriname	Measles	0<br>(0–0)	0<br>(0–0)	0<br>(0–0)	0<br>(0–0)
Trinidad and Tobago	All causes	587<br>(430–766)	391<br>(204–619)	317<br>(252–382)	158<br>(106–227)
Trinidad and Tobago	Diarrheal diseases	105<br>(45·7–162)	33·8<br>(20·2–52·0)	70·4<br>(-2·67–147)	26·0<br>(16·0–37·2)
Trinidad and Tobago	Lower respiratory infections	328<br>(220–462)	203<br>(8·44–428)	92·8<br>(53·9–136)	132<br>(80·1–195)
Trinidad and Tobago	Malaria	0<br>(0–0)	0<br>(0–0)	--	0<br>(0–0)
Trinidad and Tobago	Measles	0<br>(0–0)	0<br>(0–0)	0<br>(0–0)	0<br>(0–0)
United States Virgin Islands	All causes	19·1<br>(12·7–26·9)	12·7<br>(8·36–17·9)	13·4<br>(8·89–18·7)	3·12<br>(2·13–4·41)
United States Virgin Islands	Diarrheal diseases	5·16<br>(1·78–9·23)	1·25<br>(0·771–1·87)	3·68<br>(-0·135–8·39)	0·997<br>(0·661–1·39)
United States Virgin Islands	Lower respiratory infections	5·93<br>(3·59–9·56)	3·4<br>(0·0946–8·53)	1·68<br>(0·894–2·64)	2·12<br>(1·39–3·15)
United States Virgin Islands	Malaria	0<br>(0–0)	0<br>(0–0)	--	0<br>(0–0)
United States Virgin Islands	Measles	0<br>(0–0)	0<br>(0–0)	0<br>(0–0)	0<br>(0–0)
Central Latin America	All causes	633 000<br>(492 000–788 000)	392 000<br>(182 000–619 000)	237 000<br>(179 000–313 000)	269 000<br>(192 000–345 000)
Central Latin America	Diarrheal diseases	138 000<br>(96 900–185 000)	48 600<br>(27 700–71 300)	65 400<br>(-1 930–161 000)	59 500<br>(41 500–75 500)
Central Latin America	Lower respiratory infections	375 000<br>(280 000–503 000)	224 000<br>(9 170–459 000)	52 800<br>(30 800–73 900)	209 000<br>(138 000–272 000)
Central Latin America	Malaria	534<br>(-235–2 150)	380<br>(-173–1 280)	--	190<br>(-68·7–1 070)
Central Latin America	Measles	0·0672<br>(0·0219–0·135)	0·0133<br>(0·00382–0·0289)	0·0243<br>(0·0021–0·0575)	0·0357<br>(0·00932–0·0756)
Colombia	All causes	78 300<br>(60 700–100 000)	57 800<br>(37 000–83 700)	47 200<br>(41 100–54 600)	17 000<br>(12 700–23 000)
Colombia	Diarrheal diseases	8 440<br>(4 050–14 500)	2 340<br>(1 470–3 430)	5 220<br>(-171–13 000)	2 200<br>(1 560–2 920)
Colombia	Lower respiratory infections	34 700<br>(22 900–52 900)	20 300<br>(650–48 000)	6 960<br>(4 010–9 900)	14 700<br>(10 600–20 300)
Colombia	Malaria	218<br>(-89·1–766)	163<br>(-66·9–534)	--	63·6<br>(-21·5–310)
Colombia	Measles	0<br>(0–0)	0<br>(0–0)	0<br>(0–0)	0<br>(0–0)
Costa Rica	All causes	786<br>(482–1 510)	380<br>(68·4–1 120)	303<br>(228–384)	218<br>(159–298)
Costa Rica	Diarrheal diseases	108<br>(21·1–249)	18·9<br>(12·9–29·0)	78·0<br>(-3·31–223)	16·6<br>(9·2–24·9)
Costa Rica	Lower respiratory infections	631<br>(397–1 220)	313<br>(6·71–1 050)	176<br>(95·9–264)	202<br>(143–282)

Costa Rica	Malaria	0·136 (-0·0459–0·579)	0·136 (-0·0459–0·579)	--	<0.001 (>-0.001–<0.001)
Costa Rica	Measles	0 (0–0)	0 (0–0)	0 (0–0)	0 (0–0)
El Salvador	All causes	9 130 (6 510–12 700)	5 670 (2 380–9 900)	3 450 (2 550–4 600)	3 420 (2 330–4 760)
El Salvador	Diarrheal diseases	1 640 (915–2 580)	537 (288–908)	929 (-28·1–2 220)	525 (313–782)
El Salvador	Lower respiratory infections	6 080 (4 040–8 880)	3 710 (124–8 000)	1 110 (614–1 680)	2 900 (1 840–4 060)
El Salvador	Malaria	0 (0–0)	0 (0–0)	--	0 (0–0)
El Salvador	Measles	0 (0–0)	0 (0–0)	0 (0–0)	0 (0–0)
Guatemala	All causes	181 000 (145 000–218 000)	111 000 (44 400–167 000)	46 700 (30 900–72 100)	97 400 (68 800–129 000)
Guatemala	Diarrheal diseases	47 400 (37 200–58 100)	20 200 (11 900–30 400)	18 000 (-486–48 100)	24 500 (17 500–32 400)
Guatemala	Lower respiratory infections	117 000 (89 600–147 000)	74 600 (3 640–133 000)	12 200 (6 570–18 400)	72 900 (47 500–97 300)
Guatemala	Malaria	4·86 (-3·19–18·5)	4·83 (-3·18–18·3)	--	0·0333 (-0·0147–0·177)
Guatemala	Measles	0 (0–0)	0 (0–0)	0 (0–0)	0 (0–0)
Honduras	All causes	16 100 (10 700–23 400)	9 440 (4 470–15 800)	6 460 (3 540–10 700)	6 280 (4 300–9 030)
Honduras	Diarrheal diseases	5 570 (2 790–9 840)	2 030 (929–3 760)	2 940 (-77·2–7 790)	1 950 (940–3 480)
Honduras	Lower respiratory infections	8 250 (4 850–13 200)	5 180 (232–11 800)	1 290 (638–2 120)	4 320 (2 750–6 490)
Honduras	Malaria	7·83 (-4·00–22·9)	7·51 (-3·82–21·8)	--	0·446 (-0·161–2·07)
Honduras	Measles	0 (0–0)	0 (0–0)	0 (0–0)	0 (0–0)
Mexico	All causes	243 000 (188 000–308 000)	144 000 (58 100–245 000)	81 100 (62 800–107 000)	109 000 (76 200–144 000)
Mexico	Diarrheal diseases	47 600 (33 800–66 100)	16 000 (8 960–23 800)	22 300 (-594–57 300)	20 800 (14 000–27 200)
Mexico	Lower respiratory infections	158 000 (117 000–217 000)	90 500 (3 500–194 000)	21 300 (11 900–31 200)	88 400 (57 700–118 000)
Mexico	Malaria	0·966 (-0·757–3·17)	0·966 (-0·757–3·17)	--	<0.001 (>-0.001–<0.001)
Mexico	Measles	0 (0–0)	0 (0–0)	0 (0–0)	0 (0–0)
Nicaragua	All causes	16 700 (11 600–22 900)	10 700 (5 350–17 500)	8 250 (6 060–10 900)	4 770 (2 850–6 840)
Nicaragua	Diarrheal diseases	3 270 (1 590–5 540)	938 (484–1 590)	2 020 (-57·4–5 020)	922 (567–1 380)
Nicaragua	Lower respiratory infections	9 110 (5 880–13 300)	5 460 (177–12 000)	1 940 (1 090–3 060)	3 850 (2 130–5 630)
Nicaragua	Malaria	19·7 (-9·12–76·5)	14·8 (-6·92–57·0)	--	6·06 (-2·55–34·7)
Nicaragua	Measles	0 (0–0)	0 (0–0)	0 (0–0)	0 (0–0)
Panama	All causes	10 300 (7 670–13 900)	6 410 (2 700–11 500)	3 840 (3 130–4 920)	3 910 (2 680–5 280)
Panama	Diarrheal diseases	1 590 (858–2 540)	473 (299–707)	818 (-22·4–2 170)	572 (396–775)

Panama	Lower respiratory infections	6 640 (4 560–9 580)	3 860 (124–8 890)	944 (514–1 410)	3 340 (2 190–4 610)
Panama	Malaria	1·23 (-0·64–5·31)	1·22 (-0·636–5·25)	--	0·0118 (-0·00404–0·0672)
Panama	Measles	0 (0–0)	0 (0–0)	0 (0–0)	0 (0–0)
Venezuela (Bolivarian Republic of)	All causes	77 800 (56 200–103 000)	46 200 (28 000–68 300)	39 800 (26 600–56 600)	26 500 (17 800–35 400)
Venezuela (Bolivarian Republic of)	Diarrheal diseases	22 300 (11 000–37 100)	6 140 (2 890–10 800)	13 100 (-440–32 300)	7 990 (4 600–12 400)
Venezuela (Bolivarian Republic of)	Lower respiratory infections	35 400 (24 100–47 400)	20 000 (720–42 600)	6 830 (3 960–9 970)	18 400 (11 600–25 500)
Venezuela (Bolivarian Republic of)	Malaria	282 (-136–1 180)	187 (-99·5–825)	--	120 (-40·9–635)
Venezuela (Bolivarian Republic of)	Measles	0·0672 (0·0219–0·135)	0·0133 (0·00382–0·0289)	0·0243 (0·0021–0·0575)	0·0357 (0·00932–0·0756)
Tropical Latin America	All causes	182 000 (128 000–253 000)	106 000 (35 900–200 000)	68 900 (55 300–82 200)	65 400 (47 000–88 000)
Tropical Latin America	Diarrheal diseases	26 500 (13 300–42 700)	6 870 (4 030–10 000)	16 600 (-496–39 000)	7 550 (5 000–10 100)
Tropical Latin America	Lower respiratory infections	129 000 (89 300–188 000)	72 700 (2 330–167 000)	26 300 (15 000–38 300)	57 800 (41 400–77 900)
Tropical Latin America	Malaria	138 (-56·1–521)	106 (-43·8–344)	--	38·5 (-12·9–196)
Tropical Latin America	Measles	3·36 (1·45–4·91)	1·05 (0·475–1·59)	0·793 (0·283–1·66)	1·88 (0·762–2·78)
Brazil	All causes	174 000 (122 000–242 000)	100 000 (33 000–192 000)	65 400 (52 100–78 100)	63 100 (45 300–84 900)
Brazil	Diarrheal diseases	25 400 (12 700–41 000)	6 550 (3 840–9 510)	16 000 (-482–37 600)	7 200 (4 790–9 550)
Brazil	Lower respiratory infections	125 000 (86 100–181 000)	70 200 (2 260–162 000)	25 700 (14 700–37 500)	55 900 (39 800–75 300)
Brazil	Malaria	138 (-56·1–521)	106 (-43·8–344)	--	38·5 (-12·9–196)
Brazil	Measles	3·36 (1·45–4·91)	1·05 (0·475–1·59)	0·793 (0·283–1·66)	1·88 (0·762–2·78)
Paraguay	All causes	7 900 (5 810–11 100)	5 250 (2 870–9 030)	3 580 (2 830–4 470)	2 320 (1 470–3 360)
Paraguay	Diarrheal diseases	1 100 (548–1 850)	321 (158–535)	587 (-13·6–1 620)	343 (191–547)
Paraguay	Lower respiratory infections	4 380 (2 750–6 950)	2 510 (74·2–5 970)	581 (316–863)	1 980 (1 260–2 900)
Paraguay	Malaria	0 (0–0)	0 (0–0)	--	0 (0–0)
Paraguay	Measles	0 (0–0)	0 (0–0)	0 (0–0)	0 (0–0)
**North Africa and Middle East**	**All causes**	**2 800 000 (2 020 000–3 590 000)**	**1 790 000 (884 000–2 690 000)**	**1 490 000 (1 070 000–1 920 000)**	**1 120 000 (816 000–1 470 000)**
**North Africa and Middle East**	**Diarrheal diseases**	**582 000 (284 000–1 010 000)**	**254 000 (128 000–458 000)**	**403 000 (-20 700–873 000)**	**203 000 (107 000–345 000)**
**North Africa and Middle East**	**Lower respiratory infections**	**1 480 000 (1 010 000–2 050 000)**	**947 000 (61 500–1 830 000)**	**565 000 (328 000–848 000)**	**749 000 (486 000–1 060 000)**
**North Africa and Middle East**	**Malaria**	**43 800 (-33 200–126 000)**	**34 500 (-23 800–98 100)**	**--**	**17 600 (-7 240–72 500)**
**North Africa and Middle East**	**Measles**	**282 000 (99 700–585 000)**	**138 000 (50 100–288 000)**	**103 000 (33 100–217 000)**	**154 000 (50 100–321 000)**
North Africa and Middle East	All causes	2 800 000 (2 020 000–3 590 000)	1 790 000 (884 000–2 690 000)	1 490 000 (1 070 000–1 920 000)	1 120 000 (816 000–1 470 000)

North Africa and Middle East	Diarrheal diseases	582 000 (284 000–1 010 000)	254 000 (128 000–458 000)	403 000 (-20 700–873 000)	203 000 (107 000–345 000)
North Africa and Middle East	Lower respiratory infections	1 480 000 (1 010 000–2 050 000)	947 000 (61 500–1 830 000)	565 000 (328 000–848 000)	749 000 (486 000–1 060 000)
North Africa and Middle East	Malaria	43 800 (-33 200–126 000)	34 500 (-23 800–98 100)	--	17 600 (-7 240–72 500)
North Africa and Middle East	Measles	282 000 (99 700–585 000)	138 000 (50 100–288 000)	103 000 (33 100–217 000)	154 000 (50 100–321 000)
Afghanistan	All causes	1 220 000 (868 000–1 530 000)	835 000 (493 000–1 120 000)	706 000 (468 000–954 000)	477 000 (319 000–646 000)
Afghanistan	Diarrheal diseases	300 000 (138 000–536 000)	152 000 (66 800–279 000)	203 000 (-9 650–448 000)	115 000 (51 500–204 000)
Afghanistan	Lower respiratory infections	484 000 (309 000–688 000)	336 000 (23 200–616 000)	186 000 (103 000–298 000)	263 000 (151 000–408 000)
Afghanistan	Malaria	814 (-609–3 350)	620 (-438–2 240)	--	325 (-176–1 710)
Afghanistan	Measles	179 000 (64 800–355 000)	91 800 (32 500–193 000)	63 300 (19 200–138 000)	99 000 (30 500–207 000)
Algeria	All causes	66 500 (43 900–95 600)	33 000 (12 500–62 700)	30 000 (20 300–41 700)	25 400 (15 900–36 800)
Algeria	Diarrheal diseases	10 400 (3 030–22 700)	2 890 (1 450–5 410)	7 320 (-317–19 900)	2 630 (1 210–4 970)
Algeria	Lower respiratory infections	37 300 (22 600–59 900)	21 100 (842–50 600)	12 900 (6 570–22 700)	15 600 (8 370–26 600)
Algeria	Malaria	0 (0–0)	0 (0–0)	--	0 (0–0)
Algeria	Measles	14 100 (4 650–28 600)	4 190 (1 380–8 980)	4 930 (1 350–10 800)	7 180 (2 390–14 700)
Bahrain	All causes	277 (154–407)	153 (43·4–294)	137 (90·2–181)	78·6 (55·1–114)
Bahrain	Diarrheal diseases	56·3 (9·86–107)	15·8 (9·14–26·6)	38·6 (-2·85–87·3)	11·2 (3·92–19·1)
Bahrain	Lower respiratory infections	187 (109–290)	104 (4·01–243)	65·0 (34·4–110)	67·3 (43·6–102)
Bahrain	Malaria	0 (0–0)	0 (0–0)	--	0 (0–0)
Bahrain	Measles	0·0405 (0–0·098)	0·0125 (0–0·0309)	0·017 (0–0·0451)	0·0163 (0–0·0394)
Egypt	All causes	501 000 (339 000–707 000)	255 000 (61 700–518 000)	234 000 (161 000–316 000)	210 000 (149 000–287 000)
Egypt	Diarrheal diseases	104 000 (43 000–182 000)	27 800 (14 300–49 900)	75 500 (-3 230–164 000)	31 800 (17 800–51 500)
Egypt	Lower respiratory infections	367 000 (259 000–530 000)	201 000 (7 750–468 000)	132 000 (76 800–192 000)	176 000 (120 000–247 000)
Egypt	Malaria	0 (0–0)	0 (0–0)	--	0 (0–0)
Egypt	Measles	4 830 (1 510–10 400)	1 290 (389–3 090)	1 780 (434–4 160)	2 690 (839–5 910)
Iran (Islamic Republic of)	All causes	26 300 (16 600–39 300)	15 500 (5 050–29 500)	13 000 (9 350–16 400)	7 180 (4 640–11 000)
Iran (Islamic Republic of)	Diarrheal diseases	5 140 (1 700–8 890)	1 500 (925–2 440)	3 660 (-183–8 010)	1 050 (657–1 620)
Iran (Islamic Republic of)	Lower respiratory infections	17 600 (10 700–27 600)	10 400 (424–24 300)	5 700 (3 150–9 180)	6 130 (3 800–9 540)
Iran (Islamic Republic of)	Malaria	3·92 (-1·89–15·8)	3·92 (-1·89–15·8)	--	<0.001 (>-0.001–0·0014)
Iran (Islamic Republic of)	Measles	3·59 (1·81–5·25)	1·33 (0·661–2)	1·38 (0·557–2·61)	1·58 (0·705–2·42)

Iraq	All causes	114 000 (75 900–158 000)	60 000 (24 100–107 000)	55 600 (38 500–78 300)	44 800 (30 100–63 900)
Iraq	Diarrheal diseases	24 600 (10 100–47 500)	7 590 (3 600–14 100)	17 300 (-729–42 700)	7 270 (3 520–13 300)
Iraq	Lower respiratory infections	64 800 (41 600–100 000)	37 200 (1 700–85 800)	22 500 (11 300–38 300)	30 500 (17 700–49 800)
Iraq	Malaria	0 (0–0)	0 (0–0)	--	0 (0–0)
Iraq	Measles	13 100 (4 290–26 400)	4 000 (1 300–8 930)	4 640 (1 260–10 400)	6 970 (2 380–14 200)
Jordan	All causes	12 400 (7 410–20 600)	6 520 (832–16 100)	3 960 (2 830–5 650)	4 900 (3 080–7 550)
Jordan	Diarrheal diseases	1 240 (339–2 350)	311 (210–504)	796 (-35·1–1 910)	317 (169–480)
Jordan	Lower respiratory infections	10 700 (6 430–18 000)	5 770 (172–15 400)	2 730 (1 360–4 740)	4 580 (2 750–7 250)
Jordan	Malaria	0 (0–0)	0 (0–0)	--	0 (0–0)
Jordan	Measles	2·44 (1·03–3·45)	0·706 (0·307–1·08)	0·664 (0·234–1·27)	1·31 (0·539–1·97)
Kuwait	All causes	1 760 (1 100–2 740)	996 (51·5–2 390)	455 (310–616)	638 (479–874)
Kuwait	Diarrheal diseases	76·1 (7·65–151)	24·6 (11·5–41·7)	42·6 (-4·41–98·1)	17·8 (1·72–33·6)
Kuwait	Lower respiratory infections	1 680 (1 070–2 610)	962 (28·4–2 340)	403 (227–580)	620 (448–860)
Kuwait	Malaria	0 (0–0)	0 (0–0)	--	0 (0–0)
Kuwait	Measles	0·398 (-0·165–1·47)	<0.001 (<0.001–<0.001)	0·369 (-0·154–1·35)	0·0303 (-0·0146–0·148)
Lebanon	All causes	10 100 (6 850–14 000)	4 430 (978–9 010)	3 840 (2 680–5 290)	4 710 (3 140–6 640)
Lebanon	Diarrheal diseases	2 030 (654–3 720)	458 (239–837)	1 370 (-44·1–3 430)	653 (346–1 130)
Lebanon	Lower respiratory infections	7 030 (4 630–9 780)	3 550 (136–8 190)	1 970 (1 060–3 130)	3 600 (2 120–5 310)
Lebanon	Malaria	0 (0–0)	0 (0–0)	--	0 (0–0)
Lebanon	Measles	807 (289–1 560)	188 (64·2–386)	271 (71·2–637)	465 (155–990)
Libya	All causes	9 610 (6 470–14 200)	5 470 (1 270–11 100)	4 020 (2 820–5 630)	4 370 (2 910–6 560)
Libya	Diarrheal diseases	773 (209–1 770)	226 (97·5–449)	514 (-35·4–1 470)	250 (90·4–496)
Libya	Lower respiratory infections	7 470 (4 580–11 700)	4 080 (165–9 510)	2 330 (1 150–4 210)	3 960 (2 500–6 230)
Libya	Malaria	0 (0–0)	0 (0–0)	--	0 (0–0)
Libya	Measles	285 (89·9–668)	79·5 (24·9–200)	98·2 (24·0–241)	162 (50·7–389)
Morocco	All causes	55 100 (34 600–84 300)	23 900 (7 630–50 200)	22 700 (11 800–41 100)	22 700 (15 100–33 000)
Morocco	Diarrheal diseases	16 500 (5 500–34 400)	3 870 (1 570–7 500)	11 300 (-452–32 600)	4 720 (2 160–8 760)
Morocco	Lower respiratory infections	31 400 (18 900–51 900)	16 900 (635–44 600)	8 260 (3 820–14 200)	14 700 (8 570–23 000)
Morocco	Malaria	0 (0–0)	0 (0–0)	--	0 (0–0)

Morocco	Measles	5 560 (1 750–11 700)	1 440 (440–3 210)	1 580 (387–3 870)	3 260 (1 020–7 140)
Oman	All causes	3 320 (2 010–4 840)	2 180 (735–3 880)	1 790 (1 260–2 370)	887 (575–1 390)
Oman	Diarrheal diseases	613 (189–1 190)	235 (122–458)	432 (-33·8–1 040)	122 (57·9–226)
Oman	Lower respiratory infections	2 180 (1 270–3 390)	1 420 (76·9–3 020)	839 (474–1 410)	765 (466–1 280)
Oman	Malaria	0·721 (-0·354–4·05)	0·721 (-0·354–4·05)	--	<0.001 (>-0.001–<0.001)
Oman	Measles	1·12 (0·492–1·83)	0·481 (0·205–0·755)	0·473 (0·171–0·888)	0·412 (0·164–0·679)
Palestine	All causes	5 020 (3 410–7 590)	2 360 (502–5 390)	1 480 (1 130–1 930)	2 210 (1 580–3 060)
Palestine	Diarrheal diseases	544 (142–1 080)	128 (85·0–199)	343 (-14–894)	149 (72·6–230)
Palestine	Lower respiratory infections	3 730 (2 490–5 950)	1 920 (60·7–5 020)	815 (420–1 320)	1 740 (1 140–2 570)
Palestine	Malaria	0 (0–0)	0 (0–0)	--	0 (0–0)
Palestine	Measles	572 (177–1 180)	145 (44·0–321)	150 (37·5–356)	326 (101–707)
Qatar	All causes	471 (268–788)	227 (35·5–547)	173 (122–231)	160 (115–241)
Qatar	Diarrheal diseases	62·5 (10·3–130)	14·7 (8·07–25·9)	42·2 (-2·99–105)	12·6 (3·87–22·3)
Qatar	Lower respiratory infections	378 (223–602)	196 (6·84–513)	113 (61·0–189)	139 (91·1–223)
Qatar	Malaria	0 (0–0)	0 (0–0)	--	0 (0–0)
Qatar	Measles	18·5 (5·32–41·5)	4·84 (1·41–10·9)	6·76 (1·68–16·5)	8·41 (2·57–19·2)
Saudi Arabia	All causes	40 800 (28 100–55 300)	20 600 (5 500–41 400)	22 200 (16 900–28 400)	11 800 (8 010–17 100)
Saudi Arabia	Diarrheal diseases	6 270 (1 390–10 800)	1 350 (829–2 330)	4 990 (-261–10 100)	1 060 (549–1 800)
Saudi Arabia	Lower respiratory infections	31 100 (22 700–42 700)	16 000 (615–36 900)	13 900 (8 260–21 100)	10 700 (7 150–15 600)
Saudi Arabia	Malaria	0·92 (-0·452–3·11)	0·914 (-0·446–3·07)	--	0·00665 (-0·00265–0·0397)
Saudi Arabia	Measles	173 (47·1–401)	47·8 (13·1–115)	90·6 (20·3–225)	61·2 (17·1–146)
Sudan	All causes	251 000 (128 000–358 000)	176 000 (49 600–280 000)	130 000 (88 900–176 000)	92 100 (59 000–125 000)
Sudan	Diarrheal diseases	41 400 (15 900–81 000)	20 000 (9 300–37 700)	30 200 (-2 630–68 500)	11 600 (5 390–22 300)
Sudan	Lower respiratory infections	128 000 (77 400–190 000)	89 600 (7 160–168 000)	57 400 (30 100–93 900)	59 700 (37 200–92 800)
Sudan	Malaria	28 400 (-18 500–84 100)	22 300 (-12 700–62 900)	--	11 000 (-5 050–47 500)
Sudan	Measles	20 900 (6 830–46 000)	11 200 (3 570–24 800)	9 500 (2 650–22 100)	9 690 (3 070–20 600)
Syrian Arab Republic	All causes	22 300 (17 100–27 900)	13 900 (8 040–20 500)	12 200 (9 370–15 500)	9 370 (6 590–12 400)
Syrian Arab Republic	Diarrheal diseases	1 710 (568–3 140)	613 (358–1 150)	1 140 (-71·2–2 480)	617 (275–1 040)
Syrian Arab Republic	Lower respiratory infections	11 100 (7 400–15 300)	6 430 (278–12 400)	3 930 (1 920–6 420)	6 510 (4 350–9 480)

Syrian Arab Republic	Malaria	0<br>(0–0)	0<br>(0–0)	--	0<br>(0–0)
Syrian Arab Republic	Measles	3 970<br>(1 410–7 860)	1 340<br>(441–2 900)	1 650<br>(464–3 460)	2 240<br>(724–4 550)
Tunisia	All causes	16 800<br>(10 700–24 300)	7 920<br>(2 940–15 200)	7 760<br>(4 540–11 700)	6 090<br>(4 070–9 010)
Tunisia	Diarrheal diseases	4 720<br>(1 450–9 080)	1 080<br>(520–2 110)	3 300<br>(-88·7–8 170)	1 250<br>(676–2 250)
Tunisia	Lower respiratory infections	9 650<br>(6 000–15 200)	5 070<br>(184–13 000)	2 640<br>(1 360–4 360)	4 330<br>(2 600–6 810)
Tunisia	Malaria	0<br>(0–0)	0<br>(0–0)	--	0<br>(0–0)
Tunisia	Measles	878<br>(284–1 860)	198<br>(61·4–446)	257<br>(64·5–623)	507<br>(164–1 120)
Türkiye	All causes	30 700<br>(20 100–45 800)	15 500<br>(6 060–30 700)	13 300<br>(9 590–18 700)	10 600<br>(7 340–15 100)
Türkiye	Diarrheal diseases	5 470<br>(1 510–11 200)	1 140<br>(697–1 910)	3 640<br>(-119–9 690)	1 340<br>(766–2 080)
Türkiye	Lower respiratory infections	18 200<br>(11 200–29 300)	9 060<br>(252–24 500)	4 450<br>(2 180–7 610)	7 830<br>(5 010–12 600)
Türkiye	Malaria	0<br>(0–0)	0<br>(0–0)	--	0<br>(0–0)
Türkiye	Measles	2 380<br>(725–4 970)	607<br>(183–1 360)	527<br>(123–1 340)	1 410<br>(436–2 960)
United Arab Emirates	All causes	3 260<br>(2 210–4 430)	1 960<br>(735–3 350)	1 820<br>(1 370–2 300)	1 010<br>(691–1 450)
United Arab Emirates	Diarrheal diseases	452<br>(117–855)	155<br>(89·6–280)	326<br>(-23·2–740)	98·3<br>(44·2–178)
United Arab Emirates	Lower respiratory infections	2 090<br>(1 290–2 920)	1 250<br>(60·3–2 650)	901<br>(514–1 440)	813<br>(516–1 240)
United Arab Emirates	Malaria	0<br>(0–0)	0<br>(0–0)	--	0<br>(0–0)
United Arab Emirates	Measles	270<br>(85·5–552)	99·6<br>(29·9–214)	142<br>(37·4–313)	95·1<br>(29·3–202)
Yemen	All causes	409 000<br>(270 000–535 000)	308 000<br>(112 000–444 000)	221 000<br>(157 000–292 000)	186 000<br>(126 000–250 000)
Yemen	Diarrheal diseases	55 300<br>(27 000–107 000)	32 600<br>(15 000–59 800)	38 300<br>(-2 670–94 100)	22 200<br>(10 200–40 900)
Yemen	Lower respiratory infections	239 000<br>(149 000–351 000)	179 000<br>(17 700–309 000)	105 000<br>(58 500–160 000)	138 000<br>(81 800–202 000)
Yemen	Malaria	14 500<br>(-13 500–59 100)	11 500<br>(-9 260–46 400)	--	6 170<br>(-2 930–26 500)
Yemen	Measles	35 700<br>(12 200–70 200)	21 400<br>(7 530–44 700)	13 700<br>(4 150–30 700)	19 800<br>(6 430–41 300)
**South Asia**	**All causes**	**15 400 000<br>(12 500 000–18 100 000)**	**10 800 000<br>(5 050 000–14 600 000)**	**9 500 000<br>(7 040 000–11 500 000)**	**6 210 000<br>(4 460 000–8 120 000)**
**South Asia**	**Diarrheal diseases**	**4 020 000<br>(2 660 000–5 630 000)**	**2 180 000<br>(1 400 000–3 290 000)**	**2 930 000<br>(-263 000–5 150 000)**	**1 390 000<br>(875 000–2 060 000)**
**South Asia**	**Lower respiratory infections**	**8 790 000<br>(6 880 000–10 700 000)**	**6 310 000<br>(526 000–9 610 000)**	**4 430 000<br>(3 020 000–6 100 000)**	**4 430 000<br>(2 850 000–6 110 000)**
**South Asia**	**Malaria**	**179 000<br>(-145 000–598 000)**	**140 000<br>(-97 700–450 000)**	**--**	**76 400<br>(-37 100–360 000)**
**South Asia**	**Measles**	**602 000<br>(231 000–1 270 000)**	**336 000<br>(119 000–717 000)**	**285 000<br>(84 700–623 000)**	**305 000<br>(111 000–653 000)**
South Asia	All causes	15 400 000<br>(12 500 000–18 100 000)	10 800 000<br>(5 050 000–14 600 000)	9 500 000<br>(7 040 000–11 500 000)	6 210 000<br>(4 460 000–8 120 000)
South Asia	Diarrheal diseases	4 020 000<br>(2 660 000–5 630 000)	2 180 000<br>(1 400 000–3 290 000)	2 930 000<br>(-263 000–5 150 000)	1 390 000<br>(875 000–2 060 000)

South Asia	Lower respiratory infections	8 790 000 (6 880 000–10 700 000)	6 310 000 (526 000–9 610 000)	4 430 000 (3 020 000–6 100 000)	4 430 000 (2 850 000–6 110 000)
South Asia	Malaria	179 000 (-145 000–598 000)	140 000 (-97 700–450 000)	--	76 400 (-37 100–360 000)
South Asia	Measles	602 000 (231 000–1 270 000)	336 000 (119 000–717 000)	285 000 (84 700–623 000)	305 000 (111 000–653 000)
Bangladesh	All causes	968 000 (745 000–1 210 000)	725 000 (387 000–999 000)	608 000 (485 000–756 000)	289 000 (191 000–416 000)
Bangladesh	Diarrheal diseases	147 000 (73 800–247 000)	69 800 (38 700–115 000)	102 000 (-5 310–225 000)	43 600 (25 200–68 500)
Bangladesh	Lower respiratory infections	488 000 (326 000–664 000)	340 000 (20 500–589 000)	196 000 (109 000–311 000)	228 000 (140 000–339 000)
Bangladesh	Malaria	107 (-103–392)	107 (-103–391)	--	1·45 (-0·529–7·47)
Bangladesh	Measles	37 600 (14 200–80 100)	19 800 (6 890–42 000)	14 600 (4 250–31 300)	18 000 (6 000–41 000)
Bhutan	All causes	2 810 (1 740–4 080)	1 430 (574–2 460)	1 320 (593–2 420)	1 090 (749–1 570)
Bhutan	Diarrheal diseases	1 170 (484–2 400)	388 (154–823)	787 (-26·5–2 100)	342 (151–668)
Bhutan	Lower respiratory infections	1 560 (987–2 440)	963 (54·8–2 200)	459 (227–745)	749 (449–1 150)
Bhutan	Malaria	0·17 (-0·0911–0·643)	0·144 (-0·073–0·515)	--	0·0364 (-0·0133–0·226)
Bhutan	Measles	1·28 (0·305–2·77)	0·487 (0·114–1·08)	0·357 (0·0685–0·799)	0·705 (0·165–1·56)
India	All causes	10 600 000 (8 580 000–12 400 000)	7 530 000 (3 620 000–10 200 000)	6 610 000 (5 180 000–7 970 000)	4 500 000 (3 220 000–6 090 000)
India	Diarrheal diseases	2 580 000 (1 680 000–3 440 000)	1 470 000 (916 000–2 150 000)	1 900 000 (-188 000–3 250 000)	950 000 (569 000–1 350 000)
India	Lower respiratory infections	6 270 000 (4 880 000–7 680 000)	4 550 000 (385 000–6 930 000)	3 350 000 (2 250 000–4 740 000)	3 240 000 (2 050 000–4 580 000)
India	Malaria	127 000 (-89 400–375 000)	100 000 (-64 000–286 000)	--	51 700 (-26 000–235 000)
India	Measles	500 000 (192 000–1 050 000)	283 000 (101 000–605 000)	244 000 (73 500–540 000)	253 000 (93 300–554 000)
Nepal	All causes	192 000 (146 000–247 000)	135 000 (71 200–197 000)	107 000 (84 700–132 000)	67 600 (47 000–93 900)
Nepal	Diarrheal diseases	23 000 (11 000–42 900)	10 800 (4 780–21 300)	16 100 (-882–35 300)	6 900 (2 910–13 900)
Nepal	Lower respiratory infections	104 000 (66 500–150 000)	71 800 (4 840–138 000)	42 000 (23 600–64 800)	48 000 (29 300–73 300)
Nepal	Malaria	5·51 (-3·9–17·1)	5·19 (-3·65–16·1)	--	0·569 (-0·241–2·85)
Nepal	Measles	25 400 (9 550–46 500)	13 100 (4 790–25 800)	9 460 (2 950–19 200)	12 700 (4 520–24 100)
Pakistan	All causes	3 680 000 (2 480 000–4 830 000)	2 430 000 (972 000–3 420 000)	2 170 000 (1 240 000–3 000 000)	1 350 000 (940 000–1 940 000)
Pakistan	Diarrheal diseases	1 270 000 (626 000–2 150 000)	628 000 (329 000–1 080 000)	914 000 (-69 300–1 880 000)	392 000 (211 000–676 000)
Pakistan	Lower respiratory infections	1 930 000 (1 230 000–2 810 000)	1 350 000 (116 000–2 440 000)	846 000 (474 000–1 350 000)	916 000 (579 000–1 450 000)
Pakistan	Malaria	52 500 (-44 200–245 000)	40 100 (-29 400–180 000)	--	24 700 (-13 600–127 000)
Pakistan	Measles	39 700 (12 500–88 000)	20 700 (6 330–44 600)	16 900 (4 780–36 200)	20 400 (6 470–47 200)
**Southeast Asia, East Asia, and Oceania**	**All causes**	**3 730 000 (2 810 000–4 660 000)**	**2 380 000 (1 020 000–3 520 000)**	**1 840 000 (1 320 000–2 370 000)**	**1 520 000 (1 110 000–1 990 000)**

**Southeast Asia, East Asia, and Oceania**	**Diarrheal diseases**	**924 000 (472 000–1 530 000)**	**417 000 (208 000–751 000)**	**611 000 (-33 600–1 340 000)**	**322 000 (168 000–552 000)**
**Southeast Asia, East Asia, and Oceania**	**Lower respiratory infections**	**2 130 000 (1 500 000–2 800 000)**	**1 430 000 (96 800–2 580 000)**	**737 000 (439 000–1 070 000)**	**1 060 000 (703 000–1 480 000)**
**Southeast Asia, East Asia, and Oceania**	**Malaria**	**12 700 (-8 810–44 300)**	**9 450 (-5 840–30 700)**	**--**	**5 450 (-2 700–26 400)**
**Southeast Asia, East Asia, and Oceania**	**Measles**	**246 000 (91 200–486 000)**	**118 000 (41 700–245 000)**	**82 000 (24 600–172 000)**	**133 000 (49 500–273 000)**
East Asia	All causes	287 000 (184 000–398 000)	179 000 (33 000–343 000)	92 700 (68 900–121 000)	114 000 (82 700–157 000)
East Asia	Diarrheal diseases	21 100 (8 080–34 600)	7 810 (5 650–11 200)	12 300 (-691–27 400)	5 690 (3 440–8 150)
East Asia	Lower respiratory infections	250 000 (160 000–351 000)	154 000 (7 510–319 000)	63 700 (36 400–96 600)	108 000 (77 000–152 000)
East Asia	Malaria	5·55 (-7·17–22·3)	5·55 (-7·17–22·3)	--	<0.001 (>-0.001–<0.001)
East Asia	Measles	17·4 (8·78–23·8)	6·22 (3·08–9·2)	5·42 (2·09–10·8)	8·2 (3·75–12·0)
China	All causes	245 000 (158 000–351 000)	147 000 (26 600–297 000)	79 700 (59 100–106 000)	93 800 (67 900–130 000)
China	Diarrheal diseases	18 100 (7 480–30 800)	6 060 (4 370–8 280)	10 900 (-529–24 100)	4 680 (2 980–6 350)
China	Lower respiratory infections	212 000 (137 000–311 000)	126 000 (4 300–274 000)	53 800 (28 900–84 100)	89 100 (63 400–125 000)
China	Malaria	0 (0–0)	0 (0–0)	--	0 (0–0)
China	Measles	15·7 (8·01–21·7)	6·03 (2·98–8·9)	4·09 (1·55–8·33)	8·01 (3·64–11·9)
Democratic People's Republic of Korea	All causes	40 200 (23 600–59 200)	29 900 (5 400–52 500)	12 100 (7 810–17 900)	19 500 (11 000–30 000)
Democratic People's Republic of Korea	Diarrheal diseases	2 700 (518–5 020)	1 580 (749–2 790)	1 270 (-128–3 300)	931 (224–1 740)
Democratic People's Republic of Korea	Lower respiratory infections	36 200 (20 100–54 500)	26 900 (3 180–49 700)	9 490 (4 990–15 500)	18 600 (9 990–29 100)
Democratic People's Republic of Korea	Malaria	5·55 (-7·17–22·3)	5·55 (-7·17–22·3)	--	<0.001 (>-0.001–<0.001)
Democratic People's Republic of Korea	Measles	0 (0–0)	0 (0–0)	0 (0–0)	0 (0–0)
Taiwan	All causes	2 440 (1 350–3 950)	1 610 (380–3 260)	927 (743–1 130)	566 (447–721)
Taiwan	Diarrheal diseases	356 (-85·1–892)	171 (3·53–423)	137 (-34·3–340)	78·2 (-50·6–249)
Taiwan	Lower respiratory infections	1 740 (1 030–2 900)	1 100 (34·4–2 610)	448 (246–681)	488 (343–694)
Taiwan	Malaria	0 (0–0)	0 (0–0)	--	0 (0–0)
Taiwan	Measles	1·66 (0·201–4·43)	0·188 (0·0541–0·418)	1·32 (-0·134–3·91)	0·192 (0·0621–0·409)
Oceania	All causes	328 000 (240 000–414 000)	220 000 (99 500–309 000)	166 000 (128 000–206 000)	154 000 (105 000–201 000)
Oceania	Diarrheal diseases	44 100 (19 200–77 900)	21 200 (10 200–39 800)	28 900 (-1 680–66 300)	18 700 (8 730–32 900)
Oceania	Lower respiratory infections	164 000 (105 000–227 000)	112 000 (7 470–200 000)	63 400 (37 400–98 100)	95 700 (59 700–139 000)
Oceania	Malaria	8 070 (-5 580–29 500)	5 650 (-3 280–19 300)	--	4 040 (-2 070–17 500)
Oceania	Measles	59 100 (22 700–108 000)	28 600 (9 680–55 200)	21 100 (5 730–45 000)	35 800 (12 500–67 000)

American Samoa	All causes	72·4 (48·6–107)	46·7 (21·6–83·8)	35·4 (27·3–45·5)	18·4 (12·3–26·1)
American Samoa	Diarrheal diseases	6·66 (2·23–13·8)	1·98 (0·97–3·75)	4·53 (-0·196–12·3)	1·32 (0·647–2·3)
American Samoa	Lower respiratory infections	38·9 (21·9–66·0)	24·2 (0·872–59·1)	11·0 (5·74–18·3)	12·8 (7·69–20·8)
American Samoa	Malaria	0 (0–0)	0 (0–0)	--	0 (0–0)
American Samoa	Measles	10·2 (3·59–19·6)	3·85 (1·34–7·99)	3·28 (0·976–7·85)	4·35 (1·34–9·18)
Cook Islands	All causes	12·7 (7·29–22·3)	7·32 (0·597–19·3)	4·3 (2·76–6·52)	3·23 (2·03–4·97)
Cook Islands	Diarrheal diseases	0·344 (-0·00321–0·851)	0·113 (0·0313–0·227)	0·197 (-0·0199–0·558)	0·0667 (-0·0161–0·157)
Cook Islands	Lower respiratory infections	11·8 (6·62–21·1)	6·91 (0·219–18·6)	3·78 (2–6·08)	3·01 (1·76–4·85)
Cook Islands	Malaria	0 (0–0)	0 (0–0)	--	0 (0–0)
Cook Islands	Measles	0·416 (0·121–0·867)	0·137 (0·0399–0·308)	0·159 (0·0388–0·375)	0·154 (0·0456–0·322)
Fiji	All causes	3 370 (2 140–4 830)	2 130 (684–3 860)	1 700 (1 290–2 130)	637 (435–929)
Fiji	Diarrheal diseases	404 (136–771)	121 (59·7–220)	304 (-12–729)	49·5 (27·8–82·3)
Fiji	Lower respiratory infections	2 330 (1 290–3 560)	1 490 (58·0–3 140)	863 (493–1 310)	533 (338–819)
Fiji	Malaria	0 (0–0)	0 (0–0)	--	0 (0–0)
Fiji	Measles	181 (58·6–380)	70·6 (22·6–158)	79·5 (20·8–186)	54·2 (16·0–117)
Guam	All causes	232 (140–369)	134 (23·2–308)	93·6 (66·7–122)	54·7 (34·1–82·5)
Guam	Diarrheal diseases	26·6 (8·06–46·9)	6·31 (3·83–10·2)	19·4 (-0·67–43·2)	4·1 (2·73–6·2)
Guam	Lower respiratory infections	189 (113–317)	112 (2·95–286)	58·1 (31·8–86·7)	50·6 (30·3–77·1)
Guam	Malaria	0 (0–0)	0 (0–0)	--	0 (0–0)
Guam	Measles	0·0484 (-0·0161–0·183)	<0.001 (<0.001–0·00131)	0·0442 (-0·0158–0·17)	0·00387 (>-0.001–0·0184)
Kiribati	All causes	2 000 (1 420–2 600)	1 470 (850–2 080)	1 260 (891–1 620)	432 (279–616)
Kiribati	Diarrheal diseases	354 (169–598)	138 (53·9–251)	233 (-9·59–521)	84·5 (35·7–144)
Kiribati	Lower respiratory infections	788 (496–1 200)	525 (25·9–1 080)	239 (133–392)	300 (187–458)
Kiribati	Malaria	0 (0–0)	0 (0–0)	--	0 (0–0)
Kiribati	Measles	105 (34·6–226)	48·6 (15·5–107)	28·6 (7·91–66·5)	47·6 (15·5–110)
Marshall Islands	All causes	217 (123–323)	168 (41·0–294)	37·1 (27·4–51·5)	72·5 (45·7–108)
Marshall Islands	Diarrheal diseases	18·8 (8·7–35·6)	11·4 (4·97–22·2)	5·44 (-0·146–18·1)	5·34 (2·26–10·2)
Marshall Islands	Lower respiratory infections	169 (79·7–275)	130 (6·62–261)	11·4 (5·59–18·8)	62·9 (37·3–98·5)
Marshall Islands	Malaria	0 (0–0)	0 (0–0)	--	0 (0–0)

Marshall Islands	Measles	9·47 (3·09–19·7)	5·9 (1·85–12·9)	0·417 (0·117–0·957)	4·32 (1·27–9·41)
Micronesia (Federated States of)	All causes	294 (202–383)	206 (117–298)	174 (117–232)	71·5 (45·7–100)
Micronesia (Federated States of)	Diarrheal diseases	47·8 (21·2–91·3)	17·1 (7·32–33·2)	31·0 (-1·25–85·5)	11·8 (5·62–21·8)
Micronesia (Federated States of)	Lower respiratory infections	113 (60·6–174)	72·9 (3·97–157)	30·6 (15·0–51·9)	46·3 (28·2–71·6)
Micronesia (Federated States of)	Malaria	0 (0–0)	0 (0–0)	--	0 (0–0)
Micronesia (Federated States of)	Measles	28·9 (9·42–60·1)	12·2 (4·05–26·5)	8·19 (2·18–18·9)	13·4 (4·05–29·1)
Nauru	All causes	119 (78·6–176)	74·9 (18·5–144)	34·1 (26·9–42·9)	49·0 (32·0–72·2)
Nauru	Diarrheal diseases	9·6 (3·59–19·7)	3·15 (1·24–6·75)	4·88 (-0·162–14·4)	3·4 (1·42–6·73)
Nauru	Lower respiratory infections	93·5 (54·9–148)	56·7 (2·31–130)	14·4 (7·25–23·9)	44·9 (28·5–67·8)
Nauru	Malaria	0 (0–0)	0 (0–0)	--	0 (0–0)
Nauru	Measles	1·07 (0·329–2·39)	0·368 (0·109–0·888)	0·121 (0·0303–0·289)	0·701 (0·208–1·56)
Niue	All causes	2·39 (1·46–3·59)	1·52 (0·377–3·03)	0·96 (0·727–1·25)	0·665 (0·432–1·01)
Niue	Diarrheal diseases	0·157 (0·0429–0·375)	0·046 (0·0195–0·096)	0·106 (-0·00569–0·316)	0·03 (0·0115–0·0597)
Niue	Lower respiratory infections	1·89 (1·07–3·05)	1·16 (0·0445–2·71)	0·536 (0·285–0·893)	0·623 (0·392–0·976)
Niue	Malaria	0 (0–0)	0 (0–0)	--	0 (0–0)
Niue	Measles	0·0297 (0·00903–0·0664)	0·0111 (0·00332–0·0268)	0·00947 (0·00235–0·0241)	0·0126 (0·00381–0·0291)
Northern Mariana Islands	All causes	34·2 (19·7–56·0)	19·6 (7·03–40·4)	15·5 (11·2–22·0)	8·61 (6·09–12·1)
Northern Mariana Islands	Diarrheal diseases	5·41 (1·73–12·1)	1·33 (0·665–2·7)	3·88 (-0·175–11·2)	0·897 (0·475–1·64)
Northern Mariana Islands	Lower respiratory infections	22·1 (11·5–39·9)	13·0 (0·468–34·1)	6·34 (3·18–10·5)	6·85 (4·4–10·5)
Northern Mariana Islands	Malaria	0 (0–0)	0 (0–0)	--	0 (0–0)
Northern Mariana Islands	Measles	2·12 (0·679–4·3)	0·724 (0·236–1·52)	0·736 (0·203–1·8)	0·854 (0·275–1·79)
Palau	All causes	33·5 (20·1–53·2)	19·4 (6·05–40·3)	15·7 (10·7–22·5)	7·98 (4·91–12·9)
Palau	Diarrheal diseases	6·96 (2·05–14·6)	1·87 (0·81–3·88)	5·03 (-0·197–13·7)	1·17 (0·537–2·25)
Palau	Lower respiratory infections	21·7 (12·1–37·4)	13·4 (0·52–34·6)	6·54 (3·2–11·0)	6·37 (3·41–10·7)
Palau	Malaria	0 (0–0)	0 (0–0)	--	0 (0–0)
Palau	Measles	1·11 (0·367–2·34)	0·41 (0·137–0·905)	0·375 (0·105–0·921)	0·446 (0·144–1·01)
Papua New Guinea	All causes	301 000 (221 000–380 000)	202 000 (91 400–285 000)	152 000 (117 000–189 000)	144 000 (97 300–187 000)
Papua New Guinea	Diarrheal diseases	40 400 (17 600–72 000)	19 700 (9 340–37 100)	26 400 (-1 540–60 900)	17 500 (8 150–30 900)
Papua New Guinea	Lower respiratory infections	150 000 (95 500–208 000)	102 000 (6 930–183 000)	58 300 (34 400–90 600)	89 200 (54 800–130 000)

Papua New Guinea	Malaria	7 670<br>(-5 290–28 100)	5 350<br>(-3 090–18 400)	--	3 870<br>(-1 980–16 800)
Papua New Guinea	Measles	55 100<br>(21 100–100 000)	26 700<br>(9 050–51 500)	19 700<br>(5 350–42 200)	33 600<br>(11 700–62 900)
Samoa	All causes	977<br>(590–1 450)	571<br>(150–1 180)	465<br>(328–631)	243<br>(145–382)
Samoa	Diarrheal diseases	75·1<br>(21·3–168)	18·5<br>(8·47–38·7)	56·3<br>(-2·64–155)	12·1<br>(5·75–23·9)
Samoa	Lower respiratory infections	759<br>(439–1 210)	433<br>(18·1–1 050)	287<br>(148–468)	217<br>(125–361)
Samoa	Malaria	0<br>(0–0)	0<br>(0–0)	--	0<br>(0–0)
Samoa	Measles	36·9<br>(10·1–80·0)	12·7<br>(3·49–30·3)	14·9<br>(3·34–37·0)	13·6<br>(3·49–30·3)
Solomon Islands	All causes	2 840<br>(1 910–3 630)	1 870<br>(980–2 760)	1 460<br>(1 080–1 790)	1 020<br>(691–1 410)
Solomon Islands	Diarrheal diseases	298<br>(115–558)	122<br>(60·0–231)	199<br>(-10·1–503)	95·3<br>(46·6–180)
Solomon Islands	Lower respiratory infections	1 200<br>(735–1 750)	779<br>(49·0–1 610)	414<br>(229–656)	563<br>(304–878)
Solomon Islands	Malaria	62·6<br>(-42·1–209)	58·9<br>(-40·6–189)	--	5·44<br>(-2·05–26·9)
Solomon Islands	Measles	674<br>(271–1 220)	299<br>(111–569)	239<br>(70·1–504)	356<br>(121–659)
Tokelau	All causes	2·22<br>(1·32–3·28)	1·4<br>(0·282–2·74)	0·864<br>(0·641–1·18)	0·645<br>(0·405–0·989)
Tokelau	Diarrheal diseases	0·165<br>(0·0458–0·363)	0·0497<br>(0·0188–0·109)	0·111<br>(-0·00556–0·304)	0·0327<br>(0·0116–0·0671)
Tokelau	Lower respiratory infections	1·79<br>(1–2·75)	1·1<br>(0·0426–2·48)	0·507<br>(0·271–0·828)	0·6<br>(0·365–0·961)
Tokelau	Malaria	0<br>(0–0)	0<br>(0–0)	--	0<br>(0–0)
Tokelau	Measles	0·0285<br>(0·00864–0·0623)	0·0108<br>(0·00325–0·0242)	0·00901<br>(0·00224–0·022)	0·0122<br>(0·00384–0·0274)
Tonga	All causes	155<br>(98·2–258)	102<br>(36·9–224)	77·0<br>(55·7–102)	28·6<br>(18·6–45·1)
Tonga	Diarrheal diseases	4·41<br>(0·886–10·7)	0·867<br>(0·474–1·57)	3·35<br>(-0·129–9·62)	0·565<br>(0·264–1)
Tonga	Lower respiratory infections	112<br>(59·2–213)	63·8<br>(1·76–185)	36·0<br>(18·3–59·2)	27·6<br>(17·6–44·1)
Tonga	Malaria	0<br>(0–0)	0<br>(0–0)	--	0<br>(0–0)
Tonga	Measles	1·47<br>(0·387–3·4)	0·419<br>(0·115–1·01)	0·679<br>(0·158–1·82)	0·466<br>(0·132–1·12)
Tuvalu	All causes	65·1<br>(41·9–97·0)	37·5<br>(10·9–74·9)	25·2<br>(18·9–34·4)	23·0<br>(13·8–34·4)
Tuvalu	Diarrheal diseases	8·51<br>(2·87–19·4)	2·26<br>(0·924–4·97)	5·76<br>(-0·275–17·9)	2·16<br>(0·974–4·33)
Tuvalu	Lower respiratory infections	46·8<br>(26·5–74·5)	27·3<br>(1·17–65·9)	11·5<br>(5·93–18·8)	19·5<br>(10·8–30·8)
Tuvalu	Malaria	0<br>(0–0)	0<br>(0–0)	--	0<br>(0–0)
Tuvalu	Measles	2·63<br>(0·874–5·63)	0·882<br>(0·288–1·9)	0·786<br>(0·215–1·87)	1·29<br>(0·412–2·87)
Vanuatu	All causes	2 610<br>(1 810–3 320)	1 470<br>(619–2 310)	1 060<br>(713–1 450)	1 030<br>(731–1 370)
Vanuatu	Diarrheal diseases	570<br>(269–1 020)	211<br>(99·7–386)	370<br>(-15·5–904)	168<br>(84·2–273)

Vanuatu	Lower respiratory infections	1 470<br>(913–2 070)	946<br>(52·5–1 860)	432<br>(237–651)	636<br>(361–939)
Vanuatu	Malaria	0·732<br>(-0·486–2·54)	0·732<br>(-0·486–2·54)	--	<0.001<br>(>-0.001–<0.001)
Vanuatu	Measles	437<br>(179–794)	181<br>(71·0–358)	128<br>(40·7–273)	229<br>(82·8–420)
Southeast Asia	All causes	3 110 000<br>(2 290 000–3 930 000)	1 980 000<br>(892 000–2 880 000)	1 580 000<br>(1 090 000–2 120 000)	1 250 000<br>(896 000–1 680 000)
Southeast Asia	Diarrheal diseases	859 000<br>(437 000–1 420 000)	388 000<br>(191 000–709 000)	570 000<br>(-31 000–1 250 000)	297 000<br>(151 000–518 000)
Southeast Asia	Lower respiratory infections	1 720 000<br>(1 210 000–2 320 000)	1 160 000<br>(81 800–2 140 000)	609 000<br>(353 000–913 000)	853 000<br>(542 000–1 230 000)
Southeast Asia	Malaria	4 670<br>(-2 800–15 800)	3 790<br>(-2 260–11 700)	--	1 410<br>(-602–6 580)
Southeast Asia	Measles	187 000<br>(66 200–383 000)	89 800<br>(32 000–192 000)	60 900<br>(18 100–134 000)	97 100<br>(34 500–210 000)
Cambodia	All causes	116 000<br>(78 300–155 000)	77 800<br>(17 600–129 000)	54 600<br>(40 600–72 700)	50 600<br>(31 600–76 100)
Cambodia	Diarrheal diseases	15 700<br>(6 270–30 900)	6 830<br>(3 000–13 800)	10 900<br>(-631–26 400)	5 040<br>(2 190–9 900)
Cambodia	Lower respiratory infections	91 700<br>(60 900–131 000)	62 000<br>(4 240–115 000)	34 800<br>(19 500–55 200)	45 500<br>(27 600–68 400)
Cambodia	Malaria	180<br>(-148–696)	147<br>(-120–570)	--	56·6<br>(-18·3–287)
Cambodia	Measles	1·8<br>(0·729–3·2)	0·888<br>(0·364–1·68)	0·636<br>(0·217–1·47)	0·945<br>(0·343–1·68)
Indonesia	All causes	1 440 000<br>(1 040 000–1 870 000)	924 000<br>(505 000–1 300 000)	780 000<br>(489 000–1 110 000)	605 000<br>(421 000–864 000)
Indonesia	Diarrheal diseases	491 000<br>(259 000–817 000)	229 000<br>(105 000–436 000)	322 000<br>(-18 500–720 000)	189 000<br>(93 100–337 000)
Indonesia	Lower respiratory infections	680 000<br>(459 000–966 000)	463 000<br>(39 300–858 000)	239 000<br>(129 000–377 000)	375 000<br>(230 000–570 000)
Indonesia	Malaria	1 030<br>(-641–3 170)	837<br>(-515–2 400)	--	327<br>(-125–1 570)
Indonesia	Measles	75 000<br>(27 300–158 000)	36 500<br>(12 500–83 100)	25 600<br>(7 130–56 900)	40 600<br>(14 000–88 900)
Lao People's Democratic Republic	All causes	128 000<br>(84 200–170 000)	80 900<br>(25 100–128 000)	60 400<br>(41 500–81 900)	50 600<br>(34 200–69 600)
Lao People's Democratic Republic	Diarrheal diseases	24 500<br>(10 300–46 700)	10 200<br>(4 440–19 400)	16 900<br>(-789–40 500)	7 390<br>(3 390–14 200)
Lao People's Democratic Republic	Lower respiratory infections	85 500<br>(52 700–123 000)	57 000<br>(3 660–109 000)	31 100<br>(17 000–49 700)	39 000<br>(22 800–59 100)
Lao People's Democratic Republic	Malaria	55·3<br>(-42·4–234)	44·6<br>(-33·8–192)	--	17·0<br>(-5·72–94·7)
Lao People's Democratic Republic	Measles	7 810<br>(2 840–15 800)	3 860<br>(1 310–8 230)	2 530<br>(734–5 310)	4 140<br>(1 370–8 380)
Malaysia	All causes	24 200<br>(17 400–33 200)	16 700<br>(8 030–26 000)	13 600<br>(10 900–17 100)	7 390<br>(5 430–10 400)
Malaysia	Diarrheal diseases	3 820<br>(1 310–6 440)	1 590<br>(1 040–2 400)	2 460<br>(-165–5 190)	908<br>(440–1 440)
Malaysia	Lower respiratory infections	13 300<br>(8 050–19 900)	8 620<br>(441–17 700)	4 680<br>(2 520–7 680)	6 020<br>(4 280–8 770)
Malaysia	Malaria	2·97<br>(-1·56–9·99)	2·39<br>(-1·27–7·39)	--	0·87<br>(-0·336–4·34)
Malaysia	Measles	1 090<br>(331–2 270)	477<br>(143–1 080)	447<br>(110–1 050)	455<br>(129–970)
Maldives	All causes	592<br>(408–749)	391<br>(187–554)	323<br>(201–431)	202<br>(143–279)

Maldives	Diarrheal diseases	205 (95·9–321)	95·5 (56·9–159)	129 (-8·06–268)	62·7 (36·6–101)
Maldives	Lower respiratory infections	291 (191–403)	199 (13·6–364)	97·4 (52·1–153)	139 (85·9–207)
Maldives	Malaria	0 (0–0)	0 (0–0)	--	0 (0–0)
Maldives	Measles	0·362 (0·0863–0·676)	0·171 (0·039–0·33)	0·132 (0·0266–0·282)	0·16 (0·0366–0·305)
Mauritius	All causes	991 (705–1 240)	599 (308–874)	644 (450–776)	228 (167–309)
Mauritius	Diarrheal diseases	304 (119–433)	104 (73·3–146)	228 (-15·6–386)	57·7 (43·6–79·2)
Mauritius	Lower respiratory infections	503 (381–634)	311 (13·9–583)	233 (157–320)	171 (113–241)
Mauritius	Malaria	0 (0–0)	0 (0–0)	--	0 (0–0)
Mauritius	Measles	0·319 (-0·124–1·08)	<0.001 (<0.001–<0.001)	0·302 (-0·115–0·989)	0·0207 (-0·00869–0·0881)
Myanmar	All causes	512 000 (344 000–707 000)	310 000 (98 200–520 000)	227 000 (159 000–329 000)	193 000 (124 000–282 000)
Myanmar	Diarrheal diseases	111 000 (43 300–231 000)	45 200 (19 100–90 400)	75 400 (-3 410–190 000)	30 100 (12 500–58 000)
Myanmar	Lower respiratory infections	325 000 (207 000–472 000)	217 000 (14 000–434 000)	115 000 (63 700–183 000)	135 000 (75 600–224 000)
Myanmar	Malaria	3 320 (-2 130–11 100)	2 700 (-1 600–8 680)	--	984 (-433–4 510)
Myanmar	Measles	53 300 (19 300–99 900)	26 000 (9 190–53 600)	16 300 (5 050–34 300)	27 000 (9 510–54 700)
Philippines	All causes	700 000 (511 000–876 000)	453 000 (186 000–636 000)	360 000 (245 000–470 000)	264 000 (189 000–351 000)
Philippines	Diarrheal diseases	191 000 (105 000–290 000)	85 300 (46 500–137 000)	129 000 (-6 310–269 000)	57 800 (33 900–90 900)
Philippines	Lower respiratory infections	404 000 (289 000–520 000)	275 000 (15 900–469 000)	142 000 (84 300–207 000)	194 000 (130 000–270 000)
Philippines	Malaria	48·1 (-35·2–219)	38·1 (-26·3–168)	--	17·3 (-7·32–91·3)
Philippines	Measles	24 100 (8 660–49 100)	12 100 (4 060–26 300)	7 950 (2 170–18 300)	12 200 (4 000–26 700)
Seychelles	All causes	139 (85·0–198)	81·8 (19·4–154)	59·7 (45·3–74·1)	43·3 (31·3–61·6)
Seychelles	Diarrheal diseases	21·8 (6·66–38·0)	6·48 (4·13–10·5)	15·1 (-0·721–32·6)	4·34 (2·72–6·64)
Seychelles	Lower respiratory infections	103 (63·8–153)	63·0 (2·84–137)	32·4 (19·0–48·6)	38·1 (26·6–55·7)
Seychelles	Malaria	0 (0–0)	0 (0–0)	--	0 (0–0)
Seychelles	Measles	2·2 (0·69–4·66)	0·836 (0·262–1·86)	0·764 (0·202–1·83)	0·923 (0·293–2·09)
Sri Lanka	All causes	14 200 (10 700–17 800)	11 100 (6 720–14 800)	9 870 (7 610–12 400)	3 200 (2 400–4 160)
Sri Lanka	Diarrheal diseases	2 750 (788–4 440)	1 380 (870–2 010)	1 750 (-166–3 510)	716 (266–1 100)
Sri Lanka	Lower respiratory infections	5 660 (3 960–7 230)	3 940 (262–6 640)	2 360 (1 410–3 460)	2 480 (1 550–3 460)
Sri Lanka	Malaria	0 (0–0)	0 (0–0)	--	0 (0–0)
Sri Lanka	Measles	29·9 (16·5–40·0)	15·2 (7·74–21·7)	13·4 (5·55–23·7)	11·6 (5·54–17·0)

Thailand	All causes	37 500 (28 100–47 600)	22 100 (10 000–32 600)	18 000 (12 800–22 900)	13 600 (10 200–17 700)
Thailand	Diarrheal diseases	8 850 (4 120–14 300)	3 350 (2 220–4 810)	5 710 (-280–12 200)	2 470 (1 660–3 490)
Thailand	Lower respiratory infections	20 200 (14 500–26 900)	12 700 (625–23 500)	6 410 (3 650–9 770)	9 300 (6 430–12 800)
Thailand	Malaria	7·91 (-4·63–30·0)	7·83 (-4·62–29·7)	--	0·11 (-0·038–0·559)
Thailand	Measles	3 990 (1 400–7 960)	1 510 (511–3 250)	1 390 (391–3 100)	1 870 (619–3 970)
Timor-Leste	All causes	29 700 (22 000–37 100)	22 100 (7 780–33 000)	17 600 (13 300–23 000)	13 100 (8 800–18 400)
Timor-Leste	Diarrheal diseases	3 030 (1 320–5 720)	1 620 (770–3 150)	2 190 (-157–5 020)	1 170 (540–2 240)
Timor-Leste	Lower respiratory infections	21 200 (14 200–28 500)	15 100 (1 170–26 500)	10 000 (6 090–15 600)	11 900 (7 910–17 500)
Timor-Leste	Malaria	0·191 (-0·173–0·528)	0·179 (-0·17–0·453)	--	0·022 (-0·0101–0·141)
Timor-Leste	Measles	30·8 (17·2–44·9)	17·5 (8·56–26·8)	14·8 (6·05–26·2)	16·5 (7·39–24·7)
Viet Nam	All causes	104 000 (65 700–147 000)	63 000 (18 700–109 000)	38 900 (25 200–56 800)	46 000 (29 200–70 300)
Viet Nam	Diarrheal diseases	5 900 (17·7–11 600)	2 810 (668–5 170)	2 920 (-416–6 990)	1 710 (-170–3 510)
Viet Nam	Lower respiratory infections	71 100 (41 000–102 000)	44 700 (2 080–87 800)	23 100 (11 200–42 700)	33 700 (19 500–57 100)
Viet Nam	Malaria	11·7 (-7·44–47·2)	9·71 (-5·94–33·7)	--	3 (-1·11–16·1)
Viet Nam	Measles	21 000 (7 340–42 000)	9 210 (3 030–19 200)	6 580 (1 860–14 700)	10 600 (3 500–21 900)
**Sub-Saharan Africa**	**All causes**	**55 200 000 (26 700 000–77 100 000)**	**35 800 000 (13 400 000–53 100 000)**	**25 400 000 (14 400 000–36 800 000)**	**23 300 000 (14 600 000–34 000 000)**
**Sub-Saharan Africa**	**Diarrheal diseases**	**15 900 000 (9 100 000–24 400 000)**	**7 600 000 (4 100 000–12 600 000)**	**10 900 000 (-762 000–21 600 000)**	**5 530 000 (2 950 000–8 790 000)**
**Sub-Saharan Africa**	**Lower respiratory infections**	**18 100 000 (11 100 000–25 400 000)**	**12 400 000 (969 000–22 400 000)**	**6 360 000 (3 670 000–10 200 000)**	**10 300 000 (6 660 000–14 400 000)**
**Sub-Saharan Africa**	**Malaria**	**9 360 000 (-6 160 000–29 600 000)**	**6 790 000 (-3 940 000–20 000 000)**	**--**	**4 360 000 (-2 290 000–19 200 000)**
**Sub-Saharan Africa**	**Measles**	**5 800 000 (2 210 000–11 100 000)**	**2 960 000 (1 090 000–5 690 000)**	**2 120 000 (628 000–4 460 000)**	**3 100 000 (1 070 000–6 070 000)**
Central Sub-Saharan Africa	All causes	6 820 000 (2 800 000–10 800 000)	4 320 000 (990 000–7 120 000)	2 370 000 (1 470 000–3 550 000)	3 230 000 (1 720 000–5 460 000)
Central Sub-Saharan Africa	Diarrheal diseases	1 300 000 (650 000–2 300 000)	592 000 (286 000–1 100 000)	858 000 (-59 700–1 940 000)	471 000 (222 000–846 000)
Central Sub-Saharan Africa	Lower respiratory infections	2 320 000 (1 430 000–3 330 000)	1 570 000 (104 000–2 930 000)	748 000 (405 000–1 240 000)	1 320 000 (864 000–1 940 000)
Central Sub-Saharan Africa	Malaria	1 790 000 (-1 140 000–5 120 000)	1 240 000 (-676 000–3 410 000)	--	881 000 (-420 000–3 520 000)
Central Sub-Saharan Africa	Measles	981 000 (357 000–1 970 000)	474 000 (168 000–970 000)	323 000 (89 300–727 000)	559 000 (185 000–1 120 000)
Angola	All causes	1 260 000 (630 000–1 830 000)	812 000 (281 000–1 220 000)	477 000 (261 000–744 000)	579 000 (363 000–807 000)
Angola	Diarrheal diseases	324 000 (155 000–562 000)	153 000 (67 000–292 000)	199 000 (-11 100–482 000)	124 000 (55 200–232 000)
Angola	Lower respiratory infections	502 000 (287 000–721 000)	343 000 (26 300–628 000)	135 000 (70 300–230 000)	291 000 (182 000–419 000)
Angola	Malaria	202 000 (-119 000–636 000)	145 000 (-80 600–433 000)	--	91 400 (-42 400–406 000)

Angola	Measles	125 000 (43 900–259 000)	61 500 (21 200–131 000)	32 800 (9 050–76 600)	73 100 (25 800–165 000)
Central African Republic	All causes	865 000 (481 000–1 160 000)	501 000 (247 000–684 000)	427 000 (139 000–710 000)	378 000 (258 000–465 000)
Central African Republic	Diarrheal diseases	462 000 (266 000–673 000)	214 000 (117 000–357 000)	304 000 (-15 900–614 000)	170 000 (86 800–264 000)
Central African Republic	Lower respiratory infections	270 000 (156 000–407 000)	185 000 (15 200–362 000)	84 600 (41 900–136 000)	159 000 (101 000–226 000)
Central African Republic	Malaria	86 600 (-55 600–251 000)	61 100 (-34 400–175 000)	--	42 700 (-20 700–177 000)
Central African Republic	Measles	12 100 (3 470–28 600)	6 200 (1 790–14 700)	3 730 (865–9 850)	7 010 (1 840–16 700)
Congo	All causes	116 000 (48 500–185 000)	67 300 (22 200–116 000)	54 100 (22 800–93 800)	41 600 (23 500–68 100)
Congo	Diarrheal diseases	45 200 (17 000–84 600)	17 500 (7 340–35 000)	31 500 (-2 010–78 000)	12 900 (5 650–24 500)
Congo	Lower respiratory infections	40 600 (23 100–61 600)	25 900 (1 590–54 200)	13 700 (7 260–22 500)	19 600 (12 400–29 400)
Congo	Malaria	20 700 (-10 900–72 800)	14 900 (-7 090–46 600)	--	8 310 (-3 290–39 900)
Congo	Measles	1 620 (498–3 350)	677 (198–1 540)	568 (144–1 480)	810 (234–1 810)
Democratic Republic of the Congo	All causes	4 540 000 (1 520 000–7 830 000)	2 910 000 (420 000–5 240 000)	1 400 000 (891 000–2 060 000)	2 220 000 (1 020 000–4 170 000)
Democratic Republic of the Congo	Diarrheal diseases	462 000 (170 000–991 000)	205 000 (83 000–457 000)	318 000 (-20 400–852 000)	162 000 (67 600–344 000)
Democratic Republic of the Congo	Lower respiratory infections	1 490 000 (812 000–2 270 000)	1 010 000 (60 700–1 930 000)	511 000 (244 000–910 000)	848 000 (508 000–1 350 000)
Democratic Republic of the Congo	Malaria	1 470 000 (-953 000–4 170 000)	1 010 000 (-563 000–2 770 000)	--	733 000 (-354 000–2 910 000)
Democratic Republic of the Congo	Measles	838 000 (304 000–1 690 000)	404 000 (141 000–831 000)	285 000 (77 600–648 000)	476 000 (152 000–967 000)
Equatorial Guinea	All causes	20 900 (5 150–43 100)	11 900 (504–25 600)	5 740 (3 000–9 450)	8 820 (3 200–21 000)
Equatorial Guinea	Diarrheal diseases	2 680 (782–5 900)	793 (284–1 770)	1 830 (-79·4–5 370)	778 (277–1 570)
Equatorial Guinea	Lower respiratory infections	7 200 (3 390–12 400)	4 170 (208–10 300)	2 120 (992–3 840)	3 370 (1 600–5 930)
Equatorial Guinea	Malaria	7 910 (-3 690–26 500)	5 110 (-2 140–15 000)	--	3 570 (-1 530–15 800)
Equatorial Guinea	Measles	1 960 (558–4 140)	623 (171–1 310)	609 (139–1 540)	1 110 (302–2 550)
Gabon	All causes	17 700 (8 380–28 800)	9 230 (2 900–17 900)	6 710 (3 200–11 900)	6 790 (4 100–10 600)
Gabon	Diarrheal diseases	5 140 (1 860–10 200)	1 570 (583–3 260)	3 390 (-159–9 260)	1 440 (587–2 740)
Gabon	Lower respiratory infections	6 400 (3 490–11 000)	3 780 (177–9 260)	1 590 (757–2 700)	3 020 (1 790–4 740)
Gabon	Malaria	2 810 (-1 260–10 300)	1 960 (-804–6 310)	--	1 090 (-423–5 420)
Gabon	Measles	2 270 (749–4 430)	799 (261–1 620)	612 (164–1 370)	1 240 (358–2 550)
Eastern Sub-Saharan Africa	All causes	13 800 000 (8 400 000–18 800 000)	8 490 000 (4 100 000–12 200 000)	6 390 000 (3 670 000–9 030 000)	5 870 000 (4 240 000–7 720 000)
Eastern Sub-Saharan Africa	Diarrheal diseases	4 360 000 (2 690 000–6 390 000)	2 030 000 (1 130 000–3 340 000)	2 900 000 (-184 000–5 940 000)	1 590 000 (949 000–2 430 000)
Eastern Sub-Saharan Africa	Lower respiratory infections	4 280 000 (2 800 000–5 660 000)	2 870 000 (199 000–5 120 000)	1 390 000 (805 000–2 090 000)	2 380 000 (1 580 000–3 240 000)

Eastern Sub-Saharan Africa	Malaria	1 700 000 (-1 030 000–5 660 000)	1 200 000 (-658 000–3 630 000)	--	797 000 (-400 000–3 710 000)
Eastern Sub-Saharan Africa	Measles	2 030 000 (851 000–3 700 000)	1 010 000 (387 000–1 940 000)	709 000 (227 000–1 430 000)	1 090 000 (412 000–2 010 000)
Burundi	All causes	916 000 (209 000–1 450 000)	639 000 (77 200–1 060 000)	283 000 (157 000–487 000)	468 000 (157 000–846 000)
Burundi	Diarrheal diseases	177 000 (78 200–335 000)	95 600 (40 300–189 000)	104 000 (-6 620–271 000)	82 500 (32 900–159 000)
Burundi	Lower respiratory infections	284 000 (153 000–451 000)	205 000 (19 500–423 000)	74 900 (36 000–126 000)	184 000 (102 000–273 000)
Burundi	Malaria	299 000 (-235 000–807 000)	213 000 (-135 000–583 000)	--	157 000 (-87 600–624 000)
Burundi	Measles	69 000 (21 900–135 000)	38 100 (10 800–78 900)	16 600 (3 940–40 800)	44 300 (13 300–91 600)
Comoros	All causes	18 700 (13 400–24 000)	11 300 (5 570–15 900)	11 100 (5 900–16 100)	7 560 (5 330–10 100)
Comoros	Diarrheal diseases	7 380 (3 480–12 600)	3 250 (1 540–5 950)	5 280 (-354–11 400)	2 600 (1 300–4 410)
Comoros	Lower respiratory infections	9 000 (5 870–12 900)	5 940 (413–11 700)	3 780 (2 180–6 090)	4 690 (2 890–6 990)
Comoros	Malaria	111 (-86·2–450)	81·8 (-59·8–323)	--	47·2 (-21–237)
Comoros	Measles	402 (145–861)	177 (61·5–396)	170 (48·3–390)	215 (72·1–486)
Djibouti	All causes	33 900 (23 800–43 800)	22 000 (14 200–28 600)	24 700 (13 000–35 300)	10 400 (6 900–14 200)
Djibouti	Diarrheal diseases	15 100 (7 220–24 900)	7 530 (3 640–12 800)	11 800 (-988–23 600)	4 560 (2 050–7 590)
Djibouti	Lower respiratory infections	11 100 (6 890–16 500)	7 650 (610–14 200)	6 310 (3 660–9 760)	5 080 (2 980–7 810)
Djibouti	Malaria	418 (-266–2 360)	323 (-191–1 680)	--	166 (-70·9–950)
Djibouti	Measles	1 320 (418–2 810)	675 (208–1 470)	754 (220–1 740)	588 (180–1 320)
Eritrea	All causes	305 000 (237 000–366 000)	215 000 (130 000–266 000)	194 000 (123 000–246 000)	112 000 (86 500–142 000)
Eritrea	Diarrheal diseases	96 900 (50 800–165 000)	51 200 (25 700–88 700)	70 200 (-6 070–142 000)	31 400 (16 900–51 400)
Eritrea	Lower respiratory infections	126 000 (86 200–174 000)	91 000 (8 180–153 000)	55 100 (30 900–85 100)	69 300 (47 100–96 300)
Eritrea	Malaria	1 450 (-1 140–5 720)	1 140 (-735–4 340)	--	613 (-300–2 670)
Eritrea	Measles	20 600 (7 130–44 000)	11 600 (3 950–25 600)	8 880 (2 630–19 700)	10 400 (3 370–22 100)
Ethiopia	All causes	2 540 000 (1 640 000–3 380 000)	1 630 000 (825 000–2 340 000)	1 270 000 (837 000–1 780 000)	1 080 000 (726 000–1 400 000)
Ethiopia	Diarrheal diseases	607 000 (326 000–1 020 000)	289 000 (135 000–533 000)	417 000 (-27 600–924 000)	222 000 (109 000–390 000)
Ethiopia	Lower respiratory infections	840 000 (514 000–1 230 000)	577 000 (49 500–1 090 000)	325 000 (177 000–526 000)	462 000 (281 000–681 000)
Ethiopia	Malaria	223 000 (-162 000–811 000)	162 000 (-100 000–558 000)	--	103 000 (-43 900–493 000)
Ethiopia	Measles	543 000 (220 000–1 040 000)	269 000 (106 000–522 000)	199 000 (63 900–424 000)	297 000 (111 000–585 000)
Kenya	All causes	706 000 (444 000–931 000)	406 000 (172 000–599 000)	317 000 (144 000–483 000)	302 000 (235 000–384 000)
Kenya	Diarrheal diseases	285 000 (168 000–429 000)	119 000 (65 900–194 000)	180 000 (-8 820–382 000)	102 000 (62 000–160 000)

Kenya	Lower respiratory infections	326 000 (204 000–447 000)	213 000 (13 600–409 000)	92 600 (51 800–141 000)	174 000 (113 000–248 000)
Kenya	Malaria	37 200 (-21 600–134 000)	27 000 (-14 300–86 400)	--	15 300 (-6 860–77 300)
Kenya	Measles	20 400 (7 320–42 300)	10 200 (3 510–21 200)	6 160 (1 760–14 300)	10 400 (3 490–22 300)
Madagascar	All causes	1 430 000 (940 000–1 860 000)	902 000 (481 000–1 190 000)	684 000 (289 000–1 060 000)	635 000 (494 000–775 000)
Madagascar	Diarrheal diseases	625 000 (351 000–967 000)	317 000 (153 000–509 000)	391 000 (-18 300–817 000)	252 000 (129 000–387 000)
Madagascar	Lower respiratory infections	417 000 (284 000–559 000)	293 000 (19 000–479 000)	127 000 (67 800–199 000)	247 000 (155 000–353 000)
Madagascar	Malaria	123 000 (-80 600–376 000)	91 000 (-49 600–275 000)	--	56 000 (-26 500–254 000)
Madagascar	Measles	140 000 (51 500–285 000)	74 700 (27 100–153 000)	40 900 (11 600–92 600)	80 300 (25 600–157 000)
Malawi	All causes	452 000 (223 000–720 000)	241 000 (110 000–390 000)	167 000 (64 900–306 000)	205 000 (127 000–333 000)
Malawi	Diarrheal diseases	165 000 (83 100–279 000)	60 900 (27 700–107 000)	96 500 (-3 420–244 000)	63 000 (32 800–104 000)
Malawi	Lower respiratory infections	95 200 (51 200–144 000)	59 300 (2 620–119 000)	19 300 (8 880–34 200)	55 900 (32 300–85 000)
Malawi	Malaria	80 600 (-44 400–317 000)	53 300 (-26 400–183 000)	--	38 500 (-15 800–206 000)
Malawi	Measles	74 900 (25 200–149 000)	30 300 (9 580–58 900)	14 500 (3 590–34 400)	47 400 (14 800–95 000)
Mozambique	All causes	1 260 000 (697 000–1 830 000)	752 000 (280 000–1 150 000)	524 000 (253 000–789 000)	554 000 (410 000–761 000)
Mozambique	Diarrheal diseases	412 000 (215 000–628 000)	172 000 (82 900–297 000)	255 000 (-11 700–555 000)	159 000 (88 400–255 000)
Mozambique	Lower respiratory infections	490 000 (323 000–659 000)	320 000 (15 800–590 000)	137 000 (75 100–220 000)	267 000 (161 000–392 000)
Mozambique	Malaria	167 000 (-95 100–574 000)	114 000 (-57 000–354 000)	--	78 100 (-36 100–385 000)
Mozambique	Measles	83 700 (27 500–178 000)	35 900 (11 000–78 600)	21 500 (5 270–52 500)	50 600 (15 800–113 000)
Rwanda	All causes	295 000 (167 000–433 000)	168 000 (55 800–278 000)	93 800 (55 600–155 000)	153 000 (104 000–197 000)
Rwanda	Diarrheal diseases	71 500 (35 200–127 000)	27 000 (11 700–52 900)	39 400 (-1 660–106 000)	30 600 (14 900–55 200)
Rwanda	Lower respiratory infections	139 000 (84 000–200 000)	86 500 (5 260–179 000)	25 600 (13 400–42 500)	85 800 (58 800–121 000)
Rwanda	Malaria	33 100 (-18 100–118 000)	20 300 (-10 200–64 800)	--	18 200 (-8 370–82 600)
Rwanda	Measles	26 900 (9 290–52 200)	10 500 (3 430–20 800)	4 650 (1 190–10 700)	18 200 (6 020–37 400)
Somalia	All causes	2 100 000 (1 490 000–2 610 000)	1 210 000 (862 000–1 480 000)	1 200 000 (592 000–1 750 000)	831 000 (539 000–1 080 000)
Somalia	Diarrheal diseases	838 000 (435 000–1 310 000)	427 000 (207 000–733 000)	602 000 (-44 700–1 190 000)	277 000 (138 000–463 000)
Somalia	Lower respiratory infections	283 000 (167 000–443 000)	197 000 (15 300–377 000)	119 000 (59 500–205 000)	150 000 (88 200–231 000)
Somalia	Malaria	26 300 (-16 800–90 500)	20 200 (-11 100–65 700)	--	10 400 (-4 180–43 800)
Somalia	Measles	804 000 (354 000–1 290 000)	424 000 (166 000–735 000)	329 000 (111 000–620 000)	393 000 (143 000–663 000)
South Sudan	All causes	960 000 (553 000–1 270 000)	609 000 (325 000–821 000)	618 000 (281 000–904 000)	335 000 (225 000–443 000)

South Sudan	Diarrheal diseases	471 000 (221 000–733 000)	238 000 (122 000–403 000)	362 000 (-29 200–696 000)	148 000 (74 200–244 000)
South Sudan	Lower respiratory infections	280 000 (165 000–428 000)	195 000 (16 600–381 000)	148 000 (81 500–228 000)	138 000 (82 500–207 000)
South Sudan	Malaria	91 300 (-57 500–285 000)	69 200 (-38 100–203 000)	--	39 100 (-17 500–169 000)
South Sudan	Measles	21 700 (6 430–48 600)	11 300 (3 310–26 300)	12 100 (3 160–28 200)	9 960 (2 680–23 600)
Uganda	All causes	1 070 000 (254 000–2 100 000)	691 000 (82 400–1 310 000)	342 000 (189 000–566 000)	434 000 (178 000–924 000)
Uganda	Diarrheal diseases	191 000 (74 600–361 000)	76 300 (29 800–161 000)	120 000 (-7 110–304 000)	65 600 (24 800–135 000)
Uganda	Lower respiratory infections	314 000 (164 000–513 000)	204 000 (12 900–439 000)	85 600 (41 600–150 000)	164 000 (88 400–256 000)
Uganda	Malaria	370 000 (-197 000–1 300 000)	261 000 (-127 000–812 000)	--	158 000 (-66 700–720 000)
Uganda	Measles	82 000 (22 800–190 000)	35 000 (9 510–82 000)	21 400 (5 090–55 700)	46 700 (12 300–109 000)
United Republic of Tanzania	All causes	1 080 000 (643 000–1 570 000)	668 000 (251 000–1 070 000)	409 000 (296 000–540 000)	475 000 (341 000–705 000)
United Republic of Tanzania	Diarrheal diseases	217 000 (109 000–332 000)	83 100 (44 000–143 000)	133 000 (-7 340–287 000)	80 500 (45 300–130 000)
United Republic of Tanzania	Lower respiratory infections	437 000 (272 000–629 000)	279 000 (11 100–527 000)	110 000 (59 800–179 000)	243 000 (152 000–360 000)
United Republic of Tanzania	Malaria	179 000 (-101 000–619 000)	120 000 (-60 800–368 000)	--	84 900 (-35 700–404 000)
United Republic of Tanzania	Measles	108 000 (39 600–214 000)	45 600 (16 300–88 700)	24 800 (6 650–56 300)	66 400 (22 500–131 000)
Zambia	All causes	566 000 (311 000–829 000)	322 000 (140 000–534 000)	250 000 (139 000–388 000)	255 000 (167 000–353 000)
Zambia	Diarrheal diseases	178 000 (97 300–301 000)	62 700 (28 900–119 000)	113 000 (-4 460–266 000)	69 400 (37 000–116 000)
Zambia	Lower respiratory infections	221 000 (136 000–332 000)	135 000 (8 030–294 000)	58 100 (29 500–100 000)	130 000 (84 500–196 000)
Zambia	Malaria	68 900 (-39 700–225 000)	42 300 (-20 800–125 000)	--	37 600 (-16 500–163 000)
Zambia	Measles	28 000 (10 200–58 600)	10 600 (3 530–22 500)	7 700 (1 960–17 100)	17 700 (6 120–38 800)
Southern Sub-Saharan Africa	All causes	1 400 000 (1 070 000–1 720 000)	881 000 (516 000–1 180 000)	740 000 (485 000–992 000)	504 000 (357 000–659 000)
Southern Sub-Saharan Africa	Diarrheal diseases	419 000 (246 000–588 000)	145 000 (79 700–231 000)	241 000 (-7 510–531 000)	167 000 (111 000–242 000)
Southern Sub-Saharan Africa	Lower respiratory infections	584 000 (407 000–777 000)	358 000 (17 900–713 000)	129 000 (74 500–195 000)	317 000 (196 000–453 000)
Southern Sub-Saharan Africa	Malaria	5 940 (-3 450–24 100)	4 130 (-2 190–16 000)	--	2 640 (-1 000–12 400)
Southern Sub-Saharan Africa	Measles	27 300 (9 590–57 500)	10 300 (3 440–23 500)	5 950 (1 610–14 200)	16 900 (5 840–37 700)
Botswana	All causes	32 300 (23 200–40 500)	20 100 (10 300–29 600)	19 600 (12 400–25 400)	11 600 (7 610–16 700)
Botswana	Diarrheal diseases	8 710 (3 970–15 700)	3 050 (1 350–5 930)	6 070 (-263–14 000)	3 220 (1 660–5 640)
Botswana	Lower respiratory infections	15 600 (10 600–22 100)	9 460 (484–19 300)	5 900 (3 330–9 190)	8 050 (4 410–12 600)
Botswana	Malaria	28·5 (-17·6–124)	17·7 (-9·49–70·1)	--	15·2 (-6·86–82·4)
Botswana	Measles	624 (203–1 330)	226 (71·2–517)	226 (61·1–513)	356 (118–775)

Eswatini	All causes	24 600<br>(18 000–32 200)	15 400<br>(5 960–26 200)	8 860<br>(6 670–11 800)	10 700<br>(6 880–15 300)
Eswatini	Diarrheal diseases	3 460<br>(1 440–7 060)	1 160<br>(447–2 380)	1 840<br>(-52·1–5 440)	1 410<br>(637–2 730)
Eswatini	Lower respiratory infections	16 500<br>(10 200–23 300)	9 900<br>(449–21 000)	2 780<br>(1 490–4 550)	8 960<br>(5 320–13 700)
Eswatini	Malaria	22·3<br>(-12·1–112)	13·0<br>(-6·13–61·8)	--	12·1<br>(-4·81–73·1)
Eswatini	Measles	471<br>(159–1 020)	160<br>(50·7–370)	66·7<br>(17·3–158)	320<br>(104–700)
Lesotho	All causes	47 300<br>(34 100–60 900)	28 400<br>(11 100–43 300)	19 400<br>(11 300–29 400)	21 800<br>(14 200–29 800)
Lesotho	Diarrheal diseases	12 900<br>(5 790–23 700)	5 020<br>(2 230–10 100)	7 580<br>(-244–19 900)	5 290<br>(2 390–9 960)
Lesotho	Lower respiratory infections	28 900<br>(19 700–39 700)	18 500<br>(952–35 000)	7 040<br>(3 920–10 700)	15 900<br>(9 360–23 600)
Lesotho	Malaria	0<br>(0–0)	0<br>(0–0)	--	0<br>(0–0)
Lesotho	Measles	951<br>(345–2 080)	393<br>(130–911)	192<br>(50·8–485)	610<br>(205–1 240)
Namibia	All causes	51 200<br>(37 400–64 500)	34 700<br>(17 200–49 700)	31 000<br>(22 300–39 000)	14 700<br>(9 730–20 900)
Namibia	Diarrheal diseases	9 780<br>(4 310–18 200)	3 590<br>(1 480–7 000)	7 040<br>(-365–16 900)	2 640<br>(1 210–4 760)
Namibia	Lower respiratory infections	26 500<br>(17 000–36 700)	16 900<br>(985–32 500)	10 100<br>(5 760–16 000)	11 400<br>(6 850–17 600)
Namibia	Malaria	442<br>(-265–2 770)	322<br>(-186–2 010)	--	183<br>(-69·6–1 160)
Namibia	Measles	943<br>(302–2 090)	408<br>(128–968)	342<br>(86·9–836)	464<br>(140–1 040)
South Africa	All causes	726 000<br>(563 000–904 000)	425 000<br>(245 000–605 000)	337 000<br>(216 000–480 000)	298 000<br>(207 000–390 000)
South Africa	Diarrheal diseases	243 000<br>(148 000–331 000)	80 300<br>(45 400–123 000)	130 000<br>(-3 510–296 000)	105 000<br>(71 400–140 000)
South Africa	Lower respiratory infections	328 000<br>(231 000–437 000)	196 000<br>(8 850–404 000)	60 900<br>(35 000–90 800)	186 000<br>(108 000–261 000)
South Africa	Malaria	47·3<br>(-22·7–275)	32·1<br>(-14·3–178)	--	20·3<br>(-6·87–139)
South Africa	Measles	11 000<br>(3 880–23 200)	3 650<br>(1 180–7 780)	2 000<br>(534–4 590)	7 280<br>(2 510–15 800)
Zimbabwe	All causes	519 000<br>(379 000–640 000)	358 000<br>(229 000–468 000)	324 000<br>(211 000–430 000)	146 000<br>(101 000–197 000)
Zimbabwe	Diarrheal diseases	142 000<br>(70 500–234 000)	52 100<br>(24 500–92 900)	88 300<br>(-3 080–206 000)	49 400<br>(26 400–83 100)
Zimbabwe	Lower respiratory infections	169 000<br>(97 600–242 000)	107 000<br>(5 860–212 000)	42 700<br>(22 700–68 100)	86 700<br>(51 000–131 000)
Zimbabwe	Malaria	5 400<br>(-3 090–20 700)	3 750<br>(-1 970–13 300)	--	2 410<br>(-911–11 500)
Zimbabwe	Measles	13 300<br>(4 670–28 700)	5 500<br>(1 800–12 600)	3 120<br>(829–7 510)	7 820<br>(2 590–17 400)
Western Sub-Saharan Africa	All causes	33 200 000<br>(15 100 000–46 400 000)	22 100 000<br>(7 880 000–32 600 000)	15 900 000<br>(8 650 000–23 100 000)	13 700 000<br>(8 150 000–20 600 000)
Western Sub-Saharan Africa	Diarrheal diseases	9 790 000<br>(5 410 000–15 500 000)	4 830 000<br>(2 600 000–8 020 000)	6 900 000<br>(-510 000–13 900 000)	3 300 000<br>(1 680 000–5 470 000)
Western Sub-Saharan Africa	Lower respiratory infections	10 900 000<br>(6 510 000–15 900 000)	7 630 000<br>(648 000–14 100 000)	4 100 000<br>(2 240 000–6 620 000)	6 280 000<br>(3 860 000–9 060 000)
Western Sub-Saharan Africa	Malaria	5 870 000<br>(-4 230 000–18 400 000)	4 350 000<br>(-2 580 000–13 000 000)	--	2 680 000<br>(-1 430 000–11 900 000)

Western Sub-Saharan Africa	Measles	2 760 000 (981 000–5 520 000)	1 470 000 (519 000–2 980 000)	1 080 000 (316 000–2 380 000)	1 430 000 (472 000–2 930 000)
Benin	All causes	620 000 (226 000–1 020 000)	411 000 (96 700–715 000)	233 000 (137 000–362 000)	258 000 (147 000–486 000)
Benin	Diarrheal diseases	110 000 (49 100–210 000)	47 600 (18 900–98 300)	72 000 (-4 260–180 000)	37 300 (15 100–75 600)
Benin	Lower respiratory infections	216 000 (126 000–321 000)	144 000 (9 760–283 000)	63 500 (32 300–108 000)	123 000 (69 800–179 000)
Benin	Malaria	155 000 (-96 500–495 000)	112 000 (-59 500–327 000)	--	66 600 (-31 000–324 000)
Benin	Measles	58 200 (17 500–131 000)	27 300 (8 390–61 200)	17 700 (4 470–44 800)	31 900 (9 780–72 000)
Burkina Faso	All causes	950 000 (424 000–1 520 000)	649 000 (138 000–1 110 000)	382 000 (246 000–532 000)	357 000 (233 000–629 000)
Burkina Faso	Diarrheal diseases	179 000 (95 300–262 000)	87 000 (43 100–147 000)	126 000 (-9 350–230 000)	55 200 (29 800–91 700)
Burkina Faso	Lower respiratory infections	299 000 (199 000–422 000)	208 000 (13 300–367 000)	117 000 (68 500–177 000)	148 000 (101 000–205 000)
Burkina Faso	Malaria	248 000 (-150 000–738 000)	196 000 (-108 000–540 000)	--	89 100 (-37 500–413 000)
Burkina Faso	Measles	137 000 (49 300–282 000)	71 600 (25 200–144 000)	53 500 (15 900–118 000)	64 400 (21 500–132 000)
Cabo Verde	All causes	1 560 (952–2 270)	894 (478–1 350)	903 (501–1 460)	395 (269–576)
Cabo Verde	Diarrheal diseases	537 (187–1 030)	172 (70·3–347)	388 (-21·1–975)	111 (50·9–209)
Cabo Verde	Lower respiratory infections	656 (395–1 060)	418 (23·2–944)	217 (115–349)	240 (145–383)
Cabo Verde	Malaria	0·56 (-0·371–1·53)	0·56 (-0·371–1·53)	--	<0.001 (>-0.001–<0.001)
Cabo Verde	Measles	104 (35·8–220)	42·6 (14·5–95·7)	37·7 (10·4–85·4)	43·4 (14·5–97·6)
Cameroon	All causes	1 450 000 (705 000–2 210 000)	811 000 (298 000–1 330 000)	592 000 (253 000–1 050 000)	614 000 (391 000–907 000)
Cameroon	Diarrheal diseases	510 000 (229 000–897 000)	198 000 (88 900–365 000)	332 000 (-16 600–790 000)	169 000 (80 100–290 000)
Cameroon	Lower respiratory infections	461 000 (260 000–689 000)	293 000 (17 000–616 000)	125 000 (62 600–214 000)	256 000 (163 000–374 000)
Cameroon	Malaria	247 000 (-145 000–775 000)	168 000 (-87 600–478 000)	--	116 000 (-50 000–474 000)
Cameroon	Measles	131 000 (45 500–256 000)	56 200 (18 400–111 000)	38 000 (9 750–85 900)	73 100 (24 300–151 000)
Chad	All causes	3 380 000 (2 340 000–4 080 000)	1 950 000 (1 450 000–2 310 000)	2 140 000 (613 000–3 110 000)	1 180 000 (933 000–1 450 000)
Chad	Diarrheal diseases	2 080 000 (1 300 000–2 790 000)	1 020 000 (565 000–1 590 000)	1 510 000 (-99 600–2 670 000)	635 000 (376 000–944 000)
Chad	Lower respiratory infections	689 000 (422 000–1 060 000)	481 000 (39 000–940 000)	293 000 (146 000–481 000)	360 000 (220 000–528 000)
Chad	Malaria	119 000 (-76 300–387 000)	91 400 (-51 700–279 000)	--	49 100 (-21 600–229 000)
Chad	Measles	298 000 (98 600–613 000)	157 000 (49 800–336 000)	136 000 (39 600–301 000)	140 000 (40 700–291 000)
Côte d'Ivoire	All causes	1 160 000 (453 000–1 860 000)	741 000 (151 000–1 280 000)	472 000 (271 000–701 000)	424 000 (240 000–653 000)
Côte d'Ivoire	Diarrheal diseases	255 000 (105 000–474 000)	102 000 (42 400–206 000)	180 000 (-12 100–426 000)	70 200 (33 000–133 000)
Côte d'Ivoire	Lower respiratory infections	499 000 (286 000–765 000)	326 000 (20 900–677 000)	173 000 (90 300–295 000)	234 000 (140 000–353 000)

Côte d'Ivoire	Malaria	250 000 (-131 000–797 000)	188 000 (-88 500–543 000)	--	91 600 (-37 700–390 000)
Côte d'Ivoire	Measles	58 500 (19 300–134 000)	26 300 (8 590–62 100)	20 700 (5 580–50 900)	28 100 (8 580–63 400)
Gambia	All causes	54 000 (36 400–69 000)	35 400 (13 000–52 800)	23 800 (18 500–29 700)	20 300 (15 800–25 800)
Gambia	Diarrheal diseases	7 190 (3 130–11 500)	2 960 (1 370–5 640)	4 920 (-323–10 200)	1 920 (962–3 430)
Gambia	Lower respiratory infections	29 400 (19 600–39 200)	19 600 (1 310–34 200)	9 530 (4 940–14 800)	14 100 (9 520–19 400)
Gambia	Malaria	2 980 (-1 850–11 100)	2 440 (-1 420–8 990)	--	853 (-308–4 560)
Gambia	Measles	7 690 (2 840–14 400)	3 690 (1 280–7 340)	2 690 (781–5 660)	3 450 (1 090–6 780)
Ghana	All causes	413 000 (158 000–710 000)	295 000 (77 100–534 000)	182 000 (114 000–267 000)	118 000 (55 400–242 000)
Ghana	Diarrheal diseases	58 100 (25 700–109 000)	22 500 (9 170–45 700)	41 500 (-2 440–93 600)	14 000 (5 750–27 300)
Ghana	Lower respiratory infections	132 000 (69 800–206 000)	86 300 (3 970–173 000)	46 700 (22 200–81 900)	58 000 (32 500–92 800)
Ghana	Malaria	114 000 (-56 800–408 000)	90 300 (-43 800–289 000)	--	35 800 (-13 800–170 000)
Ghana	Measles	23 700 (7 290–50 300)	10 700 (3 350–22 200)	8 790 (2 330–20 400)	10 500 (3 100–23 700)
Guinea	All causes	843 000 (438 000–1 240 000)	536 000 (152 000–868 000)	328 000 (221 000–446 000)	377 000 (216 000–573 000)
Guinea	Diarrheal diseases	98 800 (40 200–204 000)	43 200 (16 200–84 700)	68 700 (-4 630–172 000)	32 100 (13 400–61 900)
Guinea	Lower respiratory infections	347 000 (206 000–518 000)	231 000 (16 700–455 000)	123 000 (66 400–202 000)	185 000 (113 000–268 000)
Guinea	Malaria	149 000 (-91 300–456 000)	106 000 (-57 400–303 000)	--	67 600 (-30 400–288 000)
Guinea	Measles	177 000 (68 500–344 000)	83 300 (30 200–164 000)	64 900 (18 900–145 000)	91 900 (30 000–183 000)
Guinea-Bissau	All causes	82 800 (55 000–109 000)	50 300 (24 400–71 700)	34 900 (21 200–53 500)	37 500 (27 400–48 300)
Guinea-Bissau	Diarrheal diseases	22 400 (10 600–39 900)	9 880 (4 340–18 600)	14 200 (-735–34 600)	8 080 (3 780–14 400)
Guinea-Bissau	Lower respiratory infections	31 100 (19 000–44 900)	20 900 (1 520–37 800)	8 290 (4 050–13 700)	18 400 (12 300–25 900)
Guinea-Bissau	Malaria	5 260 (-3 040–18 000)	3 920 (-2 170–12 200)	--	2 140 (-861–9 830)
Guinea-Bissau	Measles	16 100 (6 000–30 700)	7 690 (2 780–15 700)	4 550 (1 310–10 200)	8 870 (3 080–16 900)
Liberia	All causes	241 000 (119 000–359 000)	147 000 (46 700–246 000)	93 400 (54 600–138 000)	92 700 (60 400–135 000)
Liberia	Diarrheal diseases	55 000 (21 500–97 400)	21 300 (8 420–42 300)	36 600 (-1 790–80 400)	16 200 (6 710–31 700)
Liberia	Lower respiratory infections	86 300 (46 300–136 000)	56 600 (3 810–126 000)	24 900 (12 600–41 700)	41 100 (23 400–64 800)
Liberia	Malaria	41 700 (-21 900–138 000)	30 800 (-15 300–92 900)	--	16 000 (-6 540–75 200)
Liberia	Measles	37 200 (13 200–74 300)	16 700 (5 560–34 300)	10 800 (2 890–23 700)	19 500 (6 230–40 000)
Mali	All causes	1 620 000 (867 000–2 330 000)	1 020 000 (412 000–1 530 000)	746 000 (468 000–1 060 000)	580 000 (381 000–979 000)
Mali	Diarrheal diseases	401 000 (177 000–678 000)	180 000 (89 100–314 000)	286 000 (-17 800–598 000)	110 000 (56 200–194 000)

Mali	Lower respiratory infections	351 000 (225 000–506 000)	238 000 (17 100–453 000)	134 000 (73 600–213 000)	175 000 (111 000–255 000)
Mali	Malaria	304 000 (-184 000–970 000)	236 000 (-128 000–693 000)	--	112 000 (-47 400–515 000)
Mali	Measles	404 000 (150 000–784 000)	204 000 (71 600–400 000)	161 000 (47 200–338 000)	182 000 (58 400–350 000)
Mauritania	All causes	89 000 (52 200–122 000)	53 400 (27 300–76 300)	51 500 (23 300–80 800)	26 900 (19 500–36 200)
Mauritania	Diarrheal diseases	38 400 (19 100–65 800)	16 400 (7 840–28 300)	27 700 (-1 810–62 100)	9 350 (4 420–16 400)
Mauritania	Lower respiratory infections	31 500 (18 200–48 900)	21 400 (1 520–43 200)	12 000 (6 300–19 900)	14 000 (8 970–20 600)
Mauritania	Malaria	4 340 (-2 520–18 600)	3 460 (-1 880–15 600)	--	1 450 (-525–7 260)
Mauritania	Measles	4 890 (1 590–9 980)	2 410 (781–5 170)	1 930 (536–4 700)	2 130 (677–4 810)
Niger	All causes	4 430 000 (2 170 000–5 790 000)	3 220 000 (1 260 000–4 290 000)	2 140 000 (1 330 000–3 020 000)	2 000 000 (1 180 000–2 870 000)
Niger	Diarrheal diseases	1 160 000 (614 000–1 900 000)	684 000 (352 000–1 180 000)	801 000 (-71 500–1 640 000)	452 000 (225 000–788 000)
Niger	Lower respiratory infections	1 460 000 (903 000–2 140 000)	1 090 000 (114 000–1 890 000)	565 000 (306 000–916 000)	916 000 (600 000–1 340 000)
Niger	Malaria	736 000 (-622 000–2 130 000)	558 000 (-388 000–1 550 000)	--	366 000 (-207 000–1 480 000)
Niger	Measles	489 000 (163 000–1 030 000)	299 000 (98 300–661 000)	187 000 (51 900–426 000)	269 000 (79 500–570 000)
Nigeria	All causes	16 700 000 (6 300 000–24 000 000)	11 500 000 (3 320 000–17 400 000)	7 960 000 (4 140 000–11 600 000)	7 190 000 (3 710 000–11 600 000)
Nigeria	Diarrheal diseases	4 390 000 (2 080 000–7 560 000)	2 210 000 (1 060 000–3 810 000)	3 100 000 (-244 000–6 860 000)	1 560 000 (734 000–2 790 000)
Nigeria	Lower respiratory infections	5 930 000 (3 290 000–8 510 000)	4 170 000 (376 000–7 620 000)	2 280 000 (1 140 000–3 800 000)	3 560 000 (2 020 000–5 310 000)
Nigeria	Malaria	3 330 000 (-2 580 000–10 300 000)	2 450 000 (-1 530 000–7 390 000)	--	1 600 000 (-907 000–7 070 000)
Nigeria	Measles	846 000 (293 000–1 700 000)	472 000 (155 000–975 000)	349 000 (96 000–810 000)	472 000 (150 000–995 000)
Sao Tome and Principe	All causes	2 190 (1 660–2 810)	1 590 (994–2 260)	1 370 (1 010–1 810)	583 (391–812)
Sao Tome and Principe	Diarrheal diseases	200 (75·2–391)	68·9 (25·7–139)	134 (-6·68–348)	59·8 (23·4–114)
Sao Tome and Principe	Lower respiratory infections	782 (483–1 220)	479 (25·9–982)	221 (104–367)	385 (232–588)
Sao Tome and Principe	Malaria	26·5 (-13·7–100)	19·6 (-9·84–66·8)	--	9·5 (-3·48–52·1)
Sao Tome and Principe	Measles	260 (90·1–528)	101 (34·0–218)	86·5 (25·3–192)	129 (44·4–275)
Senegal	All causes	248 000 (158 000–328 000)	151 000 (75 100–221 000)	124 000 (79 200–167 000)	79 300 (55 200–111 000)
Senegal	Diarrheal diseases	60 500 (27 200–95 400)	23 800 (11 500–43 600)	42 300 (-2 670–82 900)	15 000 (7 330–25 000)
Senegal	Lower respiratory infections	82 200 (47 200–125 000)	54 300 (2 530–108 000)	27 800 (14 000–48 500)	36 600 (23 000–56 700)
Senegal	Malaria	19 000 (-10 200–71 500)	15 100 (-7 960–52 500)	--	5 850 (-2 020–30 800)
Senegal	Measles	52 400 (19 200–96 000)	23 900 (8 420–46 500)	20 400 (6 170–42 500)	21 900 (6 950–42 000)
Sierra Leone	All causes	574 000 (257 000–879 000)	344 000 (101 000–573 000)	264 000 (114 000–400 000)	215 000 (143 000–346 000)

Sierra Leone	Diarrheal diseases	217 000 (107 000–345 000)	90 400 (45 100–165 000)	148 000 (-8 640–305 000)	68 100 (36 700–117 000)
Sierra Leone	Lower respiratory infections	196 000 (121 000–292 000)	130 000 (5 520–257 000)	67 500 (35 300–113 000)	93 800 (56 300–151 000)
Sierra Leone	Malaria	104 000 (-59 400–367 000)	73 200 (-36 200–235 000)	--	45 600 (-20 600–215 000)
Sierra Leone	Measles	14 200 (4 260–31 900)	6 390 (1 810–14 800)	5 210 (1 250–13 900)	7 200 (1 870–16 600)
Togo	All causes	296 000 (148 000–433 000)	171 000 (67 600–253 000)	149 000 (46 200–265 000)	101 000 (64 000–133 000)
Togo	Diarrheal diseases	149 000 (74 200–245 000)	63 800 (30 900–114 000)	102 000 (-6 560–227 000)	42 600 (22 100–74 400)
Togo	Lower respiratory infections	81 700 (45 900–123 000)	54 800 (4 220–110 000)	25 800 (12 900–43 300)	42 000 (25 200–61 900)
Togo	Malaria	38 200 (-22 100–130 000)	29 900 (-16 900–92 500)	--	13 100 (-5 720–65 900)
Togo	Measles	6 920 (2 090–14 600)	3 310 (1 010–7 210)	2 330 (571–5 860)	3 330 (966–7 640)

Table S2. All-cause and cause-specific population attributable fraction (PAF, %) of DALYs among children under 5 years for child growth failure at the global and super-regional, regional, and national levels, 2023 Estimates combine burden associated with mild, moderate, and severe forms of CGF: stunting was defined as height-for-age z-score (HAZ) < –1; underweight as weight-for-age z-score (WAZ) < –1; wasting as weight-for-height z-score (WHZ) < –1, according to WHO Child Growth Standards.

Location	Cause name	Child growth failure	Child underweight	Child wasting	Child stunting
**Global**	**All causes**	**17·9 (10·6–23·8)**	**11·7 (5·0–16·8)**	**8·8 (5·4–11·9)**	**7·4 (5·6–9·5)**
**Global**	**Diarrheal diseases**	**75·2 (53·1–88·1)**	**36·6 (24·9–47·3)**	**51·9 (-3·1–84·2)**	**26·2 (18·6–31·9)**
**Global**	**Lower respiratory infections**	**59·1 (48·5–66·5)**	**40·5 (2·7–63·0)**	**23·0 (16·0–29·3)**	**31·8 (24·3–37·9)**
**Global**	**Malaria**	**26·3 (-17·8–69·4)**	**19·1 (-11·6–48·9)**	**--**	**12·1 (-6·2–44·7)**
**Global**	**Measles**	**66·4 (39·9–75·8)**	**34·0 (18·3–42·4)**	**24·9 (10·8–40·9)**	**35·3 (17·3–43·5)**
**Central Europe, Eastern Europe, and Central Asia**	**All causes**	**11·2 (8·2–14·7)**	**6·2 (0·76–12·6)**	**4·4 (3·5–5·5)**	**4·3 (2·8–5·7)**
**Central Europe, Eastern Europe, and Central Asia**	**Diarrheal diseases**	**56·4 (27·8–78·1)**	**17·7 (10·5–24·7)**	**40·7 (-1·8–74·2)**	**14·4 (10·0–18·3)**
**Central Europe, Eastern Europe, and Central Asia**	**Lower respiratory infections**	**43·5 (32·8–57·2)**	**24·9 (0·93–52·3)**	**15·3 (10·0–20·4)**	**17·5 (11·4–23·5)**
**Central Europe, Eastern Europe, and Central Asia**	**Malaria**	**0 (0–0)**	**0 (0–0)**	**--**	**0 (0–0)**
**Central Europe, Eastern Europe, and Central Asia**	**Measles**	**38·9 (18·9–48·4)**	**13·1 (6·2–17·5)**	**14·0 (5·3–25·6)**	**19·0 (8·7–25·4)**
Central Asia	All causes	13·3 (9·6–17·4)	7·5 (0·83–14·9)	5·3 (4·1–6·6)	5·1 (3·3–6·8)
Central Asia	Diarrheal diseases	62·5 (32·7–83·4)	20·7 (12·1–28·9)	45·5 (-2·0–80·3)	15·8 (10·7–20·2)
Central Asia	Lower respiratory infections	43·2 (32·6–56·6)	25·0 (0·95–51·9)	15·4 (10·1–20·7)	17·1 (11·1–23·0)
Central Asia	Malaria	0 (0–0)	0 (0–0)	--	0 (0–0)
Central Asia	Measles	39·4 (19·1–49·0)	13·3 (6·3–17·8)	14·3 (5·4–26·1)	19·2 (8·7–25·6)
Armenia	All causes	12·6 (9·3–16·4)	6·4 (0·47–13·8)	3·9 (2·7–5·2)	6·1 (3·9–8·2)
Armenia	Diarrheal diseases	46·4 (21·1–69·9)	11·2 (7·1–16·2)	31·3 (-1·1–65·1)	14·8 (10·3–19·1)
Armenia	Lower respiratory infections	45·2 (35·3–58·5)	23·7 (0·82–51·4)	12·8 (7·9–17·9)	22·3 (14·5–29·5)
Armenia	Malaria	0 (0–0)	0 (0–0)	--	0 (0–0)
Armenia	Measles	5·0 (-0·81–12·8)	0·11 (<0.1–0·27)	3·7 (-0·88–9·6)	1·3 (>-0.1–3·7)
Azerbaijan	All causes	15·0 (10·4–19·9)	8·6 (1·6–16·2)	6·3 (4·5–7·9)	5·7 (3·3–8·4)
Azerbaijan	Diarrheal diseases	60·1 (31·7–81·8)	20·2 (11·7–28·6)	42·8 (-1·6–79·0)	15·9 (10·3–21·5)
Azerbaijan	Lower respiratory infections	44·2 (32·8–56·7)	27·1 (1·1–52·4)	15·7 (10·0–21·2)	18·2 (10·3–25·5)
Azerbaijan	Malaria	0 (0–0)	0 (0–0)	--	0 (0–0)
Azerbaijan	Measles	41·3 (20·0–50·8)	14·3 (6·8–18·9)	14·7 (5·6–26·8)	20·2 (9·1–26·8)

Georgia	All causes	4·6 (3·0–7·0)	2·3 (0·13–5·6)	1·1 (0·70–1·5)	2·1 (1·6–2·8)
Georgia	Diarrheal diseases	22·1 (4·8–40·8)	5·7 (3·6–9·0)	12·7 (-0·67–30·4)	6·5 (1·6–11·6)
Georgia	Lower respiratory infections	30·0 (21·0–44·8)	15·1 (0·43–37·4)	6·3 (3·5–9·1)	14·0 (10·9–18·1)
Georgia	Malaria	0 (0–0)	0 (0–0)	--	0 (0–0)
Georgia	Measles	2·9 (>-0.1–7·0)	0·15 (<0.1–0·40)	2·0 (-0·36–5·0)	0·87 (<0.1–2·5)
Kazakhstan	All causes	7·3 (5·3–10·0)	4·1 (0·69–8·6)	2·8 (2·1–3·8)	2·7 (1·7–3·8)
Kazakhstan	Diarrheal diseases	47·0 (19·6–72·4)	11·4 (7·3–16·2)	33·4 (-1·2–67·1)	11·5 (8·2–15·0)
Kazakhstan	Lower respiratory infections	38·3 (28·2–52·0)	21·0 (0·69–46·7)	12·4 (7·7–17·3)	15·4 (9·7–21·2)
Kazakhstan	Malaria	0 (0–0)	0 (0–0)	--	0 (0–0)
Kazakhstan	Measles	22·6 (9·6–32·3)	5·8 (2·1–9·3)	8·1 (3·0–14·9)	11·4 (4·3–17·5)
Kyrgyzstan	All causes	8·2 (5·6–11·9)	4·3 (0·53–9·6)	2·6 (2·0–3·2)	3·6 (2·5–4·9)
Kyrgyzstan	Diarrheal diseases	49·0 (23·5–77·2)	11·5 (6·4–16·5)	32·8 (-0·90–72·8)	14·1 (9·4–18·3)
Kyrgyzstan	Lower respiratory infections	51·9 (38·7–72·0)	28·1 (0·90–65·1)	12·8 (7·9–17·5)	24·1 (15·9–32·0)
Kyrgyzstan	Malaria	0 (0–0)	0 (0–0)	--	0 (0–0)
Kyrgyzstan	Measles	31·1 (14·3–39·7)	8·7 (3·7–12·3)	8·4 (3·1–15·9)	18·1 (7·9–25·2)
Mongolia	All causes	6·6 (4·2–10·0)	3·3 (0·24–8·1)	1·3 (0·84–1·8)	3·2 (2·2–4·7)
Mongolia	Diarrheal diseases	36·2 (18·2–62·1)	7·9 (4·5–11·6)	22·1 (-0·53–56·8)	11·2 (7·4–15·0)
Mongolia	Lower respiratory infections	33·4 (24·5–48·6)	17·1 (0·49–40·8)	5·7 (3·3–8·0)	17·0 (11·7–22·2)
Mongolia	Malaria	0 (0–0)	0 (0–0)	--	0 (0–0)
Mongolia	Measles	0 (0–0)	0 (0–0)	0 (0–0)	0 (0–0)
Tajikistan	All causes	17·4 (12·4–22·3)	9·8 (2·3–15·8)	8·7 (5·8–11·2)	6·6 (4·7–8·8)
Tajikistan	Diarrheal diseases	71·0 (40·0–89·1)	25·9 (15·4–36·0)	52·2 (-2·8–86·2)	18·1 (12·2–23·1)
Tajikistan	Lower respiratory infections	67·2 (53·3–80·3)	42·3 (2·1–73·3)	26·7 (17·2–35·1)	29·3 (21·4–36·7)
Tajikistan	Malaria	0 (0–0)	0 (0–0)	--	0 (0–0)
Tajikistan	Measles	53·0 (28·1–64·0)	21·0 (10·4–27·2)	21·0 (8·5–36·4)	25·2 (11·7–32·1)
Turkmenistan	All causes	16·2 (11·3–21·2)	9·3 (0·59–18·5)	6·2 (4·3–8·2)	5·9 (4·3–7·9)
Turkmenistan	Diarrheal diseases	55·8 (25·0–78·9)	15·7 (9·1–22·5)	41·9 (-1·7–76·1)	11·0 (7·2–14·8)
Turkmenistan	Lower respiratory infections	51·2 (37·5–67·2)	30·1 (1·1–60·6)	18·4 (11·7–24·4)	19·2 (14·1–24·6)
Turkmenistan	Malaria	0 (0–0)	0 (0–0)	--	0 (0–0)

Turkmenistan	Measles	0 (0–0)	0 (0–0)	0 (0–0)	0 (0–0)
Uzbekistan	All causes	13·9 (10·0–18·4)	7·8 (0·40–16·8)	5·2 (3·4–7·0)	5·2 (3·3–7·2)
Uzbekistan	Diarrheal diseases	54·2 (23·4–78·8)	14·3 (8·5–20·3)	40·3 (-1·6–75·0)	12·0 (8·3–15·5)
Uzbekistan	Lower respiratory infections	39·3 (29·0–52·7)	22·3 (0·77–48·5)	14·3 (9·0–19·4)	14·9 (9·3–20·4)
Uzbekistan	Malaria	0 (0–0)	0 (0–0)	--	0 (0–0)
Uzbekistan	Measles	33·0 (14·7–44·0)	9·3 (4·0–13·0)	14·4 (5·2–26·5)	14·1 (6·2–19·5)
Central Europe	All causes	5·8 (3·8–8·5)	2·8 (0·53–5·9)	2·4 (1·6–3·1)	2·1 (1·4–2·9)
Central Europe	Diarrheal diseases	38·3 (13·2–62·8)	8·3 (5·7–11·5)	27·7 (-1·0–58·0)	8·9 (6·6–11·8)
Central Europe	Lower respiratory infections	47·7 (35·2–65·7)	26·1 (0·88–59·0)	13·9 (8·7–18·7)	20·2 (12·6–27·0)
Central Europe	Malaria	0 (0–0)	0 (0–0)	--	0 (0–0)
Central Europe	Measles	33·5 (16·3–41·6)	10·3 (4·7–14·4)	9·3 (3·4–17·4)	18·6 (8·7–24·8)
Albania	All causes	6·0 (3·8–8·9)	3·2 (0·62–6·9)	2·8 (1·9–3·9)	2·5 (1·3–4·0)
Albania	Diarrheal diseases	45·5 (15·3–69·2)	11·1 (8·3–16·3)	31·8 (-1·8–61·5)	14·7 (7·8–20·4)
Albania	Lower respiratory infections	57·5 (47·4–71·5)	29·3 (0·97–64·5)	21·8 (14·1–28·7)	27·7 (16·1–37·6)
Albania	Malaria	0 (0–0)	0 (0–0)	--	0 (0–0)
Albania	Measles	29·3 (0–55·8)	6·4 (0–12·8)	13·5 (0–34·8)	15·4 (0–30·3)
Bosnia and Herzegovina	All causes	2·4 (1·5–3·6)	1·0 (0·29–2·3)	1·1 (0·61–1·5)	0·99 (0·68–1·4)
Bosnia and Herzegovina	Diarrheal diseases	26·4 (7·8–42·5)	5·5 (3·5–8·2)	18·4 (-0·83–37·9)	7·8 (4·2–10·5)
Bosnia and Herzegovina	Lower respiratory infections	30·2 (21·2–43·0)	14·0 (0·40–35·1)	9·3 (5·4–13·2)	14·8 (10·0–19·4)
Bosnia and Herzegovina	Malaria	0 (0–0)	0 (0–0)	--	0 (0–0)
Bosnia and Herzegovina	Measles	22·1 (0–34·6)	3·7 (0–6·1)	7·5 (0–16·6)	13·1 (0–20·9)
Bulgaria	All causes	6·9 (4·3–11·1)	3·5 (0·54–8·2)	2·8 (2·1–3·5)	2·1 (1·4–3·0)
Bulgaria	Diarrheal diseases	38·9 (12·5–67·0)	7·9 (4·9–11·3)	29·4 (-1·1–63·5)	6·8 (4·7–9·1)
Bulgaria	Lower respiratory infections	41·9 (29·0–63·0)	22·4 (0·64–55·7)	13·2 (8·1–17·9)	14·3 (9·9–19·5)
Bulgaria	Malaria	0 (0–0)	0 (0–0)	--	0 (0–0)
Bulgaria	Measles	0 (0–0)	0 (0–0)	0 (0–0)	0 (0–0)
Croatia	All causes	3·9 (2·4–5·7)	1·7 (0·37–3·6)	1·8 (1·0–2·4)	1·4 (0·94–1·9)
Croatia	Diarrheal diseases	34·4 (10·8–56·8)	7·1 (5·3–10·2)	25·0 (-1·1–51·2)	8·0 (5·6–10·9)
Croatia	Lower respiratory infections	40·1 (30·2–55·9)	19·9 (0·57–48·6)	12·7 (7·9–17·5)	16·9 (12·0–22·2)

Croatia	Malaria	0 (0–0)	0 (0–0)	--	0 (0–0)
Croatia	Measles	0 (0–0)	0 (0–0)	0 (0–0)	0 (0–0)
Czechia	All causes	6·3 (3·5–9·8)	2·7 (1·1–5·2)	3·7 (1·6–5·7)	1·3 (0·93–1·9)
Czechia	Diarrheal diseases	38·6 (11·5–63·1)	8·8 (6·0–12·5)	30·0 (-1·2–59·4)	5·6 (4·2–7·6)
Czechia	Lower respiratory infections	39·6 (27·5–57·5)	21·6 (0·63–50·4)	15·0 (9·4–20·6)	11·7 (8·5–15·9)
Czechia	Malaria	0 (0–0)	0 (0–0)	--	0 (0–0)
Czechia	Measles	15·4 (6·1–25·2)	3·5 (1·3–6·3)	8·1 (3·1–15·7)	4·9 (1·7–8·5)
Hungary	All causes	4·4 (2·2–7·0)	1·3 (0·63–2·5)	2·6 (0·59–4·8)	1·3 (0·88–1·8)
Hungary	Diarrheal diseases	37·4 (12·3–62·3)	6·9 (4·5–9·7)	27·5 (-0·97–58·0)	9·1 (6·6–12·2)
Hungary	Lower respiratory infections	38·4 (28·9–53·8)	18·2 (0·49–45·6)	11·9 (7·3–16·3)	16·8 (11·3–22·2)
Hungary	Malaria	0 (0–0)	0 (0–0)	--	0 (0–0)
Hungary	Measles	0 (0–0)	0 (0–0)	0 (0–0)	0 (0–0)
Montenegro	All causes	3·7 (2·4–5·8)	1·9 (0·32–4·4)	1·3 (0·91–1·7)	1·5 (0·89–2·4)
Montenegro	Diarrheal diseases	33·2 (9·8–55·6)	7·8 (6·0–11·4)	20·9 (-0·94–46·0)	10·0 (5·0–14·1)
Montenegro	Lower respiratory infections	46·6 (33·9–66·1)	24·0 (0·68–59·9)	12·1 (7·3–16·8)	21·1 (12·2–29·2)
Montenegro	Malaria	0 (0–0)	0 (0–0)	--	0 (0–0)
Montenegro	Measles	0 (0–0)	0 (0–0)	0 (0–0)	0 (0–0)
North Macedonia	All causes	4·1 (2·2–5·8)	1·3 (0·45–2·9)	2·4 (0·96–3·6)	1·3 (0·83–1·9)
North Macedonia	Diarrheal diseases	39·3 (10·1–63·3)	5·8 (3·9–8·3)	30·4 (-1·3–59·1)	8·9 (6·1–11·8)
North Macedonia	Lower respiratory infections	42·1 (33·6–56·1)	18·0 (0·46–47·5)	15·7 (10·0–21·2)	17·7 (10·6–24·7)
North Macedonia	Malaria	0 (0–0)	0 (0–0)	--	0 (0–0)
North Macedonia	Measles	25·3 (11·5–31·9)	4·8 (2·3–6·4)	9·0 (3·3–16·5)	13·8 (6·3–18·6)
Poland	All causes	3·0 (1·8–4·5)	1·2 (0·30–2·5)	1·4 (0·73–2·2)	1·0 (0·68–1·4)
Poland	Diarrheal diseases	37·5 (12·9–61·7)	6·9 (4·1–9·9)	28·0 (-0·92–58·8)	8·5 (5·8–11·2)
Poland	Lower respiratory infections	42·3 (31·5–60·8)	20·8 (0·55–52·6)	12·9 (8·0–17·6)	18·0 (12·8–23·7)
Poland	Malaria	0 (0–0)	0 (0–0)	--	0 (0–0)
Poland	Measles	17·0 (6·9–24·3)	3·6 (1·4–5·5)	6·8 (2·9–13·0)	8·2 (3·2–12·8)
Romania	All causes	10·7 (7·9–15·4)	6·1 (0·75–12·6)	3·3 (2·5–4·3)	4·5 (2·8–6·4)
Romania	Diarrheal diseases	39·1 (17·7–60·9)	11·3 (8·0–15·5)	24·4 (-0·91–53·4)	11·8 (8·8–15·1)

Romania	Lower respiratory infections	54·1 (39·4–72·4)	31·7 (1·2–67·0)	13·8 (8·7–19·0)	24·3 (13·8–32·7)
Romania	Malaria	0 (0–0)	0 (0–0)	--	0 (0–0)
Romania	Measles	33·8 (16·4–41·9)	10·5 (4·8–14·7)	9·3 (3·4–17·5)	18·8 (8·8–25·1)
Serbia	All causes	2·9 (1·8–4·2)	1·4 (0·38–2·8)	1·3 (0·78–1·8)	1·1 (0·78–1·5)
Serbia	Diarrheal diseases	39·3 (13·6–61·5)	10·0 (7·6–13·8)	27·3 (-1·3–54·3)	10·5 (6·6–13·9)
Serbia	Lower respiratory infections	44·6 (33·8–61·1)	23·9 (0·85–53·2)	13·9 (8·6–19·1)	20·0 (13·8–26·8)
Serbia	Malaria	0 (0–0)	0 (0–0)	--	0 (0–0)
Serbia	Measles	28·4 (13·4–35·0)	7·4 (3·6–9·8)	8·2 (3·0–15·2)	16·1 (7·3–21·5)
Slovakia	All causes	7·8 (5·0–11·0)	3·5 (0·53–7·9)	3·3 (2·2–4·3)	2·8 (1·8–4·1)
Slovakia	Diarrheal diseases	44·7 (15·3–71·2)	9·1 (5·8–13·1)	33·2 (-1·1–67·0)	10·4 (7·2–13·5)
Slovakia	Lower respiratory infections	45·1 (33·9–62·9)	23·1 (0·68–56·4)	14·7 (9·0–20·2)	18·4 (11·5–25·2)
Slovakia	Malaria	0 (0–0)	0 (0–0)	--	0 (0–0)
Slovakia	Measles	0 (0–0)	0 (0–0)	0 (0–0)	0 (0–0)
Slovenia	All causes	4·4 (2·9–6·8)	2·0 (0·19–5·1)	1·7 (1·2–2·3)	1·6 (1·0–2·2)
Slovenia	Diarrheal diseases	23·7 (3·9–42·2)	5·0 (3·1–8·2)	16·3 (-1·2–34·8)	5·4 (1·9–9·2)
Slovenia	Lower respiratory infections	38·0 (28·2–54·9)	18·4 (0·46–47·6)	12·7 (8·0–17·5)	14·7 (10·0–20·0)
Slovenia	Malaria	0 (0–0)	0 (0–0)	--	0 (0–0)
Slovenia	Measles	0 (0–0)	0 (0–0)	0 (0–0)	0 (0–0)
Eastern Europe	All causes	4·2 (3·2–5·7)	2·2 (0·54–4·5)	1·6 (1·2–2·0)	1·8 (1·2–2·5)
Eastern Europe	Diarrheal diseases	37·8 (14·3–60·7)	9·0 (6·8–12·8)	24·7 (-1·1–53·0)	12·2 (8·2–16·5)
Eastern Europe	Lower respiratory infections	46·2 (35·5–61·9)	23·4 (0·70–55·2)	13·1 (8·0–18·0)	23·0 (14·9–30·4)
Eastern Europe	Malaria	0 (0–0)	0 (0–0)	--	0 (0–0)
Eastern Europe	Measles	8·2 (2·7–15·4)	1·4 (0·61–2·8)	4·1 (<0.1–9·3)	3·2 (1·4–6·7)
Belarus	All causes	2·1 (1·1–3·4)	1·3 (0·15–2·9)	0·64 (0·45–0·85)	0·61 (0·43–0·86)
Belarus	Diarrheal diseases	14·6 (<0.1–30·7)	6·4 (1·7–13·2)	6·6 (-0·98–16·2)	3·2 (-0·45–7·3)
Belarus	Lower respiratory infections	40·1 (24·9–61·7)	25·3 (0·77–57·6)	10·1 (6·2–14·0)	12·9 (7·9–18·2)
Belarus	Malaria	0 (0–0)	0 (0–0)	--	0 (0–0)
Belarus	Measles	17·3 (7·7–22·7)	7·4 (3·1–10·4)	4·6 (1·6–9·0)	6·5 (2·8–9·1)
Estonia	All causes	3·1 (1·7–5·0)	1·6 (0·10–3·9)	0·96 (0·65–1·3)	1·2 (0·86–1·6)

Estonia	Diarrheal diseases	12·9 (-2·2–28·5)	4·5 (0·60–10·0)	6·0 (-1·2–13·9)	3·7 (-1·4–9·3)
Estonia	Lower respiratory infections	34·8 (24·8–51·9)	18·3 (0·51–45·6)	9·4 (5·7–13·0)	14·2 (10·6–19·1)
Estonia	Malaria	0 (0–0)	0 (0–0)	--	0 (0–0)
Estonia	Measles	15·5 (5·5–24·1)	4·0 (1·4–8·1)	6·3 (2·2–11·6)	6·5 (2·2–11·6)
Latvia	All causes	3·2 (1·9–5·0)	1·7 (<0.1–4·0)	0·92 (0·61–1·3)	1·3 (0·88–1·8)
Latvia	Diarrheal diseases	15·2 (-1·5–32·3)	5·4 (1·1–11·5)	7·2 (-1·3–16·3)	4·3 (-1·1–10·3)
Latvia	Lower respiratory infections	35·1 (24·2–51·0)	19·0 (0·52–45·7)	9·2 (5·5–12·7)	14·4 (10·1–19·3)
Latvia	Malaria	0 (0–0)	0 (0–0)	--	0 (0–0)
Latvia	Measles	0 (0–0)	0 (0–0)	0 (0–0)	0 (0–0)
Lithuania	All causes	2·5 (1·3–4·1)	1·2 (0·12–2·8)	0·84 (0·53–1·1)	0·91 (0·64–1·3)
Lithuania	Diarrheal diseases	18·4 (1·2–36·3)	5·4 (2·2–10·1)	10·6 (-1·1–24·9)	4·7 (0·36–9·5)
Lithuania	Lower respiratory infections	32·1 (22·2–48·1)	17·0 (0·44–42·1)	8·6 (5·2–12·0)	13·0 (9·4–17·5)
Lithuania	Malaria	0 (0–0)	0 (0–0)	--	0 (0–0)
Lithuania	Measles	12·2 (2·3–27·8)	2·7 (0·23–7·8)	5·1 (0·70–12·5)	5·3 (0·71–14·8)
Republic of Moldova	All causes	4·9 (3·2–7·3)	2·6 (0·23–6·0)	1·6 (1·2–2·1)	1·8 (1·2–2·5)
Republic of Moldova	Diarrheal diseases	29·7 (7·6–51·7)	7·8 (5·9–11·3)	19·7 (-1·1–42·7)	6·7 (3·4–9·6)
Republic of Moldova	Lower respiratory infections	34·1 (23·7–50·5)	18·9 (0·60–45·4)	9·7 (5·8–13·7)	13·1 (8·8–18·0)
Republic of Moldova	Malaria	0 (0–0)	0 (0–0)	--	0 (0–0)
Republic of Moldova	Measles	22·1 (9·9–27·9)	6·2 (2·9–8·3)	7·4 (2·8–13·7)	10·5 (4·6–14·4)
Russian Federation	All causes	4·4 (3·2–6·1)	2·1 (0·31–4·8)	1·6 (1·2–2·0)	2·1 (1·3–2·9)
Russian Federation	Diarrheal diseases	42·1 (16·7–66·4)	9·3 (6·5–12·9)	28·3 (-1·1–60·3)	13·8 (10·2–18·1)
Russian Federation	Lower respiratory infections	48·6 (37·9–64·6)	24·1 (0·70–57·3)	14·2 (8·7–19·5)	24·5 (15·7–32·5)
Russian Federation	Malaria	0 (0–0)	0 (0–0)	--	0 (0–0)
Russian Federation	Measles	5·4 (-0·56–13·6)	0·21 (<0.1–0·40)	3·8 (-0·72–9·5)	1·6 (<0.1–4·4)
Ukraine	All causes	3·7 (2·9–4·8)	2·6 (1·4–4·2)	2·0 (1·5–2·6)	1·2 (0·83–1·6)
Ukraine	Diarrheal diseases	37·1 (15·7–58·6)	11·3 (8·4–16·0)	21·4 (-0·96–49·4)	13·4 (7·8–18·4)
Ukraine	Lower respiratory infections	39·4 (29·8–52·5)	21·3 (0·72–45·8)	8·7 (5·1–12·1)	20·8 (13·8–27·0)
Ukraine	Malaria	0 (0–0)	0 (0–0)	--	0 (0–0)
Ukraine	Measles	34·2 (16·0–43·0)	8·0 (3·1–12·3)	8·0 (3·0–15·2)	22·7 (10·3–29·9)

**High-income**	**All causes**	**0·98 (0·62–1·6)**	**0·51 (0·17–1·1)**	**0·42 (0·30–0·57)**	**0·30 (0·23–0·41)**
**High-income**	**Diarrheal diseases**	**18·4 (3·9–35·5)**	**4·4 (2·8–6·9)**	**11·9 (-0·69–29·1)**	**3·9 (1·7–6·1)**
**High-income**	**Lower respiratory infections**	**34·6 (24·4–54·5)**	**17·6 (0·44–47·2)**	**8·8 (5·1–12·2)**	**13·6 (9·9–17·7)**
**High-income**	**Malaria**	**8·3 (-3·4–29·8)**	**8·3 (-3·4–29·8)**	**--**	**<0.1 (>-0.1–<0.1)**
**High-income**	**Measles**	**13·6 (6·0–17·7)**	**3·8 (1·8–5·2)**	**5·4 (1·9–10·0)**	**5·2 (2·4–7·2)**
Australasia	All causes	0·44 (0·24–0·81)	0·15 (<0.1–0·42)	0·21 (0·12–0·35)	0·14 (0·11–0·19)
Australasia	Diarrheal diseases	10·4 (-0·47–23·8)	1·5 (0·49–2·9)	7·2 (-0·83–19·0)	2·3 (>-0.1–5·1)
Australasia	Lower respiratory infections	19·6 (15·3–31·2)	7·4 (0·15–24·9)	6·5 (3·8–9·1)	7·5 (5·1–10·6)
Australasia	Malaria	0 (0–0)	0 (0–0)	--	0 (0–0)
Australasia	Measles	9·4 (4·1–13·1)	1·2 (0·56–1·6)	4·4 (1·6–8·5)	4·0 (1·8–5·5)
Australia	All causes	0·37 (0·20–0·68)	0·12 (<0.1–0·32)	0·20 (0·11–0·32)	0·11 (<0.1–0·15)
Australia	Diarrheal diseases	9·2 (-0·97–21·4)	1·2 (0·26–2·6)	6·3 (-0·84–16·7)	2·0 (-0·35–4·8)
Australia	Lower respiratory infections	18·1 (14·8–27·7)	5·9 (0·10–20·8)	6·6 (3·9–9·3)	7·0 (5·1–9·6)
Australia	Malaria	0 (0–0)	0 (0–0)	--	0 (0–0)
Australia	Measles	9·3 (4·0–13·0)	1·1 (0·52–1·5)	4·4 (1·6–8·5)	4·0 (1·8–5·4)
New Zealand	All causes	0·75 (0·41–1·3)	0·29 (<0.1–0·85)	0·28 (0·15–0·47)	0·25 (0·17–0·36)
New Zealand	Diarrheal diseases	16·5 (2·5–35·4)	2·8 (1·7–4·4)	11·3 (-0·79–30·0)	3·6 (1·3–6·1)
New Zealand	Lower respiratory infections	22·9 (16·6–38·6)	10·9 (0·24–33·9)	6·2 (3·7–8·7)	8·7 (5·0–12·7)
New Zealand	Malaria	0 (0–0)	0 (0–0)	--	0 (0–0)
New Zealand	Measles	10·6 (4·4–14·4)	1·9 (0·86–2·7)	4·2 (1·6–8·1)	4·8 (2·1–6·7)
High-income Asia Pacific	All causes	1·2 (0·63–2·0)	0·64 (0·17–1·4)	0·50 (0·34–0·69)	0·32 (0·23–0·43)
High-income Asia Pacific	Diarrheal diseases	18·6 (1·5–36·4)	6·0 (2·3–11·2)	10·8 (-1·1–25·0)	3·7 (0·50–7·3)
High-income Asia Pacific	Lower respiratory infections	39·6 (26·5–62·4)	22·4 (0·57–56·1)	10·5 (6·2–14·6)	13·8 (10·4–18·2)
High-income Asia Pacific	Malaria	8·3 (-3·4–29·8)	8·3 (-3·4–29·8)	--	<0.1 (>-0.1–<0.1)
High-income Asia Pacific	Measles	21·9 (10·0–29·1)	7·4 (3·4–10·0)	8·3 (3·0–15·6)	8·1 (3·6–11·1)
Brunei Darussalam	All causes	1·7 (1·2–2·5)	0·86 (0·14–2·0)	0·51 (0·37–0·68)	0·92 (0·56–1·4)
Brunei Darussalam	Diarrheal diseases	42·8 (20·6–63·3)	10·5 (7·0–15·0)	25·7 (-0·85–56·8)	17·6 (12·6–22·9)
Brunei Darussalam	Lower respiratory infections	51·1 (40·4–64·9)	26·1 (0·84–57·1)	11·4 (6·9–15·8)	29·2 (19·6–36·7)
Brunei Darussalam	Malaria	0 (0–0)	0 (0–0)	--	0 (0–0)

Brunei Darussalam	Measles	0 (0–0)	0 (0–0)	0 (0–0)	0 (0–0)
Japan	All causes	1·3 (0·67–2·2)	0·72 (0·20–1·5)	0·54 (0·36–0·76)	0·35 (0·25–0·47)
Japan	Diarrheal diseases	17·2 (0·47–34·8)	6·0 (1·8–11·9)	9·3 (-1·2–21·3)	3·7 (0·13–7·7)
Japan	Lower respiratory infections	40·3 (27·2–63·4)	22·8 (0·58–57·4)	10·4 (6·2–14·5)	14·4 (10·6–19·0)
Japan	Malaria	0 (0–0)	0 (0–0)	--	0 (0–0)
Japan	Measles	21·9 (9·9–29·0)	7·4 (3·4–10·1)	8·2 (3·0–15·5)	8·2 (3·7–11·1)
Republic of Korea	All causes	0·72 (0·37–1·2)	0·34 (<0.1–0·75)	0·34 (0·18–0·55)	0·15 (0·11–0·21)
Republic of Korea	Diarrheal diseases	25·0 (5·9–48·2)	5·8 (4·4–8·2)	18·3 (-0·91–43·7)	3·1 (1·8–4·4)
Republic of Korea	Lower respiratory infections	33·5 (20·9–56·2)	19·2 (0·48–50·5)	9·7 (5·6–13·8)	9·2 (7·2–11·7)
Republic of Korea	Malaria	8·3 (-3·4–29·8)	8·3 (-3·4–29·8)	--	<0.1 (>-0.1–<0.1)
Republic of Korea	Measles	15·5 (6·0–21·9)	4·8 (1·9–6·5)	7·4 (2·6–14·0)	4·3 (1·5–6·0)
Singapore	All causes	2·0 (1·2–3·3)	1·1 (0·12–2·7)	0·73 (0·42–1·0)	0·61 (0·33–0·97)
Singapore	Diarrheal diseases	36·4 (13·2–61·0)	9·2 (6·0–13·1)	26·4 (-0·92–56·9)	6·4 (4·6–8·5)
Singapore	Lower respiratory infections	42·3 (28·1–63·3)	24·7 (0·65–58·2)	12·8 (7·9–17·3)	13·5 (8·6–18·7)
Singapore	Malaria	0 (0–0)	0 (0–0)	--	0 (0–0)
Singapore	Measles	25·1 (11·3–33·4)	8·3 (3·7–11·6)	10·8 (4·1–20·5)	8·8 (4·1–12·2)
High-income North America	All causes	0·89 (0·59–1·4)	0·46 (0·14–1·0)	0·40 (0·32–0·48)	0·26 (0·19–0·35)
High-income North America	Diarrheal diseases	29·6 (8·5–55·7)	5·1 (3·3–7·3)	22·5 (-0·66–53·3)	5·0 (3·5–6·6)
High-income North America	Lower respiratory infections	37·1 (26·9–58·2)	18·3 (0·45–50·0)	11·1 (6·5–15·4)	13·4 (9·7–17·7)
High-income North America	Malaria	0 (0–0)	0 (0–0)	--	0 (0–0)
High-income North America	Measles	17·4 (7·6–24·3)	4·3 (1·9–5·8)	8·1 (2·9–15·3)	6·3 (2·8–8·7)
Canada	All causes	0·66 (0·30–1·2)	0·32 (<0.1–0·75)	0·36 (0·19–0·60)	<0.1 (<0.1–0·10)
Canada	Diarrheal diseases	26·4 (6·0–53·7)	5·3 (3·3–7·8)	21·2 (-0·64–51·8)	1·7 (1·2–2·4)
Canada	Lower respiratory infections	26·9 (15·1–50·9)	16·3 (0·37–46·6)	9·0 (5·3–12·6)	4·0 (3·2–5·4)
Canada	Malaria	0 (0–0)	0 (0–0)	--	0 (0–0)
Canada	Measles	11·0 (4·5–17·3)	2·7 (1·2–3·8)	7·0 (2·6–13·1)	1·7 (0·71–2·4)
Greenland	All causes	2·0 (1·1–3·3)	1·0 (0·23–2·3)	0·72 (0·48–0·98)	0·62 (0·42–0·93)
Greenland	Diarrheal diseases	38·1 (15·4–67·2)	8·4 (4·4–12·5)	27·0 (-0·57–64·6)	7·7 (4·6–10·4)
Greenland	Lower respiratory infections	40·5 (27·0–62·7)	22·5 (0·62–55·9)	10·0 (5·9–14·1)	14·6 (10·0–19·3)

Greenland	Malaria	0 (0–0)	0 (0–0)	--	0 (0–0)
Greenland	Measles	18·3 (8·3–24·9)	4·8 (2·3–6·4)	7·8 (2·7–14·8)	7·0 (3·2–9·6)
United States of America	All causes	0·91 (0·61–1·4)	0·47 (0·15–1·1)	0·41 (0·33–0·49)	0·27 (0·20–0·37)
United States of America	Diarrheal diseases	30·2 (8·9–56·3)	5·1 (3·3–7·2)	22·8 (-0·67–53·6)	5·7 (3·9–7·3)
United States of America	Lower respiratory infections	37·7 (27·7–58·7)	18·4 (0·45–50·2)	11·3 (6·6–15·6)	14·0 (10·1–18·5)
United States of America	Malaria	0 (0–0)	0 (0–0)	--	0 (0–0)
United States of America	Measles	18·8 (8·2–26·8)	3·6 (1·7–4·8)	9·8 (3·4–18·6)	6·7 (3·1–9·1)
Southern Latin America	All causes	2·3 (1·6–3·4)	1·3 (0·52–2·6)	0·89 (0·69–1·1)	0·90 (0·66–1·2)
Southern Latin America	Diarrheal diseases	25·7 (9·7–46·3)	6·3 (5·1–8·9)	14·7 (-0·63–38·1)	8·0 (5·1–10·4)
Southern Latin America	Lower respiratory infections	37·2 (27·0–54·5)	18·7 (0·50–46·4)	6·5 (3·7–9·1)	18·8 (14·1–24·1)
Southern Latin America	Malaria	0 (0–0)	0 (0–0)	--	0 (0–0)
Southern Latin America	Measles	13·6 (6·2–17·2)	5·7 (2·6–7·6)	4·2 (1·5–7·8)	4·5 (2·0–6·1)
Argentina	All causes	2·5 (1·7–3·6)	1·3 (0·44–2·7)	0·86 (0·68–1·1)	1·1 (0·77–1·4)
Argentina	Diarrheal diseases	26·9 (10·3–47·5)	6·0 (4·8–8·6)	15·2 (-0·65–39·1)	9·4 (6·0–12·4)
Argentina	Lower respiratory infections	39·1 (29·1–55·8)	18·9 (0·51–46·9)	6·8 (3·9–9·5)	20·9 (15·4–26·5)
Argentina	Malaria	0 (0–0)	0 (0–0)	--	0 (0–0)
Argentina	Measles	0 (0–0)	0 (0–0)	0 (0–0)	0 (0–0)
Chile	All causes	1·8 (1·1–2·8)	1·2 (0·66–2·1)	0·95 (0·70–1·3)	0·33 (0·26–0·43)
Chile	Diarrheal diseases	21·5 (7·0–42·3)	6·6 (5·2–9·1)	13·3 (-0·59–35·0)	3·6 (2·1–5·2)
Chile	Lower respiratory infections	27·0 (15·1–47·3)	17·1 (0·45–42·3)	5·0 (2·8–7·3)	8·5 (7·0–11·0)
Chile	Malaria	0 (0–0)	0 (0–0)	--	0 (0–0)
Chile	Measles	13·6 (6·2–17·2)	5·7 (2·6–7·6)	4·2 (1·5–7·8)	4·5 (2·0–6·1)
Uruguay	All causes	2·4 (1·6–3·6)	1·4 (0·64–2·5)	0·96 (0·59–1·5)	0·90 (0·65–1·2)
Uruguay	Diarrheal diseases	30·1 (13·2–49·7)	9·2 (7·2–13·0)	15·5 (-0·61–40·3)	10·5 (6·7–13·7)
Uruguay	Lower respiratory infections	42·1 (29·5–61·8)	24·1 (0·69–56·8)	6·6 (3·8–9·2)	20·5 (13·5–27·0)
Uruguay	Malaria	0 (0–0)	0 (0–0)	--	0 (0–0)
Uruguay	Measles	0 (0–0)	0 (0–0)	0 (0–0)	0 (0–0)
Western Europe	All causes	0·63 (0·31–1·2)	0·31 (<0.1–0·72)	0·28 (0·14–0·49)	0·16 (0·12–0·21)
Western Europe	Diarrheal diseases	12·7 (1·4–26·6)	3·4 (1·6–6·0)	7·8 (-0·61–19·5)	2·5 (0·42–4·8)

Western Europe	Lower respiratory infections	26·2 (16·6–46·6)	14·5 (0·33–41·5)	6·3 (3·7–8·8)	8·4 (5·7–11·4)
Western Europe	Malaria	0 (0–0)	0 (0–0)	--	0 (0–0)
Western Europe	Measles	10·2 (4·4–12·9)	3·0 (1·4–4·2)	3·4 (1·2–6·3)	4·2 (1·9–5·8)
Andorra	All causes	0·56 (0·26–1·1)	0·29 (<0.1–0·81)	0·16 (0·10–0·24)	0·17 (0·11–0·27)
Andorra	Diarrheal diseases	6·2 (-1·5–15·4)	2·2 (>-0.1–5·1)	2·8 (-0·61–7·7)	1·6 (-0·88–4·4)
Andorra	Lower respiratory infections	26·1 (16·9–46·5)	14·1 (0·31–41·1)	6·3 (3·7–9·0)	8·5 (6·0–11·5)
Andorra	Malaria	0 (0–0)	0 (0–0)	--	0 (0–0)
Andorra	Measles	0 (0–0)	0 (0–0)	0 (0–0)	0 (0–0)
Austria	All causes	0·31 (<0.1–0·65)	0·13 (<0.1–0·33)	0·11 (<0.1–0·23)	<0.1 (<0.1–0·15)
Austria	Diarrheal diseases	7·8 (-1·1–18·3)	2·6 (0·33–5·7)	3·7 (-0·64–9·6)	1·9 (-0·74–5·0)
Austria	Lower respiratory infections	26·4 (16·9–45·3)	14·0 (0·30–38·6)	6·0 (3·4–8·5)	9·4 (7·7–12·2)
Austria	Malaria	0 (0–0)	0 (0–0)	--	0 (0–0)
Austria	Measles	1·8 (-0·36–4·7)	<0.1 (<0.1–<0.1)	1·7 (-0·46–4·3)	0·16 (<0.1–0·39)
Belgium	All causes	0·92 (0·39–1·7)	0·39 (0·10–0·93)	0·41 (0·15–0·82)	0·24 (0·18–0·32)
Belgium	Diarrheal diseases	15·0 (2·7–30·0)	3·5 (2·0–5·6)	9·8 (-0·60–24·3)	2·9 (1·0–5·0)
Belgium	Lower respiratory infections	28·4 (18·4–50·2)	15·6 (0·36–45·1)	6·8 (4·1–9·5)	9·3 (6·3–12·8)
Belgium	Malaria	0 (0–0)	0 (0–0)	--	0 (0–0)
Belgium	Measles	2·2 (<0.1–5·3)	0·18 (<0.1–0·39)	1·7 (-0·30–4·3)	0·35 (0·11–0·73)
Cyprus	All causes	0·91 (0·32–1·9)	0·36 (0·12–0·75)	0·46 (<0.1–1·2)	0·22 (0·13–0·32)
Cyprus	Diarrheal diseases	19·0 (3·6–38·1)	5·0 (3·1–7·8)	12·1 (-0·69–31·7)	3·8 (1·3–6·1)
Cyprus	Lower respiratory infections	34·6 (21·7–58·1)	19·8 (0·50–52·2)	8·4 (5·0–11·9)	11·4 (6·8–16·1)
Cyprus	Malaria	0 (0–0)	0 (0–0)	--	0 (0–0)
Cyprus	Measles	0 (0–0)	0 (0–0)	0 (0–0)	0 (0–0)
Denmark	All causes	1·0 (0·45–1·9)	0·43 (0·13–0·95)	0·51 (0·16–1·1)	0·25 (0·17–0·36)
Denmark	Diarrheal diseases	18·6 (4·4–37·5)	4·3 (3·1–6·4)	12·5 (-0·63–32·1)	3·5 (1·6–5·4)
Denmark	Lower respiratory infections	33·0 (20·8–56·1)	18·7 (0·45–51·5)	8·5 (5·1–11·8)	10·1 (5·2–14·9)
Denmark	Malaria	0 (0–0)	0 (0–0)	--	0 (0–0)
Denmark	Measles	0 (0–0)	0 (0–0)	0 (0–0)	0 (0–0)
Finland	All causes	0·56 (0·24–1·1)	0·27 (<0.1–0·69)	0·20 (<0.1–0·31)	0·16 (0·12–0·21)

Finland	Diarrheal diseases	10·4 (>-0.1–22·9)	3·2 (0·93–6·3)	5·7 (-0·69–14·9)	2·3 (-0·25–5·2)
Finland	Lower respiratory infections	29·6 (19·2–50·7)	16·3 (0·40–45·0)	7·1 (4·2–9·9)	9·9 (7·1–13·2)
Finland	Malaria	0 (0–0)	0 (0–0)	--	0 (0–0)
Finland	Measles	0 (0–0)	0 (0–0)	0 (0–0)	0 (0–0)
France	All causes	0·75 (0·43–1·3)	0·43 (0·22–0·82)	0·40 (0·26–0·62)	0·16 (0·12–0·20)
France	Diarrheal diseases	11·1 (0·72–23·6)	3·0 (1·3–5·5)	6·6 (-0·62–16·9)	2·3 (0·14–4·8)
France	Lower respiratory infections	26·4 (16·9–45·9)	14·4 (0·32–40·7)	6·4 (3·7–9·1)	8·7 (6·0–11·9)
France	Malaria	0 (0–0)	0 (0–0)	--	0 (0–0)
France	Measles	7·7 (3·0–10·8)	2·1 (0·70–3·2)	2·8 (1·1–5·2)	3·0 (1·1–4·9)
Germany	All causes	0·50 (0·16–0·95)	0·21 (<0.1–0·46)	0·25 (<0.1–0·54)	0·10 (<0.1–0·13)
Germany	Diarrheal diseases	13·5 (1·8–27·2)	3·9 (1·8–6·9)	8·4 (-0·65–20·4)	2·3 (0·52–4·2)
Germany	Lower respiratory infections	29·1 (17·7–50·3)	17·1 (0·37–46·1)	7·6 (4·4–10·6)	8·3 (5·3–11·7)
Germany	Malaria	0 (0–0)	0 (0–0)	--	0 (0–0)
Germany	Measles	10·1 (4·2–13·5)	3·4 (1·4–4·7)	3·7 (1·4–7·0)	3·4 (1·6–4·8)
Greece	All causes	0·68 (0·34–1·3)	0·36 (<0.1–1·0)	0·18 (0·13–0·24)	0·22 (0·16–0·32)
Greece	Diarrheal diseases	7·8 (-1·3–17·8)	2·7 (0·29–6·1)	3·4 (-0·65–8·8)	2·1 (-0·94–5·6)
Greece	Lower respiratory infections	32·1 (20·7–54·5)	17·8 (0·42–49·6)	7·7 (4·6–10·8)	10·7 (6·0–15·3)
Greece	Malaria	0 (0–0)	0 (0–0)	--	0 (0–0)
Greece	Measles	0 (0–0)	0 (0–0)	0 (0–0)	0 (0–0)
Iceland	All causes	0·76 (0·36–1·4)	0·36 (<0.1–0·98)	0·29 (0·16–0·46)	0·21 (0·16–0·28)
Iceland	Diarrheal diseases	11·7 (0·77–24·2)	3·0 (1·3–5·5)	7·1 (-0·63–18·4)	2·4 (0·19–4·8)
Iceland	Lower respiratory infections	28·3 (18·3–50·3)	15·5 (0·36–45·5)	7·1 (4·2–10·0)	8·9 (5·5–12·7)
Iceland	Malaria	0 (0–0)	0 (0–0)	--	0 (0–0)
Iceland	Measles	0 (0–0)	0 (0–0)	0 (0–0)	0 (0–0)
Ireland	All causes	0·70 (0·32–1·4)	0·35 (<0.1–1·0)	0·23 (0·14–0·33)	0·20 (0·14–0·28)
Ireland	Diarrheal diseases	8·4 (-0·47–18·0)	2·4 (0·58–4·9)	4·6 (-0·62–11·9)	1·9 (-0·39–4·4)
Ireland	Lower respiratory infections	25·7 (16·1–46·3)	14·1 (0·30–42·3)	6·6 (3·9–9·3)	7·9 (4·6–11·4)
Ireland	Malaria	0 (0–0)	0 (0–0)	--	0 (0–0)
Ireland	Measles	0 (0–0)	0 (0–0)	0 (0–0)	0 (0–0)

Israel	All causes	0·96 (0·39–1·8)	0·38 (0·11–0·89)	0·46 (0·14–0·95)	0·24 (0·18–0·32)
Israel	Diarrheal diseases	14·7 (2·7–29·0)	3·3 (2·0–5·3)	9·6 (-0·57–23·4)	2·9 (0·98–4·8)
Israel	Lower respiratory infections	28·9 (18·5–51·9)	15·8 (0·35–46·9)	6·9 (4·1–9·7)	9·4 (5·9–13·0)
Israel	Malaria	0 (0–0)	0 (0–0)	--	0 (0–0)
Israel	Measles	9·4 (4·2–11·9)	2·5 (1·1–3·4)	3·1 (1·1–5·7)	4·2 (1·9–5·8)
Italy	All causes	0·71 (0·36–1·3)	0·35 (<0.1–0·90)	0·27 (0·16–0·43)	0·20 (0·14–0·27)
Italy	Diarrheal diseases	13·1 (1·6–27·4)	3·2 (1·7–5·4)	8·3 (-0·58–21·2)	2·6 (0·58–4·8)
Italy	Lower respiratory infections	27·6 (17·7–48·5)	15·1 (0·33–43·4)	6·7 (4·0–9·4)	9·0 (5·9–12·6)
Italy	Malaria	0 (0–0)	0 (0–0)	--	0 (0–0)
Italy	Measles	9·4 (4·0–12·2)	2·7 (1·2–3·8)	3·2 (1·2–6·1)	3·8 (1·7–5·5)
Luxembourg	All causes	0·61 (0·26–1·1)	0·27 (0·10–0·59)	0·30 (0·11–0·60)	0·14 (0·10–0·18)
Luxembourg	Diarrheal diseases	13·8 (1·9–29·1)	3·6 (1·9–6·1)	8·6 (-0·65–22·8)	2·7 (0·63–5·0)
Luxembourg	Lower respiratory infections	26·7 (16·9–46·6)	15·1 (0·35–42·3)	6·6 (3·8–9·5)	8·4 (5·4–12·0)
Luxembourg	Malaria	0 (0–0)	0 (0–0)	--	0 (0–0)
Luxembourg	Measles	0 (0–0)	0 (0–0)	0 (0–0)	0 (0–0)
Malta	All causes	0·64 (0·31–1·2)	0·34 (<0.1–0·94)	0·19 (0·14–0·24)	0·19 (0·15–0·27)
Malta	Diarrheal diseases	8·4 (-0·89–18·7)	3·0 (0·46–6·4)	4·0 (-0·67–9·9)	2·1 (-0·68–5·3)
Malta	Lower respiratory infections	28·2 (17·8–48·6)	16·0 (0·39–43·9)	7·0 (4·1–9·8)	9·0 (5·4–12·9)
Malta	Malaria	0 (0–0)	0 (0–0)	--	0 (0–0)
Malta	Measles	0 (0–0)	0 (0–0)	0 (0–0)	0 (0–0)
Monaco	All causes	0·65 (0·27–1·4)	0·34 (<0.1–1·1)	0·18 (0·12–0·28)	0·20 (0·12–0·32)
Monaco	Diarrheal diseases	6·0 (-1·3–14·7)	1·9 (<0.1–4·4)	2·8 (-0·58–7·9)	1·5 (-0·79–4·2)
Monaco	Lower respiratory infections	26·9 (17·5–49·4)	14·5 (0·32–44·8)	6·8 (4·0–9·6)	8·5 (5·0–12·2)
Monaco	Malaria	0 (0–0)	0 (0–0)	--	0 (0–0)
Monaco	Measles	0 (0–0)	0 (0–0)	0 (0–0)	0 (0–0)
Netherlands	All causes	0·54 (0·26–1·0)	0·35 (0·11–0·75)	0·19 (0·11–0·32)	0·12 (<0.1–0·17)
Netherlands	Diarrheal diseases	15·7 (5·3–30·1)	5·5 (4·1–7·8)	8·7 (-0·44–24·4)	2·7 (1·4–4·0)
Netherlands	Lower respiratory infections	26·4 (13·1–50·2)	17·8 (0·47–46·8)	3·8 (2·1–5·5)	7·1 (4·9–9·9)
Netherlands	Malaria	0 (0–0)	0 (0–0)	--	0 (0–0)

Netherlands	Measles	8·0 (3·4–10·7)	2·8 (1·2–4·1)	2·4 (0·85–4·9)	3·1 (1·4–4·3)
Norway	All causes	0·39 (0·17–0·75)	0·19 (<0.1–0·45)	0·17 (<0.1–0·30)	<0.1 (<0.1–0·13)
Norway	Diarrheal diseases	9·5 (0·29–22·0)	2·5 (0·74–4·9)	5·5 (-0·62–15·1)	2·0 (>-0.1–4·3)
Norway	Lower respiratory infections	22·6 (14·5–41·7)	12·3 (0·25–37·0)	5·6 (3·3–7·9)	7·2 (4·9–9·9)
Norway	Malaria	0 (0–0)	0 (0–0)	--	0 (0–0)
Norway	Measles	11·2 (4·8–14·6)	3·4 (1·5–4·6)	3·7 (1·3–7·4)	4·5 (2·0–6·2)
Portugal	All causes	0·90 (0·49–1·6)	0·48 (<0.1–1·2)	0·31 (0·21–0·44)	0·26 (0·19–0·37)
Portugal	Diarrheal diseases	14·2 (2·0–29·3)	4·0 (2·2–6·9)	8·5 (-0·64–21·8)	3·0 (0·67–5·6)
Portugal	Lower respiratory infections	32·9 (20·9–56·6)	18·6 (0·46–50·9)	7·7 (4·6–10·8)	11·0 (7·5–15·0)
Portugal	Malaria	0 (0–0)	0 (0–0)	--	0 (0–0)
Portugal	Measles	12·7 (5·8–16·0)	4·2 (1·9–5·6)	3·8 (1·4–7·2)	5·4 (2·4–7·3)
San Marino	All causes	0·47 (0·19–0·93)	0·25 (<0.1–0·70)	0·14 (<0.1–0·21)	0·14 (<0.1–0·25)
San Marino	Diarrheal diseases	6·3 (-1·6–15·5)	2·2 (<0.1–5·2)	2·7 (-0·63–7·5)	1·6 (-0·93–4·7)
San Marino	Lower respiratory infections	25·7 (15·8–45·0)	14·1 (0·31–40·5)	6·4 (3·8–9·1)	8·2 (4·9–11·9)
San Marino	Malaria	0 (0–0)	0 (0–0)	--	0 (0–0)
San Marino	Measles	0 (0–0)	0 (0–0)	0 (0–0)	0 (0–0)
Spain	All causes	0·67 (0·30–1·3)	0·31 (<0.1–0·80)	0·27 (0·12–0·49)	0·18 (0·13–0·25)
Spain	Diarrheal diseases	12·9 (1·4–27·2)	3·4 (1·6–6·0)	7·9 (-0·63–20·7)	2·6 (0·45–5·1)
Spain	Lower respiratory infections	30·2 (19·5–53·2)	16·9 (0·39–48·1)	7·4 (4·3–10·3)	9·7 (6·0–13·9)
Spain	Malaria	0 (0–0)	0 (0–0)	--	0 (0–0)
Spain	Measles	5·0 (1·6–7·9)	1·1 (0·36–2·1)	2·3 (0·56–4·8)	1·7 (0·49–3·0)
Sweden	All causes	0·68 (0·27–1·3)	0·28 (<0.1–0·68)	0·32 (0·10–0·65)	0·17 (0·12–0·22)
Sweden	Diarrheal diseases	13·5 (1·8–28·5)	3·2 (1·7–5·2)	8·8 (-0·61–22·8)	2·5 (0·68–4·5)
Sweden	Lower respiratory infections	27·4 (17·4–49·6)	14·9 (0·32–44·1)	6·7 (3·9–9·3)	8·9 (6·2–12·0)
Sweden	Malaria	0 (0–0)	0 (0–0)	--	0 (0–0)
Sweden	Measles	9·4 (4·1–12·1)	2·6 (1·2–3·7)	3·2 (1·2–5·9)	3·9 (1·8–5·6)
Switzerland	All causes	0·50 (0·18–0·97)	0·22 (<0.1–0·53)	0·23 (<0.1–0·43)	0·12 (<0.1–0·17)
Switzerland	Diarrheal diseases	10·4 (0·51–22·3)	2·8 (1·1–5·4)	6·2 (-0·65–15·5)	2·1 (<0.1–4·6)
Switzerland	Lower respiratory infections	25·4 (16·0–45·6)	13·9 (0·30–40·8)	6·3 (3·7–8·8)	8·1 (5·4–11·1)

Switzerland	Malaria	0<br>(0–0)	0<br>(0–0)	--	0<br>(0–0)
Switzerland	Measles	2·2<br>(>-0.1–4·9)	0·15<br>(<0.1–0·34)	1·7<br>(-0·30–4·3)	0·31<br>(<0.1–0·66)
United Kingdom	All causes	0·49<br>(0·24–0·96)	0·23<br>(<0.1–0·64)	0·17<br>(0·11–0·26)	0·14<br>(0·11–0·19)
United Kingdom	Diarrheal diseases	11·9<br>(0·25–27·1)	2·9<br>(0·98–6·0)	7·1<br>(-0·58–20·3)	2·4<br>(>-0.1–5·5)
United Kingdom	Lower respiratory infections	20·5<br>(13·2–38·2)	10·6<br>(0·23–33·1)	4·9<br>(2·9–7·0)	6·8<br>(5·3–9·1)
United Kingdom	Malaria	0<br>(0–0)	0<br>(0–0)	--	0<br>(0–0)
United Kingdom	Measles	10·8<br>(4·7–14·0)	2·8<br>(1·3–3·9)	3·9<br>(1·4–7·6)	4·5<br>(2·0–6·3)
**Latin America and Caribbean**	**All causes**	**9·2<br>(6·9–11·6)**	**5·8<br>(2·7–9·1)**	**3·9<br>(2·8–5·0)**	**3·5<br>(2·6–4·5)**
**Latin America and Caribbean**	**Diarrheal diseases**	**55·0<br>(36·4–73·7)**	**20·2<br>(12·1–28·7)**	**31·3<br>(-1·1–66·5)**	**19·6<br>(13·7–24·7)**
**Latin America and Caribbean**	**Lower respiratory infections**	**52·2<br>(38·7–68·7)**	**31·3<br>(1·3–63·2)**	**10·0<br>(6·0–13·6)**	**26·8<br>(18·7–33·5)**
**Latin America and Caribbean**	**Malaria**	**14·5<br>(-7·2–45·5)**	**10·8<br>(-5·3–31·2)**	**--**	**5·1<br>(-2·2–23·4)**
**Latin America and Caribbean**	**Measles**	**25·7<br>(12·3–32·4)**	**8·0<br>(3·8–10·6)**	**6·2<br>(2·4–11·7)**	**14·3<br>(6·5–19·7)**
Andean Latin America	All causes	9·8<br>(7·3–12·7)	6·3<br>(2·3–11·0)	3·6<br>(3·0–4·1)	3·9<br>(2·8–5·1)
Andean Latin America	Diarrheal diseases	44·9<br>(29·0–65·1)	14·3<br>(8·8–20·8)	23·4<br>(-0·73–56·4)	16·4<br>(11·4–21·3)
Andean Latin America	Lower respiratory infections	54·9<br>(40·0–74·2)	31·9<br>(1·1–67·2)	9·2<br>(5·6–12·6)	28·2<br>(19·9–35·0)
Andean Latin America	Malaria	13·8<br>(-7·0–45·3)	12·2<br>(-6·5–39·3)	--	2·0<br>(-0·79–11·3)
Andean Latin America	Measles	36·9<br>(18·5–45·2)	12·5<br>(6·1–16·9)	8·2<br>(3·1–15·7)	21·9<br>(10·5–29·0)
Bolivia (Plurinational State of)	All causes	12·0<br>(8·0–16·8)	7·7<br>(2·4–14·5)	4·4<br>(3·4–5·6)	4·8<br>(2·9–7·2)
Bolivia (Plurinational State of)	Diarrheal diseases	49·4<br>(30·4–73·5)	14·8<br>(8·5–22·2)	28·2<br>(-0·89–66·7)	17·5<br>(11·9–22·6)
Bolivia (Plurinational State of)	Lower respiratory infections	52·0<br>(38·4–70·2)	30·2<br>(1·0–64·4)	9·7<br>(5·8–13·4)	26·2<br>(16·1–33·9)
Bolivia (Plurinational State of)	Malaria	14·0<br>(-7·2–46·6)	12·2<br>(-6·4–39·0)	--	2·1<br>(-0·84–11·9)
Bolivia (Plurinational State of)	Measles	0<br>(0–0)	0<br>(0–0)	0<br>(0–0)	0<br>(0–0)
Ecuador	All causes	9·6<br>(7·3–12·2)	6·2<br>(2·2–10·4)	4·2<br>(3·6–4·9)	3·4<br>(2·4–4·6)
Ecuador	Diarrheal diseases	52·9<br>(29·1–75·9)	16·0<br>(9·6–22·6)	33·8<br>(-1·2–70·3)	15·8<br>(11·0–20·3)
Ecuador	Lower respiratory infections	59·3<br>(44·0–77·4)	34·9<br>(1·3–71·2)	15·2<br>(9·7–20·3)	27·5<br>(18·8–35·3)
Ecuador	Malaria	15·4<br>(-8·4–49·6)	14·0<br>(-8·0–43·8)	--	1·9<br>(-0·65–11·5)
Ecuador	Measles	36·9<br>(18·5–45·2)	12·5<br>(6·1–16·9)	8·2<br>(3·1–15·7)	21·9<br>(10·5–29·0)
Peru	All causes	8·8<br>(6·5–12·1)	5·6<br>(2·3–9·7)	2·7<br>(2·2–3·3)	3·8<br>(2·6–5·0)
Peru	Diarrheal diseases	38·6<br>(27·8–56·8)	13·2<br>(8·3–19·9)	15·6<br>(-0·46–46·2)	16·2<br>(11·4–21·5)

Peru	Lower respiratory infections	54·3 (38·4–76·2)	31·3 (1·0–67·9)	4·6 (2·5–6·7)	30·4 (23·7–36·4)
Peru	Malaria	13·5 (-6·7–44·2)	12·0 (-6·3–38·9)	--	1·9 (-0·74–11·1)
Peru	Measles	0 (0–0)	0 (0–0)	0 (0–0)	0 (0–0)
Caribbean	All causes	14·8 (10·1–19·1)	9·5 (5·0–13·3)	8·3 (4·9–11·2)	4·8 (3·3–6·6)
Caribbean	Diarrheal diseases	65·6 (42·6–83·0)	27·0 (16·6–37·5)	43·7 (-1·8–79·1)	19·1 (12·9–24·6)
Caribbean	Lower respiratory infections	50·6 (39·7–62·2)	33·1 (1·7–57·9)	15·7 (9·7–21·0)	23·8 (17·3–30·1)
Caribbean	Malaria	19·7 (-10·5–57·1)	14·4 (-7·4–38·5)	--	7·7 (-3·2–32·2)
Caribbean	Measles	11·6 (4·8–20·2)	3·0 (0·97–5·6)	5·5 (1·8–10·6)	4·0 (1·3–7·3)
Antigua and Barbuda	All causes	6·8 (4·8–9·4)	4·4 (1·8–7·9)	3·5 (2·9–4·3)	1·7 (1·2–2·5)
Antigua and Barbuda	Diarrheal diseases	43·1 (15·1–68·5)	11·3 (8·1–15·6)	31·2 (-1·4–62·9)	8·1 (5·9–10·8)
Antigua and Barbuda	Lower respiratory infections	45·9 (32·3–65·4)	26·4 (0·87–59·8)	15·1 (9·3–20·5)	16·1 (11·8–21·6)
Antigua and Barbuda	Malaria	0 (0–0)	0 (0–0)	--	0 (0–0)
Antigua and Barbuda	Measles	0 (0–0)	0 (0–0)	0 (0–0)	0 (0–0)
Bahamas	All causes	3·8 (2·6–5·4)	2·4 (0·77–4·7)	1·7 (1·3–2·1)	1·1 (0·74–1·6)
Bahamas	Diarrheal diseases	40·3 (15·8–64·1)	11·5 (8·2–15·6)	27·6 (-1·1–57·9)	8·7 (6·3–11·5)
Bahamas	Lower respiratory infections	42·0 (28·8–60·0)	25·0 (0·83–55·5)	11·9 (7·5–16·3)	15·5 (10·2–20·9)
Bahamas	Malaria	0 (0–0)	0 (0–0)	--	0 (0–0)
Bahamas	Measles	0 (0–0)	0 (0–0)	0 (0–0)	0 (0–0)
Barbados	All causes	2·6 (1·8–3·9)	1·6 (0·61–3·1)	1·2 (1·0–1·5)	0·71 (0·47–1·0)
Barbados	Diarrheal diseases	32·4 (8·4–53·7)	8·6 (6·3–12·4)	22·0 (-1·2–46·8)	6·9 (3·4–9·6)
Barbados	Lower respiratory infections	36·1 (24·9–51·9)	20·5 (0·60–47·7)	11·0 (6·8–14·9)	12·7 (7·7–17·9)
Barbados	Malaria	0 (0–0)	0 (0–0)	--	0 (0–0)
Barbados	Measles	0 (0–0)	0 (0–0)	0 (0–0)	0 (0–0)
Belize	All causes	7·8 (5·8–10·2)	5·1 (2·3–8·1)	3·4 (2·8–4·1)	2·7 (1·8–3·7)
Belize	Diarrheal diseases	46·6 (26·9–69·4)	14·4 (8·6–20·2)	27·1 (-0·76–63·3)	15·4 (10·5–19·6)
Belize	Lower respiratory infections	48·4 (34·3–64·4)	28·8 (1·1–59·8)	9·2 (5·5–12·8)	23·6 (15·2–30·9)
Belize	Malaria	0 (0–0)	0 (0–0)	--	0 (0–0)
Belize	Measles	0 (0–0)	0 (0–0)	0 (0–0)	0 (0–0)
Bermuda	All causes	0·90 (0·53–1·4)	0·51 (0·12–1·1)	0·39 (0·30–0·48)	0·23 (0·16–0·32)

Bermuda	Diarrheal diseases	15·4 (-0·81–31·7)	5·3 (1·2–10·9)	8·4 (-1·3–20·0)	3·2 (-0·67–7·7)
Bermuda	Lower respiratory infections	28·4 (18·4–44·5)	16·0 (0·42–40·5)	9·0 (5·5–12·3)	8·4 (5·2–11·9)
Bermuda	Malaria	0 (0–0)	0 (0–0)	--	0 (0–0)
Bermuda	Measles	3·7 (-1·2–9·9)	<0.1 (<0.1–<0.1)	3·4 (-1·1–9·0)	0·31 (>-0.1–1·0)
Cuba	All causes	4·7 (3·0–7·5)	2·5 (0·44–5·7)	1·4 (1·0–1·8)	1·8 (1·3–2·5)
Cuba	Diarrheal diseases	30·0 (10·5–54·2)	7·5 (5·9–10·6)	19·1 (-0·78–46·2)	7·3 (4·8–9·9)
Cuba	Lower respiratory infections	34·5 (23·3–51·9)	18·6 (0·54–45·4)	7·3 (4·2–10·3)	15·0 (11·7–19·3)
Cuba	Malaria	0 (0–0)	0 (0–0)	--	0 (0–0)
Cuba	Measles	0 (0–0)	0 (0–0)	0 (0–0)	0 (0–0)
Dominica	All causes	4·1 (2·3–6·2)	2·6 (1·2–5·0)	2·1 (1·4–2·8)	1·1 (0·64–1·7)
Dominica	Diarrheal diseases	42·5 (17·8–69·6)	12·0 (7·9–17·1)	29·2 (-1·1–64·7)	9·2 (6·5–12·5)
Dominica	Lower respiratory infections	45·3 (29·8–64·3)	27·0 (0·92–59·3)	12·8 (7·9–17·4)	16·6 (10·9–22·3)
Dominica	Malaria	0 (0–0)	0 (0–0)	--	0 (0–0)
Dominica	Measles	0 (0–0)	0 (0–0)	0 (0–0)	0 (0–0)
Dominican Republic	All causes	3·9 (2·7–5·5)	2·5 (1·3–4·1)	2·0 (1·5–2·8)	1·1 (0·58–1·6)
Dominican Republic	Diarrheal diseases	42·6 (21·1–65·8)	12·3 (7·1–18·0)	27·8 (-0·80–61·1)	10·6 (7·1–14·7)
Dominican Republic	Lower respiratory infections	37·8 (23·9–55·2)	22·9 (0·84–50·1)	9·2 (4·7–14·0)	14·9 (7·6–22·6)
Dominican Republic	Malaria	9·2 (-4·4–32·7)	7·8 (-3·8–25·6)	--	1·6 (-0·61–8·4)
Dominican Republic	Measles	0 (0–0)	0 (0–0)	0 (0–0)	0 (0–0)
Grenada	All causes	4·4 (2·9–6·3)	2·8 (0·75–5·3)	1·7 (1·2–2·2)	1·4 (0·97–2·1)
Grenada	Diarrheal diseases	35·1 (12·4–55·8)	11·5 (9·1–16·2)	22·3 (-1·2–47·4)	8·2 (4·6–11·4)
Grenada	Lower respiratory infections	31·6 (22·3–43·9)	19·1 (0·64–40·6)	8·6 (5·1–12·2)	12·4 (8·6–16·7)
Grenada	Malaria	0 (0–0)	0 (0–0)	--	0 (0–0)
Grenada	Measles	0 (0–0)	0 (0–0)	0 (0–0)	0 (0–0)
Guyana	All causes	8·5 (5·8–10·5)	6·1 (3·4–8·6)	5·6 (4·2–6·7)	1·8 (1·2–2·5)
Guyana	Diarrheal diseases	58·3 (32·2–77·5)	20·7 (11·9–29·1)	42·2 (-1·8–74·8)	12·7 (7·8–16·9)
Guyana	Lower respiratory infections	44·2 (31·7–56·3)	28·4 (1·3–52·9)	16·1 (9·9–22·2)	16·4 (10·5–22·2)
Guyana	Malaria	14·5 (-7·4–45·3)	12·0 (-6·3–35·8)	--	3·4 (-1·2–15·7)
Guyana	Measles	0 (0–0)	0 (0–0)	0 (0–0)	0 (0–0)

Haiti	All causes	18·9 (12·7–24·2)	12·1 (6·4–17·1)	10·6 (6·0–14·8)	6·1 (4·1–8·6)
Haiti	Diarrheal diseases	67·5 (44·7–84·3)	28·2 (17·4–39·1)	45·0 (-1·9–80·4)	19·9 (13·6–25·8)
Haiti	Lower respiratory infections	52·3 (41·1–63·9)	34·5 (1·8–59·1)	16·6 (10·3–22·0)	25·1 (18·8–31·6)
Haiti	Malaria	20·0 (-10·8–58·0)	14·6 (-7·5–38·8)	--	8·0 (-3·3–33·5)
Haiti	Measles	0 (0–0)	0 (0–0)	0 (0–0)	0 (0–0)
Jamaica	All causes	2·0 (1·3–2·8)	1·3 (0·72–2·0)	1·2 (0·76–1·6)	0·48 (0·31–0·70)
Jamaica	Diarrheal diseases	33·9 (15·6–55·0)	8·9 (5·3–13·0)	22·6 (-0·73–50·8)	8·0 (5·5–10·8)
Jamaica	Lower respiratory infections	29·0 (19·4–43·3)	16·9 (0·48–39·9)	7·0 (4·1–10·1)	11·4 (6·9–16·2)
Jamaica	Malaria	0 (0–0)	0 (0–0)	--	0 (0–0)
Jamaica	Measles	0 (0–0)	0 (0–0)	0 (0–0)	0 (0–0)
Puerto Rico	All causes	2·6 (1·7–3·9)	1·5 (0·77–2·8)	1·5 (1·1–1·9)	0·55 (0·40–0·76)
Puerto Rico	Diarrheal diseases	30·4 (8·1–53·8)	6·8 (5·2–9·8)	22·0 (-0·98–48·7)	5·2 (3·1–7·0)
Puerto Rico	Lower respiratory infections	30·4 (20·1–48·0)	16·9 (0·44–43·2)	8·9 (5·3–12·3)	10·1 (7·5–13·6)
Puerto Rico	Malaria	0 (0–0)	0 (0–0)	--	0 (0–0)
Puerto Rico	Measles	22·5 (10·1–29·8)	7·1 (3·2–9·6)	8·5 (3·2–15·6)	9·0 (3·9–12·5)
Saint Kitts and Nevis	All causes	5·0 (3·5–6·8)	3·3 (1·9–5·2)	2·9 (2·2–3·5)	1·1 (0·70–1·5)
Saint Kitts and Nevis	Diarrheal diseases	41·9 (17·6–65·8)	11·6 (7·3–16·3)	29·3 (-0·99–61·1)	8·7 (6·0–11·5)
Saint Kitts and Nevis	Lower respiratory infections	38·6 (26·1–55·0)	23·2 (0·77–50·8)	11·0 (6·7–15·1)	13·6 (8·8–18·6)
Saint Kitts and Nevis	Malaria	0 (0–0)	0 (0–0)	--	0 (0–0)
Saint Kitts and Nevis	Measles	0 (0–0)	0 (0–0)	0 (0–0)	0 (0–0)
Saint Lucia	All causes	4·5 (3·2–6·2)	3·1 (1·6–4·9)	2·7 (2·1–3·4)	0·76 (0·51–1·1)
Saint Lucia	Diarrheal diseases	41·8 (16·0–67·1)	11·7 (7·7–16·3)	30·5 (-1·2–62·8)	6·1 (4·1–8·3)
Saint Lucia	Lower respiratory infections	39·0 (25·3–57·9)	24·4 (0·85–53·7)	12·1 (7·5–16·7)	10·9 (7·4–15·2)
Saint Lucia	Malaria	0 (0–0)	0 (0–0)	--	0 (0–0)
Saint Lucia	Measles	0 (0–0)	0 (0–0)	0 (0–0)	0 (0–0)
Saint Vincent and the Grenadines	All causes	8·4 (6·1–11·4)	6·0 (3·2–9·5)	4·9 (4·0–5·9)	1·9 (1·2–2·6)
Saint Vincent and the Grenadines	Diarrheal diseases	46·6 (21·5–71·5)	13·3 (8·1–18·5)	32·2 (-1·0–67·3)	10·1 (6·7–13·1)
Saint Vincent and the Grenadines	Lower respiratory infections	47·6 (32·3–67·2)	28·7 (0·98–61·6)	13·0 (8·0–17·6)	18·1 (12·3–24·2)
Saint Vincent and the Grenadines	Malaria	0 (0–0)	0 (0–0)	--	0 (0–0)

Saint Vincent and the Grenadines	Measles	0<br>(0–0)	0<br>(0–0)	0<br>(0–0)	0<br>(0–0)
Suriname	All causes	6·7<br>(4·0–9·3)	4·1<br>(2·0–6·2)	3·9<br>(2·2–5·7)	1·5<br>(0·96–2·2)
Suriname	Diarrheal diseases	55·4<br>(27·3–78·6)	18·6<br>(10·7–27·0)	39·9<br>(-1·5–76·0)	10·5<br>(6·5–14·7)
Suriname	Lower respiratory infections	51·9<br>(35·7–68·1)	33·6<br>(1·4–64·5)	17·3<br>(10·4–23·2)	17·4<br>(10·9–24·0)
Suriname	Malaria	20·0<br>(-12·0–58·6)	20·0<br>(-12·0–58·6)	--	<0.1<br>(>-0.1–<0.1)
Suriname	Measles	0<br>(0–0)	0<br>(0–0)	0<br>(0–0)	0<br>(0–0)
Trinidad and Tobago	All causes	3·4<br>(2·5–4·5)	2·3<br>(1·2–3·6)	1·9<br>(1·5–2·2)	0·93<br>(0·61–1·3)
Trinidad and Tobago	Diarrheal diseases	48·0<br>(22·5–70·4)	15·5<br>(10·1–21·7)	32·2<br>(-1·2–65·1)	11·9<br>(8·2–15·7)
Trinidad and Tobago	Lower respiratory infections	43·0<br>(30·3–57·7)	26·6<br>(1·0–54·3)	12·2<br>(7·4–16·6)	17·4<br>(11·2–23·6)
Trinidad and Tobago	Malaria	0<br>(0–0)	0<br>(0–0)	--	0<br>(0–0)
Trinidad and Tobago	Measles	0<br>(0–0)	0<br>(0–0)	0<br>(0–0)	0<br>(0–0)
United States Virgin Islands	All causes	3·1<br>(2·0–4·3)	2·0<br>(1·3–2·9)	2·1<br>(1·4–3·0)	0·50<br>(0·33–0·72)
United States Virgin Islands	Diarrheal diseases	36·1<br>(13·1–59·7)	8·7<br>(6·0–12·2)	25·6<br>(-0·93–54·7)	7·0<br>(5·1–9·3)
United States Virgin Islands	Lower respiratory infections	35·1<br>(23·6–51·8)	20·1<br>(0·57–46·8)	9·9<br>(6·0–13·9)	12·6<br>(8·7–17·2)
United States Virgin Islands	Malaria	0<br>(0–0)	0<br>(0–0)	--	0<br>(0–0)
United States Virgin Islands	Measles	0<br>(0–0)	0<br>(0–0)	0<br>(0–0)	0<br>(0–0)
Central Latin America	All causes	10·0<br>(7·9–12·4)	6·2<br>(2·9–9·7)	3·8<br>(2·9–5·0)	4·3<br>(3·0–5·5)
Central Latin America	Diarrheal diseases	51·7<br>(37·0–70·3)	18·2<br>(10·7–26·1)	24·5<br>(-0·70–60·5)	22·3<br>(15·8–27·9)
Central Latin America	Lower respiratory infections	52·4<br>(38·9–68·4)	31·2<br>(1·3–63·4)	7·4<br>(4·4–10·2)	29·2<br>(19·3–36·9)
Central Latin America	Malaria	10·6<br>(-4·7–36·6)	7·8<br>(-3·4–23·0)	--	3·4<br>(-1·4–17·5)
Central Latin America	Measles	8·9<br>(2·7–17·9)	1·8<br>(0·61–3·6)	3·2<br>(0·30–6·9)	4·7<br>(1·5–9·7)
Colombia	All causes	8·9<br>(6·8–11·3)	6·5<br>(4·2–9·6)	5·3<br>(4·6–6·2)	1·9<br>(1·4–2·6)
Colombia	Diarrheal diseases	37·8<br>(18·2–62·2)	10·5<br>(6·8–14·7)	23·4<br>(-0·77–56·6)	9·9<br>(7·1–12·6)
Colombia	Lower respiratory infections	43·0<br>(29·1–64·0)	25·2<br>(0·80–57·7)	8·6<br>(5·1–11·8)	18·3<br>(13·4–23·8)
Colombia	Malaria	8·9<br>(-3·9–30·8)	6·7<br>(-3·0–21·4)	--	2·6<br>(-0·98–11·9)
Colombia	Measles	0<br>(0–0)	0<br>(0–0)	0<br>(0–0)	0<br>(0–0)
Costa Rica	All causes	1·6<br>(0·98–3·1)	0·79<br>(0·15–2·4)	0·63<br>(0·48–0·81)	0·46<br>(0·33–0·63)
Costa Rica	Diarrheal diseases	20·1<br>(3·9–44·3)	3·5<br>(2·6–5·3)	14·5<br>(-0·62–39·3)	3·1<br>(1·7–4·6)
Costa Rica	Lower respiratory infections	19·3<br>(12·7–35·7)	9·5<br>(0·20–31·0)	5·4<br>(3·1–7·7)	6·2<br>(5·0–8·2)

Costa Rica	Malaria	8·4 (-3·5–28·7)	8·4 (-3·5–28·7)	--	<0.1 (>-0.1–<0.1)
Costa Rica	Measles	0 (0–0)	0 (0–0)	0 (0–0)	0 (0–0)
El Salvador	All causes	7·3 (5·3–10·3)	4·5 (2·0–8·0)	2·8 (2·1–3·7)	2·7 (1·8–3·7)
El Salvador	Diarrheal diseases	46·3 (27·7–68·3)	15·2 (9·2–21·8)	26·2 (-0·82–61·1)	14·9 (10·2–19·0)
El Salvador	Lower respiratory infections	50·2 (35·9–69·1)	30·6 (1·2–64·1)	9·2 (5·3–12·8)	24·0 (15·6–31·2)
El Salvador	Malaria	0 (0–0)	0 (0–0)	--	0 (0–0)
El Salvador	Measles	0 (0–0)	0 (0–0)	0 (0–0)	0 (0–0)
Guatemala	All causes	23·3 (18·5–28·1)	14·3 (5·6–21·7)	6·0 (3·9–9·2)	12·5 (8·8–16·0)
Guatemala	Diarrheal diseases	60·7 (50·6–73·5)	25·7 (15·7–36·2)	23·2 (-0·66–59·6)	31·3 (22·6–38·5)
Guatemala	Lower respiratory infections	64·4 (49·6–77·4)	41·2 (1·9–73·3)	6·7 (3·8–9·4)	40·1 (26·3–49·7)
Guatemala	Malaria	24·3 (-16·4–65·7)	23·6 (-16·4–63·3)	--	1·0 (-0·88–15·5)
Guatemala	Measles	0 (0–0)	0 (0–0)	0 (0–0)	0 (0–0)
Honduras	All causes	6·0 (3·9–8·7)	3·5 (1·6–5·8)	2·4 (1·3–4·1)	2·4 (1·6–3·3)
Honduras	Diarrheal diseases	46·1 (31·5–65·1)	16·8 (10·2–24·1)	24·2 (-0·75–58·1)	16·2 (11·0–20·5)
Honduras	Lower respiratory infections	38·0 (27·1–49·6)	23·8 (0·96–44·5)	6·0 (3·4–8·5)	20·0 (14·3–25·6)
Honduras	Malaria	19·7 (-12·2–57·1)	19·0 (-11·8–56·5)	--	0·97 (-0·40–5·3)
Honduras	Measles	0 (0–0)	0 (0–0)	0 (0–0)	0 (0–0)
Mexico	All causes	8·1 (6·4–10·2)	4·8 (2·0–8·0)	2·7 (2·1–3·6)	3·6 (2·5–4·8)
Mexico	Diarrheal diseases	50·1 (36·3–68·8)	16·8 (9·4–24·4)	23·5 (-0·63–60·0)	21·9 (14·9–27·8)
Mexico	Lower respiratory infections	53·7 (40·1–71·0)	30·8 (1·2–64·9)	7·3 (4·3–10·1)	30·1 (19·7–38·5)
Mexico	Malaria	17·5 (-9·9–49·0)	17·5 (-9·9–49·0)	--	<0.1 (>-0.1–<0.1)
Mexico	Measles	0 (0–0)	0 (0–0)	0 (0–0)	0 (0–0)
Nicaragua	All causes	9·5 (6·6–13·0)	6·1 (3·1–9·9)	4·7 (3·4–6·1)	2·7 (1·7–3·8)
Nicaragua	Diarrheal diseases	45·6 (23·6–70·1)	13·0 (7·4–18·7)	28·3 (-0·75–64·9)	12·8 (8·5–16·5)
Nicaragua	Lower respiratory infections	43·1 (29·5–61·3)	25·8 (0·90–57·5)	9·2 (5·4–12·7)	18·2 (10·8–25·0)
Nicaragua	Malaria	12·4 (-6·3–43·0)	10·4 (-5·8–36·9)	--	2·5 (-1·0–13·3)
Nicaragua	Measles	0 (0–0)	0 (0–0)	0 (0–0)	0 (0–0)
Panama	All causes	10·4 (7·7–14·0)	6·4 (2·7–11·4)	3·9 (3·1–4·9)	3·9 (2·7–5·3)
Panama	Diarrheal diseases	40·2 (23·1–63·2)	12·0 (7·8–16·9)	20·7 (-0·60–54·7)	14·5 (10·7–18·4)

Panama	Lower respiratory infections	48·5 (34·4–68·6)	28·2 (0·91–63·0)	6·9 (4·0–9·5)	24·4 (16·5–31·6)
Panama	Malaria	12·7 (-5·9–40·3)	12·1 (-5·9–39·7)	--	0·65 (-0·20–5·7)
Panama	Measles	0 (0–0)	0 (0–0)	0 (0–0)	0 (0–0)
Venezuela (Bolivarian Republic of)	All causes	8·5 (6·3–10·8)	5·0 (3·1–7·2)	4·3 (2·9–5·9)	2·9 (2·0–3·8)
Venezuela (Bolivarian Republic of)	Diarrheal diseases	50·6 (30·3–73·8)	14·0 (8·0–20·3)	29·5 (-0·82–67·8)	18·2 (12·2–23·0)
Venezuela (Bolivarian Republic of)	Lower respiratory infections	39·7 (29·4–53·1)	22·5 (0·81–47·2)	7·7 (4·4–10·9)	20·7 (13·3–27·5)
Venezuela (Bolivarian Republic of)	Malaria	13·4 (-6·7–44·9)	9·9 (-4·7–32·4)	--	4·4 (-2·1–21·9)
Venezuela (Bolivarian Republic of)	Measles	8·9 (2·7–17·9)	1·8 (0·61–3·6)	3·2 (0·30–6·9)	4·7 (1·5–9·7)
Tropical Latin America	All causes	4·2 (2·9–5·9)	2·4 (0·83–4·6)	1·6 (1·3–1·9)	1·5 (1·1–2·0)
Tropical Latin America	Diarrheal diseases	42·8 (21·6–68·9)	11·1 (6·7–15·8)	26·9 (-0·80–63·8)	12·2 (8·3–15·6)
Tropical Latin America	Lower respiratory infections	50·3 (35·5–73·6)	28·4 (0·90–65·8)	10·2 (6·1–14·0)	22·5 (16·3–29·0)
Tropical Latin America	Malaria	10·4 (-4·8–36·6)	8·2 (-3·9–26·6)	--	2·6 (-1·1–12·2)
Tropical Latin America	Measles	27·3 (12·9–34·5)	8·5 (4·0–11·2)	6·4 (2·5–12·3)	15·3 (6·9–21·5)
Brazil	All causes	4·2 (2·9–5·9)	2·4 (0·79–4·6)	1·6 (1·2–1·9)	1·5 (1·1–2·0)
Brazil	Diarrheal diseases	43·6 (21·9–70·1)	11·2 (6·8–16·0)	27·6 (-0·82–64·9)	12·4 (8·5–15·8)
Brazil	Lower respiratory infections	50·8 (36·0–74·1)	28·7 (0·91–66·3)	10·5 (6·2–14·4)	22·8 (16·5–29·2)
Brazil	Malaria	10·4 (-4·8–36·6)	8·2 (-3·9–26·6)	--	2·6 (-1·1–12·2)
Brazil	Measles	27·3 (12·9–34·5)	8·5 (4·0–11·2)	6·4 (2·5–12·3)	15·3 (6·9–21·5)
Paraguay	All causes	4·1 (3·0–5·8)	2·7 (1·5–4·6)	1·8 (1·4–2·3)	1·2 (0·76–1·7)
Paraguay	Diarrheal diseases	29·8 (15·6–49·0)	8·7 (4·9–13·2)	16·0 (-0·37–43·3)	9·3 (5·8–12·7)
Paraguay	Lower respiratory infections	39·2 (25·5–60·9)	22·4 (0·67–53·8)	5·2 (2·9–7·4)	17·7 (12·3–23·7)
Paraguay	Malaria	0 (0–0)	0 (0–0)	--	0 (0–0)
Paraguay	Measles	0 (0–0)	0 (0–0)	0 (0–0)	0 (0–0)
**North Africa and Middle East**	**All causes**	**10·1 (7·3–12·8)**	**6·5 (3·2–9·6)**	**5·4 (3·9–6·9)**	**4·1 (3·0–5·3)**
**North Africa and Middle East**	**Diarrheal diseases**	**64·7 (39·9–80·9)**	**28·2 (19·2–37·9)**	**44·7 (-2·1–77·1)**	**22·5 (16·2–27·9)**
**North Africa and Middle East**	**Lower respiratory infections**	**54·0 (43·8–63·1)**	**34·5 (2·0–58·7)**	**20·7 (13·9–27·0)**	**27·5 (18·9–34·8)**
**North Africa and Middle East**	**Malaria**	**31·3 (-23·3–78·1)**	**24·7 (-17·2–60·2)**	**--**	**12·3 (-6·3–49·0)**
**North Africa and Middle East**	**Measles**	**61·1 (34·4–70·9)**	**29·8 (15·1–38·1)**	**22·4 (9·3–38·7)**	**33·3 (15·5–41·2)**
North Africa and Middle East	All causes	10·1 (7·3–12·8)	6·5 (3·2–9·6)	5·4 (3·9–6·9)	4·1 (3·0–5·3)

North Africa and Middle East	Diarrheal diseases	64·7 (39·9–80·9)	28·2 (19·2–37·9)	44·7 (-2·1–77·1)	22·5 (16·2–27·9)
North Africa and Middle East	Lower respiratory infections	54·0 (43·8–63·1)	34·5 (2·0–58·7)	20·7 (13·9–27·0)	27·5 (18·9–34·8)
North Africa and Middle East	Malaria	31·3 (-23·3–78·1)	24·7 (-17·2–60·2)	--	12·3 (-6·3–49·0)
North Africa and Middle East	Measles	61·1 (34·4–70·9)	29·8 (15·1–38·1)	22·4 (9·3–38·7)	33·3 (15·5–41·2)
Afghanistan	All causes	18·4 (13·9–22·6)	12·6 (7·2–17·0)	10·6 (7·2–14·0)	7·2 (4·8–9·8)
Afghanistan	Diarrheal diseases	76·0 (53·6–87·4)	38·5 (26·1–49·8)	51·3 (-2·2–83·6)	29·2 (21·5–35·9)
Afghanistan	Lower respiratory infections	56·0 (44·0–65·1)	38·7 (2·2–61·4)	21·6 (14·4–29·0)	30·4 (20·6–39·2)
Afghanistan	Malaria	27·1 (-19·3–72·5)	21·5 (-15·3–55·4)	--	9·3 (-4·5–40·2)
Afghanistan	Measles	69·1 (40·0–79·6)	35·5 (18·2–44·9)	24·7 (10·1–42·9)	38·2 (18·1–46·8)
Algeria	All causes	4·2 (2·7–6·0)	2·1 (0·77–4·0)	1·9 (1·3–2·6)	1·6 (1·0–2·3)
Algeria	Diarrheal diseases	46·6 (17·8–70·3)	13·1 (9·6–18·3)	32·7 (-1·5–63·8)	11·9 (7·9–16·1)
Algeria	Lower respiratory infections	49·6 (36·9–64·1)	27·9 (0·97–58·5)	17·2 (11·2–22·6)	20·8 (13·7–27·5)
Algeria	Malaria	0 (0–0)	0 (0–0)	--	0 (0–0)
Algeria	Measles	36·4 (17·1–46·1)	10·8 (5·0–14·5)	12·9 (4·7–23·6)	18·6 (8·2–24·5)
Bahrain	All causes	2·2 (1·2–3·2)	1·2 (0·36–2·4)	1·1 (0·74–1·5)	0·63 (0·43–0·91)
Bahrain	Diarrheal diseases	33·6 (6·8–54·0)	9·4 (6·3–14·3)	23·1 (-1·6–44·2)	6·7 (2·4–10·7)
Bahrain	Lower respiratory infections	41·7 (29·1–57·9)	23·2 (0·73–52·1)	14·5 (8·9–19·9)	15·0 (11·2–19·6)
Bahrain	Malaria	0 (0–0)	0 (0–0)	--	0 (0–0)
Bahrain	Measles	28·7 (0–39·2)	8·8 (0–12·3)	12·2 (0–23·1)	11·5 (0–16·3)
Egypt	All causes	10·0 (7·0–14·3)	5·1 (1·3–10·0)	4·7 (3·3–6·3)	4·2 (3·0–5·7)
Egypt	Diarrheal diseases	52·8 (24·6–75·3)	14·1 (8·5–20·5)	38·1 (-1·4–71·7)	16·2 (11·2–20·8)
Egypt	Lower respiratory infections	45·3 (35·2–57·2)	24·6 (1·0–51·2)	16·4 (9·8–23·0)	21·8 (14·4–29·5)
Egypt	Malaria	0 (0–0)	0 (0–0)	--	0 (0–0)
Egypt	Measles	42·4 (20·8–52·5)	11·3 (5·4–15·1)	15·6 (5·7–28·8)	23·6 (10·7–30·4)
Iran (Islamic Republic of)	All causes	2·1 (1·3–3·1)	1·2 (0·40–2·4)	1·0 (0·74–1·3)	0·57 (0·36–0·86)
Iran (Islamic Republic of)	Diarrheal diseases	43·1 (16·8–67·2)	12·6 (9·0–17·3)	30·6 (-1·6–61·5)	8·8 (6·4–11·6)
Iran (Islamic Republic of)	Lower respiratory infections	43·6 (30·3–62·2)	25·8 (0·88–56·6)	14·2 (8·9–18·9)	15·2 (10·4–20·5)
Iran (Islamic Republic of)	Malaria	17·1 (-8·6–51·7)	17·1 (-8·6–51·6)	--	<0.1 (>-0.1–<0.1)
Iran (Islamic Republic of)	Measles	35·9 (18·2–44·4)	13·3 (6·6–17·4)	13·9 (5·6–24·2)	15·8 (7·3–20·7)

Iraq	All causes	5·8 (3·9–8·0)	3·1 (1·2–5·4)	2·8 (1·9–3·9)	2·3 (1·5–3·3)
Iraq	Diarrheal diseases	52·3 (25·4–72·6)	16·2 (11·0–22·6)	36·6 (-1·6–67·5)	15·5 (11·4–19·8)
Iraq	Lower respiratory infections	54·5 (42·6–67·3)	31·3 (1·2–62·1)	19·0 (12·5–24·9)	25·7 (17·9–33·2)
Iraq	Malaria	0 (0–0)	0 (0–0)	--	0 (0–0)
Iraq	Measles	42·4 (20·2–52·5)	12·9 (6·1–17·1)	15·2 (5·6–27·6)	22·6 (10·0–30·1)
Jordan	All causes	4·0 (2·4–6·6)	2·1 (0·26–5·3)	1·3 (0·92–1·8)	1·6 (0·99–2·5)
Jordan	Diarrheal diseases	32·3 (9·9–55·1)	8·1 (6·3–11·7)	20·7 (-1·0–46·4)	8·3 (4·5–11·5)
Jordan	Lower respiratory infections	40·0 (28·6–58·2)	21·6 (0·63–52·9)	10·1 (6·1–13·8)	17·1 (11·8–22·6)
Jordan	Malaria	0 (0–0)	0 (0–0)	--	0 (0–0)
Jordan	Measles	25·6 (12·2–31·2)	7·4 (3·6–9·7)	7·0 (2·6–12·8)	13·7 (6·3–18·2)
Kuwait	All causes	4·0 (2·5–6·4)	2·3 (0·12–5·5)	1·0 (0·70–1·4)	1·5 (1·1–2·0)
Kuwait	Diarrheal diseases	18·4 (1·7–34·5)	5·9 (2·6–10·4)	10·3 (-1·1–24·4)	4·3 (0·41–8·1)
Kuwait	Lower respiratory infections	33·1 (22·1–51·2)	19·0 (0·53–46·0)	7·9 (4·7–10·9)	12·2 (9·0–16·3)
Kuwait	Malaria	0 (0–0)	0 (0–0)	--	0 (0–0)
Kuwait	Measles	3·0 (-1·0–8·1)	<0.1 (<0.1–<0.1)	2·8 (-0·93–7·4)	0·23 (>-0.1–0·80)
Lebanon	All causes	6·2 (4·3–8·5)	2·7 (0·63–5·4)	2·4 (1·6–3·2)	2·9 (2·0–4·1)
Lebanon	Diarrheal diseases	42·0 (17·5–65·2)	9·6 (6·5–13·4)	28·1 (-1·1–60·1)	13·6 (10·1–17·3)
Lebanon	Lower respiratory infections	40·3 (31·3–52·9)	20·5 (0·63–46·5)	11·3 (6·8–15·7)	20·5 (13·8–27·2)
Lebanon	Malaria	0 (0–0)	0 (0–0)	--	0 (0–0)
Lebanon	Measles	35·2 (16·7–44·4)	8·2 (3·9–10·9)	11·9 (4·3–22·2)	20·2 (9·3–26·9)
Libya	All causes	2·9 (1·9–4·1)	1·6 (0·38–3·2)	1·2 (0·83–1·7)	1·3 (0·85–1·9)
Libya	Diarrheal diseases	48·9 (20·0–73·7)	14·4 (11·3–20·3)	32·2 (-2·0–64·7)	15·9 (10·6–21·2)
Libya	Lower respiratory infections	62·8 (50·0–78·0)	34·1 (1·3–68·6)	19·6 (12·2–26·5)	33·4 (26·2–40·3)
Libya	Malaria	0 (0–0)	0 (0–0)	--	0 (0–0)
Libya	Measles	42·8 (21·6–52·8)	11·9 (5·9–15·7)	14·9 (5·6–26·8)	24·3 (11·2–31·1)
Morocco	All causes	6·1 (3·8–9·4)	2·7 (0·87–5·6)	2·5 (1·4–4·5)	2·5 (1·7–3·7)
Morocco	Diarrheal diseases	48·5 (23·6–75·9)	11·5 (6·6–16·8)	32·9 (-1·1–72·0)	14·0 (9·5–18·1)
Morocco	Lower respiratory infections	42·1 (31·1–58·3)	22·6 (0·73–51·6)	11·1 (6·8–15·5)	19·8 (13·3–25·9)
Morocco	Malaria	0 (0–0)	0 (0–0)	--	0 (0–0)

Morocco	Measles	31·9 (15·0–39·8)	8·2 (3·9–10·9)	9·1 (3·2–17·4)	18·6 (8·5–24·3)
Oman	All causes	4·4 (2·7–6·4)	2·9 (1·0–5·1)	2·4 (1·7–3·2)	1·2 (0·77–1·8)
Oman	Diarrheal diseases	48·9 (18·9–69·1)	18·8 (14·6–25·5)	34·2 (-2·4–61·6)	9·7 (6·4–13·6)
Oman	Lower respiratory infections	48·8 (34·8–62·7)	31·6 (1·4–58·7)	18·8 (12·1–24·9)	17·1 (12·4–22·3)
Oman	Malaria	21·9 (-14·3–61·2)	21·9 (-14·3–61·2)	--	<0.1 (>-0.1–<0.1)
Oman	Measles	43·3 (22·0–54·8)	18·6 (9·0–24·1)	18·3 (7·5–31·7)	15·9 (6·9–21·3)
Palestine	All causes	1·5 (1·0–2·4)	0·72 (0·15–1·7)	0·45 (0·34–0·57)	0·67 (0·49–0·92)
Palestine	Diarrheal diseases	29·3 (9·0–52·4)	6·9 (5·5–10·1)	18·4 (-0·83–44·3)	8·1 (4·5–11·4)
Palestine	Lower respiratory infections	34·7 (25·4–51·8)	17·9 (0·49–44·0)	7·6 (4·4–10·7)	16·1 (11·9–20·7)
Palestine	Malaria	0 (0–0)	0 (0–0)	--	0 (0–0)
Palestine	Measles	23·3 (10·8–28·3)	5·9 (2·8–7·8)	6·2 (2·3–11·5)	13·3 (6·0–17·8)
Qatar	All causes	2·4 (1·4–4·0)	1·2 (0·18–2·7)	0·90 (0·63–1·2)	0·83 (0·59–1·3)
Qatar	Diarrheal diseases	23·6 (4·1–44·1)	5·5 (3·4–9·3)	15·9 (-1·1–35·6)	4·7 (1·4–8·5)
Qatar	Lower respiratory infections	31·5 (22·2–50·7)	16·3 (0·42–44·2)	9·4 (5·7–13·4)	11·6 (8·9–15·3)
Qatar	Malaria	0 (0–0)	0 (0–0)	--	0 (0–0)
Qatar	Measles	20·1 (9·2–26·3)	5·3 (2·5–7·1)	7·4 (2·8–13·6)	9·1 (4·2–12·5)
Saudi Arabia	All causes	7·9 (5·6–10·8)	4·0 (1·1–8·0)	4·3 (3·3–5·5)	2·3 (1·6–3·3)
Saudi Arabia	Diarrheal diseases	48·6 (13·0–74·5)	10·6 (7·7–14·8)	38·5 (-2·1–70·0)	8·3 (5·8–11·0)
Saudi Arabia	Lower respiratory infections	52·0 (40·2–68·3)	26·8 (0·86–60·8)	23·2 (15·1–30·6)	17·8 (13·4–22·9)
Saudi Arabia	Malaria	15·1 (-7·6–47·5)	15·0 (-7·6–47·3)	--	0·11 (>-0.1–0·62)
Saudi Arabia	Measles	34·5 (16·6–45·9)	9·5 (4·5–12·7)	18·1 (6·8–32·0)	12·2 (5·3–16·6)
Sudan	All causes	11·2 (5·9–15·7)	7·8 (2·2–12·1)	5·8 (4·0–7·6)	4·1 (2·7–5·5)
Sudan	Diarrheal diseases	72·1 (43·7–85·9)	35·0 (25·6–45·7)	52·0 (-3·9–81·1)	20·4 (14·8–25·2)
Sudan	Lower respiratory infections	70·1 (55·9–78·9)	48·9 (3·1–75·2)	31·5 (21·7–40·3)	32·8 (24·6–40·1)
Sudan	Malaria	30·2 (-22·0–77·0)	23·8 (-16·2–58·7)	--	11·6 (-5·3–45·5)
Sudan	Measles	70·3 (43·0–81·7)	37·6 (20·5–46·8)	32·1 (14·5–50·9)	32·7 (15·8–40·9)
Syrian Arab Republic	All causes	7·5 (5·9–9·3)	4·7 (2·8–6·8)	4·1 (3·2–5·0)	3·2 (2·3–4·2)
Syrian Arab Republic	Diarrheal diseases	54·5 (23·5–74·9)	19·7 (15·8–26·6)	36·0 (-2·4–65·0)	19·7 (12·0–25·2)
Syrian Arab Republic	Lower respiratory infections	70·7 (58·1–82·9)	41·1 (1·8–73·2)	24·9 (15·2–34·0)	41·3 (34·6–47·7)

Syrian Arab Republic	Malaria	0 (0–0)	0 (0–0)	--	0 (0–0)
Syrian Arab Republic	Measles	58·0 (30·5–70·2)	19·5 (9·5–25·5)	24·4 (9·4–41·7)	32·6 (15·2–40·9)
Tunisia	All causes	3·9 (2·5–5·7)	1·8 (0·67–3·5)	1·8 (1·1–2·8)	1·4 (0·94–2·1)
Tunisia	Diarrheal diseases	41·7 (17·4–66·7)	9·6 (5·8–14·0)	29·0 (-0·87–62·9)	11·1 (7·7–14·6)
Tunisia	Lower respiratory infections	42·4 (31·4–59·6)	22·3 (0·65–52·8)	11·6 (7·1–15·8)	19·0 (12·7–25·3)
Tunisia	Malaria	0 (0–0)	0 (0–0)	--	0 (0–0)
Tunisia	Measles	25·2 (11·6–31·8)	5·7 (2·6–7·6)	7·4 (2·7–14·1)	14·5 (6·5–19·2)
Türkiye	All causes	1·7 (1·1–2·4)	0·84 (0·35–1·6)	0·72 (0·50–0·97)	0·57 (0·38–0·82)
Türkiye	Diarrheal diseases	28·6 (8·9–51·7)	5·9 (4·5–8·3)	19·0 (-0·69–45·2)	7·0 (4·3–9·2)
Türkiye	Lower respiratory infections	34·0 (21·6–52·8)	17·0 (0·43–45·9)	8·3 (4·4–12·4)	14·6 (10·0–20·1)
Türkiye	Malaria	0 (0–0)	0 (0–0)	--	0 (0–0)
Türkiye	Measles	19·8 (9·1–24·1)	5·1 (2·4–6·8)	4·4 (1·6–8·2)	11·7 (5·1–15·7)
United Arab Emirates	All causes	6·3 (4·3–8·3)	3·8 (1·4–6·4)	3·5 (2·7–4·6)	1·9 (1·3–2·7)
United Arab Emirates	Diarrheal diseases	47·1 (15·9–68·6)	16·3 (12·9–22·7)	33·8 (-2·5–59·5)	10·3 (5·7–14·2)
United Arab Emirates	Lower respiratory infections	50·2 (38·2–63·3)	30·1 (1·1–57·9)	21·7 (14·3–28·5)	19·6 (14·6–25·3)
United Arab Emirates	Malaria	0 (0–0)	0 (0–0)	--	0 (0–0)
United Arab Emirates	Measles	47·9 (24·3–62·7)	17·7 (8·6–23·5)	25·4 (10·4–43·5)	16·9 (7·6–22·6)
Yemen	All causes	11·7 (7·7–15·1)	8·8 (3·2–12·6)	6·3 (4·6–8·1)	5·3 (3·7–7·1)
Yemen	Diarrheal diseases	77·8 (58·3–86·7)	46·0 (34·7–56·2)	53·5 (-4·0–81·5)	31·3 (23·2–37·4)
Yemen	Lower respiratory infections	73·8 (63·4–80·7)	54·9 (4·6–77·1)	32·3 (22·4–40·8)	42·6 (30·3–51·1)
Yemen	Malaria	34·8 (-31·0–81·8)	27·9 (-22·9–66·5)	--	13·9 (-7·5–53·4)
Yemen	Measles	77·4 (50·0–85·4)	46·4 (26·2–55·3)	29·8 (13·7–47·6)	43·0 (21·4–51·5)
**South Asia**	**All causes**	**14·1 (11·4–16·3)**	**9·9 (4·6–12·8)**	**8·7 (6·4–10·2)**	**5·7 (4·1–7·3)**
**South Asia**	**Diarrheal diseases**	**77·1 (55·2–86·8)**	**41·8 (30·2–51·9)**	**56·2 (-3·9–83·6)**	**26·7 (19·0–32·4)**
**South Asia**	**Lower respiratory infections**	**53·1 (45·1–58·8)**	**38·2 (2·9–55·9)**	**26·8 (19·4–33·3)**	**26·8 (17·3–33·8)**
**South Asia**	**Malaria**	**32·1 (-24·4–78·9)**	**25·2 (-17·7–60·4)**	**--**	**13·4 (-7·2–50·9)**
**South Asia**	**Measles**	**75·2 (48·7–84·8)**	**42·0 (23·7–51·0)**	**35·8 (16·4–55·2)**	**38·0 (19·0–46·4)**
South Asia	All causes	14·1 (11·4–16·3)	9·9 (4·6–12·8)	8·7 (6·4–10·2)	5·7 (4·1–7·3)
South Asia	Diarrheal diseases	77·1 (55·2–86·8)	41·8 (30·2–51·9)	56·2 (-3·9–83·6)	26·7 (19·0–32·4)

South Asia	Lower respiratory infections	53·1 (45·1–58·8)	38·2 (2·9–55·9)	26·8 (19·4–33·3)	26·8 (17·3–33·8)
South Asia	Malaria	32·1 (-24·4–78·9)	25·2 (-17·7–60·4)	--	13·4 (-7·2–50·9)
South Asia	Measles	75·2 (48·7–84·8)	42·0 (23·7–51·0)	35·8 (16·4–55·2)	38·0 (19·0–46·4)
Bangladesh	All causes	9·9 (7·5–12·0)	7·4 (4·0–9·9)	6·2 (5·0–7·5)	3·0 (1·9–4·1)
Bangladesh	Diarrheal diseases	68·4 (43·9–82·6)	32·8 (22·2–42·7)	47·5 (-3·1–78·5)	20·5 (14·1–25·4)
Bangladesh	Lower respiratory infections	40·5 (29·0–50·2)	28·3 (1·7–46·8)	16·2 (9·9–22·9)	18·9 (12·0–26·3)
Bangladesh	Malaria	29·9 (-28·7–74·1)	29·2 (-28·0–71·1)	--	1·2 (-0·57–10·8)
Bangladesh	Measles	65·4 (38·5–75·0)	34·4 (18·5–42·5)	25·5 (11·0–42·4)	31·3 (15·2–39·6)
Bhutan	All causes	10·1 (6·1–15·2)	5·1 (2·0–8·9)	4·8 (2·1–8·9)	3·9 (2·7–5·7)
Bhutan	Diarrheal diseases	60·2 (34·7–81·5)	20·1 (11·8–28·5)	40·4 (-1·5–77·2)	17·7 (11·9–22·6)
Bhutan	Lower respiratory infections	52·3 (39·8–66·2)	32·1 (1·4–61·6)	15·4 (9·5–20·9)	25·1 (18·5–31·8)
Bhutan	Malaria	20·3 (-13·1–59·5)	18·0 (-10·6–50·2)	--	3·2 (-1·3–22·4)
Bhutan	Measles	46·1 (24·3–54·9)	17·6 (8·8–22·7)	13·0 (4·8–23·3)	25·3 (12·3–32·6)
India	All causes	15·6 (12·7–18·2)	11·1 (5·2–14·5)	9·8 (7·5–11·7)	6·6 (4·9–8·6)
India	Diarrheal diseases	77·5 (56·6–86·3)	44·1 (32·6–54·2)	57·1 (-4·1–82·2)	28·5 (20·5–34·6)
India	Lower respiratory infections	51·7 (44·8–57·7)	37·5 (3·0–54·8)	27·5 (20·1–34·7)	26·7 (16·6–34·3)
India	Malaria	31·9 (-24·0–78·8)	25·2 (-17·6–60·3)	--	12·9 (-6·4–48·8)
India	Measles	77·2 (50·8–86·9)	43·7 (24·9–52·8)	38·0 (17·6–57·8)	39·1 (19·7–47·6)
Nepal	All causes	12·0 (9·1–15·2)	8·5 (4·4–12·2)	6·7 (5·3–8·3)	4·2 (2·9–5·8)
Nepal	Diarrheal diseases	67·4 (42·7–80·4)	31·7 (21·5–41·2)	47·0 (-2·5–76·0)	20·2 (13·7–25·2)
Nepal	Lower respiratory infections	51·6 (39·8–61·6)	35·6 (2·1–57·3)	20·9 (13·8–28·5)	23·9 (16·0–31·2)
Nepal	Malaria	28·5 (-25·0–72·2)	26·9 (-23·8–68·1)	--	2·8 (-1·0–14·5)
Nepal	Measles	65·6 (38·3–75·7)	33·7 (17·8–42·2)	24·5 (10·5–40·6)	32·8 (15·6–41·0)
Pakistan	All causes	12·1 (8·6–15·9)	8·0 (3·3–11·3)	7·1 (4·1–9·9)	4·4 (3·1–6·2)
Pakistan	Diarrheal diseases	77·3 (53·1–90·0)	38·3 (26·9–49·7)	55·5 (-3·6–86·0)	24·0 (17·3–29·8)
Pakistan	Lower respiratory infections	64·4 (52·9–74·3)	45·1 (3·0–69·7)	28·3 (19·6–36·0)	30·6 (21·0–38·9)
Pakistan	Malaria	32·7 (-26·8–79·2)	25·3 (-19·0–62·2)	--	14·4 (-8·9–53·2)
Pakistan	Measles	69·2 (42·7–79·5)	36·1 (19·8–44·6)	29·8 (13·2–48·1)	35·5 (17·9–44·2)
**Southeast Asia, East Asia, and Oceania**	**All causes**	**11·2 (8·4–13·7)**	**7·1 (3·0–10·4)**	**5·5 (4·0–7·1)**	**4·6 (3·4–5·9)**

**Southeast Asia, East Asia, and Oceania**	**Diarrheal diseases**	**68·5 (44·7–83·9)**	**31·0 (21·3–40·3)**	**45·1 (-2·3–78·0)**	**23·8 (17·2–29·2)**
**Southeast Asia, East Asia, and Oceania**	**Lower respiratory infections**	**56·5 (43·5–66·0)**	**37·8 (2·2–62·6)**	**19·5 (13·0–25·3)**	**28·0 (19·3–34·9)**
**Southeast Asia, East Asia, and Oceania**	**Malaria**	**26·1 (-18·5–69·2)**	**19·6 (-12·5–51·3)**	**--**	**10·6 (-5·5–42·1)**
**Southeast Asia, East Asia, and Oceania**	**Measles**	**60·9 (35·4–70·2)**	**29·3 (15·6–36·7)**	**20·5 (8·5–35·1)**	**32·9 (16·3–40·7)**
East Asia	All causes	4·4 (2·8–6·2)	2·8 (0·50–5·2)	1·4 (1·1–1·9)	1·8 (1·3–2·4)
East Asia	Diarrheal diseases	40·6 (15·5–64·5)	15·0 (11·5–20·2)	23·8 (-1·4–51·1)	11·0 (6·0–15·0)
East Asia	Lower respiratory infections	50·0 (34·5–67·3)	30·9 (1·4–62·0)	12·7 (7·8–17·4)	21·7 (15·7–27·8)
East Asia	Malaria	34·1 (-56·2–82·3)	34·1 (-56·2–82·3)	--	<0.1 (>-0.1–<0.1)
East Asia	Measles	22·4 (11·3–31·1)	8·0 (3·5–12·6)	6·9 (2·7–12·4)	10·6 (4·4–15·9)
China	All causes	4·1 (2·6–5·8)	2·5 (0·44–4·9)	1·3 (0·98–1·8)	1·6 (1·1–2·1)
China	Diarrheal diseases	40·2 (15·2–64·7)	13·5 (10·9–18·2)	24·4 (-1·3–52·9)	10·4 (6·1–13·6)
China	Lower respiratory infections	48·5 (33·6–66·4)	29·0 (0·99–60·6)	12·3 (7·3–16·9)	20·4 (15·4–25·9)
China	Malaria	0 (0–0)	0 (0–0)	--	0 (0–0)
China	Measles	33·9 (17·3–40·7)	13·0 (6·5–17·0)	8·8 (3·4–16·0)	17·2 (8·2–22·5)
Democratic People's Republic of Korea	All causes	10·2 (5·9–14·6)	7·5 (1·4–13·3)	3·1 (2·0–4·4)	4·9 (2·7–7·6)
Democratic People's Republic of Korea	Diarrheal diseases	56·6 (13·2–79·1)	33·4 (18·5–45·0)	26·1 (-2·4–53·8)	19·5 (5·1–29·5)
Democratic People's Republic of Korea	Lower respiratory infections	63·0 (47·2–73·2)	46·8 (4·0–69·9)	16·6 (10·5–22·3)	32·4 (19·4–43·5)
Democratic People's Republic of Korea	Malaria	34·1 (-56·2–82·3)	34·1 (-56·2–82·3)	--	<0.1 (>-0.1–<0.1)
Democratic People's Republic of Korea	Measles	0 (0–0)	0 (0–0)	0 (0–0)	0 (0–0)
Taiwan	All causes	3·1 (1·7–5·1)	2·0 (0·49–4·3)	1·2 (0·91–1·5)	0·72 (0·55–0·91)
Taiwan	Diarrheal diseases	15·0 (-3·4–34·4)	7·2 (0·15–16·5)	5·8 (-1·5–13·9)	3·3 (-2·0–9·6)
Taiwan	Lower respiratory infections	33·1 (19·9–52·2)	20·9 (0·62–48·2)	8·5 (5·2–11·6)	9·3 (6·7–12·5)
Taiwan	Malaria	0 (0–0)	0 (0–0)	--	0 (0–0)
Taiwan	Measles	5·3 (0·71–11·7)	0·67 (0·16–1·6)	4·1 (-0·39–9·7)	0·67 (0·18–1·6)
Oceania	All causes	23·4 (17·2–28·5)	15·7 (7·2–22·2)	11·8 (9·3–14·4)	11·0 (7·8–14·1)
Oceania	Diarrheal diseases	73·2 (48·9–87·4)	35·3 (25·0–45·7)	47·8 (-2·5–81·4)	31·0 (23·2–37·5)
Oceania	Lower respiratory infections	53·0 (42·5–61·5)	35·9 (2·3–56·8)	20·5 (13·2–27·8)	30·9 (22·8–38·6)
Oceania	Malaria	29·2 (-21·5–75·4)	20·7 (-14·3–53·6)	--	14·0 (-7·9–52·8)
Oceania	Measles	70·9 (43·2–80·4)	34·3 (18·3–43·2)	25·4 (10·6–43·0)	42·9 (21·2–51·4)

American Samoa	All causes	8·6 (5·8–12·6)	5·5 (2·7–9·6)	4·2 (3·2–5·4)	2·2 (1·5–3·1)
American Samoa	Diarrheal diseases	43·8 (18·4–71·3)	13·2 (9·3–18·5)	29·5 (-1·3–64·9)	8·8 (6·5–11·6)
American Samoa	Lower respiratory infections	48·6 (32·2–70·6)	30·0 (0·97–65·7)	13·9 (8·4–19·0)	16·0 (10·6–21·6)
American Samoa	Malaria	0 (0–0)	0 (0–0)	--	0 (0–0)
American Samoa	Measles	29·3 (14·0–36·9)	11·0 (5·3–14·6)	9·4 (3·4–17·1)	12·4 (5·7–16·8)
Cook Islands	All causes	6·9 (4·0–11·9)	4·0 (0·31–10·2)	2·3 (1·5–3·6)	1·8 (1·1–2·7)
Cook Islands	Diarrheal diseases	18·3 (-0·17–36·5)	6·0 (1·6–11·7)	10·5 (-1·2–25·7)	3·5 (-0·77–8·4)
Cook Islands	Lower respiratory infections	39·2 (24·4–63·8)	22·8 (0·59–58·7)	12·6 (7·7–17·2)	10·0 (6·7–13·8)
Cook Islands	Malaria	0 (0–0)	0 (0–0)	--	0 (0–0)
Cook Islands	Measles	20·1 (9·1–26·9)	6·6 (3·1–9·0)	7·7 (2·8–14·3)	7·4 (3·3–10·2)
Fiji	All causes	8·5 (5·5–12·4)	5·4 (1·7–9·7)	4·3 (3·3–5·5)	1·6 (1·1–2·4)
Fiji	Diarrheal diseases	51·5 (20·8–79·4)	15·6 (9·8–22·0)	38·3 (-1·6–76·0)	6·5 (4·4–8·5)
Fiji	Lower respiratory infections	53·1 (33·4–75·6)	34·1 (1·2–70·0)	19·7 (12·4–26·4)	12·1 (8·5–16·2)
Fiji	Malaria	0 (0–0)	0 (0–0)	--	0 (0–0)
Fiji	Measles	31·9 (15·2–41·8)	12·4 (6·0–16·4)	14·1 (5·4–25·6)	9·5 (4·4–12·8)
Guam	All causes	6·6 (3·9–10·5)	3·8 (0·66–8·4)	2·7 (1·9–3·5)	1·6 (0·94–2·4)
Guam	Diarrheal diseases	37·4 (12·5–64·8)	8·8 (5·9–12·5)	27·3 (-0·95–60·0)	5·7 (4·3–7·6)
Guam	Lower respiratory infections	40·1 (24·7–64·2)	23·7 (0·62–59·1)	12·3 (7·6–17·0)	10·8 (6·6–15·1)
Guam	Malaria	0 (0–0)	0 (0–0)	--	0 (0–0)
Guam	Measles	3·7 (-1·1–9·9)	<0.1 (<0.1–0·16)	3·4 (-1·0–9·0)	0·30 (>-0.1–0·90)
Kiribati	All causes	18·3 (13·3–23·8)	13·4 (7·9–18·8)	11·5 (8·0–14·6)	3·9 (2·6–5·7)
Kiribati	Diarrheal diseases	63·3 (39·3–84·7)	24·7 (14·8–35·1)	41·6 (-1·7–80·2)	15·1 (9·9–20·1)
Kiribati	Lower respiratory infections	66·0 (46·3–84·9)	43·9 (2·0–80·3)	20·1 (12·7–26·9)	25·2 (17·6–32·5)
Kiribati	Malaria	0 (0–0)	0 (0–0)	--	0 (0–0)
Kiribati	Measles	42·7 (22·1–51·6)	19·8 (9·9–25·6)	11·7 (4·6–21·0)	19·4 (9·0–25·3)
Marshall Islands	All causes	14·1 (8·0–20·9)	10·9 (2·7–19·1)	2·4 (1·8–3·4)	4·7 (3·0–7·0)
Marshall Islands	Diarrheal diseases	42·8 (35·2–51·1)	26·1 (17·8–35·4)	12·2 (-0·34–33·2)	12·2 (8·5–15·8)
Marshall Islands	Lower respiratory infections	56·0 (28·6–78·7)	43·2 (1·9–76·2)	3·8 (2·2–5·2)	20·9 (13·9–27·5)
Marshall Islands	Malaria	0 (0–0)	0 (0–0)	--	0 (0–0)

Marshall Islands	Measles	35·7 (18·1–43·0)	22·2 (10·9–28·7)	1·6 (0·69–2·8)	16·3 (7·4–21·4)
Micronesia (Federated States of)	All causes	11·9 (8·2–15·7)	8·4 (4·7–11·9)	7·0 (4·8–9·3)	2·9 (1·9–4·0)
Micronesia (Federated States of)	Diarrheal diseases	57·0 (33·3–80·2)	20·5 (12·7–28·9)	36·8 (-1·4–75·0)	14·2 (9·6–18·5)
Micronesia (Federated States of)	Lower respiratory infections	59·6 (41·6–79·1)	38·3 (1·6–73·5)	16·1 (9·9–21·9)	24·3 (17·9–31·1)
Micronesia (Federated States of)	Malaria	0 (0–0)	0 (0–0)	--	0 (0–0)
Micronesia (Federated States of)	Measles	39·2 (19·9–47·4)	16·6 (8·2–21·6)	11·2 (4·3–20·2)	18·2 (8·5–24·0)
Nauru	All causes	14·8 (9·9–21·7)	9·3 (2·3–17·7)	4·2 (3·4–5·3)	6·1 (4·0–8·9)
Nauru	Diarrheal diseases	44·6 (29·2–65·0)	14·7 (8·9–21·6)	22·6 (-0·69–55·7)	15·9 (11·2–20·6)
Nauru	Lower respiratory infections	55·3 (37·9–76·6)	33·4 (1·2–68·4)	8·5 (5·1–11·6)	26·6 (19·0–34·0)
Nauru	Malaria	0 (0–0)	0 (0–0)	--	0 (0–0)
Nauru	Measles	32·4 (16·0–39·2)	11·1 (5·4–14·6)	3·7 (1·5–6·7)	21·2 (9·7–27·6)
Niue	All causes	9·3 (5·8–13·9)	5·9 (1·4–12·0)	3·7 (2·8–5·0)	2·6 (1·7–4·0)
Niue	Diarrheal diseases	40·8 (16·2–68·0)	12·1 (8·7–17·3)	27·4 (-1·3–61·1)	7·8 (6·0–10·7)
Niue	Lower respiratory infections	49·3 (31·9–71·9)	30·0 (0·98–66·0)	14·0 (8·6–18·9)	16·2 (11·7–21·2)
Niue	Malaria	0 (0–0)	0 (0–0)	--	0 (0–0)
Niue	Measles	27·8 (13·0–35·1)	10·3 (4·9–13·8)	8·9 (3·4–16·4)	11·8 (5·2–15·7)
Northern Mariana Islands	All causes	5·0 (2·9–8·3)	2·9 (1·1–5·8)	2·3 (1·6–3·2)	1·3 (0·90–1·8)
Northern Mariana Islands	Diarrheal diseases	37·2 (14·4–64·4)	9·3 (6·3–13·5)	26·5 (-1·0–60·0)	6·3 (4·6–8·3)
Northern Mariana Islands	Lower respiratory infections	36·5 (23·7–57·1)	21·4 (0·60–51·2)	10·6 (6·3–14·7)	11·4 (8·9–14·9)
Northern Mariana Islands	Malaria	0 (0–0)	0 (0–0)	--	0 (0–0)
Northern Mariana Islands	Measles	22·8 (10·6–29·7)	7·8 (3·7–10·5)	8·0 (3·0–14·6)	9·2 (4·2–12·4)
Palau	All causes	7·3 (4·3–11·5)	4·2 (1·3–8·4)	3·4 (2·3–4·9)	1·7 (1·0–2·8)
Palau	Diarrheal diseases	46·9 (19·8–74·9)	12·7 (7·7–18·0)	33·7 (-1·1–71·3)	7·9 (5·4–10·5)
Palau	Lower respiratory infections	48·1 (31·1–70·3)	29·6 (0·94–65·7)	14·6 (9·1–19·9)	14·1 (8·2–19·7)
Palau	Malaria	0 (0–0)	0 (0–0)	--	0 (0–0)
Palau	Measles	27·1 (12·8–34·8)	10·1 (4·8–13·4)	9·3 (3·5–16·9)	10·9 (4·9–14·8)
Papua New Guinea	All causes	24·5 (18·0–30·0)	16·4 (7·5–23·2)	12·4 (9·6–15·2)	11·7 (8·4–15·0)
Papua New Guinea	Diarrheal diseases	74·2 (50·3–87·8)	36·2 (25·7–46·7)	48·4 (-2·6–82·0)	32·1 (24·3–38·4)
Papua New Guinea	Lower respiratory infections	53·2 (42·9–62·0)	36·1 (2·3–56·8)	20·7 (13·3–28·1)	31·6 (23·3–39·4)

Papua New Guinea	Malaria	29·2 (-21·6–75·5)	20·7 (-14·3–53·6)	--	14·2 (-8·0–53·1)
Papua New Guinea	Measles	71·9 (43·9–81·5)	34·9 (18·6–43·9)	25·8 (10·8–43·6)	43·8 (21·6–52·3)
Samoa	All causes	10·2 (6·3–14·8)	5·9 (1·6–12·3)	4·8 (3·4–6·7)	2·5 (1·5–4·0)
Samoa	Diarrheal diseases	47·8 (18·8–76·0)	11·9 (7·8–17·4)	35·6 (-1·5–71·9)	7·8 (5·5–10·4)
Samoa	Lower respiratory infections	50·5 (35·7–71·9)	28·8 (0·95–65·5)	19·1 (12·1–25·5)	14·4 (9·8–19·0)
Samoa	Malaria	0 (0–0)	0 (0–0)	--	0 (0–0)
Samoa	Measles	28·4 (13·5–35·7)	9·7 (4·7–12·7)	11·5 (4·4–20·9)	10·5 (4·7–14·2)
Solomon Islands	All causes	11·8 (7·8–14·8)	7·8 (3·9–11·3)	6·1 (4·5–7·6)	4·2 (2·8–5·8)
Solomon Islands	Diarrheal diseases	60·4 (34·6–78·0)	24·9 (17·4–33·0)	40·0 (-1·9–72·2)	19·4 (14·3–24·7)
Solomon Islands	Lower respiratory infections	35·2 (25·9–43·7)	22·9 (1·1–40·4)	12·2 (7·8–16·9)	16·6 (10·1–22·1)
Solomon Islands	Malaria	21·9 (-15·9–60·3)	20·7 (-15·0–55·0)	--	1·9 (-0·75–10·4)
Solomon Islands	Measles	58·8 (32·1–70·2)	26·0 (13·1–33·6)	20·8 (8·3–36·1)	31·0 (14·4–39·2)
Tokelau	All causes	9·7 (5·9–14·4)	6·1 (1·3–12·3)	3·8 (2·8–5·1)	2·8 (1·8–4·3)
Tokelau	Diarrheal diseases	44·6 (19·5–71·6)	13·5 (9·3–19·5)	30·0 (-1·2–65·6)	8·9 (6·3–11·9)
Tokelau	Lower respiratory infections	49·6 (32·4–71·4)	30·6 (1·0–65·4)	14·1 (8·6–19·3)	16·7 (12·2–21·8)
Tokelau	Malaria	0 (0–0)	0 (0–0)	--	0 (0–0)
Tokelau	Measles	29·1 (13·9–36·3)	11·0 (5·3–14·6)	9·2 (3·5–16·7)	12·4 (5·7–16·7)
Tonga	All causes	6·1 (3·7–10·2)	4·0 (1·4–8·8)	3·0 (2·2–4·1)	1·1 (0·72–1·8)
Tonga	Diarrheal diseases	27·3 (6·0–53·5)	5·4 (4·2–8·1)	20·6 (-0·87–49·1)	3·5 (2·0–5·0)
Tonga	Lower respiratory infections	35·6 (21·8–61·5)	20·1 (0·47–55·1)	11·5 (6·6–16·5)	8·8 (6·3–12·1)
Tonga	Malaria	0 (0–0)	0 (0–0)	--	0 (0–0)
Tonga	Measles	16·2 (6·9–23·2)	4·6 (2·1–6·3)	7·5 (2·6–14·6)	5·1 (2·3–7·1)
Tuvalu	All causes	12·3 (7·9–18·1)	7·1 (2·1–14·0)	4·8 (3·5–6·6)	4·3 (2·5–6·6)
Tuvalu	Diarrheal diseases	47·5 (23·2–75·7)	12·7 (7·4–19·2)	32·0 (-1·2–71·4)	12·1 (8·1–16·4)
Tuvalu	Lower respiratory infections	48·6 (34·2–68·1)	28·1 (0·94–62·0)	12·0 (7·1–16·7)	20·3 (12·5–27·3)
Tuvalu	Malaria	0 (0–0)	0 (0–0)	--	0 (0–0)
Tuvalu	Measles	27·6 (13·0–34·3)	9·2 (4·3–12·3)	8·3 (3·2–15·8)	13·5 (6·1–18·1)
Vanuatu	All causes	18·6 (13·0–24·4)	10·5 (4·4–16·4)	7·6 (5·1–10·3)	7·4 (5·2–9·7)
Vanuatu	Diarrheal diseases	59·9 (35·3–79·9)	22·3 (14·1–30·7)	38·9 (-1·6–74·7)	17·7 (12·5–22·3)

Vanuatu	Lower respiratory infections	57·0 (41·6–71·6)	36·7 (1·6–67·7)	16·8 (10·4–22·3)	24·7 (16·1–32·5)
Vanuatu	Malaria	23·8 (-16·9–64·9)	23·8 (-16·9–64·9)	--	<0.1 (>-0.1–<0.1)
Vanuatu	Measles	47·7 (25·2–56·9)	19·8 (10·0–25·3)	14·1 (5·6–24·7)	25·0 (11·9–32·0)
Southeast Asia	All causes	12·2 (9·1–15·3)	7·8 (3·4–11·2)	6·2 (4·3–8·2)	4·9 (3·5–6·5)
Southeast Asia	Diarrheal diseases	69·5 (45·8–84·8)	31·4 (21·4–40·9)	45·9 (-2·3–79·0)	24·0 (17·1–29·5)
Southeast Asia	Lower respiratory infections	58·0 (44·9–66·8)	39·2 (2·3–63·1)	20·6 (13·8–26·4)	28·8 (19·4–35·9)
Southeast Asia	Malaria	22·0 (-14·4–61·4)	18·1 (-11·0–48·6)	--	6·2 (-2·6–27·5)
Southeast Asia	Measles	58·3 (33·4–67·5)	28·0 (14·8–35·1)	19·2 (7·9–33·0)	30·3 (14·9–37·8)
Cambodia	All causes	15·1 (10·6–20·0)	10·1 (2·3–16·6)	7·1 (5·3–9·4)	6·6 (4·1–9·7)
Cambodia	Diarrheal diseases	71·3 (43·6–86·6)	31·2 (21·2–40·6)	49·2 (-2·4–81·8)	22·9 (16·7–28·7)
Cambodia	Lower respiratory infections	56·2 (44·5–65·5)	37·9 (2·2–61·0)	21·4 (14·1–28·4)	28·0 (18·9–35·3)
Cambodia	Malaria	26·9 (-21·4–69·3)	22·4 (-18·1–56·1)	--	7·5 (-2·8–35·4)
Cambodia	Measles	63·9 (37·5–73·6)	31·6 (16·9–39·3)	22·5 (9·3–38·6)	33·6 (17·1–42·0)
Indonesia	All causes	12·8 (9·2–16·5)	8·2 (4·3–11·2)	6·9 (4·3–9·7)	5·4 (3·8–7·3)
Indonesia	Diarrheal diseases	70·3 (48·9–83·9)	32·8 (22·1–42·7)	45·9 (-2·3–78·2)	27·1 (19·3–33·0)
Indonesia	Lower respiratory infections	61·9 (50·0–70·5)	42·2 (2·7–66·9)	21·8 (14·4–28·5)	34·1 (22·8–42·8)
Indonesia	Malaria	27·1 (-19·6–71·4)	22·2 (-16·1–57·9)	--	8·2 (-3·8–36·0)
Indonesia	Measles	63·0 (37·1–72·7)	30·6 (16·4–38·5)	21·7 (8·9–36·7)	34·1 (17·0–42·3)
Lao People's Democratic Republic	All causes	17·5 (11·7–22·4)	11·1 (3·6–18·0)	8·3 (5·6–10·9)	6·9 (4·6–9·7)
Lao People's Democratic Republic	Diarrheal diseases	71·6 (43·6–89·2)	30·0 (19·4–40·1)	49·1 (-2·2–85·1)	21·6 (15·0–27·4)
Lao People's Democratic Republic	Lower respiratory infections	64·7 (49·8–77·0)	43·0 (2·3–73·2)	23·6 (15·3–30·7)	29·6 (19·4–38·3)
Lao People's Democratic Republic	Malaria	23·1 (-15·7–63·1)	18·8 (-13·0–48·8)	--	6·7 (-2·5–31·5)
Lao People's Democratic Republic	Measles	61·5 (35·2–71·2)	30·4 (16·0–38·2)	20·0 (8·4–34·1)	32·6 (15·5–40·4)
Malaysia	All causes	6·5 (4·9–8·7)	4·5 (2·2–6·8)	3·7 (3·0–4·5)	2·0 (1·5–2·7)
Malaysia	Diarrheal diseases	46·3 (16·4–68·0)	19·5 (14·6–25·9)	29·7 (-2·3–56·0)	11·1 (6·1–15·2)
Malaysia	Lower respiratory infections	51·3 (38·2–63·7)	33·3 (1·7–58·4)	18·1 (11·4–24·6)	23·3 (19·4–27·9)
Malaysia	Malaria	18·6 (-10·3–55·0)	15·0 (-8·0–43·3)	--	5·2 (-2·0–21·7)
Malaysia	Measles	50·3 (26·3–60·5)	22·0 (10·7–28·1)	20·7 (8·4–36·2)	21·0 (9·5–27·2)
Maldives	All causes	6·2 (4·3–7·9)	4·1 (2·0–5·8)	3·4 (2·1–4·6)	2·1 (1·5–2·9)

Maldives	Diarrheal diseases	59·3 (31·3–77·4)	27·7 (21·8–35·4)	37·2 (-2·4–66·4)	18·1 (13·2–23·1)
Maldives	Lower respiratory infections	50·6 (38·6–59·4)	34·6 (2·0–55·7)	17·0 (10·7–22·3)	24·1 (16·6–30·4)
Maldives	Malaria	0 (0–0)	0 (0–0)	--	0 (0–0)
Maldives	Measles	53·4 (29·3–64·0)	25·2 (13·0–32·1)	19·6 (8·1–33·8)	23·5 (11·2–30·3)
Mauritius	All causes	5·2 (3·7–6·5)	3·1 (1·6–4·6)	3·4 (2·3–4·0)	1·2 (0·87–1·6)
Mauritius	Diarrheal diseases	56·0 (21·8–76·4)	19·2 (14·5–25·1)	42·0 (-2·9–69·2)	10·6 (8·0–13·6)
Mauritius	Lower respiratory infections	47·1 (36·9–57·7)	29·1 (1·3–54·6)	21·8 (15·0–28·0)	16·0 (10·6–21·4)
Mauritius	Malaria	0 (0–0)	0 (0–0)	--	0 (0–0)
Mauritius	Measles	9·4 (-3·4–24·3)	<0.1 (<0.1–<0.1)	8·9 (-3·1–22·9)	0·60 (-0·23–2·1)
Myanmar	All causes	11·6 (7·7–15·7)	7·1 (2·3–11·9)	5·1 (3·6–7·4)	4·4 (2·8–6·5)
Myanmar	Diarrheal diseases	65·5 (37·6–83·9)	27·0 (17·7–35·9)	44·3 (-1·8–78·8)	17·9 (12·4–23·5)
Myanmar	Lower respiratory infections	49·0 (35·6–61·1)	32·7 (1·7–57·7)	17·4 (11·2–23·7)	20·3 (12·5–27·9)
Myanmar	Malaria	20·3 (-12·3–57·0)	16·7 (-9·7–44·3)	--	5·6 (-2·3–25·5)
Myanmar	Measles	56·5 (31·8–65·8)	27·5 (14·5–34·7)	17·4 (7·1–30·4)	28·6 (13·8–36·4)
Philippines	All causes	13·5 (10·1–16·6)	8·7 (3·5–12·1)	6·9 (4·8–8·9)	5·1 (3·6–6·8)
Philippines	Diarrheal diseases	73·4 (47·8–89·8)	33·0 (22·5–43·7)	49·4 (-2·8–84·7)	22·3 (15·7–27·9)
Philippines	Lower respiratory infections	64·3 (50·3–74·3)	43·8 (2·5–70·4)	22·6 (14·8–29·4)	30·8 (22·2–38·0)
Philippines	Malaria	27·3 (-21·9–72·7)	22·8 (-18·2–57·6)	--	7·7 (-3·9–37·8)
Philippines	Measles	60·0 (34·3–69·0)	30·0 (15·6–37·0)	19·9 (8·4–33·9)	30·2 (14·7–37·6)
Seychelles	All causes	7·2 (4·5–10·3)	4·2 (0·98–8·0)	3·1 (2·3–3·9)	2·2 (1·7–3·2)
Seychelles	Diarrheal diseases	46·4 (16·6–70·7)	13·8 (10·2–18·2)	32·3 (-1·8–61·9)	9·2 (6·8–12·0)
Seychelles	Lower respiratory infections	42·4 (29·5–56·4)	25·8 (1·0–51·6)	13·4 (8·5–17·9)	15·7 (11·0–20·4)
Seychelles	Malaria	0 (0–0)	0 (0–0)	--	0 (0–0)
Seychelles	Measles	33·1 (16·9–39·2)	12·6 (6·3–16·2)	11·5 (4·8–20·1)	13·9 (6·5–18·3)
Sri Lanka	All causes	5·6 (4·2–6·9)	4·4 (2·7–5·8)	3·9 (3·1–4·8)	1·3 (0·94–1·7)
Sri Lanka	Diarrheal diseases	52·7 (18·0–72·9)	26·5 (18·6–35·4)	33·4 (-3·6–57·7)	13·7 (5·9–20·4)
Sri Lanka	Lower respiratory infections	53·3 (41·1–62·4)	37·2 (2·3–58·6)	22·2 (14·8–29·6)	23·3 (16·0–30·5)
Sri Lanka	Malaria	0 (0–0)	0 (0–0)	--	0 (0–0)
Sri Lanka	Measles	60·0 (34·8–69·8)	30·5 (16·4–38·0)	27·0 (11·9–44·9)	23·3 (11·3–30·1)

Thailand	All causes	8·3 (6·2–10·6)	4·9 (2·2–7·3)	4·0 (2·8–5·1)	3·0 (2·3–3·9)
Thailand	Diarrheal diseases	50·4 (23·0–71·9)	19·1 (15·0–25·2)	32·5 (-1·8–61·7)	14·1 (9·4–18·2)
Thailand	Lower respiratory infections	57·6 (41·8–73·5)	36·2 (1·7–66·6)	18·3 (11·0–25·6)	26·5 (19·3–34·6)
Thailand	Malaria	19·1 (-10·3–55·7)	18·7 (-10·1–52·3)	--	0·51 (-0·11–3·1)
Thailand	Measles	41·3 (20·8–50·7)	15·6 (7·7–20·3)	14·4 (5·6–25·8)	19·3 (8·8–25·2)
Timor-Leste	All causes	21·8 (16·4–26·8)	16·3 (5·9–24·1)	13·0 (9·9–16·5)	9·6 (6·6–13·4)
Timor-Leste	Diarrheal diseases	82·5 (58·1–93·3)	44·2 (32·6–55·5)	59·3 (-4·5–89·2)	31·8 (23·4–38·1)
Timor-Leste	Lower respiratory infections	76·9 (65·6–84·5)	54·5 (3·8–80·9)	36·4 (25·6–45·7)	43·3 (31·5–52·1)
Timor-Leste	Malaria	33·1 (-35·6–80·2)	31·5 (-32·7–76·9)	--	3·0 (-1·5–17·8)
Timor-Leste	Measles	78·6 (51·2–88·4)	44·6 (25·0–54·3)	37·7 (17·6–58·4)	42·1 (20·9–51·1)
Viet Nam	All causes	5·7 (3·7–7·4)	3·4 (1·0–5·7)	2·1 (1·4–3·0)	2·5 (1·6–3·8)
Viet Nam	Diarrheal diseases	36·6 (0·17–60·2)	17·4 (5·1–29·9)	18·1 (-2·4–38·1)	10·6 (-1·1–20·4)
Viet Nam	Lower respiratory infections	40·9 (26·2–53·9)	25·8 (1·1–47·9)	13·2 (6·9–20·4)	19·3 (12·2–28·1)
Viet Nam	Malaria	21·1 (-14·0–59·5)	18·1 (-11·5–47·8)	--	4·3 (-1·7–22·1)
Viet Nam	Measles	50·8 (27·0–59·0)	22·3 (11·0–28·4)	16·1 (6·7–28·5)	25·7 (12·1–32·5)
**Sub-Saharan Africa**	**All causes**	**22·5 (10·9–31·7)**	**14·6 (5·5–21·7)**	**10·4 (5·9–15·0)**	**9·5 (6·0–13·9)**
**Sub-Saharan Africa**	**Diarrheal diseases**	**76·5 (54·2–89·5)**	**36·6 (24·5–47·9)**	**52·4 (-3·0–86·0)**	**26·6 (18·9–32·7)**
**Sub-Saharan Africa**	**Lower respiratory infections**	**64·8 (52·2–73·6)**	**44·4 (2·9–69·3)**	**22·8 (15·1–30·0)**	**36·9 (30·2–42·9)**
**Sub-Saharan Africa**	**Malaria**	**26·2 (-17·7–69·3)**	**19·0 (-11·5–48·7)**	**--**	**12·1 (-6·2–44·6)**
**Sub-Saharan Africa**	**Measles**	**66·1 (39·4–75·7)**	**33·8 (18·1–42·1)**	**24·3 (10·4–40·2)**	**35·3 (17·3–43·6)**
Central Sub-Saharan Africa	All causes	22·8 (9·3–35·8)	14·4 (3·3–23·8)	7·9 (5·0–11·9)	10·8 (5·8–18·3)
Central Sub-Saharan Africa	Diarrheal diseases	76·3 (53·0–90·6)	34·8 (22·6–46·3)	50·2 (-2·4–86·1)	27·6 (19·8–33·9)
Central Sub-Saharan Africa	Lower respiratory infections	69·4 (55·0–80·0)	46·8 (2·8–74·6)	22·5 (14·0–30·4)	39·7 (31·5–47·3)
Central Sub-Saharan Africa	Malaria	26·7 (-17·4–71·3)	18·5 (-10·8–48·0)	--	13·1 (-6·6–48·1)
Central Sub-Saharan Africa	Measles	65·3 (38·2–75·4)	31·5 (16·6–39·7)	21·6 (8·7–37·0)	37·2 (18·3–45·6)
Angola	All causes	19·2 (9·6–27·4)	12·3 (4·3–18·7)	7·2 (3·9–11·4)	8·8 (5·5–12·1)
Angola	Diarrheal diseases	75·5 (55·7–89·6)	35·8 (23·2–47·5)	46·3 (-2·1–83·6)	28·9 (20·7–35·5)
Angola	Lower respiratory infections	71·0 (55·3–81·1)	48·5 (3·0–76·1)	19·1 (11·9–25·6)	41·2 (32·3–48·5)
Angola	Malaria	26·4 (-16·9–71·7)	19·0 (-11·1–49·1)	--	11·9 (-5·6–45·9)

Angola	Measles	62·3 (35·9–71·7)	30·5 (16·1–38·7)	16·4 (6·6–28·6)	36·3 (17·9–44·7)
Central African Republic	All causes	31·6 (18·0–41·7)	18·3 (9·0–25·3)	15·6 (5·0–26·2)	13·8 (9·6–17·2)
Central African Republic	Diarrheal diseases	77·0 (54·6–91·0)	35·7 (23·0–47·5)	50·6 (-2·4–86·8)	28·3 (20·2–34·6)
Central African Republic	Lower respiratory infections	69·0 (55·1–78·0)	47·2 (3·0–72·4)	21·7 (13·6–29·1)	40·6 (33·8–47·0)
Central African Republic	Malaria	27·5 (-18·9–71·8)	19·4 (-11·8–49·7)	--	13·5 (-7·2–48·7)
Central African Republic	Measles	67·2 (40·1–76·7)	34·2 (18·3–42·9)	20·7 (8·3–35·6)	38·9 (19·3–47·3)
Congo	All causes	16·2 (6·7–25·3)	9·4 (3·1–16·0)	7·5 (3·2–13·0)	5·8 (3·3–9·3)
Congo	Diarrheal diseases	70·4 (43·3–88·3)	27·2 (16·5–37·6)	49·0 (-2·2–84·8)	20·1 (13·8–25·9)
Congo	Lower respiratory infections	60·5 (47·4–72·9)	38·6 (1·9–67·0)	20·4 (13·3–27·8)	29·2 (22·8–36·2)
Congo	Malaria	19·5 (-10·4–58·1)	14·1 (-7·5–39·0)	--	7·7 (-2·9–31·8)
Congo	Measles	52·2 (27·4–63·1)	21·8 (10·9–28·1)	18·3 (7·3–32·0)	26·1 (12·1–33·6)
Democratic Republic of the Congo	All causes	23·3 (7·6–40·2)	15·0 (2·1–26·9)	7·2 (4·6–10·7)	11·4 (5·3–21·5)
Democratic Republic of the Congo	Diarrheal diseases	77·1 (51·5–91·4)	34·3 (22·7–46·2)	52·9 (-2·6–87·7)	27·1 (19·7–33·6)
Democratic Republic of the Congo	Lower respiratory infections	69·2 (54·6–81·1)	46·5 (2·7–74·5)	23·8 (14·6–32·3)	39·5 (30·4–48·1)
Democratic Republic of the Congo	Malaria	27·0 (-17·6–71·7)	18·6 (-10·8–48·1)	--	13·5 (-6·8–49·3)
Democratic Republic of the Congo	Measles	66·0 (38·6–76·3)	31·8 (16·7–40·0)	22·5 (9·1–38·3)	37·5 (18·4–45·9)
Equatorial Guinea	All causes	10·5 (2·6–21·7)	6·0 (0·26–13·1)	2·9 (1·5–4·7)	4·4 (1·6–10·6)
Equatorial Guinea	Diarrheal diseases	60·1 (31·9–83·2)	17·9 (10·5–25·8)	40·7 (-1·4–78·7)	17·5 (11·9–22·8)
Equatorial Guinea	Lower respiratory infections	52·0 (39·7–67·6)	30·1 (1·1–62·0)	15·4 (9·3–21·4)	24·4 (16·6–31·8)
Equatorial Guinea	Malaria	14·2 (-6·9–45·9)	9·2 (-4·2–26·7)	--	6·4 (-2·5–27·2)
Equatorial Guinea	Measles	44·1 (22·1–54·6)	14·1 (6·7–18·6)	13·7 (5·1–25·4)	24·9 (11·9–32·1)
Gabon	All causes	10·7 (5·0–17·5)	5·6 (1·8–10·6)	4·1 (1·9–7·2)	4·1 (2·5–6·5)
Gabon	Diarrheal diseases	56·5 (31·7–81·1)	17·3 (9·9–25·6)	37·2 (-1·2–76·5)	15·9 (10·7–21·0)
Gabon	Lower respiratory infections	48·7 (35·9–65·3)	28·6 (1·1–58·6)	12·1 (7·3–16·6)	23·0 (17·3–29·4)
Gabon	Malaria	12·5 (-5·8–40·5)	8·7 (-4·1–25·8)	--	4·7 (-1·8–20·9)
Gabon	Measles	38·2 (18·7–46·7)	13·4 (6·4–17·8)	10·4 (3·9–19·1)	20·7 (9·6–27·2)
Eastern Sub-Saharan Africa	All causes	18·6 (11·3–25·3)	11·5 (5·6–16·5)	8·6 (5·0–12·1)	7·9 (5·7–10·4)
Eastern Sub-Saharan Africa	Diarrheal diseases	73·4 (52·2–86·9)	34·3 (22·8–45·0)	48·7 (-2·6–83·1)	26·8 (19·2–32·9)
Eastern Sub-Saharan Africa	Lower respiratory infections	61·4 (49·2–69·9)	41·2 (2·5–65·7)	20·0 (13·2–26·1)	34·2 (26·1–41·0)

Eastern Sub-Saharan Africa	Malaria	23·9 (-15·1–65·8)	16·8 (-9·7–44·2)	--	11·1 (-5·3–42·4)
Eastern Sub-Saharan Africa	Measles	64·5 (38·0–74·2)	32·1 (17·0–40·3)	22·7 (9·7–37·9)	34·8 (17·1–43·2)
Burundi	All causes	24·9 (5·6–39·2)	17·4 (2·1–29·1)	7·7 (4·2–13·3)	12·7 (4·3–23·2)
Burundi	Diarrheal diseases	81·1 (66·9–91·1)	44·0 (30·3–56·4)	47·1 (-2·4–84·3)	37·9 (28·7–44·5)
Burundi	Lower respiratory infections	72·8 (60·1–80·7)	52·5 (3·9–76·7)	19·3 (12·3–26·0)	47·3 (37·2–54·7)
Burundi	Malaria	32·2 (-25·8–78·4)	22·9 (-15·7–55·5)	--	16·9 (-9·2–57·9)
Burundi	Measles	72·8 (45·5–80·5)	40·1 (22·5–49·0)	17·5 (7·1–30·3)	46·8 (23·9–55·4)
Comoros	All causes	19·3 (13·9–25·0)	11·6 (5·8–15·9)	11·4 (6·0–16·3)	7·8 (5·6–10·4)
Comoros	Diarrheal diseases	78·0 (53·6–91·0)	34·3 (22·3–46·8)	55·7 (-3·1–88·2)	27·5 (19·7–34·4)
Comoros	Lower respiratory infections	65·8 (54·3–73·8)	43·4 (2·4–70·2)	27·7 (19·1–35·1)	34·4 (24·1–42·5)
Comoros	Malaria	25·8 (-19·0–70·1)	20·5 (-13·4–56·0)	--	8·2 (-4·3–44·4)
Comoros	Measles	65·7 (38·0–77·6)	28·8 (14·8–37·2)	28·0 (11·5–46·5)	35·1 (17·0–43·9)
Djibouti	All causes	21·0 (14·3–26·8)	13·7 (8·8–17·8)	15·3 (7·8–21·4)	6·4 (4·3–8·8)
Djibouti	Diarrheal diseases	82·2 (54·6–92·4)	41·1 (28·1–52·8)	64·0 (-4·8–90·4)	24·8 (17·6–31·1)
Djibouti	Lower respiratory infections	67·0 (57·2–73·9)	45·9 (2·9–71·1)	38·1 (28·4–46·5)	30·7 (20·7–39·2)
Djibouti	Malaria	28·4 (-22·9–74·2)	22·8 (-16·9–58·6)	--	9·8 (-4·3–45·0)
Djibouti	Measles	75·5 (46·7–87·3)	38·6 (20·5–48·3)	43·3 (20·3–64·7)	33·6 (15·8–42·5)
Eritrea	All causes	27·5 (21·2–32·7)	19·4 (11·7–23·8)	17·5 (11·1–22·2)	10·1 (7·7–12·7)
Eritrea	Diarrheal diseases	83·8 (61·3–93·9)	44·4 (31·1–56·8)	60·5 (-4·8–91·0)	27·3 (19·5–33·9)
Eritrea	Lower respiratory infections	75·7 (63·9–82·3)	54·5 (4·0–78·5)	33·0 (22·4–42·4)	41·5 (35·3–47·1)
Eritrea	Malaria	32·7 (-25·9–80·4)	25·8 (-18·5–62·5)	--	13·3 (-7·4–49·6)
Eritrea	Measles	74·6 (47·7–84·4)	41·9 (23·8–50·9)	32·2 (14·6–50·9)	37·5 (18·7–45·5)
Ethiopia	All causes	14·1 (9·1–18·4)	9·0 (4·6–12·9)	7·1 (4·6–9·8)	6·0 (4·0–7·7)
Ethiopia	Diarrheal diseases	75·0 (52·8–87·3)	35·8 (23·8–46·5)	51·4 (-3·0–83·4)	27·5 (19·6–33·1)
Ethiopia	Lower respiratory infections	66·0 (55·0–73·8)	45·3 (3·0–69·2)	25·6 (17·1–33·2)	36·3 (27·6–43·7)
Ethiopia	Malaria	24·5 (-16·6–65·1)	17·8 (-10·8–45·2)	--	11·0 (-5·4–41·7)
Ethiopia	Measles	66·5 (39·8–76·4)	32·9 (17·8–41·3)	24·5 (10·6–40·4)	36·3 (17·9–44·4)
Kenya	All causes	17·9 (11·1–23·7)	10·3 (4·5–15·3)	8·0 (3·6–12·1)	7·7 (5·9–9·8)
Kenya	Diarrheal diseases	67·7 (46·1–83·4)	28·4 (18·0–38·8)	42·7 (-2·0–78·2)	24·4 (17·3–30·1)

Kenya	Lower respiratory infections	52·0 (40·4–61·9)	33·9 (1·8–58·7)	14·8 (9·6–19·5)	27·8 (19·3–34·4)
Kenya	Malaria	20·4 (-12·2–58·7)	14·8 (-8·6–40·0)	--	8·2 (-3·7–34·2)
Kenya	Measles	56·3 (31·8–65·7)	28·0 (14·8–35·5)	17·1 (7·1–29·3)	28·6 (13·7–36·2)
Madagascar	All causes	26·4 (17·1–34·2)	16·6 (8·7–22·0)	12·6 (5·4–19·5)	11·7 (9·2–14·3)
Madagascar	Diarrheal diseases	78·2 (60·0–90·8)	39·9 (27·2–52·5)	48·6 (-2·4–85·4)	31·7 (23·0–38·6)
Madagascar	Lower respiratory infections	57·3 (43·9–65·7)	40·6 (2·5–61·8)	17·3 (10·8–23·8)	33·9 (26·2–41·0)
Madagascar	Malaria	28·2 (-19·5–73·5)	20·8 (-13·3–52·8)	--	12·7 (-6·4–48·5)
Madagascar	Measles	68·1 (41·1–76·6)	36·4 (19·9–44·9)	20·0 (8·3–34·3)	39·2 (19·5–47·6)
Malawi	All causes	14·2 (7·1–22·3)	7·6 (3·5–12·3)	5·3 (2·0–9·6)	6·4 (4·0–10·4)
Malawi	Diarrheal diseases	62·5 (41·2–82·3)	23·2 (13·8–32·6)	36·6 (-1·2–76·4)	23·9 (16·5–30·1)
Malawi	Lower respiratory infections	59·0 (44·3–73·8)	36·6 (1·7–67·1)	12·0 (6·8–17·4)	34·6 (25·7–43·6)
Malawi	Malaria	19·9 (-11·1–59·4)	13·2 (-7·0–37·8)	--	9·3 (-4·0–37·2)
Malawi	Measles	51·2 (27·7–59·9)	20·7 (10·4–26·7)	9·9 (3·9–18·3)	32·4 (15·8–40·5)
Mozambique	All causes	18·3 (10·1–26·3)	10·9 (4·1–16·8)	7·6 (3·7–11·3)	8·0 (5·8–11·1)
Mozambique	Diarrheal diseases	72·5 (51·8–89·6)	30·1 (18·2–41·7)	45·0 (-1·8–84·8)	27·8 (19·0–34·9)
Mozambique	Lower respiratory infections	69·1 (53·1–81·1)	45·0 (2·4–77·1)	19·4 (12·5–25·7)	37·7 (26·8–46·9)
Mozambique	Malaria	22·6 (-13·1–64·3)	15·5 (-8·4–42·3)	--	10·4 (-4·5–39·7)
Mozambique	Measles	56·9 (31·8–66·1)	24·4 (12·4–31·3)	14·7 (5·6–26·5)	34·3 (17·1–42·8)
Rwanda	All causes	16·6 (9·3–24·0)	9·5 (3·1–15·7)	5·3 (3·0–8·8)	8·6 (5·8–11·1)
Rwanda	Diarrheal diseases	65·3 (47·4–83·2)	24·7 (14·9–35·1)	35·7 (-1·2–75·1)	28·0 (19·9–34·6)
Rwanda	Lower respiratory infections	65·5 (52·2–77·7)	40·6 (1·9–71·6)	12·1 (7·3–16·6)	40·5 (31·0–47·4)
Rwanda	Malaria	21·7 (-12·7–63·7)	13·2 (-6·9–36·4)	--	11·8 (-5·8–45·5)
Rwanda	Measles	54·6 (29·7–63·2)	21·2 (10·8–27·3)	9·5 (3·6–17·2)	36·9 (18·2–45·2)
Somalia	All causes	30·1 (21·3–37·0)	17·4 (12·5–21·4)	17·2 (8·4–25·1)	11·9 (7·8–15·5)
Somalia	Diarrheal diseases	70·8 (50·6–81·5)	36·1 (24·9–47·0)	50·7 (-3·2–79·3)	23·4 (16·7–29·6)
Somalia	Lower respiratory infections	43·8 (33·8–51·6)	30·5 (1·9–47·9)	18·4 (12·1–25·0)	23·2 (17·3–29·3)
Somalia	Malaria	25·6 (-16·0–69·3)	19·7 (-11·2–51·5)	--	10·0 (-4·5–39·3)
Somalia	Measles	67·9 (40·2–78·3)	35·8 (18·7–44·9)	27·9 (12·2–45·5)	33·2 (16·1–41·7)
South Sudan	All causes	32·1 (18·6–42·5)	20·4 (10·9–27·5)	20·7 (9·3–30·3)	11·2 (7·6–14·9)

South Sudan	Diarrheal diseases	84·8 (58·7–94·9)	42·9 (29·7–55·1)	64·9 (-4·8–93·2)	26·6 (19·1–33·2)
South Sudan	Lower respiratory infections	75·9 (65·1–82·6)	52·8 (3·4–79·0)	40·1 (29·5–48·8)	37·4 (28·2–45·4)
South Sudan	Malaria	28·2 (-18·6–73·6)	21·4 (-13·0–54·3)	--	12·0 (-5·1–46·4)
South Sudan	Measles	76·0 (47·6–86·8)	39·5 (21·4–48·6)	42·5 (20·0–64·5)	35·0 (16·8–43·6)
Uganda	All causes	13·3 (3·3–25·7)	8·6 (1·0–16·6)	4·2 (2·3–6·9)	5·4 (2·3–11·5)
Uganda	Diarrheal diseases	68·2 (46·7–86·1)	27·3 (17·1–38·2)	42·6 (-1·9–80·1)	23·4 (16·5–29·8)
Uganda	Lower respiratory infections	72·8 (56·2–86·6)	47·0 (2·3–80·2)	19·9 (12·7–26·3)	38·1 (28·2–46·3)
Uganda	Malaria	20·6 (-11·4–61·3)	14·6 (-7·7–40·4)	--	8·7 (-3·6–36·3)
Uganda	Measles	52·9 (28·6–61·8)	22·6 (11·5–28·9)	13·8 (5·4–24·4)	30·1 (14·5–37·9)
United Republic of Tanzania	All causes	12·7 (7·5–18·4)	7·9 (3·0–12·5)	4·8 (3·4–6·4)	5·6 (4·0–8·2)
United Republic of Tanzania	Diarrheal diseases	66·1 (45·3–84·2)	25·4 (15·5–35·3)	40·7 (-1·6–78·7)	24·6 (17·6–30·7)
United Republic of Tanzania	Lower respiratory infections	50·1 (36·4–60·9)	31·9 (1·4–56·4)	12·6 (7·7–17·7)	27·9 (19·5–35·4)
United Republic of Tanzania	Malaria	21·2 (-12·1–61·6)	14·3 (-7·8–39·4)	--	9·9 (-4·1–38·8)
United Republic of Tanzania	Measles	55·1 (30·0–63·8)	23·2 (11·6–29·3)	12·7 (4·9–22·8)	33·8 (16·5–42·2)
Zambia	All causes	17·7 (10·0–25·4)	10·0 (4·4–16·5)	7·8 (4·4–12·0)	7·9 (5·3–10·7)
Zambia	Diarrheal diseases	69·3 (46·1–88·0)	24·6 (15·0–34·7)	44·0 (-1·6–83·4)	27·2 (19·3–33·5)
Zambia	Lower respiratory infections	65·2 (53·6–77·3)	39·8 (1·8–71·2)	17·2 (10·7–23·6)	38·4 (29·1–45·3)
Zambia	Malaria	22·2 (-12·9–65·6)	13·6 (-7·1–38·7)	--	12·0 (-5·9–45·8)
Zambia	Measles	56·8 (30·5–65·4)	21·4 (10·2–27·6)	15·7 (5·9–28·1)	35·8 (17·3–43·9)
Southern Sub-Saharan Africa	All causes	21·0 (16·2–25·5)	13·2 (7·8–17·7)	11·1 (7·3–14·8)	7·6 (5·4–9·9)
Southern Sub-Saharan Africa	Diarrheal diseases	62·6 (42·0–82·5)	21·7 (12·5–30·6)	35·8 (-1·2–76·0)	25·1 (17·5–31·3)
Southern Sub-Saharan Africa	Lower respiratory infections	64·8 (50·2–79·0)	39·6 (1·7–73·8)	14·4 (9·1–19·2)	35·2 (21·6–44·6)
Southern Sub-Saharan Africa	Malaria	21·2 (-11·8–61·4)	14·9 (-7·9–40·7)	--	9·0 (-3·8–36·5)
Southern Sub-Saharan Africa	Measles	47·6 (25·2–56·3)	18·0 (9·0–23·3)	10·4 (4·0–19·0)	29·3 (14·3–37·2)
Botswana	All causes	26·8 (19·7–33·1)	16·7 (8·6–24·6)	16·2 (10·2–20·9)	9·6 (6·4–13·7)
Botswana	Diarrheal diseases	74·4 (46·3–92·3)	26·1 (15·5–36·5)	51·6 (-2·0–89·1)	27·6 (19·4–34·5)
Botswana	Lower respiratory infections	74·5 (61·5–86·8)	45·1 (2·0–82·0)	28·3 (19·1–36·7)	38·5 (22·9–49·4)
Botswana	Malaria	22·9 (-13·5–66·9)	15·8 (-8·8–47·7)	--	9·8 (-4·9–46·7)
Botswana	Measles	56·2 (29·8–67·0)	20·4 (10·0–26·2)	20·5 (7·7–36·3)	32·0 (15·3–40·5)

Eswatini	All causes	19·3 (14·2–25·6)	12·1 (4·8–20·5)	6·9 (5·2–9·2)	8·4 (5·3–12·1)
Eswatini	Diarrheal diseases	58·1 (40·2–80·2)	19·5 (10·9–28·8)	30·8 (-0·82–73·0)	23·7 (16·7–30·5)
Eswatini	Lower respiratory infections	64·3 (48·4–81·3)	38·5 (1·5–75·4)	10·9 (6·6–15·0)	35·0 (21·1–45·2)
Eswatini	Malaria	17·7 (-9·0–55·5)	10·8 (-5·1–31·9)	--	8·8 (-3·8–37·5)
Eswatini	Measles	42·5 (21·6–50·9)	14·4 (6·7–19·1)	6·1 (2·2–11·4)	28·8 (13·8–36·7)
Lesotho	All causes	19·3 (13·8–24·6)	11·6 (4·6–17·7)	7·9 (4·6–11·9)	8·9 (5·8–12·1)
Lesotho	Diarrheal diseases	68·8 (47·9–86·5)	26·8 (16·1–37·3)	40·2 (-1·2–80·6)	28·2 (20·3–34·9)
Lesotho	Lower respiratory infections	67·0 (52·1–80·2)	42·7 (2·0–75·0)	16·4 (10·4–22·2)	37·0 (22·5–47·1)
Lesotho	Malaria	0 (0–0)	0 (0–0)	--	0 (0–0)
Lesotho	Measles	54·6 (30·0–63·6)	22·5 (11·3–29·0)	11·0 (4·1–20·3)	35·0 (17·2–43·3)
Namibia	All causes	21·0 (15·3–26·2)	14·2 (7·0–20·4)	12·7 (9·1–15·8)	6·0 (4·0–8·6)
Namibia	Diarrheal diseases	72·5 (43·3–91·7)	26·8 (16·1–37·7)	52·0 (-2·5–89·0)	19·6 (13·3–25·3)
Namibia	Lower respiratory infections	71·1 (54·9–85·4)	45·3 (2·1–80·3)	27·2 (18·3–34·9)	30·5 (19·9–39·3)
Namibia	Malaria	23·3 (-14·7–65·9)	18·9 (-11·8–54·9)	--	6·7 (-3·3–36·6)
Namibia	Measles	54·1 (29·1–64·8)	23·4 (12·0–30·0)	19·6 (7·9–33·9)	26·6 (12·6–34·0)
South Africa	All causes	20·2 (15·6–25·1)	11·8 (6·8–16·8)	9·4 (6·0–13·4)	8·3 (5·8–10·9)
South Africa	Diarrheal diseases	59·8 (40·7–79·6)	19·8 (11·3–28·1)	32·0 (-0·95–72·0)	26·0 (18·3–32·1)
South Africa	Lower respiratory infections	65·4 (50·8–80·3)	39·0 (1·6–75·4)	12·2 (7·4–16·6)	37·1 (21·9–47·2)
South Africa	Malaria	18·1 (-10·7–54·7)	14·2 (-7·7–41·4)	--	5·1 (-2·1–30·3)
South Africa	Measles	43·2 (22·6–51·4)	14·3 (7·1–18·7)	7·9 (2·9–14·7)	28·5 (13·9–36·3)
Zimbabwe	All causes	22·3 (16·0–27·5)	15·4 (9·9–19·9)	13·9 (9·0–18·5)	6·3 (4·4–8·5)
Zimbabwe	Diarrheal diseases	66·4 (44·0–86·4)	24·5 (14·2–34·4)	41·1 (-1·4–81·7)	23·2 (16·0–29·1)
Zimbabwe	Lower respiratory infections	61·8 (47·2–74·7)	39·1 (1·9–69·5)	15·7 (9·9–20·9)	31·8 (21·1–40·4)
Zimbabwe	Malaria	21·1 (-11·7–61·2)	14·8 (-7·9–40·4)	--	9·0 (-3·9–36·5)
Zimbabwe	Measles	50·7 (27·3–59·5)	20·9 (10·6–26·9)	12·0 (4·7–21·6)	29·8 (14·5–37·7)
Western Sub-Saharan Africa	All causes	24·7 (11·3–34·5)	16·5 (5·9–24·4)	11·9 (6·5–17·3)	10·2 (6·1–15·4)
Western Sub-Saharan Africa	Diarrheal diseases	78·7 (55·8–91·2)	38·9 (26·3–50·6)	55·3 (-3·4–87·9)	26·5 (18·8–32·6)
Western Sub-Saharan Africa	Lower respiratory infections	65·2 (53·2–73·6)	45·5 (3·1–68·7)	24·5 (16·2–32·2)	37·5 (31·3–42·8)
Western Sub-Saharan Africa	Malaria	26·8 (-18·8–69·2)	19·9 (-12·4–50·2)	--	12·1 (-6·3–44·3)

Western Sub-Saharan Africa	Measles	67·8 (41·3–77·2)	36·1 (19·7–44·6)	26·7 (11·8–43·7)	35·1 (17·2–42·9)
Benin	All causes	20·0 (7·1–32·8)	13·3 (3·1–22·6)	7·5 (4·4–11·8)	8·4 (4·6–15·5)
Benin	Diarrheal diseases	73·9 (52·0–89·8)	32·1 (20·4–44·3)	48·1 (-2·3–85·2)	25·1 (17·9–31·8)
Benin	Lower respiratory infections	68·5 (54·6–79·0)	45·5 (2·6–72·9)	20·1 (12·7–27·1)	38·8 (32·4–44·8)
Benin	Malaria	24·5 (-15·1–67·2)	17·7 (-10·2–46·3)	--	10·4 (-4·5–40·3)
Benin	Measles	59·8 (33·9–69·4)	28·0 (14·5–35·6)	18·2 (7·3–31·9)	32·8 (16·2–41·0)
Burkina Faso	All causes	18·5 (8·4–29·2)	12·6 (2·7–21·2)	7·4 (4·8–10·3)	6·9 (4·5–12·0)
Burkina Faso	Diarrheal diseases	72·9 (48·6–86·0)	35·3 (23·5–46·5)	51·1 (-2·9–83·0)	22·4 (15·4–28·2)
Burkina Faso	Lower respiratory infections	51·0 (40·4–61·1)	35·4 (2·3–55·7)	20·0 (13·2–26·7)	25·4 (17·2–33·2)
Burkina Faso	Malaria	24·9 (-15·7–68·0)	19·7 (-11·9–50·2)	--	8·9 (-3·8–37·4)
Burkina Faso	Measles	65·0 (37·6–74·0)	33·9 (18·1–42·0)	25·4 (11·1–43·2)	30·5 (14·2–38·2)
Cabo Verde	All causes	9·0 (5·6–12·9)	5·1 (2·7–7·7)	5·2 (2·9–8·3)	2·3 (1·5–3·3)
Cabo Verde	Diarrheal diseases	61·4 (32·6–84·4)	19·7 (11·5–28·5)	44·1 (-2·1–81·0)	12·8 (8·3–16·9)
Cabo Verde	Lower respiratory infections	52·8 (38·5–67·7)	33·4 (1·5–63·1)	17·6 (11·3–23·1)	19·4 (13·0–25·4)
Cabo Verde	Malaria	24·5 (-17·1–66·5)	24·5 (-17·1–66·4)	--	<0.1 (>-0.1–<0.1)
Cabo Verde	Measles	40·7 (21·2–49·4)	16·6 (8·4–21·2)	14·8 (6·1–26·0)	16·9 (8·1–22·3)
Cameroon	All causes	21·1 (10·3–31·7)	11·8 (4·3–19·2)	8·6 (3·8–15·3)	9·0 (5·5–13·2)
Cameroon	Diarrheal diseases	69·7 (46·1–88·0)	27·1 (16·6–37·9)	45·2 (-1·9–83·4)	23·1 (16·3–29·2)
Cameroon	Lower respiratory infections	60·6 (46·9–72·3)	38·4 (1·8–65·7)	16·5 (10·2–22·6)	33·7 (27·7–39·5)
Cameroon	Malaria	21·6 (-12·1–62·9)	14·7 (-7·6–40·1)	--	10·1 (-4·1–39·6)
Cameroon	Measles	55·6 (29·9–65·7)	23·9 (11·7–30·8)	16·3 (6·2–29·0)	31·1 (15·1–39·1)
Chad	All causes	39·4 (27·2–47·9)	22·7 (16·9–26·9)	24·9 (7·3–36·8)	13·8 (11·0–16·9)
Chad	Diarrheal diseases	80·0 (55·3–91·7)	39·3 (26·4–51·5)	58·3 (-3·8–89·4)	24·4 (16·9–30·2)
Chad	Lower respiratory infections	62·9 (51·0–71·0)	43·7 (2·8–66·1)	26·9 (18·1–34·7)	33·0 (27·3–38·4)
Chad	Malaria	27·5 (-18·1–73·1)	21·1 (-13·0–53·8)	--	11·2 (-5·0–45·3)
Chad	Measles	71·2 (42·9–81·9)	37·5 (20·0–46·5)	32·8 (15·0–53·1)	33·5 (15·9–41·8)
Côte d'Ivoire	All causes	16·3 (6·4–25·9)	10·4 (2·2–17·9)	6·6 (3·8–9·9)	6·0 (3·4–9·3)
Côte d'Ivoire	Diarrheal diseases	71·9 (45·1–89·6)	28·7 (17·6–39·7)	50·4 (-2·5–86·2)	19·8 (13·3–25·4)
Côte d'Ivoire	Lower respiratory infections	59·0 (45·7–70·6)	38·5 (2·0–65·6)	20·5 (13·4–26·6)	27·8 (21·1–33·9)

Côte d'Ivoire	Malaria	20·3 (-11·2–59·2)	15·3 (-8·3–41·4)	--	7·4 (-3·0–31·6)
Côte d'Ivoire	Measles	54·6 (29·6–65·3)	24·5 (12·5–31·4)	19·5 (8·0–33·4)	26·2 (12·5–33·5)
Gambia	All causes	13·7 (9·4–17·6)	9·0 (3·3–13·2)	6·0 (4·7–7·5)	5·1 (4·0–6·5)
Gambia	Diarrheal diseases	63·9 (39·8–80·6)	26·2 (16·4–36·6)	44·0 (-2·2–77·1)	17·0 (11·6–22·1)
Gambia	Lower respiratory infections	62·4 (47·4–74·8)	41·8 (2·3–69·7)	20·2 (12·5–27·6)	29·7 (24·6–35·2)
Gambia	Malaria	19·4 (-11·4–53·8)	15·9 (-9·4–41·8)	--	5·3 (-2·1–22·5)
Gambia	Measles	52·9 (28·9–62·4)	25·3 (13·1–32·0)	18·5 (7·9–31·9)	23·7 (11·1–30·6)
Ghana	All causes	11·2 (4·3–19·2)	8·0 (2·1–14·4)	4·9 (3·1–7·2)	3·2 (1·6–6·6)
Ghana	Diarrheal diseases	66·6 (40·2–84·9)	25·8 (15·7–35·6)	47·5 (-2·4–81·4)	16·0 (10·5–21·0)
Ghana	Lower respiratory infections	53·2 (38·3–66·7)	34·6 (1·8–58·6)	18·9 (11·8–25·6)	23·4 (18·3–29·0)
Ghana	Malaria	18·5 (-10·1–55·0)	14·7 (-7·8–40·5)	--	5·7 (-2·2–25·3)
Ghana	Measles	51·8 (27·8–62·0)	23·4 (12·0–29·8)	19·3 (7·9–32·7)	22·8 (10·8–29·3)
Guinea	All causes	22·3 (11·8–32·4)	14·2 (4·0–23·2)	8·7 (5·7–11·9)	10·0 (5·8–15·0)
Guinea	Diarrheal diseases	75·0 (50·6–89·4)	32·9 (21·1–44·4)	51·8 (-2·7–86·0)	24·4 (17·2–30·6)
Guinea	Lower respiratory infections	63·6 (51·1–73·4)	42·4 (2·4–68·2)	22·6 (14·7–29·9)	34·0 (27·5–40·1)
Guinea	Malaria	24·5 (-14·7–67·7)	17·4 (-10·0–45·6)	--	11·0 (-5·0–43·2)
Guinea	Measles	62·7 (35·6–73·3)	29·5 (15·3–37·6)	23·1 (9·7–39·5)	32·5 (15·5–40·8)
Guinea-Bissau	All causes	17·1 (11·5–22·3)	10·4 (5·1–14·7)	7·2 (4·5–10·9)	7·7 (5·5–10·1)
Guinea-Bissau	Diarrheal diseases	73·1 (52·4–88·6)	32·3 (20·6–43·9)	45·9 (-1·9–83·8)	26·4 (18·5–32·9)
Guinea-Bissau	Lower respiratory infections	67·7 (53·2–77·4)	45·4 (2·7–71·5)	18·1 (10·7–25·0)	40·2 (35·3–45·7)
Guinea-Bissau	Malaria	23·4 (-14·4–64·3)	17·5 (-10·4–45·2)	--	9·3 (-4·0–37·2)
Guinea-Bissau	Measles	59·0 (33·5–68·5)	28·1 (14·8–35·5)	16·8 (6·7–29·4)	32·5 (15·9–40·7)
Liberia	All causes	18·3 (9·0–27·5)	11·1 (3·6–18·7)	7·1 (4·1–10·3)	7·0 (4·6–10·3)
Liberia	Diarrheal diseases	69·1 (44·0–88·7)	26·9 (16·3–37·7)	45·8 (-1·9–85·3)	20·3 (13·8–26·3)
Liberia	Lower respiratory infections	63·1 (46·9–77·1)	41·2 (2·0–72·0)	18·3 (11·5–24·4)	30·1 (21·0–37·8)
Liberia	Malaria	19·8 (-10·9–57·0)	14·7 (-7·8–39·2)	--	7·5 (-2·9–30·3)
Liberia	Measles	53·0 (28·4–62·6)	23·8 (12·0–30·4)	15·4 (6·2–27·3)	27·6 (13·2–35·2)
Mali	All causes	19·8 (10·7–28·9)	12·5 (5·0–18·9)	9·1 (5·7–13·1)	7·1 (4·7–12·1)
Mali	Diarrheal diseases	76·0 (51·3–90·8)	34·4 (22·7–46·3)	53·9 (-3·2–87·7)	21·0 (14·3–26·8)

Mali	Lower respiratory infections	61·6 (49·0–72·1)	41·9 (2·5–66·2)	23·5 (15·5–31·2)	30·7 (26·0–35·6)
Mali	Malaria	23·8 (-14·2–65·3)	18·5 (-10·6–47·6)	--	8·7 (-3·7–35·7)
Mali	Measles	62·1 (35·5–72·7)	31·3 (16·5–39·5)	24·8 (10·9–41·5)	28·0 (13·1–35·6)
Mauritania	All causes	16·3 (9·5–22·1)	9·8 (5·0–14·0)	9·4 (4·2–14·8)	4·9 (3·6–6·6)
Mauritania	Diarrheal diseases	71·8 (44·4–88·3)	30·7 (19·6–42·0)	51·6 (-2·9–85·0)	17·5 (11·9–22·5)
Mauritania	Lower respiratory infections	53·2 (39·4–64·9)	36·1 (2·0–59·9)	20·3 (13·0–27·8)	23·7 (19·0–29·4)
Mauritania	Malaria	19·3 (-11·0–53·4)	15·5 (-8·7–40·4)	--	6·0 (-2·4–25·2)
Mauritania	Measles	58·1 (32·6–68·7)	28·5 (15·0–36·1)	23·1 (9·8–38·8)	25·2 (11·8–32·4)
Niger	All causes	34·0 (17·0–44·4)	24·7 (9·8–33·3)	16·4 (10·3–23·0)	15·4 (9·3–22·0)
Niger	Diarrheal diseases	85·7 (68·7–93·6)	50·6 (36·6–62·8)	59·1 (-4·5–90·1)	33·5 (24·4–40·1)
Niger	Lower respiratory infections	73·7 (63·4–79·9)	55·0 (4·5–75·5)	28·5 (18·8–37·0)	46·1 (39·6–51·3)
Niger	Malaria	36·0 (-31·5–84·2)	27·3 (-20·7–64·0)	--	17·9 (-9·6–61·3)
Niger	Measles	78·1 (51·5–86·6)	47·8 (27·7–57·3)	29·9 (13·4–47·7)	42·8 (21·4–51·4)
Nigeria	All causes	25·4 (9·7–36·3)	17·5 (5·0–26·8)	12·1 (6·3–17·4)	10·9 (5·7–17·6)
Nigeria	Diarrheal diseases	79·9 (57·7–92·0)	40·4 (28·1–52·1)	56·2 (-3·5–88·7)	28·5 (20·3–34·6)
Nigeria	Lower respiratory infections	66·2 (54·8–75·0)	46·6 (3·3–69·9)	25·5 (16·6–33·8)	39·7 (33·4–45·0)
Nigeria	Malaria	28·1 (-20·7–70·9)	20·7 (-13·1–51·0)	--	13·3 (-7·3–47·1)
Nigeria	Measles	74·5 (47·6–83·3)	41·6 (23·3–50·7)	30·8 (13·9–49·7)	41·6 (20·7–49·8)
Sao Tome and Principe	All causes	15·2 (11·5–19·5)	11·0 (6·8–15·5)	9·5 (6·9–12·6)	4·0 (2·7–5·6)
Sao Tome and Principe	Diarrheal diseases	61·3 (36·0–82·0)	21·1 (12·7–29·9)	41·1 (-1·6–78·4)	18·3 (12·4–23·4)
Sao Tome and Principe	Lower respiratory infections	52·0 (38·6–64·4)	31·8 (1·4–58·4)	14·7 (9·1–20·1)	25·6 (19·4–31·9)
Sao Tome and Principe	Malaria	15·8 (-7·8–48·8)	11·7 (-5·8–33·9)	--	5·5 (-2·1–24·2)
Sao Tome and Principe	Measles	44·8 (22·8–54·2)	17·4 (8·6–22·7)	15·0 (5·9–26·6)	22·1 (10·3–29·0)
Senegal	All causes	10·8 (6·8–14·1)	6·6 (3·3–9·4)	5·4 (3·4–7·2)	3·5 (2·4–4·8)
Senegal	Diarrheal diseases	62·8 (35·6–81·5)	24·6 (15·1–35·0)	44·0 (-2·3–77·5)	15·5 (10·0–20·7)
Senegal	Lower respiratory infections	58·8 (42·1–74·2)	38·7 (1·8–68·0)	20·0 (12·0–27·9)	26·2 (19·8–33·0)
Senegal	Malaria	17·2 (-9·2–50·4)	13·7 (-7·5–38·3)	--	5·0 (-2·0–21·8)
Senegal	Measles	51·1 (26·7–60·7)	23·3 (11·6–29·4)	19·9 (8·5–34·6)	21·3 (9·5–27·7)
Sierra Leone	All causes	24·7 (11·2–37·5)	14·8 (4·4–24·4)	11·4 (4·9–17·2)	9·2 (6·0–14·6)

Sierra Leone	Diarrheal diseases	75·4 (48·9–92·3)	31·5 (19·3–43·4)	51·6 (-2·3–89·2)	23·7 (16·3–30·1)
Sierra Leone	Lower respiratory infections	70·8 (55·8–83·8)	46·7 (2·3–79·4)	24·5 (16·4–32·0)	34·0 (22·8–43·0)
Sierra Leone	Malaria	22·8 (-12·9–65·3)	16·1 (-8·6–43·2)	--	9·9 (-4·3–39·7)
Sierra Leone	Measles	59·0 (32·3–69·5)	26·5 (13·2–34·0)	21·6 (8·8–38·0)	30·0 (14·1–38·1)
Togo	All causes	21·6 (10·9–31·8)	12·5 (5·0–18·6)	10·9 (3·3–19·3)	7·4 (4·7–9·9)
Togo	Diarrheal diseases	72·4 (48·5–88·6)	31·0 (19·3–42·4)	49·2 (-2·6–84·8)	20·7 (14·0–26·5)
Togo	Lower respiratory infections	59·7 (45·4–70·0)	40·0 (2·3–64·4)	18·9 (11·8–25·4)	30·7 (26·1–35·8)
Togo	Malaria	22·3 (-13·3–61·7)	17·5 (-10·1–45·9)	--	7·5 (-3·0–30·5)
Togo	Measles	56·3 (31·4–65·9)	26·8 (14·1–34·1)	19·1 (7·9–32·8)	27·0 (13·1–34·5)

Table S3. All-cause and cause-specific deaths associated with child growth failure at the global, super-regional, regional, and national levels among children under 5 years, 2023 Estimates combine burden associated with mild, moderate, and severe forms of CGF: stunting was defined as height-for-age z-score (HAZ) < –1; underweight as weight-for-age z-score (WAZ) < –1; wasting as weight-for-height z-score (WHZ) < –1, according to WHO Child Growth Standards.

Location	Cause name	Child growth failure	Child underweight	Child wasting	Child stunting
**Global**	**All causes**	**880 000 (517 000–1 170 000)**	**573 000 (236 000–824 000)**	**428 000 (256 000–583 000)**	**373 000 (272 000–477 000)**
**Global**	**Diarrheal diseases**	**243 000 (146 000–360 000)**	**118 000 (63 600–195 000)**	**169 000 (-11 900–327 000)**	**84 800 (49 900–132 000)**
**Global**	**Lower respiratory infections**	**362 000 (253 000–465 000)**	**249 000 (19 200–428 000)**	**141 000 (88 700–204 000)**	**196 000 (138 000–264 000)**
**Global**	**Malaria**	**107 000 (-71 300–341 000)**	**77 400 (-44 700–229 000)**	**--**	**50 800 (-26 800–224 000)**
**Global**	**Measles**	**78 600 (30 700–150 000)**	**40 400 (15 200–78 100)**	**29 300 (8 850–62 600)**	**42 000 (15 000–80 600)**
**Central Europe, Eastern Europe, and Central Asia**	**All causes**	**8 790 (6 390–11 400)**	**4 860 (512–9 720)**	**3 420 (2 670–4 320)**	**3 410 (2 230–4 560)**
**Central Europe, Eastern Europe, and Central Asia**	**Diarrheal diseases**	**774 (380–1 140)**	**239 (123–386)**	**568 (-21·4–1 080)**	**195 (117–284)**
**Central Europe, Eastern Europe, and Central Asia**	**Lower respiratory infections**	**7 920 (5 900–10 300)**	**4 540 (169–9 430)**	**2 770 (1 770–3 830)**	**3 200 (2 090–4 320)**
**Central Europe, Eastern Europe, and Central Asia**	**Malaria**	**0 (0–0)**	**0 (0–0)**	**--**	**0 (0–0)**
**Central Europe, Eastern Europe, and Central Asia**	**Measles**	**11·7 (5·63–16·4)**	**3·96 (1·84–5·75)**	**4·18 (1·55–8·42)**	**5·76 (2·63–8·34)**
Central Asia	All causes	7 920 (5 760–10 200)	4 440 (454–8 790)	3 110 (2 370–3 960)	3 030 (1 980–4 110)
Central Asia	Diarrheal diseases	650 (324–966)	213 (109–352)	478 (-19–915)	163 (95·8–245)
Central Asia	Lower respiratory infections	7 200 (5 360–9 360)	4 170 (156–8 540)	2 570 (1 630–3 540)	2 860 (1 870–3 900)
Central Asia	Malaria	0 (0–0)	0 (0–0)	--	0 (0–0)
Central Asia	Measles	11·4 (5·47–16·0)	3·86 (1·81–5·6)	4·09 (1·53–8·17)	5·58 (2·56–8·11)
Armenia	All causes	56·3 (42·0–75·3)	28·6 (1·77–63·0)	17·1 (11·7–23·8)	27·4 (17·9–37·8)
Armenia	Diarrheal diseases	2·66 (1·25–3·98)	0·622 (0·317–1)	1·83 (-0·0478–3·81)	0·842 (0·53–1·23)
Armenia	Lower respiratory infections	53·6 (40·4–71·6)	28·0 (1–62·4)	15·2 (9·03–22·6)	26·6 (17·0–36·6)
Armenia	Malaria	0 (0–0)	0 (0–0)	--	0 (0–0)
Armenia	Measles	<0.001 (<0.001–<0.001)	<0.001 (<0.001–<0.001)	<0.001 (<0.001–<0.001)	<0.001 (<0.001–<0.001)
Azerbaijan	All causes	940 (656–1 240)	539 (92·6–1 010)	390 (275–503)	364 (212–533)
Azerbaijan	Diarrheal diseases	139 (65·0–253)	46·6 (21·1–85·5)	99·4 (-3·93–242)	36·9 (18·4–62·9)
Azerbaijan	Lower respiratory infections	783 (538–1 060)	480 (18·6–965)	277 (164–396)	323 (178–475)
Azerbaijan	Malaria	0 (0–0)	0 (0–0)	--	0 (0–0)
Azerbaijan	Measles	9·02 (4·29–12·9)	3·13 (1·42–4·56)	3·18 (1·17–6·65)	4·43 (2·06–6·58)

Georgia	All causes	21·8 (14·8–33·6)	10·9 (0·648–27·3)	5·03 (3·27–7·3)	10·0 (7·39–13·5)
Georgia	Diarrheal diseases	0·68 (0·293–1·24)	0·145 (0·0687–0·236)	0·466 (-0·0108–1·2)	0·168 (0·0965–0·242)
Georgia	Lower respiratory infections	20·9 (14·3–32·6)	10·5 (0·292–26·9)	4·36 (2·41–6·64)	9·85 (7·25–13·3)
Georgia	Malaria	0 (0–0)	0 (0–0)	--	0 (0–0)
Georgia	Measles	<0.001 (<0.001–<0.001)	<0.001 (<0.001–<0.001)	<0.001 (<0.001–<0.001)	<0.001 (<0.001–<0.001)
Kazakhstan	All causes	454 (318–630)	253 (39·9–539)	174 (126–235)	169 (105–246)
Kazakhstan	Diarrheal diseases	23·2 (9·81–36·4)	5·42 (2·78–8·5)	16·9 (-0·513–34·8)	5·57 (3·31–7·9)
Kazakhstan	Lower respiratory infections	405 (286–566)	222 (7·93–507)	131 (76·6–189)	163 (99·9–238)
Kazakhstan	Malaria	0 (0–0)	0 (0–0)	--	0 (0–0)
Kazakhstan	Measles	0·306 (0·0545–0·677)	0·0837 (0·0136–0·208)	0·101 (0·0145–0·254)	0·159 (0·0285–0·364)
Kyrgyzstan	All causes	288 (197–407)	148 (18·2–338)	89·2 (69·6–113)	125 (86·4–177)
Kyrgyzstan	Diarrheal diseases	32·3 (14·8–51·6)	7·44 (3·79–11·7)	21·8 (-0·485–49·5)	9·28 (5·77–13·3)
Kyrgyzstan	Lower respiratory infections	249 (174–362)	135 (4·41–324)	61·5 (36·3–90·7)	116 (79·1–165)
Kyrgyzstan	Malaria	0 (0–0)	0 (0–0)	--	0 (0–0)
Kyrgyzstan	Measles	0·437 (0·133–0·938)	0·123 (0·037–0·261)	0·114 (0·0275–0·256)	0·258 (0·0763–0·576)
Mongolia	All causes	83·6 (56·0–127)	42·4 (2·95–106)	16·2 (10·2–23·2)	41·6 (28·0–60·0)
Mongolia	Diarrheal diseases	2·71 (1·13–5·43)	0·572 (0·227–1·25)	1·69 (-0·032–5·13)	0·83 (0·389–1·58)
Mongolia	Lower respiratory infections	79·8 (52·5–122)	40·8 (1·36–105)	13·5 (6·87–21·4)	40·8 (27·6–59·1)
Mongolia	Malaria	0 (0–0)	0 (0–0)	--	0 (0–0)
Mongolia	Measles	0 (0–0)	0 (0–0)	0 (0–0)	0 (0–0)
Tajikistan	All causes	1 290 (886–1 650)	719 (165–1 170)	634 (407–804)	500 (343–673)
Tajikistan	Diarrheal diseases	348 (165–571)	125 (61·4–212)	259 (-11·7–526)	88·0 (49·5–137)
Tajikistan	Lower respiratory infections	941 (680–1 230)	592 (34·1–1 090)	373 (216–561)	412 (277–566)
Tajikistan	Malaria	0 (0–0)	0 (0–0)	--	0 (0–0)
Tajikistan	Measles	0·529 (0·272–0·777)	0·21 (0·102–0·332)	0·209 (0·0827–0·403)	0·252 (0·114–0·379)
Turkmenistan	All causes	589 (401–781)	338 (17·9–661)	223 (148–300)	216 (155–294)
Turkmenistan	Diarrheal diseases	24·8 (11·2–36·9)	6·96 (3·4–11·6)	18·7 (-0·467–36·2)	4·92 (2·82–7·46)
Turkmenistan	Lower respiratory infections	562 (387–748)	329 (10·7–652)	201 (119–285)	212 (152–286)
Turkmenistan	Malaria	0 (0–0)	0 (0–0)	--	0 (0–0)

Turkmenistan	Measles	0 (0–0)	0 (0–0)	0 (0–0)	0 (0–0)
Uzbekistan	All causes	4 200 (2 970–5 560)	2 360 (113–5 040)	1 560 (1 020–2 160)	1 580 (992–2 180)
Uzbekistan	Diarrheal diseases	76·9 (33·6–116)	19·8 (10·5–31·8)	58·3 (-1·86–113)	16·7 (10·1–24·0)
Uzbekistan	Lower respiratory infections	4 110 (2 930–5 430)	2 330 (77·7–5 010)	1 490 (909–2 100)	1 560 (977–2 160)
Uzbekistan	Malaria	0 (0–0)	0 (0–0)	--	0 (0–0)
Uzbekistan	Measles	1·12 (0·365–2·76)	0·314 (0·0987–0·79)	0·484 (0·133–1·39)	0·476 (0·153–1·12)
Central Europe	All causes	368 (250–522)	178 (32·4–371)	149 (99·8–189)	138 (90·0–189)
Central Europe	Diarrheal diseases	79·2 (31·7–127)	16·1 (8·38–24·4)	59·2 (-1·6–123)	18·0 (11·1–24·6)
Central Europe	Lower respiratory infections	281 (210–389)	154 (5·55–348)	81·8 (49·8–112)	119 (74·4–166)
Central Europe	Malaria	0 (0–0)	0 (0–0)	--	0 (0–0)
Central Europe	Measles	0·322 (0·12–0·689)	0·0997 (0·0343–0·222)	0·0882 (0·0259–0·241)	0·18 (0·0661–0·391)
Albania	All causes	13·9 (8·41–20·9)	7·15 (1·28–16·3)	6·28 (3·98–8·93)	6·11 (3·11–9·72)
Albania	Diarrheal diseases	1·23 (0·415–2·49)	0·272 (0·105–0·564)	0·923 (-0·0253–2·37)	0·388 (0·174–0·728)
Albania	Lower respiratory infections	11·8 (6·84–18·4)	6·01 (0·197–15·1)	4·48 (2·04–7·35)	5·72 (2·65–9·33)
Albania	Malaria	0 (0–0)	0 (0–0)	--	0 (0–0)
Albania	Measles	<0.001 (0–0·00119)	<0.001 (0–<0.001)	<0.001 (0–<0.001)	<0.001 (0–<0.001)
Bosnia and Herzegovina	All causes	4·31 (2·61–6·27)	1·68 (0·343–3·87)	1·91 (0·989–2·73)	1·82 (1·16–2·56)
Bosnia and Herzegovina	Diarrheal diseases	1·29 (0·452–2·16)	0·25 (0·107–0·444)	0·949 (-0·0282–2·03)	0·369 (0·192–0·608)
Bosnia and Herzegovina	Lower respiratory infections	2·96 (2·07–4·39)	1·37 (0·044–3·69)	0·902 (0·463–1·32)	1·45 (0·927–2·06)
Bosnia and Herzegovina	Malaria	0 (0–0)	0 (0–0)	--	0 (0–0)
Bosnia and Herzegovina	Measles	<0.001 (0–0·00104)	<0.001 (0–<0.001)	<0.001 (0–<0.001)	<0.001 (0–<0.001)
Bulgaria	All causes	30·4 (19·1–47·6)	14·9 (1·71–37·1)	11·9 (8·64–14·6)	9·48 (6·47–13·5)
Bulgaria	Diarrheal diseases	4·61 (1·55–7·97)	0·896 (0·433–1·46)	3·55 (-0·0962–7·77)	0·794 (0·452–1·21)
Bulgaria	Lower respiratory infections	25·5 (17·0–40·6)	13·7 (0·394–36·0)	8·02 (4·55–11·4)	8·69 (5·88–12·3)
Bulgaria	Malaria	0 (0–0)	0 (0–0)	--	0 (0–0)
Bulgaria	Measles	0 (0–0)	0 (0–0)	0 (0–0)	0 (0–0)
Croatia	All causes	7·44 (4·75–10·8)	3·11 (0·657–6·79)	3·34 (1·96–4·5)	2·68 (1·88–3·63)
Croatia	Diarrheal diseases	2·04 (0·768–3·35)	0·379 (0·179–0·611)	1·56 (-0·0456–3·25)	0·455 (0·255–0·661)
Croatia	Lower respiratory infections	5·28 (3·64–7·57)	2·62 (0·0804–6·47)	1·67 (0·939–2·46)	2·22 (1·58–3·02)

Croatia	Malaria	0 (0–0)	0 (0–0)	--	0 (0–0)
Croatia	Measles	0 (0–0)	0 (0–0)	0 (0–0)	0 (0–0)
Czechia	All causes	26·9 (15·0–40·4)	11·2 (4·14–21·7)	15·8 (6·78–23·6)	5·84 (4·05–8·24)
Czechia	Diarrheal diseases	11·8 (3·97–18·9)	2·53 (1·24–3·99)	9·42 (-0·281–18·4)	1·66 (0·878–2·46)
Czechia	Lower respiratory infections	14·1 (9·49–21·6)	7·68 (0·25–18·7)	5·32 (3·02–7·67)	4·18 (2·91–5·99)
Czechia	Malaria	0 (0–0)	0 (0–0)	--	0 (0–0)
Czechia	Measles	<0.001 (<0.001–<0.001)	<0.001 (<0.001–<0.001)	<0.001 (<0.001–<0.001)	<0.001 (<0.001–<0.001)
Hungary	All causes	21·5 (11·8–33·7)	6·41 (3·18–11·6)	12·8 (2·93–24·4)	6·54 (4·29–9·36)
Hungary	Diarrheal diseases	13·2 (4·86–22·9)	2·26 (1·14–3·66)	9·99 (-0·286–22·2)	3·11 (1·78–4·54)
Hungary	Lower respiratory infections	7·87 (5·49–11·8)	3·74 (0·109–9·89)	2·42 (1·33–3·67)	3·43 (2·25–4·84)
Hungary	Malaria	0 (0–0)	0 (0–0)	--	0 (0–0)
Hungary	Measles	0 (0–0)	0 (0–0)	0 (0–0)	0 (0–0)
Montenegro	All causes	1·63 (1·03–2·51)	0·819 (0·115–1·98)	0·534 (0·385–0·73)	0·686 (0·381–1·09)
Montenegro	Diarrheal diseases	0·163 (0·0632–0·321)	0·033 (0·0137–0·066)	0·113 (-0·00337–0·3)	0·0466 (0·0233–0·0823)
Montenegro	Lower respiratory infections	1·41 (0·887–2·2)	0·731 (0·0263–1·9)	0·366 (0·2–0·605)	0·639 (0·346–1·02)
Montenegro	Malaria	0 (0–0)	0 (0–0)	--	0 (0–0)
Montenegro	Measles	0 (0–0)	0 (0–0)	0 (0–0)	0 (0–0)
North Macedonia	All causes	5·04 (2·9–7·32)	1·54 (0·408–3·41)	2·87 (1·17–4·45)	1·65 (0·992–2·52)
North Macedonia	Diarrheal diseases	2·21 (0·583–3·79)	0·297 (0·131–0·522)	1·77 (-0·0433–3·61)	0·486 (0·264–0·761)
North Macedonia	Lower respiratory infections	2·75 (1·96–3·91)	1·17 (0·034–3·11)	1·03 (0·59–1·56)	1·16 (0·663–1·82)
North Macedonia	Malaria	0 (0–0)	0 (0–0)	--	0 (0–0)
North Macedonia	Measles	0·00269 (<0.001–0·00479)	<0.001 (<0.001–<0.001)	<0.001 (<0.001–0·00214)	0·00149 (<0.001–0·00285)
Poland	All causes	53·2 (32·9–78·5)	21·0 (5·04–47·3)	24·8 (12·5–37·3)	18·7 (12·8–26·2)
Poland	Diarrheal diseases	17·5 (6·58–30·3)	3·08 (1·56–4·93)	13·4 (-0·342–29·3)	3·89 (2·39–5·56)
Poland	Lower respiratory infections	34·8 (23·7–50·1)	17·1 (0·542–43·6)	10·6 (6·13–15·6)	14·8 (10·2–21·0)
Poland	Malaria	0 (0–0)	0 (0–0)	--	0 (0–0)
Poland	Measles	<0.001 (<0.001–<0.001)	<0.001 (<0.001–<0.001)	<0.001 (<0.001–<0.001)	<0.001 (<0.001–<0.001)
Romania	All causes	155 (110–216)	87·2 (11·1–178)	47·9 (36·0–61·0)	65·6 (38·2–93·6)
Romania	Diarrheal diseases	14·5 (7·61–22·9)	3·94 (2·04–6·22)	9·53 (-0·231–22·1)	4·22 (2·62–6·12)

Romania	Lower respiratory infections	137 (98·1–190)	79·8 (3·17–171)	34·9 (20·0–50·6)	61·2 (34·9–87·9)
Romania	Malaria	0 (0–0)	0 (0–0)	--	0 (0–0)
Romania	Measles	0·307 (0·112–0·668)	0·0957 (0·0328–0·215)	0·0836 (0·0237–0·232)	0·171 (0·0621–0·378)
Serbia	All causes	12·6 (8·09–18·4)	5·87 (1·29–12·2)	5·53 (3·16–7·9)	4·88 (3·33–6·95)
Serbia	Diarrheal diseases	3·32 (1·16–5·75)	0·761 (0·337–1·34)	2·45 (-0·081–5·52)	0·864 (0·479–1·48)
Serbia	Lower respiratory infections	8·91 (6·08–13·1)	4·78 (0·2–11·2)	2·76 (1·6–4·24)	4·02 (2·65–5·93)
Serbia	Malaria	0 (0–0)	0 (0–0)	--	0 (0–0)
Serbia	Measles	0·00693 (0·00261–0·0111)	0·00181 (<0.001–0·00303)	0·00195 (<0.001–0·00414)	0·00396 (0·00134–0·00693)
Slovakia	All causes	28·3 (18·9–39·8)	12·8 (1·86–29·4)	12·0 (8·49–15·2)	10·4 (6·63–14·8)
Slovakia	Diarrheal diseases	5·9 (1·97–10·2)	1·15 (0·569–1·98)	4·49 (-0·107–9·62)	1·35 (0·763–2·1)
Slovakia	Lower respiratory infections	22·1 (16·4–31·8)	11·3 (0·355–28·0)	7·18 (4·21–10·3)	9·05 (5·52–13·0)
Slovakia	Malaria	0 (0–0)	0 (0–0)	--	0 (0–0)
Slovakia	Measles	0 (0–0)	0 (0–0)	0 (0–0)	0 (0–0)
Slovenia	All causes	2·98 (1·98–4·54)	1·37 (0·119–3·54)	1·14 (0·823–1·5)	1·09 (0·743–1·53)
Slovenia	Diarrheal diseases	0·281 (0·0905–0·48)	0·0451 (0·0209–0·0741)	0·22 (-0·00572–0·464)	0·056 (0·0322–0·0829)
Slovenia	Lower respiratory infections	2·67 (1·86–4·13)	1·29 (0·0338–3·47)	0·89 (0·508–1·28)	1·04 (0·709–1·45)
Slovenia	Malaria	0 (0–0)	0 (0–0)	--	0 (0–0)
Slovenia	Measles	0 (0–0)	0 (0–0)	0 (0–0)	0 (0–0)
Eastern Europe	All causes	494 (365–675)	240 (25·3–542)	163 (123–213)	234 (152–321)
Eastern Europe	Diarrheal diseases	44·8 (21·0–68·1)	9·71 (4·88–15·4)	31·0 (-0·808–65·9)	14·3 (8·68–20·3)
Eastern Europe	Lower respiratory infections	442 (333–604)	223 (7·11–527)	125 (74·4–181)	220 (140–304)
Eastern Europe	Malaria	0 (0–0)	0 (0–0)	--	0 (0–0)
Eastern Europe	Measles	0·0129 (0·00535–0·0254)	0·00415 (0·00165–0·00886)	0·00341 (0·0012–0·00768)	0·0067 (0·00273–0·0138)
Belarus	All causes	9·54 (5·91–15·1)	5·91 (0·736–13·2)	2·94 (2·1–4)	2·87 (1·81–4·23)
Belarus	Diarrheal diseases	0·697 (0·32–1·2)	0·2 (0·102–0·319)	0·48 (-0·011–1·14)	0·133 (0·0779–0·197)
Belarus	Lower respiratory infections	8·52 (5·22–13·6)	5·38 (0·185–12·6)	2·14 (1·22–3·23)	2·74 (1·69–4·04)
Belarus	Malaria	0 (0–0)	0 (0–0)	--	0 (0–0)
Belarus	Measles	0·00558 (0·00164–0·0135)	0·00243 (<0.001–0·00617)	0·00145 (<0.001–0·00392)	0·00211 (<0.001–0·00503)
Estonia	All causes	1·51 (0·943–2·26)	0·77 (0·0687–1·89)	0·469 (0·315–0·629)	0·587 (0·41–0·815)

Estonia	Diarrheal diseases	0·099 (0·036–0·174)	0·02 (0·00977–0·0331)	0·0722 (-0·00172–0·169)	0·0215 (0·012–0·0321)
Estonia	Lower respiratory infections	1·39 (0·88–2·13)	0·727 (0·0223–1·84)	0·374 (0·205–0·561)	0·565 (0·396–0·788)
Estonia	Malaria	0 (0–0)	0 (0–0)	--	0 (0–0)
Estonia	Measles	<0.001 (<0.001–<0.001)	<0.001 (<0.001–<0.001)	<0.001 (<0.001–<0.001)	<0.001 (<0.001–<0.001)
Latvia	All causes	2·61 (1·75–3·97)	1·39 (0·0933–3·32)	0·751 (0·48–1·07)	1·04 (0·71–1·47)
Latvia	Diarrheal diseases	0·111 (0·0465–0·192)	0·0241 (0·0121–0·0392)	0·0798 (-0·00206–0·182)	0·0256 (0·015–0·0385)
Latvia	Lower respiratory infections	2·47 (1·68–3·78)	1·34 (0·0407–3·26)	0·646 (0·364–0·99)	1·02 (0·695–1·43)
Latvia	Malaria	0 (0–0)	0 (0–0)	--	0 (0–0)
Latvia	Measles	0 (0–0)	0 (0–0)	0 (0–0)	0 (0–0)
Lithuania	All causes	2·65 (1·56–4·07)	1·28 (0·154–3·12)	0·914 (0·618–1·18)	0·989 (0·655–1·43)
Lithuania	Diarrheal diseases	0·401 (0·152–0·697)	0·0818 (0·0408–0·137)	0·291 (-0·00671–0·68)	0·0886 (0·0518–0·134)
Lithuania	Lower respiratory infections	2·21 (1·38–3·38)	1·17 (0·0322–3)	0·592 (0·331–0·892)	0·901 (0·606–1·29)
Lithuania	Malaria	0 (0–0)	0 (0–0)	--	0 (0–0)
Lithuania	Measles	<0.001 (<0.001–<0.001)	<0.001 (<0.001–<0.001)	<0.001 (<0.001–<0.001)	<0.001 (<0.001–<0.001)
Republic of Moldova	All causes	17·3 (11·6–25·7)	9·14 (0·848–20·7)	5·66 (4·25–7·55)	6·32 (4·26–8·93)
Republic of Moldova	Diarrheal diseases	1·44 (0·562–2·42)	0·314 (0·153–0·493)	1·05 (-0·0279–2·34)	0·307 (0·183–0·437)
Republic of Moldova	Lower respiratory infections	15·6 (10·9–23·5)	8·64 (0·285–20·3)	4·42 (2·6–6·54)	6·01 (4–8·51)
Republic of Moldova	Malaria	0 (0–0)	0 (0–0)	--	0 (0–0)
Republic of Moldova	Measles	<0.001 (<0.001–0·0013)	<0.001 (<0.001–<0.001)	<0.001 (<0.001–<0.001)	<0.001 (<0.001–<0.001)
Russian Federation	All causes	404 (294–550)	192 (19·9–436)	138 (103–182)	194 (125–271)
Russian Federation	Diarrheal diseases	37·9 (17·3–58·3)	7·85 (3·91–12·5)	26·4 (-0·689–55·6)	12·3 (7·43–17·6)
Russian Federation	Lower respiratory infections	361 (270–490)	178 (5·56–425)	106 (62·3–155)	182 (115–256)
Russian Federation	Malaria	0 (0–0)	0 (0–0)	--	0 (0–0)
Russian Federation	Measles	0·00225 (<0.001–0·00376)	<0.001 (<0.001–<0.001)	<0.001 (<0.001–0·00184)	0·00131 (<0.001–0·00231)
Ukraine	All causes	55·7 (40·7–78·9)	29·4 (3·33–63·8)	14·5 (10·7–19·3)	28·2 (19·1–39·0)
Ukraine	Diarrheal diseases	4·21 (2·29–6·65)	1·22 (0·661–1·88)	2·6 (-0·0699–6·14)	1·48 (0·939–2·07)
Ukraine	Lower respiratory infections	50·6 (37·6–71·4)	27·4 (0·979–62·1)	11·1 (6·18–16·4)	26·7 (18·1–36·9)
Ukraine	Malaria	0 (0–0)	0 (0–0)	--	0 (0–0)
Ukraine	Measles	0·00453 (0·00158–0·0133)	0·00108 (<0.001–0·00324)	0·00102 (<0.001–0·00352)	0·00303 (0·00103–0·00922)

**High-income**	**All causes**	**623 (422–964)**	**322 (111–697)**	**269 (201–352)**	**193 (138–264)**
**High-income**	**Diarrheal diseases**	**107 (39·1–210)**	**19·6 (9·8–30·3)**	**78·2 (-1·7–202)**	**20·8 (12·5–29·5)**
**High-income**	**Lower respiratory infections**	**436 (299–718)**	**222 (5·93–612)**	**110 (63·1–162)**	**172 (124–238)**
**High-income**	**Malaria**	**<0.001 (>-0.001–<0.001)**	**<0.001 (>-0.001–<0.001)**	**--**	**<0.001 (>-0.001–<0.001)**
**High-income**	**Measles**	**0·0505 (0·0185–0·0775)**	**0·0142 (0·00544–0·0228)**	**0·0198 (0·00615–0·0404)**	**0·0197 (0·00742–0·0321)**
Australasia	All causes	7·63 (4·92–12·8)	2·66 (0·715–7·32)	3·74 (2·38–5·84)	2·33 (1·58–3·37)
Australasia	Diarrheal diseases	1·82 (0·406–4·21)	0·147 (0·0702–0·238)	1·5 (-0·0318–4·11)	0·284 (0·168–0·411)
Australasia	Lower respiratory infections	5·31 (3·78–8·63)	2·02 (0·0413–6·79)	1·75 (0·911–2·66)	2·05 (1·37–3·01)
Australasia	Malaria	0 (0–0)	0 (0–0)	--	0 (0–0)
Australasia	Measles	0·0035 (<0.001–0·00754)	<0.001 (<0.001–<0.001)	0·00165 (<0.001–0·00464)	0·00152 (<0.001–0·00338)
Australia	All causes	5·12 (3·36–8·53)	1·67 (0·587–4·57)	2·78 (1·8–4·33)	1·49 (1·04–2·16)
Australia	Diarrheal diseases	1·25 (0·232–3·01)	0·0745 (0·0343–0·127)	1·06 (-0·0228–2·94)	0·176 (0·0999–0·264)
Australia	Lower respiratory infections	3·37 (2·39–5·5)	1·1 (0·0203–4·07)	1·23 (0·615–1·86)	1·31 (0·912–1·91)
Australia	Malaria	0 (0–0)	0 (0–0)	--	0 (0–0)
Australia	Measles	0·00325 (<0.001–0·00724)	<0.001 (<0.001–<0.001)	0·00155 (<0.001–0·00452)	0·0014 (<0.001–0·00313)
New Zealand	All causes	2·51 (1·57–4·29)	0·993 (0·108–2·92)	0·962 (0·577–1·55)	0·843 (0·525–1·23)
New Zealand	Diarrheal diseases	0·573 (0·177–1·22)	0·0721 (0·0349–0·117)	0·439 (-0·00899–1·18)	0·108 (0·0636–0·156)
New Zealand	Lower respiratory infections	1·94 (1·33–3·28)	0·919 (0·021–2·87)	0·521 (0·286–0·789)	0·734 (0·424–1·1)
New Zealand	Malaria	0 (0–0)	0 (0–0)	--	0 (0–0)
New Zealand	Measles	<0.001 (<0.001–<0.001)	<0.001 (<0.001–<0.001)	<0.001 (<0.001–<0.001)	<0.001 (<0.001–<0.001)
High-income Asia Pacific	All causes	46·1 (28·3–72·1)	23·6 (5·09–53·2)	18·5 (12·3–25·3)	13·6 (9·75–18·9)
High-income Asia Pacific	Diarrheal diseases	9·81 (3·58–17·7)	2·06 (1·04–3·24)	7·31 (-0·187–17·1)	1·68 (0·973–2·43)
High-income Asia Pacific	Lower respiratory infections	34·1 (22·0–56·5)	19·4 (0·563–50·1)	9·06 (4·97–13·5)	11·9 (8·5–16·6)
High-income Asia Pacific	Malaria	<0.001 (>-0.001–<0.001)	<0.001 (>-0.001–<0.001)	--	<0.001 (>-0.001–<0.001)
High-income Asia Pacific	Measles	0·0117 (0·00433–0·0195)	0·00393 (0·00146–0·00681)	0·00444 (0·00139–0·00974)	0·00433 (0·00161–0·00786)
Brunei Darussalam	All causes	1·05 (0·691–1·51)	0·508 (0·0592–1·13)	0·293 (0·202–0·406)	0·577 (0·362–0·85)
Brunei Darussalam	Diarrheal diseases	0·128 (0·0594–0·23)	0·0302 (0·0133–0·0584)	0·0793 (-0·00248–0·197)	0·0531 (0·0271–0·0917)
Brunei Darussalam	Lower respiratory infections	0·916 (0·608–1·34)	0·468 (0·0187–1·1)	0·204 (0·103–0·337)	0·524 (0·317–0·797)
Brunei Darussalam	Malaria	0 (0–0)	0 (0–0)	--	0 (0–0)

Brunei Darussalam	Measles	0<br>(0–0)	0<br>(0–0)	0<br>(0–0)	0<br>(0–0)
Japan	All causes	34·3<br>(20·8–52·9)	17·7<br>(3·93–39·9)	13·5<br>(8·97–18·6)	10·4<br>(7·3–14·5)
Japan	Diarrheal diseases	6·9<br>(2·62–12·8)	1·45<br>(0·738–2·3)	5·06<br>(-0·126–12·2)	1·29<br>(0·771–1·85)
Japan	Lower respiratory infections	25·5<br>(16·1–41·9)	14·4<br>(0·409–37·2)	6·58<br>(3·57–9·91)	9·11<br>(6·34–12·9)
Japan	Malaria	0<br>(0–0)	0<br>(0–0)	--	0<br>(0–0)
Japan	Measles	0·0109<br>(0·00396–0·0185)	0·00369<br>(0·00136–0·00641)	0·00412<br>(0·0013–0·00893)	0·00409<br>(0·00152–0·00746)
Republic of Korea	All causes	7·42<br>(4·04–12·7)	3·47<br>(0·915–8·03)	3·54<br>(1·85–5·98)	1·57<br>(1·11–2·17)
Republic of Korea	Diarrheal diseases	2·42<br>(0·748–4·91)	0·493<br>(0·225–0·861)	1·89<br>(-0·0509–4·71)	0·273<br>(0·132–0·448)
Republic of Korea	Lower respiratory infections	4·71<br>(2·98–8·3)	2·7<br>(0·0823–7·3)	1·37<br>(0·717–2·12)	1·3<br>(0·923–1·8)
Republic of Korea	Malaria	<0.001<br>(>-0.001–<0.001)	<0.001<br>(>-0.001–<0.001)	--	<0.001<br>(>-0.001–<0.001)
Republic of Korea	Measles	<0.001<br>(<0.001–<0.001)	<0.001<br>(<0.001–<0.001)	<0.001<br>(<0.001–<0.001)	<0.001<br>(<0.001–<0.001)
Singapore	All causes	3·38<br>(1·89–5·58)	1·86<br>(0·17–4·63)	1·19<br>(0·764–1·62)	1·02<br>(0·59–1·61)
Singapore	Diarrheal diseases	0·363<br>(0·139–0·651)	0·0868<br>(0·0435–0·147)	0·271<br>(-0·00746–0·627)	0·0626<br>(0·0344–0·0952)
Singapore	Lower respiratory infections	3<br>(1·75–4·97)	1·76<br>(0·0529–4·55)	0·909<br>(0·46–1·42)	0·957<br>(0·535–1·52)
Singapore	Malaria	0<br>(0–0)	0<br>(0–0)	--	0<br>(0–0)
Singapore	Measles	<0.001<br>(<0.001–0·00109)	<0.001<br>(<0.001–<0.001)	<0.001<br>(<0.001–<0.001)	<0.001<br>(<0.001–<0.001)
High-income North America	All causes	280<br>(191–434)	144<br>(45·0–322)	127<br>(101–152)	81·2<br>(60·3–112)
High-income North America	Diarrheal diseases	37·9<br>(11·4–73·4)	6·18<br>(3·06–9·57)	29·5<br>(-0·61–72·2)	6·24<br>(3·66–9·01)
High-income North America	Lower respiratory infections	207<br>(144–336)	102<br>(2·57–285)	61·9<br>(35·2–91·2)	74·9<br>(54·8–103)
High-income North America	Malaria	0<br>(0–0)	0<br>(0–0)	--	0<br>(0–0)
High-income North America	Measles	0·0158<br>(0·00506–0·0302)	0·0039<br>(0·00129–0·00814)	0·00722<br>(0·00195–0·0157)	0·00573<br>(0·00187–0·0117)
Canada	All causes	15·5<br>(7·35–27·6)	7·63<br>(2·33–17·8)	8·48<br>(4·63–13·6)	1·67<br>(1·22–2·41)
Canada	Diarrheal diseases	5·28<br>(1·29–10·8)	1·01<br>(0·498–1·6)	4·31<br>(-0·0811–10·5)	0·333<br>(0·187–0·507)
Canada	Lower respiratory infections	9·06<br>(4·92–17·8)	5·49<br>(0·129–16·2)	3·04<br>(1·69–4·55)	1·34<br>(0·988–1·93)
Canada	Malaria	0<br>(0–0)	0<br>(0–0)	--	0<br>(0–0)
Canada	Measles	0·00126<br>(<0.001–0·00259)	<0.001<br>(<0.001–<0.001)	<0.001<br>(<0.001–0·00197)	<0.001<br>(<0.001–<0.001)
Greenland	All causes	0·214<br>(0·124–0·353)	0·108<br>(0·0247–0·257)	0·0782<br>(0·0533–0·107)	0·0682<br>(0·0463–0·102)
Greenland	Diarrheal diseases	0·0345<br>(0·0118–0·0728)	0·00759<br>(0·00325–0·0151)	0·0246<br>(>-0.001–0·0688)	0·00691<br>(0·00326–0·0126)
Greenland	Lower respiratory infections	0·158<br>(0·0905–0·269)	0·0879<br>(0·00295–0·236)	0·0388<br>(0·0206–0·0634)	0·0568<br>(0·0364–0·0883)

Greenland	Malaria	0 (0–0)	0 (0–0)	--	0 (0–0)
Greenland	Measles	0·0115 (0·0031–0·0248)	0·00301 (<0.001–0·00696)	0·00486 (0·00112–0·012)	0·00446 (0·00135–0·0104)
United States of America	All causes	265 (183–403)	136 (42·7–304)	119 (94·2–140)	79·4 (58·9–110)
United States of America	Diarrheal diseases	32·6 (10·1–62·5)	5·16 (2·53–8·02)	25·2 (-0·528–61·4)	5·9 (3·46–8·52)
United States of America	Lower respiratory infections	197 (139–318)	96·2 (2·43–269)	58·9 (33·5–86·6)	73·5 (53·6–102)
United States of America	Malaria	0 (0–0)	0 (0–0)	--	0 (0–0)
United States of America	Measles	0·00301 (<0.001–0·00509)	<0.001 (<0.001–<0.001)	0·00156 (<0.001–0·00332)	0·00109 (<0.001–0·00183)
Southern Latin America	All causes	174 (127–254)	96·4 (40·1–189)	67·1 (54·0–83·7)	67·5 (48·9–92·0)
Southern Latin America	Diarrheal diseases	23·8 (12·3–41·7)	4·99 (2·43–7·73)	14·9 (-0·333–39·6)	7·23 (4·37–9·97)
Southern Latin America	Lower respiratory infections	119 (82·9–185)	59·9 (1·8–157)	20·7 (11·3–31·6)	60·2 (43·8–83·3)
Southern Latin America	Malaria	0 (0–0)	0 (0–0)	--	0 (0–0)
Southern Latin America	Measles	<0.001 (<0.001–<0.001)	<0.001 (<0.001–<0.001)	<0.001 (<0.001–<0.001)	<0.001 (<0.001–<0.001)
Argentina	All causes	139 (102–202)	73·0 (25·7–151)	49·0 (39·4–59·6)	59·9 (43·0–82·5)
Argentina	Diarrheal diseases	17·2 (9–29·5)	3·27 (1·55–5·37)	10·6 (-0·235–27·8)	5·9 (3·57–8·26)
Argentina	Lower respiratory infections	101 (72·1–154)	48·8 (1·44–129)	17·5 (9·5–27·1)	54·0 (38·5–75·0)
Argentina	Malaria	0 (0–0)	0 (0–0)	--	0 (0–0)
Argentina	Measles	0 (0–0)	0 (0–0)	0 (0–0)	0 (0–0)
Chile	All causes	28·9 (19·4–44·1)	19·9 (11·0–33·2)	15·6 (11·6–20·5)	5·29 (4·05–6·98)
Chile	Diarrheal diseases	4·98 (2·22–9·28)	1·28 (0·642–2·01)	3·4 (-0·0792–8·97)	0·765 (0·431–1·1)
Chile	Lower respiratory infections	14·4 (8·4–25·6)	9·08 (0·297–22·6)	2·63 (1·39–4·01)	4·53 (3·44–6·03)
Chile	Malaria	0 (0–0)	0 (0–0)	--	0 (0–0)
Chile	Measles	<0.001 (<0.001–<0.001)	<0.001 (<0.001–<0.001)	<0.001 (<0.001–<0.001)	<0.001 (<0.001–<0.001)
Uruguay	All causes	6·22 (4·44–8·81)	3·5 (1·66–6·23)	2·49 (1·61–3·66)	2·3 (1·53–3·21)
Uruguay	Diarrheal diseases	1·63 (0·894–2·59)	0·445 (0·218–0·693)	0·913 (-0·0188–2·36)	0·565 (0·352–0·771)
Uruguay	Lower respiratory infections	3·56 (2·45–5·56)	2·03 (0·0642–4·87)	0·555 (0·296–0·835)	1·74 (1·11–2·5)
Uruguay	Malaria	0 (0–0)	0 (0–0)	--	0 (0–0)
Uruguay	Measles	0 (0–0)	0 (0–0)	0 (0–0)	0 (0–0)
Western Europe	All causes	115 (67·3–200)	55·7 (19·1–130)	52·2 (29·7–90·3)	28·1 (19·3–39·9)
Western Europe	Diarrheal diseases	33·6 (11·2–70·8)	6·21 (3·09–9·73)	25·1 (-0·535–68·3)	5·33 (3·14–7·75)

Western Europe	Lower respiratory infections	70·9 (43·9–130)	39·2 (0·958–116)	16·9 (9·23–25·0)	22·7 (15·6–32·3)
Western Europe	Malaria	0 (0–0)	0 (0–0)	--	0 (0–0)
Western Europe	Measles	0·0194 (0·0067–0·0314)	0·00583 (0·00203–0·00999)	0·00637 (0·00183–0·0146)	0·00799 (0·003–0·0137)
Andorra	All causes	0·011 (0·00581–0·0214)	0·00579 (<0.001–0·0169)	0·00314 (0·00198–0·00476)	0·00339 (0·00206–0·00574)
Andorra	Diarrheal diseases	<0.001 (<0.001–0·00172)	<0.001 (<0.001–<0.001)	<0.001 (>-0.001–0·00166)	<0.001 (<0.001–<0.001)
Andorra	Lower respiratory infections	0·0101 (0·00519–0·0194)	0·00547 (<0.001–0·0165)	0·0024 (0·00111–0·00428)	0·00328 (0·00198–0·00561)
Andorra	Malaria	0 (0–0)	0 (0–0)	--	0 (0–0)
Andorra	Measles	0 (0–0)	0 (0–0)	0 (0–0)	0 (0–0)
Austria	All causes	0·832 (0·435–1·48)	0·345 (0·0739–0·846)	0·343 (0·146–0·661)	0·24 (0·17–0·332)
Austria	Diarrheal diseases	0·286 (0·0956–0·594)	0·0515 (0·0251–0·0837)	0·212 (-0·00449–0·576)	0·0483 (0·0268–0·073)
Austria	Lower respiratory infections	0·537 (0·313–0·949)	0·285 (0·00697–0·797)	0·122 (0·0622–0·196)	0·191 (0·138–0·274)
Austria	Malaria	0 (0–0)	0 (0–0)	--	0 (0–0)
Austria	Measles	<0.001 (<0.001–<0.001)	<0.001 (<0.001–<0.001)	<0.001 (<0.001–<0.001)	<0.001 (<0.001–<0.001)
Belgium	All causes	3·78 (1·99–6·72)	1·61 (0·49–4)	1·76 (0·723–3·44)	0·953 (0·63–1·41)
Belgium	Diarrheal diseases	1·47 (0·479–3·01)	0·263 (0·121–0·428)	1·09 (-0·0253–2·92)	0·242 (0·134–0·363)
Belgium	Lower respiratory infections	2·16 (1·32–3·97)	1·19 (0·0279–3·6)	0·518 (0·277–0·765)	0·711 (0·478–1·05)
Belgium	Malaria	0 (0–0)	0 (0–0)	--	0 (0–0)
Belgium	Measles	<0.001 (<0.001–<0.001)	<0.001 (<0.001–<0.001)	<0.001 (<0.001–<0.001)	<0.001 (<0.001–<0.001)
Cyprus	All causes	0·595 (0·255–1·26)	0·223 (0·0864–0·491)	0·317 (0·068–0·854)	0·141 (0·0804–0·23)
Cyprus	Diarrheal diseases	0·34 (0·111–0·827)	0·0711 (0·0282–0·147)	0·247 (-0·00537–0·798)	0·0606 (0·028–0·12)
Cyprus	Lower respiratory infections	0·245 (0·123–0·478)	0·141 (0·00466–0·429)	0·0593 (0·0284–0·107)	0·0802 (0·0415–0·143)
Cyprus	Malaria	0 (0–0)	0 (0–0)	--	0 (0–0)
Cyprus	Measles	0 (0–0)	0 (0–0)	0 (0–0)	0 (0–0)
Denmark	All causes	2·86 (1·39–5)	1·15 (0·387–2·7)	1·45 (0·49–2·88)	0·662 (0·397–1·02)
Denmark	Diarrheal diseases	1·35 (0·439–2·67)	0·256 (0·126–0·419)	1 (-0·021–2·56)	0·225 (0·129–0·346)
Denmark	Lower respiratory infections	1·42 (0·86–2·57)	0·812 (0·0203–2·37)	0·365 (0·19–0·579)	0·436 (0·228–0·715)
Denmark	Malaria	0 (0–0)	0 (0–0)	--	0 (0–0)
Denmark	Measles	0 (0–0)	0 (0–0)	0 (0–0)	0 (0–0)
Finland	All causes	0·838 (0·453–1·47)	0·401 (0·0667–1·03)	0·305 (0·174–0·475)	0·244 (0·167–0·36)

Finland	Diarrheal diseases	0·187 (0·0613–0·382)	0·0356 (0·0171–0·0596)	0·139 (-0·00304–0·369)	0·0314 (0·0179–0·048)
Finland	Lower respiratory infections	0·638 (0·361–1·16)	0·352 (0·00917–1)	0·153 (0·0807–0·241)	0·213 (0·145–0·311)
Finland	Malaria	0 (0–0)	0 (0–0)	--	0 (0–0)
Finland	Measles	0 (0–0)	0 (0–0)	0 (0–0)	0 (0–0)
France	All causes	25·9 (16·7–42·1)	15·1 (8·41–28·7)	14·7 (10·1–21·6)	5·16 (3·4–7·49)
France	Diarrheal diseases	6·02 (2·19–13·0)	1·07 (0·518–1·74)	4·49 (-0·102–12·6)	0·986 (0·557–1·44)
France	Lower respiratory infections	12·7 (7·93–23·1)	6·96 (0·171–20·4)	3·07 (1·68–4·7)	4·17 (2·77–6·2)
France	Malaria	0 (0–0)	0 (0–0)	--	0 (0–0)
France	Measles	<0.001 (<0.001–0·00112)	<0.001 (<0.001–<0.001)	<0.001 (<0.001–<0.001)	<0.001 (<0.001–<0.001)
Germany	All causes	15·8 (7·66–28·9)	6·24 (2·57–13·5)	8·62 (2·52–18·2)	3·18 (2·03–4·61)
Germany	Diarrheal diseases	8·35 (2·88–16·9)	1·67 (0·831–2·6)	6·3 (-0·129–16·5)	1·22 (0·719–1·75)
Germany	Lower respiratory infections	6·86 (4·04–12·3)	4·03 (0·1–11·3)	1·78 (0·987–2·62)	1·96 (1·26–2·94)
Germany	Malaria	0 (0–0)	0 (0–0)	--	0 (0–0)
Germany	Measles	0·00137 (<0.001–0·00291)	<0.001 (<0.001–<0.001)	<0.001 (<0.001–0·00118)	<0.001 (<0.001–0·00104)
Greece	All causes	2·69 (1·6–4·59)	1·43 (0·0809–3·99)	0·731 (0·501–1·01)	0·871 (0·489–1·33)
Greece	Diarrheal diseases	0·172 (0·062–0·332)	0·0324 (0·015–0·0527)	0·125 (-0·00278–0·32)	0·0317 (0·0171–0·0469)
Greece	Lower respiratory infections	2·51 (1·54–4·34)	1·39 (0·0336–3·95)	0·6 (0·321–0·918)	0·839 (0·469–1·29)
Greece	Malaria	0 (0–0)	0 (0–0)	--	0 (0–0)
Greece	Measles	0 (0–0)	0 (0–0)	0 (0–0)	0 (0–0)
Iceland	All causes	0·149 (0·0833–0·266)	0·0712 (0·013–0·189)	0·0579 (0·0339–0·0901)	0·0403 (0·025–0·0599)
Iceland	Diarrheal diseases	0·0338 (0·0108–0·0693)	0·00593 (0·00279–0·00965)	0·0254 (>-0.001–0·0669)	0·00545 (0·00284–0·00791)
Iceland	Lower respiratory infections	0·111 (0·0664–0·201)	0·0607 (0·00159–0·181)	0·0279 (0·0151–0·0414)	0·0349 (0·0214–0·0524)
Iceland	Malaria	0 (0–0)	0 (0–0)	--	0 (0–0)
Iceland	Measles	0 (0–0)	0 (0–0)	0 (0–0)	0 (0–0)
Ireland	All causes	1·7 (0·96–3·15)	0·856 (0·0866–2·46)	0·57 (0·404–0·766)	0·48 (0·291–0·729)
Ireland	Diarrheal diseases	0·234 (0·0715–0·488)	0·0392 (0·0177–0·0656)	0·177 (-0·00371–0·468)	0·0369 (0·0201–0·0576)
Ireland	Lower respiratory infections	1·45 (0·857–2·71)	0·794 (0·0174–2·41)	0·37 (0·196–0·575)	0·443 (0·261–0·689)
Ireland	Malaria	0 (0–0)	0 (0–0)	--	0 (0–0)
Ireland	Measles	0 (0–0)	0 (0–0)	0 (0–0)	0 (0–0)

Israel	All causes	7·25 (3·7–12·7)	2·81 (1·04–6·33)	3·66 (1·25–7·47)	1·74 (1·08–2·5)
Israel	Diarrheal diseases	3·45 (1·13–6·87)	0·609 (0·284–0·993)	2·55 (-0·056–6·63)	0·58 (0·315–0·866)
Israel	Lower respiratory infections	3·54 (2·12–6·46)	1·94 (0·0446–5·77)	0·844 (0·452–1·3)	1·16 (0·717–1·75)
Israel	Malaria	0 (0–0)	0 (0–0)	--	0 (0–0)
Israel	Measles	0·00123 (<0.001–0·00268)	<0.001 (<0.001–<0.001)	<0.001 (<0.001–0·00101)	<0.001 (<0.001–0·0013)
Italy	All causes	11·3 (6·52–19·5)	5·6 (1·19–15·0)	4·37 (2·73–6·49)	3·11 (1·94–4·61)
Italy	Diarrheal diseases	2·32 (0·758–4·72)	0·408 (0·196–0·649)	1·73 (-0·0342–4·55)	0·384 (0·22–0·562)
Italy	Lower respiratory infections	8·33 (4·96–15·9)	4·57 (0·111–14·2)	2·01 (1·09–3·07)	2·73 (1·69–4·13)
Italy	Malaria	0 (0–0)	0 (0–0)	--	0 (0–0)
Italy	Measles	<0.001 (<0.001–0·00108)	<0.001 (<0.001–<0.001)	<0.001 (<0.001–<0.001)	<0.001 (<0.001–<0.001)
Luxembourg	All causes	0·143 (0·0765–0·258)	0·0636 (0·0288–0·134)	0·0765 (0·0318–0·147)	0·0314 (0·02–0·0464)
Luxembourg	Diarrheal diseases	0·0625 (0·0223–0·126)	0·0119 (0·0058–0·0192)	0·0464 (-0·00109–0·122)	0·0102 (0·00591–0·0154)
Luxembourg	Lower respiratory infections	0·0672 (0·0397–0·122)	0·0381 (0·00105–0·11)	0·0165 (0·00878–0·0254)	0·0212 (0·013–0·0316)
Luxembourg	Malaria	0 (0–0)	0 (0–0)	--	0 (0–0)
Luxembourg	Measles	0 (0–0)	0 (0–0)	0 (0–0)	0 (0–0)
Malta	All causes	0·153 (0·0907–0·265)	0·0821 (0·007–0·223)	0·045 (0·0325–0·0594)	0·0464 (0·0283–0·0679)
Malta	Diarrheal diseases	0·0139 (0·00468–0·026)	0·00272 (0·00125–0·00421)	0·0102 (>-0.001–0·025)	0·00238 (0·00131–0·00341)
Malta	Lower respiratory infections	0·138 (0·0842–0·242)	0·0784 (0·00202–0·22)	0·0338 (0·019–0·0502)	0·0441 (0·0263–0·0649)
Malta	Malaria	0 (0–0)	0 (0–0)	--	0 (0–0)
Malta	Measles	0 (0–0)	0 (0–0)	0 (0–0)	0 (0–0)
Monaco	All causes	0·00946 (0·00477–0·0191)	0·00496 (<0.001–0·016)	0·0027 (0·00161–0·00426)	0·00286 (0·00139–0·00519)
Monaco	Diarrheal diseases	<0.001 (<0.001–0·00147)	<0.001 (<0.001–<0.001)	<0.001 (>-0.001–0·00142)	<0.001 (<0.001–<0.001)
Monaco	Lower respiratory infections	0·00882 (0·00432–0·0182)	0·00478 (<0.001–0·0158)	0·00219 (0·00101–0·0039)	0·00277 (0·00132–0·00505)
Monaco	Malaria	0 (0–0)	0 (0–0)	--	0 (0–0)
Monaco	Measles	0 (0–0)	0 (0–0)	0 (0–0)	0 (0–0)
Netherlands	All causes	4·3 (2·1–8·02)	2·59 (0·578–6·13)	1·25 (0·691–2·17)	1 (0·686–1·47)
Netherlands	Diarrheal diseases	1·01 (0·413–1·88)	0·307 (0·154–0·5)	0·62 (-0·0131–1·7)	0·162 (0·0909–0·248)
Netherlands	Lower respiratory infections	3·1 (1·5–6·18)	2·1 (0·0586–5·68)	0·446 (0·219–0·729)	0·837 (0·561–1·25)
Netherlands	Malaria	0 (0–0)	0 (0–0)	--	0 (0–0)

Netherlands	Measles	0·00256 (<0.001–0·00456)	<0.001 (<0.001–0·00162)	<0.001 (<0.001–0·00178)	<0.001 (<0.001–0·00187)
Norway	All causes	0·679 (0·381–1·22)	0·33 (0·0951–0·795)	0·302 (0·173–0·526)	0·168 (0·108–0·263)
Norway	Diarrheal diseases	0·172 (0·05–0·399)	0·0267 (0·0129–0·0454)	0·132 (-0·00325–0·383)	0·0261 (0·0148–0·0399)
Norway	Lower respiratory infections	0·446 (0·264–0·803)	0·243 (0·00577–0·719)	0·11 (0·0555–0·171)	0·142 (0·0884–0·223)
Norway	Malaria	0 (0–0)	0 (0–0)	--	0 (0–0)
Norway	Measles	<0.001 (<0.001–<0.001)	<0.001 (<0.001–<0.001)	<0.001 (<0.001–<0.001)	<0.001 (<0.001–<0.001)
Portugal	All causes	3·3 (2·01–5·79)	1·74 (0·334–4·37)	1·13 (0·772–1·59)	0·976 (0·676–1·43)
Portugal	Diarrheal diseases	0·519 (0·195–1·04)	0·104 (0·0516–0·174)	0·376 (-0·00967–1·01)	0·0917 (0·0524–0·139)
Portugal	Lower respiratory infections	2·64 (1·6–4·64)	1·49 (0·0387–4·11)	0·614 (0·321–0·922)	0·882 (0·596–1·31)
Portugal	Malaria	0 (0–0)	0 (0–0)	--	0 (0–0)
Portugal	Measles	0·00583 (0·00117–0·0141)	0·0019 (<0.001–0·00469)	0·00175 (<0.001–0·00529)	0·00245 (<0.001–0·00547)
San Marino	All causes	0·0048 (0·00233–0·00919)	0·00254 (<0.001–0·00756)	0·00141 (<0.001–0·00217)	0·00145 (<0.001–0·0027)
San Marino	Diarrheal diseases	<0.001 (<0.001–<0.001)	<0.001 (<0.001–<0.001)	<0.001 (>-0.001–<0.001)	<0.001 (<0.001–<0.001)
San Marino	Lower respiratory infections	0·00438 (0·00215–0·00869)	0·00241 (<0.001–0·00745)	0·00108 (<0.001–0·00198)	0·0014 (<0.001–0·00264)
San Marino	Malaria	0 (0–0)	0 (0–0)	--	0 (0–0)
San Marino	Measles	0 (0–0)	0 (0–0)	0 (0–0)	0 (0–0)
Spain	All causes	10·0 (5·65–18·1)	4·69 (1·07–12·0)	4·19 (2·16–7·39)	2·65 (1·66–3·85)
Spain	Diarrheal diseases	2·94 (0·941–6·08)	0·548 (0·265–0·897)	2·19 (-0·0447–5·93)	0·49 (0·276–0·732)
Spain	Lower respiratory infections	6·75 (4–12·7)	3·79 (0·0938–11·1)	1·65 (0·873–2·52)	2·16 (1·33–3·24)
Spain	Malaria	0 (0–0)	0 (0–0)	--	0 (0–0)
Spain	Measles	<0.001 (<0.001–<0.001)	<0.001 (<0.001–<0.001)	<0.001 (<0.001–<0.001)	<0.001 (<0.001–<0.001)
Sweden	All causes	2·62 (1·32–4·8)	1·08 (0·344–2·6)	1·28 (0·502–2·55)	0·627 (0·43–0·897)
Sweden	Diarrheal diseases	1·08 (0·33–2·28)	0·187 (0·0899–0·302)	0·817 (-0·0169–2·18)	0·17 (0·0972–0·256)
Sweden	Lower respiratory infections	1·42 (0·837–2·64)	0·776 (0·0169–2·34)	0·345 (0·183–0·522)	0·457 (0·319–0·667)
Sweden	Malaria	0 (0–0)	0 (0–0)	--	0 (0–0)
Sweden	Measles	<0.001 (<0.001–<0.001)	<0.001 (<0.001–<0.001)	<0.001 (<0.001–<0.001)	<0.001 (<0.001–<0.001)
Switzerland	All causes	1·69 (0·907–3·09)	0·744 (0·249–1·8)	0·831 (0·381–1·55)	0·399 (0·272–0·591)
Switzerland	Diarrheal diseases	0·632 (0·198–1·31)	0·109 (0·0535–0·177)	0·479 (-0·0103–1·27)	0·0991 (0·0557–0·15)
Switzerland	Lower respiratory infections	0·943 (0·567–1·78)	0·517 (0·0118–1·57)	0·234 (0·121–0·364)	0·3 (0·199–0·453)

Switzerland	Malaria	0<br>(0–0)	0<br>(0–0)	--	0<br>(0–0)
Switzerland	Measles	<0.001<br>(<0.001–<0.001)	<0.001<br>(<0.001–<0.001)	<0.001<br>(<0.001–<0.001)	<0.001<br>(<0.001–<0.001)
United Kingdom	All causes	18·1<br>(10·4–34·2)	8·47<br>(1·09–24·0)	6·26<br>(4·27–9·27)	5·31<br>(3·84–7·45)
United Kingdom	Diarrheal diseases	2·93<br>(0·835–6·76)	0·398<br>(0·182–0·67)	2·27<br>(-0·0472–6·56)	0·417<br>(0·226–0·643)
United Kingdom	Lower respiratory infections	14·8<br>(9·07–27·3)	7·63<br>(0·18–23·3)	3·54<br>(1·87–5·24)	4·89<br>(3·55–6·89)
United Kingdom	Malaria	0<br>(0–0)	0<br>(0–0)	--	0<br>(0–0)
United Kingdom	Measles	0·00725<br>(0·0027–0·0116)	0·00191<br>(<0.001–0·00312)	0·00259<br>(<0.001–0·00535)	0·00305<br>(0·00129–0·00516)
**Latin America and Caribbean**	**All causes**	**16 500<br>(12 500–20 800)**	**10 300<br>(4 780–16 000)**	**6 980<br>(5 030–8 820)**	**6 370<br>(4 660–8 200)**
**Latin America and Caribbean**	**Diarrheal diseases**	**3 580<br>(2 290–5 100)**	**1 310<br>(730–2 110)**	**2 060<br>(-75·7–4 660)**	**1 280<br>(824–1 790)**
**Latin America and Caribbean**	**Lower respiratory infections**	**9 870<br>(7 210–13 300)**	**5 930<br>(256–12 000)**	**1 890<br>(1 090–2 710)**	**5 080<br>(3 600–6 500)**
**Latin America and Caribbean**	**Malaria**	**19·2<br>(-9·6–74·3)**	**13·6<br>(-6·94–46·2)**	**--**	**7·78<br>(-2·72–40·0)**
**Latin America and Caribbean**	**Measles**	**0·0394<br>(0·0172–0·0574)**	**0·0123<br>(0·0056–0·0186)**	**0·00933<br>(0·00331–0·0195)**	**0·022<br>(0·00896–0·0325)**
Andean Latin America	All causes	2 890<br>(2 200–3 770)	1 850<br>(689–3 190)	1 050<br>(899–1 190)	1 160<br>(813–1 510)
Andean Latin America	Diarrheal diseases	252<br>(157–396)	78·6<br>(39·2–132)	134<br>(-4·37–347)	91·8<br>(52·7–135)
Andean Latin America	Lower respiratory infections	2 080<br>(1 490–2 860)	1 210<br>(42·0–2 580)	346<br>(207–487)	1 070<br>(735–1 390)
Andean Latin America	Malaria	0·164<br>(-0·0756–0·722)	0·108<br>(-0·0502–0·389)	--	0·0678<br>(-0·0258–0·415)
Andean Latin America	Measles	<0.001<br>(<0.001–<0.001)	<0.001<br>(<0.001–<0.001)	<0.001<br>(<0.001–<0.001)	<0.001<br>(<0.001–<0.001)
Bolivia (Plurinational State of)	All causes	876<br>(589–1 220)	559<br>(168–1 050)	321<br>(239–401)	349<br>(209–522)
Bolivia (Plurinational State of)	Diarrheal diseases	66·7<br>(25·3–137)	19·7<br>(7·14–44·2)	38·4<br>(-1·31–111)	23·6<br>(9·48–48·0)
Bolivia (Plurinational State of)	Lower respiratory infections	647<br>(384–1 020)	376<br>(15·4–908)	120<br>(61·9–200)	326<br>(189–495)
Bolivia (Plurinational State of)	Malaria	0·0515<br>(-0·0234–0·228)	0·0336<br>(-0·0142–0·123)	--	0·0221<br>(-0·0085–0·126)
Bolivia (Plurinational State of)	Measles	0<br>(0–0)	0<br>(0–0)	0<br>(0–0)	0<br>(0–0)
Ecuador	All causes	860<br>(658–1 090)	554<br>(207–933)	374<br>(318–437)	309<br>(219–418)
Ecuador	Diarrheal diseases	76·4<br>(44·6–113)	22·7<br>(12·0–35·1)	49·7<br>(-1·64–105)	22·7<br>(14·3–31·4)
Ecuador	Lower respiratory infections	617<br>(446–837)	364<br>(13·9–760)	157<br>(94·2–220)	286<br>(197–389)
Ecuador	Malaria	0·00861<br>(-0·00428–0·0385)	0·00565<br>(-0·00274–0·0207)	--	0·00371<br>(-0·00149–0·0223)
Ecuador	Measles	<0.001<br>(<0.001–<0.001)	<0.001<br>(<0.001–<0.001)	<0.001<br>(<0.001–<0.001)	<0.001<br>(<0.001–<0.001)
Peru	All causes	1 160<br>(842–1 560)	738<br>(315–1 310)	351<br>(285–438)	502<br>(344–665)
Peru	Diarrheal diseases	109<br>(69·5–173)	36·1<br>(18·1–67·2)	45·7<br>(-1·43–142)	45·5<br>(26·4–72·8)

Peru	Lower respiratory infections	811 (536–1 210)	465 (12·7–1 040)	68·4 (33·6–106)	456 (307–604)
Peru	Malaria	0·104 (-0·0471–0·453)	0·0693 (-0·0312–0·258)	--	0·042 (-0·0157–0·256)
Peru	Measles	0 (0–0)	0 (0–0)	0 (0–0)	0 (0–0)
Caribbean	All causes	4 500 (3 000–5 920)	2 880 (1 500–4 050)	2 520 (1 470–3 470)	1 450 (956–2 010)
Caribbean	Diarrheal diseases	1 510 (748–2 530)	622 (293–1 150)	1 000 (-45·9–2 140)	441 (227–788)
Caribbean	Lower respiratory infections	2 130 (1 310–2 940)	1 400 (84·1–2 640)	657 (352–1 050)	1 010 (599–1 500)
Caribbean	Malaria	12·3 (-6·58–44·3)	8·88 (-4·67–32·3)	--	5·13 (-1·75–23·1)
Caribbean	Measles	<0.001 (<0.001–0·00104)	<0.001 (<0.001–<0.001)	<0.001 (<0.001–<0.001)	<0.001 (<0.001–<0.001)
Antigua and Barbuda	All causes	0·725 (0·526–0·991)	0·468 (0·193–0·824)	0·371 (0·297–0·453)	0·188 (0·128–0·272)
Antigua and Barbuda	Diarrheal diseases	0·0522 (0·0214–0·0828)	0·0127 (0·00627–0·0202)	0·0395 (-0·00135–0·0801)	0·00954 (0·0056–0·0139)
Antigua and Barbuda	Lower respiratory infections	0·508 (0·345–0·729)	0·291 (0·0104–0·652)	0·167 (0·0924–0·247)	0·179 (0·121–0·261)
Antigua and Barbuda	Malaria	0 (0–0)	0 (0–0)	--	0 (0–0)
Antigua and Barbuda	Measles	0 (0–0)	0 (0–0)	0 (0–0)	0 (0–0)
Bahamas	All causes	2·04 (1·41–2·87)	1·3 (0·422–2·54)	0·896 (0·716–1·12)	0·6 (0·406–0·842)
Bahamas	Diarrheal diseases	0·163 (0·0769–0·262)	0·0438 (0·0221–0·07)	0·117 (-0·00322–0·247)	0·0343 (0·0199–0·0516)
Bahamas	Lower respiratory infections	1·53 (1·02–2·34)	0·912 (0·0302–2·16)	0·435 (0·249–0·649)	0·565 (0·378–0·803)
Bahamas	Malaria	0 (0–0)	0 (0–0)	--	0 (0–0)
Bahamas	Measles	0 (0–0)	0 (0–0)	0 (0–0)	0 (0–0)
Barbados	All causes	0·756 (0·515–1·09)	0·456 (0·153–0·897)	0·35 (0·288–0·428)	0·211 (0·131–0·312)
Barbados	Diarrheal diseases	0·0796 (0·0295–0·139)	0·018 (0·00895–0·0292)	0·0589 (-0·0015–0·136)	0·0161 (0·00943–0·0234)
Barbados	Lower respiratory infections	0·554 (0·368–0·841)	0·315 (0·0102–0·771)	0·169 (0·0948–0·247)	0·195 (0·119–0·289)
Barbados	Malaria	0 (0–0)	0 (0–0)	--	0 (0–0)
Barbados	Measles	0 (0–0)	0 (0–0)	0 (0–0)	0 (0–0)
Belize	All causes	8·28 (6·15–10·6)	5·42 (2·4–8·6)	3·63 (2·94–4·33)	2·91 (1·88–4·06)
Belize	Diarrheal diseases	1·09 (0·629–1·68)	0·328 (0·165–0·522)	0·646 (-0·015–1·53)	0·358 (0·213–0·526)
Belize	Lower respiratory infections	5·21 (3·52–7·18)	3·1 (0·109–6·41)	0·994 (0·557–1·49)	2·55 (1·62–3·6)
Belize	Malaria	0 (0–0)	0 (0–0)	--	0 (0–0)
Belize	Measles	0 (0–0)	0 (0–0)	0 (0–0)	0 (0–0)
Bermuda	All causes	0·0249 (0·0167–0·0378)	0·0142 (0·00379–0·0306)	0·0109 (0·00851–0·0142)	0·00625 (0·00382–0·00959)

Bermuda	Diarrheal diseases	0·00266<br>(<0.001–0·00472)	<0.001<br>(<0.001–<0.001)	0·00204<br>(>-0.001–0·00459)	<0.001<br>(<0.001–<0.001)
Bermuda	Lower respiratory infections	0·0197<br>(0·0128–0·0316)	0·0111<br>(<0.001–0·0275)	0·00628<br>(0·00347–0·00971)	0·00583<br>(0·00346–0·00902)
Bermuda	Malaria	0<br>(0–0)	0<br>(0–0)	--	0<br>(0–0)
Bermuda	Measles	<0.001<br>(<0.001–<0.001)	<0.001<br>(<0.001–<0.001)	<0.001<br>(<0.001–<0.001)	<0.001<br>(<0.001–<0.001)
Cuba	All causes	33·4<br>(21·6–51·5)	17·9<br>(3·32–40·5)	10·2<br>(7·38–12·9)	13·1<br>(9·38–17·7)
Cuba	Diarrheal diseases	3·3<br>(1·43–5·89)	0·714<br>(0·333–1·22)	2·27<br>(-0·057–5·53)	0·759<br>(0·42–1·16)
Cuba	Lower respiratory infections	28·2<br>(18·0–43·8)	15·3<br>(0·475–38·0)	5·92<br>(3·07–9·15)	12·3<br>(8·82–16·7)
Cuba	Malaria	0<br>(0–0)	0<br>(0–0)	--	0<br>(0–0)
Cuba	Measles	0<br>(0–0)	0<br>(0–0)	0<br>(0–0)	0<br>(0–0)
Dominica	All causes	0·296<br>(0·174–0·444)	0·189<br>(0·0809–0·365)	0·149<br>(0·105–0·198)	0·0776<br>(0·0448–0·125)
Dominica	Diarrheal diseases	0·0437<br>(0·0161–0·0834)	0·0116<br>(0·0041–0·0236)	0·0312<br>(>-0.001–0·0784)	0·00919<br>(0·00358–0·0172)
Dominica	Lower respiratory infections	0·187<br>(0·0999–0·324)	0·112<br>(0·00284–0·292)	0·0528<br>(0·0262–0·0896)	0·0684<br>(0·0382–0·112)
Dominica	Malaria	0<br>(0–0)	0<br>(0–0)	--	0<br>(0–0)
Dominica	Measles	0<br>(0–0)	0<br>(0–0)	0<br>(0–0)	0<br>(0–0)
Dominican Republic	All causes	206<br>(141–295)	129<br>(66·9–218)	106<br>(76·2–151)	55·2<br>(29·3–86·7)
Dominican Republic	Diarrheal diseases	45·1<br>(20·0–79·1)	12·8<br>(5·88–22·3)	29·8<br>(-0·866–74·0)	11·2<br>(6·08–18·3)
Dominican Republic	Lower respiratory infections	111<br>(67·3–166)	67·2<br>(2·34–153)	27·0<br>(12·5–45·9)	44·0<br>(20·9–69·9)
Dominican Republic	Malaria	0·001<br>(>-0.001–0·00457)	<0.001<br>(>-0.001–0·00365)	--	<0.001<br>(>-0.001–0·00166)
Dominican Republic	Measles	0<br>(0–0)	0<br>(0–0)	0<br>(0–0)	0<br>(0–0)
Grenada	All causes	1·05<br>(0·741–1·48)	0·672<br>(0·179–1·29)	0·404<br>(0·302–0·521)	0·351<br>(0·236–0·5)
Grenada	Diarrheal diseases	0·0452<br>(0·023–0·0699)	0·0132<br>(0·00657–0·0217)	0·0313<br>(-0·00101–0·0657)	0·0102<br>(0·00558–0·0153)
Grenada	Lower respiratory infections	0·863<br>(0·579–1·26)	0·521<br>(0·0194–1·13)	0·235<br>(0·132–0·35)	0·341<br>(0·229–0·488)
Grenada	Malaria	0<br>(0–0)	0<br>(0–0)	--	0<br>(0–0)
Grenada	Measles	0<br>(0–0)	0<br>(0–0)	0<br>(0–0)	0<br>(0–0)
Guyana	All causes	38·4<br>(27·9–49·8)	27·5<br>(16·0–39·5)	25·0<br>(19·1–30·5)	8·11<br>(5·65–11·1)
Guyana	Diarrheal diseases	7·13<br>(3·99–10·5)	2·54<br>(1·31–4·19)	5·16<br>(-0·221–9·99)	1·56<br>(0·874–2·48)
Guyana	Lower respiratory infections	17·3<br>(11·9–23·1)	11·1<br>(0·48–21·6)	6·28<br>(3·67–9·18)	6·42<br>(4·14–9·07)
Guyana	Malaria	0·48<br>(-0·253–1·94)	0·381<br>(-0·204–1·42)	--	0·136<br>(-0·0466–0·845)
Guyana	Measles	0<br>(0–0)	0<br>(0–0)	0<br>(0–0)	0<br>(0–0)

Haiti	All causes	4 020<br>(2 670–5 320)	2 580<br>(1 360–3 610)	2 260<br>(1 290–3 170)	1 310<br>(868–1 840)
Haiti	Diarrheal diseases	1 390<br>(690–2 360)	582<br>(273–1 080)	923<br>(-42·9–2 000)	410<br>(209–739)
Haiti	Lower respiratory infections	1 870<br>(1 070–2 630)	1 240<br>(77·3–2 340)	588<br>(302–956)	899<br>(525–1 360)
Haiti	Malaria	11·4<br>(-6·08–41·0)	8·21<br>(-4·32–30·0)	--	4·82<br>(-1·65–21·5)
Haiti	Measles	0<br>(0–0)	0<br>(0–0)	0<br>(0–0)	0<br>(0–0)
Jamaica	All causes	13·6<br>(9·49–18·8)	8·48<br>(4·97–13·0)	7·78<br>(5·42–10·6)	3·26<br>(1·99–4·75)
Jamaica	Diarrheal diseases	3·32<br>(1·49–5·59)	0·844<br>(0·391–1·41)	2·26<br>(-0·0622–5·21)	0·782<br>(0·432–1·15)
Jamaica	Lower respiratory infections	6·29<br>(4·03–9·5)	3·65<br>(0·0948–8·52)	1·53<br>(0·84–2·35)	2·48<br>(1·48–3·77)
Jamaica	Malaria	0<br>(0–0)	0<br>(0–0)	--	0<br>(0–0)
Jamaica	Measles	0<br>(0–0)	0<br>(0–0)	0<br>(0–0)	0<br>(0–0)
Puerto Rico	All causes	4·14<br>(2·76–6·06)	2·49<br>(1·28–4·38)	2·36<br>(1·77–2·97)	0·878<br>(0·616–1·24)
Puerto Rico	Diarrheal diseases	0·814<br>(0·262–1·47)	0·163<br>(0·083–0·26)	0·623<br>(-0·015–1·43)	0·131<br>(0·0767–0·195)
Puerto Rico	Lower respiratory infections	2·25<br>(1·43–3·68)	1·25<br>(0·0355–3·24)	0·657<br>(0·368–0·981)	0·747<br>(0·53–1·07)
Puerto Rico	Malaria	0<br>(0–0)	0<br>(0–0)	--	0<br>(0–0)
Puerto Rico	Measles	<0.001<br>(<0.001–0·001)	<0.001<br>(<0.001–<0.001)	<0.001<br>(<0.001–<0.001)	<0.001<br>(<0.001–<0.001)
Saint Kitts and Nevis	All causes	0·405<br>(0·279–0·542)	0·272<br>(0·153–0·422)	0·239<br>(0·187–0·284)	0·0862<br>(0·0558–0·121)
Saint Kitts and Nevis	Diarrheal diseases	0·07<br>(0·0321–0·115)	0·0187<br>(0·00938–0·0295)	0·05<br>(-0·00146–0·109)	0·0144<br>(0·00817–0·0209)
Saint Kitts and Nevis	Lower respiratory infections	0·203<br>(0·129–0·294)	0·122<br>(0·00427–0·27)	0·0578<br>(0·0332–0·0843)	0·0718<br>(0·0457–0·102)
Saint Kitts and Nevis	Malaria	0<br>(0–0)	0<br>(0–0)	--	0<br>(0–0)
Saint Kitts and Nevis	Measles	0<br>(0–0)	0<br>(0–0)	0<br>(0–0)	0<br>(0–0)
Saint Lucia	All causes	1·01<br>(0·705–1·44)	0·69<br>(0·366–1·13)	0·599<br>(0·472–0·746)	0·173<br>(0·117–0·248)
Saint Lucia	Diarrheal diseases	0·164<br>(0·0645–0·28)	0·0433<br>(0·0216–0·0678)	0·123<br>(-0·00349–0·271)	0·0234<br>(0·0126–0·0348)
Saint Lucia	Lower respiratory infections	0·536<br>(0·338–0·83)	0·337<br>(0·013–0·757)	0·166<br>(0·0938–0·254)	0·15<br>(0·102–0·216)
Saint Lucia	Malaria	0<br>(0–0)	0<br>(0–0)	--	0<br>(0–0)
Saint Lucia	Measles	0<br>(0–0)	0<br>(0–0)	0<br>(0–0)	0<br>(0–0)
Saint Vincent and the Grenadines	All causes	1·46<br>(1·06–1·93)	1·04<br>(0·572–1·66)	0·846<br>(0·692–1·05)	0·328<br>(0·213–0·463)
Saint Vincent and the Grenadines	Diarrheal diseases	0·144<br>(0·0665–0·24)	0·0397<br>(0·0204–0·0619)	0·102<br>(-0·00263–0·229)	0·0309<br>(0·0176–0·0456)
Saint Vincent and the Grenadines	Lower respiratory infections	0·779<br>(0·497–1·14)	0·471<br>(0·0166–1·02)	0·212<br>(0·11–0·326)	0·297<br>(0·183–0·431)
Saint Vincent and the Grenadines	Malaria	0<br>(0–0)	0<br>(0–0)	--	0<br>(0–0)

Saint Vincent and the Grenadines	Measles	0 (0–0)	0 (0–0)	0 (0–0)	0 (0–0)
Suriname	All causes	18·8 (11·0–26·0)	11·3 (5·39–17·2)	10·9 (5·98–16·3)	4·31 (2·7–6·24)
Suriname	Diarrheal diseases	6·26 (2·7–10·7)	2·07 (0·957–3·91)	4·56 (-0·157–10·3)	1·18 (0·578–2·08)
Suriname	Lower respiratory infections	9·32 (5·65–14·0)	6·06 (0·21–12·3)	3·1 (1·65–4·85)	3·13 (1·75–4·6)
Suriname	Malaria	<0.001 (>-0.001–<0.001)	<0.001 (>-0.001–<0.001)	--	<0.001 (>-0.001–<0.001)
Suriname	Measles	0 (0–0)	0 (0–0)	0 (0–0)	0 (0–0)
Trinidad and Tobago	All causes	5·91 (4·13–7·65)	3·73 (1·54–6·19)	2·92 (2·2–3·5)	1·77 (1·15–2·55)
Trinidad and Tobago	Diarrheal diseases	1·13 (0·55–1·74)	0·354 (0·174–0·573)	0·778 (-0·0234–1·62)	0·28 (0·158–0·414)
Trinidad and Tobago	Lower respiratory infections	3·68 (2·47–5·17)	2·27 (0·0961–4·79)	1·04 (0·603–1·52)	1·49 (0·904–2·18)
Trinidad and Tobago	Malaria	0 (0–0)	0 (0–0)	--	0 (0–0)
Trinidad and Tobago	Measles	0 (0–0)	0 (0–0)	0 (0–0)	0 (0–0)
United States Virgin Islands	All causes	0·212 (0·142–0·295)	0·141 (0·0936–0·198)	0·149 (0·1–0·209)	0·0342 (0·0227–0·0497)
United States Virgin Islands	Diarrheal diseases	0·0552 (0·0211–0·0975)	0·0125 (0·00591–0·0206)	0·0406 (-0·00113–0·0927)	0·0104 (0·00557–0·0154)
United States Virgin Islands	Lower respiratory infections	0·0665 (0·0404–0·107)	0·0381 (0·00112–0·0954)	0·0188 (0·01–0·0296)	0·0238 (0·0157–0·0354)
United States Virgin Islands	Malaria	0 (0–0)	0 (0–0)	--	0 (0–0)
United States Virgin Islands	Measles	0 (0–0)	0 (0–0)	0 (0–0)	0 (0–0)
Central Latin America	All causes	7 080 (5 540–8 790)	4 380 (2 040–6 910)	2 650 (2 000–3 500)	3 020 (2 150–3 890)
Central Latin America	Diarrheal diseases	1 530 (1 110–2 040)	537 (295–799)	733 (-20·5–1 800)	663 (447–852)
Central Latin America	Lower respiratory infections	4 220 (3 150–5 650)	2 520 (104–5 150)	592 (345–830)	2 350 (1 560–3 060)
Central Latin America	Malaria	5·55 (-2·43–23·0)	3·8 (-1·77–13·5)	--	2·15 (-0·778–12·1)
Central Latin America	Measles	<0.001 (<0.001–0·00103)	<0.001 (<0.001–<0.001)	<0.001 (<0.001–<0.001)	<0.001 (<0.001–<0.001)
Colombia	All causes	877 (684–1 120)	647 (417–935)	530 (461–613)	190 (142–259)
Colombia	Diarrheal diseases	90·9 (48·1–156)	24·1 (12·0–37·6)	58·0 (-1·59–145)	23·4 (14·0–32·9)
Colombia	Lower respiratory infections	390 (258–595)	228 (7·38–539)	78·0 (44·9–111)	166 (120–228)
Colombia	Malaria	2·33 (-0·937–8·21)	1·72 (-0·685–5·64)	--	0·719 (-0·243–3·5)
Colombia	Measles	0 (0–0)	0 (0–0)	0 (0–0)	0 (0–0)
Costa Rica	All causes	8·71 (5·46–16·6)	4·2 (0·789–12·4)	3·37 (2·57–4·26)	2·43 (1·74–3·36)
Costa Rica	Diarrheal diseases	1·08 (0·304–2·48)	0·14 (0·0682–0·23)	0·848 (-0·0196–2·43)	0·149 (0·085–0·223)
Costa Rica	Lower respiratory infections	7·1 (4·47–13·7)	3·52 (0·0789–11·8)	1·98 (1·08–2·96)	2·28 (1·62–3·18)

Costa Rica	Malaria	<0.001 (>-0.001–<0.001)	<0.001 (>-0.001–<0.001)	--	<0.001 (>-0.001–<0.001)
Costa Rica	Measles	0 (0–0)	0 (0–0)	0 (0–0)	0 (0–0)
El Salvador	All causes	102 (72·9–142)	63·3 (26·8–110)	38·6 (28·5–51·5)	38·4 (25·7–53·5)
El Salvador	Diarrheal diseases	17·9 (9·82–28·0)	5·76 (2·74–10·1)	10·4 (-0·288–24·7)	5·72 (3·25–8·86)
El Salvador	Lower respiratory infections	68·3 (45·5–99·7)	41·7 (1·41–89·8)	12·4 (6·87–18·9)	32·6 (20·9–45·7)
El Salvador	Malaria	0 (0–0)	0 (0–0)	--	0 (0–0)
El Salvador	Measles	0 (0–0)	0 (0–0)	0 (0–0)	0 (0–0)
Guatemala	All causes	2 030 (1 630–2 440)	1 250 (502–1 870)	525 (348–811)	1 090 (770–1 450)
Guatemala	Diarrheal diseases	530 (419–648)	225 (130–342)	203 (-5·3–541)	275 (194–366)
Guatemala	Lower respiratory infections	1 310 (1 010–1 650)	838 (41·0–1 500)	137 (73·6–207)	820 (536–1 090)
Guatemala	Malaria	<0.001 (>-0.001–0·00319)	<0.001 (>-0.001–0·0019)	--	<0.001 (>-0.001–0·002)
Guatemala	Measles	0 (0–0)	0 (0–0)	0 (0–0)	0 (0–0)
Honduras	All causes	180 (121–262)	106 (50·7–176)	72·6 (39·9–121)	70·6 (47·9–102)
Honduras	Diarrheal diseases	61·8 (31·5–109)	22·3 (9·67–41·9)	33·0 (-0·811–87·4)	21·7 (10·4–39·3)
Honduras	Lower respiratory infections	93·0 (54·7–148)	58·3 (2·65–133)	14·5 (7·16–23·8)	48·9 (31·1–73·3)
Honduras	Malaria	0·0128 (-0·00642–0·0629)	0·00914 (-0·00445–0·0456)	--	0·00509 (-0·00184–0·0237)
Honduras	Measles	0 (0–0)	0 (0–0)	0 (0–0)	0 (0–0)
Mexico	All causes	2 720 (2 110–3 440)	1 610 (645–2 740)	901 (695–1 190)	1 230 (857–1 620)
Mexico	Diarrheal diseases	531 (384–732)	177 (96·2–267)	250 (-6·39–643)	233 (155–307)
Mexico	Lower respiratory infections	1 770 (1 310–2 440)	1 020 (39·5–2 180)	239 (134–350)	996 (653–1 320)
Mexico	Malaria	<0.001 (>-0.001–<0.001)	<0.001 (>-0.001–<0.001)	--	<0.001 (>-0.001–<0.001)
Mexico	Measles	0 (0–0)	0 (0–0)	0 (0–0)	0 (0–0)
Nicaragua	All causes	186 (131–256)	119 (60·0–195)	92·2 (67·9–122)	53·4 (31·4–76·7)
Nicaragua	Diarrheal diseases	36·0 (18·3–60·5)	10·2 (4·83–17·6)	22·5 (-0·602–56·0)	10·1 (5·87–15·5)
Nicaragua	Lower respiratory infections	102 (66·0–149)	61·2 (2–135)	21·7 (12·2–34·2)	43·2 (24·0–63·1)
Nicaragua	Malaria	0·184 (-0·0892–0·844)	0·129 (-0·0623–0·521)	--	0·0683 (-0·0288–0·391)
Nicaragua	Measles	0 (0–0)	0 (0–0)	0 (0–0)	0 (0–0)
Panama	All causes	115 (86·9–154)	71·7 (30·6–127)	43·2 (35·2–55·2)	43·8 (29·6–59·4)
Panama	Diarrheal diseases	17·1 (10·2–26·7)	4·92 (2·53–7·88)	9·12 (-0·205–24·2)	6·11 (3·75–8·62)

Panama	Lower respiratory infections	74·7 (51·3–108)	43·3 (1·39–99·7)	10·6 (5·76–15·7)	37·7 (24·8–51·9)
Panama	Malaria	<0.001 (>-0.001–0·00193)	<0.001 (>-0.001–0·00139)	--	<0.001 (>-0.001–<0.001)
Panama	Measles	0 (0–0)	0 (0–0)	0 (0–0)	0 (0–0)
Venezuela (Bolivarian Republic of)	All causes	870 (632–1 150)	516 (314–761)	445 (297–633)	297 (198–398)
Venezuela (Bolivarian Republic of)	Diarrheal diseases	247 (125–412)	67·4 (30·0–121)	147 (-4·71–362)	88·6 (49·7–140)
Venezuela (Bolivarian Republic of)	Lower respiratory infections	398 (271–531)	225 (8·13–477)	76·4 (44·3–111)	207 (132–286)
Venezuela (Bolivarian Republic of)	Malaria	3·02 (-1·52–13·1)	1·95 (-1·01–9·14)	--	1·36 (-0·463–7·18)
Venezuela (Bolivarian Republic of)	Measles	<0.001 (<0.001–0·00103)	<0.001 (<0.001–<0.001)	<0.001 (<0.001–<0.001)	<0.001 (<0.001–<0.001)
Tropical Latin America	All causes	2 030 (1 440–2 820)	1 180 (402–2 230)	771 (619–919)	735 (524–991)
Tropical Latin America	Diarrheal diseases	289 (154–462)	73·0 (36·1–112)	185 (-4·87–435)	81·9 (49·8–114)
Tropical Latin America	Lower respiratory infections	1 450 (1 010–2 110)	817 (26·7–1 880)	295 (168–430)	652 (468–878)
Tropical Latin America	Malaria	1·17 (-0·511–4·47)	0·805 (-0·341–2·84)	--	0·435 (-0·145–2·21)
Tropical Latin America	Measles	0·0381 (0·0165–0·0557)	0·0119 (0·00539–0·0181)	0·00899 (0·00321–0·0188)	0·0213 (0·00866–0·0316)
Brazil	All causes	1 940 (1 380–2 700)	1 120 (370–2 140)	731 (583–872)	709 (503–956)
Brazil	Diarrheal diseases	277 (147–442)	69·5 (34·5–106)	179 (-4·73–418)	78·2 (47·6–108)
Brazil	Lower respiratory infections	1 400 (971–2 030)	789 (25·9–1 810)	288 (165–421)	630 (451–848)
Brazil	Malaria	1·17 (-0·511–4·47)	0·805 (-0·341–2·84)	--	0·435 (-0·145–2·21)
Brazil	Measles	0·0381 (0·0165–0·0557)	0·0119 (0·00539–0·0181)	0·00899 (0·00321–0·0188)	0·0213 (0·00866–0·0316)
Paraguay	All causes	88·1 (65·2–123)	58·4 (32·0–101)	39·9 (31·6–49·8)	26·1 (16·3–37·7)
Paraguay	Diarrheal diseases	12·0 (6·3–20·2)	3·46 (1·49–5·91)	6·53 (-0·135–18·0)	3·73 (1·86–6)
Paraguay	Lower respiratory infections	49·2 (30·9–78·1)	28·1 (0·836–66·9)	6·5 (3·54–9·67)	22·3 (14·2–32·6)
Paraguay	Malaria	0 (0–0)	0 (0–0)	--	0 (0–0)
Paraguay	Measles	0 (0–0)	0 (0–0)	0 (0–0)	0 (0–0)
**North Africa and Middle East**	**All causes**	**30 600 (21 900–39 400)**	**19 300 (9 290–29 400)**	**15 900 (11 400–20 700)**	**12 600 (9 160–16 600)**
**North Africa and Middle East**	**Diarrheal diseases**	**6 410 (3 260–11 100)**	**2 780 (1 340–5 090)**	**4 500 (-216–9 740)**	**2 240 (1 150–3 860)**
**North Africa and Middle East**	**Lower respiratory infections**	**16 600 (11 400–23 000)**	**10 600 (698–20 600)**	**6 330 (3 670–9 520)**	**8 440 (5 490–11 900)**
**North Africa and Middle East**	**Malaria**	**472 (-357–1 400)**	**366 (-253–1 070)**	**--**	**200 (-82·7–827)**
**North Africa and Middle East**	**Measles**	**3 200 (1 130–6 630)**	**1 570 (568–3 260)**	**1 160 (373–2 460)**	**1 750 (568–3 640)**
North Africa and Middle East	All causes	30 600 (21 900–39 400)	19 300 (9 290–29 400)	15 900 (11 400–20 700)	12 600 (9 160–16 600)

North Africa and Middle East	Diarrheal diseases	6 410 (3 260–11 100)	2 780 (1 340–5 090)	4 500 (-216–9 740)	2 240 (1 150–3 860)
North Africa and Middle East	Lower respiratory infections	16 600 (11 400–23 000)	10 600 (698–20 600)	6 330 (3 670–9 520)	8 440 (5 490–11 900)
North Africa and Middle East	Malaria	472 (-357–1 400)	366 (-253–1 070)	--	200 (-82·7–827)
North Africa and Middle East	Measles	3 200 (1 130–6 630)	1 570 (568–3 260)	1 160 (373–2 460)	1 750 (568–3 640)
Afghanistan	All causes	13 600 (9 650–17 000)	9 280 (5 450–12 500)	7 830 (5 180–10 600)	5 370 (3 590–7 270)
Afghanistan	Diarrheal diseases	3 340 (1 530–5 970)	1 690 (736–3 120)	2 270 (-105–5 010)	1 290 (577–2 290)
Afghanistan	Lower respiratory infections	5 430 (3 470–7 720)	3 770 (262–6 910)	2 080 (1 160–3 340)	2 950 (1 710–4 580)
Afghanistan	Malaria	6·87 (-4·83–34·6)	4·67 (-2·72–22·9)	--	3·68 (-1·99–19·4)
Afghanistan	Measles	2 030 (735–4 030)	1 040 (368–2 190)	716 (217–1 560)	1 120 (346–2 350)
Algeria	All causes	717 (473–1 030)	343 (118–681)	311 (204–443)	284 (176–414)
Algeria	Diarrheal diseases	109 (35·6–238)	28·7 (10·9–58·0)	80·6 (-2·71–221)	27·2 (12·0–54·5)
Algeria	Lower respiratory infections	419 (254–672)	237 (9·6–567)	145 (73·5–254)	176 (94·2–299)
Algeria	Malaria	0 (0–0)	0 (0–0)	--	0 (0–0)
Algeria	Measles	159 (52·7–324)	47·5 (15·7–102)	55·6 (15·2–122)	81·5 (27·2–167)
Bahrain	All causes	2·97 (1·71–4·3)	1·63 (0·478–3·17)	1·48 (0·991–1·97)	0·855 (0·557–1·3)
Bahrain	Diarrheal diseases	0·527 (0·176–1)	0·121 (0·0515–0·233)	0·407 (-0·0164–0·915)	0·0954 (0·0464–0·167)
Bahrain	Lower respiratory infections	2·1 (1·23–3·24)	1·17 (0·0478–2·73)	0·73 (0·385–1·23)	0·76 (0·488–1·15)
Bahrain	Malaria	0 (0–0)	0 (0–0)	--	0 (0–0)
Bahrain	Measles	<0.001 (0–0·00111)	<0.001 (0–<0.001)	<0.001 (0–<0.001)	<0.001 (0–<0.001)
Egypt	All causes	5 520 (3 750–7 800)	2 760 (618–5 680)	2 540 (1 740–3 450)	2 360 (1 670–3 230)
Egypt	Diarrheal diseases	1 150 (498–1 990)	299 (143–551)	841 (-33–1 830)	349 (181–574)
Egypt	Lower respiratory infections	4 120 (2 920–5 950)	2 250 (87·4–5 250)	1 480 (861–2 150)	1 980 (1 350–2 770)
Egypt	Malaria	0 (0–0)	0 (0–0)	--	0 (0–0)
Egypt	Measles	54·7 (17·1–117)	14·6 (4·4–35·0)	20·1 (4·9–47·0)	30·5 (9·51–67·0)
Iran (Islamic Republic of)	All causes	271 (166–411)	151 (36·4–305)	123 (83·6–161)	79·9 (50·4–123)
Iran (Islamic Republic of)	Diarrheal diseases	53·9 (21·9–92·3)	14·7 (7·27–26·4)	40·1 (-1·48–87·5)	10·8 (6·06–18·2)
Iran (Islamic Republic of)	Lower respiratory infections	198 (120–309)	117 (4·87–273)	63·9 (35·2–103)	69·1 (42·9–107)
Iran (Islamic Republic of)	Malaria	<0.001 (>-0.001–<0.001)	<0.001 (>-0.001–<0.001)	--	<0.001 (>-0.001–<0.001)
Iran (Islamic Republic of)	Measles	0·0406 (0·0205–0·0594)	0·0151 (0·00752–0·0227)	0·0155 (0·00625–0·0293)	0·018 (0·00802–0·0275)

Iraq	All causes	1 240 (828–1 730)	641 (240–1 170)	594 (408–844)	501 (331–722)
Iraq	Diarrheal diseases	265 (114–511)	79·0 (32·7–154)	191 (-6·64–473)	77·9 (37·1–147)
Iraq	Lower respiratory infections	728 (467–1 130)	418 (19·3–962)	252 (127–430)	344 (200–561)
Iraq	Malaria	0 (0–0)	0 (0–0)	--	0 (0–0)
Iraq	Measles	148 (48·5–300)	45·4 (14·7–101)	52·3 (14·2–118)	79·1 (27·0–161)
Jordan	All causes	137 (83·7–227)	72·1 (9·43–179)	43·9 (31·3–63·2)	54·6 (33·0–85·3)
Jordan	Diarrheal diseases	12·2 (4·73–22·6)	2·59 (1·14–4·95)	8·61 (-0·206–20·7)	2·98 (1·57–4·9)
Jordan	Lower respiratory infections	120 (72·3–202)	64·7 (1·97–173)	30·5 (15·2–53·1)	51·6 (31·0–81·6)
Jordan	Malaria	0 (0–0)	0 (0–0)	--	0 (0–0)
Jordan	Measles	0·0276 (0·0117–0·0391)	0·00802 (0·00349–0·0122)	0·00744 (0·00256–0·0143)	0·0149 (0·00613–0·0224)
Kuwait	All causes	19·5 (12·5–30·2)	11·0 (0·584–26·4)	5·02 (3·32–6·84)	7·11 (5·18–9·8)
Kuwait	Diarrheal diseases	0·593 (0·239–0·995)	0·142 (0·0705–0·229)	0·415 (-0·0114–0·941)	0·121 (0·0697–0·177)
Kuwait	Lower respiratory infections	18·9 (12·1–29·3)	10·8 (0·326–26·2)	4·52 (2·54–6·51)	6·99 (5·08–9·66)
Kuwait	Malaria	0 (0–0)	0 (0–0)	--	0 (0–0)
Kuwait	Measles	<0.001 (<0.001–<0.001)	<0.001 (<0.001–<0.001)	<0.001 (<0.001–<0.001)	<0.001 (<0.001–<0.001)
Lebanon	All causes	112 (75·8–154)	48·3 (10·3–99·0)	42·0 (28·8–58·1)	52·7 (34·7–74·8)
Lebanon	Diarrheal diseases	21·6 (8·1–40·1)	4·61 (1·92–9·06)	15·1 (-0·36–38·0)	6·9 (3·49–12·3)
Lebanon	Lower respiratory infections	79·1 (52·1–110)	39·9 (1·55–91·8)	22·1 (11·9–35·1)	40·5 (24·0–59·7)
Lebanon	Malaria	0 (0–0)	0 (0–0)	--	0 (0–0)
Lebanon	Measles	9·12 (3·26–17·5)	2·13 (0·729–4·39)	3·05 (0·807–7·21)	5·27 (1·76–11·2)
Libya	All causes	103 (69·3–154)	57·0 (10·5–120)	40·9 (27·6–59·0)	49·2 (32·3–74·1)
Libya	Diarrheal diseases	7·91 (2·27–18·1)	2·11 (0·603–4·78)	5·61 (-0·287–16·1)	2·54 (0·808–5·34)
Libya	Lower respiratory infections	84·3 (51·7–132)	46·0 (1·9–107)	26·2 (13·0–47·4)	44·8 (28·3–70·5)
Libya	Malaria	0 (0–0)	0 (0–0)	--	0 (0–0)
Libya	Measles	3·24 (1·02–7·61)	0·906 (0·284–2·28)	1·11 (0·273–2·73)	1·85 (0·577–4·43)
Morocco	All causes	615 (385–937)	264 (84·0–559)	252 (131–458)	255 (169–373)
Morocco	Diarrheal diseases	183 (60·8–379)	42·2 (15·3–83·3)	126 (-4·71–365)	52·2 (22·5–97·6)
Morocco	Lower respiratory infections	353 (212–583)	190 (7·21–500)	92·6 (42·8–159)	166 (97·0–259)
Morocco	Malaria	0 (0–0)	0 (0–0)	--	0 (0–0)

Morocco	Measles	63·1<br>(19·9–133)	16·3<br>(5–36·4)	17·8<br>(4·37–43·8)	37·0<br>(11·6–81·0)
Oman	All causes	32·6<br>(18·9–49·3)	20·0<br>(4·59–37·5)	15·9<br>(10·4–21·9)	9·86<br>(6·17–15·7)
Oman	Diarrheal diseases	6·25<br>(2·35–11·8)	2·24<br>(0·904–4·98)	4·67<br>(-0·26–11·3)	1·24<br>(0·52–2·5)
Oman	Lower respiratory infections	24·5<br>(14·3–38·1)	15·9<br>(0·899–33·9)	9·41<br>(5·32–15·8)	8·62<br>(5·26–14·4)
Oman	Malaria	<0.001<br>(>-0.001–<0.001)	<0.001<br>(>-0.001–<0.001)	--	<0.001<br>(>-0.001–<0.001)
Oman	Measles	0·0127<br>(0·00552–0·0207)	0·00546<br>(0·00232–0·00856)	0·00529<br>(0·00189–0·01)	0·00467<br>(0·00185–0·00771)
Palestine	All causes	55·7<br>(38·5–82·6)	26·1<br>(5·65–59·9)	16·4<br>(12·4–21·4)	24·7<br>(17·3–34·5)
Palestine	Diarrheal diseases	5·39<br>(2·05–10·2)	1·09<br>(0·507–1·95)	3·72<br>(-0·0871–9·71)	1·38<br>(0·759–2·28)
Palestine	Lower respiratory infections	42·0<br>(28·1–66·9)	21·5<br>(0·701–56·3)	9·15<br>(4·7–14·9)	19·6<br>(12·9–29·0)
Palestine	Malaria	0<br>(0–0)	0<br>(0–0)	--	0<br>(0–0)
Palestine	Measles	6·49<br>(2·01–13·4)	1·64<br>(0·5–3·65)	1·69<br>(0·422–4·02)	3·71<br>(1·15–8·03)
Qatar	All causes	5·15<br>(3·11–8·57)	2·48<br>(0·395–5·97)	1·9<br>(1·35–2·57)	1·76<br>(1·23–2·76)
Qatar	Diarrheal diseases	0·566<br>(0·166–1·14)	0·0997<br>(0·0413–0·197)	0·438<br>(-0·0142–1·1)	0·0975<br>(0·0498–0·172)
Qatar	Lower respiratory infections	4·25<br>(2·52–6·76)	2·2<br>(0·0801–5·75)	1·27<br>(0·683–2·13)	1·57<br>(1·03–2·53)
Qatar	Malaria	0<br>(0–0)	0<br>(0–0)	--	0<br>(0–0)
Qatar	Measles	0·208<br>(0·0602–0·466)	0·055<br>(0·016–0·124)	0·0748<br>(0·0184–0·186)	0·0954<br>(0·0292–0·218)
Saudi Arabia	All causes	430<br>(297–591)	205<br>(34·5–436)	223<br>(161–294)	132<br>(87·6–193)
Saudi Arabia	Diarrheal diseases	65·8<br>(17·4–113)	12·8<br>(5·72–24·8)	54·5<br>(-2·03–111)	10·7<br>(5·62–19·6)
Saudi Arabia	Lower respiratory infections	350<br>(255–479)	180<br>(6·99–414)	156<br>(92·5–237)	121<br>(81·2–176)
Saudi Arabia	Malaria	<0.001<br>(>-0.001–0·00137)	<0.001<br>(>-0.001–<0.001)	--	<0.001<br>(>-0.001–<0.001)
Saudi Arabia	Measles	1·95<br>(0·527–4·52)	0·543<br>(0·149–1·31)	1·01<br>(0·227–2·53)	0·695<br>(0·195–1·66)
Sudan	All causes	2 620<br>(1 280–3 810)	1 780<br>(361–2 930)	1 270<br>(854–1 780)	1 040<br>(667–1 410)
Sudan	Diarrheal diseases	451<br>(188–891)	215<br>(95·7–417)	336<br>(-26·5–766)	127<br>(55·6–248)
Sudan	Lower respiratory infections	1 440<br>(875–2 140)	1 010<br>(81·7–1 900)	646<br>(338–1 060)	676<br>(422–1 050)
Sudan	Malaria	312<br>(-204–930)	243<br>(-138–688)	--	126<br>(-57·7–543)
Sudan	Measles	237<br>(77·6–521)	127<br>(40·6–282)	108<br>(30·0–250)	110<br>(34·9–234)
Syrian Arab Republic	All causes	199<br>(146–259)	104<br>(37·2–172)	86·2<br>(61·4–115)	106<br>(73·4–139)
Syrian Arab Republic	Diarrheal diseases	17·2<br>(7·4–30·8)	5·71<br>(2·65–12·1)	12·4<br>(-0·491–27·1)	6·25<br>(3·09–11·3)
Syrian Arab Republic	Lower respiratory infections	126<br>(84·3–173)	72·8<br>(3·25–140)	44·4<br>(21·8–72·6)	73·9<br>(49·3–108)

Syrian Arab Republic	Malaria	0 (0–0)	0 (0–0)	--	0 (0–0)
Syrian Arab Republic	Measles	45·0 (16·0–89·1)	15·2 (5·01–32·9)	18·7 (5·21–39·1)	25·4 (8·2–51·6)
Tunisia	All causes	187 (121–270)	87·5 (32·5–169)	85·9 (50·3–130)	68·0 (44·5–101)
Tunisia	Diarrheal diseases	51·6 (17·2–99·9)	11·6 (4·85–23·2)	36·7 (-0·838–91·0)	13·6 (6·67–25·3)
Tunisia	Lower respiratory infections	108 (67·5–170)	56·8 (2·08–145)	29·5 (15·2–48·7)	48·7 (29·3–76·6)
Tunisia	Malaria	0 (0–0)	0 (0–0)	--	0 (0–0)
Tunisia	Measles	9·94 (3·21–21·0)	2·24 (0·696–5·05)	2·9 (0·725–7·04)	5·75 (1·86–12·7)
Türkiye	All causes	340 (232–501)	171 (68·6–338)	148 (105–209)	117 (78·0–170)
Türkiye	Diarrheal diseases	56·1 (20·1–115)	10·2 (4·26–19·1)	39·9 (-0·827–106)	13·0 (6·97–21·2)
Türkiye	Lower respiratory infections	204 (126–329)	102 (2·94–276)	49·8 (24·4–85·2)	88·4 (56·8–142)
Türkiye	Malaria	0 (0–0)	0 (0–0)	--	0 (0–0)
Türkiye	Measles	26·9 (8·21–56·3)	6·9 (2·08–15·5)	5·88 (1·38–15·0)	16·0 (4·97–33·6)
United Arab Emirates	All causes	32·4 (22·1–44·1)	18·0 (4·71–32·8)	16·6 (12·0–22·6)	11·2 (7·49–16·4)
United Arab Emirates	Diarrheal diseases	4·48 (1·67–8·33)	1·39 (0·562–2·99)	3·49 (-0·15–7·95)	0·954 (0·413–1·88)
United Arab Emirates	Lower respiratory infections	23·5 (14·6–32·8)	14·1 (0·7–29·7)	10·1 (5·76–16·2)	9·17 (5·82–14·0)
United Arab Emirates	Malaria	0 (0–0)	0 (0–0)	--	0 (0–0)
United Arab Emirates	Measles	3·03 (0·958–6·23)	1·13 (0·34–2·44)	1·59 (0·413–3·52)	1·08 (0·332–2·3)
Yemen	All causes	4 350 (2 870–5 730)	3 220 (1 060–4 750)	2 250 (1 600–3 050)	2 090 (1 420–2 820)
Yemen	Diarrheal diseases	607 (290–1 170)	356 (156–665)	428 (-27·9–1 050)	245 (107–455)
Yemen	Lower respiratory infections	2 690 (1 670–3 950)	2 010 (201–3 470)	1 170 (656–1 800)	1 550 (924–2 280)
Yemen	Malaria	152 (-137–653)	118 (-89·1–506)	--	70·3 (-33·4–302)
Yemen	Measles	405 (139–797)	243 (85·7–507)	155 (47·0–347)	225 (73·0–469)
**South Asia**	**All causes**	**165 000 (134 000–194 000)**	**113 000 (49 000–155 000)**	**98 900 (71 200–120 000)**	**69 600 (49 700–91 300)**
**South Asia**	**Diarrheal diseases**	**44 200 (30 000–61 900)**	**23 800 (14 900–36 600)**	**32 600 (-2 800–57 400)**	**15 400 (9 690–23 100)**
**South Asia**	**Lower respiratory infections**	**98 600 (77 200–120 000)**	**70 800 (5 950–108 000)**	**49 600 (33 800–68 300)**	**49 900 (32 100–68 600)**
**South Asia**	**Malaria**	**1 990 (-1 620–6 700)**	**1 540 (-1 090–5 020)**	**--**	**864 (-421–4 070)**
**South Asia**	**Measles**	**6 810 (2 620–14 300)**	**3 810 (1 350–8 140)**	**3 220 (956–7 040)**	**3 450 (1 250–7 410)**
South Asia	All causes	165 000 (134 000–194 000)	113 000 (49 000–155 000)	98 900 (71 200–120 000)	69 600 (49 700–91 300)
South Asia	Diarrheal diseases	44 200 (30 000–61 900)	23 800 (14 900–36 600)	32 600 (-2 800–57 400)	15 400 (9 690–23 100)

South Asia	Lower respiratory infections	98 600 (77 200–120 000)	70 800 (5 950–108 000)	49 600 (33 800–68 300)	49 900 (32 100–68 600)
South Asia	Malaria	1 990 (-1 620–6 700)	1 540 (-1 090–5 020)	--	864 (-421–4 070)
South Asia	Measles	6 810 (2 620–14 300)	3 810 (1 350–8 140)	3 220 (956–7 040)	3 450 (1 250–7 410)
Bangladesh	All causes	10 500 (8 010–13 100)	7 740 (3 930–10 700)	6 430 (5 040–8 100)	3 250 (2 120–4 690)
Bangladesh	Diarrheal diseases	1 610 (814–2 710)	761 (402–1 260)	1 140 (-54·4–2 520)	481 (272–769)
Bangladesh	Lower respiratory infections	5 490 (3 670–7 440)	3 820 (234–6 620)	2 200 (1 220–3 490)	2 570 (1 580–3 810)
Bangladesh	Malaria	0·0487 (-0·026–0·204)	0·0394 (-0·0212–0·157)	--	0·0164 (-0·006–0·0845)
Bangladesh	Measles	427 (161–909)	225 (78·2–478)	165 (48·2–356)	205 (68·2–465)
Bhutan	All causes	31·2 (19·4–45·5)	15·8 (6·32–27·2)	14·6 (6·42–27·0)	12·3 (8·36–17·7)
Bhutan	Diarrheal diseases	13·0 (5·44–26·9)	4·27 (1·64–9·21)	8·8 (-0·284–23·6)	3·8 (1·62–7·36)
Bhutan	Lower respiratory infections	17·6 (11·1–27·5)	10·8 (0·623–24·7)	5·15 (2·55–8·37)	8·47 (5·09–13·0)
Bhutan	Malaria	0·00106 (>-0.001–0·00506)	<0.001 (>-0.001–0·0035)	--	<0.001 (>-0.001–0·00257)
Bhutan	Measles	0·0145 (0·00346–0·0314)	0·00553 (0·0013–0·0123)	0·00404 (<0.001–0·00905)	0·00802 (0·00187–0·0177)
India	All causes	112 000 (88 300–131 000)	78 200 (34 600–107 000)	68 100 (50 800–83 000)	50 300 (35 800–68 300)
India	Diarrheal diseases	28 300 (18 700–37 700)	16 000 (9 610–24 000)	21 100 (-1 990–36 000)	10 500 (6 350–15 100)
India	Lower respiratory infections	70 300 (54 700–86 100)	51 000 (4 340–77 500)	37 500 (25 200–53 200)	36 400 (23 000–51 400)
India	Malaria	1 410 (-992–4 190)	1 110 (-699–3 200)	--	584 (-295–2 660)
India	Measles	5 650 (2 180–11 900)	3 200 (1 150–6 860)	2 750 (828–6 110)	2 870 (1 050–6 280)
Nepal	All causes	2 120 (1 600–2 710)	1 480 (766–2 180)	1 170 (908–1 450)	761 (527–1 060)
Nepal	Diarrheal diseases	253 (118–476)	118 (51·2–237)	179 (-9·3–394)	76·1 (32·2–154)
Nepal	Lower respiratory infections	1 160 (747–1 690)	806 (55·0–1 540)	471 (264–726)	540 (329–822)
Nepal	Malaria	0·0164 (-0·0104–0·0775)	0·0127 (-0·0074–0·0583)	--	0·00647 (-0·00275–0·0325)
Nepal	Measles	288 (108–528)	148 (54·3–293)	107 (33·3–217)	144 (51·3–274)
Pakistan	All causes	40 000 (26 900–52 800)	26 000 (9 900–36 900)	23 200 (12 800–32 300)	15 200 (10 500–21 800)
Pakistan	Diarrheal diseases	14 100 (6 900–23 900)	6 930 (3 500–12 100)	10 200 (-750–21 100)	4 360 (2 330–7 560)
Pakistan	Lower respiratory infections	21 600 (13 800–31 600)	15 200 (1 320–27 400)	9 490 (5 310–15 200)	10 300 (6 570–16 300)
Pakistan	Malaria	577 (-483–2 740)	436 (-315–2 010)	--	280 (-155–1 440)
Pakistan	Measles	451 (141–1 000)	235 (71·9–507)	191 (54·0–409)	232 (73·5–537)
**Southeast Asia, East Asia, and Oceania**	**All causes**	**40 400 (30 300–50 500)**	**25 300 (10 200–37 400)**	**19 400 (13 800–25 400)**	**17 000 (12 400–22 400)**

**Southeast Asia, East Asia, and Oceania**	**Diarrheal diseases**	**10 100 (5 410–16 800)**	**4 480 (2 060–8 360)**	**6 800 (-339–14 900)**	**3 520 (1 690–6 200)**
**Southeast Asia, East Asia, and Oceania**	**Lower respiratory infections**	**24 000 (16 900–31 500)**	**16 000 (1 100–28 900)**	**8 260 (4 920–12 100)**	**11 900 (7 940–16 600)**
**Southeast Asia, East Asia, and Oceania**	**Malaria**	**128 (-82·9–477)**	**90·6 (-51–317)**	**--**	**61·9 (-30·7–299)**
**Southeast Asia, East Asia, and Oceania**	**Measles**	**2 790 (1 040–5 520)**	**1 350 (474–2 790)**	**926 (277–1 950)**	**1 510 (562–3 100)**
East Asia	All causes	3 180 (2 100–4 360)	1 970 (369–3 790)	1 020 (753–1 350)	1 270 (905–1 770)
East Asia	Diarrheal diseases	201 (113–302)	64·7 (34·6–103)	133 (-4·17–290)	52·7 (32·1–76·9)
East Asia	Lower respiratory infections	2 810 (1 800–3 930)	1 730 (87·3–3 580)	715 (408–1 080)	1 220 (870–1 710)
East Asia	Malaria	<0.001 (>-0.001–<0.001)	<0.001 (>-0.001–<0.001)	--	<0.001 (>-0.001–<0.001)
East Asia	Measles	0·185 (0·0921–0·251)	0·0709 (0·0351–0·105)	0·0484 (0·0181–0·0986)	0·0932 (0·0425–0·137)
China	All causes	2 720 (1 790–3 830)	1 630 (296–3 280)	879 (647–1 170)	1 050 (749–1 460)
China	Diarrheal diseases	178 (97·9–272)	52·9 (27·7–84·7)	119 (-3·42–259)	44·6 (27·2–65·1)
China	Lower respiratory infections	2 380 (1 550–3 480)	1 420 (50·8–3 070)	604 (324–943)	1 010 (715–1 410)
China	Malaria	0 (0–0)	0 (0–0)	--	0 (0–0)
China	Measles	0·179 (0·0907–0·246)	0·0687 (0·034–0·102)	0·046 (0·0175–0·094)	0·0913 (0·0415–0·136)
Democratic People's Republic of Korea	All causes	442 (268–651)	327 (61·9–580)	134 (86·0–199)	216 (118–336)
Democratic People's Republic of Korea	Diarrheal diseases	22·2 (9·75–43·7)	11·5 (4·46–25·1)	13·0 (-0·719–34·3)	7·94 (3·14–17·8)
Democratic People's Republic of Korea	Lower respiratory infections	405 (226–610)	301 (36·1–556)	106 (55·8–174)	208 (112–327)
Democratic People's Republic of Korea	Malaria	<0.001 (>-0.001–<0.001)	<0.001 (>-0.001–<0.001)	--	<0.001 (>-0.001–<0.001)
Democratic People's Republic of Korea	Measles	0 (0–0)	0 (0–0)	0 (0–0)	0 (0–0)
Taiwan	All causes	24·6 (15·8–38·3)	16·4 (4·65–33·2)	9·75 (7·38–12·5)	5·66 (3·97–8·06)
Taiwan	Diarrheal diseases	1·24 (0·506–2·27)	0·304 (0·149–0·485)	0·922 (-0·0297–2·19)	0·168 (0·093–0·241)
Taiwan	Lower respiratory infections	19·5 (11·6–32·5)	12·3 (0·414–29·2)	5·03 (2·75–7·63)	5·49 (3·84–7·85)
Taiwan	Malaria	0 (0–0)	0 (0–0)	--	0 (0–0)
Taiwan	Measles	0·00599 (0·00167–0·0127)	0·00213 (<0.001–0·00473)	0·00245 (<0.001–0·0061)	0·00193 (<0.001–0·00413)
Oceania	All causes	3 600 (2 640–4 540)	2 380 (1 050–3 380)	1 790 (1 380–2 230)	1 740 (1 180–2 270)
Oceania	Diarrheal diseases	484 (225–862)	231 (101–443)	323 (-17·1–742)	207 (99·6–369)
Oceania	Lower respiratory infections	1 850 (1 180–2 560)	1 250 (84·6–2 250)	711 (419–1 100)	1 080 (673–1 560)
Oceania	Malaria	81·7 (-55·1–321)	54·3 (-29·7–203)	--	45·9 (-23·5–199)
Oceania	Measles	671 (258–1 230)	325 (110–628)	238 (64·9–510)	407 (142–761)

American Samoa	All causes	0·808 (0·547–1·19)	0·521 (0·242–0·934)	0·395 (0·304–0·508)	0·207 (0·136–0·294)
American Samoa	Diarrheal diseases	0·0706 (0·0281–0·147)	0·0198 (0·008–0·0415)	0·0501 (-0·00179–0·137)	0·0135 (0·00632–0·026)
American Samoa	Lower respiratory infections	0·437 (0·246–0·74)	0·272 (0·00996–0·663)	0·123 (0·0643–0·205)	0·144 (0·0867–0·235)
American Samoa	Malaria	0 (0–0)	0 (0–0)	--	0 (0–0)
American Samoa	Measles	0·115 (0·0406–0·221)	0·0437 (0·0152–0·0906)	0·0366 (0·0106–0·088)	0·0494 (0·0153–0·104)
Cook Islands	All causes	0·141 (0·0819–0·246)	0·0813 (0·00689–0·214)	0·0478 (0·0299–0·0729)	0·036 (0·0217–0·0561)
Cook Islands	Diarrheal diseases	0·00244 (<0.001–0·00556)	<0.001 (<0.001–0·00113)	0·00189 (>-0.001–0·00538)	<0.001 (<0.001–<0.001)
Cook Islands	Lower respiratory infections	0·132 (0·0743–0·237)	0·0774 (0·00248–0·209)	0·0423 (0·0224–0·068)	0·0339 (0·0198–0·0546)
Cook Islands	Malaria	0 (0–0)	0 (0–0)	--	0 (0–0)
Cook Islands	Measles	0·00468 (0·00136–0·00975)	0·00155 (<0.001–0·00349)	0·00176 (<0.001–0·00421)	0·00175 (<0.001–0·00366)
Fiji	All causes	37·1 (23·7–53·4)	23·4 (7·1–42·6)	18·5 (13·9–23·3)	7·17 (4·85–10·5)
Fiji	Diarrheal diseases	4·42 (1·56–8·45)	1·28 (0·541–2·45)	3·39 (-0·117–8·14)	0·529 (0·256–0·915)
Fiji	Lower respiratory infections	26·1 (14·5–39·9)	16·7 (0·656–35·2)	9·66 (5·51–14·7)	6·02 (3·83–9·23)
Fiji	Malaria	0 (0–0)	0 (0–0)	--	0 (0–0)
Fiji	Measles	2·05 (0·663–4·31)	0·801 (0·256–1·79)	0·894 (0·233–2·1)	0·618 (0·182–1·33)
Guam	All causes	2·58 (1·58–4·08)	1·49 (0·261–3·42)	1·04 (0·743–1·36)	0·611 (0·373–0·924)
Guam	Diarrheal diseases	0·285 (0·0995–0·494)	0·0635 (0·0289–0·108)	0·214 (-0·00565–0·477)	0·042 (0·0221–0·0665)
Guam	Lower respiratory infections	2·11 (1·27–3·54)	1·25 (0·0334–3·2)	0·649 (0·356–0·969)	0·568 (0·343–0·864)
Guam	Malaria	0 (0–0)	0 (0–0)	--	0 (0–0)
Guam	Measles	<0.001 (<0.001–<0.001)	<0.001 (<0.001–<0.001)	<0.001 (<0.001–<0.001)	<0.001 (<0.001–<0.001)
Kiribati	All causes	22·5 (16·0–29·2)	16·5 (9·54–23·3)	14·1 (9·98–18·1)	4·88 (3·14–6·95)
Kiribati	Diarrheal diseases	3·97 (1·91–6·71)	1·54 (0·586–2·82)	2·62 (-0·105–5·85)	0·947 (0·392–1·63)
Kiribati	Lower respiratory infections	8·86 (5·57–13·4)	5·9 (0·293–12·1)	2·68 (1·48–4·39)	3·39 (2·12–5·16)
Kiribati	Malaria	0 (0–0)	0 (0–0)	--	0 (0–0)
Kiribati	Measles	1·18 (0·392–2·56)	0·551 (0·176–1·21)	0·322 (0·0892–0·749)	0·541 (0·176–1·25)
Marshall Islands	All causes	2·43 (1·38–3·61)	1·88 (0·465–3·29)	0·417 (0·308–0·58)	0·816 (0·511–1·22)
Marshall Islands	Diarrheal diseases	0·204 (0·0919–0·392)	0·123 (0·0503–0·246)	0·0608 (-0·00145–0·202)	0·0582 (0·0237–0·113)
Marshall Islands	Lower respiratory infections	1·9 (0·895–3·08)	1·46 (0·0749–2·92)	0·127 (0·0625–0·211)	0·709 (0·422–1·11)
Marshall Islands	Malaria	0 (0–0)	0 (0–0)	--	0 (0–0)

Marshall Islands	Measles	0·108 (0·035–0·224)	0·0669 (0·021–0·147)	0·00467 (0·00131–0·0106)	0·0492 (0·0145–0·107)
Micronesia (Federated States of)	All causes	3·26 (2·24–4·26)	2·28 (1·28–3·3)	1·91 (1·29–2·58)	0·807 (0·514–1·13)
Micronesia (Federated States of)	Diarrheal diseases	0·53 (0·238–1)	0·187 (0·0725–0·373)	0·348 (-0·0131–0·96)	0·131 (0·057–0·246)
Micronesia (Federated States of)	Lower respiratory infections	1·28 (0·685–1·96)	0·82 (0·0454–1·76)	0·343 (0·168–0·583)	0·523 (0·319–0·808)
Micronesia (Federated States of)	Malaria	0 (0–0)	0 (0–0)	--	0 (0–0)
Micronesia (Federated States of)	Measles	0·327 (0·107–0·682)	0·139 (0·0461–0·301)	0·0923 (0·0245–0·213)	0·153 (0·046–0·331)
Nauru	All causes	1·34 (0·887–1·97)	0·842 (0·21–1·61)	0·384 (0·303–0·483)	0·553 (0·361–0·813)
Nauru	Diarrheal diseases	0·106 (0·0411–0·22)	0·0343 (0·012–0·0752)	0·0547 (-0·00169–0·161)	0·0377 (0·0158–0·0756)
Nauru	Lower respiratory infections	1·05 (0·618–1·67)	0·637 (0·0261–1·46)	0·161 (0·0811–0·267)	0·507 (0·324–0·764)
Nauru	Malaria	0 (0–0)	0 (0–0)	--	0 (0–0)
Nauru	Measles	0·0122 (0·00374–0·0272)	0·00417 (0·00123–0·0101)	0·00137 (<0.001–0·00327)	0·00798 (0·00236–0·0177)
Niue	All causes	0·0267 (0·0165–0·04)	0·017 (0·00425–0·0339)	0·0107 (0·00816–0·0141)	0·00749 (0·00485–0·0114)
Niue	Diarrheal diseases	0·00165 (<0.001–0·00398)	<0.001 (<0.001–0·00105)	0·00117 (>-0.001–0·00352)	<0.001 (<0.001–<0.001)
Niue	Lower respiratory infections	0·0213 (0·0121–0·0342)	0·013 (<0.001–0·0304)	0·00601 (0·00319–0·01)	0·00704 (0·00445–0·011)
Niue	Malaria	0 (0–0)	0 (0–0)	--	0 (0–0)
Niue	Measles	<0.001 (<0.001–<0.001)	<0.001 (<0.001–<0.001)	<0.001 (<0.001–<0.001)	<0.001 (<0.001–<0.001)
Northern Mariana Islands	All causes	0·382 (0·224–0·623)	0·219 (0·0795–0·45)	0·174 (0·126–0·246)	0·0966 (0·0674–0·138)
Northern Mariana Islands	Diarrheal diseases	0·0582 (0·0196–0·129)	0·0135 (0·00539–0·0298)	0·0431 (-0·00162–0·124)	0·00935 (0·00432–0·0184)
Northern Mariana Islands	Lower respiratory infections	0·248 (0·13–0·448)	0·146 (0·00535–0·383)	0·071 (0·0356–0·117)	0·0775 (0·0496–0·118)
Northern Mariana Islands	Malaria	0 (0–0)	0 (0–0)	--	0 (0–0)
Northern Mariana Islands	Measles	0·0239 (0·00768–0·0485)	0·00822 (0·00268–0·0173)	0·00821 (0·00228–0·0202)	0·00971 (0·00312–0·0203)
Palau	All causes	0·374 (0·225–0·593)	0·216 (0·068–0·448)	0·175 (0·119–0·252)	0·0894 (0·0546–0·144)
Palau	Diarrheal diseases	0·0766 (0·0234–0·16)	0·0201 (0·0077–0·043)	0·0561 (-0·00201–0·153)	0·0127 (0·00542–0·025)
Palau	Lower respiratory infections	0·243 (0·135–0·419)	0·15 (0·00586–0·387)	0·0732 (0·0358–0·123)	0·0716 (0·0386–0·12)
Palau	Malaria	0 (0–0)	0 (0–0)	--	0 (0–0)
Palau	Measles	0·0125 (0·00415–0·0266)	0·00465 (0·00155–0·0103)	0·00419 (0·00116–0·0102)	0·00506 (0·00164–0·0115)
Papua New Guinea	All causes	3 310 (2 430–4 150)	2 190 (968–3 120)	1 640 (1 250–2 060)	1 620 (1 100–2 110)
Papua New Guinea	Diarrheal diseases	443 (201–796)	214 (92·9–415)	295 (-15·7–681)	193 (93·7–346)
Papua New Guinea	Lower respiratory infections	1 690 (1 070–2 340)	1 150 (78·5–2 060)	654 (386–1 020)	1 010 (617–1 470)

Papua New Guinea	Malaria	78·2 (-52·8–307)	51·9 (-28·4–194)	--	43·9 (-22·5–190)
Papua New Guinea	Measles	626 (240–1 140)	304 (103–586)	223 (60·6–478)	382 (133–715)
Samoa	All causes	10·7 (6·45–15·9)	6·19 (1·51–13·0)	5 (3·45–6·88)	2·73 (1·62–4·3)
Samoa	Diarrheal diseases	0·815 (0·256–1·85)	0·192 (0·0681–0·43)	0·626 (-0·0261–1·73)	0·127 (0·0512–0·267)
Samoa	Lower respiratory infections	8·52 (4·93–13·6)	4·86 (0·205–11·7)	3·21 (1·66–5·23)	2·45 (1·42–4·07)
Samoa	Malaria	0 (0–0)	0 (0–0)	--	0 (0–0)
Samoa	Measles	0·418 (0·114–0·906)	0·143 (0·0396–0·343)	0·168 (0·0375–0·416)	0·155 (0·0396–0·345)
Solomon Islands	All causes	29·0 (20·1–37·1)	18·1 (9·41–27·0)	14·2 (9·91–18·0)	11·4 (7·63–15·9)
Solomon Islands	Diarrheal diseases	3·2 (1·4–6·01)	1·27 (0·518–2·55)	2·19 (-0·095–5·56)	1·03 (0·477–1·97)
Solomon Islands	Lower respiratory infections	13·4 (8·25–19·6)	8·73 (0·564–18·0)	4·63 (2·56–7·35)	6·32 (3·4–9·86)
Solomon Islands	Malaria	0·129 (-0·0784–0·528)	0·0886 (-0·0481–0·342)	--	0·0614 (-0·0231–0·304)
Solomon Islands	Measles	7·63 (3·07–13·8)	3·38 (1·25–6·45)	2·69 (0·79–5·7)	4·04 (1·37–7·48)
Tokelau	All causes	0·0248 (0·0149–0·0367)	0·0156 (0·00315–0·0307)	0·00964 (0·00714–0·0133)	0·00727 (0·00454–0·0111)
Tokelau	Diarrheal diseases	0·00177 (<0.001–0·00397)	<0.001 (<0.001–0·0012)	0·00124 (>-0.001–0·0034)	<0.001 (<0.001–<0.001)
Tokelau	Lower respiratory infections	0·0201 (0·0113–0·0309)	0·0124 (<0.001–0·0278)	0·00569 (0·00304–0·00929)	0·00678 (0·00414–0·0108)
Tokelau	Malaria	0 (0–0)	0 (0–0)	--	0 (0–0)
Tokelau	Measles	<0.001 (<0.001–<0.001)	<0.001 (<0.001–<0.001)	<0.001 (<0.001–<0.001)	<0.001 (<0.001–<0.001)
Tonga	All causes	1·68 (1·04–2·81)	1·08 (0·345–2·44)	0·802 (0·562–1·08)	0·322 (0·21–0·507)
Tonga	Diarrheal diseases	0·0459 (0·0107–0·114)	0·00785 (0·00303–0·0162)	0·0367 (>-0.001–0·107)	0·00534 (0·00223–0·0105)
Tonga	Lower respiratory infections	1·26 (0·665–2·39)	0·716 (0·0199–2·07)	0·404 (0·205–0·663)	0·311 (0·2–0·497)
Tonga	Malaria	0 (0–0)	0 (0–0)	--	0 (0–0)
Tonga	Measles	0·0166 (0·00438–0·0384)	0·00475 (0·0013–0·0114)	0·00764 (0·00176–0·0205)	0·00529 (0·0015–0·0127)
Tuvalu	All causes	0·73 (0·472–1·09)	0·421 (0·123–0·838)	0·283 (0·213–0·387)	0·258 (0·155–0·386)
Tuvalu	Diarrheal diseases	0·0944 (0·0325–0·215)	0·0246 (0·00937–0·0557)	0·0645 (-0·00294–0·201)	0·0239 (0·0108–0·0486)
Tuvalu	Lower respiratory infections	0·525 (0·297–0·836)	0·306 (0·0132–0·739)	0·129 (0·0663–0·211)	0·219 (0·122–0·346)
Tuvalu	Malaria	0 (0–0)	0 (0–0)	--	0 (0–0)
Tuvalu	Measles	0·0298 (0·00991–0·0638)	0·01 (0·00328–0·0216)	0·00886 (0·00241–0·0211)	0·0146 (0·00467–0·0325)
Vanuatu	All causes	29·1 (20·4–37·0)	16·3 (6·81–25·6)	11·8 (7·92–16·2)	11·6 (8·13–15·4)
Vanuatu	Diarrheal diseases	6·28 (3·18–11·4)	2·3 (0·97–4·29)	4·14 (-0·159–10·1)	1·86 (0·868–3·09)

Vanuatu	Lower respiratory infections	16·5 (10·3–23·2)	10·6 (0·595–20·9)	4·84 (2·65–7·29)	7·16 (4·08–10·6)
Vanuatu	Malaria	<0.001 (>-0.001–<0.001)	<0.001 (>-0.001–<0.001)	--	<0.001 (>-0.001–<0.001)
Vanuatu	Measles	4·95 (2·03–9)	2·06 (0·805–4·06)	1·44 (0·459–3·07)	2·6 (0·94–4·77)
Southeast Asia	All causes	33 600 (24 700–42 100)	21 000 (8 840–30 600)	16 600 (11 200–22 600)	14 000 (9 860–18 800)
Southeast Asia	Diarrheal diseases	9 390 (4 940–15 500)	4 190 (1 890–7 880)	6 350 (-315–14 000)	3 260 (1 540–5 790)
Southeast Asia	Lower respiratory infections	19 300 (13 700–26 100)	13 000 (929–24 100)	6 830 (3 950–10 200)	9 620 (6 130–13 800)
Southeast Asia	Malaria	46·2 (-27·7–163)	36·4 (-19·8–121)	--	16·0 (-6·84–74·5)
Southeast Asia	Measles	2 120 (751–4 350)	1 020 (364–2 180)	688 (206–1 520)	1 100 (393–2 390)
Cambodia	All causes	1 280 (861–1 700)	847 (175–1 420)	586 (426–790)	569 (354–856)
Cambodia	Diarrheal diseases	172 (71·3–337)	73·9 (29·6–154)	121 (-6·51–295)	55·3 (23·6–111)
Cambodia	Lower respiratory infections	1 030 (684–1 470)	697 (48·1–1 290)	390 (218–619)	513 (312–770)
Cambodia	Malaria	1·59 (-1·01–7·14)	1·21 (-0·735–5·12)	--	0·644 (-0·208–3·27)
Cambodia	Measles	0·0204 (0·00828–0·0363)	0·0101 (0·00415–0·0191)	0·00719 (0·00245–0·0166)	0·0108 (0·0039–0·0191)
Indonesia	All causes	15 300 (11 000–20 200)	9 550 (4 900–13 900)	7 990 (4 620–11 700)	6 770 (4 700–9 710)
Indonesia	Diarrheal diseases	5 390 (2 810–9 100)	2 490 (1 070–4 860)	3 580 (-192–8 040)	2 090 (962–3 780)
Indonesia	Lower respiratory infections	7 640 (5 160–10 800)	5 200 (445–9 630)	2 680 (1 440–4 230)	4 220 (2 590–6 400)
Indonesia	Malaria	8·67 (-4·99–30·4)	6·47 (-3·7–22·8)	--	3·68 (-1·41–17·6)
Indonesia	Measles	849 (310–1 790)	414 (142–944)	289 (80·6–643)	461 (159–1 010)
Lao People's Democratic Republic	All causes	1 420 (935–1 890)	898 (275–1 420)	668 (457–910)	569 (383–783)
Lao People's Democratic Republic	Diarrheal diseases	272 (117–518)	112 (45·8–215)	189 (-8·33–452)	81·9 (35·5–157)
Lao People's Democratic Republic	Lower respiratory infections	959 (591–1 370)	639 (41·3–1 220)	348 (190–556)	439 (257–664)
Lao People's Democratic Republic	Malaria	0·482 (-0·267–2·37)	0·361 (-0·184–1·75)	--	0·193 (-0·0647–1·07)
Lao People's Democratic Republic	Measles	88·7 (32·2–179)	43·9 (14·9–93·6)	28·6 (8·29–59·9)	47·1 (15·6–95·3)
Malaysia	All causes	212 (150–300)	130 (38·7–230)	97·9 (75·4–129)	82·0 (60·0–117)
Malaysia	Diarrheal diseases	35·8 (17·4–61·4)	13·3 (7·11–21·3)	26·1 (-0·846–55·1)	8·41 (4·8–12·8)
Malaysia	Lower respiratory infections	150 (91·5–224)	97·3 (5·24–199)	52·8 (28·3–86·8)	68·4 (48·7–99·4)
Malaysia	Malaria	0·0313 (-0·0158–0·108)	0·0246 (-0·0121–0·0774)	--	0·00994 (-0·00383–0·0496)
Malaysia	Measles	12·3 (3·74–25·7)	5·44 (1·63–12·3)	5·04 (1·2–11·9)	5·19 (1·46–11·1)
Maldives	All causes	6·03 (4·33–7·56)	3·84 (1·76–5·5)	3·17 (1·88–4·28)	2·21 (1·52–3·13)

Maldives	Diarrheal diseases	2·09 (1·22–3·26)	0·932 (0·459–1·73)	1·4 (-0·059–2·93)	0·648 (0·33–1·12)
Maldives	Lower respiratory infections	3·26 (2·16–4·53)	2·23 (0·156–4·09)	1·09 (0·584–1·72)	1·56 (0·968–2·33)
Maldives	Malaria	0 (0–0)	0 (0–0)	--	0 (0–0)
Maldives	Measles	0·0041 (<0.001–0·00766)	0·00195 (<0.001–0·00375)	0·00149 (<0.001–0·0032)	0·00181 (<0.001–0·00346)
Mauritius	All causes	9·59 (6·84–11·9)	5·29 (2·11–7·98)	5·89 (3·81–7·06)	2·5 (1·74–3·48)
Mauritius	Diarrheal diseases	3·14 (1·52–4·26)	1 (0·558–1·56)	2·48 (-0·122–4·15)	0·583 (0·358–0·859)
Mauritius	Lower respiratory infections	5·64 (4·3–7·1)	3·49 (0·164–6·53)	2·61 (1·76–3·59)	1·92 (1·27–2·71)
Mauritius	Malaria	0 (0–0)	0 (0–0)	--	0 (0–0)
Mauritius	Measles	<0.001 (<0.001–<0.001)	<0.001 (<0.001–<0.001)	<0.001 (<0.001–<0.001)	<0.001 (<0.001–<0.001)
Myanmar	All causes	5 680 (3 830–7 780)	3 430 (1 090–5 750)	2 500 (1 760–3 640)	2 170 (1 380–3 170)
Myanmar	Diarrheal diseases	1 210 (505–2 540)	489 (184–1 010)	838 (-34·4–2 120)	330 (130–644)
Myanmar	Lower respiratory infections	3 640 (2 330–5 290)	2 430 (158–4 860)	1 290 (712–2 050)	1 520 (853–2 520)
Myanmar	Malaria	34·8 (-20·6–119)	27·8 (-16·3–92·5)	--	11·2 (-4·93–51·1)
Myanmar	Measles	606 (219–1 140)	297 (105–611)	185 (57·3–389)	308 (108–625)
Philippines	All causes	7 680 (5 620–9 540)	4 920 (1 950–6 930)	3 880 (2 580–5 130)	2 970 (2 120–3 970)
Philippines	Diarrheal diseases	2 100 (1 200–3 180)	932 (488–1 540)	1 440 (-65·3–3 010)	640 (364–1 010)
Philippines	Lower respiratory infections	4 550 (3 260–5 850)	3 100 (182–5 290)	1 590 (946–2 330)	2 190 (1 470–3 040)
Philippines	Malaria	0·495 (-0·33–2·4)	0·381 (-0·266–1·81)	--	0·198 (-0·0836–1·04)
Philippines	Measles	273 (98·1–556)	137 (46·2–298)	89·5 (24·3–206)	138 (45·4–303)
Seychelles	All causes	1·53 (0·966–2·16)	0·902 (0·218–1·69)	0·661 (0·496–0·825)	0·484 (0·346–0·694)
Seychelles	Diarrheal diseases	0·224 (0·089–0·396)	0·0604 (0·0273–0·114)	0·165 (-0·00526–0·36)	0·0435 (0·0228–0·0721)
Seychelles	Lower respiratory infections	1·16 (0·719–1·72)	0·708 (0·0328–1·54)	0·364 (0·213–0·545)	0·43 (0·299–0·628)
Seychelles	Malaria	0 (0–0)	0 (0–0)	--	0 (0–0)
Seychelles	Measles	0·0249 (0·00775–0·0529)	0·00951 (0·00299–0·0211)	0·00853 (0·0022–0·0205)	0·0105 (0·00333–0·0237)
Sri Lanka	All causes	95·9 (67·6–121)	63·0 (21·9–90·7)	52·0 (37·5–65·7)	34·4 (23·4–46·4)
Sri Lanka	Diarrheal diseases	24·8 (12·9–38·2)	11·4 (6·39–19·1)	18·1 (-0·858–35·0)	6·4 (3·69–10·2)
Sri Lanka	Lower respiratory infections	63·4 (44·8–80·9)	44·2 (3·21–74·3)	26·5 (15·7–39·0)	27·9 (17·3–39·0)
Sri Lanka	Malaria	0 (0–0)	0 (0–0)	--	0 (0–0)
Sri Lanka	Measles	0·338 (0·187–0·453)	0·173 (0·088–0·247)	0·151 (0·0609–0·268)	0·132 (0·0628–0·194)

Thailand	All causes	391 (296–493)	220 (92·8–330)	179 (124–232)	151 (109–199)
Thailand	Diarrheal diseases	89·8 (50·3–144)	31·8 (16·2–50·5)	62·1 (-1·88–133)	25·0 (14·6–37·0)
Thailand	Lower respiratory infections	227 (163–302)	142 (7·22–264)	71·9 (40·8–110)	105 (72·6–144)
Thailand	Malaria	0·00347 (-0·00179–0·0144)	0·00257 (-0·00127–0·0108)	--	0·00124 (>-0.001–0·00634)
Thailand	Measles	45·2 (15·8–90·4)	17·2 (5·81–37·0)	15·7 (4·42–34·8)	21·2 (7·01–45·1)
Timor-Leste	All causes	318 (232–399)	233 (71·4–351)	183 (134–243)	148 (99·3–207)
Timor-Leste	Diarrheal diseases	33·4 (14·6–63·6)	17·7 (8–35·2)	24·5 (-1·63–55·9)	13·0 (5·83–25·0)
Timor-Leste	Lower respiratory infections	239 (160–322)	170 (13·3–299)	113 (68·4–176)	135 (89·2–197)
Timor-Leste	Malaria	<0.001 (>-0.001–0·00268)	<0.001 (>-0.001–0·00185)	--	<0.001 (>-0.001–0·0016)
Timor-Leste	Measles	0·349 (0·195–0·51)	0·198 (0·0973–0·304)	0·167 (0·0684–0·296)	0·187 (0·0838–0·28)
Viet Nam	All causes	1 100 (698–1 530)	640 (163–1 120)	378 (223–581)	514 (317–796)
Viet Nam	Diarrheal diseases	40·0 (13·5–79·1)	14·4 (5·5–30·7)	27·5 (-0·982–70·0)	11·4 (4·43–22·6)
Viet Nam	Lower respiratory infections	801 (461–1 150)	503 (24·1–986)	260 (126–480)	381 (221–645)
Viet Nam	Malaria	0·0853 (-0·0453–0·456)	0·0626 (-0·0321–0·32)	--	0·0342 (-0·0126–0·184)
Viet Nam	Measles	239 (83·7–478)	105 (34·7–219)	74·4 (21·1–166)	121 (40·0–250)
**Sub-Saharan Africa**	**All causes**	**618 000 (299 000–862 000)**	**399 000 (149 000–594 000)**	**283 000 (160 000–412 000)**	**264 000 (165 000–386 000)**
**Sub-Saharan Africa**	**Diarrheal diseases**	**178 000 (104 000–274 000)**	**85 100 (45 200–143 000)**	**123 000 (-8 480–243 000)**	**62 200 (33 400–99 100)**
**Sub-Saharan Africa**	**Lower respiratory infections**	**205 000 (126 000–288 000)**	**141 000 (11 000–253 000)**	**71 800 (41 300–115 000)**	**117 000 (75 700–163 000)**
**Sub-Saharan Africa**	**Malaria**	**105 000 (-69 000–333 000)**	**75 400 (-43 300–223 000)**	**--**	**49 700 (-26 200–219 000)**
**Sub-Saharan Africa**	**Measles**	**65 800 (25 100–127 000)**	**33 700 (12 400–64 700)**	**24 000 (7 110–50 600)**	**35 200 (12 200–69 000)**
Central Sub-Saharan Africa	All causes	76 400 (31 400–121 000)	48 100 (11 000–79 500)	26 300 (16 300–39 600)	36 700 (19 500–62 000)
Central Sub-Saharan Africa	Diarrheal diseases	14 600 (7 300–25 700)	6 620 (3 130–12 400)	9 630 (-661–21 800)	5 280 (2 530–9 510)
Central Sub-Saharan Africa	Lower respiratory infections	26 200 (16 200–37 700)	17 800 (1 190–33 100)	8 420 (4 560–14 000)	15 000 (9 810–22 000)
Central Sub-Saharan Africa	Malaria	20 000 (-12 700–57 500)	13 800 (-7 420–38 000)	--	10 000 (-4 790–40 000)
Central Sub-Saharan Africa	Measles	11 100 (4 050–22 400)	5 380 (1 910–11 000)	3 660 (1 010–8 240)	6 360 (2 100–12 700)
Angola	All causes	14 200 (7 100–20 500)	9 070 (3 180–13 600)	5 330 (2 910–8 350)	6 550 (4 110–9 160)
Angola	Diarrheal diseases	3 620 (1 730–6 280)	1 710 (736–3 280)	2 240 (-123–5 420)	1 390 (621–2 600)
Angola	Lower respiratory infections	5 670 (3 250–8 150)	3 870 (298–7 080)	1 520 (791–2 590)	3 300 (2 060–4 730)
Angola	Malaria	2 240 (-1 320–7 100)	1 590 (-891–4 790)	--	1 040 (-483–4 630)

Angola	Measles	1 420 (498–2 940)	698 (240–1 480)	371 (102–867)	831 (294–1 880)
Central African Republic	All causes	9 700 (5 440–13 000)	5 600 (2 770–7 640)	4 770 (1 530–7 960)	4 280 (2 930–5 270)
Central African Republic	Diarrheal diseases	5 190 (3 000–7 550)	2 410 (1 310–4 020)	3 420 (-178–6 910)	1 910 (988–2 980)
Central African Republic	Lower respiratory infections	3 050 (1 770–4 600)	2 090 (173–4 090)	954 (472–1 530)	1 800 (1 150–2 570)
Central African Republic	Malaria	961 (-619–2 770)	670 (-369–1 910)	--	488 (-237–2 020)
Central African Republic	Measles	138 (39·5–326)	70·6 (20·4–168)	42·4 (9·81–112)	80·0 (21·0–190)
Congo	All causes	1 300 (542–2 060)	742 (245–1 270)	600 (248–1 050)	470 (266–771)
Congo	Diarrheal diseases	506 (191–949)	195 (80·5–394)	354 (-22·3–876)	145 (61·3–275)
Congo	Lower respiratory infections	458 (261–695)	292 (18·0–611)	154 (81·5–253)	222 (140–333)
Congo	Malaria	228 (-121–808)	162 (-77·7–511)	--	94·4 (-37·4–454)
Congo	Measles	18·4 (5·65–38·1)	7·69 (2·24–17·5)	6·42 (1·63–16·7)	9·2 (2·66–20·6)
Democratic Republic of the Congo	All causes	50 800 (17 000–87 800)	32 400 (4 590–58 500)	15 400 (9 740–22 900)	25 200 (11 600–47 400)
Democratic Republic of the Congo	Diarrheal diseases	5 150 (1 910–11 100)	2 280 (893–5 110)	3 560 (-224–9 550)	1 820 (752–3 870)
Democratic Republic of the Congo	Lower respiratory infections	16 800 (9 190–25 600)	11 400 (691–21 800)	5 750 (2 750–10 300)	9 630 (5 770–15 300)
Democratic Republic of the Congo	Malaria	16 400 (-10 700–46 700)	11 300 (-6 210–30 900)	--	8 350 (-4 040–33 100)
Democratic Republic of the Congo	Measles	9 520 (3 460–19 200)	4 590 (1 600–9 450)	3 220 (878–7 340)	5 410 (1 730–11 000)
Equatorial Guinea	All causes	234 (58·0–481)	132 (5·97–284)	64·1 (33·5–106)	99·6 (36·1–237)
Equatorial Guinea	Diarrheal diseases	29·8 (9–65·6)	8·73 (3–19·9)	20·5 (-0·849–60·2)	8·63 (3·09–17·6)
Equatorial Guinea	Lower respiratory infections	80·9 (38·1–139)	46·9 (2·34–116)	23·8 (11·1–43·1)	38·0 (18·1–66·8)
Equatorial Guinea	Malaria	87·9 (-41–295)	56·2 (-23·4–164)	--	40·4 (-17·3–178)
Equatorial Guinea	Measles	22·1 (6·31–46·8)	7·06 (1·94–14·8)	6·84 (1·56–17·3)	12·5 (3·43–28·9)
Gabon	All causes	198 (94·7–320)	102 (33·0–196)	75·1 (35·9–134)	76·6 (46·2–119)
Gabon	Diarrheal diseases	57·2 (20·5–113)	17·4 (6·08–36·5)	38·0 (-1·73–104)	16·1 (6·39–30·9)
Gabon	Lower respiratory infections	72·1 (39·4–123)	42·5 (2–104)	17·8 (8·49–30·3)	34·1 (20·4–53·5)
Gabon	Malaria	30·6 (-13·9–112)	20·9 (-8·7–66·1)	--	12·3 (-4·8–61·4)
Gabon	Measles	25·7 (8·48–50·1)	9·05 (2·96–18·4)	6·9 (1·82–15·4)	14·0 (4·05–28·9)
Eastern Sub-Saharan Africa	All causes	154 000 (93 900–210 000)	94 300 (45 600–135 000)	70 900 (40 500–101 000)	66 300 (47 900–87 200)
Eastern Sub-Saharan Africa	Diarrheal diseases	48 800 (30 500–71 600)	22 700 (12 300–37 600)	32 600 (-2 040–66 700)	17 900 (10 600–27 400)
Eastern Sub-Saharan Africa	Lower respiratory infections	48 200 (31 600–63 800)	32 400 (2 260–57 700)	15 600 (9 030–23 500)	26 900 (17 800–36 600)

Eastern Sub-Saharan Africa	Malaria	18 900<br>(-11 500–63 100)	13 200<br>(-7 170–40 100)	--	9 040<br>(-4 540–42 100)
Eastern Sub-Saharan Africa	Measles	23 000<br>(9 650–42 000)	11 500<br>(4 400–22 000)	8 030<br>(2 570–16 200)	12 400<br>(4 680–22 900)
Burundi	All causes	10 300<br>(2 350–16 300)	7 180<br>(880–11 900)	3 170<br>(1 760–5 480)	5 300<br>(1 780–9 590)
Burundi	Diarrheal diseases	1 990<br>(874–3 760)	1 070<br>(445–2 130)	1 170<br>(-73·6–3 050)	928<br>(369–1 790)
Burundi	Lower respiratory infections	3 210<br>(1 720–5 090)	2 320<br>(222–4 770)	844<br>(405–1 420)	2 080<br>(1 160–3 090)
Burundi	Malaria	3 360<br>(-2 650–9 070)	2 380<br>(-1 510–6 550)	--	1 790<br>(-1 000–7 080)
Burundi	Measles	783<br>(248–1 530)	432<br>(123–896)	188<br>(44·6–462)	503<br>(151–1 040)
Comoros	All causes	207<br>(148–267)	124<br>(59·4–174)	122<br>(62·9–177)	85·1<br>(59·9–114)
Comoros	Diarrheal diseases	82·6<br>(39·5–141)	36·3<br>(17·1–66·8)	59·3<br>(-3·93–128)	29·2<br>(14·5–49·5)
Comoros	Lower respiratory infections	101<br>(66·0–145)	66·8<br>(4·66–131)	42·5<br>(24·5–68·4)	52·9<br>(32·7–78·7)
Comoros	Malaria	1·06<br>(-0·725–4·48)	0·723<br>(-0·45–2·93)	--	0·534<br>(-0·238–2·68)
Comoros	Measles	4·55<br>(1·64–9·74)	2<br>(0·696–4·48)	1·92<br>(0·546–4·4)	2·44<br>(0·817–5·5)
Djibouti	All causes	366<br>(257–477)	234<br>(143–308)	265<br>(134–382)	117<br>(77·5–159)
Djibouti	Diarrheal diseases	169<br>(79·8–279)	84·0<br>(39·9–144)	132<br>(-10·9–264)	51·0<br>(22·9–85·4)
Djibouti	Lower respiratory infections	125<br>(77·4–186)	85·9<br>(6·9–160)	70·8<br>(41·1–109)	57·3<br>(33·6–87·9)
Djibouti	Malaria	4·22<br>(-2·27–23·9)	3·15<br>(-1·39–17·3)	--	1·88<br>(-0·802–10·8)
Djibouti	Measles	14·9<br>(4·75–31·8)	7·65<br>(2·36–16·7)	8·53<br>(2·48–19·7)	6·67<br>(2·04–14·9)
Eritrea	All causes	3 420<br>(2 660–4 120)	2 400<br>(1 440–2 980)	2 160<br>(1 360–2 760)	1 270<br>(984–1 620)
Eritrea	Diarrheal diseases	1 090<br>(580–1 850)	576<br>(286–1 000)	791<br>(-67·7–1 600)	355<br>(189–583)
Eritrea	Lower respiratory infections	1 430<br>(978–1 970)	1 030<br>(93·2–1 740)	622<br>(348–962)	789<br>(538–1 090)
Eritrea	Malaria	15·7<br>(-12·3–62·9)	12·2<br>(-7·68–47·8)	--	7<br>(-3·44–30·5)
Eritrea	Measles	235<br>(81·0–501)	132<br>(45·0–291)	101<br>(29·8–223)	118<br>(38·5–252)
Ethiopia	All causes	28 300<br>(18 200–37 700)	17 900<br>(9 030–25 800)	14 000<br>(9 150–19 600)	12 200<br>(8 220–15 900)
Ethiopia	Diarrheal diseases	6 770<br>(3 680–11 400)	3 210<br>(1 450–5 980)	4 680<br>(-302–10 400)	2 480<br>(1 230–4 390)
Ethiopia	Lower respiratory infections	9 470<br>(5 800–13 900)	6 510<br>(562–12 300)	3 660<br>(1 980–5 920)	5 220<br>(3 190–7 690)
Ethiopia	Malaria	2 500<br>(-1 800–9 130)	1 810<br>(-1 120–6 290)	--	1 160<br>(-498–5 590)
Ethiopia	Measles	6 170<br>(2 490–11 800)	3 060<br>(1 200–5 930)	2 260<br>(719–4 810)	3 380<br>(1 260–6 640)
Kenya	All causes	7 860<br>(4 980–10 300)	4 510<br>(1 920–6 660)	3 520<br>(1 590–5 390)	3 390<br>(2 620–4 320)
Kenya	Diarrheal diseases	3 170<br>(1 910–4 790)	1 320<br>(711–2 170)	2 020<br>(-95·7–4 280)	1 140<br>(681–1 790)

Kenya	Lower respiratory infections	3 660 (2 300–5 030)	2 390 (155–4 600)	1 040 (581–1 580)	1 960 (1 270–2 800)
Kenya	Malaria	410 (-239–1 470)	294 (-158–935)	--	173 (-77·7–873)
Kenya	Measles	231 (82·8–480)	116 (39·9–241)	69·5 (19·8–162)	118 (39·7–253)
Madagascar	All causes	16 000 (10 600–20 700)	10 000 (5 330–13 200)	7 590 (3 150–11 800)	7 180 (5 580–8 750)
Madagascar	Diarrheal diseases	7 020 (3 960–10 900)	3 560 (1 700–5 740)	4 410 (-202–9 210)	2 840 (1 440–4 370)
Madagascar	Lower respiratory infections	4 700 (3 210–6 310)	3 310 (216–5 390)	1 430 (761–2 250)	2 800 (1 760–3 980)
Madagascar	Malaria	1 370 (-899–4 220)	1 010 (-542–3 060)	--	635 (-301–2 880)
Madagascar	Measles	1 580 (584–3 230)	849 (308–1 740)	464 (130–1 050)	912 (291–1 780)
Malawi	All causes	5 060 (2 520–8 040)	2 680 (1 240–4 320)	1 870 (725–3 430)	2 310 (1 440–3 770)
Malawi	Diarrheal diseases	1 840 (930–3 110)	677 (299–1 210)	1 080 (-37·2–2 740)	703 (357–1 170)
Malawi	Lower respiratory infections	1 080 (579–1 630)	670 (30·0–1 350)	217 (99·6–386)	633 (366–961)
Malawi	Malaria	890 (-494–3 570)	580 (-289–2 040)	--	437 (-180–2 340)
Malawi	Measles	851 (286–1 690)	344 (109–669)	164 (40·4–389)	538 (168–1 080)
Mozambique	All causes	14 100 (7 880–20 300)	8 360 (3 210–12 700)	5 880 (2 830–8 850)	6 260 (4 630–8 610)
Mozambique	Diarrheal diseases	4 630 (2 420–7 040)	1 930 (925–3 340)	2 870 (-130–6 240)	1 790 (989–2 870)
Mozambique	Lower respiratory infections	5 500 (3 630–7 410)	3 590 (179–6 630)	1 540 (842–2 460)	3 010 (1 820–4 420)
Mozambique	Malaria	1 810 (-1 040–6 300)	1 210 (-616–3 840)	--	885 (-409–4 360)
Mozambique	Measles	950 (311–2 020)	407 (125–890)	243 (59·5–594)	575 (179–1 280)
Rwanda	All causes	3 320 (1 890–4 870)	1 900 (632–3 120)	1 060 (627–1 740)	1 730 (1 180–2 230)
Rwanda	Diarrheal diseases	799 (399–1 420)	300 (126–595)	442 (-18·3–1 190)	343 (166–622)
Rwanda	Lower respiratory infections	1 570 (950–2 260)	977 (59·8–2 010)	288 (151–478)	973 (666–1 360)
Rwanda	Malaria	374 (-205–1 330)	228 (-114–727)	--	207 (-95·1–937)
Rwanda	Measles	305 (105–593)	119 (38·9–236)	52·5 (13·4–121)	207 (68·5–424)
Somalia	All causes	23 400 (16 600–29 300)	13 500 (9 550–16 400)	13 300 (6 490–19 500)	9 390 (6 110–12 200)
Somalia	Diarrheal diseases	9 400 (4 870–14 700)	4 790 (2 310–8 230)	6 760 (-499–13 400)	3 110 (1 550–5 200)
Somalia	Lower respiratory infections	3 200 (1 890–5 000)	2 220 (174–4 250)	1 340 (669–2 310)	1 700 (1 000–2 610)
Somalia	Malaria	290 (-185–1 010)	221 (-121–729)	--	118 (-47·5–497)
Somalia	Measles	9 120 (4 020–14 600)	4 810 (1 890–8 340)	3 730 (1 260–7 030)	4 460 (1 620–7 540)
South Sudan	All causes	10 600 (6 090–14 200)	6 680 (3 530–8 970)	6 790 (3 010–10 000)	3 780 (2 540–5 010)

South Sudan	Diarrheal diseases	5 290 (2 500–8 230)	2 670 (1 360–4 540)	4 070 (-326–7 830)	1 660 (833–2 750)
South Sudan	Lower respiratory infections	3 150 (1 850–4 820)	2 190 (188–4 290)	1 660 (917–2 560)	1 560 (935–2 340)
South Sudan	Malaria	1 010 (-638–3 190)	762 (-418–2 260)	--	443 (-198–1 920)
South Sudan	Measles	246 (73·0–551)	128 (37·7–298)	137 (35·9–320)	113 (30·5–268)
Uganda	All causes	12 000 (2 860–23 500)	7 670 (916–14 600)	3 810 (2 090–6 340)	4 910 (2 020–10 500)
Uganda	Diarrheal diseases	2 140 (848–4 050)	848 (317–1 810)	1 350 (-77·7–3 420)	734 (282–1 520)
Uganda	Lower respiratory infections	3 540 (1 850–5 780)	2 290 (147–4 940)	962 (467–1 690)	1 860 (999–2 900)
Uganda	Malaria	4 110 (-2 190–14 400)	2 880 (-1 400–8 880)	--	1 790 (-757–8 160)
Uganda	Measles	929 (258–2 150)	397 (108–929)	241 (57·3–629)	530 (139–1 240)
United Republic of Tanzania	All causes	12 100 (7 270–17 500)	7 450 (2 840–12 000)	4 580 (3 300–6 060)	5 360 (3 860–7 960)
United Republic of Tanzania	Diarrheal diseases	2 410 (1 230–3 700)	919 (465–1 610)	1 500 (-80·4–3 220)	897 (486–1 460)
United Republic of Tanzania	Lower respiratory infections	4 930 (3 060–7 090)	3 140 (127–5 940)	1 240 (671–2 020)	2 750 (1 730–4 070)
United Republic of Tanzania	Malaria	1 970 (-1 120–6 850)	1 310 (-646–4 010)	--	961 (-405–4 570)
United Republic of Tanzania	Measles	1 230 (449–2 420)	518 (185–1 000)	280 (75·2–636)	754 (255–1 480)
Zambia	All causes	6 350 (3 500–9 280)	3 590 (1 580–5 980)	2 810 (1 560–4 350)	2 880 (1 890–4 000)
Zambia	Diarrheal diseases	1 990 (1 100–3 370)	701 (311–1 330)	1 280 (-49·4–2 990)	779 (413–1 310)
Zambia	Lower respiratory infections	2 490 (1 540–3 750)	1 520 (91·3–3 310)	655 (332–1 130)	1 470 (961–2 210)
Zambia	Malaria	760 (-439–2 490)	458 (-226–1 380)	--	427 (-188–1 850)
Zambia	Measles	318 (116–665)	120 (40·1–256)	87·1 (22·1–194)	201 (69·4–440)
Southern Sub-Saharan Africa	All causes	15 700 (12 000–19 200)	9 860 (5 800–13 200)	8 290 (5 430–11 100)	5 650 (4 000–7 400)
Southern Sub-Saharan Africa	Diarrheal diseases	4 680 (2 770–6 550)	1 610 (869–2 580)	2 700 (-82·3–5 950)	1 870 (1 220–2 710)
Southern Sub-Saharan Africa	Lower respiratory infections	6 550 (4 570–8 710)	4 010 (202–7 980)	1 450 (834–2 190)	3 560 (2 210–5 080)
Southern Sub-Saharan Africa	Malaria	66·1 (-38·5–272)	45·6 (-24·3–180)	--	30·0 (-11·4–141)
Southern Sub-Saharan Africa	Measles	309 (109–652)	117 (39·1–266)	67·0 (18·0–160)	191 (66·3–427)
Botswana	All causes	360 (258–449)	223 (113–329)	217 (136–283)	130 (85·1–187)
Botswana	Diarrheal diseases	97·2 (45·0–175)	33·9 (14·8–66·3)	67·9 (-2·89–156)	35·9 (18·4–63·2)
Botswana	Lower respiratory infections	175 (119–247)	106 (5·44–216)	66·1 (37·2–103)	90·3 (49·6–141)
Botswana	Malaria	0·313 (-0·194–1·38)	0·19 (-0·104–0·776)	--	0·173 (-0·0778–0·935)
Botswana	Measles	7·04 (2·3–15·0)	2·57 (0·807–5·86)	2·55 (0·687–5·79)	4·03 (1·32–8·76)

Eswatini	All causes	276 (202–361)	173 (67·1–294)	99·5 (75·0–132)	120 (77·5–171)
Eswatini	Diarrheal diseases	38·7 (16·1–79·0)	12·9 (4·92–26·7)	20·7 (-0·578–61·0)	15·8 (7·11–30·6)
Eswatini	Lower respiratory infections	185 (114–261)	111 (5·04–235)	31·1 (16·7–51·0)	101 (60·0–153)
Eswatini	Malaria	0·25 (-0·135–1·27)	0·144 (-0·0678–0·693)	--	0·137 (-0·0547–0·831)
Eswatini	Measles	5·33 (1·8–11·6)	1·81 (0·574–4·19)	0·751 (0·193–1·78)	3·63 (1·18–7·94)
Lesotho	All causes	530 (382–682)	318 (124–485)	217 (126–330)	245 (159–335)
Lesotho	Diarrheal diseases	144 (64·3–266)	55·9 (24·5–113)	84·8 (-2·69–223)	59·1 (26·5–112)
Lesotho	Lower respiratory infections	324 (221–445)	207 (10·7–392)	78·8 (43·8–120)	179 (105–265)
Lesotho	Malaria	0 (0–0)	0 (0–0)	--	0 (0–0)
Lesotho	Measles	10·8 (3·91–23·6)	4·45 (1·48–10·3)	2·16 (0·572–5·47)	6·91 (2·32–14·0)
Namibia	All causes	572 (418–721)	387 (190–553)	345 (247–435)	165 (110–235)
Namibia	Diarrheal diseases	109 (49·1–204)	40·1 (16·3–78·7)	79·0 (-4·04–190)	29·6 (13·5–53·6)
Namibia	Lower respiratory infections	298 (191–412)	190 (11·1–364)	113 (64·4–179)	128 (77·4–198)
Namibia	Malaria	4·95 (-2·94–31·4)	3·58 (-2·01–22·6)	--	2·09 (-0·793–13·2)
Namibia	Measles	10·7 (3·43–23·7)	4·64 (1·46–11·0)	3·86 (0·976–9·48)	5·27 (1·59–11·9)
South Africa	All causes	8 110 (6 310–10 100)	4 740 (2 740–6 750)	3 770 (2 400–5 360)	3 340 (2 300–4 380)
South Africa	Diarrheal diseases	2 700 (1 680–3 670)	891 (489–1 380)	1 450 (-38·1–3 310)	1 180 (780–1 570)
South Africa	Lower respiratory infections	3 670 (2 590–4 900)	2 190 (99·6–4 520)	681 (392–1 020)	2 080 (1 220–2 920)
South Africa	Malaria	0·475 (-0·236–2·97)	0·303 (-0·132–1·91)	--	0·23 (-0·0777–1·58)
South Africa	Measles	124 (44·0–261)	41·4 (13·4–88·1)	22·5 (6·01–52·0)	82·5 (28·4–179)
Zimbabwe	All causes	5 830 (4 270–7 190)	4 020 (2 570–5 250)	3 640 (2 380–4 830)	1 650 (1 140–2 220)
Zimbabwe	Diarrheal diseases	1 590 (790–2 620)	581 (270–1 040)	990 (-34·1–2 310)	554 (292–935)
Zimbabwe	Lower respiratory infections	1 900 (1 100–2 720)	1 200 (66·3–2 380)	479 (254–764)	977 (577–1 480)
Zimbabwe	Malaria	60·1 (-34·5–232)	41·4 (-21·8–150)	--	27·4 (-10·4–131)
Zimbabwe	Measles	151 (52·9–325)	62·3 (20·4–143)	35·2 (9·34–84·7)	88·8 (29·4–197)
Western Sub-Saharan Africa	All causes	372 000 (170 000–521 000)	247 000 (87 300–365 000)	177 000 (95 800–258 000)	155 000 (92 500–234 000)
Western Sub-Saharan Africa	Diarrheal diseases	110 000 (61 100–174 000)	54 200 (28 800–90 500)	77 600 (-5 690–156 000)	37 200 (19 200–61 800)
Western Sub-Saharan Africa	Lower respiratory infections	124 000 (73 800–180 000)	86 500 (7 380–160 000)	46 300 (25 200–74 800)	71 400 (44 000–103 000)
Western Sub-Saharan Africa	Malaria	65 800 (-47 500–206 000)	48 400 (-28 600–145 000)	--	30 600 (-16 400–136 000)

Western Sub-Saharan Africa	Measles	31 400 (11 100–62 700)	16 700 (5 900–33 800)	12 300 (3 570–27 000)	16 300 (5 370–33 400)
Benin	All causes	6 950 (2 550–11 500)	4 590 (1 090–7 980)	2 600 (1 510–4 060)	2 940 (1 670–5 530)
Benin	Diarrheal diseases	1 240 (543–2 350)	532 (205–1 110)	811 (-47·2–2 030)	420 (171–856)
Benin	Lower respiratory infections	2 450 (1 430–3 640)	1 630 (111–3 200)	716 (364–1 220)	1 400 (796–2 030)
Benin	Malaria	1 720 (-1 060–5 560)	1 240 (-645–3 650)	--	760 (-355–3 700)
Benin	Measles	662 (199–1 490)	310 (95·3–695)	200 (50·5–507)	363 (111–819)
Burkina Faso	All causes	10 500 (4 670–16 900)	7 100 (1 480–12 100)	4 150 (2 640–5 830)	4 030 (2 640–7 130)
Burkina Faso	Diarrheal diseases	2 000 (1 070–2 940)	970 (467–1 650)	1 410 (-103–2 580)	618 (329–1 030)
Burkina Faso	Lower respiratory infections	3 370 (2 240–4 750)	2 350 (151–4 140)	1 310 (770–1 980)	1 670 (1 140–2 310)
Burkina Faso	Malaria	2 750 (-1 660–8 210)	2 150 (-1 180–5 960)	--	1 010 (-426–4 690)
Burkina Faso	Measles	1 550 (559–3 200)	811 (286–1 630)	605 (180–1 340)	731 (244–1 500)
Cabo Verde	All causes	17·5 (10·8–25·4)	10·0 (5·38–15·1)	10·1 (5·64–16·3)	4·44 (2·99–6·5)
Cabo Verde	Diarrheal diseases	5·98 (2·15–11·5)	1·9 (0·737–3·89)	4·34 (-0·23–10·9)	1·24 (0·55–2·36)
Cabo Verde	Lower respiratory infections	7·37 (4·44–11·9)	4·69 (0·262–10·6)	2·43 (1·29–3·92)	2·71 (1·64–4·31)
Cabo Verde	Malaria	<0.001 (>-0.001–<0.001)	<0.001 (>-0.001–<0.001)	--	<0.001 (>-0.001–<0.001)
Cabo Verde	Measles	1·18 (0·406–2·49)	0·483 (0·164–1·08)	0·425 (0·118–0·964)	0·493 (0·165–1·11)
Cameroon	All causes	16 300 (7 990–24 800)	9 080 (3 380–14 900)	6 630 (2 830–11 800)	6 970 (4 430–10 300)
Cameroon	Diarrheal diseases	5 730 (2 570–10 100)	2 220 (977–4 110)	3 740 (-184–8 890)	1 900 (899–3 280)
Cameroon	Lower respiratory infections	5 220 (2 940–7 800)	3 310 (194–6 970)	1 410 (704–2 410)	2 910 (1 860–4 240)
Cameroon	Malaria	2 770 (-1 620–8 630)	1 860 (-968–5 240)	--	1 320 (-571–5 400)
Cameroon	Measles	1 480 (517–2 900)	638 (210–1 260)	430 (110–970)	832 (276–1 710)
Chad	All causes	37 900 (26 300–45 800)	21 700 (16 200–25 900)	23 900 (6 740–34 900)	13 400 (10 600–16 400)
Chad	Diarrheal diseases	23 400 (14 700–31 400)	11 500 (6 330–17 900)	17 000 (-1 110–30 100)	7 160 (4 270–10 700)
Chad	Lower respiratory infections	7 790 (4 780–12 000)	5 440 (444–10 600)	3 310 (1 650–5 430)	4 090 (2 500–6 000)
Chad	Malaria	1 320 (-836–4 330)	1 000 (-551–3 090)	--	559 (-247–2 610)
Chad	Measles	3 380 (1 120–6 960)	1 780 (565–3 810)	1 550 (448–3 420)	1 600 (463–3 310)
Côte d'Ivoire	All causes	13 000 (5 120–20 800)	8 240 (1 690–14 200)	5 260 (3 010–7 840)	4 800 (2 710–7 410)
Côte d'Ivoire	Diarrheal diseases	2 850 (1 180–5 300)	1 130 (462–2 310)	2 020 (-134–4 780)	787 (366–1 490)
Côte d'Ivoire	Lower respiratory infections	5 620 (3 220–8 620)	3 670 (237–7 620)	1 940 (1 010–3 320)	2 650 (1 590–3 980)

Côte d'Ivoire	Malaria	2 760 (-1 450–8 890)	2 060 (-976–5 950)	--	1 040 (-430–4 430)
Côte d'Ivoire	Measles	662 (218–1 520)	298 (97·3–704)	234 (63·0–575)	319 (97·4–720)
Gambia	All causes	604 (409–773)	394 (143–589)	264 (205–330)	230 (179–293)
Gambia	Diarrheal diseases	80·4 (35·3–128)	33·0 (14·7–63·7)	55·3 (-3·53–115)	21·5 (10·5–38·7)
Gambia	Lower respiratory infections	333 (222–443)	222 (14·9–386)	108 (55·6–168)	160 (108–220)
Gambia	Malaria	33·0 (-20·2–125)	26·8 (-15·4–101)	--	9·7 (-3·5–51·8)
Gambia	Measles	87·3 (32·2–164)	41·9 (14·6–83·3)	30·5 (8·84–64·1)	39·2 (12·4–77·1)
Ghana	All causes	4 600 (1 770–7 920)	3 260 (848–5 950)	2 010 (1 250–2 960)	1 340 (627–2 760)
Ghana	Diarrheal diseases	648 (288–1 210)	250 (97·5–512)	465 (-26·3–1 050)	156 (64·2–307)
Ghana	Lower respiratory infections	1 490 (789–2 320)	974 (45·3–1 950)	525 (249–921)	659 (370–1 050)
Ghana	Malaria	1 270 (-625–4 600)	998 (-481–3 210)	--	408 (-157–1 940)
Ghana	Measles	269 (82·7–571)	122 (38·1–252)	99·2 (26·3–231)	119 (35·2–270)
Guinea	All causes	9 460 (4 930–13 900)	5 980 (1 700–9 670)	3 650 (2 440–4 990)	4 280 (2 450–6 510)
Guinea	Diarrheal diseases	1 110 (454–2 280)	482 (175–947)	771 (-51·1–1 930)	360 (148–698)
Guinea	Lower respiratory infections	3 920 (2 330–5 850)	2 610 (190–5 130)	1 380 (747–2 280)	2 100 (1 280–3 030)
Guinea	Malaria	1 670 (-1 020–5 100)	1 180 (-631–3 370)	--	769 (-347–3 270)
Guinea	Measles	2 010 (778–3 900)	946 (342–1 860)	734 (214–1 640)	1 050 (341–2 080)
Guinea-Bissau	All causes	932 (624–1 220)	565 (273–802)	391 (237–602)	426 (311–549)
Guinea-Bissau	Diarrheal diseases	252 (120–449)	111 (48·2–210)	160 (-8·2–390)	91·0 (42·4–163)
Guinea-Bissau	Lower respiratory infections	353 (215–509)	237 (17·3–428)	93·8 (45·7–155)	210 (141–295)
Guinea-Bissau	Malaria	58·5 (-34–201)	43·2 (-23·4–137)	--	24·3 (-9·81–112)
Guinea-Bissau	Measles	183 (68·3–349)	87·5 (31·7–179)	51·7 (14·8–116)	101 (35·0–192)
Liberia	All causes	2 700 (1 340–4 020)	1 640 (526–2 720)	1 040 (608–1 540)	1 050 (682–1 530)
Liberia	Diarrheal diseases	616 (242–1 090)	238 (92·8–473)	410 (-19·9–902)	181 (75·0–355)
Liberia	Lower respiratory infections	971 (522–1 530)	638 (43·1–1 420)	279 (142–469)	464 (266–731)
Liberia	Malaria	459 (-240–1 540)	336 (-165–1 020)	--	182 (-74·3–854)
Liberia	Measles	422 (149–841)	190 (62·9–388)	122 (32·5–268)	221 (70·7–454)
Mali	All causes	18 300 (9 870–26 300)	11 500 (4 620–17 200)	8 350 (5 210–11 900)	6 590 (4 320–11 100)
Mali	Diarrheal diseases	4 500 (1 970–7 630)	2 020 (985–3 540)	3 230 (-197–6 740)	1 240 (623–2 190)

Mali	Lower respiratory infections	3 970 (2 550–5 730)	2 700 (194–5 130)	1 510 (830–2 410)	1 990 (1 260–2 910)
Mali	Malaria	3 420 (-2 060–11 000)	2 640 (-1 430–7 800)	--	1 280 (-541–5 880)
Mali	Measles	4 580 (1 710–8 910)	2 310 (814–4 540)	1 820 (534–3 840)	2 070 (665–3 990)
Mauritania	All causes	983 (572–1 350)	583 (291–841)	562 (247–891)	304 (220–408)
Mauritania	Diarrheal diseases	430 (217–735)	182 (85·1–318)	311 (-19·8–698)	105 (49·7–185)
Mauritania	Lower respiratory infections	356 (206–551)	241 (17·3–486)	135 (70·9–224)	159 (102–233)
Mauritania	Malaria	48·3 (-28–209)	38·2 (-21·1–175)	--	16·4 (-5·97–82·5)
Mauritania	Measles	55·5 (18·0–113)	27·4 (8·88–58·8)	21·9 (6·09–53·1)	24·2 (7·71–54·7)
Niger	All causes	49 900 (24 400–65 200)	36 200 (14 100–48 300)	23 900 (14 800–34 000)	22 800 (13 400–32 700)
Niger	Diarrheal diseases	13 000 (6 960–21 500)	7 690 (3 940–13 400)	9 030 (-800–18 500)	5 100 (2 540–8 910)
Niger	Lower respiratory infections	16 600 (10 300–24 300)	12 400 (1 300–21 500)	6 400 (3 450–10 400)	10 400 (6 830–15 300)
Niger	Malaria	8 330 (-7 010–24 200)	6 290 (-4 340–17 500)	--	4 180 (-2 370–16 800)
Niger	Measles	5 560 (1 850–11 700)	3 400 (1 120–7 510)	2 120 (587–4 820)	3 050 (903–6 480)
Nigeria	All causes	188 000 (70 200–270 000)	129 000 (36 600–194 000)	88 600 (45 500–130 000)	81 800 (42 100–132 000)
Nigeria	Diarrheal diseases	49 300 (23 400–85 000)	24 800 (11 700–43 000)	34 900 (-2 720–77 300)	17 600 (8 150–31 500)
Nigeria	Lower respiratory infections	67 300 (37 300–96 400)	47 400 (4 290–86 400)	25 800 (12 900–43 000)	40 600 (23 000–60 500)
Nigeria	Malaria	37 400 (-28 900–117 000)	27 300 (-17 000–82 100)	--	18 300 (-10 400–80 600)
Nigeria	Measles	9 620 (3 330–19 300)	5 370 (1 760–11 100)	3 950 (1 080–9 170)	5 380 (1 710–11 300)
Sao Tome and Principe	All causes	24·6 (18·6–31·5)	17·8 (11·1–25·3)	15·3 (11·2–20·3)	6·58 (4·41–9·18)
Sao Tome and Principe	Diarrheal diseases	2·22 (0·848–4·37)	0·76 (0·277–1·55)	1·5 (-0·0724–3·9)	0·664 (0·253–1·28)
Sao Tome and Principe	Lower respiratory infections	8·82 (5·45–13·7)	5·39 (0·293–11·1)	2·48 (1·17–4·13)	4·36 (2·63–6·64)
Sao Tome and Principe	Malaria	0·288 (-0·15–1·09)	0·21 (-0·107–0·736)	--	0·107 (-0·0391–0·586)
Sao Tome and Principe	Measles	2·95 (1·02–5·98)	1·14 (0·385–2·47)	0·976 (0·286–2·16)	1·46 (0·503–3·12)
Senegal	All causes	2 770 (1 780–3 670)	1 680 (838–2 460)	1 380 (880–1 860)	897 (624–1 250)
Senegal	Diarrheal diseases	675 (313–1 060)	263 (120–489)	475 (-28·9–932)	167 (78·5–283)
Senegal	Lower respiratory infections	928 (535–1 410)	613 (28·9–1 220)	313 (158–547)	415 (262–641)
Senegal	Malaria	212 (-113–805)	168 (-88·8–589)	--	66·4 (-22·9–349)
Senegal	Measles	596 (218–1 090)	272 (95·8–529)	232 (70·0–483)	249 (79·1–478)
Sierra Leone	All causes	6 420 (2 870–9 830)	3 830 (1 130–6 400)	2 950 (1 270–4 480)	2 420 (1 620–3 910)

Sierra Leone	Diarrheal diseases	2 430 (1 200–3 860)	1 010 (500–1 850)	1 660 (-96·1–3 420)	764 (410–1 320)
Sierra Leone	Lower respiratory infections	2 200 (1 360–3 280)	1 460 (62·3–2 880)	758 (396–1 270)	1 060 (638–1 700)
Sierra Leone	Malaria	1 160 (-663–4 120)	813 (-402–2 620)	--	518 (-234–2 440)
Sierra Leone	Measles	161 (48·2–361)	72·3 (20·5–167)	58·8 (14·1–157)	81·7 (21·3–188)
Togo	All causes	3 320 (1 670–4 850)	1 920 (777–2 820)	1 680 (515–2 980)	1 150 (725–1 510)
Togo	Diarrheal diseases	1 680 (839–2 760)	716 (342–1 280)	1 150 (-73·2–2 560)	480 (251–842)
Togo	Lower respiratory infections	926 (521–1 400)	621 (48·1–1 240)	292 (145–489)	479 (287–703)
Togo	Malaria	423 (-243–1 440)	328 (-181–1 010)	--	150 (-65·4–752)
Togo	Measles	78·7 (23·8–167)	37·6 (11·4–82·0)	26·3 (6·42–66·4)	37·9 (11·0–87·1)

Table S4. All-cause and cause-specific population attributable fraction (PAF, %) of deaths among children under 5 years for child growth failure at the global and super-regional, regional, and national levels, 2023 Estimates combine burden associated with mild, moderate, and severe forms of CGF: stunting was defined as height-for-age z-score (HAZ) < –1; underweight as weight-for-age z-score (WAZ) < –1; wasting as weight-for-height z-score (WHZ) < –1, according to WHO Child Growth Standards.

Location	Cause name	Child growth failure	Child underweight	Child wasting	Child stunting
**Global**	**All causes**	**18·8 (11·1–25·0)**	**12·3 (5·1–17·6)**	**9·2 (5·5–12·5)**	**8·0 (6·0–10·3)**
**Global**	**Diarrheal diseases**	**76·7 (54·9–89·1)**	**37·2 (24·7–48·4)**	**53·2 (-3·1–86·1)**	**26·8 (19·2–33·0)**
**Global**	**Lower respiratory infections**	**59·4 (48·9–66·8)**	**40·8 (2·7–63·3)**	**23·1 (16·0–29·4)**	**32·1 (24·7–38·1)**
**Global**	**Malaria**	**26·4 (-17·8–69·5)**	**19·0 (-11·3–48·2)**	**--**	**12·4 (-6·4–45·9)**
**Global**	**Measles**	**66·6 (40·0–76·0)**	**34·2 (18·4–42·5)**	**24·9 (10·8–41·0)**	**35·5 (17·4–43·7)**
**Central Europe, Eastern Europe, and Central Asia**	**All causes**	**12·4 (9·0–16·2)**	**6·9 (0·72–13·8)**	**4·8 (3·8–6·0)**	**4·8 (3·2–6·4)**
**Central Europe, Eastern Europe, and Central Asia**	**Diarrheal diseases**	**61·2 (31·8–83·2)**	**18·9 (10·3–27·1)**	**44·8 (-1·8–81·1)**	**15·5 (9·9–20·1)**
**Central Europe, Eastern Europe, and Central Asia**	**Lower respiratory infections**	**43·6 (33·0–57·4)**	**25·0 (0·94–52·4)**	**15·3 (10·0–20·5)**	**17·6 (11·5–23·7)**
**Central Europe, Eastern Europe, and Central Asia**	**Malaria**	**0 (0–0)**	**0 (0–0)**	**--**	**0 (0–0)**
**Central Europe, Eastern Europe, and Central Asia**	**Measles**	**40·2 (19·5–49·8)**	**13·6 (6·4–18·0)**	**14·4 (5·5–26·4)**	**19·7 (9·0–26·4)**
Central Asia	All causes	14·2 (10·4–18·4)	8·0 (0·82–15·9)	5·6 (4·3–7·0)	5·4 (3·6–7·3)
Central Asia	Diarrheal diseases	65·0 (35·1–86·0)	21·3 (11·7–30·3)	47·6 (-2·0–83·8)	16·4 (10·5–21·3)
Central Asia	Lower respiratory infections	43·3 (32·7–56·8)	25·1 (0·95–52·0)	15·4 (10·1–20·7)	17·2 (11·3–23·1)
Central Asia	Malaria	0 (0–0)	0 (0–0)	--	0 (0–0)
Central Asia	Measles	40·4 (19·6–50·2)	13·7 (6·5–18·2)	14·5 (5·5–26·7)	19·7 (8·9–26·4)
Armenia	All causes	14·3 (10·5–18·3)	7·3 (0·44–15·6)	4·3 (3·0–5·9)	6·9 (4·4–9·4)
Armenia	Diarrheal diseases	51·7 (25·5–76·0)	12·0 (6·4–17·7)	35·7 (-0·97–73·5)	16·3 (10·8–21·2)
Armenia	Lower respiratory infections	45·3 (35·5–58·7)	23·7 (0·82–51·5)	12·9 (7·9–17·9)	22·4 (14·7–29·6)
Armenia	Malaria	0 (0–0)	0 (0–0)	--	0 (0–0)
Armenia	Measles	33·7 (15·6–42·5)	7·5 (3·4–10·2)	10·5 (3·7–19·9)	20·2 (9·1–26·9)
Azerbaijan	All causes	15·4 (10·7–20·6)	8·9 (1·6–16·8)	6·4 (4·6–8·1)	6·0 (3·5–8·7)
Azerbaijan	Diarrheal diseases	61·6 (33·3–83·1)	20·6 (11·5–29·5)	44·2 (-1·5–80·8)	16·3 (10·2–22·1)
Azerbaijan	Lower respiratory infections	44·3 (32·9–56·9)	27·2 (1·1–52·6)	15·7 (10·0–21·3)	18·3 (10·4–25·6)
Azerbaijan	Malaria	0 (0–0)	0 (0–0)	--	0 (0–0)
Azerbaijan	Measles	41·8 (20·2–51·4)	14·5 (6·8–19·1)	14·8 (5·6–27·0)	20·5 (9·2–27·2)

Georgia	All causes	5·4 (3·7–8·2)	2·7 (0·17–6·7)	1·3 (0·82–1·8)	2·5 (1·9–3·3)
Georgia	Diarrheal diseases	36·9 (17·1–61·3)	7·9 (3·9–11·8)	25·2 (-0·61–59·0)	9·1 (5·5–12·4)
Georgia	Lower respiratory infections	30·2 (21·1–45·0)	15·2 (0·43–37·5)	6·3 (3·5–9·1)	14·2 (11·1–18·3)
Georgia	Malaria	0 (0–0)	0 (0–0)	--	0 (0–0)
Georgia	Measles	22·7 (10·2–28·3)	5·5 (2·5–7·6)	5·1 (1·8–9·9)	13·8 (6·1–18·7)
Kazakhstan	All causes	8·2 (5·9–11·1)	4·6 (0·71–9·7)	3·1 (2·3–4·2)	3·0 (1·9–4·2)
Kazakhstan	Diarrheal diseases	53·3 (24·3–80·8)	12·4 (6·4–18·3)	38·9 (-1·1–78·1)	12·8 (7·9–17·0)
Kazakhstan	Lower respiratory infections	38·5 (28·3–52·2)	21·1 (0·69–46·8)	12·4 (7·7–17·3)	15·5 (9·8–21·3)
Kazakhstan	Malaria	0 (0–0)	0 (0–0)	--	0 (0–0)
Kazakhstan	Measles	30·3 (13·7–38·7)	8·2 (3·7–11·8)	10·0 (3·7–19·3)	15·8 (6·9–21·6)
Kyrgyzstan	All causes	9·1 (6·3–13·1)	4·7 (0·58–10·6)	2·8 (2·2–3·5)	3·9 (2·8–5·5)
Kyrgyzstan	Diarrheal diseases	50·8 (25·3–79·0)	11·7 (6·0–17·2)	34·2 (-0·88–75·8)	14·6 (9·2–19·3)
Kyrgyzstan	Lower respiratory infections	52·1 (38·9–72·2)	28·1 (0·91–65·2)	12·8 (7·9–17·6)	24·2 (16·1–32·1)
Kyrgyzstan	Malaria	0 (0–0)	0 (0–0)	--	0 (0–0)
Kyrgyzstan	Measles	34·0 (16·1–42·3)	9·6 (4·4–13·3)	8·9 (3·3–17·1)	20·0 (9·0–27·3)
Mongolia	All causes	7·3 (4·7–11·1)	3·7 (0·27–9·0)	1·4 (0·93–2·0)	3·6 (2·5–5·3)
Mongolia	Diarrheal diseases	38·0 (20·3–65·7)	8·1 (4·1–12·3)	23·6 (-0·51–61·6)	11·7 (7·1–15·8)
Mongolia	Lower respiratory infections	33·6 (24·6–48·8)	17·2 (0·50–41·0)	5·7 (3·3–8·0)	17·2 (11·9–22·4)
Mongolia	Malaria	0 (0–0)	0 (0–0)	--	0 (0–0)
Mongolia	Measles	0 (0–0)	0 (0–0)	0 (0–0)	0 (0–0)
Tajikistan	All causes	18·4 (13·1–23·6)	10·3 (2·1–16·8)	9·0 (6·0–11·8)	7·1 (5·0–9·6)
Tajikistan	Diarrheal diseases	72·8 (42·0–90·7)	26·4 (15·1–37·4)	53·8 (-2·8–88·9)	18·5 (12·1–24·2)
Tajikistan	Lower respiratory infections	67·4 (53·4–80·4)	42·4 (2·1–73·4)	26·7 (17·2–35·1)	29·5 (21·8–36·9)
Tajikistan	Malaria	0 (0–0)	0 (0–0)	--	0 (0–0)
Tajikistan	Measles	53·2 (28·3–64·2)	21·1 (10·5–27·3)	21·1 (8·5–36·5)	25·4 (11·7–32·4)
Turkmenistan	All causes	17·1 (12·0–22·5)	9·8 (0·55–19·6)	6·4 (4·4–8·5)	6·3 (4·6–8·4)
Turkmenistan	Diarrheal diseases	57·9 (26·8–81·3)	16·1 (8·6–23·5)	43·8 (-1·7–79·2)	11·4 (7·0–15·3)
Turkmenistan	Lower respiratory infections	51·4 (37·6–67·4)	30·2 (1·1–60·7)	18·3 (11·7–24·4)	19·3 (14·4–24·8)
Turkmenistan	Malaria	0 (0–0)	0 (0–0)	--	0 (0–0)

Turkmenistan	Measles	0 (0–0)	0 (0–0)	0 (0–0)	0 (0–0)
Uzbekistan	All causes	14·7 (10·6–19·6)	8·3 (0·40–17·7)	5·5 (3·6–7·4)	5·5 (3·5–7·6)
Uzbekistan	Diarrheal diseases	57·7 (26·9–82·0)	14·9 (7·9–21·9)	43·6 (-1·6–80·5)	12·6 (7·8–16·9)
Uzbekistan	Lower respiratory infections	39·4 (29·2–52·9)	22·4 (0·78–48·6)	14·3 (9·1–19·5)	15·0 (9·4–20·5)
Uzbekistan	Malaria	0 (0–0)	0 (0–0)	--	0 (0–0)
Uzbekistan	Measles	33·3 (14·8–44·4)	9·3 (4·0–13·1)	14·5 (5·2–26·7)	14·2 (6·3–19·7)
Central Europe	All causes	7·8 (5·4–11·1)	3·8 (0·70–7·9)	3·2 (2·1–4·0)	2·9 (1·9–4·0)
Central Europe	Diarrheal diseases	45·0 (17·9–70·6)	9·2 (4·6–13·4)	33·6 (-0·92–68·7)	10·2 (6·3–13·6)
Central Europe	Lower respiratory infections	48·0 (35·5–66·1)	26·2 (0·90–59·3)	14·0 (8·7–18·8)	20·4 (12·7–27·3)
Central Europe	Malaria	0 (0–0)	0 (0–0)	--	0 (0–0)
Central Europe	Measles	35·0 (16·8–43·0)	10·8 (4·9–15·1)	9·6 (3·6–17·8)	19·5 (9·0–25·8)
Albania	All causes	6·9 (4·2–10·2)	3·5 (0·61–8·0)	3·1 (2·0–4·4)	3·0 (1·5–4·8)
Albania	Diarrheal diseases	60·0 (28·3–84·7)	13·3 (7·1–20·4)	44·7 (-1·5–83·1)	18·9 (12·5–25·4)
Albania	Lower respiratory infections	57·7 (47·6–71·8)	29·4 (0·98–64·8)	21·9 (14·1–28·8)	27·9 (16·2–37·8)
Albania	Malaria	0 (0–0)	0 (0–0)	--	0 (0–0)
Albania	Measles	29·8 (0–56·9)	6·6 (0–13·1)	13·7 (0–35·7)	15·8 (0–31·0)
Bosnia and Herzegovina	All causes	3·1 (1·9–4·6)	1·2 (0·24–2·8)	1·4 (0·72–1·9)	1·3 (0·84–1·9)
Bosnia and Herzegovina	Diarrheal diseases	31·7 (12·8–49·4)	6·1 (3·0–9·7)	23·3 (-0·62–47·8)	9·0 (5·3–12·5)
Bosnia and Herzegovina	Lower respiratory infections	30·6 (21·5–43·4)	14·1 (0·41–35·4)	9·3 (5·5–13·3)	15·0 (10·1–19·7)
Bosnia and Herzegovina	Malaria	0 (0–0)	0 (0–0)	--	0 (0–0)
Bosnia and Herzegovina	Measles	22·6 (0–35·4)	3·8 (0–6·2)	7·6 (0–16·9)	13·5 (0–21·6)
Bulgaria	All causes	8·8 (5·6–13·9)	4·3 (0·50–10·7)	3·4 (2·5–4·2)	2·7 (1·9–3·9)
Bulgaria	Diarrheal diseases	44·3 (15·4–73·7)	8·6 (4·2–12·8)	34·1 (-0·96–72·0)	7·6 (4·5–10·4)
Bulgaria	Lower respiratory infections	42·1 (29·1–63·2)	22·5 (0·65–55·8)	13·3 (8·1–18·0)	14·4 (10·1–19·6)
Bulgaria	Malaria	0 (0–0)	0 (0–0)	--	0 (0–0)
Bulgaria	Measles	0 (0–0)	0 (0–0)	0 (0–0)	0 (0–0)
Croatia	All causes	5·5 (3·5–7·9)	2·3 (0·48–5·0)	2·4 (1·4–3·3)	2·0 (1·4–2·7)
Croatia	Diarrheal diseases	44·4 (17·0–70·1)	8·2 (4·1–12·4)	33·8 (-0·93–68·3)	9·9 (6·1–13·3)
Croatia	Lower respiratory infections	40·5 (30·5–56·4)	20·1 (0·59–49·1)	12·8 (7·9–17·6)	17·1 (12·2–22·5)

Croatia	Malaria	0 (0–0)	0 (0–0)	--	0 (0–0)
Croatia	Measles	0 (0–0)	0 (0–0)	0 (0–0)	0 (0–0)
Czechia	All causes	10·1 (5·6–15·1)	4·2 (1·6–8·4)	5·9 (2·5–8·7)	2·2 (1·5–3·1)
Czechia	Diarrheal diseases	47·3 (16·1–74·9)	10·2 (5·2–14·9)	37·9 (-1·1–73·5)	6·7 (3·9–9·1)
Czechia	Lower respiratory infections	39·9 (27·8–57·8)	21·7 (0·65–50·8)	15·1 (9·5–20·7)	11·8 (8·7–16·0)
Czechia	Malaria	0 (0–0)	0 (0–0)	--	0 (0–0)
Czechia	Measles	20·4 (9·0–29·0)	5·0 (2·3–6·8)	10·1 (3·6–19·1)	6·9 (3·1–9·4)
Hungary	All causes	6·0 (3·2–9·4)	1·8 (0·87–3·2)	3·6 (0·81–6·7)	1·8 (1·2–2·6)
Hungary	Diarrheal diseases	44·5 (16·4–71·8)	7·6 (3·7–11·4)	33·7 (-0·88–69·9)	10·5 (6·4–14·0)
Hungary	Lower respiratory infections	38·9 (29·5–54·2)	18·4 (0·52–46·0)	12·0 (7·3–16·4)	17·0 (11·4–22·5)
Hungary	Malaria	0 (0–0)	0 (0–0)	--	0 (0–0)
Hungary	Measles	0 (0–0)	0 (0–0)	0 (0–0)	0 (0–0)
Montenegro	All causes	4·9 (3·1–7·6)	2·5 (0·35–5·9)	1·6 (1·2–2·2)	2·1 (1·1–3·3)
Montenegro	Diarrheal diseases	45·9 (20·6–73·2)	9·3 (4·6–14·0)	31·8 (-0·84–70·6)	13·1 (8·1–17·8)
Montenegro	Lower respiratory infections	46·9 (34·2–66·5)	24·2 (0·70–60·2)	12·2 (7·3–16·8)	21·3 (12·3–29·5)
Montenegro	Malaria	0 (0–0)	0 (0–0)	--	0 (0–0)
Montenegro	Measles	0 (0–0)	0 (0–0)	0 (0–0)	0 (0–0)
North Macedonia	All causes	5·1 (2·9–7·4)	1·6 (0·41–3·5)	2·9 (1·1–4·5)	1·7 (1·0–2·5)
North Macedonia	Diarrheal diseases	46·5 (14·8–73·4)	6·3 (3·0–9·7)	37·1 (-1·1–72·0)	10·3 (6·4–13·8)
North Macedonia	Lower respiratory infections	42·4 (33·9–56·4)	18·1 (0·48–47·7)	15·8 (10·0–21·3)	17·9 (10·8–25·0)
North Macedonia	Malaria	0 (0–0)	0 (0–0)	--	0 (0–0)
North Macedonia	Measles	26·1 (11·8–32·9)	5·0 (2·4–6·6)	9·2 (3·4–16·9)	14·3 (6·5–19·1)
Poland	All causes	4·2 (2·6–6·2)	1·6 (0·39–3·6)	1·9 (0·98–2·9)	1·5 (1·0–2·1)
Poland	Diarrheal diseases	41·0 (15·2–67·0)	7·2 (3·5–10·8)	31·2 (-0·85–65·4)	9·1 (5·6–12·3)
Poland	Lower respiratory infections	43·1 (32·4–61·8)	21·2 (0·60–53·5)	13·2 (8·1–18·0)	18·4 (13·0–24·2)
Poland	Malaria	0 (0–0)	0 (0–0)	--	0 (0–0)
Poland	Measles	25·3 (11·4–33·0)	5·8 (2·6–7·7)	9·1 (3·3–17·0)	12·9 (5·7–17·8)
Romania	All causes	13·7 (10·1–19·1)	7·7 (0·99–15·8)	4·3 (3·2–5·5)	5·8 (3·4–8·1)
Romania	Diarrheal diseases	46·9 (24·9–69·6)	12·8 (6·5–18·3)	30·9 (-0·80–66·4)	13·7 (8·4–17·8)

Romania	Lower respiratory infections	54·3 (39·6–72·6)	31·7 (1·2–67·1)	13·9 (8·7–19·0)	24·4 (13·9–32·8)
Romania	Malaria	0 (0–0)	0 (0–0)	--	0 (0–0)
Romania	Measles	35·3 (17·0–43·3)	11·0 (5·0–15·5)	9·6 (3·6–17·9)	19·7 (9·1–26·1)
Serbia	All causes	3·7 (2·3–5·3)	1·7 (0·39–3·5)	1·6 (0·92–2·3)	1·4 (0·96–2·1)
Serbia	Diarrheal diseases	49·9 (22·8–73·6)	11·5 (6·0–17·0)	36·7 (-1·1–71·3)	13·0 (8·2–17·3)
Serbia	Lower respiratory infections	45·0 (34·2–61·5)	24·1 (0·87–53·6)	13·9 (8·6–19·2)	20·3 (14·0–27·1)
Serbia	Malaria	0 (0–0)	0 (0–0)	--	0 (0–0)
Serbia	Measles	29·4 (13·8–36·3)	7·7 (3·7–10·2)	8·3 (3·1–15·6)	16·8 (7·6–22·4)
Slovakia	All causes	10·1 (6·8–14·1)	4·6 (0·67–10·5)	4·3 (3·0–5·6)	3·7 (2·4–5·4)
Slovakia	Diarrheal diseases	50·5 (19·3–78·6)	9·9 (5·1–14·7)	38·3 (-1·0–76·6)	11·6 (7·2–15·5)
Slovakia	Lower respiratory infections	45·3 (34·1–63·2)	23·2 (0·69–56·6)	14·7 (9·0–20·3)	18·5 (11·7–25·3)
Slovakia	Malaria	0 (0–0)	0 (0–0)	--	0 (0–0)
Slovakia	Measles	0 (0–0)	0 (0–0)	0 (0–0)	0 (0–0)
Slovenia	All causes	7·2 (4·8–11·1)	3·3 (0·29–8·6)	2·8 (2·0–3·6)	2·6 (1·8–3·7)
Slovenia	Diarrheal diseases	43·3 (14·2–71·8)	7·0 (3·4–10·5)	33·9 (-0·88–70·3)	8·7 (5·3–11·7)
Slovenia	Lower respiratory infections	38·3 (28·5–55·2)	18·5 (0·48–47·8)	12·8 (8·0–17·6)	14·9 (10·1–20·2)
Slovenia	Malaria	0 (0–0)	0 (0–0)	--	0 (0–0)
Slovenia	Measles	0 (0–0)	0 (0–0)	0 (0–0)	0 (0–0)
Eastern Europe	All causes	4·8 (3·5–6·5)	2·3 (0·24–5·4)	1·6 (1·2–2·0)	2·3 (1·5–3·1)
Eastern Europe	Diarrheal diseases	51·2 (24·9–77·1)	11·1 (5·8–16·4)	35·5 (-0·92–74·1)	16·3 (10·7–21·4)
Eastern Europe	Lower respiratory infections	46·4 (35·7–62·2)	23·4 (0·71–55·4)	13·1 (8·0–18·0)	23·1 (15·1–30·6)
Eastern Europe	Malaria	0 (0–0)	0 (0–0)	--	0 (0–0)
Eastern Europe	Measles	25·5 (10·8–34·0)	8·1 (3·6–11·2)	6·8 (2·4–13·2)	13·4 (5·4–21·2)
Belarus	All causes	2·6 (1·6–4·1)	1·6 (0·20–3·6)	0·80 (0·56–1·1)	0·78 (0·49–1·2)
Belarus	Diarrheal diseases	42·5 (20·2–68·6)	12·2 (6·4–17·9)	29·2 (-0·69–66·0)	8·2 (4·8–11·1)
Belarus	Lower respiratory infections	40·4 (25·2–62·2)	25·5 (0·81–58·2)	10·1 (6·2–14·1)	13·0 (8·0–18·3)
Belarus	Malaria	0 (0–0)	0 (0–0)	--	0 (0–0)
Belarus	Measles	18·5 (8·3–24·1)	8·0 (3·6–11·2)	4·8 (1·7–9·7)	7·0 (3·1–9·8)
Estonia	All causes	4·6 (2·9–6·8)	2·3 (0·21–5·8)	1·4 (0·96–1·9)	1·8 (1·2–2·5)

Estonia	Diarrheal diseases	41·6 (17·1–69·9)	8·4 (4·3–12·7)	30·3 (-0·75–67·9)	9·1 (5·4–12·3)
Estonia	Lower respiratory infections	35·1 (25·1–52·3)	18·4 (0·53–46·0)	9·5 (5·7–13·1)	14·3 (10·8–19·2)
Estonia	Malaria	0 (0–0)	0 (0–0)	--	0 (0–0)
Estonia	Measles	24·3 (10·4–32·3)	7·0 (2·9–10·9)	8·8 (3·2–16·3)	10·9 (4·6–16·3)
Latvia	All causes	4·4 (2·9–6·6)	2·3 (0·16–5·5)	1·3 (0·82–1·8)	1·7 (1·2–2·4)
Latvia	Diarrheal diseases	43·8 (18·8–71·9)	9·5 (4·8–14·0)	31·3 (-0·80–69·1)	10·1 (6·2–13·4)
Latvia	Lower respiratory infections	35·3 (24·5–51·3)	19·1 (0·54–45·9)	9·3 (5·5–12·8)	14·6 (10·3–19·4)
Latvia	Malaria	0 (0–0)	0 (0–0)	--	0 (0–0)
Latvia	Measles	0 (0–0)	0 (0–0)	0 (0–0)	0 (0–0)
Lithuania	All causes	3·4 (2·1–5·3)	1·7 (0·20–3·9)	1·2 (0·77–1·5)	1·3 (0·87–1·8)
Lithuania	Diarrheal diseases	40·5 (16·8–67·9)	8·3 (4·2–12·2)	29·4 (-0·71–65·6)	9·0 (5·4–12·1)
Lithuania	Lower respiratory infections	32·4 (22·5–48·5)	17·1 (0·47–42·5)	8·7 (5·2–12·1)	13·2 (9·5–17·7)
Lithuania	Malaria	0 (0–0)	0 (0–0)	--	0 (0–0)
Lithuania	Measles	22·5 (10·0–29·3)	5·8 (2·7–7·9)	7·5 (2·8–14·1)	11·1 (4·9–15·1)
Republic of Moldova	All causes	5·5 (3·6–8·2)	2·9 (0·27–6·7)	1·8 (1·4–2·4)	2·0 (1·4–2·8)
Republic of Moldova	Diarrheal diseases	43·8 (18·2–70·6)	9·5 (4·8–14·1)	32·0 (-0·88–68·4)	9·3 (5·7–12·5)
Republic of Moldova	Lower respiratory infections	34·3 (23·8–50·7)	18·9 (0·60–45·5)	9·7 (5·8–13·7)	13·2 (8·9–18·1)
Republic of Moldova	Malaria	0 (0–0)	0 (0–0)	--	0 (0–0)
Republic of Moldova	Measles	22·8 (10·4–29·1)	6·4 (2·9–8·5)	7·6 (2·9–13·9)	10·9 (4·9–14·7)
Russian Federation	All causes	5·4 (3·9–7·3)	2·5 (0·26–6·0)	1·8 (1·4–2·4)	2·6 (1·7–3·6)
Russian Federation	Diarrheal diseases	52·1 (25·1–78·6)	10·8 (5·6–16·0)	36·4 (-0·95–75·6)	16·8 (11·0–22·0)
Russian Federation	Lower respiratory infections	48·8 (38·1–64·7)	24·1 (0·71–57·5)	14·3 (8·7–19·6)	24·6 (15·9–32·6)
Russian Federation	Malaria	0 (0–0)	0 (0–0)	--	0 (0–0)
Russian Federation	Measles	35·8 (16·5–45·5)	7·9 (3·4–10·9)	12·1 (4·2–23·0)	21·0 (9·5–28·1)
Ukraine	All causes	2·9 (2·1–4·0)	1·5 (0·17–3·3)	0·75 (0·56–0·99)	1·5 (1·0–2·0)
Ukraine	Diarrheal diseases	50·2 (28·3–72·0)	14·6 (8·0–20·7)	31·0 (-0·76–67·8)	17·7 (11·9–22·8)
Ukraine	Lower respiratory infections	39·7 (30·1–52·8)	21·4 (0·73–46·0)	8·7 (5·2–12·2)	20·9 (14·0–27·2)
Ukraine	Malaria	0 (0–0)	0 (0–0)	--	0 (0–0)
Ukraine	Measles	36·8 (17·2–45·4)	8·7 (3·3–12·9)	8·4 (3·1–15·7)	24·6 (11·0–31·8)

**High-income**	**All causes**	**1·2 (0·82–1·9)**	**0·63 (0·22–1·4)**	**0·53 (0·39–0·69)**	**0·38 (0·27–0·52)**
**High-income**	**Diarrheal diseases**	**32·7 (12·1–60·1)**	**6·0 (3·0–9·0)**	**23·9 (-0·52–58·1)**	**6·4 (3·8–8·4)**
**High-income**	**Lower respiratory infections**	**34·8 (24·6–54·8)**	**17·7 (0·45–47·4)**	**8·8 (5·2–12·3)**	**13·7 (10·1–17·9)**
**High-income**	**Malaria**	**4·2 (-1·8–13·4)**	**3·8 (-1·5–11·7)**	**--**	**0·48 (-0·21–2·0)**
**High-income**	**Measles**	**14·2 (6·2–18·4)**	**4·0 (1·8–5·4)**	**5·5 (2·0–10·3)**	**5·5 (2·4–7·6)**
Australasia	All causes	0·57 (0·37–0·96)	0·20 (<0.1–0·56)	0·28 (0·18–0·44)	0·17 (0·12–0·25)
Australasia	Diarrheal diseases	30·0 (6·9–65·1)	2·4 (1·2–3·9)	24·6 (-0·56–63·6)	4·7 (2·7–6·6)
Australasia	Lower respiratory infections	19·7 (15·5–31·4)	7·5 (0·15–25·0)	6·5 (3·8–9·2)	7·6 (5·1–10·7)
Australasia	Malaria	0 (0–0)	0 (0–0)	--	0 (0–0)
Australasia	Measles	9·5 (4·2–13·3)	1·2 (0·57–1·6)	4·5 (1·7–8·6)	4·1 (1·9–5·7)
Australia	All causes	0·47 (0·31–0·80)	0·15 (<0.1–0·43)	0·26 (0·17–0·40)	0·14 (<0.1–0·20)
Australia	Diarrheal diseases	28·9 (5·6–64·5)	1·7 (0·80–2·8)	24·4 (-0·54–63·2)	4·1 (2·3–5·7)
Australia	Lower respiratory infections	18·2 (14·9–27·9)	5·9 (0·11–21·0)	6·6 (3·9–9·4)	7·1 (5·1–9·7)
Australia	Malaria	0 (0–0)	0 (0–0)	--	0 (0–0)
Australia	Measles	9·4 (4·1–13·1)	1·1 (0·53–1·5)	4·5 (1·7–8·6)	4·1 (1·8–5·5)
New Zealand	All causes	0·98 (0·61–1·7)	0·39 (<0.1–1·1)	0·38 (0·23–0·60)	0·33 (0·20–0·49)
New Zealand	Diarrheal diseases	32·9 (10·4–66·4)	4·2 (2·0–6·4)	25·1 (-0·59–64·4)	6·3 (3·7–8·6)
New Zealand	Lower respiratory infections	23·1 (16·7–38·9)	10·9 (0·24–34·1)	6·2 (3·7–8·8)	8·8 (5·1–12·8)
New Zealand	Malaria	0 (0–0)	0 (0–0)	--	0 (0–0)
New Zealand	Measles	11·8 (5·2–15·5)	2·2 (1·0–3·1)	4·6 (1·7–8·8)	5·6 (2·5–7·7)
High-income Asia Pacific	All causes	1·8 (1·1–2·8)	0·91 (0·20–2·1)	0·71 (0·48–0·97)	0·52 (0·38–0·72)
High-income Asia Pacific	Diarrheal diseases	37·9 (14·5–66·2)	8·0 (4·1–12·1)	28·2 (-0·71–64·4)	6·5 (3·9–8·8)
High-income Asia Pacific	Lower respiratory infections	40·1 (27·0–63·0)	22·7 (0·62–56·7)	10·7 (6·3–14·8)	14·0 (10·6–18·5)
High-income Asia Pacific	Malaria	4·2 (-1·8–13·4)	3·8 (-1·5–11·7)	--	0·48 (-0·21–2·0)
High-income Asia Pacific	Measles	22·1 (10·0–29·3)	7·5 (3·4–10·2)	8·4 (3·0–15·7)	8·2 (3·7–11·2)
Brunei Darussalam	All causes	2·0 (1·4–3·0)	0·98 (0·11–2·2)	0·56 (0·40–0·77)	1·1 (0·71–1·7)
Brunei Darussalam	Diarrheal diseases	47·5 (26·8–67·9)	11·1 (5·8–16·4)	29·4 (-0·75–64·1)	19·6 (13·7–24·9)
Brunei Darussalam	Lower respiratory infections	51·5 (40·7–65·3)	26·3 (0·86–57·5)	11·5 (6·9–15·9)	29·5 (19·8–36·9)
Brunei Darussalam	Malaria	0 (0–0)	0 (0–0)	--	0 (0–0)

Brunei Darussalam	Measles	0 (0–0)	0 (0–0)	0 (0–0)	0 (0–0)
Japan	All causes	1·9 (1·2–3·0)	0·98 (0·22–2·2)	0·75 (0·51–1·0)	0·58 (0·40–0·80)
Japan	Diarrheal diseases	38·8 (15·3–67·2)	8·2 (4·1–12·5)	28·4 (-0·72–65·5)	7·3 (4·3–9·9)
Japan	Lower respiratory infections	40·8 (27·6–64·0)	23·1 (0·63–58·0)	10·6 (6·2–14·6)	14·6 (10·7–19·3)
Japan	Malaria	0 (0–0)	0 (0–0)	--	0 (0–0)
Japan	Measles	22·1 (10·0–29·2)	7·5 (3·4–10·1)	8·3 (3·0–15·6)	8·3 (3·7–11·2)
Republic of Korea	All causes	1·1 (0·59–1·9)	0·53 (0·13–1·2)	0·54 (0·29–0·89)	0·24 (0·17–0·33)
Republic of Korea	Diarrheal diseases	34·6 (11·5–64·6)	7·0 (3·6–10·9)	27·0 (-0·69–63·1)	3·9 (2·2–5·5)
Republic of Korea	Lower respiratory infections	34·2 (21·5–57·1)	19·6 (0·53–51·3)	9·9 (5·7–14·2)	9·4 (7·4–12·0)
Republic of Korea	Malaria	4·2 (-1·8–13·4)	3·8 (-1·5–11·7)	--	0·48 (-0·21–2·0)
Republic of Korea	Measles	16·5 (6·3–23·3)	5·2 (2·1–7·1)	7·8 (2·6–14·8)	4·6 (1·7–6·3)
Singapore	All causes	3·8 (2·1–6·3)	2·1 (0·18–5·1)	1·3 (0·87–1·8)	1·1 (0·66–1·8)
Singapore	Diarrheal diseases	42·2 (16·6–68·6)	10·1 (5·4–15·3)	31·4 (-0·82–67·0)	7·3 (4·2–9·9)
Singapore	Lower respiratory infections	42·8 (28·7–63·8)	25·0 (0·69–58·7)	13·0 (8·0–17·5)	13·7 (8·7–19·0)
Singapore	Malaria	0 (0–0)	0 (0–0)	--	0 (0–0)
Singapore	Measles	26·3 (11·6–34·7)	8·7 (3·8–12·2)	11·2 (4·1–21·2)	9·4 (4·2–12·7)
High-income North America	All causes	1·1 (0·73–1·7)	0·55 (0·17–1·2)	0·49 (0·39–0·58)	0·31 (0·23–0·43)
High-income North America	Diarrheal diseases	33·9 (10·7–62·1)	5·5 (2·7–8·3)	26·3 (-0·57–60·8)	5·6 (3·3–7·6)
High-income North America	Lower respiratory infections	37·3 (27·1–58·5)	18·3 (0·46–50·1)	11·2 (6·5–15·5)	13·5 (9·8–17·8)
High-income North America	Malaria	0 (0–0)	0 (0–0)	--	0 (0–0)
High-income North America	Measles	18·0 (7·8–25·0)	4·4 (2·0–6·0)	8·3 (3·0–15·6)	6·5 (2·9–9·0)
Canada	All causes	0·87 (0·42–1·6)	0·43 (0·13–1·0)	0·48 (0·26–0·79)	<0.1 (<0.1–0·13)
Canada	Diarrheal diseases	31·1 (7·8–62·2)	5·9 (3·0–9·0)	25·4 (-0·51–61·0)	2·0 (1·1–2·8)
Canada	Lower respiratory infections	27·0 (15·2–51·0)	16·3 (0·38–46·7)	9·1 (5·3–12·7)	4·0 (3·2–5·4)
Canada	Malaria	0 (0–0)	0 (0–0)	--	0 (0–0)
Canada	Measles	11·1 (4·5–17·4)	2·7 (1·2–3·8)	7·0 (2·6–13·1)	1·7 (0·71–2·4)
Greenland	All causes	2·2 (1·2–3·7)	1·1 (0·26–2·7)	0·80 (0·54–1·1)	0·70 (0·47–1·0)
Greenland	Diarrheal diseases	39·1 (15·9–69·2)	8·6 (4·3–12·8)	27·8 (-0·55–66·9)	7·8 (4·6–10·7)
Greenland	Lower respiratory infections	40·5 (27·1–62·8)	22·4 (0·62–55·9)	10·0 (5·9–14·1)	14·6 (10·2–19·4)

Greenland	Malaria	0 (0–0)	0 (0–0)	--	0 (0–0)
Greenland	Measles	18·9 (8·5–25·7)	4·9 (2·3–6·7)	8·0 (2·9–15·2)	7·3 (3·3–9·9)
United States of America	All causes	1·1 (0·75–1·7)	0·56 (0·18–1·3)	0·49 (0·39–0·58)	0·33 (0·24–0·45)
United States of America	Diarrheal diseases	34·4 (11·2–62·2)	5·5 (2·7–8·2)	26·5 (-0·58–60·9)	6·2 (3·7–8·4)
United States of America	Lower respiratory infections	37·9 (27·9–59·0)	18·4 (0·46–50·4)	11·3 (6·6–15·7)	14·2 (10·2–18·7)
United States of America	Malaria	0 (0–0)	0 (0–0)	--	0 (0–0)
United States of America	Measles	19·6 (8·6–28·1)	3·8 (1·7–5·1)	10·2 (3·7–19·2)	7·1 (3·2–9·5)
Southern Latin America	All causes	2·7 (2·0–4·0)	1·5 (0·63–3·0)	1·1 (0·84–1·3)	1·1 (0·77–1·5)
Southern Latin America	Diarrheal diseases	33·5 (16·8–57·7)	7·0 (3·5–10·7)	21·0 (-0·46–54·3)	10·2 (6·5–13·1)
Southern Latin America	Lower respiratory infections	37·5 (27·2–54·8)	18·8 (0·52–46·6)	6·5 (3·7–9·1)	19·0 (14·3–24·2)
Southern Latin America	Malaria	0 (0–0)	0 (0–0)	--	0 (0–0)
Southern Latin America	Measles	13·8 (6·3–17·3)	5·8 (2·7–7·7)	4·2 (1·6–7·9)	4·5 (2·0–6·2)
Argentina	All causes	2·9 (2·0–4·2)	1·5 (0·53–3·1)	1·0 (0·82–1·2)	1·2 (0·89–1·7)
Argentina	Diarrheal diseases	34·5 (17·5–58·4)	6·5 (3·3–9·9)	21·2 (-0·47–54·8)	11·8 (7·7–15·4)
Argentina	Lower respiratory infections	39·4 (29·3–56·0)	19·0 (0·53–47·1)	6·8 (3·9–9·6)	21·0 (15·6–26·7)
Argentina	Malaria	0 (0–0)	0 (0–0)	--	0 (0–0)
Argentina	Measles	0 (0–0)	0 (0–0)	0 (0–0)	0 (0–0)
Chile	All causes	2·2 (1·5–3·4)	1·5 (0·85–2·6)	1·2 (0·90–1·6)	0·40 (0·31–0·53)
Chile	Diarrheal diseases	29·5 (13·1–55·5)	7·6 (3·9–11·6)	20·2 (-0·43–53·2)	4·5 (2·6–6·4)
Chile	Lower respiratory infections	27·3 (15·3–47·6)	17·2 (0·47–42·6)	5·0 (2·8–7·3)	8·6 (7·1–11·1)
Chile	Malaria	0 (0–0)	0 (0–0)	--	0 (0–0)
Chile	Measles	13·8 (6·3–17·3)	5·8 (2·7–7·7)	4·2 (1·6–7·9)	4·5 (2·0–6·2)
Uruguay	All causes	3·0 (2·2–4·3)	1·7 (0·81–3·1)	1·2 (0·77–1·8)	1·1 (0·75–1·5)
Uruguay	Diarrheal diseases	37·9 (22·2–59·5)	10·4 (5·3–15·4)	21·2 (-0·45–54·7)	13·1 (8·4–17·0)
Uruguay	Lower respiratory infections	42·5 (29·9–62·2)	24·3 (0·73–57·1)	6·6 (3·8–9·3)	20·7 (13·7–27·2)
Uruguay	Malaria	0 (0–0)	0 (0–0)	--	0 (0–0)
Uruguay	Measles	0 (0–0)	0 (0–0)	0 (0–0)	0 (0–0)
Western Europe	All causes	0·78 (0·46–1·4)	0·38 (0·13–0·88)	0·35 (0·20–0·61)	0·19 (0·13–0·27)
Western Europe	Diarrheal diseases	30·0 (10·3–58·4)	5·6 (2·7–8·4)	22·3 (-0·46–56·4)	4·8 (2·8–6·4)

Western Europe	Lower respiratory infections	26·4 (16·8–46·9)	14·6 (0·34–41·9)	6·3 (3·7–8·9)	8·5 (5·8–11·6)
Western Europe	Malaria	0 (0–0)	0 (0–0)	--	0 (0–0)
Western Europe	Measles	10·9 (4·8–13·6)	3·3 (1·5–4·5)	3·6 (1·3–6·7)	4·5 (2·0–6·2)
Andorra	All causes	0·70 (0·37–1·4)	0·37 (<0.1–1·1)	0·20 (0·13–0·31)	0·22 (0·13–0·37)
Andorra	Diarrheal diseases	28·9 (9·5–57·6)	5·0 (2·5–7·6)	21·7 (-0·45–55·8)	4·6 (2·7–6·2)
Andorra	Lower respiratory infections	26·3 (17·1–46·8)	14·2 (0·32–41·3)	6·3 (3·7–9·0)	8·6 (6·1–11·6)
Andorra	Malaria	0 (0–0)	0 (0–0)	--	0 (0–0)
Andorra	Measles	0 (0–0)	0 (0–0)	0 (0–0)	0 (0–0)
Austria	All causes	0·33 (0·18–0·60)	0·14 (<0.1–0·34)	0·14 (<0.1–0·27)	<0.1 (<0.1–0·14)
Austria	Diarrheal diseases	30·1 (10·5–59·4)	5·4 (2·7–8·2)	22·3 (-0·46–57·5)	5·1 (2·9–6·9)
Austria	Lower respiratory infections	27·2 (17·6–47·0)	14·4 (0·34–40·2)	6·2 (3·5–8·7)	9·7 (8·0–12·5)
Austria	Malaria	0 (0–0)	0 (0–0)	--	0 (0–0)
Austria	Measles	8·9 (4·0–11·4)	2·3 (1·1–3·2)	2·8 (1·1–5·4)	4·1 (1·8–5·6)
Belgium	All causes	1·3 (0·68–2·2)	0·53 (0·17–1·3)	0·58 (0·24–1·1)	0·32 (0·21–0·46)
Belgium	Diarrheal diseases	29·8 (10·3–58·6)	5·4 (2·6–8·1)	22·2 (-0·46–56·5)	4·9 (2·8–6·7)
Belgium	Lower respiratory infections	28·6 (18·6–50·6)	15·7 (0·37–45·4)	6·9 (4·1–9·6)	9·4 (6·4–13·0)
Belgium	Malaria	0 (0–0)	0 (0–0)	--	0 (0–0)
Belgium	Measles	11·2 (4·9–14·5)	3·2 (1·4–4·4)	3·7 (1·4–7·1)	4·7 (2·1–6·5)
Cyprus	All causes	1·1 (0·45–2·3)	0·40 (0·15–0·89)	0·58 (0·12–1·5)	0·26 (0·14–0·43)
Cyprus	Diarrheal diseases	35·9 (13·7–65·7)	7·6 (3·9–11·6)	25·9 (-0·55–63·4)	6·5 (3·9–9·0)
Cyprus	Lower respiratory infections	34·9 (21·9–58·5)	20·0 (0·52–52·7)	8·5 (5·0–12·0)	11·5 (6·9–16·2)
Cyprus	Malaria	0 (0–0)	0 (0–0)	--	0 (0–0)
Cyprus	Measles	0 (0–0)	0 (0–0)	0 (0–0)	0 (0–0)
Denmark	All causes	1·3 (0·67–2·3)	0·53 (0·19–1·2)	0·67 (0·23–1·4)	0·31 (0·18–0·47)
Denmark	Diarrheal diseases	33·7 (11·7–63·9)	6·4 (3·2–9·6)	24·9 (-0·51–61·9)	5·7 (3·3–7·7)
Denmark	Lower respiratory infections	33·3 (21·1–56·5)	18·9 (0·47–51·8)	8·5 (5·1–11·9)	10·2 (5·3–15·0)
Denmark	Malaria	0 (0–0)	0 (0–0)	--	0 (0–0)
Denmark	Measles	0 (0–0)	0 (0–0)	0 (0–0)	0 (0–0)
Finland	All causes	0·77 (0·42–1·3)	0·37 (<0.1–0·94)	0·28 (0·16–0·44)	0·22 (0·15–0·33)

Finland	Diarrheal diseases	31·8 (11·4–60·5)	6·1 (3·0–9·1)	23·5 (-0·50–58·7)	5·4 (3·1–7·2)
Finland	Lower respiratory infections	30·0 (19·5–51·6)	16·6 (0·41–45·8)	7·2 (4·2–10·0)	10·1 (7·2–13·5)
Finland	Malaria	0 (0–0)	0 (0–0)	--	0 (0–0)
Finland	Measles	0 (0–0)	0 (0–0)	0 (0–0)	0 (0–0)
France	All causes	0·91 (0·59–1·5)	0·53 (0·30–0·99)	0·51 (0·35–0·77)	0·18 (0·12–0·26)
France	Diarrheal diseases	28·2 (9·6–55·2)	5·0 (2·5–7·7)	21·1 (-0·44–53·4)	4·6 (2·7–6·4)
France	Lower respiratory infections	26·7 (17·1–46·2)	14·5 (0·34–40·9)	6·4 (3·7–9·1)	8·8 (6·1–12·0)
France	Malaria	0 (0–0)	0 (0–0)	--	0 (0–0)
France	Measles	10·7 (4·8–13·4)	3·2 (1·5–4·3)	3·4 (1·3–6·4)	4·5 (2·0–6·2)
Germany	All causes	0·57 (0·27–1·0)	0·22 (<0.1–0·48)	0·31 (<0.1–0·65)	0·11 (<0.1–0·17)
Germany	Diarrheal diseases	32·9 (11·6–61·9)	6·6 (3·3–9·9)	24·8 (-0·54–60·2)	4·8 (2·8–6·6)
Germany	Lower respiratory infections	29·8 (18·1–51·6)	17·4 (0·43–47·4)	7·7 (4·5–10·8)	8·5 (5·4–12·0)
Germany	Malaria	0 (0–0)	0 (0–0)	--	0 (0–0)
Germany	Measles	11·1 (5·0–14·4)	3·9 (1·8–5·3)	3·9 (1·4–7·5)	3·9 (1·7–5·3)
Greece	All causes	0·85 (0·51–1·5)	0·45 (<0.1–1·3)	0·23 (0·16–0·32)	0·28 (0·16–0·42)
Greece	Diarrheal diseases	32·8 (11·9–61·9)	6·2 (3·0–9·3)	23·8 (-0·47–59·6)	6·1 (3·5–8·2)
Greece	Lower respiratory infections	32·3 (20·8–54·8)	17·9 (0·43–49·9)	7·7 (4·6–10·8)	10·8 (6·0–15·4)
Greece	Malaria	0 (0–0)	0 (0–0)	--	0 (0–0)
Greece	Measles	0 (0–0)	0 (0–0)	0 (0–0)	0 (0–0)
Iceland	All causes	0·98 (0·53–1·7)	0·47 (<0.1–1·2)	0·38 (0·23–0·60)	0·26 (0·17–0·39)
Iceland	Diarrheal diseases	31·2 (10·4–61·2)	5·5 (2·7–8·3)	23·4 (-0·51–59·1)	5·0 (2·8–7·0)
Iceland	Lower respiratory infections	28·5 (18·5–50·8)	15·6 (0·37–45·9)	7·2 (4·2–10·1)	9·0 (5·6–12·8)
Iceland	Malaria	0 (0–0)	0 (0–0)	--	0 (0–0)
Iceland	Measles	0 (0–0)	0 (0–0)	0 (0–0)	0 (0–0)
Ireland	All causes	0·93 (0·53–1·7)	0·47 (<0.1–1·4)	0·31 (0·22–0·42)	0·26 (0·16–0·39)
Ireland	Diarrheal diseases	29·3 (9·3–58·1)	4·9 (2·4–7·4)	22·2 (-0·44–56·6)	4·6 (2·7–6·3)
Ireland	Lower respiratory infections	25·9 (16·2–46·7)	14·2 (0·31–42·7)	6·6 (3·9–9·4)	8·0 (4·6–11·5)
Ireland	Malaria	0 (0–0)	0 (0–0)	--	0 (0–0)
Ireland	Measles	0 (0–0)	0 (0–0)	0 (0–0)	0 (0–0)

Israel	All causes	1·2 (0·61–2·2)	0·47 (0·17–1·1)	0·61 (0·21–1·2)	0·29 (0·18–0·42)
Israel	Diarrheal diseases	30·3 (10·4–59·8)	5·4 (2·6–8·1)	22·5 (-0·45–58·1)	5·1 (2·9–7·0)
Israel	Lower respiratory infections	29·1 (18·7–52·3)	15·9 (0·36–47·2)	6·9 (4·1–9·7)	9·5 (6·0–13·2)
Israel	Malaria	0 (0–0)	0 (0–0)	--	0 (0–0)
Israel	Measles	9·5 (4·2–12·0)	2·5 (1·1–3·4)	3·1 (1·1–5·8)	4·3 (1·9–5·8)
Italy	All causes	0·98 (0·57–1·7)	0·48 (0·10–1·3)	0·38 (0·24–0·57)	0·27 (0·17–0·40)
Italy	Diarrheal diseases	29·7 (9·9–58·1)	5·2 (2·6–7·9)	22·1 (-0·43–56·0)	4·9 (2·8–6·7)
Italy	Lower respiratory infections	27·8 (17·9–48·7)	15·2 (0·35–43·6)	6·7 (4·0–9·4)	9·1 (5·9–12·7)
Italy	Malaria	0 (0–0)	0 (0–0)	--	0 (0–0)
Italy	Measles	10·5 (4·7–13·4)	3·1 (1·4–4·2)	3·4 (1·3–6·4)	4·4 (2·0–5·9)
Luxembourg	All causes	0·77 (0·41–1·4)	0·34 (0·15–0·72)	0·41 (0·17–0·80)	0·17 (0·11–0·25)
Luxembourg	Diarrheal diseases	30·4 (10·8–57·9)	5·8 (2·8–8·8)	22·5 (-0·49–56·0)	5·0 (2·8–6·9)
Luxembourg	Lower respiratory infections	27·1 (17·2–47·6)	15·4 (0·38–43·3)	6·7 (3·8–9·6)	8·6 (5·4–12·2)
Luxembourg	Malaria	0 (0–0)	0 (0–0)	--	0 (0–0)
Luxembourg	Measles	0 (0–0)	0 (0–0)	0 (0–0)	0 (0–0)
Malta	All causes	0·75 (0·44–1·3)	0·40 (<0.1–1·1)	0·22 (0·16–0·28)	0·23 (0·14–0·33)
Malta	Diarrheal diseases	29·9 (11·1–57·0)	5·9 (2·9–8·8)	22·0 (-0·46–55·5)	5·1 (2·9–7·1)
Malta	Lower respiratory infections	28·4 (17·9–48·9)	16·1 (0·40–44·2)	7·0 (4·1–9·8)	9·1 (5·5–13·0)
Malta	Malaria	0 (0–0)	0 (0–0)	--	0 (0–0)
Malta	Measles	0 (0–0)	0 (0–0)	0 (0–0)	0 (0–0)
Monaco	All causes	0·81 (0·40–1·7)	0·43 (<0.1–1·4)	0·23 (0·14–0·36)	0·24 (0·13–0·44)
Monaco	Diarrheal diseases	29·5 (9·4–59·9)	4·8 (2·3–7·4)	22·3 (-0·43–58·0)	4·8 (2·8–6·8)
Monaco	Lower respiratory infections	27·1 (17·6–49·9)	14·5 (0·32–45·3)	6·8 (4·0–9·6)	8·6 (5·1–12·2)
Monaco	Malaria	0 (0–0)	0 (0–0)	--	0 (0–0)
Monaco	Measles	0 (0–0)	0 (0–0)	0 (0–0)	0 (0–0)
Netherlands	All causes	0·66 (0·32–1·2)	0·40 (<0.1–0·94)	0·19 (0·11–0·34)	0·15 (0·10–0·22)
Netherlands	Diarrheal diseases	23·7 (11·2–43·0)	7·2 (3·6–10·8)	14·5 (-0·30–39·6)	3·8 (2·2–5·3)
Netherlands	Lower respiratory infections	26·6 (13·2–50·6)	18·0 (0·48–47·2)	3·8 (2·1–5·5)	7·2 (5·0–10·0)
Netherlands	Malaria	0 (0–0)	0 (0–0)	--	0 (0–0)

Netherlands	Measles	8·0 (3·4–10·7)	2·8 (1·2–4·1)	2·4 (0·84–4·9)	3·1 (1·4–4·3)
Norway	All causes	0·55 (0·30–0·99)	0·27 (<0.1–0·65)	0·25 (0·14–0·43)	0·14 (<0.1–0·21)
Norway	Diarrheal diseases	26·6 (9·0–55·7)	4·2 (2·0–6·6)	20·2 (-0·47–53·9)	4·1 (2·3–5·8)
Norway	Lower respiratory infections	23·1 (14·9–42·4)	12·5 (0·28–37·7)	5·7 (3·4–8·0)	7·4 (5·0–10·1)
Norway	Malaria	0 (0–0)	0 (0–0)	--	0 (0–0)
Norway	Measles	11·2 (4·8–14·5)	3·4 (1·5–4·6)	3·7 (1·3–7·4)	4·5 (2·0–6·2)
Portugal	All causes	1·2 (0·71–2·0)	0·62 (0·12–1·5)	0·40 (0·28–0·56)	0·35 (0·24–0·51)
Portugal	Diarrheal diseases	31·5 (12·1–59·2)	6·3 (3·1–9·5)	22·8 (-0·48–57·2)	5·6 (3·3–7·6)
Portugal	Lower respiratory infections	33·1 (21·0–56·7)	18·6 (0·47–51·0)	7·7 (4·6–10·8)	11·1 (7·6–15·1)
Portugal	Malaria	0 (0–0)	0 (0–0)	--	0 (0–0)
Portugal	Measles	12·7 (5·8–16·0)	4·2 (1·9–5·6)	3·8 (1·4–7·2)	5·4 (2·4–7·3)
San Marino	All causes	0·56 (0·27–1·1)	0·30 (<0.1–0·88)	0·17 (0·10–0·26)	0·17 (<0.1–0·32)
San Marino	Diarrheal diseases	30·1 (10·0–59·3)	5·2 (2·6–8·1)	22·6 (-0·46–57·4)	4·9 (2·9–6·9)
San Marino	Lower respiratory infections	25·9 (16·0–45·3)	14·2 (0·31–40·8)	6·4 (3·8–9·2)	8·3 (5·0–12·0)
San Marino	Malaria	0 (0–0)	0 (0–0)	--	0 (0–0)
San Marino	Measles	0 (0–0)	0 (0–0)	0 (0–0)	0 (0–0)
Spain	All causes	0·89 (0·50–1·6)	0·41 (<0.1–1·1)	0·37 (0·19–0·65)	0·23 (0·15–0·34)
Spain	Diarrheal diseases	31·8 (10·9–61·6)	5·9 (2·9–9·0)	23·6 (-0·48–59·5)	5·3 (3·0–7·3)
Spain	Lower respiratory infections	30·5 (19·7–53·5)	17·0 (0·40–48·4)	7·5 (4·3–10·4)	9·8 (6·0–14·0)
Spain	Malaria	0 (0–0)	0 (0–0)	--	0 (0–0)
Spain	Measles	10·9 (4·9–14·2)	3·3 (1·5–4·4)	3·6 (1·4–6·7)	4·6 (2·1–6·3)
Sweden	All causes	0·96 (0·48–1·7)	0·40 (0·13–0·94)	0·47 (0·19–0·95)	0·23 (0·16–0·33)
Sweden	Diarrheal diseases	29·3 (9·7–58·3)	5·1 (2·5–7·7)	22·1 (-0·47–56·4)	4·6 (2·7–6·3)
Sweden	Lower respiratory infections	27·7 (17·7–50·1)	15·1 (0·34–44·4)	6·8 (3·9–9·4)	9·0 (6·3–12·2)
Sweden	Malaria	0 (0–0)	0 (0–0)	--	0 (0–0)
Sweden	Measles	9·8 (4·3–12·6)	2·8 (1·3–3·8)	3·3 (1·2–6·1)	4·1 (1·9–5·7)
Switzerland	All causes	0·58 (0·31–1·0)	0·26 (<0.1–0·62)	0·29 (0·13–0·54)	0·14 (<0.1–0·20)
Switzerland	Diarrheal diseases	29·0 (9·6–57·7)	5·0 (2·5–7·6)	21·9 (-0·46–56·0)	4·6 (2·7–6·2)
Switzerland	Lower respiratory infections	25·7 (16·3–46·3)	14·0 (0·32–41·5)	6·4 (3·7–8·9)	8·2 (5·5–11·2)

Switzerland	Malaria	0 (0–0)	0 (0–0)	--	0 (0–0)
Switzerland	Measles	9·3 (4·1–12·1)	2·6 (1·2–3·5)	3·1 (1·2–6·2)	3·9 (1·8–5·4)
United Kingdom	All causes	0·58 (0·33–1·1)	0·27 (<0.1–0·76)	0·20 (0·14–0·30)	0·17 (0·12–0·24)
United Kingdom	Diarrheal diseases	25·7 (7·3–55·6)	3·5 (1·6–5·4)	19·9 (-0·40–53·9)	3·7 (2·1–5·1)
United Kingdom	Lower respiratory infections	20·7 (13·4–38·5)	10·7 (0·24–33·3)	5·0 (2·9–7·0)	6·9 (5·4–9·2)
United Kingdom	Malaria	0 (0–0)	0 (0–0)	--	0 (0–0)
United Kingdom	Measles	11·4 (4·9–14·6)	3·0 (1·4–4·1)	4·1 (1·4–8·0)	4·8 (2·1–6·7)
**Latin America and Caribbean**	**All causes**	**10·3 (7·8–12·9)**	**6·4 (3·0–10·0)**	**4·4 (3·1–5·5)**	**4·0 (2·9–5·1)**
**Latin America and Caribbean**	**Diarrheal diseases**	**56·8 (38·6–75·5)**	**20·7 (11·9–29·7)**	**32·6 (-1·1–69·0)**	**20·3 (13·7–26·0)**
**Latin America and Caribbean**	**Lower respiratory infections**	**52·4 (38·9–68·9)**	**31·5 (1·3–63·4)**	**10·0 (6·0–13·6)**	**27·0 (19·0–33·7)**
**Latin America and Caribbean**	**Malaria**	**14·4 (-7·1–45·4)**	**10·3 (-5·0–29·4)**	**--**	**5·6 (-2·4–24·8)**
**Latin America and Caribbean**	**Measles**	**27·3 (12·9–34·5)**	**8·5 (4·0–11·2)**	**6·5 (2·5–12·3)**	**15·3 (6·9–21·6)**
Andean Latin America	All causes	10·7 (8·0–13·8)	6·9 (2·5–11·8)	3·9 (3·4–4·4)	4·3 (3·0–5·6)
Andean Latin America	Diarrheal diseases	47·7 (33·1–68·3)	14·9 (8·1–22·4)	25·3 (-0·72–60·7)	17·4 (11·1–23·2)
Andean Latin America	Lower respiratory infections	55·0 (40·2–74·4)	32·0 (1·1–67·3)	9·2 (5·5–12·6)	28·4 (20·2–35·1)
Andean Latin America	Malaria	11·9 (-5·2–39·6)	7·9 (-3·5–23·3)	--	4·9 (-1·9–21·4)
Andean Latin America	Measles	37·8 (18·5–45·7)	12·9 (6·1–17·4)	8·2 (3·0–15·6)	22·5 (10·5–29·2)
Bolivia (Plurinational State of)	All causes	13·1 (8·7–18·7)	8·3 (2·6–15·7)	4·8 (3·6–6·0)	5·2 (3·1–7·8)
Bolivia (Plurinational State of)	Diarrheal diseases	51·0 (32·1–75·0)	15·1 (8·2–22·9)	29·3 (-0·88–69·8)	18·0 (11·9–23·9)
Bolivia (Plurinational State of)	Lower respiratory infections	52·2 (38·6–70·4)	30·3 (1·1–64·5)	9·7 (5·8–13·4)	26·3 (16·3–34·0)
Bolivia (Plurinational State of)	Malaria	12·0 (-5·3–40·6)	7·9 (-3·5–23·7)	--	5·1 (-2·0–22·4)
Bolivia (Plurinational State of)	Measles	0 (0–0)	0 (0–0)	0 (0–0)	0 (0–0)
Ecuador	All causes	10·2 (7·8–12·9)	6·6 (2·4–11·0)	4·4 (3·8–5·1)	3·7 (2·6–5·0)
Ecuador	Diarrheal diseases	56·0 (32·3–78·8)	16·6 (9·0–24·4)	36·3 (-1·2–74·9)	16·6 (10·7–21·8)
Ecuador	Lower respiratory infections	59·4 (44·1–77·6)	35·0 (1·3–71·3)	15·2 (9·7–20·2)	27·6 (19·0–35·4)
Ecuador	Malaria	13·2 (-6·1–44·7)	8·7 (-3·9–25·7)	--	5·6 (-2·1–25·6)
Ecuador	Measles	37·8 (18·5–45·7)	12·9 (6·1–17·4)	8·2 (3·0–15·6)	22·5 (10·5–29·2)
Peru	All causes	9·8 (7·3–13·2)	6·2 (2·6–10·8)	3·0 (2·4–3·7)	4·2 (3·0–5·6)
Peru	Diarrheal diseases	41·7 (32·5–59·4)	13·9 (7·4–21·8)	17·4 (-0·45–49·8)	17·5 (11·0–23·9)

Peru	Lower respiratory infections	54·5 (38·6–76·5)	31·3 (1·0–68·0)	4·6 (2·5–6·7)	30·6 (24·0–36·6)
Peru	Malaria	11·7 (-5·2–38·7)	7·8 (-3·4–23·0)	--	4·7 (-1·8–20·6)
Peru	Measles	0 (0–0)	0 (0–0)	0 (0–0)	0 (0–0)
Caribbean	All causes	15·8 (10·8–20·2)	10·1 (5·3–14·3)	8·8 (5·2–11·9)	5·1 (3·5–7·1)
Caribbean	Diarrheal diseases	66·3 (43·7–83·7)	27·3 (16·6–37·9)	44·3 (-1·8–80·0)	19·4 (12·9–25·0)
Caribbean	Lower respiratory infections	50·8 (40·0–62·5)	33·2 (1·7–58·1)	15·7 (9·7–21·0)	24·1 (17·6–30·3)
Caribbean	Malaria	19·6 (-10·5–57·3)	14·2 (-7·3–38·4)	--	7·9 (-3·3–32·8)
Caribbean	Measles	22·7 (10·1–29·9)	7·1 (3·2–9·7)	8·5 (3·2–15·6)	9·1 (4·0–12·5)
Antigua and Barbuda	All causes	8·5 (6·1–11·5)	5·5 (2·3–10·0)	4·3 (3·5–5·3)	2·2 (1·5–3·2)
Antigua and Barbuda	Diarrheal diseases	51·7 (21·4–79·5)	12·6 (6·6–18·5)	39·0 (-1·2–77·7)	9·5 (5·7–12·7)
Antigua and Barbuda	Lower respiratory infections	46·1 (32·5–65·7)	26·5 (0·88–60·0)	15·1 (9·3–20·6)	16·2 (12·0–21·7)
Antigua and Barbuda	Malaria	0 (0–0)	0 (0–0)	--	0 (0–0)
Antigua and Barbuda	Measles	0 (0–0)	0 (0–0)	0 (0–0)	0 (0–0)
Bahamas	All causes	4·5 (3·1–6·2)	2·8 (0·93–5·5)	2·0 (1·6–2·5)	1·3 (0·88–1·8)
Bahamas	Diarrheal diseases	47·4 (22·5–72·0)	12·7 (6·6–18·6)	33·8 (-0·98–69·6)	9·9 (6·1–13·3)
Bahamas	Lower respiratory infections	42·2 (28·9–60·3)	25·1 (0·84–55·8)	12·0 (7·5–16·3)	15·6 (10·3–21·0)
Bahamas	Malaria	0 (0–0)	0 (0–0)	--	0 (0–0)
Bahamas	Measles	0 (0–0)	0 (0–0)	0 (0–0)	0 (0–0)
Barbados	All causes	3·0 (2·1–4·4)	1·8 (0·62–3·7)	1·4 (1·1–1·7)	0·85 (0·53–1·2)
Barbados	Diarrheal diseases	44·6 (18·5–70·8)	10·1 (5·2–14·9)	32·9 (-0·90–68·7)	9·0 (5·5–12·3)
Barbados	Lower respiratory infections	36·3 (25·1–52·2)	20·7 (0·63–48·1)	11·0 (6·8–15·0)	12·8 (7·8–18·0)
Barbados	Malaria	0 (0–0)	0 (0–0)	--	0 (0–0)
Barbados	Measles	0 (0–0)	0 (0–0)	0 (0–0)	0 (0–0)
Belize	All causes	8·8 (6·6–11·3)	5·7 (2·6–9·1)	3·8 (3·2–4·5)	3·1 (2·0–4·2)
Belize	Diarrheal diseases	49·1 (29·9–71·5)	14·8 (7·9–21·3)	29·1 (-0·72–66·7)	16·2 (10·1–20·8)
Belize	Lower respiratory infections	48·6 (34·5–64·7)	28·9 (1·1–60·0)	9·3 (5·5–12·9)	23·8 (15·4–31·1)
Belize	Malaria	0 (0–0)	0 (0–0)	--	0 (0–0)
Belize	Measles	0 (0–0)	0 (0–0)	0 (0–0)	0 (0–0)
Bermuda	All causes	1·1 (0·74–1·7)	0·64 (0·17–1·4)	0·49 (0·38–0·63)	0·28 (0·17–0·43)

Bermuda	Diarrheal diseases	40·9<br>(15·2–69·1)	8·6<br>(4·4–12·8)	31·3<br>(-0·83–67·2)	6·5<br>(3·8–8·7)
Bermuda	Lower respiratory infections	28·9<br>(18·9–45·3)	16·3<br>(0·46–41·3)	9·2<br>(5·6–12·6)	8·6<br>(5·2–12·1)
Bermuda	Malaria	0<br>(0–0)	0<br>(0–0)	--	0<br>(0–0)
Bermuda	Measles	19·3<br>(8·3–25·7)	5·6<br>(2·5–7·7)	7·3<br>(2·9–13·6)	7·8<br>(3·1–10·7)
Cuba	All causes	6·2<br>(3·9–9·4)	3·3<br>(0·61–7·6)	1·9<br>(1·4–2·4)	2·4<br>(1·8–3·2)
Cuba	Diarrheal diseases	39·1<br>(17·5–67·9)	8·4<br>(4·3–12·8)	26·9<br>(-0·64–65·0)	9·0<br>(5·3–12·2)
Cuba	Lower respiratory infections	34·7<br>(23·6–52·3)	18·8<br>(0·56–45·7)	7·3<br>(4·2–10·4)	15·2<br>(11·9–19·6)
Cuba	Malaria	0<br>(0–0)	0<br>(0–0)	--	0<br>(0–0)
Cuba	Measles	0<br>(0–0)	0<br>(0–0)	0<br>(0–0)	0<br>(0–0)
Dominica	All causes	5·1<br>(3·1–7·7)	3·2<br>(1·4–6·2)	2·6<br>(1·8–3·4)	1·3<br>(0·77–2·1)
Dominica	Diarrheal diseases	48·9<br>(22·8–75·7)	13·1<br>(7·1–19·1)	34·7<br>(-0·95–73·0)	10·4<br>(6·5–14·3)
Dominica	Lower respiratory infections	45·6<br>(30·0–64·6)	27·2<br>(0·96–59·6)	12·9<br>(7·9–17·5)	16·7<br>(11·0–22·5)
Dominica	Malaria	0<br>(0–0)	0<br>(0–0)	--	0<br>(0–0)
Dominica	Measles	0<br>(0–0)	0<br>(0–0)	0<br>(0–0)	0<br>(0–0)
Dominican Republic	All causes	4·2<br>(2·9–5·9)	2·7<br>(1·4–4·5)	2·2<br>(1·6–3·1)	1·1<br>(0·62–1·8)
Dominican Republic	Diarrheal diseases	44·8<br>(23·4–69·4)	12·6<br>(6·6–18·9)	29·6<br>(-0·77–65·8)	11·1<br>(6·9–15·3)
Dominican Republic	Lower respiratory infections	38·0<br>(24·1–55·4)	23·0<br>(0·85–50·2)	9·2<br>(4·7–14·0)	15·0<br>(7·7–22·7)
Dominican Republic	Malaria	7·7<br>(-3·4–26·8)	5·9<br>(-2·6–18·5)	--	2·1<br>(-0·80–10·3)
Dominican Republic	Measles	0<br>(0–0)	0<br>(0–0)	0<br>(0–0)	0<br>(0–0)
Grenada	All causes	5·0<br>(3·3–7·1)	3·2<br>(0·84–6·0)	1·9<br>(1·4–2·5)	1·7<br>(1·1–2·4)
Grenada	Diarrheal diseases	44·5<br>(23·0–66·8)	12·9<br>(6·8–19·0)	31·0<br>(-0·92–64·4)	10·0<br>(6·1–13·3)
Grenada	Lower respiratory infections	31·8<br>(22·5–44·1)	19·2<br>(0·65–40·8)	8·6<br>(5·1–12·2)	12·6<br>(8·8–16·8)
Grenada	Malaria	0<br>(0–0)	0<br>(0–0)	--	0<br>(0–0)
Grenada	Measles	0<br>(0–0)	0<br>(0–0)	0<br>(0–0)	0<br>(0–0)
Guyana	All causes	9·1<br>(6·2–11·3)	6·5<br>(3·6–9·2)	5·9<br>(4·4–7·1)	1·9<br>(1·3–2·7)
Guyana	Diarrheal diseases	59·1<br>(33·0–78·4)	20·9<br>(11·7–29·6)	42·9<br>(-1·7–76·1)	12·9<br>(7·8–17·2)
Guyana	Lower respiratory infections	44·4<br>(31·9–56·6)	28·5<br>(1·3–53·1)	16·1<br>(9·9–22·2)	16·5<br>(10·6–22·3)
Guyana	Malaria	13·9<br>(-6·7–42·6)	11·0<br>(-5·4–31·5)	--	3·8<br>(-1·3–16·8)
Guyana	Measles	0<br>(0–0)	0<br>(0–0)	0<br>(0–0)	0<br>(0–0)

Haiti	All causes	19·7 (13·3–25·3)	12·6 (6·7–17·9)	11·1 (6·3–15·4)	6·4 (4·3–9·1)
Haiti	Diarrheal diseases	67·9 (45·2–85·0)	28·3 (17·4–39·4)	45·3 (-1·9–81·5)	20·0 (13·6–26·0)
Haiti	Lower respiratory infections	52·6 (41·3–64·2)	34·7 (1·8–59·4)	16·6 (10·3–22·1)	25·3 (19·1–31·8)
Haiti	Malaria	20·0 (-10·8–58·1)	14·4 (-7·4–38·8)	--	8·2 (-3·4–34·2)
Haiti	Measles	0 (0–0)	0 (0–0)	0 (0–0)	0 (0–0)
Jamaica	All causes	2·3 (1·6–3·2)	1·4 (0·83–2·2)	1·3 (0·89–1·8)	0·56 (0·35–0·81)
Jamaica	Diarrheal diseases	36·4 (17·8–58·0)	9·3 (4·7–14·2)	24·7 (-0·66–55·4)	8·6 (5·4–11·7)
Jamaica	Lower respiratory infections	29·2 (19·6–43·6)	17·0 (0·50–40·2)	7·1 (4·1–10·1)	11·5 (7·0–16·3)
Jamaica	Malaria	0 (0–0)	0 (0–0)	--	0 (0–0)
Jamaica	Measles	0 (0–0)	0 (0–0)	0 (0–0)	0 (0–0)
Puerto Rico	All causes	3·1 (2·1–4·6)	1·9 (0·97–3·3)	1·8 (1·4–2·3)	0·67 (0·47–0·93)
Puerto Rico	Diarrheal diseases	39·5 (13·6–68·0)	7·9 (3·9–11·8)	30·2 (-0·79–66·3)	6·4 (3·8–8·7)
Puerto Rico	Lower respiratory infections	30·7 (20·4–48·3)	17·1 (0·47–43·4)	9·0 (5·4–12·4)	10·2 (7·6–13·7)
Puerto Rico	Malaria	0 (0–0)	0 (0–0)	--	0 (0–0)
Puerto Rico	Measles	22·7 (10·2–29·9)	7·1 (3·2–9·7)	8·5 (3·2–15·6)	9·1 (4·0–12·5)
Saint Kitts and Nevis	All causes	5·8 (4·2–7·9)	3·9 (2·3–6·0)	3·5 (2·7–4·2)	1·2 (0·81–1·8)
Saint Kitts and Nevis	Diarrheal diseases	45·7 (20·9–70·3)	12·2 (6·4–18·1)	32·6 (-0·91–68·2)	9·4 (5·7–12·6)
Saint Kitts and Nevis	Lower respiratory infections	38·8 (26·3–55·2)	23·3 (0·78–51·0)	11·0 (6·8–15·1)	13·7 (8·9–18·7)
Saint Kitts and Nevis	Malaria	0 (0–0)	0 (0–0)	--	0 (0–0)
Saint Kitts and Nevis	Measles	0 (0–0)	0 (0–0)	0 (0–0)	0 (0–0)
Saint Lucia	All causes	5·3 (3·8–7·4)	3·6 (2·0–6·0)	3·2 (2·4–4·1)	0·92 (0·60–1·3)
Saint Lucia	Diarrheal diseases	47·5 (19·9–73·9)	12·7 (6·6–18·4)	35·6 (-1·1–72·1)	6·8 (3·9–9·2)
Saint Lucia	Lower respiratory infections	39·3 (25·5–58·2)	24·6 (0·86–54·1)	12·2 (7·5–16·8)	11·0 (7·5–15·3)
Saint Lucia	Malaria	0 (0–0)	0 (0–0)	--	0 (0–0)
Saint Lucia	Measles	0 (0–0)	0 (0–0)	0 (0–0)	0 (0–0)
Saint Vincent and the Grenadines	All causes	10·0 (7·5–13·1)	7·1 (3·8–11·0)	5·8 (4·8–6·9)	2·2 (1·5–3·1)
Saint Vincent and the Grenadines	Diarrheal diseases	49·8 (24·3–75·4)	13·8 (7·3–19·9)	34·9 (-0·96–73·0)	10·7 (6·5–14·2)
Saint Vincent and the Grenadines	Lower respiratory infections	47·7 (32·4–67·4)	28·8 (0·99–61·7)	13·0 (8·0–17·7)	18·2 (12·5–24·3)
Saint Vincent and the Grenadines	Malaria	0 (0–0)	0 (0–0)	--	0 (0–0)

Saint Vincent and the Grenadines	Measles	0 (0–0)	0 (0–0)	0 (0–0)	0 (0–0)
Suriname	All causes	7·2 (4·2–9·9)	4·3 (2·1–6·6)	4·1 (2·3–6·1)	1·6 (1·0–2·4)
Suriname	Diarrheal diseases	56·6 (28·2–79·5)	18·8 (10·4–27·6)	40·9 (-1·4–77·4)	10·7 (6·4–15·0)
Suriname	Lower respiratory infections	52·0 (35·9–68·3)	33·7 (1·4–64·6)	17·4 (10·4–23·3)	17·5 (11·1–24·1)
Suriname	Malaria	8·2 (-4·5–32·9)	6·9 (-3·7–26·0)	--	1·8 (-0·71–10·3)
Suriname	Measles	0 (0–0)	0 (0–0)	0 (0–0)	0 (0–0)
Trinidad and Tobago	All causes	3·7 (2·6–4·9)	2·3 (0·97–3·8)	1·8 (1·4–2·2)	1·1 (0·73–1·6)
Trinidad and Tobago	Diarrheal diseases	53·0 (28·0–76·1)	16·6 (9·1–23·8)	36·4 (-1·1–73·3)	13·1 (8·4–17·2)
Trinidad and Tobago	Lower respiratory infections	43·3 (30·5–58·0)	26·7 (1·0–54·5)	12·3 (7·4–16·6)	17·5 (11·4–23·8)
Trinidad and Tobago	Malaria	0 (0–0)	0 (0–0)	--	0 (0–0)
Trinidad and Tobago	Measles	0 (0–0)	0 (0–0)	0 (0–0)	0 (0–0)
United States Virgin Islands	All causes	3·9 (2·5–5·5)	2·6 (1·7–3·7)	2·7 (1·8–3·8)	0·63 (0·41–0·91)
United States Virgin Islands	Diarrheal diseases	41·2 (17·1–67·5)	9·4 (4·7–14·0)	30·2 (-0·83–65·5)	7·7 (4·6–10·5)
United States Virgin Islands	Lower respiratory infections	35·5 (24·0–52·3)	20·3 (0·61–47·3)	10·0 (6·0–14·0)	12·8 (8·8–17·5)
United States Virgin Islands	Malaria	0 (0–0)	0 (0–0)	--	0 (0–0)
United States Virgin Islands	Measles	0 (0–0)	0 (0–0)	0 (0–0)	0 (0–0)
Central Latin America	All causes	11·2 (8·8–13·8)	6·9 (3·2–11·0)	4·2 (3·2–5·5)	4·8 (3·4–6·1)
Central Latin America	Diarrheal diseases	53·3 (39·2–71·4)	18·6 (10·3–27·1)	25·5 (-0·69–62·5)	23·0 (15·9–29·2)
Central Latin America	Lower respiratory infections	52·5 (39·0–68·6)	31·3 (1·3–63·6)	7·4 (4·4–10·2)	29·3 (19·5–37·0)
Central Latin America	Malaria	10·1 (-4·5–35·2)	7·0 (-2·9–21·6)	--	3·7 (-1·5–18·1)
Central Latin America	Measles	36·5 (18·1–44·2)	11·4 (5·5–14·8)	6·0 (2·3–11·0)	24·0 (11·3–30·7)
Colombia	All causes	10·2 (8·0–13·0)	7·6 (4·9–10·9)	6·2 (5·4–7·2)	2·2 (1·7–3·0)
Colombia	Diarrheal diseases	42·0 (22·4–66·3)	11·1 (5·7–16·8)	26·8 (-0·74–62·6)	10·8 (6·5–14·5)
Colombia	Lower respiratory infections	43·2 (29·2–64·3)	25·2 (0·81–57·9)	8·6 (5·1–11·8)	18·4 (13·6–24·0)
Colombia	Malaria	8·8 (-3·8–30·1)	6·5 (-2·8–19·8)	--	2·7 (-1·0–12·8)
Colombia	Measles	0 (0–0)	0 (0–0)	0 (0–0)	0 (0–0)
Costa Rica	All causes	2·0 (1·3–3·9)	0·99 (0·19–3·0)	0·79 (0·60–1·0)	0·57 (0·41–0·79)
Costa Rica	Diarrheal diseases	25·1 (7·1–53·8)	3·3 (1·7–5·3)	19·8 (-0·46–52·4)	3·5 (2·1–4·8)
Costa Rica	Lower respiratory infections	19·4 (12·9–35·9)	9·6 (0·21–31·1)	5·5 (3·1–7·7)	6·3 (5·0–8·3)

Costa Rica	Malaria	3·9 (-1·6–13·4)	3·0 (-1·2–9·5)	--	0·95 (-0·35–4·4)
Costa Rica	Measles	0 (0–0)	0 (0–0)	0 (0–0)	0 (0–0)
El Salvador	All causes	8·2 (5·9–11·5)	5·1 (2·3–8·8)	3·1 (2·3–4·1)	3·1 (2·1–4·2)
El Salvador	Diarrheal diseases	48·7 (30·9–70·3)	15·7 (8·3–23·1)	28·2 (-0·81–65·5)	15·6 (10·0–20·5)
El Salvador	Lower respiratory infections	50·4 (36·1–69·3)	30·7 (1·2–64·3)	9·2 (5·3–12·8)	24·1 (15·8–31·3)
El Salvador	Malaria	0 (0–0)	0 (0–0)	--	0 (0–0)
El Salvador	Measles	0 (0–0)	0 (0–0)	0 (0–0)	0 (0–0)
Guatemala	All causes	25·3 (20·0–30·5)	15·6 (6·1–23·5)	6·6 (4·2–10·0)	13·7 (9·8–17·4)
Guatemala	Diarrheal diseases	61·5 (52·0–74·1)	26·0 (15·5–36·8)	23·6 (-0·66–60·8)	31·8 (22·6–39·2)
Guatemala	Lower respiratory infections	64·5 (49·7–77·5)	41·3 (1·9–73·5)	6·7 (3·8–9·4)	40·3 (26·5–49·8)
Guatemala	Malaria	22·4 (-13·4–66·8)	13·7 (-7·2–37·5)	--	12·2 (-6·3–48·5)
Guatemala	Measles	0 (0–0)	0 (0–0)	0 (0–0)	0 (0–0)
Honduras	All causes	7·2 (4·8–10·3)	4·2 (2·0–7·0)	2·9 (1·6–4·8)	2·8 (1·9–4·1)
Honduras	Diarrheal diseases	47·4 (33·4–66·1)	17·1 (9·7–25·1)	25·2 (-0·74–59·8)	16·6 (10·8–21·4)
Honduras	Lower respiratory infections	38·3 (27·4–49·9)	24·0 (0·99–44·8)	6·0 (3·4–8·6)	20·2 (14·5–25·8)
Honduras	Malaria	15·6 (-7·9–47·5)	11·2 (-5·6–31·4)	--	5·9 (-2·4–25·2)
Honduras	Measles	0 (0–0)	0 (0–0)	0 (0–0)	0 (0–0)
Mexico	All causes	9·0 (7·1–11·4)	5·3 (2·2–9·0)	3·0 (2·3–4·0)	4·1 (2·8–5·4)
Mexico	Diarrheal diseases	51·3 (37·7–69·7)	17·1 (9·2–25·1)	24·2 (-0·62–61·4)	22·5 (14·9–28·6)
Mexico	Lower respiratory infections	53·9 (40·3–71·2)	30·8 (1·2–65·0)	7·3 (4·3–10·1)	30·3 (19·9–38·6)
Mexico	Malaria	11·1 (-5·0–38·7)	7·1 (-3·2–21·4)	--	5·0 (-2·0–23·5)
Mexico	Measles	0 (0–0)	0 (0–0)	0 (0–0)	0 (0–0)
Nicaragua	All causes	10·9 (7·5–14·8)	7·0 (3·5–11·5)	5·4 (3·9–6·9)	3·1 (1·9–4·4)
Nicaragua	Diarrheal diseases	47·4 (25·5–72·4)	13·3 (6·9–19·8)	29·8 (-0·74–68·2)	13·3 (8·3–17·5)
Nicaragua	Lower respiratory infections	43·2 (29·7–61·5)	25·9 (0·90–57·7)	9·2 (5·4–12·7)	18·3 (10·9–25·1)
Nicaragua	Malaria	7·6 (-3·8–34·4)	5·3 (-2·6–21·6)	--	2·9 (-1·2–16·8)
Nicaragua	Measles	0 (0–0)	0 (0–0)	0 (0–0)	0 (0–0)
Panama	All causes	11·6 (8·6–15·4)	7·2 (3·1–13·1)	4·3 (3·5–5·6)	4·4 (3·0–6·0)
Panama	Diarrheal diseases	44·4 (29·1–67·2)	12·8 (6·8–19·3)	23·7 (-0·56–61·3)	15·9 (10·2–21·0)

Panama	Lower respiratory infections	48·7 (34·6–68·8)	28·2 (0·92–63·1)	6·9 (4·0–9·5)	24·6 (16·7–31·7)
Panama	Malaria	8·1 (-3·4–27·4)	5·8 (-2·5–17·6)	--	2·7 (-1·0–12·2)
Panama	Measles	0 (0–0)	0 (0–0)	0 (0–0)	0 (0–0)
Venezuela (Bolivarian Republic of)	All causes	9·0 (6·7–11·5)	5·3 (3·3–7·6)	4·6 (3·1–6·3)	3·1 (2·1–4·1)
Venezuela (Bolivarian Republic of)	Diarrheal diseases	51·9 (31·7–75·1)	14·2 (7·4–21·0)	30·5 (-0·81–69·9)	18·7 (12·1–23·9)
Venezuela (Bolivarian Republic of)	Lower respiratory infections	39·9 (29·5–53·3)	22·6 (0·82–47·4)	7·7 (4·5–10·9)	20·8 (13·5–27·7)
Venezuela (Bolivarian Republic of)	Malaria	11·2 (-5·9–38·8)	7·3 (-3·8–22·3)	--	4·8 (-2·1–22·7)
Venezuela (Bolivarian Republic of)	Measles	36·5 (18·1–44·2)	11·4 (5·5–14·8)	6·0 (2·3–11·0)	24·0 (11·3–30·7)
Tropical Latin America	All causes	4·9 (3·4–6·7)	2·8 (0·96–5·4)	1·8 (1·5–2·2)	1·8 (1·3–2·4)
Tropical Latin America	Diarrheal diseases	46·1 (24·6–73·0)	11·6 (5·9–17·2)	29·6 (-0·76–68·8)	13·1 (8·0–17·1)
Tropical Latin America	Lower respiratory infections	50·6 (35·8–73·9)	28·6 (0·92–66·1)	10·3 (6·1–14·1)	22·7 (16·5–29·2)
Tropical Latin America	Malaria	9·5 (-4·2–31·4)	6·6 (-2·9–19·7)	--	3·4 (-1·3–15·2)
Tropical Latin America	Measles	27·2 (12·9–34·5)	8·5 (4·0–11·2)	6·4 (2·5–12·3)	15·3 (6·9–21·5)
Brazil	All causes	4·9 (3·4–6·7)	2·8 (0·92–5·4)	1·8 (1·5–2·2)	1·8 (1·3–2·4)
Brazil	Diarrheal diseases	47·0 (24·9–74·4)	11·8 (6·0–17·5)	30·3 (-0·79–70·1)	13·3 (8·2–17·4)
Brazil	Lower respiratory infections	51·1 (36·2–74·5)	28·8 (0·93–66·5)	10·5 (6·2–14·4)	22·9 (16·7–29·4)
Brazil	Malaria	9·5 (-4·2–31·4)	6·6 (-2·9–19·7)	--	3·4 (-1·3–15·2)
Brazil	Measles	27·2 (12·9–34·5)	8·5 (4·0–11·2)	6·4 (2·5–12·3)	15·3 (6·9–21·5)
Paraguay	All causes	4·6 (3·3–6·5)	3·0 (1·7–5·1)	2·1 (1·6–2·6)	1·3 (0·86–2·0)
Paraguay	Diarrheal diseases	31·9 (18·0–52·2)	9·1 (4·6–14·2)	17·4 (-0·35–47·8)	9·9 (5·8–13·7)
Paraguay	Lower respiratory infections	39·3 (25·7–61·1)	22·5 (0·68–53·9)	5·2 (2·9–7·4)	17·8 (12·5–23·8)
Paraguay	Malaria	0 (0–0)	0 (0–0)	--	0 (0–0)
Paraguay	Measles	0 (0–0)	0 (0–0)	0 (0–0)	0 (0–0)
**North Africa and Middle East**	**All causes**	**10·9 (7·9–13·8)**	**6·9 (3·3–10·3)**	**5·7 (4·0–7·4)**	**4·5 (3·3–5·9)**
**North Africa and Middle East**	**Diarrheal diseases**	**68·4 (43·9–83·7)**	**29·6 (19·1–40·0)**	**47·8 (-2·1–81·2)**	**23·9 (16·4–29·7)**
**North Africa and Middle East**	**Lower respiratory infections**	**54·3 (44·1–63·4)**	**34·7 (2·0–58·9)**	**20·8 (13·9–27·1)**	**27·7 (19·2–35·0)**
**North Africa and Middle East**	**Malaria**	**31·3 (-22·7–78·3)**	**24·3 (-16·2–58·1)**	**--**	**13·1 (-6·7–50·9)**
**North Africa and Middle East**	**Measles**	**61·4 (34·5–71·2)**	**30·0 (15·2–38·3)**	**22·4 (9·3–38·9)**	**33·5 (15·6–41·5)**
North Africa and Middle East	All causes	10·9 (7·9–13·8)	6·9 (3·3–10·3)	5·7 (4·0–7·4)	4·5 (3·3–5·9)

North Africa and Middle East	Diarrheal diseases	68·4 (43·9–83·7)	29·6 (19·1–40·0)	47·8 (-2·1–81·2)	23·9 (16·4–29·7)
North Africa and Middle East	Lower respiratory infections	54·3 (44·1–63·4)	34·7 (2·0–58·9)	20·8 (13·9–27·1)	27·7 (19·2–35·0)
North Africa and Middle East	Malaria	31·3 (-22·7–78·3)	24·3 (-16·2–58·1)	--	13·1 (-6·7–50·9)
North Africa and Middle East	Measles	61·4 (34·5–71·2)	30·0 (15·2–38·3)	22·4 (9·3–38·9)	33·5 (15·6–41·5)
Afghanistan	All causes	19·2 (14·6–23·6)	13·1 (7·6–17·7)	11·1 (7·5–14·7)	7·6 (5·1–10·4)
Afghanistan	Diarrheal diseases	77·3 (55·6–88·2)	39·1 (25·9–50·6)	52·4 (-2·2–85·1)	29·7 (21·9–36·5)
Afghanistan	Lower respiratory infections	56·2 (44·3–65·3)	38·9 (2·2–61·6)	21·6 (14·4–29·1)	30·6 (20·9–39·4)
Afghanistan	Malaria	27·1 (-16·7–72·9)	18·3 (-9·6–48·5)	--	14·4 (-6·8–54·3)
Afghanistan	Measles	69·2 (40·0–79·6)	35·5 (18·3–44·9)	24·7 (10·1–42·9)	38·3 (18·1–46·9)
Algeria	All causes	4·5 (2·9–6·5)	2·1 (0·72–4·2)	1·9 (1·3–2·7)	1·8 (1·1–2·5)
Algeria	Diarrheal diseases	56·3 (27·3–79·7)	14·9 (8·1–21·8)	41·3 (-1·4–77·7)	14·1 (9·1–18·8)
Algeria	Lower respiratory infections	49·9 (37·3–64·5)	28·1 (0·99–58·9)	17·3 (11·3–22·7)	21·0 (13·9–27·6)
Algeria	Malaria	0 (0–0)	0 (0–0)	--	0 (0–0)
Algeria	Measles	36·8 (17·3–46·6)	11·0 (5·1–14·7)	13·0 (4·8–23·8)	18·8 (8·3–24·8)
Bahrain	All causes	2·8 (1·6–4·2)	1·5 (0·47–3·1)	1·4 (0·93–1·8)	0·81 (0·53–1·2)
Bahrain	Diarrheal diseases	50·3 (20·4–75·5)	11·5 (5·9–17·2)	38·9 (-1·3–74·1)	9·1 (5·5–12·3)
Bahrain	Lower respiratory infections	42·3 (29·6–58·5)	23·5 (0·79–52·7)	14·7 (9·0–20·1)	15·3 (11·4–20·0)
Bahrain	Malaria	0 (0–0)	0 (0–0)	--	0 (0–0)
Bahrain	Measles	29·7 (0–40·6)	9·2 (0–12·9)	12·5 (0–23·5)	12·0 (0–16·9)
Egypt	All causes	10·8 (7·7–15·4)	5·4 (1·2–10·9)	4·9 (3·5–6·8)	4·6 (3·2–6·3)
Egypt	Diarrheal diseases	56·0 (27·2–78·4)	14·7 (8·1–22·0)	40·9 (-1·3–76·2)	17·1 (11·1–22·4)
Egypt	Lower respiratory infections	45·5 (35·4–57·5)	24·7 (1·0–51·5)	16·4 (9·8–23·0)	21·9 (14·5–29·6)
Egypt	Malaria	0 (0–0)	0 (0–0)	--	0 (0–0)
Egypt	Measles	42·8 (20·9–52·8)	11·4 (5·4–15·2)	15·7 (5·8–28·9)	23·8 (10·7–30·6)
Iran (Islamic Republic of)	All causes	2·3 (1·4–3·4)	1·3 (0·30–2·6)	1·0 (0·70–1·3)	0·67 (0·42–1·0)
Iran (Islamic Republic of)	Diarrheal diseases	51·4 (23·6–76·6)	14·0 (7·5–20·6)	38·2 (-1·4–74·4)	10·3 (6·2–13·8)
Iran (Islamic Republic of)	Lower respiratory infections	43·9 (30·6–62·6)	26·0 (0·91–57·0)	14·2 (8·9–18·9)	15·4 (10·6–20·7)
Iran (Islamic Republic of)	Malaria	7·2 (-3·4–24·0)	5·8 (-2·7–17·8)	--	1·8 (-0·68–8·7)
Iran (Islamic Republic of)	Measles	36·8 (18·6–45·4)	13·7 (6·8–17·9)	14·1 (5·8–24·6)	16·3 (7·5–21·2)

Iraq	All causes	6·1 (4·1–8·4)	3·2 (1·2–5·7)	2·9 (2·0–4·1)	2·5 (1·6–3·6)
Iraq	Diarrheal diseases	58·5 (31·1–78·0)	17·5 (9·9–25·2)	42·1 (-1·5–75·9)	17·3 (11·5–22·5)
Iraq	Lower respiratory infections	54·8 (42·8–67·7)	31·5 (1·2–62·3)	19·0 (12·6–25·0)	25·9 (18·1–33·4)
Iraq	Malaria	0 (0–0)	0 (0–0)	--	0 (0–0)
Iraq	Measles	42·9 (20·5–52·9)	13·1 (6·1–17·3)	15·3 (5·7–27·9)	22·9 (10·2–30·4)
Jordan	All causes	4·6 (2·8–7·5)	2·4 (0·31–6·0)	1·5 (1·1–2·1)	1·8 (1·1–2·9)
Jordan	Diarrheal diseases	42·9 (19·5–67·5)	9·1 (4·5–13·9)	30·1 (-0·85–65·5)	10·5 (6·2–14·1)
Jordan	Lower respiratory infections	40·3 (28·8–58·5)	21·8 (0·65–53·1)	10·2 (6·1–13·9)	17·2 (11·9–22·8)
Jordan	Malaria	0 (0–0)	0 (0–0)	--	0 (0–0)
Jordan	Measles	26·2 (12·5–32·1)	7·6 (3·7–10·0)	7·1 (2·7–13·0)	14·1 (6·4–18·7)
Kuwait	All causes	5·0 (3·1–7·8)	2·8 (0·15–6·7)	1·3 (0·87–1·7)	1·8 (1·3–2·5)
Kuwait	Diarrheal diseases	37·5 (16·7–62·9)	9·0 (4·5–13·5)	26·2 (-0·71–60·5)	7·7 (4·6–10·4)
Kuwait	Lower respiratory infections	33·4 (22·3–51·5)	19·2 (0·55–46·2)	8·0 (4·7–11·0)	12·4 (9·2–16·5)
Kuwait	Malaria	0 (0–0)	0 (0–0)	--	0 (0–0)
Kuwait	Measles	17·6 (7·9–22·0)	5·4 (2·4–7·4)	5·7 (2·3–10·3)	7·7 (3·4–10·5)
Lebanon	All causes	6·8 (4·6–9·4)	2·9 (0·66–6·1)	2·6 (1·8–3·5)	3·2 (2·2–4·6)
Lebanon	Diarrheal diseases	47·9 (24·1–70·9)	10·3 (5·3–15·5)	33·2 (-0·94–68·2)	15·4 (9·8–20·1)
Lebanon	Lower respiratory infections	40·6 (31·6–53·2)	20·6 (0·65–46·7)	11·3 (6·8–15·8)	20·7 (13·9–27·3)
Lebanon	Malaria	0 (0–0)	0 (0–0)	--	0 (0–0)
Lebanon	Measles	36·1 (17·0–45·5)	8·4 (4·0–11·2)	12·2 (4·5–22·7)	20·8 (9·5–27·5)
Libya	All causes	2·9 (2·0–4·3)	1·6 (0·30–3·3)	1·2 (0·78–1·7)	1·4 (0·92–2·1)
Libya	Diarrheal diseases	63·4 (35·1–86·0)	16·9 (9·5–25·0)	44·7 (-1·8–83·6)	20·4 (13·9–26·2)
Libya	Lower respiratory infections	63·2 (50·5–78·3)	34·3 (1·3–68·8)	19·7 (12·3–26·6)	33·7 (26·6–40·6)
Libya	Malaria	0 (0–0)	0 (0–0)	--	0 (0–0)
Libya	Measles	43·4 (21·8–53·6)	12·1 (6·0–15·9)	15·1 (5·7–27·1)	24·7 (11·3–31·5)
Morocco	All causes	6·9 (4·4–10·7)	3·0 (0·96–6·2)	2·8 (1·5–5·2)	2·9 (1·9–4·2)
Morocco	Diarrheal diseases	51·0 (25·7–78·5)	11·8 (6·3–17·7)	35·0 (-1·0–75·5)	14·6 (9·3–19·4)
Morocco	Lower respiratory infections	42·4 (31·3–58·6)	22·7 (0·74–51·8)	11·2 (6·8–15·6)	19·9 (13·5–26·1)
Morocco	Malaria	0 (0–0)	0 (0–0)	--	0 (0–0)

Morocco	Measles	32·2 (15·1–40·2)	8·3 (3·9–11·1)	9·2 (3·3–17·5)	18·9 (8·6–24·5)
Oman	All causes	4·9 (2·9–7·3)	3·0 (0·71–5·7)	2·4 (1·6–3·4)	1·5 (0·94–2·2)
Oman	Diarrheal diseases	59·6 (32·3–78·1)	21·3 (12·4–30·4)	44·4 (-2·1–76·4)	11·8 (7·4–15·9)
Oman	Lower respiratory infections	49·3 (35·2–63·2)	31·9 (1·4–59·3)	19·0 (12·2–25·1)	17·4 (12·6–22·5)
Oman	Malaria	13·3 (-6·6–41·6)	10·2 (-5·1–28·6)	--	4·4 (-1·6–20·9)
Oman	Measles	44·6 (22·4–56·5)	19·3 (9·3–25·0)	18·7 (7·8–32·2)	16·4 (7·1–21·9)
Palestine	All causes	1·7 (1·2–2·6)	0·78 (0·17–1·8)	0·49 (0·37–0·63)	0·74 (0·51–1·0)
Palestine	Diarrheal diseases	40·0 (18·6–67·0)	8·1 (4·2–12·4)	27·4 (-0·70–64·5)	10·3 (6·4–13·6)
Palestine	Lower respiratory infections	35·0 (25·7–52·2)	18·0 (0·50–44·3)	7·6 (4·4–10·7)	16·3 (12·1–21·0)
Palestine	Malaria	0 (0–0)	0 (0–0)	--	0 (0–0)
Palestine	Measles	23·6 (10·9–28·7)	6·0 (2·8–7·9)	6·2 (2·3–11·6)	13·5 (6·0–18·0)
Qatar	All causes	3·3 (2·0–5·3)	1·6 (0·25–3·8)	1·2 (0·87–1·7)	1·1 (0·79–1·7)
Qatar	Diarrheal diseases	39·2 (14·0–67·2)	7·0 (3·5–10·6)	30·2 (-0·89–65·6)	6·8 (4·1–9·4)
Qatar	Lower respiratory infections	31·8 (22·5–51·3)	16·4 (0·44–44·7)	9·5 (5·8–13·5)	11·8 (9·1–15·6)
Qatar	Malaria	0 (0–0)	0 (0–0)	--	0 (0–0)
Qatar	Measles	21·1 (9·6–27·1)	5·6 (2·6–7·5)	7·6 (2·9–14·0)	9·6 (4·4–13·1)
Saudi Arabia	All causes	9·1 (6·4–12·1)	4·3 (0·72–9·2)	4·7 (3·5–6·1)	2·8 (1·9–4·0)
Saudi Arabia	Diarrheal diseases	59·4 (20·8–86·4)	11·7 (6·2–17·8)	49·0 (-1·9–85·3)	9·8 (6·1–13·3)
Saudi Arabia	Lower respiratory infections	52·3 (40·4–68·6)	27·0 (0·88–61·0)	23·2 (15·1–30·6)	18·0 (13·6–23·0)
Saudi Arabia	Malaria	8·9 (-3·9–30·2)	6·7 (-3·0–20·6)	--	2·6 (-0·98–12·1)
Saudi Arabia	Measles	36·0 (17·1–47·7)	10·0 (4·8–13·3)	18·7 (7·2–33·0)	12·9 (5·6–17·3)
Sudan	All causes	11·5 (5·9–16·3)	7·8 (1·7–12·6)	5·6 (3·7–7·7)	4·6 (3·0–6·2)
Sudan	Diarrheal diseases	76·8 (52·2–88·3)	36·8 (24·0–48·6)	56·7 (-3·8–86·3)	21·9 (14·9–27·8)
Sudan	Lower respiratory infections	70·5 (56·3–79·2)	49·1 (3·2–75·5)	31·6 (21·8–40·4)	33·1 (24·9–40·4)
Sudan	Malaria	30·2 (-21·7–77·2)	23·5 (-15·7–57·7)	--	12·1 (-5·5–47·9)
Sudan	Measles	70·8 (43·3–82·1)	38·0 (20·7–47·3)	32·2 (14·6–51·1)	33·0 (15·9–41·2)
Syrian Arab Republic	All causes	7·5 (5·6–9·7)	3·9 (1·4–6·4)	3·2 (2·3–4·2)	4·0 (2·8–5·3)
Syrian Arab Republic	Diarrheal diseases	70·4 (44·8–86·5)	23·6 (13·8–33·9)	50·1 (-2·2–84·3)	25·7 (18·3–32·1)
Syrian Arab Republic	Lower respiratory infections	71·4 (58·8–83·4)	41·5 (1·8–73·7)	25·1 (15·3–34·2)	41·8 (35·1–48·2)

Syrian Arab Republic	Malaria	0 (0–0)	0 (0–0)	--	0 (0–0)
Syrian Arab Republic	Measles	58·7 (30·9–71·1)	19·8 (9·6–25·9)	24·6 (9·5–42·3)	33·1 (15·4–41·7)
Tunisia	All causes	4·1 (2·6–6·0)	1·9 (0·71–3·7)	1·9 (1·1–3·0)	1·5 (0·99–2·2)
Tunisia	Diarrheal diseases	45·8 (20·7–71·6)	10·3 (5·5–15·2)	32·4 (-0·82–69·3)	12·1 (7·6–16·0)
Tunisia	Lower respiratory infections	42·6 (31·6–59·9)	22·4 (0·66–52·9)	11·6 (7·1–15·8)	19·2 (12·9–25·5)
Tunisia	Malaria	0 (0–0)	0 (0–0)	--	0 (0–0)
Tunisia	Measles	25·5 (11·7–32·2)	5·7 (2·6–7·7)	7·5 (2·7–14·2)	14·7 (6·6–19·5)
Türkiye	All causes	1·9 (1·2–2·7)	0·94 (0·39–1·8)	0·81 (0·58–1·1)	0·64 (0·43–0·94)
Türkiye	Diarrheal diseases	36·6 (15·0–63·5)	6·6 (3·2–10·3)	26·0 (-0·63–61·1)	8·5 (5·0–11·6)
Türkiye	Lower respiratory infections	34·4 (21·9–53·2)	17·2 (0·45–46·2)	8·3 (4·4–12·4)	14·8 (10·2–20·3)
Türkiye	Malaria	0 (0–0)	0 (0–0)	--	0 (0–0)
Türkiye	Measles	20·3 (9·2–24·6)	5·2 (2·4–7·0)	4·5 (1·6–8·3)	12·1 (5·2–16·1)
United Arab Emirates	All causes	8·0 (5·5–10·9)	4·5 (1·2–7·9)	4·1 (3·0–5·7)	2·8 (1·9–3·9)
United Arab Emirates	Diarrheal diseases	62·0 (30·6–80·9)	19·4 (11·3–27·8)	48·1 (-2·1–79·5)	13·3 (8·4–17·6)
United Arab Emirates	Lower respiratory infections	50·8 (38·8–63·8)	30·4 (1·2–58·4)	21·9 (14·4–28·8)	19·8 (14·9–25·7)
United Arab Emirates	Malaria	0 (0–0)	0 (0–0)	--	0 (0–0)
United Arab Emirates	Measles	50·5 (25·8–65·6)	18·9 (9·1–25·1)	26·6 (11·0–45·3)	17·9 (8·1–23·7)
Yemen	All causes	12·7 (8·3–16·6)	9·4 (3·1–13·8)	6·6 (4·7–8·6)	6·1 (4·3–8·3)
Yemen	Diarrheal diseases	80·7 (64·1–88·0)	47·5 (34·2–58·7)	56·4 (-3·9–84·5)	32·7 (24·0–39·1)
Yemen	Lower respiratory infections	74·1 (63·8–80·9)	55·2 (4·6–77·3)	32·4 (22·4–41·0)	42·9 (30·6–51·4)
Yemen	Malaria	34·9 (-29·4–83·1)	26·9 (-20·2–62·8)	--	16·1 (-8·4–59·0)
Yemen	Measles	77·7 (50·2–85·7)	46·6 (26·4–55·5)	29·9 (13·7–47·6)	43·3 (21·5–51·7)
**South Asia**	**All causes**	**14·6 (11·8–16·8)**	**10·0 (4·3–13·2)**	**8·7 (6·3–10·4)**	**6·1 (4·4–7·9)**
**South Asia**	**Diarrheal diseases**	**79·4 (58·3–88·3)**	**42·8 (29·6–54·0)**	**58·6 (-3·9–86·6)**	**27·7 (19·6–34·2)**
**South Asia**	**Lower respiratory infections**	**53·4 (45·4–59·0)**	**38·4 (2·9–56·2)**	**26·9 (19·5–33·4)**	**27·0 (17·4–34·0)**
**South Asia**	**Malaria**	**32·1 (-24·3–78·9)**	**25·0 (-17·4–60·1)**	**--**	**13·8 (-7·3–51·8)**
**South Asia**	**Measles**	**75·9 (49·1–85·7)**	**42·5 (23·9–51·6)**	**36·0 (16·5–55·4)**	**38·4 (19·2–46·8)**
South Asia	All causes	14·6 (11·8–16·8)	10·0 (4·3–13·2)	8·7 (6·3–10·4)	6·1 (4·4–7·9)
South Asia	Diarrheal diseases	79·4 (58·3–88·3)	42·8 (29·6–54·0)	58·6 (-3·9–86·6)	27·7 (19·6–34·2)

South Asia	Lower respiratory infections	53·4 (45·4–59·0)	38·4 (2·9–56·2)	26·9 (19·5–33·4)	27·0 (17·4–34·0)
South Asia	Malaria	32·1 (-24·3–78·9)	25·0 (-17·4–60·1)	--	13·8 (-7·3–51·8)
South Asia	Measles	75·9 (49·1–85·7)	42·5 (23·9–51·6)	36·0 (16·5–55·4)	38·4 (19·2–46·8)
Bangladesh	All causes	10·2 (7·7–12·5)	7·5 (3·9–10·3)	6·2 (5·0–7·7)	3·2 (2·0–4·4)
Bangladesh	Diarrheal diseases	70·7 (47·8–84·4)	33·6 (21·3–45·0)	49·7 (-3·0–81·3)	21·3 (13·9–27·0)
Bangladesh	Lower respiratory infections	40·8 (29·3–50·5)	28·5 (1·7–47·0)	16·3 (10·0–23·0)	19·1 (12·2–26·5)
Bangladesh	Malaria	25·0 (-15·9–66·0)	20·2 (-12·4–51·1)	--	8·2 (-3·1–33·1)
Bangladesh	Measles	65·8 (38·7–75·6)	34·7 (18·6–42·9)	25·6 (11·0–42·6)	31·5 (15·3–39·9)
Bhutan	All causes	10·8 (6·6–16·2)	5·4 (2·1–9·5)	5·0 (2·2–9·5)	4·2 (2·9–6·2)
Bhutan	Diarrheal diseases	61·8 (36·6–82·6)	20·5 (11·3–29·6)	41·8 (-1·5–79·8)	18·2 (11·7–23·7)
Bhutan	Lower respiratory infections	52·5 (40·0–66·5)	32·3 (1·4–61·9)	15·4 (9·6–20·9)	25·3 (18·8–32·0)
Bhutan	Malaria	12·3 (-7·7–50·5)	8·9 (-5·1–33·0)	--	4·8 (-2·2–27·0)
Bhutan	Measles	46·3 (24·4–55·0)	17·6 (8·9–22·8)	13·0 (4·8–23·3)	25·5 (12·3–32·8)
India	All causes	16·3 (13·1–19·3)	11·3 (4·9–15·2)	9·9 (7·3–12·0)	7·3 (5·3–9·4)
India	Diarrheal diseases	80·0 (59·9–87·6)	45·3 (31·9–56·4)	59·8 (-4·1–85·8)	29·6 (20·7–36·1)
India	Lower respiratory infections	51·9 (45·1–57·9)	37·7 (3·0–54·9)	27·6 (20·2–34·8)	26·9 (16·8–34·4)
India	Malaria	31·9 (-24·0–78·9)	25·1 (-17·5–60·0)	--	13·2 (-6·5–50·0)
India	Measles	78·0 (51·2–87·8)	44·3 (25·1–53·5)	38·2 (17·8–58·1)	39·6 (19·8–48·1)
Nepal	All causes	12·7 (9·7–16·1)	8·9 (4·5–12·7)	7·0 (5·5–8·7)	4·6 (3·1–6·2)
Nepal	Diarrheal diseases	69·6 (47·0–82·5)	32·4 (20·8–42·6)	49·3 (-2·4–79·7)	20·9 (14·0–26·2)
Nepal	Lower respiratory infections	51·9 (40·1–61·9)	35·8 (2·2–57·5)	21·0 (13·9–28·6)	24·1 (16·2–31·4)
Nepal	Malaria	25·0 (-15·8–70·3)	19·6 (-10·7–51·7)	--	9·2 (-4·3–40·7)
Nepal	Measles	65·9 (38·4–76·1)	33·9 (17·9–42·5)	24·5 (10·5–40·7)	33·0 (15·7–41·3)
Pakistan	All causes	12·4 (8·8–16·4)	8·1 (3·1–11·5)	7·2 (4·0–10·1)	4·7 (3·2–6·7)
Pakistan	Diarrheal diseases	79·3 (55·6–91·5)	39·1 (26·3–51·5)	57·5 (-3·5–89·3)	24·7 (17·5–31·0)
Pakistan	Lower respiratory infections	64·7 (53·3–74·6)	45·3 (3·0–70·0)	28·4 (19·7–36·1)	30·9 (21·3–39·2)
Pakistan	Malaria	32·8 (-26·1–80·0)	24·8 (-16·9–60·2)	--	15·7 (-9·1–56·3)
Pakistan	Measles	69·5 (43·0–79·8)	36·3 (19·9–44·9)	29·8 (13·3–48·2)	35·8 (18·1–44·5)
**Southeast Asia, East Asia, and Oceania**	**All causes**	**12·3 (9·1–15·2)**	**7·7 (3·1–11·2)**	**5·9 (4·1–7·7)**	**5·2 (3·8–6·8)**

**Southeast Asia, East Asia, and Oceania**	**Diarrheal diseases**	**73·0 (50·6–86·9)**	**32·5 (20·3–43·5)**	**49·1 (-2·2–84·1)**	**25·5 (17·3–31·7)**
**Southeast Asia, East Asia, and Oceania**	**Lower respiratory infections**	**56·8 (43·9–66·3)**	**38·0 (2·2–62·9)**	**19·6 (13·0–25·3)**	**28·2 (19·6–35·1)**
**Southeast Asia, East Asia, and Oceania**	**Malaria**	**25·7 (-17·2–68·9)**	**18·1 (-10·6–47·0)**	**--**	**12·3 (-6·1–46·3)**
**Southeast Asia, East Asia, and Oceania**	**Measles**	**61·5 (35·8–70·9)**	**29·7 (15·8–37·2)**	**20·6 (8·6–35·3)**	**33·3 (16·4–41·2)**
East Asia	All causes	6·0 (3·9–7·9)	3·7 (0·70–6·9)	1·9 (1·4–2·5)	2·4 (1·7–3·3)
East Asia	Diarrheal diseases	55·6 (31·7–80·3)	17·9 (10·1–26·4)	36·6 (-1·2–76·6)	14·6 (9·2–19·3)
East Asia	Lower respiratory infections	50·5 (35·0–67·7)	31·2 (1·5–62·4)	12·8 (7·8–17·5)	21·9 (15·9–28·2)
East Asia	Malaria	21·5 (-19·6–54·1)	20·3 (-17·5–50·2)	--	2·2 (-1·6–10·2)
East Asia	Measles	34·1 (17·2–40·9)	13·1 (6·6–17·2)	8·9 (3·5–16·2)	17·2 (8·1–22·7)
China	All causes	5·6 (3·7–7·6)	3·3 (0·62–6·5)	1·8 (1·3–2·3)	2·2 (1·6–3·0)
China	Diarrheal diseases	54·0 (30·0–79·8)	16·1 (8·7–23·9)	36·0 (-1·1–76·3)	13·6 (8·4–18·2)
China	Lower respiratory infections	49·0 (34·0–67·0)	29·3 (1·1–61·0)	12·4 (7·4–17·1)	20·7 (15·6–26·4)
China	Malaria	0 (0–0)	0 (0–0)	--	0 (0–0)
China	Measles	34·7 (17·6–41·7)	13·4 (6·7–17·4)	8·9 (3·5–16·2)	17·7 (8·4–23·2)
Democratic People's Republic of Korea	All causes	11·3 (6·7–16·2)	8·4 (1·6–14·7)	3·4 (2·2–5·0)	5·5 (3·0–8·6)
Democratic People's Republic of Korea	Diarrheal diseases	73·5 (56·9–85·2)	38·2 (23·8–50·4)	42·9 (-1·7–79·5)	26·4 (16·9–34·7)
Democratic People's Republic of Korea	Lower respiratory infections	63·3 (47·6–73·4)	47·0 (4·1–70·1)	16·7 (10·5–22·4)	32·6 (19·5–43·8)
Democratic People's Republic of Korea	Malaria	21·5 (-19·6–54·1)	20·3 (-17·5–50·2)	--	2·2 (-1·6–10·2)
Democratic People's Republic of Korea	Measles	0 (0–0)	0 (0–0)	0 (0–0)	0 (0–0)
Taiwan	All causes	4·0 (2·6–6·1)	2·6 (0·75–5·3)	1·6 (1·2–2·0)	0·91 (0·64–1·3)
Taiwan	Diarrheal diseases	43·0 (16·9–73·5)	10·5 (5·3–15·8)	31·8 (-0·87–71·2)	5·8 (3·3–7·9)
Taiwan	Lower respiratory infections	33·6 (20·3–52·9)	21·1 (0·67–49·0)	8·7 (5·2–11·7)	9·5 (6·8–12·8)
Taiwan	Malaria	0 (0–0)	0 (0–0)	--	0 (0–0)
Taiwan	Measles	21·9 (10·1–28·1)	7·9 (3·6–10·5)	8·8 (3·2–16·4)	7·1 (3·1–9·9)
Oceania	All causes	24·8 (18·5–30·7)	16·4 (7·3–23·2)	12·3 (9·6–15·1)	12·0 (8·5–15·3)
Oceania	Diarrheal diseases	77·3 (57·6–89·9)	36·8 (24·4–48·5)	51·5 (-2·4–86·8)	33·0 (24·4–39·5)
Oceania	Lower respiratory infections	53·3 (42·8–61·8)	36·1 (2·3–57·1)	20·5 (13·3–27·8)	31·1 (23·2–38·9)
Oceania	Malaria	29·2 (-20·8–75·9)	19·4 (-11·6–49·4)	--	16·3 (-8·9–58·4)
Oceania	Measles	71·2 (43·3–80·9)	34·5 (18·4–43·4)	25·4 (10·6–43·1)	43·2 (21·3–51·7)

American Samoa	All causes	10·3 (7·0–14·8)	6·6 (3·2–11·5)	5·0 (3·9–6·4)	2·6 (1·7–3·8)
American Samoa	Diarrheal diseases	51·5 (25·6–80·1)	14·6 (7·9–21·8)	36·2 (-1·2–77·0)	9·9 (6·0–13·6)
American Samoa	Lower respiratory infections	48·9 (32·4–70·8)	30·2 (0·99–65·9)	13·9 (8·4–19·1)	16·2 (10·8–21·7)
American Samoa	Malaria	0 (0–0)	0 (0–0)	--	0 (0–0)
American Samoa	Measles	30·1 (14·5–37·9)	11·4 (5·4–15·1)	9·6 (3·5–17·4)	12·9 (5·8–17·2)
Cook Islands	All causes	8·5 (5·1–14·6)	4·9 (0·42–13·1)	2·9 (1·8–4·3)	2·2 (1·3–3·4)
Cook Islands	Diarrheal diseases	43·2 (15·1–74·9)	8·9 (4·6–13·5)	33·5 (-0·94–72·7)	5·7 (3·3–7·8)
Cook Islands	Lower respiratory infections	39·3 (24·5–64·0)	22·9 (0·60–58·8)	12·6 (7·7–17·3)	10·1 (6·8–13·9)
Cook Islands	Malaria	0 (0–0)	0 (0–0)	--	0 (0–0)
Cook Islands	Measles	20·9 (9·5–27·8)	6·9 (3·2–9·4)	7·9 (3·0–14·5)	7·8 (3·4–10·7)
Fiji	All causes	9·3 (5·9–13·3)	5·9 (1·8–10·6)	4·6 (3·5–6·0)	1·8 (1·2–2·6)
Fiji	Diarrheal diseases	56·1 (24·8–83·5)	16·6 (9·0–24·2)	42·5 (-1·5–81·6)	6·9 (4·0–9·7)
Fiji	Lower respiratory infections	53·3 (33·5–75·8)	34·2 (1·2–70·2)	19·7 (12·4–26·4)	12·2 (8·7–16·3)
Fiji	Malaria	0 (0–0)	0 (0–0)	--	0 (0–0)
Fiji	Measles	32·4 (15·4–42·4)	12·6 (6·1–16·7)	14·2 (5·5–25·7)	9·7 (4·4–13·1)
Guam	All causes	7·4 (4·5–11·9)	4·3 (0·75–9·5)	3·0 (2·2–3·9)	1·7 (1·0–2·6)
Guam	Diarrheal diseases	43·6 (16·4–74·3)	9·7 (4·9–14·5)	32·9 (-0·88–72·1)	6·4 (3·8–8·8)
Guam	Lower respiratory infections	40·2 (24·8–64·4)	23·8 (0·63–59·2)	12·4 (7·6–17·0)	10·8 (6·7–15·1)
Guam	Malaria	0 (0–0)	0 (0–0)	--	0 (0–0)
Guam	Measles	23·5 (10·4–30·7)	8·2 (3·7–11·1)	8·9 (3·4–16·5)	8·6 (3·7–11·7)
Kiribati	All causes	19·5 (14·1–25·4)	14·3 (8·4–20·2)	12·2 (8·6–15·7)	4·2 (2·8–6·1)
Kiribati	Diarrheal diseases	64·4 (40·8–85·9)	25·0 (14·6–35·9)	42·4 (-1·7–81·9)	15·4 (9·9–20·6)
Kiribati	Lower respiratory infections	66·2 (46·4–85·0)	44·0 (2·0–80·4)	20·1 (12·6–26·9)	25·4 (17·8–32·7)
Kiribati	Malaria	0 (0–0)	0 (0–0)	--	0 (0–0)
Kiribati	Measles	42·9 (22·3–51·8)	19·9 (9·9–25·8)	11·7 (4·7–21·0)	19·6 (9·1–25·5)
Marshall Islands	All causes	16·4 (9·1–23·9)	12·6 (3·1–21·8)	2·8 (2·1–3·9)	5·5 (3·4–8·0)
Marshall Islands	Diarrheal diseases	45·6 (40·5–52·8)	27·6 (17·1–38·3)	13·4 (-0·32–37·4)	13·1 (8·1–17·3)
Marshall Islands	Lower respiratory infections	56·2 (28·8–78·9)	43·3 (1·9–76·4)	3·8 (2·2–5·2)	21·1 (14·1–27·6)
Marshall Islands	Malaria	0 (0–0)	0 (0–0)	--	0 (0–0)

Marshall Islands	Measles	36·3 (18·3–43·7)	22·6 (11·1–29·3)	1·6 (0·69–2·8)	16·6 (7·5–21·7)
Micronesia (Federated States of)	All causes	13·6 (9·2–17·9)	9·5 (5·4–13·5)	8·0 (5·3–10·6)	3·4 (2·2–4·6)
Micronesia (Federated States of)	Diarrheal diseases	59·6 (36·1–82·5)	21·2 (12·1–30·8)	38·9 (-1·3–78·6)	14·8 (9·3–19·8)
Micronesia (Federated States of)	Lower respiratory infections	59·9 (41·9–79·4)	38·5 (1·6–73·7)	16·1 (9·9–22·0)	24·5 (18·1–31·3)
Micronesia (Federated States of)	Malaria	0 (0–0)	0 (0–0)	--	0 (0–0)
Micronesia (Federated States of)	Measles	39·6 (20·1–47·9)	16·8 (8·3–22·0)	11·2 (4·3–20·3)	18·5 (8·6–24·3)
Nauru	All causes	16·0 (10·7–23·4)	10·1 (2·5–19·5)	4·6 (3·7–5·7)	6·6 (4·4–9·7)
Nauru	Diarrheal diseases	46·9 (31·8–67·0)	15·2 (8·2–22·8)	24·1 (-0·66–59·6)	16·7 (10·9–22·0)
Nauru	Lower respiratory infections	55·4 (38·1–76·8)	33·4 (1·2–68·5)	8·5 (5·1–11·6)	26·8 (19·3–34·1)
Nauru	Malaria	0 (0–0)	0 (0–0)	--	0 (0–0)
Nauru	Measles	32·6 (16·0–39·5)	11·2 (5·4–14·7)	3·7 (1·5–6·7)	21·4 (9·8–27·8)
Niue	All causes	10·8 (6·7–16·1)	6·9 (1·6–13·9)	4·3 (3·3–5·8)	3·0 (2·0–4·6)
Niue	Diarrheal diseases	50·4 (24·3–78·7)	13·7 (7·5–20·7)	35·7 (-1·1–76·1)	9·3 (5·7–13·1)
Niue	Lower respiratory infections	49·5 (32·1–72·1)	30·2 (1·0–66·2)	14·0 (8·6–19·0)	16·4 (11·9–21·3)
Niue	Malaria	0 (0–0)	0 (0–0)	--	0 (0–0)
Niue	Measles	28·4 (13·4–36·0)	10·6 (5·1–14·1)	9·0 (3·5–16·6)	12·1 (5·4–16·1)
Northern Mariana Islands	All causes	5·8 (3·4–9·5)	3·4 (1·2–6·8)	2·7 (1·9–3·8)	1·5 (1·0–2·1)
Northern Mariana Islands	Diarrheal diseases	42·9 (18·3–71·0)	10·1 (5·3–15·5)	31·5 (-0·98–68·8)	7·0 (4·3–9·7)
Northern Mariana Islands	Lower respiratory infections	36·8 (23·9–57·4)	21·5 (0·62–51·4)	10·6 (6·3–14·7)	11·5 (9·1–15·1)
Northern Mariana Islands	Malaria	0 (0–0)	0 (0–0)	--	0 (0–0)
Northern Mariana Islands	Measles	23·5 (11·0–30·3)	8·1 (3·8–10·9)	8·1 (3·1–14·9)	9·5 (4·3–12·9)
Palau	All causes	7·9 (4·7–12·4)	4·6 (1·4–9·2)	3·7 (2·5–5·3)	1·9 (1·1–3·0)
Palau	Diarrheal diseases	50·2 (22·1–79·1)	13·2 (7·0–19·5)	36·7 (-1·1–76·7)	8·4 (5·0–11·4)
Palau	Lower respiratory infections	48·2 (31·2–70·5)	29·6 (0·95–65·8)	14·6 (9·1–19·9)	14·2 (8·3–19·8)
Palau	Malaria	0 (0–0)	0 (0–0)	--	0 (0–0)
Palau	Measles	27·7 (13·1–35·6)	10·3 (4·9–13·7)	9·4 (3·5–17·2)	11·2 (4·9–15·2)
Papua New Guinea	All causes	25·9 (19·1–32·0)	17·1 (7·7–24·2)	12·8 (9·9–15·8)	12·7 (9·1–16·2)
Papua New Guinea	Diarrheal diseases	78·3 (59·2–90·5)	37·8 (25·1–49·7)	52·1 (-2·5–87·4)	34·1 (25·3–40·7)
Papua New Guinea	Lower respiratory infections	53·5 (43·2–62·3)	36·3 (2·4–57·0)	20·8 (13·4–28·2)	31·9 (23·7–39·7)

Papua New Guinea	Malaria	29·3 (-20·8–75·9)	19·4 (-11·7–49·5)	--	16·3 (-8·9–58·5)
Papua New Guinea	Measles	72·2 (44·0–81·9)	35·1 (18·7–44·1)	25·8 (10·8–43·7)	44·0 (21·7–52·6)
Samoa	All causes	11·3 (6·9–16·7)	6·5 (1·6–13·4)	5·2 (3·7–7·2)	2·9 (1·7–4·6)
Samoa	Diarrheal diseases	53·6 (22·9–82·4)	12·7 (6·6–19·3)	41·1 (-1·5–80·4)	8·4 (5·0–11·9)
Samoa	Lower respiratory infections	50·6 (35·8–72·1)	28·9 (0·96–65·6)	19·1 (12·1–25·5)	14·5 (9·9–19·1)
Samoa	Malaria	0 (0–0)	0 (0–0)	--	0 (0–0)
Samoa	Measles	28·7 (13·6–35·9)	9·8 (4·7–12·9)	11·6 (4·5–21·0)	10·6 (4·7–14·4)
Solomon Islands	All causes	13·3 (9·0–16·9)	8·3 (4·2–12·1)	6·5 (4·6–8·3)	5·2 (3·5–7·3)
Solomon Islands	Diarrheal diseases	66·4 (41·8–82·0)	26·6 (16·0–36·1)	45·3 (-1·7–79·6)	21·4 (14·8–27·1)
Solomon Islands	Lower respiratory infections	35·5 (26·3–44·0)	23·1 (1·1–40·7)	12·3 (7·8–17·1)	16·8 (10·2–22·4)
Solomon Islands	Malaria	15·2 (-8·9–44·4)	10·4 (-5·4–27·7)	--	7·0 (-3·1–29·7)
Solomon Islands	Measles	59·3 (32·3–70·7)	26·3 (13·2–33·9)	21·0 (8·3–36·4)	31·4 (14·5–39·5)
Tokelau	All causes	11·8 (7·2–17·1)	7·4 (1·6–14·6)	4·6 (3·4–6·2)	3·4 (2·2–5·3)
Tokelau	Diarrheal diseases	51·6 (25·6–79·2)	14·7 (8·0–22·2)	36·2 (-1·2–76·2)	10·0 (6·1–13·8)
Tokelau	Lower respiratory infections	49·8 (32·6–71·6)	30·7 (1·0–65·6)	14·2 (8·6–19·3)	16·9 (12·4–21·9)
Tokelau	Malaria	0 (0–0)	0 (0–0)	--	0 (0–0)
Tokelau	Measles	30·0 (14·4–37·6)	11·4 (5·5–15·1)	9·4 (3·6–17·0)	12·9 (5·8–17·2)
Tonga	All causes	6·9 (4·2–11·9)	4·5 (1·4–10·2)	3·3 (2·3–4·4)	1·3 (0·85–2·1)
Tonga	Diarrheal diseases	38·2 (12·0–71·7)	6·6 (3·3–10·3)	30·5 (-0·77–70·1)	4·5 (2·4–6·5)
Tonga	Lower respiratory infections	35·7 (21·9–61·7)	20·2 (0·48–55·3)	11·5 (6·6–16·5)	8·9 (6·4–12·3)
Tonga	Malaria	0 (0–0)	0 (0–0)	--	0 (0–0)
Tonga	Measles	16·4 (7·0–23·4)	4·7 (2·1–6·4)	7·6 (2·6–14·8)	5·2 (2·3–7·2)
Tuvalu	All causes	13·4 (8·7–20·0)	7·7 (2·2–15·3)	5·2 (3·9–7·2)	4·7 (2·8–7·2)
Tuvalu	Diarrheal diseases	49·8 (25·3–78·6)	13·0 (7·1–20·1)	33·9 (-1·2–75·4)	12·7 (7·8–17·2)
Tuvalu	Lower respiratory infections	48·7 (34·3–68·3)	28·2 (0·95–62·1)	12·0 (7·1–16·7)	20·4 (12·6–27·4)
Tuvalu	Malaria	0 (0–0)	0 (0–0)	--	0 (0–0)
Tuvalu	Measles	27·9 (13·1–34·5)	9·3 (4·4–12·4)	8·3 (3·2–15·9)	13·6 (6·1–18·3)
Vanuatu	All causes	20·8 (14·6–26·8)	11·7 (4·9–18·4)	8·4 (5·7–11·5)	8·3 (5·8–11·0)
Vanuatu	Diarrheal diseases	62·7 (38·5–81·8)	23·0 (13·3–32·9)	41·3 (-1·6–78·3)	18·6 (12·2–23·9)

Vanuatu	Lower respiratory infections	57·2 (41·8–71·8)	36·9 (1·7–68·0)	16·8 (10·5–22·4)	24·9 (16·3–32·7)
Vanuatu	Malaria	5·5 (-3·3–29·3)	4·4 (-2·7–21·6)	--	1·6 (-0·76–11·6)
Vanuatu	Measles	48·3 (25·5–57·5)	20·1 (10·1–25·7)	14·2 (5·7–24·9)	25·4 (12·0–32·4)
Southeast Asia	All causes	12·9 (9·6–16·1)	8·0 (3·3–11·6)	6·3 (4·3–8·5)	5·4 (3·8–7·2)
Southeast Asia	Diarrheal diseases	73·3 (50·6–87·1)	32·8 (20·4–43·7)	49·4 (-2·2–84·1)	25·5 (17·3–31·7)
Southeast Asia	Lower respiratory infections	58·3 (45·2–67·0)	39·4 (2·3–63·4)	20·6 (13·8–26·5)	29·0 (19·6–36·2)
Southeast Asia	Malaria	21·1 (-12·4–58·8)	16·5 (-9·2–43·8)	--	7·1 (-2·9–29·7)
Southeast Asia	Measles	58·9 (33·7–68·0)	28·4 (15·0–35·6)	19·3 (8·0–33·2)	30·7 (15·0–38·4)
Cambodia	All causes	16·3 (11·5–21·9)	10·8 (2·3–17·7)	7·5 (5·5–10·1)	7·3 (4·5–10·7)
Cambodia	Diarrheal diseases	74·7 (48·7–89·1)	32·2 (19·7–42·9)	52·4 (-2·3–86·2)	24·1 (16·6–30·2)
Cambodia	Lower respiratory infections	56·5 (44·8–65·8)	38·1 (2·2–61·2)	21·5 (14·1–28·6)	28·2 (19·2–35·5)
Cambodia	Malaria	25·8 (-17·4–68·4)	19·8 (-12·1–49·5)	--	10·2 (-4·2–39·9)
Cambodia	Measles	64·3 (37·8–74·1)	31·9 (17·1–39·7)	22·6 (9·3–38·7)	33·9 (17·2–42·3)
Indonesia	All causes	13·2 (9·4–17·3)	8·2 (4·0–11·3)	6·9 (4·0–9·9)	5·8 (4·0–7·9)
Indonesia	Diarrheal diseases	73·5 (52·7–85·8)	33·9 (21·3–44·8)	48·8 (-2·2–82·5)	28·4 (19·6–34·9)
Indonesia	Lower respiratory infections	62·2 (50·3–70·8)	42·3 (2·7–67·2)	21·8 (14·4–28·6)	34·4 (23·0–43·0)
Indonesia	Malaria	26·4 (-17·0–71·1)	19·7 (-11·7–49·8)	--	11·0 (-5·2–44·6)
Indonesia	Measles	63·8 (37·5–73·8)	31·0 (16·7–39·0)	21·9 (9·1–36·9)	34·5 (17·1–42·9)
Lao People's Democratic Republic	All causes	18·3 (12·3–23·4)	11·5 (3·7–18·8)	8·6 (5·8–11·5)	7·3 (4·9–10·2)
Lao People's Democratic Republic	Diarrheal diseases	73·6 (46·4–90·4)	30·6 (18·6–41·3)	51·0 (-2·2–87·3)	22·3 (15·3–28·5)
Lao People's Democratic Republic	Lower respiratory infections	64·9 (50·0–77·2)	43·1 (2·4–73·4)	23·6 (15·3–30·8)	29·8 (19·7–38·5)
Lao People's Democratic Republic	Malaria	21·6 (-12·6–62·0)	16·1 (-9·0–43·3)	--	8·4 (-3·4–35·0)
Lao People's Democratic Republic	Measles	62·0 (35·5–71·7)	30·7 (16·1–38·6)	20·1 (8·5–34·2)	32·9 (15·7–40·9)
Malaysia	All causes	6·5 (4·7–8·9)	4·0 (1·2–6·7)	3·0 (2·4–3·9)	2·5 (1·9–3·5)
Malaysia	Diarrheal diseases	62·3 (36·8–81·8)	23·5 (13·6–32·6)	44·9 (-1·9–79·8)	14·8 (9·5–19·3)
Malaysia	Lower respiratory infections	52·0 (39·0–64·2)	33·7 (1·8–58·9)	18·3 (11·5–25·0)	23·7 (19·8–28·4)
Malaysia	Malaria	18·3 (-9·6–53·2)	14·4 (-7·5–39·9)	--	5·6 (-2·2–23·7)
Malaysia	Measles	52·0 (26·8–62·4)	22·9 (11·0–29·4)	21·2 (8·8–37·3)	21·9 (9·7–28·2)
Maldives	All causes	6·6 (4·6–8·2)	4·2 (1·9–5·9)	3·4 (2·1–4·7)	2·4 (1·6–3·4)

Maldives	Diarrheal diseases	70·6 (47·5–85·2)	31·5 (19·1–42·2)	47·2 (-2·0–82·1)	21·9 (14·4–27·8)
Maldives	Lower respiratory infections	51·0 (39·0–59·8)	34·9 (2·0–56·1)	17·1 (10·7–22·5)	24·3 (16·8–30·7)
Maldives	Malaria	0 (0–0)	0 (0–0)	--	0 (0–0)
Maldives	Measles	54·3 (29·7–65·1)	25·7 (13·2–32·6)	19·9 (8·3–34·2)	23·9 (11·4–30·8)
Mauritius	All causes	5·2 (3·7–6·5)	2·9 (1·2–4·4)	3·2 (2·0–3·9)	1·4 (0·93–1·9)
Mauritius	Diarrheal diseases	66·1 (32·0–84·9)	21·1 (11·8–30·1)	52·3 (-2·6–83·8)	12·3 (7·5–16·7)
Mauritius	Lower respiratory infections	47·6 (37·4–58·0)	29·4 (1·4–55·0)	22·0 (15·2–28·2)	16·2 (10·7–21·7)
Mauritius	Malaria	0 (0–0)	0 (0–0)	--	0 (0–0)
Mauritius	Measles	51·7 (26·8–66·3)	20·1 (9·8–26·0)	28·2 (12·0–47·7)	16·7 (7·6–22·1)
Myanmar	All causes	12·2 (8·1–16·4)	7·4 (2·4–12·6)	5·4 (3·7–7·7)	4·6 (2·9–6·9)
Myanmar	Diarrheal diseases	68·5 (40·9–85·7)	27·9 (16·6–37·7)	47·1 (-1·7–83·1)	18·8 (12·3–24·5)
Myanmar	Lower respiratory infections	49·2 (35·8–61·3)	32·8 (1·7–57·9)	17·4 (11·3–23·8)	20·5 (12·7–28·1)
Myanmar	Malaria	19·6 (-10·9–56·3)	15·7 (-8·4–42·2)	--	6·1 (-2·4–27·3)
Myanmar	Measles	56·9 (32·0–66·2)	27·8 (14·6–35·0)	17·5 (7·1–30·6)	28·9 (13·9–36·7)
Philippines	All causes	14·4 (10·8–17·9)	9·2 (3·6–12·9)	7·3 (4·9–9·3)	5·6 (4·0–7·4)
Philippines	Diarrheal diseases	76·5 (52·6–91·8)	34·0 (21·3–46·0)	52·2 (-2·7–88·5)	23·3 (15·7–29·8)
Philippines	Lower respiratory infections	64·6 (50·6–74·6)	44·0 (2·5–70·7)	22·6 (14·9–29·5)	31·1 (22·5–38·3)
Philippines	Malaria	26·3 (-17·3–71·1)	20·2 (-12·3–51·3)	--	10·3 (-5·0–43·3)
Philippines	Measles	60·8 (34·7–69·8)	30·5 (15·8–37·6)	20·0 (8·5–34·1)	30·8 (14·9–38·3)
Seychelles	All causes	8·3 (5·3–11·7)	4·9 (1·2–9·3)	3·6 (2·8–4·5)	2·6 (1·9–3·7)
Seychelles	Diarrheal diseases	54·8 (25·2–80·4)	14·7 (7·6–21·4)	40·4 (-1·5–78·1)	10·6 (6·3–14·1)
Seychelles	Lower respiratory infections	42·8 (29·8–56·8)	26·0 (1·1–51·9)	13·5 (8·5–17·9)	15·9 (11·1–20·6)
Seychelles	Malaria	0 (0–0)	0 (0–0)	--	0 (0–0)
Seychelles	Measles	34·1 (17·2–40·3)	13·0 (6·5–16·7)	11·7 (4·8–20·4)	14·4 (6·7–18·9)
Sri Lanka	All causes	4·3 (3·1–5·5)	2·8 (1·0–4·1)	2·3 (1·7–2·9)	1·5 (1·0–2·1)
Sri Lanka	Diarrheal diseases	67·5 (44·3–81·0)	31·2 (19·9–41·7)	48·8 (-2·7–78·4)	17·5 (11·6–22·6)
Sri Lanka	Lower respiratory infections	54·0 (42·0–62·8)	37·7 (2·5–59·2)	22·5 (15·0–30·0)	23·7 (16·2–31·0)
Sri Lanka	Malaria	0 (0–0)	0 (0–0)	--	0 (0–0)
Sri Lanka	Measles	61·5 (35·5–71·4)	31·5 (16·8–39·0)	27·5 (12·2–45·8)	24·0 (11·6–30·9)

Thailand	All causes	9·8 (7·4–12·3)	5·5 (2·4–8·2)	4·5 (3·0–5·7)	3·8 (2·8–5·0)
Thailand	Diarrheal diseases	63·4 (37·3–84·3)	22·5 (12·8–32·0)	43·7 (-1·6–81·5)	17·6 (11·5–23·0)
Thailand	Lower respiratory infections	58·2 (42·4–74·1)	36·5 (1·8–67·0)	18·4 (11·0–25·8)	26·8 (19·6–34·9)
Thailand	Malaria	15·4 (-7·4–48·0)	11·4 (-5·5–32·6)	--	5·3 (-2·1–23·1)
Thailand	Measles	41·9 (21·0–51·6)	15·9 (7·8–20·7)	14·6 (5·7–26·1)	19·6 (8·9–25·7)
Timor-Leste	All causes	22·3 (16·5–27·8)	16·3 (5·1–24·6)	12·8 (9·5–16·7)	10·4 (7·0–14·3)
Timor-Leste	Diarrheal diseases	86·3 (65·0–95·3)	46·0 (32·1–58·4)	63·0 (-4·4–93·2)	33·6 (24·7–40·7)
Timor-Leste	Lower respiratory infections	77·1 (65·9–84·6)	54·7 (3·8–81·1)	36·4 (25·6–45·8)	43·5 (31·8–52·3)
Timor-Leste	Malaria	24·4 (-24·3–84·0)	17·6 (-14·3–59·9)	--	13·1 (-8·8–62·4)
Timor-Leste	Measles	78·9 (51·4–88·8)	44·9 (25·1–54·6)	37·8 (17·7–58·6)	42·3 (21·0–51·3)
Viet Nam	All causes	6·2 (4·1–8·3)	3·6 (0·90–6·3)	2·1 (1·3–3·2)	2·9 (1·9–4·4)
Viet Nam	Diarrheal diseases	62·3 (36·7–80·2)	22·4 (13·2–31·7)	42·9 (-1·7–77·2)	17·7 (11·7–22·8)
Viet Nam	Lower respiratory infections	41·3 (26·6–54·4)	26·0 (1·1–48·2)	13·3 (6·9–20·5)	19·6 (12·4–28·4)
Viet Nam	Malaria	18·6 (-9·9–55·7)	13·7 (-7·2–37·6)	--	7·2 (-2·9–31·6)
Viet Nam	Measles	51·5 (27·2–59·7)	22·7 (11·2–28·9)	16·2 (6·7–28·8)	26·2 (12·2–33·1)
**Sub-Saharan Africa**	**All causes**	**23·4 (11·3–32·8)**	**15·1 (5·6–22·3)**	**10·7 (6·1–15·6)**	**10·0 (6·3–14·6)**
**Sub-Saharan Africa**	**Diarrheal diseases**	**77·3 (55·2–90·2)**	**37·0 (24·5–48·6)**	**53·1 (-3·0–87·0)**	**27·0 (19·4–33·3)**
**Sub-Saharan Africa**	**Lower respiratory infections**	**65·2 (52·5–73·9)**	**44·7 (2·9–69·7)**	**22·9 (15·1–30·1)**	**37·2 (30·6–43·2)**
**Sub-Saharan Africa**	**Malaria**	**26·3 (-17·7–69·4)**	**18·9 (-11·2–48·0)**	**--**	**12·4 (-6·3–45·8)**
**Sub-Saharan Africa**	**Measles**	**66·2 (39·5–75·8)**	**33·9 (18·1–42·2)**	**24·3 (10·4–40·2)**	**35·4 (17·4–43·7)**
Central Sub-Saharan Africa	All causes	23·7 (9·7–37·4)	14·9 (3·3–24·4)	8·1 (5·1–12·3)	11·4 (6·1–19·2)
Central Sub-Saharan Africa	Diarrheal diseases	77·2 (54·4–91·3)	35·1 (22·4–47·0)	51·0 (-2·4–87·7)	28·0 (19·9–34·6)
Central Sub-Saharan Africa	Lower respiratory infections	69·7 (55·3–80·2)	47·0 (2·8–74·9)	22·5 (14·0–30·4)	40·1 (31·9–47·6)
Central Sub-Saharan Africa	Malaria	26·7 (-17·4–71·5)	18·4 (-10·5–47·4)	--	13·4 (-6·8–49·6)
Central Sub-Saharan Africa	Measles	65·6 (38·3–75·7)	31·6 (16·6–39·9)	21·6 (8·7–37·0)	37·4 (18·4–45·8)
Angola	All causes	19·9 (10·0–28·5)	12·8 (4·4–19·3)	7·5 (4·1–11·9)	9·2 (5·8–12·8)
Angola	Diarrheal diseases	76·7 (57·1–90·6)	36·2 (23·0–48·3)	47·2 (-2·1–85·6)	29·4 (21·1–36·2)
Angola	Lower respiratory infections	71·2 (55·5–81·3)	48·7 (3·0–76·3)	19·1 (11·9–25·6)	41·4 (32·7–48·7)
Angola	Malaria	26·4 (-16·9–71·9)	18·8 (-10·8–48·4)	--	12·2 (-5·7–47·8)

Angola	Measles	62·5 (36·0–71·8)	30·6 (16·1–38·8)	16·4 (6·6–28·6)	36·4 (17·9–44·8)
Central African Republic	All causes	32·6 (18·7–43·1)	18·8 (9·3–25·8)	16·0 (5·0–27·1)	14·4 (9·9–17·9)
Central African Republic	Diarrheal diseases	77·5 (55·4–91·3)	36·0 (23·0–48·1)	51·0 (-2·4–87·4)	28·5 (20·2–35·0)
Central African Republic	Lower respiratory infections	69·4 (55·4–78·3)	47·4 (3·0–72·7)	21·7 (13·6–29·1)	41·0 (34·2–47·3)
Central African Republic	Malaria	27·6 (-18·9–72·0)	19·2 (-11·3–48·7)	--	14·0 (-7·4–50·6)
Central African Republic	Measles	67·4 (40·3–76·8)	34·3 (18·4–43·0)	20·6 (8·3–35·6)	39·0 (19·4–47·4)
Congo	All causes	16·9 (7·1–26·6)	9·7 (3·2–16·6)	7·8 (3·3–13·5)	6·1 (3·5–9·9)
Congo	Diarrheal diseases	71·3 (44·2–89·3)	27·4 (16·4–38·1)	49·9 (-2·2–86·5)	20·4 (13·7–26·3)
Congo	Lower respiratory infections	60·8 (47·6–73·1)	38·7 (1·9–67·2)	20·5 (13·3–27·8)	29·5 (23·1–36·4)
Congo	Malaria	19·5 (-10·2–57·8)	13·9 (-7·1–38·0)	--	8·0 (-2·9–33·3)
Congo	Measles	52·6 (27·5–63·4)	22·0 (10·9–28·3)	18·4 (7·3–32·1)	26·3 (12·2–33·9)
Democratic Republic of the Congo	All causes	24·2 (7·9–41·5)	15·4 (2·1–27·6)	7·3 (4·6–11·1)	12·0 (5·5–22·6)
Democratic Republic of the Congo	Diarrheal diseases	78·4 (53·5–92·6)	34·7 (22·5–47·2)	54·0 (-2·5–89·9)	27·6 (19·7–34·5)
Democratic Republic of the Congo	Lower respiratory infections	69·5 (55·0–81·3)	46·7 (2·8–74·6)	23·9 (14·6–32·4)	39·8 (30·9–48·4)
Democratic Republic of the Congo	Malaria	27·1 (-17·6–71·9)	18·6 (-10·6–47·6)	--	13·7 (-6·9–50·2)
Democratic Republic of the Congo	Measles	66·3 (38·8–76·4)	31·9 (16·7–40·2)	22·5 (9·1–38·3)	37·7 (18·5–46·2)
Equatorial Guinea	All causes	11·1 (2·7–22·8)	6·2 (0·28–13·5)	3·0 (1·6–5·0)	4·7 (1·7–11·2)
Equatorial Guinea	Diarrheal diseases	62·1 (34·3–85·2)	18·3 (10·2–26·5)	42·5 (-1·3–81·9)	18·1 (11·8–23·7)
Equatorial Guinea	Lower respiratory infections	52·3 (40·0–67·9)	30·2 (1·1–62·1)	15·4 (9·3–21·4)	24·6 (16·8–32·0)
Equatorial Guinea	Malaria	14·2 (-6·8–45·9)	9·1 (-4·1–26·4)	--	6·5 (-2·5–27·7)
Equatorial Guinea	Measles	44·6 (22·4–55·0)	14·2 (6·8–18·8)	13·8 (5·1–25·5)	25·3 (12·0–32·5)
Gabon	All causes	11·4 (5·4–18·6)	5·9 (1·9–11·0)	4·3 (2·1–7·7)	4·4 (2·7–7·0)
Gabon	Diarrheal diseases	58·2 (33·0–83·1)	17·6 (9·7–26·3)	38·6 (-1·2–79·0)	16·4 (10·5–21·9)
Gabon	Lower respiratory infections	48·9 (36·2–65·6)	28·8 (1·1–58·8)	12·1 (7·3–16·7)	23·2 (17·6–29·6)
Gabon	Malaria	12·4 (-5·7–40·2)	8·5 (-3·7–24·8)	--	4·9 (-1·8–21·4)
Gabon	Measles	38·5 (18·9–46·9)	13·5 (6·5–17·9)	10·4 (3·9–19·2)	20·9 (9·7–27·4)
Eastern Sub-Saharan Africa	All causes	19·4 (11·8–26·6)	11·9 (5·8–16·9)	9·0 (5·1–12·6)	8·4 (6·0–11·0)
Eastern Sub-Saharan Africa	Diarrheal diseases	74·3 (53·3–87·6)	34·6 (22·6–45·8)	49·5 (-2·6–84·1)	27·2 (19·6–33·4)
Eastern Sub-Saharan Africa	Lower respiratory infections	61·7 (49·5–70·2)	41·4 (2·5–66·0)	20·0 (13·2–26·2)	34·5 (26·4–41·2)

Eastern Sub-Saharan Africa	Malaria	23·9 (-15·0–65·9)	16·6 (-9·4–43·4)	--	11·3 (-5·5–43·8)
Eastern Sub-Saharan Africa	Measles	64·7 (38·1–74·3)	32·2 (17·0–40·4)	22·7 (9·7–37·9)	34·9 (17·2–43·3)
Burundi	All causes	25·8 (5·9–40·6)	18·0 (2·2–30·2)	8·0 (4·4–13·8)	13·3 (4·5–24·1)
Burundi	Diarrheal diseases	81·9 (68·2–91·7)	44·4 (30·2–57·3)	47·7 (-2·4–85·5)	38·4 (28·8–45·4)
Burundi	Lower respiratory infections	73·1 (60·4–80·9)	52·7 (3·9–76·9)	19·3 (12·3–26·0)	47·6 (37·7–55·0)
Burundi	Malaria	32·3 (-25·8–78·6)	22·8 (-15·4–55·2)	--	17·2 (-9·4–58·8)
Burundi	Measles	73·0 (45·5–80·5)	40·2 (22·5–49·1)	17·5 (7·1–30·2)	46·9 (23·9–55·6)
Comoros	All causes	20·3 (14·5–26·1)	12·1 (5·9–16·9)	11·9 (6·1–17·0)	8·3 (5·9–11·1)
Comoros	Diarrheal diseases	78·8 (54·5–91·8)	34·6 (22·2–47·4)	56·5 (-3·1–89·7)	27·9 (19·7–34·9)
Comoros	Lower respiratory infections	66·1 (54·5–74·1)	43·5 (2·5–70·4)	27·8 (19·1–35·2)	34·6 (24·3–42·7)
Comoros	Malaria	20·6 (-15·3–69·3)	14·1 (-8·7–44·1)	--	10·1 (-5·8–47·2)
Comoros	Measles	65·9 (38·1–77·8)	28·9 (14·9–37·3)	28·0 (11·6–46·5)	35·2 (17·1–44·0)
Djibouti	All causes	21·8 (15·2–28·1)	13·9 (8·6–18·2)	15·7 (7·7–22·5)	7·0 (4·6–9·4)
Djibouti	Diarrheal diseases	83·4 (56·0–93·3)	41·6 (28·0–54·0)	65·2 (-4·8–92·0)	25·3 (17·7–31·8)
Djibouti	Lower respiratory infections	67·3 (57·5–74·1)	46·0 (2·9–71·3)	38·2 (28·4–46·5)	30·9 (21·0–39·4)
Djibouti	Malaria	28·2 (-18·0–74·8)	21·0 (-12·0–53·4)	--	12·5 (-5·3–49·2)
Djibouti	Measles	75·8 (47·0–87·6)	38·8 (20·6–48·6)	43·4 (20·4–64·9)	33·8 (15·9–42·7)
Eritrea	All causes	28·9 (22·3–34·4)	20·2 (12·2–25·1)	18·2 (11·5–23·2)	10·7 (8·1–13·6)
Eritrea	Diarrheal diseases	84·6 (62·4–94·3)	44·8 (31·0–57·6)	61·2 (-4·8–91·8)	27·6 (19·6–34·6)
Eritrea	Lower respiratory infections	76·0 (64·2–82·6)	54·7 (4·0–78·8)	33·0 (22·4–42·5)	41·9 (35·8–47·5)
Eritrea	Malaria	32·8 (-25·3–80·7)	25·3 (-17·3–61·1)	--	14·4 (-7·8–53·9)
Eritrea	Measles	74·7 (47·8–84·5)	42·0 (23·8–51·1)	32·2 (14·6–50·9)	37·6 (18·8–45·7)
Ethiopia	All causes	14·7 (9·5–19·2)	9·3 (4·8–13·4)	7·3 (4·7–10·1)	6·4 (4·3–8·2)
Ethiopia	Diarrheal diseases	76·6 (54·7–88·1)	36·3 (23·6–47·9)	52·7 (-3·0–85·2)	28·1 (20·5–34·3)
Ethiopia	Lower respiratory infections	66·3 (55·3–74·1)	45·6 (3·1–69·4)	25·7 (17·2–33·3)	36·6 (27·9–43·9)
Ethiopia	Malaria	24·4 (-16·5–65·2)	17·6 (-10·6–44·8)	--	11·2 (-5·4–43·4)
Ethiopia	Measles	66·6 (39·9–76·6)	33·0 (17·9–41·4)	24·5 (10·6–40·5)	36·5 (18·0–44·6)
Kenya	All causes	19·1 (11·8–25·1)	10·9 (4·8–16·2)	8·5 (3·8–12·9)	8·2 (6·3–10·5)
Kenya	Diarrheal diseases	69·1 (48·0–85·2)	28·9 (17·7–40·2)	43·9 (-2·0–80·6)	24·9 (17·2–31·1)

Kenya	Lower respiratory infections	52·3 (40·7–62·2)	34·1 (1·9–59·0)	14·9 (9·6–19·6)	28·0 (19·5–34·6)
Kenya	Malaria	20·3 (-12·0–58·4)	14·5 (-8·0–38·7)	--	8·4 (-3·7–34·9)
Kenya	Measles	56·9 (32·1–66·2)	28·4 (15·0–35·9)	17·2 (7·2–29·6)	28·9 (13·9–36·6)
Madagascar	All causes	27·3 (17·8–35·4)	17·1 (9·0–22·6)	13·0 (5·4–20·2)	12·3 (9·7–14·9)
Madagascar	Diarrheal diseases	79·1 (61·7–91·4)	40·3 (27·1–53·3)	49·3 (-2·4–86·6)	32·1 (23·1–39·2)
Madagascar	Lower respiratory infections	57·7 (44·3–66·0)	40·8 (2·6–62·1)	17·4 (10·9–23·9)	34·2 (26·5–41·3)
Madagascar	Malaria	28·2 (-19·2–73·6)	20·7 (-12·8–52·3)	--	13·0 (-6·5–49·6)
Madagascar	Measles	68·3 (41·2–76·8)	36·5 (19·9–45·1)	20·0 (8·3–34·3)	39·3 (19·5–47·8)
Malawi	All causes	14·9 (7·6–23·4)	7·9 (3·7–12·7)	5·5 (2·1–10·0)	6·8 (4·3–11·0)
Malawi	Diarrheal diseases	63·6 (43·1–83·4)	23·5 (13·5–33·3)	37·4 (-1·2–77·9)	24·4 (16·5–30·8)
Malawi	Lower respiratory infections	59·3 (44·7–74·1)	36·9 (1·8–67·3)	12·0 (6·8–17·5)	34·9 (26·0–43·8)
Malawi	Malaria	19·8 (-11·0–59·3)	12·9 (-6·6–35·6)	--	9·6 (-4·1–39·0)
Malawi	Measles	51·4 (27·8–60·1)	20·7 (10·5–26·8)	9·9 (3·9–18·3)	32·5 (15·9–40·6)
Mozambique	All causes	19·2 (10·8–27·6)	11·3 (4·4–17·5)	8·0 (3·9–11·9)	8·5 (6·2–11·7)
Mozambique	Diarrheal diseases	72·9 (52·5–89·8)	30·2 (18·2–42·1)	45·3 (-1·8–85·4)	28·0 (19·1–35·2)
Mozambique	Lower respiratory infections	69·3 (53·3–81·3)	45·1 (2·4–77·2)	19·4 (12·5–25·7)	37·9 (27·0–47·0)
Mozambique	Malaria	22·6 (-13·0–64·3)	15·1 (-7·8–40·2)	--	10·9 (-4·6–42·4)
Mozambique	Measles	57·0 (31·8–66·1)	24·4 (12·4–31·3)	14·7 (5·6–26·5)	34·4 (17·1–42·9)
Rwanda	All causes	17·4 (9·7–25·1)	9·9 (3·3–16·3)	5·5 (3·2–9·2)	9·0 (6·1–11·6)
Rwanda	Diarrheal diseases	66·4 (48·7–84·2)	25·1 (14·7–36·0)	36·5 (-1·2–77·3)	28·6 (20·2–35·5)
Rwanda	Lower respiratory infections	65·8 (52·4–78·0)	40·7 (1·9–71·7)	12·1 (7·3–16·6)	40·8 (31·4–47·6)
Rwanda	Malaria	21·7 (-12·7–63·9)	13·2 (-6·7–36·3)	--	11·9 (-5·8–46·2)
Rwanda	Measles	54·7 (29·8–63·3)	21·3 (10·8–27·4)	9·5 (3·6–17·2)	37·1 (18·2–45·3)
Somalia	All causes	31·0 (22·1–38·1)	17·8 (12·6–21·9)	17·5 (8·5–25·8)	12·4 (8·1–16·1)
Somalia	Diarrheal diseases	71·2 (51·0–81·8)	36·3 (24·9–47·2)	51·1 (-3·2–79·7)	23·6 (16·7–29·8)
Somalia	Lower respiratory infections	44·2 (34·1–52·0)	30·7 (1·9–48·3)	18·6 (12·2–25·2)	23·5 (17·5–29·6)
Somalia	Malaria	25·6 (-15·9–69·4)	19·5 (-10·8–50·6)	--	10·3 (-4·6–41·2)
Somalia	Measles	68·0 (40·3–78·4)	35·8 (18·7–44·9)	27·9 (12·2–45·5)	33·2 (16·1–41·8)
South Sudan	All causes	32·8 (18·9–43·3)	20·6 (10·9–27·9)	21·0 (9·2–30·8)	11·7 (7·9–15·4)

South Sudan	Diarrheal diseases	85·3 (59·4–95·2)	43·2 (29·7–55·5)	65·3 (-4·8–93·8)	26·8 (19·1–33·4)
South Sudan	Lower respiratory infections	76·2 (65·3–82·8)	52·9 (3·5–79·2)	40·2 (29·5–48·9)	37·6 (28·5–45·6)
South Sudan	Malaria	28·2 (-18·5–73·7)	21·2 (-12·5–53·4)	--	12·3 (-5·2–47·9)
South Sudan	Measles	76·1 (47·6–86·9)	39·6 (21·4–48·7)	42·5 (20·0–64·5)	35·1 (16·8–43·7)
Uganda	All causes	13·9 (3·5–26·8)	8·9 (1·1–17·2)	4·4 (2·4–7·2)	5·7 (2·4–12·2)
Uganda	Diarrheal diseases	69·9 (48·8–87·6)	27·8 (16·9–39·2)	43·9 (-1·9–82·9)	24·1 (16·3–30·9)
Uganda	Lower respiratory infections	73·0 (56·4–86·8)	47·1 (2·3–80·4)	19·9 (12·7–26·3)	38·4 (28·5–46·6)
Uganda	Malaria	20·6 (-11·3–61·2)	14·4 (-7·5–39·7)	--	8·9 (-3·6–37·0)
Uganda	Measles	53·0 (28·7–61·9)	22·6 (11·5–29·0)	13·7 (5·4–24·3)	30·2 (14·6–38·0)
United Republic of Tanzania	All causes	13·5 (8·0–19·5)	8·3 (3·1–13·2)	5·1 (3·7–6·7)	6·0 (4·3–8·8)
United Republic of Tanzania	Diarrheal diseases	67·7 (47·4–85·1)	25·8 (15·1–36·6)	41·9 (-1·6–80·9)	25·2 (17·6–31·9)
United Republic of Tanzania	Lower respiratory infections	50·4 (36·8–61·2)	32·1 (1·4–56·7)	12·7 (7·8–17·8)	28·1 (19·8–35·7)
United Republic of Tanzania	Malaria	21·2 (-11·9–61·5)	14·1 (-7·4–38·4)	--	10·2 (-4·2–39·8)
United Republic of Tanzania	Measles	55·3 (30·1–64·0)	23·3 (11·6–29·4)	12·7 (4·9–22·8)	34·0 (16·5–42·4)
Zambia	All causes	18·6 (10·5–26·9)	10·5 (4·7–17·2)	8·2 (4·6–12·8)	8·4 (5·7–11·5)
Zambia	Diarrheal diseases	70·2 (47·1–88·7)	24·8 (14·9–35·2)	44·7 (-1·6–84·8)	27·5 (19·5–34·1)
Zambia	Lower respiratory infections	65·5 (53·9–77·6)	40·0 (1·8–71·4)	17·2 (10·7–23·7)	38·6 (29·4–45·6)
Zambia	Malaria	22·2 (-12·9–65·8)	13·4 (-6·8–37·2)	--	12·4 (-6·1–48·7)
Zambia	Measles	57·0 (30·6–65·6)	21·5 (10·3–27·7)	15·7 (5·9–28·1)	36·0 (17·4–44·1)
Southern Sub-Saharan Africa	All causes	22·3 (17·2–27·0)	14·0 (8·3–18·8)	11·8 (7·8–15·7)	8·0 (5·8–10·5)
Southern Sub-Saharan Africa	Diarrheal diseases	63·5 (43·1–83·3)	21·9 (12·3–31·1)	36·5 (-1·2–77·3)	25·4 (17·5–32·1)
Southern Sub-Saharan Africa	Lower respiratory infections	64·9 (50·4–79·2)	39·7 (1·8–73·9)	14·4 (9·1–19·2)	35·3 (21·8–44·7)
Southern Sub-Saharan Africa	Malaria	21·1 (-11·7–61·4)	14·5 (-7·6–39·4)	--	9·3 (-3·9–37·3)
Southern Sub-Saharan Africa	Measles	48·0 (25·4–56·7)	18·2 (9·1–23·6)	10·5 (4·0–19·1)	29·7 (14·4–37·6)
Botswana	All causes	29·3 (21·4–36·1)	18·1 (9·4–26·7)	17·6 (11·1–22·9)	10·6 (7·0–15·0)
Botswana	Diarrheal diseases	75·5 (47·4–93·1)	26·4 (15·4–37·3)	52·5 (-2·0–90·5)	28·0 (19·8–35·1)
Botswana	Lower respiratory infections	74·6 (61·6–86·9)	45·2 (2·0–82·1)	28·3 (19·1–36·7)	38·6 (23·1–49·5)
Botswana	Malaria	19·2 (-12·6–66·7)	11·7 (-6·8–36·9)	--	10·4 (-5·1–47·5)
Botswana	Measles	57·1 (30·4–68·0)	20·8 (10·2–26·7)	20·8 (7·8–36·7)	32·6 (15·6–41·3)

Eswatini	All causes	20·1 (14·8–26·4)	12·6 (5·0–21·4)	7·3 (5·4–9·7)	8·8 (5·6–12·7)
Eswatini	Diarrheal diseases	58·6 (40·6–80·5)	19·6 (10·9–29·1)	31·1 (-0·81–73·9)	24·0 (16·7–30·9)
Eswatini	Lower respiratory infections	64·4 (48·5–81·4)	38·6 (1·5–75·5)	10·9 (6·6–15·0)	35·1 (21·3–45·3)
Eswatini	Malaria	17·6 (-9·0–56·6)	10·2 (-4·7–29·6)	--	9·4 (-3·9–38·6)
Eswatini	Measles	42·8 (21·7–51·4)	14·5 (6·8–19·3)	6·1 (2·2–11·4)	29·1 (13·9–37·0)
Lesotho	All causes	20·1 (14·3–25·8)	12·1 (4·8–18·5)	8·2 (4·8–12·5)	9·3 (6·1–12·7)
Lesotho	Diarrheal diseases	69·6 (48·7–87·0)	27·1 (15·9–37·8)	40·8 (-1·2–81·8)	28·5 (20·3–35·3)
Lesotho	Lower respiratory infections	67·2 (52·3–80·4)	42·8 (2·0–75·1)	16·4 (10·4–22·2)	37·1 (22·7–47·3)
Lesotho	Malaria	0 (0–0)	0 (0–0)	--	0 (0–0)
Lesotho	Measles	55·1 (30·2–64·1)	22·7 (11·4–29·3)	11·1 (4·1–20·4)	35·4 (17·4–43·8)
Namibia	All causes	22·2 (16·1–27·8)	15·0 (7·4–21·7)	13·4 (9·7–16·7)	6·4 (4·2–9·3)
Namibia	Diarrheal diseases	73·3 (44·1–92·4)	27·0 (16·0–38·3)	52·7 (-2·5–90·0)	19·9 (13·3–25·9)
Namibia	Lower respiratory infections	71·3 (55·1–85·6)	45·4 (2·2–80·4)	27·2 (18·3–34·9)	30·7 (20·2–39·4)
Namibia	Malaria	16·6 (-12·1–62·4)	12·0 (-7·9–41·4)	--	7·0 (-3·4–36·9)
Namibia	Measles	54·6 (29·4–65·3)	23·6 (12·1–30·4)	19·7 (7·9–34·0)	26·8 (12·7–34·4)
South Africa	All causes	21·8 (16·9–26·9)	12·7 (7·3–18·1)	10·1 (6·5–14·3)	9·0 (6·1–11·8)
South Africa	Diarrheal diseases	60·8 (42·1–80·5)	20·0 (11·1–28·7)	32·7 (-0·94–73·5)	26·4 (18·2–32·9)
South Africa	Lower respiratory infections	65·5 (51·0–80·5)	39·0 (1·6–75·5)	12·2 (7·4–16·6)	37·2 (22·0–47·3)
South Africa	Malaria	13·5 (-8·9–53·1)	8·6 (-5·0–31·9)	--	6·4 (-3·4–33·2)
South Africa	Measles	43·9 (22·8–52·3)	14·6 (7·2–19·0)	8·0 (2·9–14·8)	29·0 (14·1–36·8)
Zimbabwe	All causes	23·2 (16·7–28·5)	16·0 (10·3–20·6)	14·5 (9·4–19·3)	6·6 (4·6–8·8)
Zimbabwe	Diarrheal diseases	67·0 (44·7–86·9)	24·6 (14·1–34·7)	41·6 (-1·4–82·3)	23·4 (16·0–29·7)
Zimbabwe	Lower respiratory infections	62·0 (47·4–74·9)	39·2 (1·9–69·7)	15·7 (9·9–20·9)	32·0 (21·3–40·6)
Zimbabwe	Malaria	21·1 (-11·7–61·2)	14·5 (-7·6–39·2)	--	9·3 (-4·0–37·2)
Zimbabwe	Measles	51·0 (27·4–59·7)	21·0 (10·6–27·0)	12·0 (4·7–21·6)	30·0 (14·5–37·9)
Western Sub-Saharan Africa	All causes	25·5 (11·7–35·8)	16·9 (6·0–25·0)	12·2 (6·6–17·8)	10·7 (6·4–16·1)
Western Sub-Saharan Africa	Diarrheal diseases	79·5 (56·8–92·1)	39·2 (26·2–51·1)	56·0 (-3·4–89·2)	26·8 (19·2–33·1)
Western Sub-Saharan Africa	Lower respiratory infections	65·6 (53·5–74·0)	45·8 (3·2–69·0)	24·6 (16·2–32·3)	37·8 (31·7–43·1)
Western Sub-Saharan Africa	Malaria	26·9 (-18·7–69·3)	19·8 (-12·1–49·6)	--	12·4 (-6·5–45·1)

Western Sub-Saharan Africa	Measles	68·0 (41·4–77·3)	36·2 (19·7–44·7)	26·7 (11·7–43·7)	35·2 (17·2–43·2)
Benin	All causes	20·7 (7·3–34·0)	13·7 (3·2–23·3)	7·8 (4·5–12·2)	8·8 (4·8–16·3)
Benin	Diarrheal diseases	75·1 (53·2–90·8)	32·5 (20·2–45·1)	49·1 (-2·3–86·7)	25·6 (17·8–32·6)
Benin	Lower respiratory infections	68·8 (54·9–79·2)	45·7 (2·6–73·2)	20·1 (12·6–27·2)	39·2 (32·8–45·1)
Benin	Malaria	24·5 (-15·1–67·2)	17·6 (-9·9–45·6)	--	10·7 (-4·6–41·7)
Benin	Measles	60·0 (34·0–69·6)	28·1 (14·6–35·6)	18·2 (7·3–31·8)	32·9 (16·3–41·2)
Burkina Faso	All causes	19·3 (8·6–30·7)	13·0 (2·7–22·1)	7·6 (4·8–10·6)	7·4 (4·8–13·0)
Burkina Faso	Diarrheal diseases	73·9 (49·9–86·9)	35·7 (23·3–47·4)	52·0 (-2·9–84·8)	22·8 (15·5–28·8)
Burkina Faso	Lower respiratory infections	51·4 (40·7–61·5)	35·6 (2·3–56·0)	20·1 (13·2–26·9)	25·6 (17·5–33·5)
Burkina Faso	Malaria	24·9 (-15·6–67·9)	19·5 (-11·6–49·4)	--	9·1 (-3·9–39·1)
Burkina Faso	Measles	65·0 (37·7–74·0)	34·0 (18·2–42·1)	25·4 (11·0–43·1)	30·6 (14·3–38·3)
Cabo Verde	All causes	9·8 (6·2–14·1)	5·6 (3·0–8·5)	5·7 (3·2–9·0)	2·5 (1·7–3·6)
Cabo Verde	Diarrheal diseases	62·9 (33·9–86·0)	20·0 (10·9–29·2)	45·5 (-2·0–83·3)	13·1 (8·0–17·7)
Cabo Verde	Lower respiratory infections	53·0 (38·7–67·9)	33·6 (1·5–63·4)	17·6 (11·4–23·2)	19·6 (13·1–25·6)
Cabo Verde	Malaria	13·2 (-6·7–38·3)	11·4 (-5·7–31·6)	--	2·4 (-1·0–10·6)
Cabo Verde	Measles	40·9 (21·3–49·6)	16·7 (8·5–21·3)	14·8 (6·1–26·0)	17·1 (8·2–22·4)
Cameroon	All causes	21·9 (10·7–32·9)	12·2 (4·5–19·9)	8·9 (3·9–15·8)	9·4 (5·8–13·8)
Cameroon	Diarrheal diseases	70·5 (47·0–89·0)	27·3 (16·4–38·6)	45·8 (-1·8–84·9)	23·5 (16·2–29·9)
Cameroon	Lower respiratory infections	60·9 (47·2–72·6)	38·6 (1·8–66·0)	16·5 (10·2–22·7)	34·0 (28·1–39·8)
Cameroon	Malaria	21·7 (-12·1–63·1)	14·5 (-7·3–39·4)	--	10·3 (-4·2–41·1)
Cameroon	Measles	55·8 (30·0–65·8)	24·0 (11·8–31·0)	16·3 (6·2–29·0)	31·3 (15·2–39·3)
Chad	All causes	40·2 (27·9–48·8)	23·1 (17·2–27·5)	25·3 (7·3–37·5)	14·2 (11·3–17·4)
Chad	Diarrheal diseases	80·4 (56·0–92·1)	39·5 (26·3–51·8)	58·6 (-3·8–90·0)	24·6 (16·9–30·5)
Chad	Lower respiratory infections	63·3 (51·4–71·4)	44·0 (2·8–66·5)	27·0 (18·1–34·9)	33·3 (27·7–38·7)
Chad	Malaria	27·5 (-17·9–73·3)	20·9 (-12·6–53·0)	--	11·6 (-5·3–47·4)
Chad	Measles	71·2 (42·9–81·9)	37·5 (20·0–46·5)	32·8 (15·0–53·1)	33·6 (15·9–41·9)
Côte d'Ivoire	All causes	16·9 (6·6–26·9)	10·7 (2·3–18·4)	6·9 (3·9–10·3)	6·3 (3·5–9·7)
Côte d'Ivoire	Diarrheal diseases	72·8 (45·9–90·2)	29·0 (17·5–40·2)	51·2 (-2·5–87·4)	20·1 (13·3–25·9)
Côte d'Ivoire	Lower respiratory infections	59·3 (45·9–70·9)	38·7 (2·0–65·8)	20·5 (13·4–26·6)	28·0 (21·4–34·1)

Côte d'Ivoire	Malaria	20·2 (-11·1–59·2)	15·1 (-8·0–40·7)	--	7·6 (-3·0–33·0)
Côte d'Ivoire	Measles	54·8 (29·7–65·4)	24·6 (12·5–31·5)	19·5 (8·0–33·4)	26·3 (12·6–33·7)
Gambia	All causes	14·3 (9·8–18·3)	9·3 (3·3–13·8)	6·2 (4·8–7·8)	5·4 (4·2–6·8)
Gambia	Diarrheal diseases	65·2 (40·8–81·6)	26·6 (16·0–37·6)	45·1 (-2·2–79·0)	17·4 (11·5–22·8)
Gambia	Lower respiratory infections	62·7 (47·6–75·1)	42·0 (2·3–70·0)	20·2 (12·5–27·7)	30·0 (25·0–35·4)
Gambia	Malaria	19·2 (-10·8–53·5)	15·7 (-8·7–41·5)	--	5·5 (-2·2–23·0)
Gambia	Measles	53·0 (29·0–62·6)	25·4 (13·1–32·1)	18·6 (7·9–31·9)	23·8 (11·1–30·7)
Ghana	All causes	11·8 (4·5–20·1)	8·3 (2·2–15·0)	5·1 (3·2–7·5)	3·4 (1·7–7·0)
Ghana	Diarrheal diseases	68·2 (41·9–85·9)	26·3 (15·3–36·5)	48·9 (-2·4–83·5)	16·4 (10·4–21·9)
Ghana	Lower respiratory infections	53·6 (38·6–67·1)	34·8 (1·9–58·9)	19·0 (11·8–25·7)	23·7 (18·6–29·2)
Ghana	Malaria	18·5 (-10·1–54·7)	14·5 (-7·7–39·9)	--	5·9 (-2·2–26·1)
Ghana	Measles	52·2 (28·0–62·4)	23·6 (12·1–30·1)	19·3 (7·9–32·7)	23·1 (10·9–29·5)
Guinea	All causes	23·1 (12·3–33·8)	14·6 (4·1–23·7)	8·9 (5·8–12·3)	10·4 (6·1–15·8)
Guinea	Diarrheal diseases	76·1 (51·7–90·3)	33·3 (20·9–45·1)	52·8 (-2·7–87·5)	24·8 (17·5–31·3)
Guinea	Lower respiratory infections	64·0 (51·4–73·7)	42·6 (2·4–68·5)	22·6 (14·7–30·0)	34·3 (27·9–40·4)
Guinea	Malaria	24·5 (-14·6–67·8)	17·3 (-9·6–45·1)	--	11·3 (-5·1–44·6)
Guinea	Measles	62·8 (35·7–73·4)	29·6 (15·4–37·6)	23·0 (9·6–39·5)	32·7 (15·6–41·0)
Guinea-Bissau	All causes	17·8 (12·0–23·2)	10·8 (5·3–15·2)	7·5 (4·6–11·3)	8·1 (5·8–10·6)
Guinea-Bissau	Diarrheal diseases	73·7 (53·1–88·9)	32·5 (20·4–44·4)	46·4 (-1·9–84·7)	26·7 (18·5–33·4)
Guinea-Bissau	Lower respiratory infections	68·0 (53·5–77·8)	45·6 (2·7–71·7)	18·2 (10·7–25·0)	40·5 (35·7–46·0)
Guinea-Bissau	Malaria	23·4 (-14·2–64·3)	17·2 (-9·8–44·6)	--	9·6 (-4·0–38·0)
Guinea-Bissau	Measles	59·1 (33·5–68·6)	28·2 (14·8–35·6)	16·8 (6·7–29·4)	32·6 (16·0–40·8)
Liberia	All causes	18·8 (9·4–28·2)	11·4 (3·7–19·3)	7·3 (4·2–10·6)	7·3 (4·8–10·7)
Liberia	Diarrheal diseases	69·7 (44·8–88·9)	27·0 (16·1–38·2)	46·3 (-1·9–85·9)	20·6 (13·8–26·8)
Liberia	Lower respiratory infections	63·3 (47·2–77·3)	41·4 (2·0–72·2)	18·3 (11·5–24·4)	30·3 (21·2–37·9)
Liberia	Malaria	19·7 (-10·8–56·9)	14·4 (-7·6–38·4)	--	7·7 (-3·0–31·9)
Liberia	Measles	53·1 (28·5–62·7)	23·9 (12·0–30·4)	15·4 (6·2–27·3)	27·8 (13·2–35·4)
Mali	All causes	20·6 (11·3–29·9)	12·9 (5·2–19·6)	9·4 (5·9–13·6)	7·4 (4·9–12·6)
Mali	Diarrheal diseases	76·9 (52·7–91·6)	34·8 (22·6–47·2)	54·8 (-3·2–89·0)	21·3 (14·5–27·4)

Mali	Lower respiratory infections	62·0 (49·3–72·5)	42·1 (2·5–66·5)	23·5 (15·5–31·3)	31·0 (26·4–35·9)
Mali	Malaria	23·9 (-14·1–65·4)	18·4 (-10·4–47·3)	--	8·9 (-3·8–36·6)
Mali	Measles	62·2 (35·5–72·7)	31·3 (16·5–39·6)	24·8 (10·8–41·5)	28·1 (13·1–35·7)
Mauritania	All causes	17·2 (9·9–23·3)	10·2 (5·1–14·5)	9·8 (4·2–15·5)	5·3 (3·8–7·3)
Mauritania	Diarrheal diseases	73·0 (45·6–89·3)	31·1 (19·4–42·8)	52·7 (-2·9–86·8)	17·9 (11·9–23·2)
Mauritania	Lower respiratory infections	53·6 (39·8–65·3)	36·3 (2·1–60·3)	20·4 (13·1–27·9)	23·9 (19·2–29·6)
Mauritania	Malaria	19·0 (-10·8–53·2)	15·1 (-8·2–39·5)	--	6·3 (-2·5–26·5)
Mauritania	Measles	58·5 (32·9–69·1)	28·8 (15·1–36·4)	23·2 (9·9–38·9)	25·5 (11·9–32·7)
Niger	All causes	34·9 (17·3–45·5)	25·3 (10·0–34·3)	16·7 (10·5–23·5)	15·9 (9·6–22·8)
Niger	Diarrheal diseases	86·2 (69·4–94·0)	50·9 (36·6–63·3)	59·5 (-4·5–90·7)	33·8 (24·4–40·6)
Niger	Lower respiratory infections	74·0 (63·7–80·2)	55·3 (4·5–75·8)	28·6 (18·8–37·1)	46·5 (40·1–51·6)
Niger	Malaria	36·1 (-31·4–84·4)	27·3 (-20·4–63·7)	--	18·1 (-9·8–62·5)
Niger	Measles	78·1 (51·5–86·6)	47·8 (27·8–57·3)	29·8 (13·3–47·6)	42·9 (21·5–51·5)
Nigeria	All causes	26·1 (9·9–37·6)	17·9 (5·1–27·5)	12·3 (6·4–17·9)	11·4 (5·9–18·4)
Nigeria	Diarrheal diseases	80·8 (58·8–92·7)	40·8 (28·0–52·7)	56·9 (-3·5–90·1)	28·9 (20·8–35·2)
Nigeria	Lower respiratory infections	66·6 (55·2–75·4)	46·9 (3·4–70·2)	25·6 (16·6–33·9)	40·1 (33·7–45·4)
Nigeria	Malaria	28·2 (-20·7–71·1)	20·5 (-12·8–50·8)	--	13·7 (-7·5–47·9)
Nigeria	Measles	74·8 (47·7–83·6)	41·8 (23·4–50·8)	30·8 (13·9–49·7)	41·8 (20·8–50·1)
Sao Tome and Principe	All causes	16·1 (12·2–20·7)	11·7 (7·1–16·4)	10·0 (7·3–13·3)	4·3 (2·9–6·0)
Sao Tome and Principe	Diarrheal diseases	63·0 (37·5–83·2)	21·5 (12·1–30·9)	42·6 (-1·6–80·3)	18·8 (12·3–24·3)
Sao Tome and Principe	Lower respiratory infections	52·2 (38·8–64·7)	32·0 (1·4–58·6)	14·7 (9·1–20·2)	25·8 (19·7–32·1)
Sao Tome and Principe	Malaria	15·6 (-7·7–48·3)	11·4 (-5·6–32·1)	--	5·7 (-2·1–24·8)
Sao Tome and Principe	Measles	45·0 (22·9–54·4)	17·4 (8·6–22·8)	15·0 (5·9–26·7)	22·2 (10·4–29·1)
Senegal	All causes	11·4 (7·3–14·9)	6·9 (3·5–9·9)	5·7 (3·6–7·6)	3·7 (2·6–5·2)
Senegal	Diarrheal diseases	64·4 (37·6–82·6)	25·0 (14·7–36·1)	45·4 (-2·3–79·4)	15·9 (9·9–21·5)
Senegal	Lower respiratory infections	59·2 (42·5–74·5)	38·9 (1·8–68·2)	20·1 (12·1–28·1)	26·5 (20·0–33·2)
Senegal	Malaria	17·1 (-9·0–50·0)	13·6 (-7·1–37·1)	--	5·1 (-2·0–22·3)
Senegal	Measles	51·2 (26·8–60·9)	23·3 (11·6–29·5)	20·0 (8·5–34·7)	21·4 (9·5–27·9)
Sierra Leone	All causes	25·3 (11·5–38·4)	15·1 (4·4–24·9)	11·6 (5·0–17·6)	9·5 (6·2–15·1)

Sierra Leone	Diarrheal diseases	75·9 (49·3–92·9)	31·6 (19·2–43·7)	52·0 (-2·3–89·9)	23·9 (16·4–30·4)
Sierra Leone	Lower respiratory infections	70·9 (56·0–84·0)	46·8 (2·4–79·5)	24·5 (16·4–32·0)	34·2 (23·0–43·2)
Sierra Leone	Malaria	22·8 (-12·8–65·4)	16·0 (-8·4–42·7)	--	10·1 (-4·4–40·7)
Sierra Leone	Measles	59·0 (32·3–69·6)	26·5 (13·2–34·0)	21·6 (8·8–37·9)	30·1 (14·2–38·2)
Togo	All causes	22·6 (11·4–33·3)	13·1 (5·3–19·6)	11·4 (3·5–20·3)	7·8 (5·0–10·5)
Togo	Diarrheal diseases	73·1 (49·3–89·1)	31·3 (19·1–43·0)	49·8 (-2·6–86·2)	20·9 (14·0–27·1)
Togo	Lower respiratory infections	60·1 (45·8–70·4)	40·3 (2·4–64·7)	19·0 (11·8–25·5)	31·1 (26·5–36·1)
Togo	Malaria	22·2 (-13·1–61·5)	17·2 (-9·8–45·0)	--	7·8 (-3·0–31·7)
Togo	Measles	56·5 (31·5–66·1)	27·0 (14·1–34·2)	19·1 (7·9–32·9)	27·2 (13·1–34·7)

Table S5. All-cause and cause-specific YLDs associated with child growth failure at the global, super-regional, regional, and national levels among children under 5 years, 2023 Estimates combine burden associated with mild, moderate, and severe forms of CGF: stunting was defined as height-for-age z-score (HAZ) < –1; underweight as weight-for-age z-score (WAZ) < –1; wasting as weight-for-height z-score (WHZ) < –1, according to WHO Child Growth Standards.

Location	Cause name	Child growth failure	Child underweight	Child wasting	Child stunting
**Global**	**All causes**	**1 540 000 (804 000–2 410 000)**	**1 470 000 (889 000–2 230 000)**	**1 220 000 (786 000–1 730 000)**	**59 000 (-76 600–209 000)**
**Global**	**Diarrheal diseases**	**189 000 (-137 000–521 000)**	**127 000 (-34 800–333 000)**	**43 300 (-29 200–134 000)**	**54 100 (-64 800–188 000)**
**Global**	**Lower respiratory infections**	**8 080 (-20 500–33 200)**	**4 920 (-8 100–20 000)**	**1 200 (-262–3 070)**	**3 530 (-10 100–18 600)**
**Global**	**Malaria**	**167 000 (-149 000–464 000)**	**167 000 (-149 000–464 000)**	**--**	**--**
**Global**	**Measles**	**4 420 (-2 210–15 600)**	**--**	**3 350 (-1 390–11 300)**	**1 280 (-701–5 720)**
**Central Europe, Eastern Europe, and Central Asia**	**All causes**	**9 410 (5 680–14 700)**	**8 640 (5 460–12 600)**	**8 210 (5 200–11 600)**	**549 (-641–2 010)**
**Central Europe, Eastern Europe, and Central Asia**	**Diarrheal diseases**	**1 470 (-931–4 310)**	**757 (-165–2 040)**	**338 (-210–1 030)**	**509 (-534–1 800)**
**Central Europe, Eastern Europe, and Central Asia**	**Lower respiratory infections**	**72·9 (-138–333)**	**30·0 (-37·6–132)**	**9·88 (-2·23–26·0)**	**38·2 (-96·8–209)**
**Central Europe, Eastern Europe, and Central Asia**	**Malaria**	**0 (0–0)**	**0 (0–0)**	**--**	**--**
**Central Europe, Eastern Europe, and Central Asia**	**Measles**	**4·47 (-1·78–13·1)**	**--**	**3·57 (-1·22–10·2)**	**0·973 (-0·464–3·71)**
Central Asia	All causes	4 440 (2 680–6 900)	4 090 (2 560–5 890)	3 830 (2 380–5 540)	264 (-325–961)
Central Asia	Diarrheal diseases	729 (-467–2 020)	409 (-93·7–1 090)	158 (-101–486)	240 (-259–831)
Central Asia	Lower respiratory infections	46·5 (-88–211)	20·8 (-26·3–92·3)	6·48 (-1·44–17·5)	23·0 (-59·2–127)
Central Asia	Malaria	0 (0–0)	0 (0–0)	--	--
Central Asia	Measles	3·39 (-1·34–10·1)	--	2·73 (-0·928–7·97)	0·716 (-0·333–2·59)
Armenia	All causes	35·2 (15·4–61·6)	30·3 (15·5–49·2)	28·3 (14·6–46·0)	3·61 (-4·49–13·0)
Armenia	Diarrheal diseases	8·21 (-5·94–24·7)	3·76 (-0·901–10·5)	1·86 (-1·29–6·13)	3·25 (-3·59–11·6)
Armenia	Lower respiratory infections	0·612 (-1·24–2·49)	0·217 (-0·29–0·826)	0·0795 (-0·0193–0·188)	0·355 (-0·903–1·67)
Armenia	Malaria	0 (0–0)	0 (0–0)	--	--
Armenia	Measles	0·0442 (-0·0173–0·136)	--	0·0349 (-0·0125–0·0999)	0·00998 (-0·0046–0·0359)
Azerbaijan	All causes	588 (321–981)	541 (300–844)	504 (290–756)	33·5 (-37·3–123)
Azerbaijan	Diarrheal diseases	103 (-61·1–302)	59·4 (-13·2–162)	22·4 (-13·9–70·7)	31·6 (-32·5–114)
Azerbaijan	Lower respiratory infections	3·45 (-6·62–14·7)	1·64 (-2·11–6·57)	0·45 (-0·103–1·14)	1·66 (-4·28–8·43)
Azerbaijan	Malaria	0 (0–0)	0 (0–0)	--	--
Azerbaijan	Measles	1·54 (-0·594–4·75)	--	1·25 (-0·427–3·75)	0·318 (-0·143–1·21)

Georgia	All causes	17·1 (-11·2–51·6)	7·72 (-1·19–20·8)	2·96 (-0·889–8·3)	7·93 (-9·07–28·3)
Georgia	Diarrheal diseases	15·9 (-10·6–49·3)	6·86 (-1·35–19·1)	2·24 (-1·26–6·9)	7·61 (-8·35–27·0)
Georgia	Lower respiratory infections	0·517 (-1·00–2·35)	0·176 (-0·219–0·693)	0·0387 (-0·00839–0·103)	0·323 (-0·774–1·64)
Georgia	Malaria	0 (0–0)	0 (0–0)	--	--
Georgia	Measles	0·00127 (>-0.001–0·00391)	--	<0.001 (>-0.001–0·00293)	<0.001 (>-0.001–0·00103)
Kazakhstan	All causes	410 (203–650)	367 (218–545)	344 (209–505)	32·3 (-36·1–116)
Kazakhstan	Diarrheal diseases	76·3 (-47·6–233)	37·2 (-7·54–104)	15·0 (-9·19–46·9)	29·4 (-30·3–105)
Kazakhstan	Lower respiratory infections	4·8 (-8·35–20·9)	1·89 (-2·31–7·72)	0·592 (-0·116–1·52)	2·59 (-6·05–13·2)
Kazakhstan	Malaria	0 (0–0)	0 (0–0)	--	--
Kazakhstan	Measles	1·22 (-0·483–3·73)	--	0·984 (-0·339–2·89)	0·249 (-0·12–0·908)
Kyrgyzstan	All causes	75·9 (3·28–165)	56·3 (25·0–103)	43·0 (20·7–74·1)	16·5 (-21·5–60·7)
Kyrgyzstan	Diarrheal diseases	35·9 (-24·1–109)	18·5 (-3·99–52·8)	5·79 (-3·54–18·6)	14·7 (-16·2–52·2)
Kyrgyzstan	Lower respiratory infections	2·8 (-6·04–12·5)	1·1 (-1·43–4·46)	0·221 (-0·0517–0·55)	1·67 (-4·42–8·47)
Kyrgyzstan	Malaria	0 (0–0)	0 (0–0)	--	--
Kyrgyzstan	Measles	0·423 (-0·177–1·3)	--	0·316 (-0·114–0·92)	0·113 (-0·0548–0·416)
Mongolia	All causes	8·51 (-3·23–21·4)	5·38 (1·22–11·9)	3·27 (1·21–6·97)	2·95 (-4·58–11·6)
Mongolia	Diarrheal diseases	4·68 (-3·57–14·9)	2·27 (-0·496–6·74)	0·502 (-0·319–1·6)	2·23 (-2·58–8·18)
Mongolia	Lower respiratory infections	1·12 (-2·51–5·29)	0·397 (-0·518–1·65)	0·0587 (-0·0127–0·155)	0·722 (-1·92–3·8)
Mongolia	Malaria	0 (0–0)	0 (0–0)	--	--
Mongolia	Measles	0 (0–0)	--	0 (0–0)	0 (0–0)
Tajikistan	All causes	2 150 (1 340–3 180)	2 030 (1 270–2 940)	1 910 (1 190–2 790)	88·5 (-105–326)
Tajikistan	Diarrheal diseases	301 (-195–822)	186 (-45·3–483)	72·3 (-48·3–218)	84·1 (-93–298)
Tajikistan	Lower respiratory infections	10·0 (-20·2–43·9)	5·34 (-7·37–23·5)	1·53 (-0·311–4·16)	4·4 (-12·1–24·2)
Tajikistan	Malaria	0 (0–0)	0 (0–0)	--	--
Tajikistan	Measles	0·0447 (-0·0186–0·13)	--	0·0367 (-0·0143–0·101)	0·00925 (-0·00484–0·0364)
Turkmenistan	All causes	295 (179–457)	281 (177–435)	271 (171–417)	9·17 (-11·3–34·2)
Turkmenistan	Diarrheal diseases	27·8 (-16–79·1)	15·7 (-3·4–42·6)	6·97 (-4·42–21·6)	7·74 (-7·78–27·7)
Turkmenistan	Lower respiratory infections	3·25 (-6·23–15·2)	1·58 (-2·24–7·18)	0·474 (-0·104–1·31)	1·42 (-3·74–7·94)
Turkmenistan	Malaria	0 (0–0)	0 (0–0)	--	--

Turkmenistan	Measles	0 (0–0)	--	0 (0–0)	0 (0–0)
Uzbekistan	All causes	859 (451–1 350)	772 (423–1 190)	718 (391–1 110)	69·4 (-96·7–249)
Uzbekistan	Diarrheal diseases	156 (-105–441)	80·1 (-17·5–211)	31·2 (-19·4–94·8)	59·5 (-68·2–201)
Uzbekistan	Lower respiratory infections	19·9 (-37·1–91·0)	8·48 (-10·8–38·3)	3·04 (-0·716–8·27)	9·9 (-24·5–54·8)
Uzbekistan	Malaria	0 (0–0)	0 (0–0)	--	--
Uzbekistan	Measles	0·118 (-0·0459–0·347)	--	0·104 (-0·0381–0·301)	0·0154 (-0·00723–0·0596)
Central Europe	All causes	702 (67·9–1 520)	495 (252–889)	411 (228–672)	140 (-159–514)
Central Europe	Diarrheal diseases	369 (-232–1 110)	172 (-36·1–481)	91·6 (-56·1–284)	131 (-137–470)
Central Europe	Lower respiratory infections	15·0 (-27·9–70·3)	5·35 (-6·75–23·1)	1·87 (-0·427–4·74)	8·57 (-20·9–46·3)
Central Europe	Malaria	0 (0–0)	0 (0–0)	--	--
Central Europe	Measles	0·119 (-0·0468–0·357)	--	0·0978 (-0·0345–0·269)	0·0228 (-0·00956–0·093)
Albania	All causes	49·1 (20·3–91·3)	42·6 (20·6–73·4)	40·4 (19·8–69·0)	4·59 (-5·24–16·5)
Albania	Diarrheal diseases	11·2 (-7·5–33·5)	5·08 (-1·02–14·4)	2·96 (-1·75–9·64)	4·31 (-4·63–15·0)
Albania	Lower respiratory infections	0·51 (-0·852–2·23)	0·172 (-0·186–0·732)	0·0973 (-0·0204–0·259)	0·283 (-0·656–1·49)
Albania	Malaria	0 (0–0)	0 (0–0)	--	--
Albania	Measles	<0.001 (>-0.001–<0.001)	--	<0.001 (>-0.001–<0.001)	<0.001 (>-0.001–<0.001)
Bosnia and Herzegovina	All causes	24·1 (7·95–48·2)	18·0 (10·0–30·1)	16·6 (9·36–27·0)	4·11 (-4·51–14·8)
Bosnia and Herzegovina	Diarrheal diseases	9·8 (-6·39–29·9)	3·94 (-0·798–10·8)	2·57 (-1·57–7·86)	3·9 (-3·96–13·8)
Bosnia and Herzegovina	Lower respiratory infections	0·328 (-0·586–1·46)	0·0925 (-0·113–0·395)	0·0442 (-0·01–0·123)	0·205 (-0·455–1·03)
Bosnia and Herzegovina	Malaria	0 (0–0)	0 (0–0)	--	--
Bosnia and Herzegovina	Measles	<0.001 (>-0.001–<0.001)	--	<0.001 (>-0.001–<0.001)	<0.001 (>-0.001–<0.001)
Bulgaria	All causes	70·7 (38·5–119)	63·1 (36·8–101)	60·5 (34·9–96·9)	4·16 (-4·47–15·0)
Bulgaria	Diarrheal diseases	13·9 (-7·95–42·3)	6·65 (-1·35–18·5)	4·19 (-2·63–13·0)	3·87 (-3·95–13·5)
Bulgaria	Lower respiratory infections	0·595 (-1·18–2·62)	0·235 (-0·35–0·984)	0·0865 (-0·0186–0·223)	0·295 (-0·786–1·55)
Bulgaria	Malaria	0 (0–0)	0 (0–0)	--	--
Bulgaria	Measles	0 (0–0)	--	0 (0–0)	0 (0–0)
Croatia	All causes	22·0 (-1·64–61·3)	13·5 (4·33–31·6)	10·7 (3·1–23·6)	5·22 (-5·46–19·4)
Croatia	Diarrheal diseases	15·3 (-8·86–46·6)	6·96 (-1·35–19·7)	4·29 (-2·51–13·4)	5·01 (-4·99–18·1)
Croatia	Lower respiratory infections	0·373 (-0·677–1·84)	0·134 (-0·166–0·615)	0·0486 (-0·0108–0·128)	0·208 (-0·506–1·18)

Croatia	Malaria	0 (0–0)	0 (0–0)	--	--
Croatia	Measles	0 (0–0)	--	0 (0–0)	0 (0–0)
Czechia	All causes	115 (29·0–235)	86·6 (43·1–153)	77·1 (38·7–128)	13·6 (-14·1–48·3)
Czechia	Diarrheal diseases	54·9 (-30–159)	27·0 (-5·63–73·7)	17·8 (-11·1–53·6)	13·3 (-13–46·6)
Czechia	Lower respiratory infections	0·833 (-1·31–3·6)	0·335 (-0·424–1·36)	0·159 (-0·0399–0·431)	0·369 (-0·858–1·91)
Czechia	Malaria	0 (0–0)	0 (0–0)	--	--
Czechia	Measles	<0.001 (>-0.001–<0.001)	--	<0.001 (>-0.001–<0.001)	<0.001 (>-0.001–<0.001)
Hungary	All causes	68·2 (-29·5–189)	34·9 (5·78–80·5)	25·8 (1·63–59·6)	21·9 (-22·7–80·5)
Hungary	Diarrheal diseases	56·2 (-35·3–174)	23·5 (-4·72–66·7)	14·6 (-8·82–45·1)	21·4 (-21·6–77·3)
Hungary	Lower respiratory infections	0·899 (-1·75–4·37)	0·27 (-0·338–1·21)	0·096 (-0·0208–0·259)	0·567 (-1·37–3·1)
Hungary	Malaria	0 (0–0)	0 (0–0)	--	--
Hungary	Measles	0 (0–0)	--	0 (0–0)	0 (0–0)
Montenegro	All causes	5·06 (1·39–10·0)	4·04 (2·12–6·6)	3·41 (1·85–5·49)	0·836 (-0·975–3·03)
Montenegro	Diarrheal diseases	1·87 (-1·22–5·64)	0·911 (-0·178–2·59)	0·312 (-0·186–1·02)	0·777 (-0·844–2·7)
Montenegro	Lower respiratory infections	0·0987 (-0·204–0·477)	0·0366 (-0·0482–0·172)	0·00791 (-0·00167–0·0212)	0·0597 (-0·149–0·331)
Montenegro	Malaria	0 (0–0)	0 (0–0)	--	--
Montenegro	Measles	0 (0–0)	--	0 (0–0)	0 (0–0)
North Macedonia	All causes	24·9 (8·17–48·0)	19·1 (10·8–31·7)	18·0 (10·1–29·8)	3·53 (-3·91–12·8)
North Macedonia	Diarrheal diseases	9·49 (-6·32–29·9)	3·87 (-0·818–11·0)	2·83 (-1·79–9·05)	3·4 (-3·6–12·1)
North Macedonia	Lower respiratory infections	0·204 (-0·379–0·93)	0·0642 (-0·0788–0·279)	0·023 (-0·00486–0·06)	0·125 (-0·289–0·657)
North Macedonia	Malaria	0 (0–0)	0 (0–0)	--	--
North Macedonia	Measles	0·00132 (>-0.001–0·00425)	--	0·00113 (>-0.001–0·00327)	<0.001 (>-0.001–<0.001)
Poland	All causes	87·6 (-1·4–206)	60·4 (21·7–117)	51·7 (20·5–105)	17·9 (-23·5–69·9)
Poland	Diarrheal diseases	41·5 (-24·9–130)	18·5 (-3·74–54·5)	11·3 (-6·92–37·3)	14·2 (-13·9–53·1)
Poland	Lower respiratory infections	6·5 (-12·3–28·5)	2·28 (-2·92–9·58)	0·851 (-0·193–2·2)	3·66 (-9·13–18·8)
Poland	Malaria	0 (0–0)	0 (0–0)	--	--
Poland	Measles	0·0011 (>-0.001–0·00378)	--	<0.001 (>-0.001–0·00309)	<0.001 (>-0.001–<0.001)
Romania	All causes	121 (-54·8–334)	67·8 (6·44–160)	35·9 (6·53–79·0)	43·8 (-50·9–157)
Romania	Diarrheal diseases	99·2 (-70·1–293)	48·3 (-10·7–135)	17·0 (-10·7–54·2)	42·0 (-45·8–147)

Romania	Lower respiratory infections	2·85 (-6·58–13·3)	1·02 (-1·39–4·26)	0·243 (-0·056–0·658)	1·77 (-5·08–9·22)
Romania	Malaria	0 (0–0)	0 (0–0)	--	--
Romania	Measles	0·111 (-0·0433–0·335)	--	0·091 (-0·0322–0·249)	0·0213 (-0·00883–0·0867)
Serbia	All causes	65·7 (19·9–130)	52·2 (26·6–87·1)	44·0 (23·0–71·4)	9·6 (-10·7–35·4)
Serbia	Diarrheal diseases	27·1 (-16·5–82·8)	14·0 (-3·01–39·6)	6·03 (-3·77–19·1)	9·21 (-9·85–33·7)
Serbia	Lower respiratory infections	0·709 (-1·33–3·17)	0·295 (-0·373–1·26)	0·0688 (-0·0144–0·181)	0·388 (-0·913–2·04)
Serbia	Malaria	0 (0–0)	0 (0–0)	--	--
Serbia	Measles	0·00347 (-0·00156–0·0103)	--	0·00285 (-0·00121–0·00821)	<0.001 (>-0.001–0·00279)
Slovakia	All causes	30·7 (0·916–73·6)	21·1 (8–39·7)	17·5 (6·69–31·9)	6·14 (-7·16–22·4)
Slovakia	Diarrheal diseases	17·1 (-11–53·7)	8·01 (-1·73–23·0)	4·56 (-2·93–14·6)	5·72 (-6·12–20·1)
Slovakia	Lower respiratory infections	0·755 (-1·43–3·42)	0·278 (-0·363–1·17)	0·0949 (-0·0201–0·26)	0·421 (-1·01–2·19)
Slovakia	Malaria	0 (0–0)	0 (0–0)	--	--
Slovakia	Measles	0 (0–0)	--	0 (0–0)	0 (0–0)
Slovenia	All causes	7·92 (-3·06–21·8)	4·29 (0·386–10·2)	3·39 (-0·0353–8·03)	2·24 (-2·35–8·11)
Slovenia	Diarrheal diseases	6·1 (-3·51–18·7)	2·58 (-0·471–7·17)	1·71 (-0·97–5·26)	2·15 (-2·16–7·66)
Slovenia	Lower respiratory infections	0·172 (-0·298–0·812)	0·0578 (-0·0698–0·261)	0·0233 (-0·00519–0·0602)	0·0978 (-0·221–0·537)
Slovenia	Malaria	0 (0–0)	0 (0–0)	--	--
Slovenia	Measles	0 (0–0)	--	0 (0–0)	0 (0–0)
Eastern Europe	All causes	4 270 (2 610–6 260)	4 060 (2 530–5 810)	3 970 (2 480–5 680)	145 (-159–521)
Eastern Europe	Diarrheal diseases	377 (-233–1 150)	175 (-35·7–503)	88·6 (-53·4–288)	138 (-140–493)
Eastern Europe	Lower respiratory infections	11·3 (-22·5–50·6)	3·81 (-5·08–15·9)	1·53 (-0·362–4·04)	6·62 (-16·6–34·1)
Eastern Europe	Malaria	0 (0–0)	0 (0–0)	--	--
Eastern Europe	Measles	0·96 (-0·386–2·93)	--	0·741 (-0·265–2·1)	0·234 (-0·108–0·971)
Belarus	All causes	68·5 (-24·6–199)	44·2 (-1·99–116)	19·0 (-0·165–46·7)	15·4 (-15·7–56·6)
Belarus	Diarrheal diseases	60·8 (-29·3–190)	36·8 (-7·07–108)	11·9 (-7·04–38·7)	15·2 (-15·2–55·7)
Belarus	Lower respiratory infections	0·582 (-1·00–2·51)	0·324 (-0·414–1·38)	0·0519 (-0·0155–0·135)	0·227 (-0·551–1·17)
Belarus	Malaria	0 (0–0)	0 (0–0)	--	--
Belarus	Measles	0·004 (-0·00148–0·0123)	--	0·00361 (-0·00127–0·0109)	<0.001 (>-0.001–0·00152)
Estonia	All causes	12·0 (-6·79–37·6)	5·75 (-1·05–16·2)	3·24 (-1·66–9·92)	3·96 (-3·92–14·5)

Estonia	Diarrheal diseases	11·6 (-6·68–37·1)	5·42 (-1·08–15·8)	2·94 (-1·74–9·56)	3·91 (-3·81–14·2)
Estonia	Lower respiratory infections	0·0861 (-0·165–0·398)	0·0332 (-0·0439–0·144)	0·0105 (-0·00265–0·0271)	0·046 (-0·117–0·245)
Estonia	Malaria	0 (0–0)	0 (0–0)	--	--
Estonia	Measles	<0.001 (>-0.001–<0.001)	--	<0.001 (>-0.001–<0.001)	<0.001 (>-0.001–<0.001)
Latvia	All causes	11·7 (-5·72–35·0)	6·12 (-0·375–16·2)	3·34 (-0·745–9·13)	3·67 (-3·8–12·9)
Latvia	Diarrheal diseases	10·8 (-5·99–33·6)	5·28 (-1·02–15·1)	2·54 (-1·48–8·06)	3·61 (-3·65–12·7)
Latvia	Lower respiratory infections	0·116 (-0·208–0·525)	0·0462 (-0·0578–0·2)	0·0131 (-0·00339–0·0373)	0·0624 (-0·142–0·325)
Latvia	Malaria	0 (0–0)	0 (0–0)	--	--
Latvia	Measles	0 (0–0)	--	0 (0–0)	0 (0–0)
Lithuania	All causes	18·0 (-10·3–52·8)	8·85 (-1·42–23·3)	4·85 (-2·32–14·2)	5·83 (-6·07–20·3)
Lithuania	Diarrheal diseases	17·3 (-10·2–51·7)	8·22 (-1·71–22·5)	4·26 (-2·61–13·1)	5·74 (-5·81–20·0)
Lithuania	Lower respiratory infections	0·158 (-0·338–0·766)	0·0609 (-0·0903–0·272)	0·0187 (-0·00516–0·0518)	0·0853 (-0·232–0·479)
Lithuania	Malaria	0 (0–0)	0 (0–0)	--	--
Lithuania	Measles	<0.001 (>-0.001–<0.001)	--	<0.001 (>-0.001–<0.001)	<0.001 (>-0.001–<0.001)
Republic of Moldova	All causes	25·1 (-10·9–69·3)	14·4 (0·506–35·0)	8·19 (0·0727–19·7)	6·79 (-7·3–23·7)
Republic of Moldova	Diarrheal diseases	21·9 (-13·1–65·4)	11·5 (-2·51–31·4)	5·32 (-3·37–16·4)	6·64 (-7·04–23·0)
Republic of Moldova	Lower respiratory infections	0·302 (-0·556–1·5)	0·131 (-0·166–0·613)	0·0299 (-0·00507–0·0873)	0·156 (-0·37–0·907)
Republic of Moldova	Malaria	0 (0–0)	0 (0–0)	--	--
Republic of Moldova	Measles	<0.001 (>-0.001–<0.001)	--	<0.001 (>-0.001–<0.001)	<0.001 (>-0.001–<0.001)
Russian Federation	All causes	1 160 (594–1 840)	1 030 (559–1 570)	994 (536–1 520)	88·8 (-99·4–326)
Russian Federation	Diarrheal diseases	212 (-140–641)	90·4 (-18·6–257)	53·0 (-32·4–171)	83·8 (-86·3–297)
Russian Federation	Lower respiratory infections	8·19 (-16–35·8)	2·66 (-3·48–11·2)	1·24 (-0·29–3·23)	4·74 (-12–24·2)
Russian Federation	Malaria	0 (0–0)	0 (0–0)	--	--
Russian Federation	Measles	0·951 (-0·383–2·91)	--	0·734 (-0·262–2·08)	0·232 (-0·107–0·962)
Ukraine	All causes	2 970 (1 840–4 280)	2 950 (1 830–4 250)	2 940 (1 830–4 240)	20·5 (-24·6–75·6)
Ukraine	Diarrheal diseases	42·4 (-30·5–127)	17·7 (-3·73–48·6)	8·59 (-5·37–27·2)	19·2 (-21–66·5)
Ukraine	Lower respiratory infections	1·92 (-4·34–9·52)	0·547 (-0·745–2·35)	0·169 (-0·0399–0·476)	1·3 (-3·53–7·29)
Ukraine	Malaria	0 (0–0)	0 (0–0)	--	--
Ukraine	Measles	0·00403 (-0·00188–0·0139)	--	0·00242 (>-0.001–0·0073)	0·00171 (>-0.001–0·00713)

**High-income**	**All causes**	**2 400 (-704–6 760)**	**1 400 (189–3 360)**	**864 (108–1 970)**	**609 (-624–2 250)**
**High-income**	**Diarrheal diseases**	**1 990 (-1 090–6 270)**	**1 000 (-205–2 930)**	**475 (-285–1 570)**	**598 (-591–2 200)**
**High-income**	**Lower respiratory infections**	**23·3 (-39·1–103)**	**9·2 (-11·6–38·7)**	**3·18 (-0·759–8·25)**	**11·7 (-26·1–61·5)**
**High-income**	**Malaria**	**0·0753 (-0·0302–0·312)**	**0·0753 (-0·0302–0·312)**	**--**	**--**
**High-income**	**Measles**	**0·0329 (-0·0118–0·0965)**	**--**	**0·0313 (-0·0111–0·0894)**	**0·00157 (>-0.001–0·00588)**
Australasia	All causes	72·4 (-45·3–246)	20·8 (-3·35–63·0)	27·4 (-15·6–90·5)	26·8 (-25·9–101)
Australasia	Diarrheal diseases	71·3 (-45·3–243)	20·0 (-3·88–61·6)	26·6 (-16·1–89·3)	26·6 (-25·4–101)
Australasia	Lower respiratory infections	0·305 (-0·503–1·39)	0·0742 (-0·0874–0·315)	0·0523 (-0·0126–0·136)	0·184 (-0·414–0·977)
Australasia	Malaria	0 (0–0)	0 (0–0)	--	--
Australasia	Measles	0·00153 (>-0.001–0·0046)	--	0·00146 (>-0.001–0·00434)	<0.001 (>-0.001–<0.001)
Australia	All causes	60·9 (-38·8–204)	16·6 (-2·77–50·0)	23·7 (-13·8–77·9)	22·7 (-22–85·5)
Australia	Diarrheal diseases	60·2 (-38·8–202)	16·1 (-3·14–49·4)	23·1 (-14·2–77·4)	22·6 (-21·7–84·8)
Australia	Lower respiratory infections	0·206 (-0·347–0·958)	0·0448 (-0·0542–0·201)	0·0382 (-0·009–0·102)	0·126 (-0·291–0·678)
Australia	Malaria	0 (0–0)	0 (0–0)	--	--
Australia	Measles	<0.001 (>-0.001–0·00284)	--	<0.001 (>-0.001–0·00271)	<0.001 (>-0.001–<0.001)
New Zealand	All causes	11·4 (-6·46–36·6)	4·2 (-0·583–12·1)	3·69 (-1·81–11·7)	4·12 (-4·06–15·1)
New Zealand	Diarrheal diseases	11·1 (-6·49–35·9)	3·95 (-0·739–11·8)	3·46 (-1·98–11·4)	4·07 (-3·94–14·8)
New Zealand	Lower respiratory infections	0·0985 (-0·16–0·446)	0·0295 (-0·0342–0·123)	0·0141 (-0·00279–0·0371)	0·0571 (-0·123–0·297)
New Zealand	Malaria	0 (0–0)	0 (0–0)	--	--
New Zealand	Measles	<0.001 (>-0.001–0·00183)	--	<0.001 (>-0.001–0·0017)	<0.001 (>-0.001–<0.001)
High-income Asia Pacific	All causes	731 (85·4–1 690)	537 (226–1 050)	396 (202–697)	112 (-110–428)
High-income Asia Pacific	Diarrheal diseases	436 (-218–1 350)	244 (-49·1–704)	105 (-61·2–347)	111 (-105–419)
High-income Asia Pacific	Lower respiratory infections	4·35 (-7·04–19·4)	2·13 (-2·74–9·15)	0·594 (-0·14–1·56)	1·8 (-4·05–9·51)
High-income Asia Pacific	Malaria	0·0753 (-0·0302–0·312)	0·0753 (-0·0302–0·312)	--	--
High-income Asia Pacific	Measles	0·00209 (>-0.001–0·00664)	--	0·00197 (>-0.001–0·00626)	<0.001 (>-0.001–<0.001)
Brunei Darussalam	All causes	2·77 (1·04–5·42)	2·5 (0·926–4·71)	2·33 (0·779–4·4)	0·22 (-0·29–0·817)
Brunei Darussalam	Diarrheal diseases	0·488 (-0·308–1·47)	0·252 (-0·0481–0·7)	0·0903 (-0·0548–0·287)	0·187 (-0·201–0·649)
Brunei Darussalam	Lower respiratory infections	0·0554 (-0·127–0·258)	0·0213 (-0·0307–0·0902)	0·00509 (-0·00114–0·0129)	0·0329 (-0·0907–0·173)
Brunei Darussalam	Malaria	0 (0–0)	0 (0–0)	--	--

Brunei Darussalam	Measles	0 (0–0)	--	0 (0–0)	0 (0–0)
Japan	All causes	699 (93·2–1 600)	518 (225–1 000)	384 (200–666)	106 (-104–402)
Japan	Diarrheal diseases	411 (-206–1 270)	231 (-46·7–668)	97·6 (-57–324)	105 (-100–396)
Japan	Lower respiratory infections	3·14 (-5·07–13·6)	1·57 (-2·02–6·65)	0·401 (-0·0966–1·04)	1·29 (-2·88–6·77)
Japan	Malaria	0 (0–0)	0 (0–0)	--	--
Japan	Measles	0·00147 (>-0.001–0·0042)	--	0·00137 (>-0.001–0·00388)	<0.001 (>-0.001–<0.001)
Republic of Korea	All causes	25·0 (-11·9–77·2)	13·1 (-1·93–37·1)	7·94 (-3·41–23·6)	5·75 (-6·05–21·5)
Republic of Korea	Diarrheal diseases	23·2 (-11·5–72·2)	11·8 (-2·2–34·2)	6·96 (-4·25–22·2)	5·43 (-5·36–19·6)
Republic of Korea	Lower respiratory infections	0·811 (-1·27–3·77)	0·375 (-0·492–1·66)	0·136 (-0·0308–0·395)	0·324 (-0·723–1·87)
Republic of Korea	Malaria	0·0753 (-0·0302–0·312)	0·0753 (-0·0302–0·312)	--	--
Republic of Korea	Measles	<0.001 (>-0.001–<0.001)	--	<0.001 (>-0.001–<0.001)	<0.001 (>-0.001–<0.001)
Singapore	All causes	3·73 (-0·575–10·6)	2·87 (0·166–9·42)	2·37 (0·209–8·37)	0·532 (-0·719–2·08)
Singapore	Diarrheal diseases	1·45 (-0·77–4·15)	0·769 (-0·156–2·02)	0·377 (-0·227–1·12)	0·384 (-0·374–1·3)
Singapore	Lower respiratory infections	0·345 (-0·576–1·59)	0·16 (-0·204–0·715)	0·0515 (-0·0116–0·14)	0·148 (-0·361–0·837)
Singapore	Malaria	0 (0–0)	0 (0–0)	--	--
Singapore	Measles	<0.001 (>-0.001–0·00156)	--	<0.001 (>-0.001–0·00146)	<0.001 (>-0.001–<0.001)
High-income North America	All causes	137 (-71·9–433)	64·5 (-5·34–179)	47·2 (-13·6–133)	41·1 (-44·2–161)
High-income North America	Diarrheal diseases	119 (-66·1–388)	52·0 (-10·1–156)	35·6 (-21·3–118)	37·1 (-34·7–142)
High-income North America	Lower respiratory infections	7·85 (-13–35·0)	2·54 (-3·17–10·8)	1·56 (-0·348–4·16)	4 (-9·41–21·7)
High-income North America	Malaria	0 (0–0)	0 (0–0)	--	--
High-income North America	Measles	0·00883 (-0·00321–0·0315)	--	0·00841 (-0·00297–0·0292)	<0.001 (>-0.001–0·00207)
Canada	All causes	15·6 (-7·34–45·9)	7·51 (-1·19–20·5)	6·83 (-3·6–20·6)	2·09 (-2·16–7·48)
Canada	Diarrheal diseases	14·9 (-7·39–44·2)	6·93 (-1·41–19·2)	6·31 (-3·97–19·6)	2·03 (-2·01–7·15)
Canada	Lower respiratory infections	0·243 (-0·309–1·03)	0·12 (-0·149–0·532)	0·0637 (-0·0153–0·169)	0·0631 (-0·137–0·344)
Canada	Malaria	0 (0–0)	0 (0–0)	--	--
Canada	Measles	<0.001 (>-0.001–<0.001)	--	<0.001 (>-0.001–<0.001)	<0.001 (>-0.001–<0.001)
Greenland	All causes	0·0275 (-0·0129–0·0933)	0·0108 (-0·00107–0·0317)	0·0125 (-0·00382–0·0415)	0·00663 (-0·00714–0·0268)
Greenland	Diarrheal diseases	0·0184 (-0·0099–0·0646)	0·00873 (-0·0015–0·027)	0·005 (-0·00287–0·0188)	0·00558 (-0·00564–0·022)
Greenland	Lower respiratory infections	0·00145 (-0·00251–0·00696)	<0.001 (>-0.001–0·00264)	<0.001 (>-0.001–<0.001)	<0.001 (-0·00169–0·00405)

Greenland	Malaria	0 (0–0)	0 (0–0)	--	--
Greenland	Measles	0·0062 (-0·00234–0·024)	--	0·00587 (-0·00218–0·0221)	<0.001 (>-0.001–0·00175)
United States of America	All causes	122 (-64·5–389)	57·0 (-4·14–157)	40·4 (-9·32–112)	39·0 (-42–154)
United States of America	Diarrheal diseases	105 (-58·7–344)	45·0 (-8·67–136)	29·3 (-17·4–96·8)	35·0 (-32·7–135)
United States of America	Lower respiratory infections	7·61 (-12·7–34·1)	2·42 (-3·03–10·3)	1·49 (-0·333–4·01)	3·93 (-9·29–21·4)
United States of America	Malaria	0 (0–0)	0 (0–0)	--	--
United States of America	Measles	0·00252 (>-0.001–0·00751)	--	0·00243 (>-0.001–0·00718)	<0.001 (>-0.001–<0.001)
Southern Latin America	All causes	292 (-186–869)	154 (-33·6–423)	53·1 (-28·8–157)	104 (-120–368)
Southern Latin America	Diarrheal diseases	284 (-175–840)	149 (-32·6–409)	49·9 (-31·4–151)	101 (-112–353)
Southern Latin America	Lower respiratory infections	5·59 (-10·7–25·3)	2·24 (-2·83–9·26)	0·42 (-0·109–1·11)	3·18 (-7·55–16·2)
Southern Latin America	Malaria	0 (0–0)	0 (0–0)	--	--
Southern Latin America	Measles	<0.001 (>-0.001–<0.001)	--	<0.001 (>-0.001–<0.001)	<0.001 (>-0.001–<0.001)
Argentina	All causes	204 (-141–596)	101 (-22–272)	37·1 (-19·7–108)	80·9 (-95·6–280)
Argentina	Diarrheal diseases	197 (-131–574)	97·0 (-21·5–263)	34·5 (-22–105)	78·2 (-88·7–267)
Argentina	Lower respiratory infections	4·46 (-8·85–20·1)	1·65 (-2·12–6·7)	0·33 (-0·087–0·885)	2·68 (-6·46–13·5)
Argentina	Malaria	0 (0–0)	0 (0–0)	--	--
Argentina	Measles	0 (0–0)	--	0 (0–0)	0 (0–0)
Chile	All causes	69·6 (-35·7–217)	42·7 (-9·41–125)	13·1 (-7·88–43·5)	17·0 (-17·5–65·1)
Chile	Diarrheal diseases	68·6 (-34·6–213)	42·0 (-8·97–122)	12·8 (-8·05–43·0)	16·7 (-16·6–63·5)
Chile	Lower respiratory infections	0·826 (-1·38–3·9)	0·45 (-0·528–2·04)	0·0695 (-0·0158–0·204)	0·334 (-0·831–1·85)
Chile	Malaria	0 (0–0)	0 (0–0)	--	--
Chile	Measles	<0.001 (>-0.001–<0.001)	--	<0.001 (>-0.001–<0.001)	<0.001 (>-0.001–<0.001)
Uruguay	All causes	18·0 (-11–55·5)	10·2 (-2·25–29·1)	2·83 (-1·49–8·73)	6·34 (-7·1–23·7)
Uruguay	Diarrheal diseases	17·5 (-10·4–54·0)	9·88 (-2·17–28·4)	2·63 (-1·61–8·46)	6·18 (-6·61–22·9)
Uruguay	Lower respiratory infections	0·301 (-0·66–1·46)	0·138 (-0·198–0·632)	0·0199 (-0·00479–0·0547)	0·161 (-0·449–0·891)
Uruguay	Malaria	0 (0–0)	0 (0–0)	--	--
Uruguay	Measles	0 (0–0)	--	0 (0–0)	0 (0–0)
Western Europe	All causes	1 170 (-526–3 580)	621 (-43·3–1 700)	340 (-88·1–953)	325 (-322–1 200)
Western Europe	Diarrheal diseases	1 080 (-583–3 480)	538 (-110–1 600)	258 (-156–861)	322 (-315–1 190)

Western Europe	Lower respiratory infections	5·19 (-8·54–23·7)	2·21 (-2·8–9·35)	0·56 (-0·143–1·47)	2·53 (-5·52–13·7)
Western Europe	Malaria	0 (0–0)	0 (0–0)	--	--
Western Europe	Measles	0·0204 (-0·00736–0·0569)	--	0·0195 (-0·00695–0·054)	<0.001 (>-0.001–0·00371)
Andorra	All causes	0·11 (-0·0575–0·355)	0·0516 (-0·00906–0·151)	0·0286 (-0·0153–0·0907)	0·0338 (-0·032–0·129)
Andorra	Diarrheal diseases	0·108 (-0·0575–0·352)	0·0501 (-0·00976–0·149)	0·0271 (-0·0162–0·0898)	0·0335 (-0·0315–0·127)
Andorra	Lower respiratory infections	<0.001 (>-0.001–0·00264)	<0.001 (>-0.001–0·00101)	<0.001 (>-0.001–<0.001)	<0.001 (>-0.001–0·00156)
Andorra	Malaria	0 (0–0)	0 (0–0)	--	--
Andorra	Measles	0 (0–0)	--	0 (0–0)	0 (0–0)
Austria	All causes	29·8 (-17·7–95·5)	14·4 (-2·84–42·3)	7·53 (-4·42–24·1)	9·31 (-9·9–34·5)
Austria	Diarrheal diseases	29·1 (-17·7–93·8)	13·8 (-2·94–41·2)	6·96 (-4·77–23·1)	9·25 (-9·78–34·1)
Austria	Lower respiratory infections	0·118 (-0·195–0·541)	0·047 (-0·0559–0·201)	0·0125 (-0·00275–0·0341)	0·0607 (-0·138–0·316)
Austria	Malaria	0 (0–0)	0 (0–0)	--	--
Austria	Measles	0·00487 (-0·00181–0·0148)	--	0·00463 (-0·0017–0·014)	<0.001 (>-0.001–0·001)
Belgium	All causes	31·2 (-15·7–94·6)	15·1 (-2·54–42·5)	8·11 (-4·18–25·5)	9·5 (-9·29–34·5)
Belgium	Diarrheal diseases	30·4 (-16·1–93·5)	14·5 (-2·71–40·8)	7·51 (-4·43–23·7)	9·43 (-9·16–34·2)
Belgium	Lower respiratory infections	0·146 (-0·24–0·698)	0·0588 (-0·0716–0·267)	0·016 (-0·00332–0·0439)	0·0741 (-0·163–0·405)
Belgium	Malaria	0 (0–0)	0 (0–0)	--	--
Belgium	Measles	0·00223 (>-0.001–0·0067)	--	0·00212 (>-0.001–0·00624)	<0.001 (>-0.001–<0.001)
Cyprus	All causes	7·12 (-2·6–21·2)	3·97 (0·259–10·2)	2·13 (-0·179–5·48)	1·95 (-1·91–7·36)
Cyprus	Diarrheal diseases	6·41 (-3·35–20·7)	3·27 (-0·635–9·76)	1·44 (-0·828–4·73)	1·94 (-1·89–7·32)
Cyprus	Lower respiratory infections	0·0176 (-0·03–0·0834)	0·00759 (-0·00958–0·0339)	0·00177 (>-0.001–0·00503)	0·0087 (-0·0201–0·0474)
Cyprus	Malaria	0 (0–0)	0 (0–0)	--	--
Cyprus	Measles	0 (0–0)	--	0 (0–0)	0 (0–0)
Denmark	All causes	21·3 (-10–66·5)	10·6 (-1·31–30·5)	5·8 (-2·26–17·7)	6·25 (-6·22–22·5)
Denmark	Diarrheal diseases	20·5 (-10·5–64·2)	9·87 (-1·81–28·4)	5·06 (-2·87–16·3)	6·21 (-6·14–22·4)
Denmark	Lower respiratory infections	0·0813 (-0·127–0·37)	0·0333 (-0·0396–0·145)	0·0092 (-0·00217–0·0255)	0·0406 (-0·085–0·209)
Denmark	Malaria	0 (0–0)	0 (0–0)	--	--
Denmark	Measles	0 (0–0)	--	0 (0–0)	0 (0–0)
Finland	All causes	12·2 (-5·44–37·1)	6·44 (-0·552–17·7)	3·6 (-1·18–10·2)	3·48 (-3·42–13·4)

Finland	Diarrheal diseases	11·2 (-6·2–36·3)	5·5 (-1·12–16·3)	2·67 (-1·62–8·85)	3·45 (-3·37–13·2)
Finland	Lower respiratory infections	0·0664 (-0·091–0·342)	0·0275 (-0·0282–0·13)	0·00705 (-0·00116–0·0194)	0·0333 (-0·0633–0·199)
Finland	Malaria	0 (0–0)	0 (0–0)	--	--
Finland	Measles	0 (0–0)	--	0 (0–0)	0 (0–0)
France	All causes	246 (-141–771)	118 (-22·9–339)	64·5 (-36·2–200)	75·7 (-78·9–278)
France	Diarrheal diseases	241 (-140–765)	113 (-24·2–332)	59·8 (-38·1–197)	75·2 (-77·4–276)
France	Lower respiratory infections	0·868 (-1·41–4·15)	0·347 (-0·421–1·53)	0·0977 (-0·0222–0·273)	0·441 (-0·983–2·47)
France	Malaria	0 (0–0)	0 (0–0)	--	--
France	Measles	0·00306 (-0·00104–0·0087)	--	0·00291 (>-0.001–0·00816)	<0.001 (>-0.001–<0.001)
Germany	All causes	268 (-123–844)	152 (-25·3–451)	68·4 (-32·4–218)	62·9 (-61·7–228)
Germany	Diarrheal diseases	259 (-131–827)	144 (-30·3–435)	60·5 (-37·8–202)	62·5 (-60·8–226)
Germany	Lower respiratory infections	1·1 (-1·74–4·6)	0·548 (-0·702–2·29)	0·123 (-0·0331–0·336)	0·452 (-0·981–2·26)
Germany	Malaria	0 (0–0)	0 (0–0)	--	--
Germany	Measles	0·00232 (>-0.001–0·00725)	--	0·00223 (>-0.001–0·00685)	<0.001 (>-0.001–<0.001)
Greece	All causes	21·9 (-12·6–69·4)	10·4 (-2·07–29·9)	4·99 (-2·85–16·0)	7·39 (-7·26–26·6)
Greece	Diarrheal diseases	21·6 (-12·6–68·7)	10·2 (-2·16–29·5)	4·82 (-3·02–15·7)	7·33 (-7·12–26·3)
Greece	Lower respiratory infections	0·104 (-0·182–0·505)	0·0408 (-0·0493–0·188)	0·0103 (-0·00221–0·0286)	0·0557 (-0·132–0·298)
Greece	Malaria	0 (0–0)	0 (0–0)	--	--
Greece	Measles	0 (0–0)	--	0 (0–0)	0 (0–0)
Iceland	All causes	1·29 (-0·675–3·89)	0·623 (-0·0874–1·67)	0·352 (-0·162–1·01)	0·389 (-0·391–1·42)
Iceland	Diarrheal diseases	1·24 (-0·703–3·85)	0·582 (-0·12–1·64)	0·313 (-0·193–0·988)	0·386 (-0·386–1·41)
Iceland	Lower respiratory infections	0·00555 (-0·00864–0·0266)	0·00223 (-0·00253–0·0107)	<0.001 (>-0.001–0·00181)	0·0028 (-0·00598–0·0148)
Iceland	Malaria	0 (0–0)	0 (0–0)	--	--
Iceland	Measles	0 (0–0)	--	0 (0–0)	0 (0–0)
Ireland	All causes	15·8 (-8·58–51·4)	7·25 (-0·973–21·8)	4·29 (-2·02–14·0)	4·89 (-4·87–18·2)
Ireland	Diarrheal diseases	15·5 (-9·04–50·7)	6·99 (-1·48–21·3)	4·04 (-2·56–13·7)	4·86 (-4·8–18·0)
Ireland	Lower respiratory infections	0·0637 (-0·105–0·329)	0·0247 (-0·031–0·12)	0·00756 (-0·00211–0·0229)	0·0326 (-0·0702–0·194)
Ireland	Malaria	0 (0–0)	0 (0–0)	--	--
Ireland	Measles	0 (0–0)	--	0 (0–0)	0 (0–0)

Israel	All causes	70·5 (-37–226)	33·2 (-5·9–97·2)	17·7 (-9·44–57·1)	22·3 (-22–84·0)
Israel	Diarrheal diseases	69·6 (-37·2–224)	32·4 (-6·33–96·1)	17·0 (-9·96–56·2)	22·2 (-21·7–83·5)
Israel	Lower respiratory infections	0·213 (-0·353–1)	0·0839 (-0·106–0·372)	0·0232 (-0·00502–0·0693)	0·11 (-0·239–0·583)
Israel	Malaria	0 (0–0)	0 (0–0)	--	--
Israel	Measles	<0.001 (>-0.001–<0.001)	--	<0.001 (>-0.001–<0.001)	<0.001 (>-0.001–<0.001)
Italy	All causes	67·5 (-32·2–215)	34·1 (-2·48–96·5)	20·2 (-5·48–59·2)	20·0 (-19·6–75·2)
Italy	Diarrheal diseases	62·1 (-34–203)	29·0 (-5·78–86·0)	15·2 (-9·22–49·8)	19·7 (-18·9–74·0)
Italy	Lower respiratory infections	0·484 (-0·858–2·19)	0·193 (-0·243–0·807)	0·0539 (-0·0142–0·138)	0·247 (-0·583–1·31)
Italy	Malaria	0 (0–0)	0 (0–0)	--	--
Italy	Measles	<0.001 (>-0.001–0·00239)	--	<0.001 (>-0.001–0·00226)	<0.001 (>-0.001–<0.001)
Luxembourg	All causes	1·97 (-0·922–6·05)	1·03 (-0·0864–2·83)	0·583 (-0·158–1·7)	0·551 (-0·535–2·04)
Luxembourg	Diarrheal diseases	1·83 (-0·949–5·93)	0·895 (-0·165–2·69)	0·45 (-0·266–1·52)	0·547 (-0·527–2·01)
Luxembourg	Lower respiratory infections	0·00866 (-0·0149–0·0421)	0·00363 (-0·00475–0·0166)	<0.001 (>-0.001–0·00284)	0·00425 (-0·00987–0·0238)
Luxembourg	Malaria	0 (0–0)	0 (0–0)	--	--
Luxembourg	Measles	0 (0–0)	--	0 (0–0)	0 (0–0)
Malta	All causes	1·54 (-0·821–4·8)	0·797 (-0·125–2·2)	0·414 (-0·188–1·28)	0·451 (-0·452–1·71)
Malta	Diarrheal diseases	1·45 (-0·845–4·64)	0·716 (-0·158–2·09)	0·334 (-0·218–1·08)	0·448 (-0·446–1·7)
Malta	Lower respiratory infections	0·00571 (-0·00956–0·028)	0·00239 (-0·0029–0·0111)	<0.001 (>-0.001–0·00162)	0·00285 (-0·00673–0·0161)
Malta	Malaria	0 (0–0)	0 (0–0)	--	--
Malta	Measles	0 (0–0)	--	0 (0–0)	0 (0–0)
Monaco	All causes	0·0731 (-0·0399–0·231)	0·0323 (-0·00591–0·0924)	0·0195 (-0·0104–0·0608)	0·0237 (-0·022–0·0888)
Monaco	Diarrheal diseases	0·0721 (-0·0397–0·229)	0·0316 (-0·00617–0·0913)	0·0189 (-0·011–0·0604)	0·0235 (-0·0216–0·0881)
Monaco	Lower respiratory infections	<0.001 (>-0.001–0·00144)	<0.001 (>-0.001–<0.001)	<0.001 (>-0.001–<0.001)	<0.001 (>-0.001–<0.001)
Monaco	Malaria	0 (0–0)	0 (0–0)	--	--
Monaco	Measles	0 (0–0)	--	0 (0–0)	0 (0–0)
Netherlands	All causes	52·2 (20·0–97·0)	45·9 (21·0–79·2)	39·8 (19·1–68·7)	3·65 (-3·55–13·1)
Netherlands	Diarrheal diseases	15·4 (-6·97–45·4)	9·15 (-1·74–25·0)	3·17 (-1·87–9·85)	3·57 (-3·42–12·7)
Netherlands	Lower respiratory infections	0·186 (-0·312–0·909)	0·0957 (-0·117–0·42)	0·0174 (-0·00397–0·0508)	0·0771 (-0·18–0·438)
Netherlands	Malaria	0 (0–0)	0 (0–0)	--	--

Netherlands	Measles	<0.001 (>-0.001–<0.001)	--	<0.001 (>-0.001–<0.001)	<0.001 (>-0.001–<0.001)
Norway	All causes	9·51 (-4·16–30·9)	4·8 (-0·472–13·7)	2·99 (-0·959–8·84)	2·69 (-2·57–10·4)
Norway	Diarrheal diseases	8·7 (-4·35–28·2)	4·04 (-0·73–11·9)	2·25 (-1·27–7·29)	2·65 (-2·48–10·2)
Norway	Lower respiratory infections	0·0773 (-0·127–0·374)	0·0311 (-0·0386–0·14)	0·00919 (-0·00208–0·0244)	0·0386 (-0·0889–0·217)
Norway	Malaria	0 (0–0)	0 (0–0)	--	--
Norway	Measles	0 (0–0)	--	0 (0–0)	0 (0–0)
Portugal	All causes	18·5 (-6·46–52·7)	10·3 (0·448–26·6)	5·6 (-0·473–15·5)	5·13 (-5·09–18·8)
Portugal	Diarrheal diseases	16·5 (-8·86–51·2)	8·31 (-1·67–23·7)	3·68 (-2·2–11·7)	5·07 (-4·95–18·5)
Portugal	Lower respiratory infections	0·111 (-0·189–0·49)	0·0471 (-0·059–0·195)	0·0114 (-0·00262–0·0287)	0·0551 (-0·127–0·286)
Portugal	Malaria	0 (0–0)	0 (0–0)	--	--
Portugal	Measles	<0.001 (>-0.001–<0.001)	--	<0.001 (>-0.001–<0.001)	<0.001 (>-0.001–<0.001)
San Marino	All causes	0·0631 (-0·0323–0·209)	0·0297 (-0·00289–0·0878)	0·0168 (-0·00646–0·0527)	0·0197 (-0·0185–0·0751)
San Marino	Diarrheal diseases	0·0616 (-0·033–0·207)	0·0283 (-0·00552–0·0866)	0·0155 (-0·00897–0·0518)	0·0196 (-0·0181–0·0747)
San Marino	Lower respiratory infections	<0.001 (>-0.001–0·00127)	<0.001 (>-0.001–<0.001)	<0.001 (>-0.001–<0.001)	<0.001 (>-0.001–<0.001)
San Marino	Malaria	0 (0–0)	0 (0–0)	--	--
San Marino	Measles	0 (0–0)	--	0 (0–0)	0 (0–0)
Spain	All causes	96·6 (-49·6–313)	47·2 (-7·96–140)	24·7 (-12·3–79·7)	29·3 (-28–110)
Spain	Diarrheal diseases	94·6 (-49·9–308)	45·4 (-8·83–137)	23·0 (-13·4–77·2)	29·1 (-27·5–109)
Spain	Lower respiratory infections	0·444 (-0·712–1·99)	0·182 (-0·215–0·753)	0·0485 (-0·00928–0·139)	0·223 (-0·481–1·15)
Spain	Malaria	0 (0–0)	0 (0–0)	--	--
Spain	Measles	<0.001 (>-0.001–0·00117)	--	<0.001 (>-0.001–0·00107)	<0.001 (>-0.001–<0.001)
Sweden	All causes	27·7 (-13·4–87·5)	13·5 (-1·75–38·6)	7·99 (-3·26–24·3)	8·11 (-7·67–30·1)
Sweden	Diarrheal diseases	26·5 (-14–86·0)	12·4 (-2·41–37·3)	6·88 (-4·11–23·1)	8·05 (-7·53–29·8)
Sweden	Lower respiratory infections	0·129 (-0·184–0·637)	0·0515 (-0·0563–0·248)	0·015 (-0·00308–0·0424)	0·0648 (-0·124–0·372)
Sweden	Malaria	0 (0–0)	0 (0–0)	--	--
Sweden	Measles	<0.001 (>-0.001–<0.001)	--	<0.001 (>-0.001–<0.001)	<0.001 (>-0.001–<0.001)
Switzerland	All causes	29·3 (-14·7–91·0)	13·9 (-2·31–39·3)	8·01 (-3·99–24·9)	8·74 (-8·31–31·9)
Switzerland	Diarrheal diseases	28·7 (-14·8–90·5)	13·4 (-2·51–38·6)	7·53 (-4·23–24·2)	8·69 (-8·17–31·7)
Switzerland	Lower respiratory infections	0·0941 (-0·158–0·427)	0·0377 (-0·0477–0·163)	0·0112 (-0·00262–0·0288)	0·0472 (-0·104–0·247)

Switzerland	Malaria	0 (0–0)	0 (0–0)	--	--
Switzerland	Measles	0·0012 (>-0.001–0·00396)	--	0·00114 (>-0.001–0·00367)	<0.001 (>-0.001–<0.001)
United Kingdom	All causes	139 (-59·1–424)	77·2 (-2·21–205)	41·7 (-4·56–106)	41·7 (-40·6–154)
United Kingdom	Diarrheal diseases	122 (-65·3–394)	59·9 (-11·8–174)	24·6 (-14·3–79·2)	41·2 (-39·6–152)
United Kingdom	Lower respiratory infections	0·867 (-1·62–4·1)	0·345 (-0·456–1·47)	0·0816 (-0·0197–0·201)	0·46 (-1·15–2·55)
United Kingdom	Malaria	0 (0–0)	0 (0–0)	--	--
United Kingdom	Measles	0·00516 (-0·00195–0·0148)	--	0·005 (-0·00187–0·0143)	<0.001 (>-0.001–<0.001)
**Latin America and Caribbean**	**All causes**	**6 860 (1 010–14 800)**	**5 390 (2 570–9 950)**	**3 760 (2 410–5 540)**	**1 340 (-1 690–4 800)**
**Latin America and Caribbean**	**Diarrheal diseases**	**3 240 (-2 180–9 330)**	**1 860 (-431–4 920)**	**420 (-256–1 310)**	**1 240 (-1 420–4 320)**
**Latin America and Caribbean**	**Lower respiratory infections**	**175 (-383–795)**	**80·3 (-108–333)**	**11·0 (-2·35–28·1)**	**97·6 (-259–504)**
**Latin America and Caribbean**	**Malaria**	**119 (-53·6–392)**	**119 (-53·6–392)**	**--**	**--**
**Latin America and Caribbean**	**Measles**	**0·0348 (-0·0147–0·115)**	**--**	**0·0268 (-0·00981–0·079)**	**0·00837 (-0·00381–0·0309)**
Andean Latin America	All causes	730 (-358–1 970)	462 (2·06–1 110)	156 (57·2–304)	263 (-330–913)
Andean Latin America	Diarrheal diseases	575 (-390–1 620)	324 (-71·4–835)	53·4 (-32·7–168)	245 (-278–824)
Andean Latin America	Lower respiratory infections	29·5 (-70·2–141)	12·6 (-16·9–53·4)	1·16 (-0·24–3·04)	18·0 (-49·8–95·7)
Andean Latin America	Malaria	24·2 (-10·7–80·1)	24·2 (-10·7–80·1)	--	--
Andean Latin America	Measles	<0.001 (>-0.001–<0.001)	--	<0.001 (>-0.001–<0.001)	<0.001 (>-0.001–<0.001)
Bolivia (Plurinational State of)	All causes	115 (-32·2–310)	79·0 (15·4–177)	40·2 (16·6–68·1)	34·7 (-48·5–137)
Bolivia (Plurinational State of)	Diarrheal diseases	66·6 (-45·8–206)	35·7 (-7·64–102)	7·29 (-4·41–23·8)	29·5 (-32·9–110)
Bolivia (Plurinational State of)	Lower respiratory infections	8·28 (-21·5–38·5)	3·36 (-4·7–14·0)	0·385 (-0·0744–0·984)	5·2 (-15·8–26·8)
Bolivia (Plurinational State of)	Malaria	7·37 (-3·31–26·2)	7·37 (-3·31–26·2)	--	--
Bolivia (Plurinational State of)	Measles	0 (0–0)	--	0 (0–0)	0 (0–0)
Ecuador	All causes	223 (-35·3–544)	157 (35·6–340)	89·1 (37·9–166)	58·8 (-76·2–208)
Ecuador	Diarrheal diseases	146 (-97·4–415)	83·8 (-20·2–225)	20·3 (-13·4–65·3)	54·7 (-66·5–188)
Ecuador	Lower respiratory infections	7·26 (-16·2–34·9)	3·31 (-4·5–14·5)	0·428 (-0·0812–1·14)	4·11 (-11·3–22·6)
Ecuador	Malaria	1·76 (-0·712–5·97)	1·76 (-0·712–5·97)	--	--
Ecuador	Measles	<0.001 (>-0.001–<0.001)	--	<0.001 (>-0.001–<0.001)	<0.001 (>-0.001–<0.001)
Peru	All causes	393 (-284–1 150)	226 (-59·2–610)	26·8 (-14·4–80·6)	169 (-206–583)
Peru	Diarrheal diseases	363 (-242–1 030)	204 (-43·6–528)	25·9 (-15–78·7)	160 (-180–539)

Peru	Lower respiratory infections	14·0 (-32·5–67·0)	5·98 (-7·98–25·2)	0·349 (-0·084–0·912)	8·65 (-24·1–46·3)
Peru	Malaria	15·1 (-6·81–52·0)	15·1 (-6·81–52·0)	--	--
Peru	Measles	0 (0–0)	--	0 (0–0)	0 (0–0)
Caribbean	All causes	2 420 (1 450–3 820)	2 250 (1 420–3 340)	2 040 (1 330–2 960)	135 (-168–495)
Caribbean	Diarrheal diseases	417 (-259–1 120)	258 (-62·1–665)	81·8 (-52·1–261)	123 (-136–427)
Caribbean	Lower respiratory infections	26·1 (-51·9–119)	14·4 (-19·4–62·7)	2·66 (-0·645–6·88)	11·9 (-30·8–66·2)
Caribbean	Malaria	17·4 (-9·47–64·4)	17·4 (-9·47–64·4)	--	--
Caribbean	Measles	0·0101 (-0·0039–0·037)	--	0·00935 (-0·00353–0·0327)	<0.001 (>-0.001–0·00397)
Antigua and Barbuda	All causes	1·22 (0·515–2·06)	1·07 (0·531–1·7)	0·984 (0·493–1·62)	0·0879 (-0·106–0·331)
Antigua and Barbuda	Diarrheal diseases	0·309 (-0·198–0·933)	0·168 (-0·0389–0·477)	0·0839 (-0·0606–0·27)	0·0817 (-0·0939–0·302)
Antigua and Barbuda	Lower respiratory infections	0·0146 (-0·024–0·061)	0·00686 (-0·0086–0·0283)	0·00225 (>-0.001–0·00607)	0·00627 (-0·0144–0·0319)
Antigua and Barbuda	Malaria	0 (0–0)	0 (0–0)	--	--
Antigua and Barbuda	Measles	0 (0–0)	--	0 (0–0)	0 (0–0)
Bahamas	All causes	1·77 (-0·0167–3·84)	1·34 (0·401–2·62)	0·99 (0·345–1·96)	0·298 (-0·36–1·1)
Bahamas	Diarrheal diseases	0·942 (-0·578–2·84)	0·534 (-0·126–1·5)	0·204 (-0·134–0·66)	0·276 (-0·303–0·99)
Bahamas	Lower respiratory infections	0·049 (-0·0849–0·243)	0·0238 (-0·0297–0·113)	0·00533 (-0·00114–0·0147)	0·0228 (-0·0539–0·133)
Bahamas	Malaria	0 (0–0)	0 (0–0)	--	--
Bahamas	Measles	0 (0–0)	--	0 (0–0)	0 (0–0)
Barbados	All causes	2·91 (1·1–5·63)	2·4 (1·11–4·53)	2·1 (0·959–4·02)	0·308 (-0·367–1·07)
Barbados	Diarrheal diseases	1·04 (-0·635–2·92)	0·555 (-0·128–1·46)	0·265 (-0·177–0·805)	0·291 (-0·325–0·996)
Barbados	Lower respiratory infections	0·0358 (-0·0586–0·164)	0·0163 (-0·0195–0·0723)	0·0048 (-0·00106–0·0134)	0·0166 (-0·0366–0·0912)
Barbados	Malaria	0 (0–0)	0 (0–0)	--	--
Barbados	Measles	0 (0–0)	--	0 (0–0)	0 (0–0)
Belize	All causes	7·46 (2·94–14·9)	6·4 (3·33–11·6)	5·29 (2·92–9·39)	0·967 (-1·13–3·53)
Belize	Diarrheal diseases	2·39 (-1·54–7·1)	1·39 (-0·317–3·81)	0·329 (-0·201–1·09)	0·901 (-0·957–3·25)
Belize	Lower respiratory infections	0·119 (-0·266–0·527)	0·0555 (-0·0775–0·222)	0·00712 (-0·00155–0·0184)	0·0661 (-0·177–0·328)
Belize	Malaria	0 (0–0)	0 (0–0)	--	--
Belize	Measles	0 (0–0)	--	0 (0–0)	0 (0–0)
Bermuda	All causes	0·236 (-0·0959–0·703)	0·125 (-0·00534–0·333)	0·0855 (-0·0146–0·243)	0·0576 (-0·0586–0·217)

Bermuda	Diarrheal diseases	0·201 (-0·107–0·626)	0·102 (-0·0205–0·296)	0·0549 (-0·0323–0·178)	0·0548 (-0·0542–0·2)
Bermuda	Lower respiratory infections	0·00436 (-0·00663–0·0183)	0·00196 (-0·00226–0·00777)	<0.001 (>-0.001–0·00143)	0·002 (-0·00414–0·00983)
Bermuda	Malaria	0 (0–0)	0 (0–0)	--	--
Bermuda	Measles	0·00968 (-0·00374–0·0356)	--	0·00897 (-0·0034–0·0314)	<0.001 (>-0.001–0·0038)
Cuba	All causes	38·3 (-18·6–108)	21·9 (-1·69–57·7)	9·69 (-0·52–26·5)	12·3 (-13·7–43·0)
Cuba	Diarrheal diseases	33·5 (-19–94·8)	17·9 (-3·56–47·4)	6·07 (-3·65–18·9)	11·7 (-12·1–39·5)
Cuba	Lower respiratory infections	1·24 (-2·28–5·7)	0·533 (-0·641–2·38)	0·102 (-0·0215–0·275)	0·667 (-1·62–3·56)
Cuba	Malaria	0 (0–0)	0 (0–0)	--	--
Cuba	Measles	0 (0–0)	--	0 (0–0)	0 (0–0)
Dominica	All causes	0·319 (-0·0244–0·731)	0·235 (0·0697–0·463)	0·166 (0·0555–0·313)	0·0574 (-0·0655–0·204)
Dominica	Diarrheal diseases	0·184 (-0·104–0·536)	0·105 (-0·0228–0·286)	0·0397 (-0·0249–0·121)	0·0529 (-0·0556–0·189)
Dominica	Lower respiratory infections	0·00954 (-0·016–0·0417)	0·00464 (-0·00566–0·0193)	0·001 (>-0.001–0·0025)	0·00445 (-0·00972–0·023)
Dominica	Malaria	0 (0–0)	0 (0–0)	--	--
Dominica	Measles	0 (0–0)	--	0 (0–0)	0 (0–0)
Dominican Republic	All causes	88·4 (-35·4–247)	57·8 (-0·797–144)	27·1 (4·05–60·1)	23·4 (-26·2–88·4)
Dominican Republic	Diarrheal diseases	70·9 (-41·6–215)	41·7 (-9·21–119)	12·0 (-7·88–39·7)	22·1 (-22·8–81·7)
Dominican Republic	Lower respiratory infections	2·51 (-4·46–12·0)	1·23 (-1·48–5·48)	0·192 (-0·0427–0·498)	1·23 (-2·9–6·84)
Dominican Republic	Malaria	0·0299 (-0·0178–0·111)	0·0299 (-0·0178–0·111)	--	--
Dominican Republic	Measles	0 (0–0)	--	0 (0–0)	0 (0–0)
Grenada	All causes	1·01 (0·0512–2·39)	0·776 (0·266–1·7)	0·557 (0·242–1·14)	0·169 (-0·204–0·617)
Grenada	Diarrheal diseases	0·544 (-0·318–1·61)	0·32 (-0·0763–0·887)	0·109 (-0·071–0·35)	0·159 (-0·18–0·567)
Grenada	Lower respiratory infections	0·0225 (-0·0401–0·106)	0·0113 (-0·0146–0·0514)	0·0023 (>-0.001–0·0063)	0·0103 (-0·0237–0·0581)
Grenada	Malaria	0 (0–0)	0 (0–0)	--	--
Grenada	Measles	0 (0–0)	--	0 (0–0)	0 (0–0)
Guyana	All causes	51·1 (28·8–81·1)	49·7 (29·0–77·4)	43·1 (26·6–63·4)	0·918 (-1·13–3·38)
Guyana	Diarrheal diseases	3·33 (-1·91–9·29)	2·05 (-0·465–5·39)	0·831 (-0·533–2·54)	0·814 (-0·908–2·87)
Guyana	Lower respiratory infections	0·264 (-0·476–1·1)	0·146 (-0·199–0·599)	0·0377 (-0·00929–0·0976)	0·104 (-0·256–0·525)
Guyana	Malaria	5·29 (-3·13–16·3)	5·29 (-3·13–16·3)	--	--
Guyana	Measles	0 (0–0)	--	0 (0–0)	0 (0–0)

Haiti	All causes	2 030 (1 270–3 120)	1 930 (1 220–2 800)	1 790 (1 160–2 550)	85·2 (-112–314)
Haiti	Diarrheal diseases	266 (-174–710)	172 (-43·3–430)	53·0 (-34·4–168)	76·1 (-88·7–261)
Haiti	Lower respiratory infections	20·1 (-41·6–91·7)	11·5 (-15·9–50·0)	2·11 (-0·512–5·48)	9·01 (-23·9–50·5)
Haiti	Malaria	11·4 (-7·01–48·0)	11·4 (-7·01–48·0)	--	--
Haiti	Measles	0 (0–0)	--	0 (0–0)	0 (0–0)
Jamaica	All causes	13·9 (0·3–32·6)	9·95 (3·3–19·6)	7·58 (2·71–14·3)	2·45 (-2·73–9·36)
Jamaica	Diarrheal diseases	8·01 (-4·51–24·4)	4·24 (-0·916–12·1)	1·99 (-1·24–6·51)	2·28 (-2·3–8·46)
Jamaica	Lower respiratory infections	0·359 (-0·631–1·7)	0·162 (-0·213–0·736)	0·0416 (-0·00977–0·113)	0·173 (-0·39–0·956)
Jamaica	Malaria	0 (0–0)	0 (0–0)	--	--
Jamaica	Measles	0 (0–0)	--	0 (0–0)	0 (0–0)
Puerto Rico	All causes	7·22 (-3·46–21·1)	3·92 (-0·258–10·2)	2·37 (-0·552–6·29)	1·86 (-2·05–6·83)
Puerto Rico	Diarrheal diseases	6·5 (-3·71–19·9)	3·28 (-0·702–9·31)	1·78 (-1·12–5·58)	1·8 (-1·84–6·52)
Puerto Rico	Lower respiratory infections	0·139 (-0·249–0·593)	0·0619 (-0·0833–0·246)	0·018 (-0·00505–0·0456)	0·0649 (-0·157–0·323)
Puerto Rico	Malaria	0 (0–0)	0 (0–0)	--	--
Puerto Rico	Measles	<0.001 (>-0.001–<0.001)	--	<0.001 (>-0.001–<0.001)	<0.001 (>-0.001–<0.001)
Saint Kitts and Nevis	All causes	0·301 (-0·0673–0·786)	0·21 (0·0221–0·489)	0·138 (0·029–0·282)	0·0597 (-0·0715–0·227)
Saint Kitts and Nevis	Diarrheal diseases	0·201 (-0·123–0·606)	0·115 (-0·0274–0·325)	0·0457 (-0·0307–0·15)	0·0557 (-0·0626–0·205)
Saint Kitts and Nevis	Lower respiratory infections	0·00889 (-0·017–0·0408)	0·00438 (-0·00607–0·0189)	<0.001 (>-0.001–0·00256)	0·00405 (-0·0104–0·0221)
Saint Kitts and Nevis	Malaria	0 (0–0)	0 (0–0)	--	--
Saint Kitts and Nevis	Measles	0 (0–0)	--	0 (0–0)	0 (0–0)
Saint Lucia	All causes	1·59 (0·391–3·33)	1·33 (0·462–2·73)	1·12 (0·376–2·42)	0·133 (-0·139–0·474)
Saint Lucia	Diarrheal diseases	0·625 (-0·289–1·72)	0·37 (-0·0727–0·964)	0·17 (-0·097–0·509)	0·126 (-0·119–0·428)
Saint Lucia	Lower respiratory infections	0·0213 (-0·0334–0·0873)	0·0118 (-0·0155–0·0485)	0·00317 (>-0.001–0·00798)	0·00733 (-0·0171–0·0368)
Saint Lucia	Malaria	0 (0–0)	0 (0–0)	--	--
Saint Lucia	Measles	0 (0–0)	--	0 (0–0)	0 (0–0)
Saint Vincent and the Grenadines	All causes	0·648 (0·0936–1·3)	0·512 (0·213–0·963)	0·39 (0·172–0·752)	0·0962 (-0·109–0·347)
Saint Vincent and the Grenadines	Diarrheal diseases	0·307 (-0·174–0·893)	0·179 (-0·0393–0·484)	0·0635 (-0·0396–0·198)	0·0883 (-0·0915–0·304)
Saint Vincent and the Grenadines	Lower respiratory infections	0·0171 (-0·0286–0·0788)	0·00849 (-0·0102–0·0382)	0·0017 (>-0.001–0·00456)	0·00797 (-0·0175–0·0441)
Saint Vincent and the Grenadines	Malaria	0 (0–0)	0 (0–0)	--	--

Saint Vincent and the Grenadines	Measles	0 (0–0)	--	0 (0–0)	0 (0–0)
Suriname	All causes	31·7 (16·8–50·1)	30·1 (18·1–45·6)	28·3 (17·1–42·8)	1·03 (-1·12–3·79)
Suriname	Diarrheal diseases	4·19 (-2·23–11·6)	2·61 (-0·577–6·9)	1·04 (-0·642–3·3)	0·962 (-0·98–3·47)
Suriname	Lower respiratory infections	0·165 (-0·268–0·704)	0·0926 (-0·114–0·382)	0·0214 (-0·00437–0·0567)	0·0642 (-0·146–0·336)
Suriname	Malaria	0·0952 (-0·053–0·386)	0·0952 (-0·053–0·386)	--	--
Suriname	Measles	0 (0–0)	--	0 (0–0)	0 (0–0)
Trinidad and Tobago	All causes	60·4 (34·9–95·0)	58·8 (34·1–92·0)	57·5 (33·3–90·8)	1·05 (-1·24–3·79)
Trinidad and Tobago	Diarrheal diseases	3·78 (-2·18–10·8)	2·18 (-0·49–5·97)	0·935 (-0·593–2·95)	0·987 (-1·05–3·47)
Trinidad and Tobago	Lower respiratory infections	0·153 (-0·272–0·628)	0·0779 (-0·103–0·304)	0·0205 (-0·00515–0·0525)	0·065 (-0·156–0·317)
Trinidad and Tobago	Malaria	0 (0–0)	0 (0–0)	--	--
Trinidad and Tobago	Measles	0 (0–0)	--	0 (0–0)	0 (0–0)
United States Virgin Islands	All causes	0·308 (-0·118–0·837)	0·189 (0·0178–0·467)	0·112 (0·014–0·264)	0·0779 (-0·0874–0·289)
United States Virgin Islands	Diarrheal diseases	0·245 (-0·135–0·736)	0·13 (-0·0266–0·368)	0·0567 (-0·034–0·183)	0·0742 (-0·0801–0·268)
United States Virgin Islands	Lower respiratory infections	0·00766 (-0·0139–0·0352)	0·00348 (-0·00462–0·015)	<0.001 (>-0.001–0·00249)	0·0037 (-0·00877–0·0198)
United States Virgin Islands	Malaria	0 (0–0)	0 (0–0)	--	--
United States Virgin Islands	Measles	0 (0–0)	--	0 (0–0)	0 (0–0)
Central Latin America	All causes	2 690 (-124–6 270)	2 010 (720–3 980)	1 200 (729–1 910)	658 (-823–2 330)
Central Latin America	Diarrheal diseases	1 570 (-1 130–4 540)	915 (-225–2 430)	173 (-109–527)	631 (-745–2 210)
Central Latin America	Lower respiratory infections	45·3 (-106–208)	21·1 (-28·2–89·6)	1·97 (-0·401–5·1)	26·7 (-72·5–140)
Central Latin America	Malaria	43·2 (-23·9–141)	43·2 (-23·9–141)	--	--
Central Latin America	Measles	0·0246 (-0·00979–0·0758)	--	0·0174 (-0·00619–0·0507)	0·00756 (-0·00348–0·0279)
Colombia	All causes	364 (-231–1 100)	214 (-52·7–613)	51·3 (-28·2–154)	125 (-143–453)
Colombia	Diarrheal diseases	341 (-212–1 030)	195 (-44·6–544)	47·7 (-30·2–148)	121 (-133–435)
Colombia	Lower respiratory infections	8·11 (-16–35·3)	3·73 (-4·76–15·0)	0·451 (-0·0944–1·22)	4·38 (-10·5–21·8)
Colombia	Malaria	11·7 (-6·18–43·0)	11·7 (-6·18–43·0)	--	--
Colombia	Measles	0 (0–0)	--	0 (0–0)	0 (0–0)
Costa Rica	All causes	12·5 (-6·71–37·5)	7·02 (-1·26–19·3)	2·85 (-1·25–8·52)	3·51 (-4·04–12·9)
Costa Rica	Diarrheal diseases	11·8 (-6·57–35·5)	6·49 (-1·43–18·1)	2·56 (-1·58–7·98)	3·37 (-3·72–12·1)
Costa Rica	Lower respiratory infections	0·295 (-0·512–1·45)	0·138 (-0·179–0·64)	0·0288 (-0·00681–0·0827)	0·138 (-0·32–0·787)

Costa Rica	Malaria	0·136 (-0·0459–0·579)	0·136 (-0·0459–0·579)	--	--
Costa Rica	Measles	0 (0–0)	--	0 (0–0)	0 (0–0)
El Salvador	All causes	48·9 (-21·8–133)	32·0 (0·288–79·3)	11·7 (2·92–24·9)	16·3 (-21·7–58·5)
El Salvador	Diarrheal diseases	40·2 (-27·5–117)	24·0 (-5·18–66·0)	4·32 (-2·73–14·6)	15·6 (-19·7–54·4)
El Salvador	Lower respiratory infections	1·33 (-2·92–6·5)	0·636 (-0·819–2·81)	0·0604 (-0·0123–0·157)	0·743 (-2·00–4·16)
El Salvador	Malaria	0 (0–0)	0 (0–0)	--	--
El Salvador	Measles	0 (0–0)	--	0 (0–0)	0 (0–0)
Guatemala	All causes	357 (-312–1 010)	233 (-73·3–614)	24·6 (-14·2–74·4)	149 (-212–531)
Guatemala	Diarrheal diseases	339 (-273–918)	221 (-63·7–574)	22·7 (-15·7–71·4)	142 (-187–493)
Guatemala	Lower respiratory infections	11·5 (-33·9–55·8)	6·06 (-8·81–26·9)	0·273 (-0·0455–0·744)	6·96 (-23·3–39·1)
Guatemala	Malaria	4·79 (-3·16–18·1)	4·79 (-3·16–18·1)	--	--
Guatemala	Measles	0 (0–0)	--	0 (0–0)	0 (0–0)
Honduras	All causes	97·5 (-65·5–285)	64·5 (-16·3–173)	11·8 (-3·56–32·2)	33·6 (-42·8–125)
Honduras	Diarrheal diseases	84·0 (-55·1–246)	52·9 (-12·2–146)	8·79 (-5·19–27·9)	31·4 (-36·6–112)
Honduras	Lower respiratory infections	4·04 (-8·46–18·9)	2·08 (-2·59–8·89)	0·167 (-0·0345–0·456)	2·22 (-5·59–11·9)
Honduras	Malaria	6·7 (-3·51–20·1)	6·7 (-3·51–20·1)	--	--
Honduras	Measles	0 (0–0)	--	0 (0–0)	0 (0–0)
Mexico	All causes	1 330 (588–2 350)	1 140 (696–1 750)	973 (626–1 390)	174 (-211–626)
Mexico	Diarrheal diseases	386 (-267–1 180)	208 (-46·2–579)	45·6 (-27–145)	167 (-190–589)
Mexico	Lower respiratory infections	12·5 (-28·7–56·5)	5·11 (-6·81–20·8)	0·638 (-0·133–1·66)	7·74 (-20·8–39·4)
Mexico	Malaria	0·966 (-0·757–3·17)	0·966 (-0·757–3·17)	--	--
Mexico	Measles	0 (0–0)	--	0 (0–0)	0 (0–0)
Nicaragua	All causes	60·3 (-37·6–190)	36·4 (-8·4–106)	7·39 (-3·01–22·9)	22·2 (-26·8–84·7)
Nicaragua	Diarrheal diseases	54·3 (-34·3–169)	31·3 (-6·77–90·3)	6·38 (-3·75–21·3)	21·2 (-23·8–79·9)
Nicaragua	Lower respiratory infections	1·76 (-3·87–8·26)	0·806 (-1·08–3·51)	0·0839 (-0·0177–0·229)	1·01 (-2·65–5·3)
Nicaragua	Malaria	3·31 (-2·35–14·0)	3·31 (-2·35–14·0)	--	--
Nicaragua	Measles	0 (0–0)	--	0 (0–0)	0 (0–0)
Panama	All causes	71·1 (-50·5–212)	38·6 (-8·62–105)	7·3 (-4·13–21·3)	30·8 (-36·7–110)
Panama	Diarrheal diseases	68·8 (-48·5–205)	36·8 (-8·18–99·4)	7·02 (-4·33–21·0)	30·2 (-35·1–107)

Panama	Lower respiratory infections	0·896 (-2·04–4·43)	0·363 (-0·479–1·63)	0·0349 (-0·00768–0·0945)	0·56 (-1·52–3·08)
Panama	Malaria	1·2 (-0·611–5·13)	1·2 (-0·611–5·13)	--	--
Panama	Measles	0 (0–0)	--	0 (0–0)	0 (0–0)
Venezuela (Bolivarian Republic of)	All causes	348 (-108–870)	241 (25·3–515)	113 (21·2–290)	103 (-131–374)
Venezuela (Bolivarian Republic of)	Diarrheal diseases	244 (-182–709)	139 (-34·3–381)	28·0 (-18·2–88·6)	100 (-122–360)
Venezuela (Bolivarian Republic of)	Lower respiratory infections	4·92 (-11·2–23·3)	2·17 (-2·81–9·42)	0·235 (-0·0468–0·621)	2·95 (-8·04–16·0)
Venezuela (Bolivarian Republic of)	Malaria	14·4 (-7·74–71·2)	14·4 (-7·74–71·2)	--	--
Venezuela (Bolivarian Republic of)	Measles	0·0246 (-0·00979–0·0758)	--	0·0174 (-0·00619–0·0507)	0·00756 (-0·00348–0·0279)
Tropical Latin America	All causes	1 030 (-330–2 810)	679 (106–1 620)	362 (151–692)	286 (-363–1 100)
Tropical Latin America	Diarrheal diseases	676 (-381–2 080)	367 (-72·5–1 040)	112 (-63·1–367)	245 (-258–900)
Tropical Latin America	Lower respiratory infections	73·6 (-149–326)	32·2 (-41·7–131)	5·2 (-1·06–13·2)	41·0 (-103–206)
Tropical Latin America	Malaria	34·4 (-16·4–128)	34·4 (-16·4–128)	--	--
Tropical Latin America	Measles	0 (0–0)	--	0 (0–0)	0 (0–0)
Brazil	All causes	983 (-338–2 710)	645 (89·5–1 560)	337 (135–663)	276 (-352–1 060)
Brazil	Diarrheal diseases	653 (-368–2 010)	356 (-70·3–1 010)	109 (-61·4–357)	236 (-249–865)
Brazil	Lower respiratory infections	72·6 (-147–322)	31·8 (-41·2–130)	5·16 (-1·06–13·1)	40·3 (-101–203)
Brazil	Malaria	34·4 (-16·4–128)	34·4 (-16·4–128)	--	--
Brazil	Measles	0 (0–0)	--	0 (0–0)	0 (0–0)
Paraguay	All causes	45·7 (7·64–105)	33·8 (16·5–62·5)	24·7 (13·7–41·6)	10·1 (-10·6–39·8)
Paraguay	Diarrheal diseases	22·9 (-13–75·3)	11·6 (-2·27–35·2)	2·9 (-1·64–9·85)	9·4 (-8·97–36·2)
Paraguay	Lower respiratory infections	1·04 (-2·09–4·73)	0·365 (-0·454–1·51)	0·042 (-0·00831–0·104)	0·664 (-1·59–3·32)
Paraguay	Malaria	0 (0–0)	0 (0–0)	--	--
Paraguay	Measles	0 (0–0)	--	0 (0–0)	0 (0–0)
**North Africa and Middle East**	**All causes**	**79 000 (47 000–119 000)**	**74 200 (46 600–106 000)**	**67 900 (43 400–95 900)**	**3 620 (-4 420–13 200)**
**North Africa and Middle East**	**Diarrheal diseases**	**10 500 (-6 810–29 500)**	**6 250 (-1 490–16 300)**	**2 420 (-1 510–7 440)**	**3 290 (-3 610–11 600)**
**North Africa and Middle East**	**Lower respiratory infections**	**566 (-1 310–2 430)**	**310 (-474–1 310)**	**78·9 (-17·3–211)**	**267 (-718–1 450)**
**North Africa and Middle East**	**Malaria**	**2 410 (-2 520–6 740)**	**2 410 (-2 520–6 740)**	**--**	**--**
**North Africa and Middle East**	**Measles**	**219 (-101–757)**	**--**	**166 (-66·9–552)**	**61·4 (-33·4–282)**
North Africa and Middle East	All causes	79 000 (47 000–119 000)	74 200 (46 600–106 000)	67 900 (43 400–95 900)	3 620 (-4 420–13 200)

North Africa and Middle East	Diarrheal diseases	10 500 (-6 810–29 500)	6 250 (-1 490–16 300)	2 420 (-1 510–7 440)	3 290 (-3 610–11 600)
North Africa and Middle East	Lower respiratory infections	566 (-1 310–2 430)	310 (-474–1 310)	78·9 (-17·3–211)	267 (-718–1 450)
North Africa and Middle East	Malaria	2 410 (-2 520–6 740)	2 410 (-2 520–6 740)	--	--
North Africa and Middle East	Measles	219 (-101–757)	--	166 (-66·9–552)	61·4 (-33·4–282)
Afghanistan	All causes	9 210 (4 310–15 100)	8 440 (4 990–13 000)	7 260 (4 440–11 000)	683 (-893–2 570)
Afghanistan	Diarrheal diseases	2 010 (-1 340–5 610)	1 340 (-341–3 580)	389 (-247–1 240)	603 (-706–2 120)
Afghanistan	Lower respiratory infections	130 (-311–571)	78·6 (-117–328)	16·3 (-3·99–44·2)	58·5 (-169–315)
Afghanistan	Malaria	206 (-156–705)	206 (-156–705)	--	--
Afghanistan	Measles	54·1 (-25·9–184)	--	34·9 (-13·3–109)	22·1 (-12·9–93·8)
Algeria	All causes	2 750 (1 320–4 560)	2 420 (1 440–3 730)	2 240 (1 360–3 260)	227 (-252–840)
Algeria	Diarrheal diseases	622 (-354–1 870)	333 (-65·6–923)	134 (-79·4–428)	210 (-222–737)
Algeria	Lower respiratory infections	23·2 (-42·3–104)	9·98 (-12·4–43·8)	2·9 (-0·614–7·86)	12·0 (-29–63·8)
Algeria	Malaria	0 (0–0)	0 (0–0)	--	--
Algeria	Measles	26·9 (-12–93·8)	--	22·0 (-8·52–77·3)	5·38 (-2·96–25·9)
Bahrain	All causes	13·0 (-2·07–34·4)	8·54 (1·75–21·3)	5·77 (1·38–15·6)	2·87 (-3·65–10·5)
Bahrain	Diarrheal diseases	9·34 (-5·35–28·0)	5·02 (-1·04–14·1)	2·37 (-1·42–7·41)	2·71 (-3·03–9·63)
Bahrain	Lower respiratory infections	0·357 (-0·648–1·51)	0·163 (-0·214–0·691)	0·0515 (-0·0106–0·14)	0·164 (-0·412–0·838)
Bahrain	Malaria	0 (0–0)	0 (0–0)	--	--
Bahrain	Measles	<0.001 (>-0.001–0·00116)	--	<0.001 (>-0.001–0·00106)	<0.001 (>-0.001–<0.001)
Egypt	All causes	9 560 (5 210–15 300)	8 480 (5 300–12 200)	7 900 (5 060–11 000)	778 (-907–2 870)
Egypt	Diarrheal diseases	2 060 (-1 300–5 920)	1 050 (-220–2 900)	490 (-299–1 520)	721 (-758–2 560)
Egypt	Lower respiratory infections	101 (-197–453)	40·2 (-51·1–172)	14·5 (-3·05–38·3)	55·1 (-136–290)
Egypt	Malaria	0 (0–0)	0 (0–0)	--	--
Egypt	Measles	6·77 (-2·8–25·6)	--	5·16 (-1·84–19·2)	1·77 (-0·83–7·96)
Iran (Islamic Republic of)	All causes	2 270 (1 330–3 500)	2 120 (1 280–3 180)	2 000 (1 170–2 950)	95·0 (-108–343)
Iran (Islamic Republic of)	Diarrheal diseases	338 (-185–999)	192 (-40·6–537)	86·5 (-51·6–275)	90·0 (-97·5–321)
Iran (Islamic Republic of)	Lower respiratory infections	11·6 (-20·4–50·6)	5·8 (-7·5–24·6)	1·6 (-0·387–4·14)	5·04 (-11·9–26·8)
Iran (Islamic Republic of)	Malaria	3·92 (-1·89–15·8)	3·92 (-1·89–15·8)	--	--
Iran (Islamic Republic of)	Measles	0·0149 (-0·00578–0·0438)	--	0·0134 (-0·00494–0·0394)	0·00167 (>-0.001–0·00713)

Iraq	All causes	3 460 (1 590–6 240)	2 960 (1 790–4 800)	2 650 (1 630–4 070)	354 (-401–1 280)
Iraq	Diarrheal diseases	991 (-627–2 860)	543 (-120–1 460)	221 (-137–671)	330 (-352–1 140)
Iraq	Lower respiratory infections	33·0 (-64·2–166)	14·5 (-18·6–69·7)	4·25 (-0·89–12·1)	17·1 (-42·6–103)
Iraq	Malaria	0 (0–0)	0 (0–0)	--	--
Iraq	Measles	29·2 (-13·6–104)	--	23·0 (-9·56–81·0)	6·78 (-3·86–30·8)
Jordan	All causes	174 (-86·3–492)	101 (-6·24–250)	47·7 (1·39–121)	55·4 (-61·7–197)
Jordan	Diarrheal diseases	150 (-87·8–441)	80·3 (-16·6–217)	28·7 (-17·1–88·6)	52·3 (-54·3–182)
Jordan	Lower respiratory infections	5·69 (-10·8–26·5)	2·45 (-3·1–10·5)	0·495 (-0·105–1·3)	3·07 (-7·24–16·6)
Jordan	Malaria	0 (0–0)	0 (0–0)	--	--
Jordan	Measles	0·00913 (-0·00325–0·0272)	--	0·00793 (-0·0027–0·0234)	0·00127 (>-0.001–0·00573)
Kuwait	All causes	26·8 (-13·5–81·0)	14·3 (-1·26–38·3)	8·01 (-1·83–22·1)	7·62 (-8·8–28·9)
Kuwait	Diarrheal diseases	23·2 (-13·6–71·6)	11·9 (-2·49–33·8)	5·66 (-3·61–18·1)	6·97 (-7·42–25·5)
Kuwait	Lower respiratory infections	1·27 (-2·27–5·66)	0·558 (-0·71–2·39)	0·137 (-0·0277–0·351)	0·622 (-1·48–3·25)
Kuwait	Malaria	0 (0–0)	0 (0–0)	--	--
Kuwait	Measles	0·397 (-0·167–1·47)	--	0·368 (-0·155–1·35)	0·0297 (-0·0153–0·147)
Lebanon	All causes	188 (3·42–444)	132 (55·8–252)	109 (44·4–210)	40·1 (-44·8–142)
Lebanon	Diarrheal diseases	97·2 (-62·5–286)	46·7 (-9·34–127)	21·1 (-12·4–64·9)	37·8 (-40·8–131)
Lebanon	Lower respiratory infections	2·54 (-5·6–12·0)	0·934 (-1·26–4·15)	0·316 (-0·0774–0·87)	1·47 (-4·09–7·99)
Lebanon	Malaria	0 (0–0)	0 (0–0)	--	--
Lebanon	Measles	3·21 (-1·4–11·3)	--	2·52 (-0·967–8·89)	0·747 (-0·343–3·37)
Libya	All causes	453 (252–755)	416 (241–665)	393 (231–631)	27·0 (-32·9–101)
Libya	Diarrheal diseases	71·6 (-46·5–210)	38·7 (-8·45–107)	16·4 (-10·5–51·4)	24·3 (-27·3–87·7)
Libya	Lower respiratory infections	4·57 (-8·32–19·6)	1·95 (-2·34–7·92)	0·596 (-0·13–1·61)	2·43 (-5·64–12·2)
Libya	Malaria	0 (0–0)	0 (0–0)	--	--
Libya	Measles	0·787 (-0·317–2·85)	--	0·607 (-0·222–2·06)	0·201 (-0·094–0·931)
Morocco	All causes	481 (51·5–1 010)	351 (174–604)	286 (155–462)	95·3 (-110–356)
Morocco	Diarrheal diseases	228 (-146–678)	114 (-24·3–319)	46·5 (-28·7–148)	85·7 (-90·9–308)
Morocco	Lower respiratory infections	13·1 (-24·9–57·8)	5·02 (-6·17–21·9)	1·32 (-0·263–3·64)	7·58 (-17·8–38·9)
Morocco	Malaria	0 (0–0)	0 (0–0)	--	--

Morocco	Measles	8·09 (-3·26–29·7)	--	6·18 (-2·34–22·4)	2·03 (-0·952–9·3)
Oman	All causes	421 (252–622)	399 (244–591)	377 (230–562)	12·6 (-15·4–47·5)
Oman	Diarrheal diseases	56·2 (-32·9–157)	35·5 (-8·78–95·9)	15·6 (-10·6–49·2)	11·5 (-12·5–41·6)
Oman	Lower respiratory infections	2·92 (-4·78–12·7)	1·71 (-2·27–7·5)	0·449 (-0·111–1·28)	1·03 (-2·22–5·73)
Oman	Malaria	0·721 (-0·354–4·05)	0·721 (-0·354–4·05)	--	--
Oman	Measles	0·00726 (-0·00286–0·021)	--	0·00653 (-0·00237–0·0181)	<0.001 (>-0.001–0·00338)
Palestine	All causes	77·7 (-36·7–229)	41·6 (1·12–104)	21·9 (1·92–52·1)	27·8 (-29·8–104)
Palestine	Diarrheal diseases	64·0 (-37·9–200)	31·0 (-6·00–89·2)	11·2 (-6·5–36·6)	25·9 (-26·3–93·3)
Palestine	Lower respiratory infections	3·04 (-5·74–14·9)	1·13 (-1·37–5·14)	0·244 (-0·0529–0·64)	1·81 (-4·28–10·1)
Palestine	Malaria	0 (0–0)	0 (0–0)	--	--
Palestine	Measles	1·06 (-0·43–3·6)	--	0·903 (-0·357–2·94)	0·161 (-0·0825–0·742)
Qatar	All causes	13·4 (-7·16–41·2)	6·6 (-0·848–18·6)	3·94 (-1·38–11·8)	4·17 (-4·56–15·8)
Qatar	Diarrheal diseases	12·1 (-6·53–37·8)	5·79 (-1·09–16·7)	3·1 (-1·73–9·87)	3·92 (-3·98–14·5)
Qatar	Lower respiratory infections	0·464 (-0·785–2·12)	0·185 (-0·225–0·806)	0·0559 (-0·0135–0·146)	0·242 (-0·543–1·29)
Qatar	Malaria	0 (0–0)	0 (0–0)	--	--
Qatar	Measles	0·168 (-0·0703–0·584)	--	0·155 (-0·0625–0·527)	0·0141 (-0·00661–0·063)
Saudi Arabia	All causes	2 580 (1 420–4 270)	2 370 (1 420–3 760)	2 290 (1 340–3 560)	113 (-132–416)
Saudi Arabia	Diarrheal diseases	408 (-246–1 190)	212 (-46·9–582)	125 (-82·9–390)	108 (-119–389)
Saudi Arabia	Lower respiratory infections	12·1 (-19·4–53·5)	5·31 (-6·68–24·3)	2·6 (-0·449–7·04)	4·99 (-11·5–28·1)
Saudi Arabia	Malaria	0·898 (-0·437–3·01)	0·898 (-0·437–3·01)	--	--
Saudi Arabia	Measles	1·54 (-0·572–5·25)	--	1·45 (-0·518–4·9)	0·108 (-0·0543–0·56)
Sudan	All causes	18 900 (12 000–27 800)	18 400 (11 700–27 400)	16 900 (11 000–23 600)	361 (-475–1 330)
Sudan	Diarrheal diseases	1 340 (-963–3 450)	907 (-245–2 170)	365 (-261–1 110)	331 (-393–1 130)
Sudan	Lower respiratory infections	61·4 (-161–259)	39·7 (-70–164)	10·8 (-2·4–30·0)	23·7 (-73·3–131)
Sudan	Malaria	1 030 (-940–3 550)	1 030 (-940–3 550)	--	--
Sudan	Measles	29·1 (-14·1–96·0)	--	24·4 (-11–76·9)	6 (-3·21–25·8)
Syrian Arab Republic	All causes	4 760 (2 920–6 840)	4 660 (2 890–6 620)	4 600 (2 860–6 510)	73·7 (-95·7–282)
Syrian Arab Republic	Diarrheal diseases	187 (-131–528)	107 (-25·5–292)	43·0 (-27·7–138)	63·0 (-73·4–228)
Syrian Arab Republic	Lower respiratory infections	14·4 (-28–64·1)	6·67 (-8·2–28·0)	2·38 (-0·478–6·67)	7·28 (-18·2–38·2)

Syrian Arab Republic	Malaria	0 (0–0)	0 (0–0)	--	--
Syrian Arab Republic	Measles	10·5 (-4·77–37·7)	--	7·45 (-3·01–25·0)	3·46 (-1·87–15·3)
Tunisia	All causes	182 (-6·27–458)	117 (48·8–231)	90·2 (40·7–159)	46·7 (-50·1–176)
Tunisia	Diarrheal diseases	112 (-70–358)	50·9 (-10·5–147)	23·5 (-14–77·0)	44·5 (-44·7–165)
Tunisia	Lower respiratory infections	3·11 (-5·92–13·8)	1·04 (-1·3–4·29)	0·316 (-0·0742–0·801)	1·9 (-4·42–9·61)
Tunisia	Malaria	0 (0–0)	0 (0–0)	--	--
Tunisia	Measles	1·57 (-0·603–5·83)	--	1·26 (-0·455–4·44)	0·325 (-0·141–1·44)
Türkiye	All causes	500 (-313–1 490)	246 (-49·4–664)	95·6 (-43·5–289)	194 (-213–696)
Türkiye	Diarrheal diseases	467 (-280–1 380)	230 (-46·2–634)	77·7 (-46·3–240)	184 (-192–648)
Türkiye	Lower respiratory infections	14·5 (-27·6–64·6)	5·44 (-6·72–22·5)	0·929 (-0·216–2·32)	8·77 (-20·2–43·5)
Türkiye	Malaria	0 (0–0)	0 (0–0)	--	--
Türkiye	Measles	7·84 (-3·04–29·8)	--	6·46 (-2·32–24·3)	1·44 (-0·72–6·72)
United Arab Emirates	All causes	392 (210–648)	365 (212–567)	351 (204–544)	14·7 (-17·6–53·8)
United Arab Emirates	Diarrheal diseases	54·0 (-32·1–152)	31·4 (-7·19–83·2)	15·4 (-9·86–47·0)	13·4 (-14·9–46·4)
United Arab Emirates	Lower respiratory infections	2·72 (-4·47–10·4)	1·4 (-1·8–5·68)	0·6 (-0·123–1·49)	0·993 (-2·36–5·05)
United Arab Emirates	Malaria	0 (0–0)	0 (0–0)	--	--
United Arab Emirates	Measles	2·84 (-1·23–9·63)	--	2·57 (-1·04–8·62)	0·321 (-0·165–1·44)
Yemen	All causes	22 500 (13 800–33 100)	22 000 (14 100–31 800)	20 300 (13 000–29 100)	403 (-613–1 400)
Yemen	Diarrheal diseases	1 220 (-919–2 930)	871 (-249–2 030)	296 (-197–859)	339 (-421–1 130)
Yemen	Lower respiratory infections	125 (-360–511)	87·2 (-152–356)	18·0 (-3·94–48·2)	53·4 (-159–300)
Yemen	Malaria	1 160 (-1 260–3 980)	1 160 (-1 260–3 980)	--	--
Yemen	Measles	34·3 (-18·1–127)	--	26·0 (-11·3–91·1)	10·5 (-6·39–48·1)
**South Asia**	**All causes**	**739 000 (469 000–1 060 000)**	**714 000 (459 000–1 010 000)**	**677 000 (436 000–959 000)**	**21 300 (-28 100–77 300)**
**South Asia**	**Diarrheal diseases**	**74 600 (-56 600–195 000)**	**52 200 (-15 300–134 000)**	**19 200 (-13 400–61 600)**	**19 600 (-23 800–69 400)**
**South Asia**	**Lower respiratory infections**	**3 270 (-8 990–13 100)**	**2 120 (-3 840–8 250)**	**612 (-133–1 570)**	**1 300 (-3 980–6 690)**
**South Asia**	**Malaria**	**3 580 (-3 200–10 100)**	**3 580 (-3 200–10 100)**	**--**	**--**
**South Asia**	**Measles**	**1 420 (-772–5 010)**	**--**	**1 110 (-509–3 740)**	**394 (-224–1 770)**
South Asia	All causes	739 000 (469 000–1 060 000)	714 000 (459 000–1 010 000)	677 000 (436 000–959 000)	21 300 (-28 100–77 300)
South Asia	Diarrheal diseases	74 600 (-56 600–195 000)	52 200 (-15 300–134 000)	19 200 (-13 400–61 600)	19 600 (-23 800–69 400)

South Asia	Lower respiratory infections	3 270 (-8 990–13 100)	2 120 (-3 840–8 250)	612 (-133–1 570)	1 300 (-3 980–6 690)
South Asia	Malaria	3 580 (-3 200–10 100)	3 580 (-3 200–10 100)	--	--
South Asia	Measles	1 420 (-772–5 010)	--	1 110 (-509–3 740)	394 (-224–1 770)
Bangladesh	All causes	37 300 (23 700–54 900)	36 200 (23 000–52 400)	34 600 (21 800–49 300)	956 (-1 320–3 690)
Bangladesh	Diarrheal diseases	3 110 (-2 260–8 020)	2 150 (-591–5 340)	756 (-534–2 350)	844 (-1 050–3 040)
Bangladesh	Lower respiratory infections	259 (-701–1 110)	168 (-298–714)	37·1 (-8·14–102)	105 (-328–589)
Bangladesh	Malaria	103 (-103–389)	103 (-103–389)	--	--
Bangladesh	Measles	42·2 (-18·2–137)	--	35·6 (-13·7–114)	8·02 (-3·86–32·6)
Bhutan	All causes	28·8 (6·23–54·7)	23·8 (12·2–39·3)	17·9 (10·5–27·0)	4·56 (-5·68–16·3)
Bhutan	Diarrheal diseases	12·6 (-8·62–34·7)	7·81 (-1·72–20·1)	2·14 (-1·33–6·73)	4·36 (-5·02–15·2)
Bhutan	Lower respiratory infections	0·391 (-1·02–1·72)	0·204 (-0·305–0·817)	0·0363 (-0·00866–0·0961)	0·2 (-0·643–1·05)
Bhutan	Malaria	0·0766 (-0·0456–0·246)	0·0766 (-0·0456–0·246)	--	--
Bhutan	Measles	<0.001 (>-0.001–0·0023)	--	<0.001 (>-0.001–0·00186)	<0.001 (>-0.001–<0.001)
India	All causes	574 000 (365 000–817 000)	556 000 (359 000–786 000)	531 000 (342 000–748 000)	15 000 (-19 600–56 100)
India	Diarrheal diseases	53 900 (-41 900–138 000)	37 900 (-11 400–96 100)	14 400 (-10 300–47 100)	13 800 (-17 100–50 700)
India	Lower respiratory infections	2 050 (-5 640–7 900)	1 340 (-2 470–5 070)	428 (-92·6–1 070)	787 (-2 390–3 940)
India	Malaria	1 950 (-1 900–6 150)	1 950 (-1 900–6 150)	--	--
India	Measles	1 320 (-722–4 680)	--	1 030 (-479–3 480)	369 (-211–1 640)
Nepal	All causes	3 600 (2 220–5 250)	3 420 (2 120–4 890)	3 210 (1 970–4 630)	152 (-211–576)
Nepal	Diarrheal diseases	438 (-299–1 150)	300 (-75·1–742)	92·4 (-58·8–289)	128 (-159–450)
Nepal	Lower respiratory infections	41·2 (-101–178)	26·0 (-40·1–105)	5·4 (-1·17–14·4)	17·7 (-54·7–94·9)
Nepal	Malaria	4·07 (-3·21–13·3)	4·07 (-3·21–13·3)	--	--
Nepal	Measles	23·3 (-10·8–87·4)	--	18·6 (-7·54–63·1)	5·57 (-3·14–25·7)
Pakistan	All causes	124 000 (76 600–187 000)	118 000 (74 300–172 000)	109 000 (69 700–152 000)	5 230 (-7 010–18 700)
Pakistan	Diarrheal diseases	17 200 (-12 200–47 300)	11 800 (-3 220–31 500)	3 940 (-2 570–12 400)	4 830 (-5 730–16 800)
Pakistan	Lower respiratory infections	918 (-2 550–3 880)	586 (-1 020–2 460)	142 (-30·7–377)	387 (-1 240–2 140)
Pakistan	Malaria	1 520 (-1 630–4 640)	1 520 (-1 630–4 640)	--	--
Pakistan	Measles	37·1 (-17·8–136)	--	27·9 (-11·3–98·3)	11·3 (-5·57–49·0)
**Southeast Asia, East Asia, and Oceania**	**All causes**	**142 000 (82 400–219 000)**	**132 000 (83 900–193 000)**	**118 000 (75 100–169 000)**	**8 750 (-11 200–31 900)**

**Southeast Asia, East Asia, and Oceania**	**Diarrheal diseases**	**27 000 (-18 700–76 300)**	**17 800 (-4 580–47 100)**	**5 450 (-3 460–16 900)**	**8 180 (-9 740–29 200)**
**Southeast Asia, East Asia, and Oceania**	**Lower respiratory infections**	**1 000 (-2 340–4 340)**	**581 (-875–2 370)**	**108 (-24·3–275)**	**462 (-1 300–2 420)**
**Southeast Asia, East Asia, and Oceania**	**Malaria**	**1 450 (-1 160–4 000)**	**1 450 (-1 160–4 000)**	**--**	**--**
**Southeast Asia, East Asia, and Oceania**	**Measles**	**421 (-192–1 500)**	**--**	**329 (-133–1 130)**	**106 (-57–463)**
East Asia	All causes	4 610 (-1 740–11 600)	3 290 (286–7 220)	1 640 (634–3 090)	1 160 (-1 540–4 300)
East Asia	Diarrheal diseases	3 270 (-2 010–9 030)	2 080 (-504–5 360)	557 (-322–1 730)	1 020 (-1 140–3 590)
East Asia	Lower respiratory infections	281 (-617–1 290)	148 (-218–623)	22·1 (-5·17–56·2)	137 (-368–749)
East Asia	Malaria	5·55 (-7·17–22·3)	5·55 (-7·17–22·3)	--	--
East Asia	Measles	1·18 (-0·448–3·86)	--	1·15 (-0·437–3·72)	0·0286 (-0·0116–0·136)
China	All causes	3 570 (-946–9 080)	2 510 (467–5 490)	1 430 (607–2 610)	859 (-1 110–3 200)
China	Diarrheal diseases	2 300 (-1 280–6 470)	1 380 (-288–3 670)	399 (-227–1 250)	734 (-796–2 560)
China	Lower respiratory infections	253 (-532–1 170)	129 (-181–549)	20·3 (-4·72–51·7)	124 (-327–675)
China	Malaria	0 (0–0)	0 (0–0)	--	--
China	Measles	0·0545 (-0·0217–0·161)	--	0·0481 (-0·0178–0·141)	0·0069 (-0·00316–0·0275)
Democratic People's Republic of Korea	All causes	794 (-649–1 980)	624 (-178–1 440)	151 (-25·8–384)	235 (-363–829)
Democratic People's Republic of Korea	Diarrheal diseases	718 (-598–1 850)	555 (-186–1 360)	103 (-65·6–336)	224 (-307–778)
Democratic People's Republic of Korea	Lower respiratory infections	24·1 (-100–112)	17·4 (-40·5–74·2)	1·52 (-0·381–4·36)	11·4 (-46·7–65·0)
Democratic People's Republic of Korea	Malaria	5·55 (-7·17–22·3)	5·55 (-7·17–22·3)	--	--
Democratic People's Republic of Korea	Measles	0 (0–0)	--	0 (0–0)	0 (0–0)
Taiwan	All causes	250 (-133–751)	146 (-31·4–415)	56·7 (-32·3–179)	64·8 (-68·6–246)
Taiwan	Diarrheal diseases	245 (-128–736)	144 (-29·8–408)	54·7 (-32·4–175)	63·3 (-65·9–237)
Taiwan	Lower respiratory infections	3·47 (-6·38–14·0)	1·83 (-2·45–7·25)	0·331 (-0·0686–0·85)	1·5 (-3·61–7·05)
Taiwan	Malaria	0 (0–0)	0 (0–0)	--	--
Taiwan	Measles	1·13 (-0·431–3·68)	--	1·11 (-0·422–3·57)	0·0217 (-0·00957–0·112)
Oceania	All causes	9 000 (4 830–14 300)	8 540 (5 020–13 000)	7 140 (4 580–10 300)	422 (-561–1 540)
Oceania	Diarrheal diseases	1 170 (-921–3 180)	777 (-220–2 030)	225 (-151–708)	381 (-489–1 360)
Oceania	Lower respiratory infections	38·8 (-114–166)	22·8 (-39–91·5)	5·1 (-1·1–14·0)	18·9 (-63·4–99·5)
Oceania	Malaria	865 (-691–2 360)	865 (-691–2 360)	--	--
Oceania	Measles	53·0 (-28·6–190)	--	34·0 (-13·9–117)	22·1 (-13·7–93·3)

American Samoa	All causes	0·639 (-0·0918–1·71)	0·425 (0·12–0·99)	0·307 (0·1–0·656)	0·132 (-0·148–0·499)
American Samoa	Diarrheal diseases	0·381 (-0·211–1·18)	0·223 (-0·0424–0·641)	0·0705 (-0·0406–0·234)	0·119 (-0·126–0·452)
American Samoa	Lower respiratory infections	0·0184 (-0·0344–0·0803)	0·00924 (-0·0121–0·0392)	0·00182 (>-0.001–0·00482)	0·00853 (-0·021–0·0445)
American Samoa	Malaria	0 (0–0)	0 (0–0)	--	--
American Samoa	Measles	0·0457 (-0·0182–0·157)	--	0·0415 (-0·0158–0·143)	0·00449 (-0·00197–0·0208)
Cook Islands	All causes	0·135 (-0·0742–0·421)	0·0704 (-0·0143–0·202)	0·0323 (-0·0177–0·101)	0·0402 (-0·0428–0·142)
Cook Islands	Diarrheal diseases	0·127 (-0·0677–0·394)	0·068 (-0·0123–0·195)	0·0281 (-0·0168–0·0894)	0·0383 (-0·0391–0·134)
Cook Islands	Lower respiratory infections	0·00364 (-0·00686–0·0177)	0·00167 (-0·00222–0·00757)	<0.001 (>-0.001–0·001)	0·00173 (-0·00444–0·00994)
Cook Islands	Malaria	0 (0–0)	0 (0–0)	--	--
Cook Islands	Measles	0·00332 (-0·00136–0·0121)	--	0·00311 (-0·00123–0·0111)	<0.001 (>-0.001–0·00117)
Fiji	All causes	59·7 (34·0–97·2)	54·5 (32·8–82·0)	50·6 (31·3–75·2)	3 (-3·32–11·2)
Fiji	Diarrheal diseases	11·2 (-5·88–32·7)	6·73 (-1·49–19·0)	2·59 (-1·63–8·68)	2·75 (-2·78–10·1)
Fiji	Lower respiratory infections	0·581 (-0·948–2·57)	0·312 (-0·394–1·32)	0·0803 (-0·018–0·215)	0·227 (-0·542–1·2)
Fiji	Malaria	0 (0–0)	0 (0–0)	--	--
Fiji	Measles	0·474 (-0·184–1·69)	--	0·453 (-0·174–1·6)	0·0227 (-0·00979–0·0943)
Guam	All causes	1·32 (-0·771–4·31)	0·686 (-0·155–2·06)	0·343 (-0·183–1·1)	0·384 (-0·429–1·5)
Guam	Diarrheal diseases	1·22 (-0·682–3·94)	0·65 (-0·135–1·96)	0·279 (-0·17–0·936)	0·359 (-0·38–1·4)
Guam	Lower respiratory infections	0·0451 (-0·0787–0·198)	0·0209 (-0·0266–0·0872)	0·00514 (-0·00109–0·0137)	0·021 (-0·0499–0·112)
Guam	Malaria	0 (0–0)	0 (0–0)	--	--
Guam	Measles	0·0466 (-0·0187–0·182)	--	0·0436 (-0·0168–0·17)	0·00323 (-0·00154–0·0178)
Kiribati	All causes	7·85 (3·24–13·7)	6·84 (3·82–10·8)	5·72 (3·42–8·7)	0·765 (-0·918–2·82)
Kiribati	Diarrheal diseases	2·42 (-1·49–6·92)	1·57 (-0·378–4·18)	0·439 (-0·28–1·44)	0·684 (-0·762–2·38)
Kiribati	Lower respiratory infections	0·149 (-0·294–0·667)	0·0854 (-0·112–0·366)	0·0126 (-0·00293–0·0353)	0·0663 (-0·174–0·361)
Kiribati	Malaria	0 (0–0)	0 (0–0)	--	--
Kiribati	Measles	0·0982 (-0·045–0·349)	--	0·0846 (-0·0354–0·285)	0·0149 (-0·00738–0·0597)
Marshall Islands	All causes	0·684 (-0·425–1·93)	0·496 (-0·149–1·33)	0·0371 (-0·0205–0·128)	0·204 (-0·263–0·753)
Marshall Islands	Diarrheal diseases	0·625 (-0·322–1·68)	0·462 (-0·105–1·2)	0·0315 (-0·0185–0·106)	0·181 (-0·216–0·624)
Marshall Islands	Lower respiratory infections	0·0518 (-0·107–0·239)	0·0343 (-0·0471–0·149)	<0.001 (>-0.001–0·00178)	0·0215 (-0·0559–0·12)
Marshall Islands	Malaria	0 (0–0)	0 (0–0)	--	--

Marshall Islands	Measles	0·00674 (-0·0029–0·0252)	--	0·00468 (-0·0019–0·0155)	0·00211 (-0·00103–0·00885)
Micronesia (Federated States of)	All causes	4·61 (2·42–7·49)	4·23 (2·39–6·45)	3·88 (2·15–5·9)	0·279 (-0·349–1·03)
Micronesia (Federated States of)	Diarrheal diseases	0·806 (-0·511–2·32)	0·506 (-0·124–1·36)	0·14 (-0·0908–0·427)	0·246 (-0·292–0·87)
Micronesia (Federated States of)	Lower respiratory infections	0·0577 (-0·129–0·259)	0·0313 (-0·0466–0·132)	0·00501 (-0·00129–0·0135)	0·0268 (-0·0764–0·145)
Micronesia (Federated States of)	Malaria	0 (0–0)	0 (0–0)	--	--
Micronesia (Federated States of)	Measles	0·0484 (-0·0196–0·167)	--	0·0423 (-0·0165–0·142)	0·00667 (-0·00323–0·0294)
Nauru	All causes	0·208 (-0·143–0·618)	0·124 (-0·0262–0·339)	0·028 (-0·00678–0·0789)	0·0786 (-0·102–0·295)
Nauru	Diarrheal diseases	0·183 (-0·112–0·549)	0·11 (-0·0242–0·307)	0·0197 (-0·0118–0·064)	0·0687 (-0·0749–0·25)
Nauru	Lower respiratory infections	0·0166 (-0·0364–0·0788)	0·00767 (-0·0102–0·0324)	<0.001 (>-0.001–0·00157)	0·00956 (-0·0253–0·0509)
Nauru	Malaria	0 (0–0)	0 (0–0)	--	--
Nauru	Measles	<0.001 (>-0.001–0·00334)	--	<0.001 (>-0.001–0·00203)	<0.001 (>-0.001–0·00153)
Niue	All causes	0·0142 (-0·00488–0·04)	0·0093 (<0.001–0·023)	0·00499 (<0.001–0·0109)	0·00343 (-0·00387–0·0132)
Niue	Diarrheal diseases	0·0108 (-0·00566–0·0325)	0·00629 (-0·00125–0·0181)	0·00214 (-0·00127–0·00688)	0·00313 (-0·00318–0·0117)
Niue	Lower respiratory infections	<0.001 (-0·00119–0·00295)	<0.001 (>-0.001–0·00142)	<0.001 (>-0.001–<0.001)	<0.001 (>-0.001–0·00157)
Niue	Malaria	0 (0–0)	0 (0–0)	--	--
Niue	Measles	<0.001 (>-0.001–<0.001)	--	<0.001 (>-0.001–<0.001)	<0.001 (>-0.001–<0.001)
Northern Mariana Islands	All causes	0·266 (-0·153–0·856)	0·141 (-0·0316–0·409)	0·0694 (-0·0348–0·226)	0·0759 (-0·0826–0·293)
Northern Mariana Islands	Diarrheal diseases	0·238 (-0·13–0·742)	0·131 (-0·0267–0·384)	0·0525 (-0·0315–0·173)	0·0701 (-0·0725–0·267)
Northern Mariana Islands	Lower respiratory infections	0·0104 (-0·0169–0·0522)	0·00494 (-0·00574–0·0233)	0·00111 (>-0.001–0·00308)	0·00489 (-0·0111–0·0289)
Northern Mariana Islands	Malaria	0 (0–0)	0 (0–0)	--	--
Northern Mariana Islands	Measles	0·0119 (-0·00469–0·0462)	--	0·0111 (-0·00421–0·042)	<0.001 (>-0.001–0·00466)
Palau	All causes	0·174 (-0·0653–0·483)	0·109 (0·00298–0·269)	0·0613 (>-0.001–0·144)	0·0406 (-0·0463–0·155)
Palau	Diarrheal diseases	0·139 (-0·0762–0·413)	0·0801 (-0·017–0·223)	0·0304 (-0·0187–0·0959)	0·0381 (-0·0411–0·143)
Palau	Lower respiratory infections	0·00486 (-0·00813–0·0217)	0·00243 (-0·00295–0·0102)	<0.001 (>-0.001–0·00131)	0·00221 (-0·00492–0·0119)
Palau	Malaria	0 (0–0)	0 (0–0)	--	--
Palau	Measles	0·00398 (-0·00163–0·0142)	--	0·00365 (-0·00144–0·0129)	<0.001 (>-0.001–0·0018)
Papua New Guinea	All causes	8 230 (4 410–13 000)	7 810 (4 580–11 900)	6 540 (4 190–9 480)	390 (-520–1 420)
Papua New Guinea	Diarrheal diseases	1 080 (-854–2 920)	717 (-204–1 870)	207 (-139–649)	353 (-455–1 250)
Papua New Guinea	Lower respiratory infections	33·9 (-102–145)	20·0 (-35–79·8)	4·5 (-0·975–12·4)	16·6 (-56·7–87·6)

Papua New Guinea	Malaria	777 (-635–2 120)	777 (-635–2 120)	--	--
Papua New Guinea	Measles	47·8 (-26·1–172)	--	30·3 (-12·4–104)	20·6 (-12·8–87·0)
Samoa	All causes	21·6 (13·0–33·0)	20·3 (12·8–30·9)	19·3 (12·2–28·8)	0·891 (-1·07–3·24)
Samoa	Diarrheal diseases	2·67 (-1·39–7·76)	1·5 (-0·288–4·16)	0·573 (-0·319–1·9)	0·814 (-0·828–2·79)
Samoa	Lower respiratory infections	0·169 (-0·302–0·742)	0·0802 (-0·107–0·324)	0·0229 (-0·00459–0·0611)	0·0756 (-0·186–0·417)
Samoa	Malaria	0 (0–0)	0 (0–0)	--	--
Samoa	Measles	0·0627 (-0·0213–0·227)	--	0·0606 (-0·0204–0·214)	0·00222 (-0·00103–0·00906)
Solomon Islands	All causes	261 (134–449)	255 (138–430)	199 (124–296)	4·76 (-6·29–18·0)
Solomon Islands	Diarrheal diseases	12·5 (-7·97–34·4)	7·96 (-1·94–20·9)	2·59 (-1·68–8)	3·66 (-4·13–12·9)
Solomon Islands	Lower respiratory infections	1·56 (-3·56–7·1)	0·878 (-1·31–3·79)	0·198 (-0·0415–0·539)	0·715 (-2·02–3·97)
Solomon Islands	Malaria	51·1 (-37·9–171)	51·1 (-37·9–171)	--	--
Solomon Islands	Measles	1·14 (-0·544–4·06)	--	0·799 (-0·313–2·72)	0·385 (-0·217–1·67)
Tokelau	All causes	0·0138 (>-0.001–0·0324)	0·0101 (0·00279–0·0203)	0·00668 (0·00242–0·0134)	0·00272 (-0·00334–0·0103)
Tokelau	Diarrheal diseases	0·00799 (-0·00446–0·0229)	0·00475 (>-0.001–0·0129)	0·00146 (>-0.001–0·00455)	0·00243 (-0·00279–0·00857)
Tokelau	Lower respiratory infections	<0.001 (-0·00102–0·00271)	<0.001 (>-0.001–0·00132)	<0.001 (>-0.001–<0.001)	<0.001 (>-0.001–0·00149)
Tokelau	Malaria	0 (0–0)	0 (0–0)	--	--
Tokelau	Measles	<0.001 (>-0.001–<0.001)	--	<0.001 (>-0.001–<0.001)	<0.001 (>-0.001–<0.001)
Tonga	All causes	6·04 (3·68–9·6)	5·85 (3·53–9·47)	5·76 (3·44–9·29)	0·107 (-0·117–0·401)
Tonga	Diarrheal diseases	0·338 (-0·184–1·04)	0·17 (-0·0353–0·484)	0·0894 (-0·055–0·285)	0·0921 (-0·0892–0·326)
Tonga	Lower respiratory infections	0·035 (-0·0486–0·149)	0·0155 (-0·0175–0·0662)	0·00557 (-0·00116–0·0149)	0·0149 (-0·0302–0·0771)
Tonga	Malaria	0 (0–0)	0 (0–0)	--	--
Tonga	Measles	0·00335 (-0·0012–0·0148)	--	0·00319 (-0·00113–0·0138)	<0.001 (>-0.001–<0.001)
Tuvalu	All causes	0·141 (-0·0758–0·409)	0·0819 (-0·0136–0·226)	0·0368 (-0·0103–0·11)	0·0423 (-0·047–0·161)
Tuvalu	Diarrheal diseases	0·121 (-0·068–0·362)	0·0691 (-0·0144–0·194)	0·0233 (-0·0139–0·0739)	0·0385 (-0·0383–0·143)
Tuvalu	Lower respiratory infections	0·00664 (-0·0127–0·0331)	0·00314 (-0·00417–0·0146)	<0.001 (>-0.001–0·0018)	0·00329 (-0·00825–0·019)
Tuvalu	Malaria	0 (0–0)	0 (0–0)	--	--
Tuvalu	Measles	0·00369 (-0·0013–0·0122)	--	0·00325 (-0·00111–0·0107)	<0.001 (>-0.001–0·00217)
Vanuatu	All causes	26·9 (2·84–53·9)	21·4 (8·52–36·6)	16·0 (7·43–26·0)	4·06 (-5·11–15·3)
Vanuatu	Diarrheal diseases	11·6 (-7·76–32·5)	7·42 (-1·84–19·5)	2·2 (-1·41–6·84)	3·57 (-4·19–13·0)

Vanuatu	Lower respiratory infections	0·554 (-1·3–2·51)	0·302 (-0·449–1·29)	0·0507 (-0·011–0·138)	0·27 (-0·766–1·49)
Vanuatu	Malaria	0·732 (-0·486–2·54)	0·732 (-0·486–2·54)	--	--
Vanuatu	Measles	0·956 (-0·423–3·27)	--	0·752 (-0·299–2·53)	0·228 (-0·116–1·02)
Southeast Asia	All causes	128 000 (77 000–195 000)	120 000 (76 400–173 000)	109 000 (69 200–156 000)	7 170 (-9 090–26 100)
Southeast Asia	Diarrheal diseases	22 600 (-15 700–64 000)	14 900 (-3 860–39 200)	4 670 (-2 990–14 500)	6 780 (-8 050–24 300)
Southeast Asia	Lower respiratory infections	682 (-1 630–2 880)	410 (-622–1 630)	80·9 (-18–207)	306 (-881–1 600)
Southeast Asia	Malaria	579 (-457–1 720)	579 (-457–1 720)	--	--
Southeast Asia	Measles	366 (-164–1 290)	--	294 (-119–1 010)	84·2 (-43·3–368)
Cambodia	All causes	2 660 (1 590–4 200)	2 530 (1 650–3 800)	2 310 (1 540–3 440)	118 (-172–449)
Cambodia	Diarrheal diseases	360 (-260–954)	244 (-67·2–613)	77·4 (-50·5–237)	105 (-129–370)
Cambodia	Lower respiratory infections	28·9 (-77·2–125)	18·2 (-29·9–75·3)	3·59 (-0·707–9·09)	12·9 (-40·2–72·3)
Cambodia	Malaria	40·2 (-42·7–181)	40·2 (-42·7–181)	--	--
Cambodia	Measles	0·00229 (>-0.001–0·00773)	--	0·00181 (>-0.001–0·00589)	<0.001 (>-0.001–0·00214)
Indonesia	All causes	77 700 (48 300–113 000)	73 700 (47 400–106 000)	68 400 (44 200–97 700)	3 660 (-4 720–13 400)
Indonesia	Diarrheal diseases	11 000 (-8 050–31 500)	7 250 (-1 900–19 300)	2 210 (-1 420–6 920)	3 480 (-4 240–12 600)
Indonesia	Lower respiratory infections	281 (-692–1 170)	168 (-256–672)	33·8 (-7·37–88·6)	130 (-393–673)
Indonesia	Malaria	262 (-204–821)	262 (-204–821)	--	--
Indonesia	Measles	170 (-76·7–593)	--	133 (-52·9–451)	43·3 (-21·9–186)
Lao People's Democratic Republic	All causes	1 030 (366–1 790)	908 (485–1 440)	754 (433–1 140)	98·2 (-126–361)
Lao People's Democratic Republic	Diarrheal diseases	318 (-228–872)	213 (-58·5–545)	70·2 (-47·5–218)	89·3 (-108–316)
Lao People's Democratic Republic	Lower respiratory infections	13·6 (-35·8–59·6)	8·5 (-13·8–34·4)	1·5 (-0·34–3·78)	6·16 (-18·7–32·7)
Lao People's Democratic Republic	Malaria	12·6 (-16·5–61·7)	12·6 (-16·5–61·7)	--	--
Lao People's Democratic Republic	Measles	10·0 (-4·76–33·8)	--	7·71 (-3·22–24·6)	2·71 (-1·45–11·5)
Malaysia	All causes	5 470 (3 320–8 020)	5 220 (3 270–7 470)	4 960 (3 080–7 030)	172 (-202–593)
Malaysia	Diarrheal diseases	642 (-364–1 780)	413 (-95·3–1 110)	149 (-89·8–474)	163 (-178–554)
Malaysia	Lower respiratory infections	20·6 (-39·9–85·3)	11·8 (-16·8–48·7)	3·14 (-0·761–8·2)	8·05 (-20·7–42·8)
Malaysia	Malaria	0·222 (-0·162–0·771)	0·222 (-0·162–0·771)	--	--
Malaysia	Measles	6·75 (-2·45–23·9)	--	5·85 (-1·99–20·5)	1·03 (-0·454–4·66)
Maldives	All causes	55·6 (22·6–95·2)	49·1 (27·6–77·0)	40·9 (25·0–62·1)	4·98 (-5·71–18·0)

Maldives	Diarrheal diseases	18·8 (-12–51·9)	12·4 (-3·18–32·1)	4·32 (-2·82–13·3)	4·88 (-5·4–17·5)
Maldives	Lower respiratory infections	0·271 (-0·603–1·17)	0·165 (-0·262–0·67)	0·0379 (-0·0091–0·104)	0·105 (-0·309–0·568)
Maldives	Malaria	0 (0–0)	0 (0–0)	--	--
Maldives	Measles	0·00101 (>-0.001–0·00345)	--	<0.001 (>-0.001–0·0029)	<0.001 (>-0.001–<0.001)
Mauritius	All causes	137 (77·5–211)	127 (77·0–186)	119 (72·3–176)	6·06 (-7·13–21·8)
Mauritius	Diarrheal diseases	24·5 (-15·5–64·9)	15·0 (-3·74–38·3)	6·99 (-4·73–20·9)	5·77 (-6·38–20·2)
Mauritius	Lower respiratory infections	0·728 (-1·39–3)	0·403 (-0·588–1·75)	0·144 (-0·0322–0·386)	0·267 (-0·748–1·49)
Mauritius	Malaria	0 (0–0)	0 (0–0)	--	--
Mauritius	Measles	0·319 (-0·125–1·08)	--	0·302 (-0·116–0·989)	0·0205 (-0·00885–0·0878)
Myanmar	All causes	5 590 (941–11 500)	4 700 (2 010–8 640)	3 380 (1 860–5 680)	713 (-859–2 610)
Myanmar	Diarrheal diseases	2 410 (-1 650–6 540)	1 610 (-435–4 130)	523 (-349–1 650)	666 (-759–2 320)
Myanmar	Lower respiratory infections	78·1 (-187–333)	48·3 (-73·8–201)	8·16 (-2·02–20·9)	34·6 (-102–189)
Myanmar	Malaria	247 (-234–774)	247 (-234–774)	--	--
Myanmar	Measles	60·3 (-28·9–216)	--	50·1 (-21·1–159)	11·9 (-5·88–52·8)
Philippines	All causes	18 200 (9 450–30 200)	16 800 (10 500–26 200)	14 900 (9 570–22 200)	1 230 (-1 540–4 380)
Philippines	Diarrheal diseases	3 970 (-2 690–11 100)	2 680 (-697–7 040)	807 (-513–2 500)	1 160 (-1 350–4 050)
Philippines	Lower respiratory infections	137 (-331–578)	84·3 (-130–339)	15·3 (-3·36–38·1)	60·2 (-175–318)
Philippines	Malaria	4·62 (-4·68–15·0)	4·62 (-4·68–15·0)	--	--
Philippines	Measles	59·6 (-26·4–211)	--	47·9 (-19·1–163)	13·5 (-6·93–64·5)
Seychelles	All causes	2·25 (-0·829–5·94)	1·53 (0·0326–3·59)	0·813 (0·0612–2·02)	0·5 (-0·612–1·91)
Seychelles	Diarrheal diseases	1·78 (-1·04–5·02)	1·11 (-0·254–3)	0·412 (-0·258–1·33)	0·465 (-0·511–1·72)
Seychelles	Lower respiratory infections	0·0788 (-0·189–0·365)	0·0435 (-0·0697–0·188)	0·00831 (-0·00179–0·022)	0·0345 (-0·108–0·194)
Seychelles	Malaria	0 (0–0)	0 (0–0)	--	--
Seychelles	Measles	0·0124 (-0·00524–0·0429)	--	0·0119 (-0·00503–0·0413)	<0.001 (>-0.001–0·00233)
Sri Lanka	All causes	5 660 (3 560–8 620)	5 470 (3 490–8 250)	5 240 (3 340–7 830)	152 (-202–530)
Sri Lanka	Diarrheal diseases	539 (-378–1 420)	363 (-97·7–908)	137 (-91·5–423)	145 (-180–497)
Sri Lanka	Lower respiratory infections	17·2 (-46·9–71·9)	10·9 (-18·6–44·6)	2·93 (-0·661–8·02)	6·65 (-22·6–35·7)
Sri Lanka	Malaria	0 (0–0)	0 (0–0)	--	--
Sri Lanka	Measles	0·145 (-0·061–0·389)	--	0·139 (-0·0568–0·37)	0·00763 (-0·00287–0·0282)

Thailand	All causes	2 840 (1 340–4 830)	2 480 (1 520–3 880)	2 140 (1 370–3 170)	256 (-296–876)
Thailand	Diarrheal diseases	857 (-516–2 400)	521 (-118–1 360)	188 (-119–572)	244 (-273–823)
Thailand	Lower respiratory infections	23·0 (-42–92·3)	12·0 (-15·5–48·0)	2·83 (-0·656–7·26)	10·3 (-25–51·7)
Thailand	Malaria	7·6 (-4·58–29·1)	7·6 (-4·58–29·1)	--	--
Thailand	Measles	11·2 (-4·52–38·0)	--	9·66 (-3·75–32·0)	1·7 (-0·847–7·84)
Timor-Leste	All causes	1 470 (945–2 100)	1 450 (955–2 060)	1 420 (928–2 020)	19·7 (-28·8–73·8)
Timor-Leste	Diarrheal diseases	65·8 (-50·7–173)	45·3 (-13·5–111)	17·2 (-12·4–52·5)	17·2 (-21·3–60·7)
Timor-Leste	Lower respiratory infections	6·17 (-16·6–24·9)	4·06 (-6·99–16·2)	1·14 (-0·233–2·95)	2·51 (-7·47–13·1)
Timor-Leste	Malaria	0·149 (-0·159–0·387)	0·149 (-0·159–0·387)	--	--
Timor-Leste	Measles	0·0272 (-0·013–0·0858)	--	0·0196 (-0·00795–0·058)	0·00975 (-0·00514–0·04)
Viet Nam	All causes	7 230 (2 740–12 700)	6 330 (3 600–10 400)	5 280 (3 170–8 190)	736 (-908–2 720)
Viet Nam	Diarrheal diseases	2 350 (-1 510–6 450)	1 520 (-373–3 930)	477 (-296–1 560)	692 (-813–2 440)
Viet Nam	Lower respiratory infections	75·6 (-160–310)	43·1 (-59·5–169)	8·25 (-1·6–19·9)	34·2 (-89·5–168)
Viet Nam	Malaria	4·22 (-2·88–14·9)	4·22 (-2·88–14·9)	--	--
Viet Nam	Measles	47·8 (-22·8–156)	--	39·1 (-16·6–123)	9·87 (-5·00–39·2)
**Sub-Saharan Africa**	**All causes**	**566 000 (167 000–1 060 000)**	**539 000 (186 000–982 000)**	**348 000 (223 000–494 000)**	**22 800 (-28 900–81 100)**
**Sub-Saharan Africa**	**Diarrheal diseases**	**70 300 (-50 600–193 000)**	**46 800 (-12 600–119 000)**	**15 000 (-10 000–45 200)**	**20 700 (-25 000–71 500)**
**Sub-Saharan Africa**	**Lower respiratory infections**	**2 970 (-7 320–12 200)**	**1 790 (-2 770–7 390)**	**375 (-82·2–966)**	**1 350 (-3 850–7 300)**
**Sub-Saharan Africa**	**Malaria**	**159 000 (-144 000–444 000)**	**159 000 (-144 000–444 000)**	**--**	**--**
**Sub-Saharan Africa**	**Measles**	**2 350 (-1 160–8 310)**	**--**	**1 740 (-685–5 840)**	**720 (-386–3 160)**
Central Sub-Saharan Africa	All causes	69 000 (3 700–153 000)	66 000 (8 440–143 000)	34 000 (21 800–50 300)	2 590 (-3 400–9 750)
Central Sub-Saharan Africa	Diarrheal diseases	7 110 (-5 330–18 900)	4 770 (-1 320–12 200)	1 330 (-888–4 130)	2 220 (-2 830–7 920)
Central Sub-Saharan Africa	Lower respiratory infections	361 (-886–1 540)	216 (-318–905)	40·2 (-8·77–107)	173 (-495–929)
Central Sub-Saharan Africa	Malaria	28 700 (-23 800–92 000)	28 700 (-23 800–92 000)	--	--
Central Sub-Saharan Africa	Measles	515 (-258–1 820)	--	352 (-142–1 160)	188 (-97·1–795)
Angola	All causes	9 860 (-2 160–22 500)	9 070 (-766–20 300)	3 430 (2 040–5 330)	751 (-1 000–2 850)
Angola	Diarrheal diseases	2 150 (-1 600–6 100)	1 460 (-398–3 980)	356 (-235–1 140)	698 (-880–2 600)
Angola	Lower respiratory infections	65·3 (-171–291)	39·3 (-61–163)	5·47 (-1·27–14·6)	32·1 (-98·2–173)
Angola	Malaria	4 550 (-3 380–14 200)	4 550 (-3 380–14 200)	--	--

Angola	Measles	58·9 (-26·4–198)	--	40·4 (-16·5–134)	20·9 (-11·4–98·6)
Central African Republic	All causes	6 590 (-162–14 400)	6 130 (723–13 100)	3 220 (2 050–4 780)	450 (-618–1 670)
Central African Republic	Diarrheal diseases	1 400 (-1 140–3 740)	949 (-291–2 450)	262 (-189–823)	433 (-570–1 580)
Central African Republic	Lower respiratory infections	32·8 (-88·4–136)	20·3 (-32·2–84·5)	3·48 (-0·676–8·92)	15·6 (-47·4–86·0)
Central African Republic	Malaria	2 200 (-2 150–7 220)	2 200 (-2 150–7 220)	--	--
Central African Republic	Measles	3·58 (-1·69–13·6)	--	2·41 (-1·05–8·67)	1·35 (-0·735–6·55)
Congo	All causes	1 600 (214–3 510)	1 510 (324–3 300)	770 (520–1 100)	65·7 (-80·6–248)
Congo	Diarrheal diseases	219 (-141–604)	138 (-33·8–368)	48·2 (-31·5–150)	62·2 (-72·7–230)
Congo	Lower respiratory infections	6·9 (-13·6–30·0)	3·77 (-5·02–15·6)	0·853 (-0·196–2·26)	3·11 (-8·07–16·4)
Congo	Malaria	649 (-370–2 130)	649 (-370–2 130)	--	--
Congo	Measles	1·99 (-0·969–7·34)	--	1·58 (-0·727–5·44)	0·465 (-0·265–2·1)
Democratic Republic of the Congo	All causes	50 600 (5 580–110 000)	49 000 (7 500–105 000)	26 500 (16 800–39 500)	1 290 (-1 730–4 880)
Democratic Republic of the Congo	Diarrheal diseases	3 270 (-2 390–8 450)	2 180 (-585–5 370)	654 (-428–2 020)	1 000 (-1 260–3 420)
Democratic Republic of the Congo	Lower respiratory infections	253 (-613–1 110)	151 (-219–646)	30·1 (-6·52–80·8)	120 (-358–665)
Democratic Republic of the Congo	Malaria	21 100 (-18 200–68 900)	21 100 (-18 200–68 900)	--	--
Democratic Republic of the Congo	Measles	444 (-220–1 560)	--	302 (-124–996)	164 (-83·9–687)
Equatorial Guinea	All causes	177 (-78·7–564)	161 (-54·2–509)	22·9 (7·56–44·2)	12·0 (-14·6–45·5)
Equatorial Guinea	Diarrheal diseases	27·4 (-18·8–81·2)	15·0 (-3·13–40·9)	5 (-3·08–15·7)	10·4 (-12·8–35·5)
Equatorial Guinea	Lower respiratory infections	1·16 (-3·07–5·47)	0·514 (-0·778–2·17)	0·121 (-0·0288–0·331)	0·648 (-2·17–3·55)
Equatorial Guinea	Malaria	130 (-67·6–450)	130 (-67·6–450)	--	--
Equatorial Guinea	Measles	3·2 (-1·44–12·3)	--	2·36 (-0·865–8·75)	0·914 (-0·452–4·38)
Gabon	All causes	179 (-86·4–616)	155 (-59·3–543)	30·2 (11·0–61·6)	17·6 (-19·6–68·9)
Gabon	Diarrheal diseases	47·8 (-29–139)	27·6 (-5·71–76·8)	8·59 (-5·12–28·6)	16·2 (-17·6–59·4)
Gabon	Lower respiratory infections	1·3 (-2·89–5·65)	0·621 (-0·894–2·55)	0·12 (-0·027–0·306)	0·671 (-1·9–3·4)
Gabon	Malaria	108 (-65·7–425)	108 (-65·7–425)	--	--
Gabon	Measles	3·03 (-1·28–11·0)	--	2·36 (-0·823–8·16)	0·719 (-0·336–3·24)
Eastern Sub-Saharan Africa	All causes	157 000 (52 500–278 000)	148 000 (65 000–253 000)	104 000 (66 100–149 000)	8 000 (-10 300–28 500)
Eastern Sub-Saharan Africa	Diarrheal diseases	23 400 (-16 700–65 300)	15 400 (-4 030–39 700)	4 620 (-3 010–14 000)	7 280 (-8 680–25 300)
Eastern Sub-Saharan Africa	Lower respiratory infections	1 040 (-2 600–4 410)	613 (-938–2 540)	116 (-26·1–298)	495 (-1 440–2 680)

Eastern Sub-Saharan Africa	Malaria	33 200 (-28 000–94 700)	33 200 (-28 000–94 700)	--	--
Eastern Sub-Saharan Africa	Measles	710 (-351–2 540)	--	520 (-207–1 760)	221 (-120–976)
Burundi	All causes	5 390 (-1 980–12 300)	5 120 (-1 270–11 500)	1 820 (1 120–2 730)	317 (-481–1 190)
Burundi	Diarrheal diseases	845 (-661–2 190)	606 (-174–1 510)	129 (-81·6–408)	279 (-373–989)
Burundi	Lower respiratory infections	56·8 (-169–253)	37·7 (-59·7–153)	4·12 (-0·922–11·0)	29·0 (-89·3–158)
Burundi	Malaria	2 800 (-2 990–8 120)	2 800 (-2 990–8 120)	--	--
Burundi	Measles	16·3 (-8·01–62·0)	--	9·28 (-3·36–32·0)	8·05 (-4·07–34·3)
Comoros	All causes	320 (191–482)	308 (190–452)	276 (178–396)	10·4 (-13·5–36·1)
Comoros	Diarrheal diseases	31·6 (-21·5–84·2)	20·1 (-4·94–51·1)	7·2 (-4·63–22·1)	9·52 (-11·4–32·0)
Comoros	Lower respiratory infections	1·68 (-4·00–6·84)	0·936 (-1·4–3·66)	0·262 (-0·057–0·668)	0·775 (-2·24–3·92)
Comoros	Malaria	17·8 (-17·5–92·9)	17·8 (-17·5–92·9)	--	--
Comoros	Measles	0·248 (-0·106–0·856)	--	0·181 (-0·0647–0·603)	0·0784 (-0·0408–0·329)
Djibouti	All causes	1 320 (822–1 980)	1 280 (813–1 900)	1 210 (779–1 740)	22·9 (-26·8–81·8)
Djibouti	Diarrheal diseases	93·1 (-62·3–243)	60·6 (-15·6–150)	29·0 (-19·8–85·2)	21·4 (-23–73·2)
Djibouti	Lower respiratory infections	3·1 (-6·48–11·7)	1·83 (-2·92–7·14)	0·809 (-0·207–2·17)	1·09 (-3·12–5·6)
Djibouti	Malaria	45·1 (-28·4–283)	45·1 (-28·4–283)	--	--
Djibouti	Measles	1·52 (-0·727–5·66)	--	1·24 (-0·52–4·35)	0·367 (-0·173–1·45)
Eritrea	All causes	3 580 (2 120–5 240)	3 430 (2 110–4 950)	3 160 (1 950–4 500)	127 (-174–454)
Eritrea	Diarrheal diseases	439 (-337–1 110)	304 (-88·8–722)	114 (-81·6–338)	114 (-142–387)
Eritrea	Lower respiratory infections	27·0 (-76·2–113)	18·0 (-32·1–75·6)	4·33 (-0·837–11·9)	11·0 (-35·4–61·5)
Eritrea	Malaria	68·7 (-64·7–208)	68·7 (-64·7–208)	--	--
Eritrea	Measles	6·24 (-3·00–22·5)	--	5·05 (-2·2–16·6)	1·48 (-0·775–6·54)
Ethiopia	All causes	41 100 (23 900–62 900)	38 800 (24 200–57 200)	34 000 (21 800–49 100)	1 850 (-2 350–6 440)
Ethiopia	Diarrheal diseases	5 660 (-3 950–15 900)	3 790 (-987–10 100)	1 250 (-812–4 020)	1 650 (-1 970–5 660)
Ethiopia	Lower respiratory infections	259 (-663–1 090)	158 (-254–644)	36·9 (-8·16–95·4)	115 (-344–619)
Ethiopia	Malaria	2 340 (-1 860–8 210)	2 340 (-1 860–8 210)	--	--
Ethiopia	Measles	260 (-129–899)	--	192 (-81·5–637)	80·9 (-46·3–348)
Kenya	All causes	7 280 (541–14 800)	6 260 (1 920–11 800)	3 950 (2 440–5 800)	905 (-1 160–3 280)
Kenya	Diarrheal diseases	2 730 (-1 880–7 920)	1 800 (-467–4 830)	521 (-334–1 640)	838 (-985–2 960)

Kenya	Lower respiratory infections	125 (-295–543)	73·6 (-112–298)	12·3 (-2·73–30·8)	58·4 (-160–308)
Kenya	Malaria	1 000 (-843–3 270)	1 000 (-843–3 270)	--	--
Kenya	Measles	36·4 (-15·9–122)	--	28·7 (-11·5–96·4)	8·71 (-3·74–40·0)
Madagascar	All causes	17 100 (6 630–29 100)	16 000 (7 960–26 000)	12 400 (7 900–18 100)	1 080 (-1 380–3 870)
Madagascar	Diarrheal diseases	3 320 (-2 520–8 870)	2 310 (-673–5 840)	610 (-410–1 870)	1 020 (-1 250–3 620)
Madagascar	Lower respiratory infections	88·3 (-255–369)	56·9 (-98·2–232)	8·75 (-1·91–22·7)	40·9 (-129–218)
Madagascar	Malaria	1 930 (-2 160–6 140)	1 930 (-2 160–6 140)	--	--
Madagascar	Measles	59·3 (-31·2–219)	--	40·9 (-17·6–133)	21·4 (-13·1–92·6)
Malawi	All causes	4 650 (-1 420–13 500)	4 160 (-687–12 100)	1 310 (771–2 020)	468 (-601–1 630)
Malawi	Diarrheal diseases	1 190 (-831–3 240)	738 (-178–1 880)	161 (-99–504)	439 (-526–1 510)
Malawi	Lower respiratory infections	33·7 (-95·6–157)	17·6 (-28·5–76·8)	2·03 (-0·471–5·63)	18·6 (-60·7–101)
Malawi	Malaria	2 270 (-1 610–8 730)	2 270 (-1 610–8 730)	--	--
Malawi	Measles	24·2 (-11·3–90·8)	--	15·2 (-6·21–57·0)	9·66 (-5·26–41·9)
Mozambique	All causes	9 930 (-3 720–24 900)	9 520 (-3 140–23 900)	2 240 (1 360–3 330)	379 (-510–1 430)
Mozambique	Diarrheal diseases	997 (-709–2 750)	632 (-154–1 680)	164 (-102–519)	345 (-417–1 250)
Mozambique	Lower respiratory infections	52·5 (-138–223)	28·4 (-41·9–109)	4·5 (-0·986–11·4)	27·6 (-88·9–138)
Mozambique	Malaria	6 800 (-5 010–19 800)	6 800 (-5 010–19 800)	--	--
Mozambique	Measles	17·9 (-9·03–64·5)	--	11·9 (-4·85–39·1)	6·59 (-3·63–30·5)
Rwanda	All causes	867 (-432–2 280)	651 (-87·7–1 630)	234 (96·7–430)	222 (-303–805)
Rwanda	Diarrheal diseases	520 (-379–1 460)	324 (-79·3–846)	61·3 (-37·2–190)	203 (-251–716)
Rwanda	Lower respiratory infections	24·9 (-69·7–116)	12·7 (-19·2–52·1)	1·26 (-0·274–3·29)	14·5 (-46·9–77·5)
Rwanda	Malaria	147 (-120–593)	147 (-120–593)	--	--
Rwanda	Measles	8·87 (-4·37–36·6)	--	4·67 (-2·02–18·0)	4·5 (-2·71–21·4)
Somalia	All causes	21 800 (13 800–31 600)	21 000 (13 300–30 000)	19 800 (12 800–28 100)	522 (-631–1 940)
Somalia	Diarrheal diseases	1 650 (-1 140–4 660)	1 050 (-259–2 850)	469 (-312–1 460)	451 (-503–1 620)
Somalia	Lower respiratory infections	83·5 (-192–336)	51·2 (-77·5–211)	13·7 (-3·02–35·2)	33·7 (-96·2–179)
Somalia	Malaria	689 (-497–2 090)	689 (-497–2 090)	--	--
Somalia	Measles	167 (-83·8–557)	--	137 (-57–444)	37·6 (-19·9–162)
South Sudan	All causes	17 500 (10 300–26 000)	17 100 (10 300–25 100)	14 800 (9 730–20 700)	269 (-326–993)

South Sudan	Diarrheal diseases	1 080<br>(-785–2 900)	715<br>(-205–1 840)	316<br>(-232–986)	257<br>(-294–930)
South Sudan	Lower respiratory infections	30·2<br>(-74·1–120)	18·2<br>(-31·9–73·6)	7·64<br>(-1·98–19·7)	10·8<br>(-32·6–57·1)
South Sudan	Malaria	1 880<br>(-1 560–5 860)	1 880<br>(-1 560–5 860)	--	--
South Sudan	Measles	5·92<br>(-3·05–21·2)	--	4·81<br>(-2·23–16·6)	1·45<br>(-0·838–6·97)
Uganda	All causes	12 500<br>(-2 240–32 500)	11 800<br>(-1 170–30 600)	4 200<br>(2 630–6 020)	646<br>(-844–2 330)
Uganda	Diarrheal diseases	1 800<br>(-1 250–4 890)	1 140<br>(-281–2 900)	302<br>(-195–950)	593<br>(-693–2 060)
Uganda	Lower respiratory infections	86·9<br>(-209–385)	47·1<br>(-67·8–197)	6·99<br>(-1·58–18·1)	44·3<br>(-127–234)
Uganda	Malaria	6 730<br>(-4 900–22 800)	6 730<br>(-4 900–22 800)	--	--
Uganda	Measles	27·6<br>(-11·7–103)	--	19·7<br>(-7·19–69·9)	8·71<br>(-4·18–38·7)
United Republic of Tanzania	All causes	9 340<br>(-4 120–24 000)	8 410<br>(-2 660–21 100)	2 820<br>(1 630–4 280)	864<br>(-1 140–3 080)
United Republic of Tanzania	Diarrheal diseases	2 220<br>(-1 490–6 000)	1 420<br>(-340–3 540)	347<br>(-214–1 040)	764<br>(-919–2 600)
United Republic of Tanzania	Lower respiratory infections	141<br>(-351–662)	76·9<br>(-111–331)	10·2<br>(-2·14–28·5)	74·5<br>(-217–408)
United Republic of Tanzania	Malaria	4 490<br>(-4 220–14 400)	4 490<br>(-4 220–14 400)	--	--
United Republic of Tanzania	Measles	64·0<br>(-30·6–238)	--	40·9<br>(-16·2–145)	25·4<br>(-14·2–112)
Zambia	All causes	4 180<br>(-563–10 500)	3 830<br>(49·6–9 350)	1 500<br>(913–2 300)	313<br>(-402–1 150)
Zambia	Diarrheal diseases	823<br>(-604–2 280)	505<br>(-120–1 330)	139<br>(-89·7–425)	293<br>(-350–1 040)
Zambia	Lower respiratory infections	27·0<br>(-70·4–120)	13·7<br>(-20·4–56·5)	2·52<br>(-0·617–6·81)	14·7<br>(-45·4–75·4)
Zambia	Malaria	1 960<br>(-1 350–6 450)	1 960<br>(-1 350–6 450)	--	--
Zambia	Measles	14·1<br>(-5·67–50·1)	--	8·5<br>(-2·76–27·8)	6·14<br>(-2·76–26·6)
Southern Sub-Saharan Africa	All causes	5 370<br>(1 150–11 100)	4 350<br>(2 190–7 690)	3 180<br>(2 000–4 600)	962<br>(-1 220–3 530)
Southern Sub-Saharan Africa	Diarrheal diseases	2 270<br>(-1 580–7 020)	1 340<br>(-311–3 840)	305<br>(-185–1 030)	891<br>(-1 040–3 200)
Southern Sub-Saharan Africa	Lower respiratory infections	103<br>(-242–473)	50·1<br>(-68–212)	7·01<br>(-1·56–18·6)	58·2<br>(-161–308)
Southern Sub-Saharan Africa	Malaria	119<br>(-67·6–449)	119<br>(-67·6–449)	--	--
Southern Sub-Saharan Africa	Measles	33·6<br>(-15·1–122)	--	22·2<br>(-8·6–80·2)	12·3<br>(-6·31–56·0)
Botswana	All causes	263<br>(149–407)	243<br>(152–362)	227<br>(143–334)	15·7<br>(-20–56·9)
Botswana	Diarrheal diseases	41·1<br>(-28·8–118)	24·1<br>(-5·76–65·5)	8·72<br>(-5·57–26·9)	13·9<br>(-16·1–49·9)
Botswana	Lower respiratory infections	2·44<br>(-5·58–10·3)	1·19<br>(-1·7–4·76)	0·341<br>(-0·0731–0·913)	1·23<br>(-3·5–6·13)
Botswana	Malaria	0·932<br>(-0·529–2·81)	0·932<br>(-0·529–2·81)	--	--
Botswana	Measles	1·79<br>(-0·876–6·28)	--	1·26<br>(-0·509–4·37)	0·597<br>(-0·306–2·45)

Eswatini	All causes	11·3 (-9·01–35·0)	6·27 (-1·86–17·4)	1·41 (-0·615–4·18)	4·81 (-6·56–18·6)
Eswatini	Diarrheal diseases	9·37 (-6·2–26·7)	5·32 (-1·12–14·2)	0·964 (-0·563–3·07)	3·99 (-4·79–13·8)
Eswatini	Lower respiratory infections	1·03 (-2·39–4·56)	0·458 (-0·577–1·86)	0·0484 (-0·0109–0·119)	0·624 (-1·73–3·14)
Eswatini	Malaria	0·304 (-0·152–0·88)	0·304 (-0·152–0·88)	--	--
Eswatini	Measles	0·411 (-0·174–1·49)	--	0·22 (-0·0773–0·716)	0·199 (-0·0968–0·741)
Lesotho	All causes	96·9 (-14·6–223)	72·5 (21·4–143)	42·7 (22·4–68·2)	24·8 (-34·8–92·3)
Lesotho	Diarrheal diseases	56·1 (-40·7–153)	35·3 (-8·53–89·8)	7·15 (-4·37–21·8)	21·4 (-26·3–73·6)
Lesotho	Lower respiratory infections	5·15 (-14·1–23·1)	2·7 (-3·94–11·9)	0·316 (-0·0642–0·838)	2·89 (-9·04–15·6)
Lesotho	Malaria	0 (0–0)	0 (0–0)	--	--
Lesotho	Measles	1·21 (-0·525–4·39)	--	0·718 (-0·262–2·62)	0·53 (-0·264–2·3)
Namibia	All causes	383 (228–582)	366 (219–542)	342 (205–511)	12·7 (-16·6–46·4)
Namibia	Diarrheal diseases	39·1 (-25·4–107)	24·8 (-6·14–63·1)	9·09 (-5·93–27·8)	10·7 (-12–37·2)
Namibia	Lower respiratory infections	3·67 (-7·55–16·5)	2·07 (-2·9–9·21)	0·484 (-0·101–1·32)	1·6 (-4·21–9·04)
Namibia	Malaria	7·76 (-5·23–36·6)	7·76 (-5·23–36·6)	--	--
Namibia	Measles	1·47 (-0·649–5·43)	--	1·2 (-0·471–4·32)	0·307 (-0·133–1·44)
South Africa	All causes	3 280 (468–7 480)	2 530 (1 220–4 750)	1 790 (1 120–2 710)	714 (-896–2 630)
South Africa	Diarrheal diseases	1 630 (-1 140–5 200)	933 (-215–2 740)	206 (-123–703)	670 (-790–2 440)
South Africa	Lower respiratory infections	60·5 (-144–284)	27·4 (-36·6–119)	3·67 (-0·828–9·77)	35·8 (-99·5–192)
South Africa	Malaria	5·33 (-2·6–16·9)	5·33 (-2·6–16·9)	--	--
South Africa	Measles	21·0 (-9·41–78·4)	--	13·3 (-5·15–48·5)	8·13 (-3·93–39·2)
Zimbabwe	All causes	1 340 (269–2 680)	1 140 (498–2 030)	780 (473–1 140)	190 (-252–688)
Zimbabwe	Diarrheal diseases	494 (-336–1 420)	315 (-74·8–874)	73·1 (-45·8–242)	172 (-204–590)
Zimbabwe	Lower respiratory infections	30·4 (-73·1–135)	16·3 (-22·9–66·5)	2·15 (-0·482–5·73)	16·0 (-47·7–83·3)
Zimbabwe	Malaria	105 (-63·7–428)	105 (-63·7–428)	--	--
Zimbabwe	Measles	7·81 (-3·48–26·5)	--	5·53 (-2·09–18·3)	2·5 (-1·21–10·3)
Western Sub-Saharan Africa	All causes	335 000 (92 900–618 000)	321 000 (108 000–577 000)	207 000 (132 000–295 000)	11 200 (-14 200–39 600)
Western Sub-Saharan Africa	Diarrheal diseases	37 500 (-27 000–101 000)	25 300 (-6 980–64 000)	8 720 (-5 960–26 100)	10 300 (-12 300–35 300)
Western Sub-Saharan Africa	Lower respiratory infections	1 460 (-3 660–5 900)	914 (-1 460–3 710)	211 (-45·8–542)	626 (-1 780–3 300)
Western Sub-Saharan Africa	Malaria	97 100 (-89 900–273 000)	97 100 (-89 900–273 000)	--	--

Western Sub-Saharan Africa	Measles	1 090 (-537–3 830)	--	845 (-344–2 840)	298 (-164–1 330)
Benin	All causes	7 110 (-1 000–15 300)	6 850 (-508–14 700)	2 950 (1 870–4 220)	239 (-319–873)
Benin	Diarrheal diseases	688 (-496–1 860)	455 (-119–1 160)	126 (-81·1–404)	218 (-278–771)
Benin	Lower respiratory infections	31·1 (-80·3–136)	18·2 (-27·8–74·3)	3·06 (-0·629–7·97)	15·0 (-46·5–78·5)
Benin	Malaria	3 570 (-3 150–10 300)	3 570 (-3 150–10 300)	--	--
Benin	Measles	18·6 (-7·93–75·7)	--	13·9 (-5·64–53·3)	5·35 (-3·03–26·0)
Burkina Faso	All causes	21 200 (7 410–37 400)	20 800 (7 710–36 200)	14 100 (9 050–20 600)	345 (-456–1 300)
Burkina Faso	Diarrheal diseases	1 210 (-892–3 340)	816 (-234–2 140)	288 (-206–900)	316 (-396–1 160)
Burkina Faso	Lower respiratory infections	50·3 (-112–206)	31·4 (-47·7–123)	7·14 (-1·7–18·5)	20·3 (-60·6–104)
Burkina Faso	Malaria	6 150 (-5 700–17 300)	6 150 (-5 700–17 300)	--	--
Burkina Faso	Measles	44·6 (-21·1–152)	--	37·2 (-15·5–124)	8·93 (-4·95–41·0)
Cabo Verde	All causes	6·19 (-4·00–17·4)	4·06 (-1·25–10·9)	1·33 (-0·717–3·89)	1·54 (-1·95–5·74)
Cabo Verde	Diarrheal diseases	5·27 (-3·27–14·9)	3·31 (-0·787–8·88)	1·16 (-0·713–3·48)	1·45 (-1·72–5·32)
Cabo Verde	Lower respiratory infections	0·201 (-0·4–0·851)	0·111 (-0·149–0·437)	0·0212 (-0·0039–0·0534)	0·0903 (-0·238–0·457)
Cabo Verde	Malaria	0·56 (-0·371–1·53)	0·56 (-0·371–1·53)	--	--
Cabo Verde	Measles	0·0798 (-0·0328–0·278)	--	0·0752 (-0·0297–0·261)	0·00515 (-0·00249–0·0235)
Cameroon	All causes	9 600 (-2 170–24 800)	8 700 (-876–21 800)	3 240 (1 940–4 930)	766 (-928–2 710)
Cameroon	Diarrheal diseases	2 150 (-1 450–5 900)	1 340 (-320–3 450)	383 (-235–1 180)	718 (-845–2 520)
Cameroon	Lower respiratory infections	51·0 (-118–222)	27·3 (-37·9–112)	4·87 (-0·981–13·2)	25·6 (-72–132)
Cameroon	Malaria	4 520 (-3 030–15 300)	4 520 (-3 030–15 300)	--	--
Cameroon	Measles	67·7 (-30·1–254)	--	47·8 (-18·6–170)	22·0 (-12–98·8)
Chad	All causes	25 300 (11 100–42 000)	23 800 (12 400–37 900)	18 900 (12 000–27 300)	1 160 (-1 540–4 090)
Chad	Diarrheal diseases	4 230 (-3 430–10 900)	2 840 (-929–7 050)	1 070 (-847–3 230)	1 110 (-1 430–3 860)
Chad	Lower respiratory infections	95·9 (-227–388)	59·9 (-96·1–238)	17·0 (-3·56–43·9)	37·4 (-112–195)
Chad	Malaria	3 150 (-2 920–9 370)	3 150 (-2 920–9 370)	--	--
Chad	Measles	72·9 (-34·6–244)	--	58·8 (-23·7–191)	17·5 (-9·71–77·6)
Côte d'Ivoire	All causes	11 000 (-2 030–28 300)	10 500 (-1 140–26 700)	3 750 (2 320–5 750)	349 (-447–1 270)
Côte d'Ivoire	Diarrheal diseases	1 150 (-759–3 160)	742 (-187–1 940)	251 (-163–786)	322 (-378–1 150)
Côte d'Ivoire	Lower respiratory infections	47·1 (-99·2–196)	26·8 (-37·8–110)	5·62 (-1·37–14·3)	20·8 (-56·3–109)

Côte d'Ivoire	Malaria	6 290 (-4 530–20 800)	6 290 (-4 530–20 800)	--	--
Côte d'Ivoire	Measles	28·2 (-11·7–94·1)	--	23·2 (-9·08–76·1)	5·69 (-2·93–26·7)
Gambia	All causes	565 (286–929)	538 (295–865)	438 (271–648)	18·6 (-24·4–67·9)
Gambia	Diarrheal diseases	63·2 (-40·5–165)	42·0 (-10·7–105)	14·4 (-9·44–43·1)	16·3 (-19·3–55·9)
Gambia	Lower respiratory infections	4·22 (-9·63–18·6)	2·58 (-4·06–10·9)	0·495 (-0·102–1·3)	1·73 (-4·91–9·49)
Gambia	Malaria	74·6 (-45·2–284)	74·6 (-45·2–284)	--	--
Gambia	Measles	4 (-1·73–13·8)	--	3·53 (-1·49–12·1)	0·544 (-0·292–2·52)
Ghana	All causes	6 530 (1 220–12 900)	6 280 (1 510–12 100)	3 650 (2 330–5 280)	160 (-201–586)
Ghana	Diarrheal diseases	555 (-324–1 660)	356 (-80·2–1 010)	133 (-84·1–438)	142 (-163–496)
Ghana	Lower respiratory infections	34·2 (-68·8–135)	19·8 (-28·2–78·6)	4·44 (-0·998–11·2)	14·2 (-38–70·3)
Ghana	Malaria	2 420 (-2 190–7 410)	2 420 (-2 190–7 410)	--	--
Ghana	Measles	30·8 (-13–101)	--	26·8 (-10·5–85·8)	4·57 (-2·15–21·6)
Guinea	All causes	7 260 (1 130–14 300)	6 990 (1 450–13 500)	4 160 (2 580–6 210)	195 (-239–739)
Guinea	Diarrheal diseases	567 (-390–1 510)	373 (-90·6–941)	125 (-85·3–383)	161 (-184–565)
Guinea	Lower respiratory infections	40·1 (-86·4–179)	23·4 (-33–106)	5·16 (-1·12–14·7)	17·9 (-48·7–103)
Guinea	Malaria	2 610 (-2 820–8 050)	2 610 (-2 820–8 050)	--	--
Guinea	Measles	58·4 (-27·7–203)	--	44·1 (-18·7–146)	16·7 (-9·5–69·3)
Guinea-Bissau	All causes	505 (139–1 010)	471 (179–910)	314 (183–463)	28·5 (-36·2–101)
Guinea-Bissau	Diarrheal diseases	79·4 (-54·5–207)	53·0 (-13·4–131)	14·2 (-9·09–43·3)	24·9 (-29·5–83·4)
Guinea-Bissau	Lower respiratory infections	4·44 (-11·7–18·3)	2·65 (-4·19–9·97)	0·413 (-0·0895–1·06)	2·1 (-6·66–10·1)
Guinea-Bissau	Malaria	121 (-116–481)	121 (-116–481)	--	--
Guinea-Bissau	Measles	5·48 (-2·42–19·6)	--	4·17 (-1·63–14·8)	1·49 (-0·794–6·76)
Liberia	All causes	1 770 (-566–4 720)	1 690 (-451–4 470)	431 (258–640)	65·4 (-86·6–234)
Liberia	Diarrheal diseases	186 (-124–501)	121 (-30–305)	32·9 (-20·8–99·7)	57·7 (-70·4–199)
Liberia	Lower respiratory infections	9·94 (-22·6–40·6)	5·6 (-7·95–22·6)	0·842 (-0·168–2·13)	4·84 (-13·3–24·9)
Liberia	Malaria	1 180 (-832–3 820)	1 180 (-832–3 820)	--	--
Liberia	Measles	11·0 (-4·65–39·7)	--	8·43 (-3·19–28·9)	2·88 (-1·46–13·1)
Mali	All causes	13 600 (2 370–26 900)	12 800 (3 340–24 800)	7 380 (4 760–10 700)	569 (-658–2 050)
Mali	Diarrheal diseases	2 110 (-1 400–5 560)	1 400 (-367–3 560)	528 (-354–1 630)	529 (-598–1 890)

Mali	Lower respiratory infections	57·8 (-114–239)	35·5 (-49·4–147)	8·34 (-1·78–22·5)	22·9 (-57·8–119)
Mali	Malaria	4 580 (-4 410–14 000)	4 580 (-4 410–14 000)	--	--
Mali	Measles	93·4 (-41·1–341)	--	79·0 (-30·3–280)	17·4 (-8·89–75·2)
Mauritania	All causes	1 890 (1 110–2 870)	1 800 (1 110–2 660)	1 600 (1 060–2 380)	65·4 (-80·4–243)
Mauritania	Diarrheal diseases	262 (-170–714)	173 (-45·7–453)	68·3 (-46·5–214)	61·2 (-70·4–221)
Mauritania	Lower respiratory infections	8·39 (-19·1–35·7)	5·19 (-8·4–21·4)	1·19 (-0·253–3·13)	3·23 (-9·05–17·4)
Mauritania	Malaria	87·0 (-70·7–343)	87·0 (-70·7–343)	--	--
Mauritania	Measles	6 (-2·7–20·5)	--	5·17 (-2·25–16·3)	0·993 (-0·486–4·73)
Niger	All causes	29 200 (9 800–49 000)	28 200 (11 500–46 400)	20 800 (13 700–29 500)	911 (-1 240–3 180)
Niger	Diarrheal diseases	3 150 (-2 360–7 770)	2 270 (-691–5 360)	738 (-527–2 130)	833 (-1 060–2 800)
Niger	Lower respiratory infections	140 (-410–590)	98·0 (-180–383)	18·8 (-4·05–48·6)	59·2 (-185–306)
Niger	Malaria	5 860 (-7 950–17 400)	5 860 (-7 950–17 400)	--	--
Niger	Measles	55·1 (-27·4–187)	--	40·2 (-17·8–128)	18·4 (-10·7–77·9)
Nigeria	All causes	191 000 (56 600–354 000)	184 000 (64 800–335 000)	121 000 (76 800–176 000)	5 840 (-7 540–20 600)
Nigeria	Diarrheal diseases	19 300 (-13 700–51 300)	13 100 (-3 550–33 600)	4 550 (-3 040–14 200)	5 310 (-6 270–18 000)
Nigeria	Lower respiratory infections	832 (-2 160–3 340)	524 (-858–2 100)	127 (-27·4–324)	357 (-1 050–1 860)
Nigeria	Malaria	53 600 (-50 400–151 000)	53 600 (-50 400–151 000)	--	--
Nigeria	Measles	562 (-292–1 950)	--	422 (-173–1 410)	170 (-95·2–762)
Sao Tome and Principe	All causes	12·5 (6·04–20·9)	11·4 (6·29–17·3)	9·82 (5·72–14·0)	0·707 (-0·901–2·66)
Sao Tome and Principe	Diarrheal diseases	1·94 (-1·21–5·5)	1·19 (-0·273–3·1)	0·384 (-0·235–1·2)	0·601 (-0·688–2·09)
Sao Tome and Principe	Lower respiratory infections	0·148 (-0·316–0·67)	0·0766 (-0·109–0·326)	0·016 (-0·00319–0·0426)	0·0706 (-0·192–0·384)
Sao Tome and Principe	Malaria	0·908 (-0·462–2·89)	0·908 (-0·462–2·89)	--	--
Sao Tome and Principe	Measles	0·202 (-0·0927–0·748)	--	0·17 (-0·0716–0·62)	0·0359 (-0·0196–0·177)
Senegal	All causes	2 610 (801–4 910)	2 340 (1 160–4 240)	1 770 (1 070–2 770)	172 (-196–596)
Senegal	Diarrheal diseases	660 (-399–1 730)	426 (-102–1 060)	167 (-110–485)	158 (-170–525)
Senegal	Lower respiratory infections	25·6 (-47·9–108)	15·1 (-20·7–61·2)	3·58 (-0·66–10·2)	9·96 (-24·8–51·3)
Senegal	Malaria	321 (-258–1 390)	321 (-258–1 390)	--	--
Senegal	Measles	26·8 (-11·8–93·3)	--	23·8 (-9·73–82·6)	3·53 (-1·71–15·2)
Sierra Leone	All causes	3 370 (35·4–8 170)	3 170 (288–7 700)	1 500 (917–2 170)	168 (-201–607)

Sierra Leone	Diarrheal diseases	534 (-357–1 460)	342 (-85·6–888)	108 (-70·5–329)	159 (-183–568)
Sierra Leone	Lower respiratory infections	15·6 (-33·7–70·6)	8·73 (-12–38·6)	1·89 (-0·424–5·28)	7·24 (-19·3–40·5)
Sierra Leone	Malaria	1 430 (-1 060–5 150)	1 430 (-1 060–5 150)	--	--
Sierra Leone	Measles	3·43 (-1·36–13·3)	--	2·57 (-1·03–9·41)	0·982 (-0·49–4·45)
Togo	All causes	2 340 (-1 090–5 790)	2 130 (-741–5 280)	701 (382–1 120)	170 (-213–615)
Togo	Diarrheal diseases	591 (-393–1 600)	392 (-101–1 010)	126 (-82·4–380)	163 (-193–579)
Togo	Lower respiratory infections	14·9 (-35·2–68·1)	8·97 (-13·7–38·3)	1·69 (-0·367–4·61)	6·46 (-19·4–35·3)
Togo	Malaria	1 160 (-1 170–3 740)	1 160 (-1 170–3 740)	--	--
Togo	Measles	4·56 (-1·84–16·0)	--	3·88 (-1·51–13·5)	0·789 (-0·371–3·74)

Table S6. All-cause and cause-specific population attributable fraction (PAF, %) of YLDs among children under 5 years for child growth failure at the global and super-regional, regional, and national levels, 2023 Estimates combine burden associated with mild, moderate, and severe forms of CGF: stunting was defined as height-for-age z-score (HAZ) < –1; underweight as weight-for-age z-score (WAZ) < –1; wasting as weight-for-height z-score (WHZ) < –1, according to WHO Child Growth Standards.

Location	Cause name	Child growth failure	Child underweight	Child wasting	Child stunting
**Global**	**All causes**	**5·5 (3·2–7·9)**	**5·3 (3·6–7·3)**	**4·4 (3·5–5·5)**	**0·21 (-0·25–0·71)**
**Global**	**Diarrheal diseases**	**24·7 (-18·1–56·9)**	**16·5 (-4·7–36·8)**	**5·6 (-3·9–15·6)**	**7·1 (-8·4–22·5)**
**Global**	**Lower respiratory infections**	**13·4 (-32·7–50·4)**	**8·1 (-12·9–29·7)**	**2·0 (-0·38–4·5)**	**5·8 (-16·7–28·0)**
**Global**	**Malaria**	**27·7 (-24·4–70·7)**	**27·7 (-24·4–70·7)**	**--**	**--**
**Global**	**Measles**	**11·7 (-4·9–30·3)**	**--**	**8·9 (-3·3–22·3)**	**3·4 (-1·7–11·1)**
**Central Europe, Eastern Europe, and Central Asia**	**All causes**	**1·3 (0·86–1·8)**	**1·2 (0·87–1·5)**	**1·1 (0·83–1·5)**	**<0.1 (>-0.1–0·26)**
**Central Europe, Eastern Europe, and Central Asia**	**Diarrheal diseases**	**12·1 (-7·8–32·2)**	**6·2 (-1·4–15·3)**	**2·8 (-1·8–7·9)**	**4·2 (-4·5–13·6)**
**Central Europe, Eastern Europe, and Central Asia**	**Lower respiratory infections**	**6·5 (-12·0–26·7)**	**2·7 (-3·3–10·5)**	**0·89 (-0·17–2·1)**	**3·4 (-8·2–16·6)**
**Central Europe, Eastern Europe, and Central Asia**	**Malaria**	**0 (0–0)**	**0 (0–0)**	**--**	**--**
**Central Europe, Eastern Europe, and Central Asia**	**Measles**	**4·6 (-1·7–12·6)**	**--**	**3·7 (-1·2–9·8)**	**1·0 (-0·43–3·5)**
Central Asia	All causes	1·3 (0·87–1·9)	1·2 (0·88–1·6)	1·1 (0·82–1·5)	<0.1 (>-0.1–0·26)
Central Asia	Diarrheal diseases	15·8 (-10·3–40·4)	8·9 (-2·1–21·4)	3·4 (-2·2–9·6)	5·2 (-5·7–16·7)
Central Asia	Lower respiratory infections	7·5 (-14·1–30·1)	3·4 (-4·3–12·9)	1·1 (-0·20–2·5)	3·7 (-9·2–18·0)
Central Asia	Malaria	0 (0–0)	0 (0–0)	--	--
Central Asia	Measles	4·7 (-1·7–12·8)	--	3·8 (-1·2–10·0)	0·99 (-0·41–3·4)
Armenia	All causes	0·73 (0·32–1·2)	0·63 (0·34–0·99)	0·59 (0·30–0·92)	<0.1 (>-0.1–0·26)
Armenia	Diarrheal diseases	11·7 (-7·9–32·0)	5·4 (-1·1–13·6)	2·7 (-1·6–7·6)	4·6 (-5·0–15·0)
Armenia	Lower respiratory infections	6·3 (-11·7–26·1)	2·2 (-2·7–8·7)	0·82 (-0·16–1·9)	3·7 (-8·6–17·5)
Armenia	Malaria	0 (0–0)	0 (0–0)	--	--
Armenia	Measles	4·6 (-1·7–12·5)	--	3·6 (-1·2–9·6)	1·0 (-0·44–3·5)
Azerbaijan	All causes	3·0 (1·8–4·4)	2·7 (1·8–3·9)	2·5 (1·7–3·6)	0·17 (-0·17–0·58)
Azerbaijan	Diarrheal diseases	15·5 (-9·5–40·0)	8·9 (-2·0–21·8)	3·4 (-2·2–9·4)	4·7 (-5·0–15·5)
Azerbaijan	Lower respiratory infections	8·1 (-15·0–32·0)	3·9 (-4·9–14·9)	1·1 (-0·20–2·5)	3·9 (-9·4–18·2)
Azerbaijan	Malaria	0 (0–0)	0 (0–0)	--	--
Azerbaijan	Measles	5·8 (-2·1–15·8)	--	4·7 (-1·5–12·7)	1·2 (-0·50–4·1)

Georgia	All causes	0·26 (-0·14–0·78)	0·12 (>-0.1–0·31)	<0.1 (>-0.1–0·11)	0·12 (-0·11–0·42)
Georgia	Diarrheal diseases	8·6 (-5·9–24·6)	3·7 (-0·77–9·8)	1·2 (-0·72–3·6)	4·1 (-4·3–13·4)
Georgia	Lower respiratory infections	4·5 (-8·4–19·3)	1·5 (-1·8–6·2)	0·34 (>-0.1–0·81)	2·8 (-6·4–13·3)
Georgia	Malaria	0 (0–0)	0 (0–0)	--	--
Georgia	Measles	2·4 (-0·85–6·5)	--	1·9 (-0·60–5·0)	0·53 (-0·21–1·8)
Kazakhstan	All causes	0·62 (0·36–0·95)	0·55 (0·36–0·78)	0·52 (0·34–0·74)	<0.1 (>-0.1–0·17)
Kazakhstan	Diarrheal diseases	11·0 (-7·0–30·1)	5·3 (-1·1–13·7)	2·2 (-1·3–6·1)	4·2 (-4·4–13·8)
Kazakhstan	Lower respiratory infections	5·8 (-10·1–23·5)	2·3 (-2·7–8·8)	0·71 (-0·13–1·6)	3·1 (-7·1–14·9)
Kazakhstan	Malaria	0 (0–0)	0 (0–0)	--	--
Kazakhstan	Measles	3·9 (-1·4–10·6)	--	3·2 (-1·0–8·3)	0·80 (-0·33–2·8)
Kyrgyzstan	All causes	0·27 (<0.1–0·56)	0·20 (<0.1–0·34)	0·16 (<0.1–0·25)	<0.1 (>-0.1–0·21)
Kyrgyzstan	Diarrheal diseases	13·1 (-8·8–35·5)	6·7 (-1·5–17·1)	2·1 (-1·3–6·1)	5·4 (-5·9–17·3)
Kyrgyzstan	Lower respiratory infections	6·8 (-14·2–29·5)	2·7 (-3·4–10·6)	0·54 (-0·11–1·2)	4·1 (-10·4–20·2)
Kyrgyzstan	Malaria	0 (0–0)	0 (0–0)	--	--
Kyrgyzstan	Measles	3·9 (-1·5–10·8)	--	2·9 (-0·95–7·7)	1·0 (-0·46–3·5)
Mongolia	All causes	<0.1 (>-0.1–0·19)	<0.1 (<0.1–<0.1)	<0.1 (<0.1–<0.1)	<0.1 (>-0.1–0·10)
Mongolia	Diarrheal diseases	11·0 (-8·0–31·0)	5·3 (-1·2–13·8)	1·2 (-0·70–3·4)	5·2 (-5·9–17·2)
Mongolia	Lower respiratory infections	5·5 (-11·7–23·9)	2·0 (-2·4–7·7)	0·29 (>-0.1–0·68)	3·6 (-9·0–17·1)
Mongolia	Malaria	0 (0–0)	0 (0–0)	--	--
Mongolia	Measles	0 (0–0)	--	0 (0–0)	0 (0–0)
Tajikistan	All causes	4·7 (3·3–6·4)	4·4 (3·3–5·9)	4·2 (3·1–5·5)	0·19 (-0·21–0·66)
Tajikistan	Diarrheal diseases	20·8 (-13·5–50·9)	12·8 (-3·1–30·1)	5·0 (-3·3–13·8)	5·8 (-6·5–18·7)
Tajikistan	Lower respiratory infections	10·8 (-21·8–41·4)	5·7 (-7·8–21·4)	1·7 (-0·30–3·7)	4·7 (-12·6–22·6)
Tajikistan	Malaria	0 (0–0)	0 (0–0)	--	--
Tajikistan	Measles	8·9 (-3·3–23·6)	--	7·3 (-2·5–18·5)	1·8 (-0·88–6·2)
Turkmenistan	All causes	1·7 (1·1–2·3)	1·6 (1·1–2·2)	1·5 (1·1–2·1)	<0.1 (>-0.1–0·19)
Turkmenistan	Diarrheal diseases	14·3 (-8·5–37·6)	8·1 (-1·8–20·1)	3·6 (-2·3–10·1)	4·0 (-4·1–13·0)
Turkmenistan	Lower respiratory infections	6·9 (-11·7–27·5)	3·4 (-4·3–13·3)	1·0 (-0·20–2·4)	3·0 (-7·0–14·4)
Turkmenistan	Malaria	0 (0–0)	0 (0–0)	--	--

Turkmenistan	Measles	0 (0–0)	--	0 (0–0)	0 (0–0)
Uzbekistan	All causes	0·60 (0·36–1·0)	0·54 (0·34–0·85)	0·50 (0·31–0·80)	<0.1 (>-0.1–0·18)
Uzbekistan	Diarrheal diseases	14·9 (-10·2–39·7)	7·7 (-1·7–19·2)	3·0 (-1·9–8·5)	5·7 (-6·4–18·4)
Uzbekistan	Lower respiratory infections	7·3 (-13·6–29·5)	3·1 (-3·9–12·1)	1·1 (-0·22–2·7)	3·6 (-8·9–17·9)
Uzbekistan	Malaria	0 (0–0)	0 (0–0)	--	--
Uzbekistan	Measles	5·5 (-1·9–14·8)	--	4·9 (-1·6–12·9)	0·72 (-0·31–2·5)
Central Europe	All causes	0·44 (<0.1–0·93)	0·31 (0·17–0·52)	0·26 (0·15–0·40)	<0.1 (>-0.1–0·30)
Central Europe	Diarrheal diseases	9·9 (-6·3–27·3)	4·6 (-0·98–11·8)	2·5 (-1·5–7·0)	3·5 (-3·7–11·6)
Central Europe	Lower respiratory infections	5·0 (-9·0–21·1)	1·8 (-2·1–7·1)	0·62 (-0·12–1·5)	2·9 (-6·6–13·9)
Central Europe	Malaria	0 (0–0)	0 (0–0)	--	--
Central Europe	Measles	3·4 (-1·2–9·3)	--	2·8 (-0·90–7·5)	0·64 (-0·27–2·3)
Albania	All causes	1·4 (0·59–2·3)	1·2 (0·61–1·9)	1·2 (0·57–1·8)	0·13 (-0·15–0·44)
Albania	Diarrheal diseases	13·9 (-9·8–37·6)	6·3 (-1·4–16·0)	3·7 (-2·3–10·4)	5·3 (-5·8–17·3)
Albania	Lower respiratory infections	8·2 (-15·0–32·7)	2·7 (-3·3–10·9)	1·6 (-0·29–3·7)	4·5 (-11·0–21·6)
Albania	Malaria	0 (0–0)	0 (0–0)	--	--
Albania	Measles	4·8 (-2·1–19·4)	--	3·7 (-1·4–14·8)	1·2 (-0·62–5·6)
Bosnia and Herzegovina	All causes	0·55 (0·20–1·1)	0·41 (0·25–0·65)	0·38 (0·23–0·60)	<0.1 (>-0.1–0·32)
Bosnia and Herzegovina	Diarrheal diseases	9·0 (-6·0–25·1)	3·6 (-0·74–9·4)	2·3 (-1·5–6·7)	3·6 (-3·7–11·6)
Bosnia and Herzegovina	Lower respiratory infections	4·8 (-8·5–20·3)	1·4 (-1·6–5·4)	0·65 (-0·12–1·5)	3·0 (-6·6–14·4)
Bosnia and Herzegovina	Malaria	0 (0–0)	0 (0–0)	--	--
Bosnia and Herzegovina	Measles	3·3 (-1·3–11·0)	--	2·5 (-0·94–7·9)	0·84 (-0·41–3·4)
Bulgaria	All causes	0·76 (0·45–1·2)	0·68 (0·44–1·0)	0·65 (0·42–0·96)	<0.1 (>-0.1–0·15)
Bulgaria	Diarrheal diseases	8·5 (-4·9–23·6)	4·1 (-0·86–10·6)	2·6 (-1·6–7·4)	2·4 (-2·3–7·9)
Bulgaria	Lower respiratory infections	4·0 (-6·4–16·4)	1·6 (-1·9–6·3)	0·58 (-0·12–1·4)	2·0 (-4·4–9·6)
Bulgaria	Malaria	0 (0–0)	0 (0–0)	--	--
Bulgaria	Measles	0 (0–0)	--	0 (0–0)	0 (0–0)
Croatia	All causes	0·42 (>-0.1–0·98)	0·26 (<0.1–0·51)	0·21 (<0.1–0·39)	<0.1 (-0·10–0·33)
Croatia	Diarrheal diseases	9·3 (-5·7–25·8)	4·2 (-0·89–10·9)	2·6 (-1·6–7·5)	3·1 (-3·1–10·1)
Croatia	Lower respiratory infections	4·9 (-8·4–20·3)	1·7 (-2·0–6·9)	0·64 (-0·13–1·5)	2·7 (-6·1–13·1)

Croatia	Malaria	0 (0–0)	0 (0–0)	--	--
Croatia	Measles	0 (0–0)	--	0 (0–0)	0 (0–0)
Czechia	All causes	0·72 (0·18–1·4)	0·54 (0·29–0·88)	0·48 (0·26–0·78)	<0.1 (>-0.1–0·29)
Czechia	Diarrheal diseases	8·5 (-4·7–23·2)	4·2 (-0·88–10·7)	2·8 (-1·7–7·9)	2·1 (-2·0–6·9)
Czechia	Lower respiratory infections	3·7 (-5·4–14·9)	1·5 (-1·7–5·9)	0·70 (-0·13–1·6)	1·6 (-3·5–8·0)
Czechia	Malaria	0 (0–0)	0 (0–0)	--	--
Czechia	Measles	2·9 (-1·1–8·9)	--	2·8 (-1·0–8·5)	0·11 (>-0.1–0·47)
Hungary	All causes	0·50 (-0·18–1·3)	0·26 (<0.1–0·55)	0·19 (<0.1–0·40)	0·16 (-0·16–0·54)
Hungary	Diarrheal diseases	8·6 (-5·6–24·3)	3·6 (-0·75–9·3)	2·2 (-1·4–6·4)	3·3 (-3·3–10·9)
Hungary	Lower respiratory infections	4·5 (-8·1–19·2)	1·3 (-1·6–5·4)	0·48 (>-0.1–1·1)	2·8 (-6·4–13·6)
Hungary	Malaria	0 (0–0)	0 (0–0)	--	--
Hungary	Measles	0 (0–0)	--	0 (0–0)	0 (0–0)
Montenegro	All causes	0·48 (0·12–0·88)	0·39 (0·23–0·59)	0·33 (0·21–0·49)	<0.1 (>-0.1–0·29)
Montenegro	Diarrheal diseases	10·6 (-6·9–29·2)	5·1 (-1·1–13·0)	1·8 (-1·1–5·0)	4·4 (-4·6–14·3)
Montenegro	Lower respiratory infections	5·9 (-11·4–24·7)	2·2 (-2·6–8·4)	0·47 (>-0.1–1·1)	3·5 (-8·4–17·0)
Montenegro	Malaria	0 (0–0)	0 (0–0)	--	--
Montenegro	Measles	0 (0–0)	--	0 (0–0)	0 (0–0)
North Macedonia	All causes	0·89 (0·32–1·7)	0·68 (0·42–1·1)	0·64 (0·39–1·0)	0·12 (-0·12–0·44)
North Macedonia	Diarrheal diseases	9·3 (-6·1–26·2)	3·8 (-0·79–9·9)	2·8 (-1·7–7·9)	3·3 (-3·4–11·0)
North Macedonia	Lower respiratory infections	5·0 (-9·1–20·9)	1·6 (-1·9–6·1)	0·57 (-0·11–1·3)	3·0 (-7·0–14·7)
North Macedonia	Malaria	0 (0–0)	0 (0–0)	--	--
North Macedonia	Measles	4·0 (-1·4–10·9)	--	3·5 (-1·1–9·2)	0·61 (-0·26–2·1)
Poland	All causes	0·18 (>-0.1–0·39)	0·12 (<0.1–0·24)	0·11 (<0.1–0·20)	<0.1 (>-0.1–0·13)
Poland	Diarrheal diseases	9·0 (-5·5–25·1)	4·0 (-0·83–10·4)	2·4 (-1·5–7·1)	3·1 (-3·1–10·1)
Poland	Lower respiratory infections	4·7 (-8·2–19·6)	1·6 (-2·0–6·5)	0·62 (-0·12–1·4)	2·6 (-5·9–12·8)
Poland	Malaria	0 (0–0)	0 (0–0)	--	--
Poland	Measles	3·5 (-1·2–9·6)	--	3·0 (-0·99–8·1)	0·50 (-0·21–1·7)
Romania	All causes	0·41 (-0·18–1·1)	0·23 (<0.1–0·53)	0·12 (<0.1–0·26)	0·15 (-0·16–0·54)
Romania	Diarrheal diseases	12·3 (-8·4–33·8)	6·0 (-1·3–14·9)	2·1 (-1·3–6·1)	5·2 (-5·7–17·3)

Romania	Lower respiratory infections	6·6 (-14·3–28·3)	2·4 (-3·0–9·6)	0·56 (-0·11–1·3)	4·1 (-10·9–19·8)
Romania	Malaria	0 (0–0)	0 (0–0)	--	--
Romania	Measles	3·4 (-1·2–9·2)	--	2·8 (-0·89–7·4)	0·64 (-0·27–2·3)
Serbia	All causes	0·66 (0·22–1·2)	0·52 (0·29–0·85)	0·44 (0·27–0·66)	<0.1 (-0·10–0·33)
Serbia	Diarrheal diseases	12·0 (-7·4–32·2)	6·2 (-1·4–15·7)	2·7 (-1·7–7·5)	4·1 (-4·2–13·3)
Serbia	Lower respiratory infections	6·2 (-11·3–25·4)	2·6 (-3·2–9·9)	0·60 (-0·12–1·4)	3·4 (-7·7–16·2)
Serbia	Malaria	0 (0–0)	0 (0–0)	--	--
Serbia	Measles	4·2 (-1·5–11·5)	--	3·5 (-1·1–9·1)	0·82 (-0·36–2·8)
Slovakia	All causes	0·38 (<0.1–0·85)	0·26 (<0.1–0·48)	0·22 (<0.1–0·37)	<0.1 (>-0.1–0·27)
Slovakia	Diarrheal diseases	9·9 (-6·1–27·3)	4·6 (-0·98–11·9)	2·6 (-1·6–7·6)	3·3 (-3·4–10·8)
Slovakia	Lower respiratory infections	5·2 (-9·1–21·4)	1·9 (-2·3–7·5)	0·66 (-0·13–1·5)	2·9 (-6·5–13·9)
Slovakia	Malaria	0 (0–0)	0 (0–0)	--	--
Slovakia	Measles	0 (0–0)	--	0 (0–0)	0 (0–0)
Slovenia	All causes	0·30 (-0·12–0·78)	0·16 (<0.1–0·36)	0·13 (>-0.1–0·29)	<0.1 (>-0.1–0·30)
Slovenia	Diarrheal diseases	8·1 (-5·0–22·9)	3·4 (-0·71–9·0)	2·3 (-1·4–6·5)	2·8 (-2·8–9·5)
Slovenia	Lower respiratory infections	4·2 (-7·0–17·5)	1·4 (-1·6–5·5)	0·57 (-0·11–1·3)	2·4 (-5·2–11·5)
Slovenia	Malaria	0 (0–0)	0 (0–0)	--	--
Slovenia	Measles	0 (0–0)	--	0 (0–0)	0 (0–0)
Eastern Europe	All causes	1·8 (1·2–2·4)	1·7 (1·3–2·3)	1·7 (1·2–2·2)	<0.1 (>-0.1–0·20)
Eastern Europe	Diarrheal diseases	9·9 (-6·3–27·4)	4·6 (-0·97–11·8)	2·3 (-1·4–6·8)	3·6 (-3·8–11·9)
Eastern Europe	Lower respiratory infections	5·7 (-10·1–23·8)	1·9 (-2·3–7·5)	0·77 (-0·15–1·8)	3·3 (-7·5–16·1)
Eastern Europe	Malaria	0 (0–0)	0 (0–0)	--	--
Eastern Europe	Measles	4·5 (-1·7–12·8)	--	3·5 (-1·1–9·4)	1·1 (-0·51–3·8)
Belarus	All causes	0·60 (-0·20–1·6)	0·38 (>-0.1–0·93)	0·17 (>-0.1–0·38)	0·13 (-0·13–0·47)
Belarus	Diarrheal diseases	8·6 (-4·3–23·3)	5·2 (-1·1–13·3)	1·7 (-1·0–4·9)	2·1 (-2·1–7·1)
Belarus	Lower respiratory infections	4·1 (-6·3–16·9)	2·3 (-2·7–9·0)	0·37 (>-0.1–0·85)	1·6 (-3·4–7·9)
Belarus	Malaria	0 (0–0)	0 (0–0)	--	--
Belarus	Measles	2·2 (-0·73–6·0)	--	2·0 (-0·64–5·3)	0·22 (>-0.1–0·76)
Estonia	All causes	0·67 (-0·37–1·9)	0·32 (>-0.1–0·83)	0·18 (>-0.1–0·51)	0·22 (-0·22–0·74)

Estonia	Diarrheal diseases	8·3 (-5·0–23·4)	3·9 (-0·81–10·1)	2·1 (-1·3–6·1)	2·8 (-2·8–9·3)
Estonia	Lower respiratory infections	4·4 (-7·4–18·4)	1·7 (-2·0–6·7)	0·53 (-0·10–1·2)	2·4 (-5·2–11·5)
Estonia	Malaria	0 (0–0)	0 (0–0)	--	--
Estonia	Measles	3·2 (-1·1–8·9)	--	2·8 (-0·94–7·6)	0·40 (-0·18–1·4)
Latvia	All causes	0·50 (-0·23–1·4)	0·26 (>-0.1–0·64)	0·14 (>-0.1–0·37)	0·16 (-0·15–0·51)
Latvia	Diarrheal diseases	9·3 (-5·6–26·0)	4·6 (-0·97–11·9)	2·2 (-1·4–6·4)	3·1 (-3·2–10·4)
Latvia	Lower respiratory infections	4·9 (-8·5–20·4)	1·9 (-2·3–7·7)	0·56 (-0·11–1·3)	2·6 (-5·9–12·7)
Latvia	Malaria	0 (0–0)	0 (0–0)	--	--
Latvia	Measles	0 (0–0)	--	0 (0–0)	0 (0–0)
Lithuania	All causes	0·53 (-0·28–1·5)	0·26 (>-0.1–0·67)	0·14 (>-0.1–0·40)	0·17 (-0·17–0·58)
Lithuania	Diarrheal diseases	8·5 (-5·1–23·9)	4·1 (-0·85–10·5)	2·1 (-1·3–6·1)	2·8 (-2·9–9·4)
Lithuania	Lower respiratory infections	4·5 (-7·6–18·6)	1·7 (-2·0–6·7)	0·52 (-0·10–1·2)	2·4 (-5·4–11·7)
Lithuania	Malaria	0 (0–0)	0 (0–0)	--	--
Lithuania	Measles	2·1 (-0·88–8·6)	--	1·8 (-0·74–7·4)	0·28 (-0·14–1·4)
Republic of Moldova	All causes	0·61 (-0·23–1·6)	0·35 (<0.1–0·81)	0·20 (<0.1–0·46)	0·16 (-0·16–0·57)
Republic of Moldova	Diarrheal diseases	10·2 (-5·9–27·9)	5·3 (-1·1–13·7)	2·5 (-1·5–7·1)	3·1 (-3·1–10·2)
Republic of Moldova	Lower respiratory infections	5·3 (-9·0–21·5)	2·3 (-2·8–8·8)	0·53 (-0·10–1·2)	2·7 (-6·0–12·9)
Republic of Moldova	Malaria	0 (0–0)	0 (0–0)	--	--
Republic of Moldova	Measles	3·5 (-1·0–9·8)	--	3·2 (-0·90–8·7)	0·35 (-0·13–1·2)
Russian Federation	All causes	0·67 (0·38–1·0)	0·60 (0·36–0·85)	0·58 (0·35–0·83)	<0.1 (>-0.1–0·17)
Russian Federation	Diarrheal diseases	10·3 (-6·9–28·8)	4·4 (-0·92–11·4)	2·6 (-1·6–7·5)	4·1 (-4·3–13·3)
Russian Federation	Lower respiratory infections	5·8 (-10·2–24·1)	1·9 (-2·2–7·4)	0·87 (-0·17–2·1)	3·3 (-7·6–16·3)
Russian Federation	Malaria	0 (0–0)	0 (0–0)	--	--
Russian Federation	Measles	4·6 (-1·7–12·8)	--	3·5 (-1·1–9·5)	1·1 (-0·51–3·8)
Ukraine	All causes	7·2 (5·2–9·5)	7·1 (5·1–9·3)	7·1 (5·1–9·3)	<0.1 (>-0.1–0·17)
Ukraine	Diarrheal diseases	11·0 (-7·9–30·9)	4·6 (-0·97–11·7)	2·2 (-1·4–6·5)	5·0 (-5·4–16·3)
Ukraine	Lower respiratory infections	6·4 (-12·8–28·0)	1·8 (-2·2–7·2)	0·57 (-0·11–1·3)	4·4 (-10·3–21·2)
Ukraine	Malaria	0 (0–0)	0 (0–0)	--	--
Ukraine	Measles	5·0 (-2·0–14·9)	--	3·0 (-0·98–8·0)	2·1 (-1·1–7·4)

**High-income**	**All causes**	**0·18 (>-0.1–0·47)**	**0·10 (<0.1–0·24)**	**<0.1 (<0.1–0·14)**	**<0.1 (>-0.1–0·16)**
**High-income**	**Diarrheal diseases**	**5·8 (-3·2–16·5)**	**2·9 (-0·60–7·7)**	**1·4 (-0·83–4·1)**	**1·7 (-1·7–5·9)**
**High-income**	**Lower respiratory infections**	**3·3 (-5·2–13·6)**	**1·3 (-1·5–5·1)**	**0·45 (>-0.1–1·0)**	**1·6 (-3·6–8·1)**
**High-income**	**Malaria**	**8·3 (-3·4–29·8)**	**8·3 (-3·4–29·8)**	**--**	**--**
**High-income**	**Measles**	**2·0 (-0·66–5·5)**	**--**	**1·9 (-0·62–5·2)**	**<0.1 (>-0.1–0·34)**
Australasia	All causes	0·14 (>-0.1–0·44)	<0.1 (>-0.1–0·12)	<0.1 (>-0.1–0·17)	<0.1 (>-0.1–0·19)
Australasia	Diarrheal diseases	4·1 (-2·6–12·1)	1·1 (-0·23–3·1)	1·5 (-0·91–4·5)	1·5 (-1·5–5·1)
Australasia	Lower respiratory infections	2·0 (-3·1–8·4)	0·48 (-0·54–1·9)	0·34 (>-0.1–0·78)	1·2 (-2·5–5·9)
Australasia	Malaria	0 (0–0)	0 (0–0)	--	--
Australasia	Measles	2·2 (-0·72–5·9)	--	2·1 (-0·68–5·6)	0·10 (>-0.1–0·38)
Australia	All causes	0·15 (>-0.1–0·46)	<0.1 (>-0.1–0·11)	<0.1 (>-0.1–0·17)	<0.1 (>-0.1–0·19)
Australia	Diarrheal diseases	3·9 (-2·6–11·8)	1·1 (-0·21–2·9)	1·5 (-0·91–4·5)	1·5 (-1·4–5·0)
Australia	Lower respiratory infections	1·8 (-2·8–7·7)	0·39 (-0·45–1·6)	0·34 (>-0.1–0·77)	1·1 (-2·3–5·5)
Australia	Malaria	0 (0–0)	0 (0–0)	--	--
Australia	Measles	2·1 (-0·70–5·7)	--	2·1 (-0·67–5·5)	<0.1 (>-0.1–0·30)
New Zealand	All causes	0·13 (>-0.1–0·41)	<0.1 (>-0.1–0·13)	<0.1 (>-0.1–0·13)	<0.1 (>-0.1–0·17)
New Zealand	Diarrheal diseases	4·9 (-3·0–14·3)	1·8 (-0·35–4·6)	1·5 (-0·93–4·5)	1·8 (-1·7–6·0)
New Zealand	Lower respiratory infections	2·5 (-3·9–10·5)	0·73 (-0·84–2·9)	0·35 (>-0.1–0·81)	1·4 (-3·0–7·0)
New Zealand	Malaria	0 (0–0)	0 (0–0)	--	--
New Zealand	Measles	2·3 (-0·76–6·2)	--	2·2 (-0·71–5·8)	0·13 (>-0.1–0·47)
High-income Asia Pacific	All causes	0·41 (<0.1–0·90)	0·31 (0·14–0·56)	0·23 (0·13–0·37)	<0.1 (>-0.1–0·22)
High-income Asia Pacific	Diarrheal diseases	9·1 (-4·8–24·8)	5·1 (-1·1–13·1)	2·2 (-1·3–6·4)	2·3 (-2·3–7·6)
High-income Asia Pacific	Lower respiratory infections	4·1 (-6·2–16·4)	2·0 (-2·4–7·7)	0·56 (-0·11–1·3)	1·7 (-3·6–8·2)
High-income Asia Pacific	Malaria	8·3 (-3·4–29·8)	8·3 (-3·4–29·8)	--	--
High-income Asia Pacific	Measles	3·4 (-1·1–9·2)	--	3·2 (-1·0–8·5)	0·21 (>-0.1–0·74)
Brunei Darussalam	All causes	0·30 (0·11–0·57)	0·27 (0·10–0·52)	0·25 (<0.1–0·48)	<0.1 (>-0.1–<0.1)
Brunei Darussalam	Diarrheal diseases	13·2 (-8·6–35·7)	6·8 (-1·5–17·4)	2·4 (-1·5–7·1)	5·1 (-5·5–16·3)
Brunei Darussalam	Lower respiratory infections	7·2 (-13·9–29·9)	2·8 (-3·4–10·8)	0·66 (-0·13–1·5)	4·3 (-10·0–20·2)
Brunei Darussalam	Malaria	0 (0–0)	0 (0–0)	--	--

Brunei Darussalam	Measles	0<br>(0–0)	--	0<br>(0–0)	0<br>(0–0)
Japan	All causes	0·53<br>(<0.1–1·1)	0·40<br>(0·18–0·72)	0·29<br>(0·17–0·48)	<0.1<br>(>-0.1–0·28)
Japan	Diarrheal diseases	9·2<br>(-4·9–25·2)	5·2<br>(-1·1–13·4)	2·2<br>(-1·3–6·4)	2·3<br>(-2·3–7·8)
Japan	Lower respiratory infections	4·4<br>(-6·7–17·7)	2·2<br>(-2·6–8·5)	0·56<br>(-0·11–1·3)	1·8<br>(-3·9–8·8)
Japan	Malaria	0<br>(0–0)	0<br>(0–0)	--	--
Japan	Measles	3·3<br>(-1·1–9·1)	--	3·1<br>(-1·0–8·4)	0·24<br>(-0·10–0·84)
Republic of Korea	All causes	<0.1<br>(>-0.1–0·19)	<0.1<br>(>-0.1–<0.1)	<0.1<br>(>-0.1–<0.1)	<0.1<br>(>-0.1–<0.1)
Republic of Korea	Diarrheal diseases	7·0<br>(-3·6–19·3)	3·6<br>(-0·72–9·1)	2·1<br>(-1·3–6·1)	1·6<br>(-1·6–5·5)
Republic of Korea	Lower respiratory infections	3·1<br>(-4·4–12·4)	1·4<br>(-1·7–5·6)	0·52<br>(-0·10–1·2)	1·2<br>(-2·6–6·1)
Republic of Korea	Malaria	8·3<br>(-3·4–29·8)	8·3<br>(-3·4–29·8)	--	--
Republic of Korea	Measles	3·1<br>(-1·0–8·2)	--	3·0<br>(-0·99–8·0)	<0.1<br>(>-0.1–0·22)
Singapore	All causes	<0.1<br>(>-0.1–0·15)	<0.1<br>(<0.1–0·13)	<0.1<br>(<0.1–0·12)	<0.1<br>(>-0.1–<0.1)
Singapore	Diarrheal diseases	9·1<br>(-5·0–25·2)	4·8<br>(-1·0–12·6)	2·4<br>(-1·5–6·9)	2·4<br>(-2·4–8·1)
Singapore	Lower respiratory infections	4·3<br>(-6·6–17·4)	2·0<br>(-2·4–8·0)	0·64<br>(-0·12–1·5)	1·8<br>(-4·0–9·1)
Singapore	Malaria	0<br>(0–0)	0<br>(0–0)	--	--
Singapore	Measles	3·8<br>(-1·3–10·3)	--	3·6<br>(-1·2–9·6)	0·23<br>(-0·10–0·81)
High-income North America	All causes	<0.1<br>(>-0.1–<0.1)	<0.1<br>(>-0.1–<0.1)	<0.1<br>(>-0.1–<0.1)	<0.1<br>(>-0.1–<0.1)
High-income North America	Diarrheal diseases	6·5<br>(-3·8–18·3)	2·8<br>(-0·58–7·3)	1·9<br>(-1·2–5·7)	2·0<br>(-2·0–6·7)
High-income North America	Lower respiratory infections	3·3<br>(-5·0–13·3)	1·1<br>(-1·2–4·2)	0·66<br>(-0·13–1·5)	1·7<br>(-3·6–8·2)
High-income North America	Malaria	0<br>(0–0)	0<br>(0–0)	--	--
High-income North America	Measles	3·1<br>(-1·0–8·3)	--	3·0<br>(-0·94–7·9)	0·15<br>(>-0.1–0·53)
Canada	All causes	<0.1<br>(>-0.1–<0.1)	<0.1<br>(>-0.1–<0.1)	<0.1<br>(>-0.1–<0.1)	<0.1<br>(>-0.1–<0.1)
Canada	Diarrheal diseases	4·5<br>(-2·2–12·6)	2·1<br>(-0·42–5·5)	1·9<br>(-1·2–5·6)	0·61<br>(-0·58–2·0)
Canada	Lower respiratory infections	1·8<br>(-2·0–6·8)	0·89<br>(-0·98–3·5)	0·47<br>(>-0.1–1·1)	0·47<br>(-0·92–2·3)
Canada	Malaria	0<br>(0–0)	0<br>(0–0)	--	--
Canada	Measles	2·4<br>(-0·92–7·4)	--	2·3<br>(-0·91–7·2)	<0.1<br>(>-0.1–0·16)
Greenland	All causes	<0.1<br>(>-0.1–<0.1)	<0.1<br>(>-0.1–<0.1)	<0.1<br>(>-0.1–<0.1)	<0.1<br>(>-0.1–<0.1)
Greenland	Diarrheal diseases	7·3<br>(-4·2–20·5)	3·5<br>(-0·72–9·1)	2·0<br>(-1·2–5·8)	2·2<br>(-2·2–7·3)
Greenland	Lower respiratory infections	3·6<br>(-5·5–15·0)	1·5<br>(-1·7–5·8)	0·52<br>(-0·10–1·2)	1·7<br>(-3·6–8·6)

Greenland	Malaria	0 (0–0)	0 (0–0)	--	--
Greenland	Measles	3·0 (-0·97–8·1)	--	2·8 (-0·90–7·6)	0·17 (>-0.1–0·56)
United States of America	All causes	<0.1 (>-0.1–<0.1)	<0.1 (>-0.1–<0.1)	<0.1 (>-0.1–<0.1)	<0.1 (>-0.1–<0.1)
United States of America	Diarrheal diseases	7·0 (-4·2–19·8)	3·0 (-0·62–7·8)	2·0 (-1·2–5·7)	2·3 (-2·3–7·8)
United States of America	Lower respiratory infections	3·4 (-5·2–13·7)	1·1 (-1·2–4·2)	0·67 (-0·13–1·5)	1·7 (-3·8–8·5)
United States of America	Malaria	0 (0–0)	0 (0–0)	--	--
United States of America	Measles	3·4 (-1·1–9·1)	--	3·2 (-1·0–8·7)	0·13 (>-0.1–0·45)
Southern Latin America	All causes	0·26 (-0·15–0·73)	0·14 (>-0.1–0·36)	<0.1 (>-0.1–0·14)	<0.1 (>-0.1–0·31)
Southern Latin America	Diarrheal diseases	9·3 (-5·5–26·0)	4·9 (-1·0–12·7)	1·6 (-1·0–4·8)	3·3 (-3·4–11·0)
Southern Latin America	Lower respiratory infections	4·8 (-8·9–20·7)	1·9 (-2·3–7·6)	0·36 (>-0.1–0·83)	2·7 (-6·4–13·5)
Southern Latin America	Malaria	0 (0–0)	0 (0–0)	--	--
Southern Latin America	Measles	1·0 (-0·62–4·6)	--	0·98 (-0·58–4·4)	<0.1 (>-0.1–0·22)
Argentina	All causes	0·26 (-0·16–0·77)	0·13 (>-0.1–0·35)	<0.1 (>-0.1–0·14)	0·10 (-0·11–0·36)
Argentina	Diarrheal diseases	9·8 (-6·2–27·8)	4·8 (-1·0–12·7)	1·7 (-1·0–5·1)	3·9 (-4·1–12·9)
Argentina	Lower respiratory infections	5·1 (-9·7–21·9)	1·9 (-2·3–7·5)	0·38 (>-0.1–0·87)	3·0 (-7·1–14·8)
Argentina	Malaria	0 (0–0)	0 (0–0)	--	--
Argentina	Measles	0 (0–0)	--	0 (0–0)	0 (0–0)
Chile	All causes	0·24 (-0·12–0·74)	0·15 (>-0.1–0·43)	<0.1 (>-0.1–0·15)	<0.1 (>-0.1–0·22)
Chile	Diarrheal diseases	7·7 (-3·7–21·3)	4·7 (-0·99–12·2)	1·4 (-0·87–4·2)	1·9 (-1·8–6·4)
Chile	Lower respiratory infections	3·6 (-5·3–14·6)	1·9 (-2·3–7·5)	0·30 (>-0.1–0·68)	1·4 (-3·1–7·1)
Chile	Malaria	0 (0–0)	0 (0–0)	--	--
Chile	Measles	1·0 (-0·62–4·6)	--	0·98 (-0·58–4·4)	<0.1 (>-0.1–0·22)
Uruguay	All causes	0·36 (-0·18–1·0)	0·20 (>-0.1–0·53)	<0.1 (>-0.1–0·16)	0·12 (-0·12–0·43)
Uruguay	Diarrheal diseases	11·0 (-6·5–29·9)	6·2 (-1·3–15·8)	1·6 (-1·0–4·8)	3·9 (-4·0–12·7)
Uruguay	Lower respiratory infections	5·9 (-10·7–24·7)	2·7 (-3·3–10·4)	0·39 (>-0.1–0·88)	3·2 (-7·1–15·3)
Uruguay	Malaria	0 (0–0)	0 (0–0)	--	--
Uruguay	Measles	0 (0–0)	--	0 (0–0)	0 (0–0)
Western Europe	All causes	0·24 (>-0.1–0·66)	0·13 (>-0.1–0·31)	<0.1 (>-0.1–0·18)	<0.1 (>-0.1–0·23)
Western Europe	Diarrheal diseases	4·8 (-2·5–13·6)	2·4 (-0·48–6·2)	1·1 (-0·68–3·3)	1·4 (-1·4–4·8)

Western Europe	Lower respiratory infections	2·2 (-3·3–9·2)	0·93 (-1·1–3·7)	0·24 (>-0.1–0·56)	1·1 (-2·2–5·3)
Western Europe	Malaria	0 (0–0)	0 (0–0)	--	--
Western Europe	Measles	1·7 (-0·55–4·5)	--	1·6 (-0·52–4·3)	<0.1 (>-0.1–0·26)
Andorra	All causes	0·20 (-0·10–0·59)	<0.1 (>-0.1–0·25)	<0.1 (>-0.1–0·15)	<0.1 (>-0.1–0·21)
Andorra	Diarrheal diseases	4·2 (-2·3–12·0)	1·9 (-0·38–5·1)	1·1 (-0·62–3·1)	1·3 (-1·2–4·4)
Andorra	Lower respiratory infections	2·0 (-3·1–8·4)	0·78 (-0·92–3·1)	0·22 (>-0.1–0·53)	1·0 (-2·1–4·9)
Andorra	Malaria	0 (0–0)	0 (0–0)	--	--
Andorra	Measles	0 (0–0)	--	0 (0–0)	0 (0–0)
Austria	All causes	0·27 (-0·15–0·77)	0·13 (>-0.1–0·34)	<0.1 (>-0.1–0·19)	<0.1 (>-0.1–0·28)
Austria	Diarrheal diseases	4·6 (-2·5–13·3)	2·2 (-0·44–5·7)	1·1 (-0·66–3·2)	1·5 (-1·4–4·9)
Austria	Lower respiratory infections	2·2 (-3·5–8·8)	0·86 (-1·0–3·3)	0·23 (>-0.1–0·51)	1·1 (-2·4–5·2)
Austria	Malaria	0 (0–0)	0 (0–0)	--	--
Austria	Measles	1·7 (-0·56–4·6)	--	1·6 (-0·53–4·4)	<0.1 (>-0.1–0·31)
Belgium	All causes	0·23 (-0·12–0·66)	0·11 (>-0.1–0·29)	<0.1 (>-0.1–0·17)	<0.1 (>-0.1–0·25)
Belgium	Diarrheal diseases	4·7 (-2·6–13·4)	2·2 (-0·46–5·8)	1·2 (-0·71–3·4)	1·5 (-1·4–4·9)
Belgium	Lower respiratory infections	2·1 (-3·3–9·0)	0·86 (-0·97–3·4)	0·24 (>-0.1–0·56)	1·1 (-2·2–5·3)
Belgium	Malaria	0 (0–0)	0 (0–0)	--	--
Belgium	Measles	1·7 (-0·56–4·6)	--	1·6 (-0·52–4·3)	<0.1 (>-0.1–0·30)
Cyprus	All causes	0·41 (-0·14–1·1)	0·23 (<0.1–0·57)	0·12 (>-0.1–0·31)	0·11 (-0·11–0·38)
Cyprus	Diarrheal diseases	5·9 (-3·2–16·8)	3·0 (-0·61–7·9)	1·3 (-0·79–3·9)	1·8 (-1·7–6·0)
Cyprus	Lower respiratory infections	2·7 (-4·4–11·5)	1·2 (-1·4–4·6)	0·28 (>-0.1–0·65)	1·3 (-2·9–6·6)
Cyprus	Malaria	0 (0–0)	0 (0–0)	--	--
Cyprus	Measles	0 (0–0)	--	0 (0–0)	0 (0–0)
Denmark	All causes	0·30 (-0·13–0·84)	0·15 (>-0.1–0·38)	<0.1 (>-0.1–0·22)	<0.1 (>-0.1–0·29)
Denmark	Diarrheal diseases	5·0 (-2·7–14·3)	2·4 (-0·49–6·3)	1·2 (-0·74–3·6)	1·5 (-1·5–5·1)
Denmark	Lower respiratory infections	2·3 (-3·6–9·4)	0·94 (-1·1–3·6)	0·26 (>-0.1–0·61)	1·1 (-2·4–5·4)
Denmark	Malaria	0 (0–0)	0 (0–0)	--	--
Denmark	Measles	0 (0–0)	--	0 (0–0)	0 (0–0)
Finland	All causes	0·21 (>-0.1–0·56)	0·11 (>-0.1–0·27)	<0.1 (>-0.1–0·16)	<0.1 (>-0.1–0·20)

Finland	Diarrheal diseases	5·1 (-2·8–14·4)	2·5 (-0·50–6·4)	1·2 (-0·73–3·5)	1·6 (-1·5–5·2)
Finland	Lower respiratory infections	2·3 (-3·7–10·1)	0·98 (-1·1–3·9)	0·25 (>-0.1–0·59)	1·2 (-2·5–5·9)
Finland	Malaria	0 (0–0)	0 (0–0)	--	--
Finland	Measles	0 (0–0)	--	0 (0–0)	0 (0–0)
France	All causes	0·29 (-0·14–0·84)	0·14 (>-0.1–0·36)	<0.1 (>-0.1–0·22)	<0.1 (>-0.1–0·30)
France	Diarrheal diseases	4·6 (-2·5–13·2)	2·2 (-0·43–5·7)	1·1 (-0·68–3·3)	1·4 (-1·4–4·8)
France	Lower respiratory infections	2·1 (-3·2–9·3)	0·85 (-0·94–3·5)	0·24 (>-0.1–0·57)	1·1 (-2·2–5·5)
France	Malaria	0 (0–0)	0 (0–0)	--	--
France	Measles	1·7 (-0·56–4·6)	--	1·6 (-0·52–4·3)	<0.1 (>-0.1–0·30)
Germany	All causes	0·30 (-0·13–0·89)	0·17 (>-0.1–0·46)	<0.1 (>-0.1–0·21)	<0.1 (>-0.1–0·25)
Germany	Diarrheal diseases	4·9 (-2·4–13·6)	2·7 (-0·55–7·1)	1·1 (-0·69–3·3)	1·2 (-1·1–3·9)
Germany	Lower respiratory infections	2·2 (-3·1–9·1)	1·1 (-1·3–4·3)	0·25 (>-0.1–0·59)	0·91 (-1·8–4·5)
Germany	Malaria	0 (0–0)	0 (0–0)	--	--
Germany	Measles	1·8 (-0·59–4·9)	--	1·7 (-0·56–4·6)	<0.1 (>-0.1–0·25)
Greece	All causes	0·21 (-0·11–0·60)	<0.1 (>-0.1–0·27)	<0.1 (>-0.1–0·14)	<0.1 (>-0.1–0·24)
Greece	Diarrheal diseases	4·9 (-2·8–14·0)	2·3 (-0·47–6·1)	1·1 (-0·67–3·2)	1·7 (-1·7–5·5)
Greece	Lower respiratory infections	2·4 (-4·0–10·2)	0·93 (-1·1–3·7)	0·24 (>-0.1–0·56)	1·3 (-2·8–6·3)
Greece	Malaria	0 (0–0)	0 (0–0)	--	--
Greece	Measles	0 (0–0)	--	0 (0–0)	0 (0–0)
Iceland	All causes	0·23 (-0·11–0·68)	0·11 (>-0.1–0·30)	<0.1 (>-0.1–0·18)	<0.1 (>-0.1–0·25)
Iceland	Diarrheal diseases	4·5 (-2·4–13·0)	2·1 (-0·42–5·6)	1·1 (-0·67–3·3)	1·4 (-1·3–4·7)
Iceland	Lower respiratory infections	2·1 (-3·2–9·1)	0·86 (-0·98–3·4)	0·25 (>-0.1–0·56)	1·1 (-2·2–5·4)
Iceland	Malaria	0 (0–0)	0 (0–0)	--	--
Iceland	Measles	0 (0–0)	--	0 (0–0)	0 (0–0)
Ireland	All causes	0·21 (-0·11–0·61)	<0.1 (>-0.1–0·25)	<0.1 (>-0.1–0·17)	<0.1 (>-0.1–0·23)
Ireland	Diarrheal diseases	4·2 (-2·3–11·9)	1·9 (-0·37–4·9)	1·1 (-0·65–3·1)	1·3 (-1·2–4·4)
Ireland	Lower respiratory infections	1·9 (-3·0–8·4)	0·76 (-0·90–3·1)	0·23 (>-0.1–0·55)	0·99 (-2·1–5·0)
Ireland	Malaria	0 (0–0)	0 (0–0)	--	--
Ireland	Measles	0 (0–0)	--	0 (0–0)	0 (0–0)

Israel	All causes	0·32 (-0·16–1·0)	0·15 (>-0.1–0·43)	<0.1 (>-0.1–0·25)	0·10 (>-0.1–0·37)
Israel	Diarrheal diseases	4·4 (-2·4–12·5)	2·0 (-0·41–5·3)	1·1 (-0·64–3·1)	1·4 (-1·4–4·6)
Israel	Lower respiratory infections	2·1 (-3·1–8·7)	0·81 (-0·89–3·2)	0·22 (>-0.1–0·51)	1·1 (-2·1–5·2)
Israel	Malaria	0 (0–0)	0 (0–0)	--	--
Israel	Measles	1·6 (-0·53–4·4)	--	1·5 (-0·50–4·1)	<0.1 (>-0.1–0·29)
Italy	All causes	0·14 (>-0.1–0·41)	<0.1 (>-0.1–0·18)	<0.1 (>-0.1–0·11)	<0.1 (>-0.1–0·14)
Italy	Diarrheal diseases	4·5 (-2·5–12·8)	2·1 (-0·42–5·5)	1·1 (-0·66–3·2)	1·4 (-1·4–4·8)
Italy	Lower respiratory infections	2·1 (-3·4–9·0)	0·85 (-0·99–3·4)	0·24 (>-0.1–0·55)	1·1 (-2·3–5·4)
Italy	Malaria	0 (0–0)	0 (0–0)	--	--
Italy	Measles	1·6 (-0·53–4·4)	--	1·6 (-0·50–4·1)	<0.1 (>-0.1–0·28)
Luxembourg	All causes	0·25 (-0·12–0·73)	0·13 (>-0.1–0·34)	<0.1 (>-0.1–0·20)	<0.1 (>-0.1–0·25)
Luxembourg	Diarrheal diseases	5·1 (-2·7–14·3)	2·5 (-0·50–6·4)	1·2 (-0·75–3·6)	1·5 (-1·5–5·0)
Luxembourg	Lower respiratory infections	2·3 (-3·4–9·7)	0·97 (-1·0–3·8)	0·26 (>-0.1–0·60)	1·1 (-2·3–5·6)
Luxembourg	Malaria	0 (0–0)	0 (0–0)	--	--
Luxembourg	Measles	0 (0–0)	--	0 (0–0)	0 (0–0)
Malta	All causes	0·28 (-0·14–0·85)	0·15 (>-0.1–0·40)	<0.1 (>-0.1–0·22)	<0.1 (>-0.1–0·30)
Malta	Diarrheal diseases	5·1 (-2·7–14·3)	2·5 (-0·51–6·5)	1·2 (-0·70–3·4)	1·6 (-1·5–5·2)
Malta	Lower respiratory infections	2·4 (-3·8–10·3)	1·0 (-1·2–4·1)	0·26 (>-0.1–0·59)	1·2 (-2·6–5·9)
Malta	Malaria	0 (0–0)	0 (0–0)	--	--
Malta	Measles	0 (0–0)	--	0 (0–0)	0 (0–0)
Monaco	All causes	0·20 (>-0.1–0·59)	<0.1 (>-0.1–0·24)	<0.1 (>-0.1–0·16)	<0.1 (>-0.1–0·22)
Monaco	Diarrheal diseases	3·8 (-2·1–10·9)	1·7 (-0·33–4·3)	1·0 (-0·59–2·9)	1·2 (-1·2–4·1)
Monaco	Lower respiratory infections	1·8 (-2·8–7·4)	0·66 (-0·78–2·6)	0·21 (>-0.1–0·47)	0·94 (-2·0–4·6)
Monaco	Malaria	0 (0–0)	0 (0–0)	--	--
Monaco	Measles	0 (0–0)	--	0 (0–0)	0 (0–0)
Netherlands	All causes	0·24 (0·10–0·41)	0·21 (0·11–0·34)	0·18 (<0.1–0·29)	<0.1 (>-0.1–<0.1)
Netherlands	Diarrheal diseases	5·3 (-2·5–14·6)	3·1 (-0·65–8·2)	1·1 (-0·64–3·2)	1·2 (-1·2–4·1)
Netherlands	Lower respiratory infections	2·2 (-3·0–9·0)	1·1 (-1·2–4·4)	0·20 (>-0.1–0·47)	0·90 (-1·8–4·4)
Netherlands	Malaria	0 (0–0)	0 (0–0)	--	--

Netherlands	Measles	1·5 (-0·51–4·2)	--	1·4 (-0·49–4·0)	<0.1 (>-0.1–0·19)
Norway	All causes	0·14 (>-0.1–0·40)	<0.1 (>-0.1–0·18)	<0.1 (>-0.1–0·12)	<0.1 (>-0.1–0·14)
Norway	Diarrheal diseases	4·3 (-2·3–12·4)	2·0 (-0·40–5·3)	1·1 (-0·67–3·3)	1·3 (-1·3–4·4)
Norway	Lower respiratory infections	2·1 (-3·1–9·0)	0·83 (-0·93–3·4)	0·25 (>-0.1–0·58)	1·0 (-2·1–5·2)
Norway	Malaria	0 (0–0)	0 (0–0)	--	--
Norway	Measles	0 (0–0)	--	0 (0–0)	0 (0–0)
Portugal	All causes	0·19 (>-0.1–0·52)	0·11 (<0.1–0·25)	<0.1 (>-0.1–0·14)	<0.1 (>-0.1–0·19)
Portugal	Diarrheal diseases	5·5 (-2·9–15·5)	2·8 (-0·55–7·2)	1·2 (-0·73–3·6)	1·7 (-1·6–5·6)
Portugal	Lower respiratory infections	2·5 (-3·9–10·6)	1·1 (-1·2–4·2)	0·26 (>-0.1–0·58)	1·3 (-2·6–6·2)
Portugal	Malaria	0 (0–0)	0 (0–0)	--	--
Portugal	Measles	0·95 (-0·22–4·2)	--	0·90 (-0·20–4·0)	<0.1 (>-0.1–0·31)
San Marino	All causes	0·23 (-0·10–0·66)	0·11 (>-0.1–0·29)	<0.1 (>-0.1–0·17)	<0.1 (>-0.1–0·24)
San Marino	Diarrheal diseases	4·4 (-2·4–12·4)	2·0 (-0·40–5·3)	1·1 (-0·65–3·2)	1·4 (-1·3–4·6)
San Marino	Lower respiratory infections	2·1 (-3·4–8·8)	0·82 (-0·99–3·3)	0·24 (>-0.1–0·55)	1·1 (-2·3–5·2)
San Marino	Malaria	0 (0–0)	0 (0–0)	--	--
San Marino	Measles	0 (0–0)	--	0 (0–0)	0 (0–0)
Spain	All causes	0·21 (-0·10–0·61)	0·10 (>-0.1–0·27)	<0.1 (>-0.1–0·16)	<0.1 (>-0.1–0·22)
Spain	Diarrheal diseases	4·8 (-2·6–13·6)	2·3 (-0·46–6·0)	1·2 (-0·69–3·4)	1·5 (-1·4–4·9)
Spain	Lower respiratory infections	2·2 (-3·3–9·7)	0·91 (-0·99–3·8)	0·24 (>-0.1–0·57)	1·1 (-2·3–5·7)
Spain	Malaria	0 (0–0)	0 (0–0)	--	--
Spain	Measles	1·8 (-0·57–4·7)	--	1·7 (-0·54–4·4)	<0.1 (>-0.1–0·31)
Sweden	All causes	0·19 (>-0.1–0·58)	<0.1 (>-0.1–0·25)	<0.1 (>-0.1–0·16)	<0.1 (>-0.1–0·20)
Sweden	Diarrheal diseases	4·4 (-2·4–12·6)	2·1 (-0·41–5·4)	1·2 (-0·68–3·3)	1·3 (-1·3–4·5)
Sweden	Lower respiratory infections	2·0 (-3·0–8·9)	0·82 (-0·91–3·4)	0·24 (>-0.1–0·58)	1·0 (-2·0–5·2)
Sweden	Malaria	0 (0–0)	0 (0–0)	--	--
Sweden	Measles	1·5 (-0·52–4·2)	--	1·4 (-0·49–4·0)	<0.1 (>-0.1–0·28)
Switzerland	All causes	0·28 (-0·13–0·81)	0·13 (>-0.1–0·35)	<0.1 (>-0.1–0·22)	<0.1 (>-0.1–0·29)
Switzerland	Diarrheal diseases	4·5 (-2·5–12·9)	2·1 (-0·42–5·5)	1·2 (-0·71–3·4)	1·4 (-1·3–4·6)
Switzerland	Lower respiratory infections	2·1 (-3·2–8·3)	0·82 (-0·97–3·1)	0·25 (>-0.1–0·57)	1·0 (-2·2–4·8)

Switzerland	Malaria	0 (0–0)	0 (0–0)	--	--
Switzerland	Measles	1·7 (-0·56–4·7)	--	1·6 (-0·53–4·4)	<0.1 (>-0.1–0·30)
United Kingdom	All causes	0·17 (>-0.1–0·47)	<0.1 (>-0.1–0·23)	<0.1 (>-0.1–0·12)	<0.1 (>-0.1–0·18)
United Kingdom	Diarrheal diseases	5·4 (-3·0–15·6)	2·6 (-0·55–7·1)	1·1 (-0·66–3·2)	1·8 (-1·8–6·3)
United Kingdom	Lower respiratory infections	2·4 (-3·9–10·1)	0·95 (-1·1–3·7)	0·22 (>-0.1–0·52)	1·3 (-2·7–6·1)
United Kingdom	Malaria	0 (0–0)	0 (0–0)	--	--
United Kingdom	Measles	1·6 (-0·51–4·2)	--	1·5 (-0·49–4·1)	<0.1 (>-0.1–0·19)
**Latin America and Caribbean**	**All causes**	**0·43 (<0.1–0·85)**	**0·34 (0·16–0·57)**	**0·23 (0·17–0·31)**	**<0.1 (>-0.1–0·28)**
**Latin America and Caribbean**	**Diarrheal diseases**	**13·8 (-9·2–36·3)**	**7·9 (-1·8–19·5)**	**1·8 (-1·1–5·2)**	**5·3 (-6·0–17·1)**
**Latin America and Caribbean**	**Lower respiratory infections**	**7·0 (-14·5–29·4)**	**3·2 (-4·1–12·4)**	**0·44 (>-0.1–1·0)**	**3·9 (-9·8–19·0)**
**Latin America and Caribbean**	**Malaria**	**15·7 (-8·0–47·4)**	**15·7 (-8·0–47·4)**	**--**	**--**
**Latin America and Caribbean**	**Measles**	**3·8 (-1·4–10·4)**	**--**	**2·9 (-0·97–7·7)**	**0·92 (-0·43–3·4)**
Andean Latin America	All causes	0·34 (-0·15–0·92)	0·22 (<0.1–0·50)	<0.1 (<0.1–0·13)	0·12 (-0·14–0·43)
Andean Latin America	Diarrheal diseases	13·7 (-9·2–37·0)	7·7 (-1·7–19·3)	1·3 (-0·78–3·7)	5·8 (-6·5–19·1)
Andean Latin America	Lower respiratory infections	7·5 (-16·4–32·2)	3·2 (-4·0–12·4)	0·29 (>-0.1–0·68)	4·5 (-11·8–22·1)
Andean Latin America	Malaria	15·0 (-7·6–47·5)	15·0 (-7·6–47·5)	--	--
Andean Latin America	Measles	3·0 (-0·39–12·0)	--	2·2 (-0·19–8·3)	0·83 (-0·24–4·0)
Bolivia (Plurinational State of)	All causes	0·23 (>-0.1–0·60)	0·16 (<0.1–0·35)	<0.1 (<0.1–0·14)	<0.1 (>-0.1–0·27)
Bolivia (Plurinational State of)	Diarrheal diseases	14·4 (-10·2–39·2)	7·7 (-1·7–19·5)	1·6 (-0·99–4·5)	6·4 (-7·2–21·0)
Bolivia (Plurinational State of)	Lower respiratory infections	7·9 (-18·2–34·2)	3·2 (-4·0–12·7)	0·37 (>-0.1–0·87)	5·0 (-13·5–24·1)
Bolivia (Plurinational State of)	Malaria	15·3 (-7·9–48·7)	15·3 (-7·9–48·7)	--	--
Bolivia (Plurinational State of)	Measles	0 (0–0)	--	0 (0–0)	0 (0–0)
Ecuador	All causes	0·48 (>-0.1–1·1)	0·34 (<0.1–0·70)	0·19 (<0.1–0·32)	0·13 (-0·15–0·44)
Ecuador	Diarrheal diseases	14·8 (-9·6–39·2)	8·5 (-1·9–21·2)	2·1 (-1·3–5·9)	5·6 (-6·2–18·1)
Ecuador	Lower respiratory infections	7·8 (-16·2–32·9)	3·6 (-4·6–13·8)	0·46 (>-0.1–1·1)	4·4 (-11·0–21·4)
Ecuador	Malaria	16·5 (-8·9–50·3)	16·5 (-8·9–50·3)	--	--
Ecuador	Measles	3·0 (-0·39–12·0)	--	2·2 (-0·19–8·3)	0·83 (-0·24–4·0)
Peru	All causes	0·33 (-0·22–0·97)	0·19 (>-0.1–0·50)	<0.1 (>-0.1–<0.1)	0·14 (-0·16–0·51)
Peru	Diarrheal diseases	13·2 (-8·9–35·9)	7·4 (-1·6–18·6)	0·94 (-0·56–2·8)	5·8 (-6·4–19·1)

Peru	Lower respiratory infections	7·1 (-15·6–30·8)	3·0 (-3·8–11·6)	0·18 (>-0.1–0·41)	4·4 (-11·2–21·4)
Peru	Malaria	14·7 (-7·3–46·6)	14·7 (-7·3–46·6)	--	--
Peru	Measles	0 (0–0)	--	0 (0–0)	0 (0–0)
Caribbean	All causes	1·5 (0·90–2·0)	1·4 (0·98–1·8)	1·2 (0·90–1·6)	<0.1 (>-0.1–0·28)
Caribbean	Diarrheal diseases	16·0 (-9·9–40·3)	9·9 (-2·4–23·7)	3·1 (-2·0–8·9)	4·7 (-5·2–15·3)
Caribbean	Lower respiratory infections	9·2 (-18·3–36·5)	5·1 (-6·8–19·4)	0·95 (-0·19–2·2)	4·2 (-10·4–20·1)
Caribbean	Malaria	22·1 (-14·2–61·3)	22·1 (-14·2–61·3)	--	--
Caribbean	Measles	3·7 (-1·2–9·9)	--	3·4 (-1·1–9·0)	0·29 (-0·13–1·0)
Antigua and Barbuda	All causes	0·62 (0·28–1·0)	0·55 (0·29–0·84)	0·50 (0·26–0·80)	<0.1 (>-0.1–0·16)
Antigua and Barbuda	Diarrheal diseases	12·2 (-7·0–32·6)	6·6 (-1·4–16·7)	3·3 (-2·1–9·5)	3·2 (-3·3–10·6)
Antigua and Barbuda	Lower respiratory infections	5·8 (-9·3–22·8)	2·7 (-3·4–10·5)	0·90 (-0·18–2·1)	2·5 (-5·5–12·0)
Antigua and Barbuda	Malaria	0 (0–0)	0 (0–0)	--	--
Antigua and Barbuda	Measles	0 (0–0)	--	0 (0–0)	0 (0–0)
Bahamas	All causes	0·24 (>-0.1–0·52)	0·18 (<0.1–0·35)	0·13 (<0.1–0·25)	<0.1 (>-0.1–0·14)
Bahamas	Diarrheal diseases	12·2 (-7·0–32·7)	6·9 (-1·5–17·5)	2·6 (-1·6–7·6)	3·6 (-3·7–11·7)
Bahamas	Lower respiratory infections	5·8 (-9·9–23·6)	2·8 (-3·5–10·9)	0·64 (-0·12–1·5)	2·7 (-6·1–13·0)
Bahamas	Malaria	0 (0–0)	0 (0–0)	--	--
Bahamas	Measles	0 (0–0)	--	0 (0–0)	0 (0–0)
Barbados	All causes	0·63 (0·26–1·2)	0·53 (0·27–0·90)	0·46 (0·23–0·83)	<0.1 (>-0.1–0·24)
Barbados	Diarrheal diseases	11·1 (-6·4–30·1)	6·0 (-1·3–15·2)	2·8 (-1·8–8·2)	3·1 (-3·2–10·2)
Barbados	Lower respiratory infections	5·4 (-8·7–21·4)	2·4 (-2·9–9·4)	0·73 (-0·14–1·7)	2·5 (-5·5–11·8)
Barbados	Malaria	0 (0–0)	0 (0–0)	--	--
Barbados	Measles	0 (0–0)	--	0 (0–0)	0 (0–0)
Belize	All causes	0·65 (0·28–1·2)	0·56 (0·32–0·93)	0·46 (0·28–0·77)	<0.1 (>-0.1–0·29)
Belize	Diarrheal diseases	15·3 (-9·9–40·5)	8·9 (-2·0–22·3)	2·1 (-1·3–6·1)	5·7 (-6·4–18·7)
Belize	Lower respiratory infections	7·9 (-16·2–33·2)	3·7 (-4·7–14·3)	0·48 (>-0.1–1·1)	4·4 (-10·9–21·3)
Belize	Malaria	0 (0–0)	0 (0–0)	--	--
Belize	Measles	0 (0–0)	--	0 (0–0)	0 (0–0)
Bermuda	All causes	0·32 (-0·12–0·89)	0·17 (>-0.1–0·43)	0·12 (>-0.1–0·30)	<0.1 (>-0.1–0·27)

Bermuda	Diarrheal diseases	8·7 (-4·8–24·0)	4·4 (-0·92–11·5)	2·4 (-1·5–6·9)	2·4 (-2·4–7·9)
Bermuda	Lower respiratory infections	4·0 (-6·3–16·5)	1·8 (-2·1–7·2)	0·53 (-0·10–1·2)	1·9 (-4·0–9·0)
Bermuda	Malaria	0 (0–0)	0 (0–0)	--	--
Bermuda	Measles	3·7 (-1·2–9·9)	--	3·4 (-1·1–9·0)	0·29 (-0·13–1·0)
Cuba	All causes	0·24 (-0·11–0·65)	0·14 (>-0.1–0·33)	<0.1 (>-0.1–0·14)	<0.1 (>-0.1–0·27)
Cuba	Diarrheal diseases	9·8 (-5·8–27·1)	5·2 (-1·1–13·5)	1·8 (-1·1–5·2)	3·4 (-3·5–11·3)
Cuba	Lower respiratory infections	4·9 (-8·8–20·4)	2·1 (-2·6–8·2)	0·41 (>-0.1–0·92)	2·6 (-6·0–12·5)
Cuba	Malaria	0 (0–0)	0 (0–0)	--	--
Cuba	Measles	0 (0–0)	--	0 (0–0)	0 (0–0)
Dominica	All causes	0·25 (>-0.1–0·55)	0·19 (<0.1–0·36)	0·13 (<0.1–0·24)	<0.1 (>-0.1–0·15)
Dominica	Diarrheal diseases	12·0 (-6·8–32·2)	6·8 (-1·5–17·3)	2·6 (-1·6–7·5)	3·4 (-3·6–11·4)
Dominica	Lower respiratory infections	5·8 (-10·0–23·3)	2·8 (-3·5–10·9)	0·62 (-0·12–1·4)	2·7 (-6·1–12·8)
Dominica	Malaria	0 (0–0)	0 (0–0)	--	--
Dominica	Measles	0 (0–0)	--	0 (0–0)	0 (0–0)
Dominican Republic	All causes	0·24 (>-0.1–0·62)	0·16 (>-0.1–0·35)	<0.1 (<0.1–0·15)	<0.1 (>-0.1–0·22)
Dominican Republic	Diarrheal diseases	11·5 (-6·5–31·0)	6·7 (-1·5–17·2)	1·9 (-1·2–5·6)	3·6 (-3·7–11·9)
Dominican Republic	Lower respiratory infections	5·7 (-10·2–23·9)	2·8 (-3·5–11·0)	0·44 (>-0.1–1·0)	2·8 (-6·4–13·6)
Dominican Republic	Malaria	13·9 (-6·7–45·4)	13·9 (-6·7–45·4)	--	--
Dominican Republic	Measles	0 (0–0)	--	0 (0–0)	0 (0–0)
Grenada	All causes	0·37 (<0.1–0·78)	0·29 (0·10–0·55)	0·20 (<0.1–0·39)	<0.1 (>-0.1–0·22)
Grenada	Diarrheal diseases	13·5 (-7·7–35·7)	7·9 (-1·8–19·8)	2·7 (-1·7–7·9)	3·9 (-4·1–12·9)
Grenada	Lower respiratory infections	6·4 (-11·1–26·1)	3·2 (-4·1–12·6)	0·67 (-0·13–1·5)	2·9 (-6·7–14·3)
Grenada	Malaria	0 (0–0)	0 (0–0)	--	--
Grenada	Measles	0 (0–0)	--	0 (0–0)	0 (0–0)
Guyana	All causes	1·7 (1·0–2·4)	1·6 (1·0–2·3)	1·4 (0·98–2·0)	<0.1 (>-0.1–0·11)
Guyana	Diarrheal diseases	17·4 (-10·2–43·6)	10·7 (-2·5–25·8)	4·3 (-2·9–12·3)	4·2 (-4·5–13·7)
Guyana	Lower respiratory infections	8·6 (-15·0–33·1)	4·8 (-6·2–18·2)	1·2 (-0·24–2·8)	3·4 (-8·0–16·0)
Guyana	Malaria	19·7 (-11·6–57·6)	19·7 (-11·6–57·6)	--	--
Guyana	Measles	0 (0–0)	--	0 (0–0)	0 (0–0)

Haiti	All causes	2·4 (1·6–3·3)	2·3 (1·7–3·0)	2·1 (1·5–2·8)	<0.1 (-0·12–0·34)
Haiti	Diarrheal diseases	20·9 (-13·4–50·9)	13·4 (-3·4–31·5)	4·2 (-2·7–11·7)	6·0 (-6·8–19·3)
Haiti	Lower respiratory infections	11·1 (-22·8–42·9)	6·4 (-8·7–23·8)	1·2 (-0·23–2·8)	5·0 (-12·7–23·4)
Haiti	Malaria	23·7 (-16·3–65·3)	23·7 (-16·3–65·3)	--	--
Haiti	Measles	0 (0–0)	--	0 (0–0)	0 (0–0)
Jamaica	All causes	0·17 (<0.1–0·41)	0·13 (<0.1–0·25)	<0.1 (<0.1–0·17)	<0.1 (>-0.1–0·12)
Jamaica	Diarrheal diseases	9·7 (-5·4–26·7)	5·1 (-1·1–13·2)	2·4 (-1·5–7·1)	2·7 (-2·8–9·2)
Jamaica	Lower respiratory infections	4·7 (-7·7–19·5)	2·1 (-2·5–8·4)	0·55 (-0·11–1·3)	2·2 (-4·9–11·1)
Jamaica	Malaria	0 (0–0)	0 (0–0)	--	--
Jamaica	Measles	0 (0–0)	--	0 (0–0)	0 (0–0)
Puerto Rico	All causes	0·26 (-0·11–0·70)	0·14 (>-0.1–0·34)	<0.1 (>-0.1–0·21)	<0.1 (>-0.1–0·23)
Puerto Rico	Diarrheal diseases	8·4 (-4·7–23·4)	4·2 (-0·89–11·1)	2·3 (-1·4–6·6)	2·3 (-2·3–7·8)
Puerto Rico	Lower respiratory infections	4·0 (-6·3–16·5)	1·8 (-2·1–7·0)	0·52 (-0·10–1·2)	1·9 (-4·0–9·1)
Puerto Rico	Malaria	0 (0–0)	0 (0–0)	--	--
Puerto Rico	Measles	2·3 (-0·90–9·1)	--	2·1 (-0·79–8·3)	0·19 (>-0.1–0·96)
Saint Kitts and Nevis	All causes	0·28 (>-0.1–0·68)	0·19 (<0.1–0·42)	0·13 (<0.1–0·25)	<0.1 (>-0.1–0·19)
Saint Kitts and Nevis	Diarrheal diseases	11·8 (-6·6–31·7)	6·7 (-1·5–17·1)	2·7 (-1·7–7·8)	3·3 (-3·4–10·8)
Saint Kitts and Nevis	Lower respiratory infections	5·8 (-9·7–23·5)	2·8 (-3·5–11·0)	0·63 (-0·13–1·5)	2·6 (-5·9–12·8)
Saint Kitts and Nevis	Malaria	0 (0–0)	0 (0–0)	--	--
Saint Kitts and Nevis	Measles	0 (0–0)	--	0 (0–0)	0 (0–0)
Saint Lucia	All causes	0·45 (0·13–0·91)	0·37 (0·14–0·75)	0·31 (0·11–0·66)	<0.1 (>-0.1–0·13)
Saint Lucia	Diarrheal diseases	11·0 (-5·6–29·2)	6·5 (-1·4–16·5)	3·0 (-1·9–8·6)	2·2 (-2·2–7·4)
Saint Lucia	Lower respiratory infections	4·9 (-7·3–19·3)	2·7 (-3·4–10·6)	0·74 (-0·15–1·7)	1·7 (-3·7–8·3)
Saint Lucia	Malaria	0 (0–0)	0 (0–0)	--	--
Saint Lucia	Measles	0 (0–0)	--	0 (0–0)	0 (0–0)
Saint Vincent and the Grenadines	All causes	0·26 (<0.1–0·53)	0·21 (<0.1–0·38)	0·16 (<0.1–0·29)	<0.1 (>-0.1–0·13)
Saint Vincent and the Grenadines	Diarrheal diseases	12·6 (-7·2–33·5)	7·3 (-1·6–18·5)	2·6 (-1·6–7·6)	3·6 (-3·8–11·9)
Saint Vincent and the Grenadines	Lower respiratory infections	6·2 (-10·5–25·0)	3·1 (-3·8–11·9)	0·62 (-0·12–1·4)	2·9 (-6·4–13·7)
Saint Vincent and the Grenadines	Malaria	0 (0–0)	0 (0–0)	--	--

Saint Vincent and the Grenadines	Measles	0 (0–0)	--	0 (0–0)	0 (0–0)
Suriname	All causes	1·7 (1·0–2·5)	1·7 (1·0–2·4)	1·6 (1·0–2·2)	<0.1 (>-0.1–0·19)
Suriname	Diarrheal diseases	16·3 (-9·1–41·5)	10·1 (-2·4–24·8)	4·1 (-2·6–11·6)	3·7 (-3·9–12·4)
Suriname	Lower respiratory infections	8·1 (-13·8–31·1)	4·5 (-5·9–17·2)	1·1 (-0·21–2·4)	3·1 (-7·2–14·9)
Suriname	Malaria	20·0 (-12·0–58·6)	20·0 (-12·0–58·6)	--	--
Suriname	Measles	0 (0–0)	--	0 (0–0)	0 (0–0)
Trinidad and Tobago	All causes	2·2 (1·4–3·2)	2·1 (1·4–3·1)	2·1 (1·3–3·0)	<0.1 (>-0.1–0·13)
Trinidad and Tobago	Diarrheal diseases	13·6 (-7·8–35·6)	7·9 (-1·8–19·5)	3·4 (-2·1–9·6)	3·6 (-3·7–11·6)
Trinidad and Tobago	Lower respiratory infections	6·7 (-11·4–26·8)	3·4 (-4·3–13·3)	0·90 (-0·17–2·1)	2·9 (-6·6–14·0)
Trinidad and Tobago	Malaria	0 (0–0)	0 (0–0)	--	--
Trinidad and Tobago	Measles	0 (0–0)	--	0 (0–0)	0 (0–0)
United States Virgin Islands	All causes	0·22 (>-0.1–0·62)	0·14 (<0.1–0·33)	<0.1 (<0.1–0·18)	<0.1 (>-0.1–0·21)
United States Virgin Islands	Diarrheal diseases	10·3 (-5·9–28·3)	5·5 (-1·2–14·1)	2·4 (-1·5–6·9)	3·1 (-3·2–10·3)
United States Virgin Islands	Lower respiratory infections	5·0 (-8·3–20·5)	2·3 (-2·7–8·9)	0·55 (-0·11–1·3)	2·4 (-5·3–11·7)
United States Virgin Islands	Malaria	0 (0–0)	0 (0–0)	--	--
United States Virgin Islands	Measles	0 (0–0)	--	0 (0–0)	0 (0–0)
Central Latin America	All causes	0·42 (>-0.1–0·85)	0·31 (0·11–0·55)	0·19 (0·13–0·26)	0·10 (-0·12–0·33)
Central Latin America	Diarrheal diseases	14·7 (-10·3–38·6)	8·6 (-2·0–21·0)	1·6 (-0·99–4·8)	5·9 (-6·8–19·0)
Central Latin America	Lower respiratory infections	7·8 (-17·4–33·6)	3·6 (-4·6–14·2)	0·34 (>-0.1–0·78)	4·6 (-12·4–22·6)
Central Latin America	Malaria	16·6 (-8·6–49·9)	16·6 (-8·6–49·9)	--	--
Central Latin America	Measles	3·8 (-1·5–10·9)	--	2·7 (-0·89–7·2)	1·2 (-0·49–4·1)
Colombia	All causes	0·31 (-0·17–0·86)	0·18 (>-0.1–0·48)	<0.1 (>-0.1–0·12)	0·11 (-0·11–0·35)
Colombia	Diarrheal diseases	11·4 (-6·8–31·3)	6·5 (-1·4–16·7)	1·6 (-0·97–4·6)	4·0 (-4·3–13·5)
Colombia	Lower respiratory infections	5·7 (-10·7–24·5)	2·6 (-3·3–10·4)	0·32 (>-0.1–0·73)	3·1 (-7·1–15·1)
Colombia	Malaria	13·3 (-6·4–43·7)	13·3 (-6·4–43·7)	--	--
Colombia	Measles	0 (0–0)	--	0 (0–0)	0 (0–0)
Costa Rica	All causes	0·13 (>-0.1–0·40)	<0.1 (>-0.1–0·21)	<0.1 (>-0.1–<0.1)	<0.1 (>-0.1–0·14)
Costa Rica	Diarrheal diseases	7·6 (-4·2–21·6)	4·2 (-0·91–11·1)	1·6 (-1·0–4·8)	2·2 (-2·2–7·4)
Costa Rica	Lower respiratory infections	3·4 (-5·5–13·6)	1·6 (-2·0–6·0)	0·33 (>-0.1–0·74)	1·6 (-3·4–7·5)

Costa Rica	Malaria	8·4 (-3·5–28·7)	8·4 (-3·5–28·7)	--	--
Costa Rica	Measles	0 (0–0)	--	0 (0–0)	0 (0–0)
El Salvador	All causes	0·36 (-0·16–0·97)	0·24 (<0.1–0·57)	<0.1 (<0.1–0·16)	0·12 (-0·15–0·43)
El Salvador	Diarrheal diseases	15·6 (-10·2–41·5)	9·3 (-2·1–23·3)	1·7 (-1·0–4·9)	6·1 (-6·8–20·0)
El Salvador	Lower respiratory infections	7·9 (-17·1–33·6)	3·8 (-4·9–14·9)	0·36 (>-0.1–0·83)	4·4 (-11·6–21·5)
El Salvador	Malaria	0 (0–0)	0 (0–0)	--	--
El Salvador	Measles	0 (0–0)	--	0 (0–0)	0 (0–0)
Guatemala	All causes	0·59 (-0·46–1·5)	0·39 (>-0.1–0·91)	<0.1 (>-0.1–0·12)	0·25 (-0·32–0·78)
Guatemala	Diarrheal diseases	22·9 (-17·5–55·9)	14·8 (-3·8–34·5)	1·5 (-0·92–4·6)	9·6 (-11·8–29·8)
Guatemala	Lower respiratory infections	12·3 (-34·6–52·0)	6·5 (-9·0–24·5)	0·29 (>-0.1–0·67)	7·4 (-23·3–36·4)
Guatemala	Malaria	24·6 (-17·4–67·9)	24·6 (-17·4–67·9)	--	--
Guatemala	Measles	0 (0–0)	--	0 (0–0)	0 (0–0)
Honduras	All causes	0·22 (-0·13–0·58)	0·14 (>-0.1–0·35)	<0.1 (>-0.1–<0.1)	<0.1 (>-0.1–0·25)
Honduras	Diarrheal diseases	18·2 (-12·2–46·4)	11·4 (-2·7–27·6)	1·9 (-1·2–5·6)	6·8 (-7·9–21·9)
Honduras	Lower respiratory infections	9·4 (-21·5–38·8)	4·8 (-6·5–18·1)	0·39 (>-0.1–0·92)	5·1 (-14·0–24·7)
Honduras	Malaria	20·5 (-12·6–58·3)	20·5 (-12·6–58·3)	--	--
Honduras	Measles	0 (0–0)	--	0 (0–0)	0 (0–0)
Mexico	All causes	0·43 (0·20–0·68)	0·37 (0·25–0·52)	0·31 (0·23–0·41)	<0.1 (>-0.1–0·18)
Mexico	Diarrheal diseases	13·5 (-9·6–36·1)	7·3 (-1·7–18·0)	1·6 (-0·98–4·7)	5·8 (-6·7–18·8)
Mexico	Lower respiratory infections	7·1 (-15·5–30·0)	2·9 (-3·6–11·1)	0·36 (>-0.1–0·82)	4·4 (-11·4–21·0)
Mexico	Malaria	17·5 (-9·9–49·0)	17·5 (-9·9–49·0)	--	--
Mexico	Measles	0 (0–0)	--	0 (0–0)	0 (0–0)
Nicaragua	All causes	0·28 (-0·17–0·77)	0·17 (>-0.1–0·43)	<0.1 (>-0.1–<0.1)	0·10 (-0·11–0·35)
Nicaragua	Diarrheal diseases	14·1 (-9·2–37·7)	8·1 (-1·8–20·4)	1·7 (-1·0–4·8)	5·5 (-6·1–17·9)
Nicaragua	Lower respiratory infections	7·3 (-15·8–31·0)	3·4 (-4·3–12·9)	0·35 (>-0.1–0·83)	4·2 (-11·0–20·3)
Nicaragua	Malaria	16·3 (-8·6–52·0)	16·3 (-8·6–52·0)	--	--
Nicaragua	Measles	0 (0–0)	--	0 (0–0)	0 (0–0)
Panama	All causes	0·66 (-0·43–1·9)	0·36 (>-0.1–0·94)	<0.1 (>-0.1–0·20)	0·28 (-0·32–0·96)
Panama	Diarrheal diseases	13·0 (-8·8–35·4)	6·9 (-1·5–17·6)	1·3 (-0·80–3·9)	5·7 (-6·3–18·5)

Panama	Lower respiratory infections	7·0 (-15·1–30·1)	2·8 (-3·5–11·1)	0·28 (>-0.1–0·64)	4·3 (-11·1–20·9)
Panama	Malaria	14·1 (-7·0–46·0)	14·1 (-7·0–46·0)	--	--
Panama	Measles	0 (0–0)	--	0 (0–0)	0 (0–0)
Venezuela (Bolivarian Republic of)	All causes	0·65 (-0·20–1·5)	0·45 (<0.1–0·99)	0·21 (<0.1–0·53)	0·19 (-0·22–0·61)
Venezuela (Bolivarian Republic of)	Diarrheal diseases	15·9 (-11·1–42·1)	9·1 (-2·1–22·5)	1·8 (-1·1–5·3)	6·6 (-7·5–21·1)
Venezuela (Bolivarian Republic of)	Lower respiratory infections	8·3 (-18·6–35·2)	3·7 (-4·7–14·1)	0·40 (>-0.1–0·92)	5·0 (-13·5–24·0)
Venezuela (Bolivarian Republic of)	Malaria	17·6 (-9·6–53·5)	17·6 (-9·6–53·5)	--	--
Venezuela (Bolivarian Republic of)	Measles	3·8 (-1·5–10·9)	--	2·7 (-0·89–7·2)	1·2 (-0·49–4·1)
Tropical Latin America	All causes	0·17 (>-0.1–0·44)	0·12 (<0.1–0·25)	<0.1 (<0.1–0·11)	<0.1 (>-0.1–0·17)
Tropical Latin America	Diarrheal diseases	11·2 (-6·8–30·2)	6·1 (-1·3–15·3)	1·8 (-1·1–5·4)	4·0 (-4·3–13·1)
Tropical Latin America	Lower respiratory infections	5·9 (-11·6–25·0)	2·6 (-3·2–10·1)	0·42 (>-0.1–0·96)	3·3 (-8·0–15·9)
Tropical Latin America	Malaria	13·3 (-6·4–43·0)	13·3 (-6·4–43·0)	--	--
Tropical Latin America	Measles	0 (0–0)	--	0 (0–0)	0 (0–0)
Brazil	All causes	0·17 (>-0.1–0·44)	0·11 (<0.1–0·25)	<0.1 (<0.1–0·11)	<0.1 (>-0.1–0·17)
Brazil	Diarrheal diseases	11·4 (-6·9–30·8)	6·2 (-1·3–15·7)	1·9 (-1·2–5·5)	4·1 (-4·4–13·3)
Brazil	Lower respiratory infections	6·0 (-11·7–25·2)	2·6 (-3·2–10·2)	0·43 (>-0.1–0·97)	3·3 (-8·1–16·0)
Brazil	Malaria	13·3 (-6·4–43·0)	13·3 (-6·4–43·0)	--	--
Brazil	Measles	0 (0–0)	--	0 (0–0)	0 (0–0)
Paraguay	All causes	0·22 (<0.1–0·45)	0·16 (<0.1–0·28)	0·12 (<0.1–0·18)	<0.1 (>-0.1–0·17)
Paraguay	Diarrheal diseases	7·2 (-4·4–20·3)	3·7 (-0·77–9·3)	0·91 (-0·54–2·6)	3·0 (-3·0–9·9)
Paraguay	Lower respiratory infections	3·6 (-6·8–16·1)	1·3 (-1·5–5·1)	0·15 (>-0.1–0·34)	2·3 (-5·2–11·3)
Paraguay	Malaria	0 (0–0)	0 (0–0)	--	--
Paraguay	Measles	0 (0–0)	--	0 (0–0)	0 (0–0)
**North Africa and Middle East**	**All causes**	**3·0 (2·0–4·1)**	**2·8 (2·1–3·7)**	**2·6 (2·0–3·3)**	**0·14 (-0·16–0·47)**
**North Africa and Middle East**	**Diarrheal diseases**	**17·3 (-11·7–43·0)**	**10·2 (-2·6–24·1)**	**4·0 (-2·6–11·0)**	**5·4 (-6·1–17·4)**
**North Africa and Middle East**	**Lower respiratory infections**	**10·6 (-24·4–41·0)**	**5·8 (-8·8–21·6)**	**1·5 (-0·28–3·4)**	**5·0 (-13·5–24·0)**
**North Africa and Middle East**	**Malaria**	**32·0 (-34·8–76·6)**	**32·0 (-34·8–76·6)**	**--**	**--**
**North Africa and Middle East**	**Measles**	**7·8 (-3·1–20·7)**	**--**	**5·9 (-2·1–15·1)**	**2·2 (-1·1–7·4)**
North Africa and Middle East	All causes	3·0 (2·0–4·1)	2·8 (2·1–3·7)	2·6 (2·0–3·3)	0·14 (-0·16–0·47)

North Africa and Middle East	Diarrheal diseases	17·3 (-11·7–43·0)	10·2 (-2·6–24·1)	4·0 (-2·6–11·0)	5·4 (-6·1–17·4)
North Africa and Middle East	Lower respiratory infections	10·6 (-24·4–41·0)	5·8 (-8·8–21·6)	1·5 (-0·28–3·4)	5·0 (-13·5–24·0)
North Africa and Middle East	Malaria	32·0 (-34·8–76·6)	32·0 (-34·8–76·6)	--	--
North Africa and Middle East	Measles	7·8 (-3·1–20·7)	--	5·9 (-2·1–15·1)	2·2 (-1·1–7·4)
Afghanistan	All causes	2·8 (1·5–4·3)	2·6 (1·7–3·8)	2·2 (1·6–3·2)	0·21 (-0·26–0·73)
Afghanistan	Diarrheal diseases	24·8 (-17·0–58·3)	16·5 (-4·3–37·4)	4·8 (-3·1–13·5)	7·4 (-8·7–23·4)
Afghanistan	Lower respiratory infections	14·7 (-33·6–55·2)	8·9 (-12·6–32·2)	1·8 (-0·34–4·2)	6·6 (-18·2–31·3)
Afghanistan	Malaria	26·9 (-22·1–69·7)	26·9 (-22·1–69·7)	--	--
Afghanistan	Measles	11·9 (-4·9–32·6)	--	7·7 (-2·6–19·7)	4·9 (-2·5–15·9)
Algeria	All causes	1·7 (0·95–2·7)	1·5 (1·0–2·1)	1·4 (0·97–2·0)	0·14 (-0·14–0·47)
Algeria	Diarrheal diseases	13·5 (-8·4–35·9)	7·2 (-1·6–18·1)	2·9 (-1·8–8·3)	4·5 (-4·8–14·8)
Algeria	Lower respiratory infections	7·2 (-13·1–28·8)	3·1 (-3·8–12·0)	0·91 (-0·17–2·2)	3·7 (-8·7–17·4)
Algeria	Malaria	0 (0–0)	0 (0–0)	--	--
Algeria	Measles	5·2 (-1·8–14·3)	--	4·2 (-1·4–11·4)	1·0 (-0·45–3·6)
Bahrain	All causes	0·42 (>-0.1–1·0)	0·28 (<0.1–0·61)	0·19 (<0.1–0·43)	<0.1 (>-0.1–0·32)
Bahrain	Diarrheal diseases	12·6 (-7·5–34·0)	6·8 (-1·5–17·3)	3·2 (-2·0–9·2)	3·6 (-3·8–12·1)
Bahrain	Lower respiratory infections	6·2 (-10·5–24·9)	2·8 (-3·5–11·1)	0·90 (-0·18–2·1)	2·8 (-6·5–13·8)
Bahrain	Malaria	0 (0–0)	0 (0–0)	--	--
Bahrain	Measles	4·9 (-1·8–13·9)	--	4·5 (-1·6–12·6)	0·42 (-0·19–1·5)
Egypt	All causes	2·1 (1·3–3·3)	1·9 (1·4–2·6)	1·7 (1·3–2·4)	0·17 (-0·21–0·59)
Egypt	Diarrheal diseases	14·6 (-9·7–38·7)	7·4 (-1·7–18·8)	3·5 (-2·2–9·7)	5·1 (-5·6–16·6)
Egypt	Lower respiratory infections	8·1 (-15·1–32·9)	3·2 (-4·0–12·6)	1·2 (-0·22–2·7)	4·4 (-10·5–21·3)
Egypt	Malaria	0 (0–0)	0 (0–0)	--	--
Egypt	Measles	6·4 (-2·3–17·9)	--	4·9 (-1·6–12·8)	1·7 (-0·83–5·9)
Iran (Islamic Republic of)	All causes	1·1 (0·71–1·7)	1·1 (0·74–1·5)	1·0 (0·70–1·4)	<0.1 (>-0.1–0·16)
Iran (Islamic Republic of)	Diarrheal diseases	13·2 (-7·7–35·0)	7·4 (-1·7–18·8)	3·4 (-2·1–9·8)	3·5 (-3·7–11·6)
Iran (Islamic Republic of)	Lower respiratory infections	6·2 (-11·0–25·0)	3·1 (-4·1–12·1)	0·86 (-0·17–2·0)	2·7 (-6·4–13·2)
Iran (Islamic Republic of)	Malaria	17·1 (-8·6–51·7)	17·1 (-8·6–51·7)	--	--
Iran (Islamic Republic of)	Measles	5·7 (-2·1–15·3)	--	5·1 (-1·8–13·4)	0·64 (-0·31–2·2)

Iraq	All causes	2·2 (1·2–3·6)	1·9 (1·2–2·6)	1·7 (1·2–2·3)	0·23 (-0·25–0·79)
Iraq	Diarrheal diseases	15·7 (-10·2–40·8)	8·6 (-1·9–21·2)	3·5 (-2·2–9·8)	5·2 (-5·7–16·8)
Iraq	Lower respiratory infections	8·4 (-16·1–33·6)	3·7 (-4·7–14·2)	1·1 (-0·21–2·6)	4·4 (-10·7–20·8)
Iraq	Malaria	0 (0–0)	0 (0–0)	--	--
Iraq	Measles	6·4 (-2·3–17·0)	--	5·1 (-1·7–12·9)	1·5 (-0·61–5·1)
Jordan	All causes	0·41 (-0·19–1·2)	0·24 (>-0.1–0·61)	0·11 (<0.1–0·27)	0·13 (-0·14–0·49)
Jordan	Diarrheal diseases	11·6 (-7·2–32·0)	6·2 (-1·4–16·0)	2·2 (-1·4–6·5)	4·0 (-4·3–13·6)
Jordan	Lower respiratory infections	5·8 (-10·7–24·5)	2·5 (-3·1–9·7)	0·51 (-0·10–1·2)	3·1 (-7·3–15·4)
Jordan	Malaria	0 (0–0)	0 (0–0)	--	--
Jordan	Measles	3·6 (-1·3–9·9)	--	3·1 (-1·0–8·3)	0·50 (-0·23–1·8)
Kuwait	All causes	0·30 (-0·15–0·88)	0·16 (>-0.1–0·42)	<0.1 (>-0.1–0·24)	<0.1 (>-0.1–0·31)
Kuwait	Diarrheal diseases	8·4 (-4·7–23·2)	4·3 (-0·90–11·0)	2·0 (-1·2–5·9)	2·5 (-2·5–8·3)
Kuwait	Lower respiratory infections	3·9 (-6·4–16·3)	1·7 (-2·1–6·8)	0·43 (>-0.1–0·97)	1·9 (-4·2–9·4)
Kuwait	Malaria	0 (0–0)	0 (0–0)	--	--
Kuwait	Measles	3·0 (-1·0–8·1)	--	2·8 (-0·93–7·4)	0·23 (-0·10–0·79)
Lebanon	All causes	1·2 (<0.1–2·6)	0·85 (0·35–1·6)	0·70 (0·32–1·3)	0·26 (-0·26–0·92)
Lebanon	Diarrheal diseases	12·6 (-8·5–34·4)	6·1 (-1·3–15·5)	2·7 (-1·7–7·9)	4·9 (-5·3–16·0)
Lebanon	Lower respiratory infections	7·0 (-13·7–29·2)	2·6 (-3·2–10·1)	0·88 (-0·17–2·0)	4·1 (-10·0–19·6)
Lebanon	Malaria	0 (0–0)	0 (0–0)	--	--
Lebanon	Measles	5·0 (-1·9–13·8)	--	3·9 (-1·3–10·4)	1·2 (-0·52–4·0)
Libya	All causes	1·9 (1·2–2·9)	1·7 (1·1–2·5)	1·6 (1·1–2·3)	0·11 (-0·12–0·40)
Libya	Diarrheal diseases	16·2 (-10·8–42·6)	8·8 (-2·0–21·9)	3·7 (-2·3–10·6)	5·5 (-6·1–18·0)
Libya	Lower respiratory infections	8·6 (-16·7–34·9)	3·7 (-4·7–14·2)	1·1 (-0·22–2·6)	4·5 (-11·2–22·2)
Libya	Malaria	0 (0–0)	0 (0–0)	--	--
Libya	Measles	7·1 (-2·7–19·9)	--	5·5 (-1·8–14·2)	1·8 (-0·91–6·5)
Morocco	All causes	0·47 (<0.1–0·98)	0·34 (0·17–0·59)	0·28 (0·16–0·45)	<0.1 (>-0.1–0·32)
Morocco	Diarrheal diseases	11·7 (-7·7–32·3)	5·9 (-1·3–15·1)	2·4 (-1·5–6·9)	4·4 (-4·8–14·5)
Morocco	Lower respiratory infections	6·4 (-12·2–27·3)	2·5 (-3·0–9·9)	0·66 (-0·13–1·6)	3·7 (-8·8–18·1)
Morocco	Malaria	0 (0–0)	0 (0–0)	--	--

Morocco	Measles	4·3 (-1·6–12·2)	--	3·3 (-1·1–8·7)	1·1 (-0·50–3·9)
Oman	All causes	2·7 (1·6–3·9)	2·5 (1·7–3·6)	2·4 (1·6–3·4)	<0.1 (>-0.1–0·29)
Oman	Diarrheal diseases	18·4 (-10·4–45·8)	11·6 (-2·8–28·0)	5·1 (-3·4–14·5)	3·8 (-3·9–12·5)
Oman	Lower respiratory infections	9·3 (-15·9–35·1)	5·4 (-7·4–20·4)	1·4 (-0·28–3·4)	3·2 (-7·6–15·8)
Oman	Malaria	21·9 (-14·3–61·2)	21·9 (-14·3–61·2)	--	--
Oman	Measles	8·1 (-3·0–21·4)	--	7·3 (-2·6–18·7)	0·94 (-0·47–3·3)
Palestine	All causes	0·24 (-0·12–0·68)	0·13 (<0.1–0·31)	<0.1 (<0.1–0·15)	<0.1 (>-0.1–0·31)
Palestine	Diarrheal diseases	9·9 (-6·3–27·6)	4·8 (-1·0–12·3)	1·7 (-1·0–5·0)	4·0 (-4·2–13·1)
Palestine	Lower respiratory infections	5·1 (-9·4–21·3)	1·9 (-2·3–7·3)	0·41 (>-0.1–0·95)	3·0 (-6·9–14·3)
Palestine	Malaria	0 (0–0)	0 (0–0)	--	--
Palestine	Measles	3·0 (-1·0–8·3)	--	2·6 (-0·84–6·9)	0·47 (-0·20–1·7)
Qatar	All causes	0·24 (-0·12–0·70)	0·12 (>-0.1–0·31)	<0.1 (>-0.1–0·19)	<0.1 (>-0.1–0·27)
Qatar	Diarrheal diseases	8·8 (-5·2–24·8)	4·2 (-0·88–11·1)	2·3 (-1·4–6·5)	2·8 (-2·9–9·6)
Qatar	Lower respiratory infections	4·3 (-7·2–17·6)	1·7 (-2·0–6·7)	0·52 (-0·10–1·2)	2·2 (-4·9–10·7)
Qatar	Malaria	0 (0–0)	0 (0–0)	--	--
Qatar	Measles	3·6 (-1·2–9·8)	--	3·3 (-1·1–8·8)	0·30 (-0·13–1·1)
Saudi Arabia	All causes	2·8 (1·7–4·2)	2·6 (1·8–3·8)	2·5 (1·7–3·6)	0·12 (-0·12–0·44)
Saudi Arabia	Diarrheal diseases	13·8 (-8·3–36·6)	7·2 (-1·6–18·2)	4·2 (-2·7–12·0)	3·6 (-3·8–12·1)
Saudi Arabia	Lower respiratory infections	7·0 (-10·7–27·2)	3·1 (-3·7–12·3)	1·5 (-0·29–3·5)	2·9 (-6·4–14·0)
Saudi Arabia	Malaria	15·4 (-7·7–48·6)	15·4 (-7·7–48·6)	--	--
Saudi Arabia	Measles	6·5 (-2·2–16·9)	--	6·1 (-2·0–15·6)	0·45 (-0·19–1·6)
Sudan	All causes	8·5 (5·9–12·1)	8·3 (6·1–11·6)	7·6 (5·7–9·9)	0·16 (-0·21–0·59)
Sudan	Diarrheal diseases	27·8 (-19·9–63·7)	18·8 (-5·3–42·2)	7·6 (-5·3–20·9)	6·8 (-8·1–22·5)
Sudan	Lower respiratory infections	15·6 (-38·0–56·3)	10·0 (-16·4–35·8)	2·7 (-0·52–6·2)	6·0 (-18·2–28·7)
Sudan	Malaria	30·9 (-31·0–75·6)	30·9 (-31·0–75·6)	--	--
Sudan	Measles	13·8 (-5·6–34·6)	--	11·5 (-4·3–28·2)	2·8 (-1·4–9·8)
Syrian Arab Republic	All causes	8·0 (5·9–10·6)	7·8 (5·7–10·4)	7·7 (5·6–10·3)	0·12 (-0·14–0·42)
Syrian Arab Republic	Diarrheal diseases	20·0 (-13·9–50·0)	11·4 (-2·7–27·3)	4·6 (-3·0–12·8)	6·7 (-7·7–21·3)
Syrian Arab Republic	Lower respiratory infections	11·5 (-23·1–44·6)	5·3 (-6·9–20·3)	1·9 (-0·36–4·4)	5·7 (-14·8–27·4)

Syrian Arab Republic	Malaria	0 (0–0)	0 (0–0)	--	--
Syrian Arab Republic	Measles	9·9 (-4·0–26·8)	--	7·1 (-2·4–18·4)	3·3 (-1·5–10·8)
Tunisia	All causes	0·68 (>-0.1–1·6)	0·44 (0·18–0·83)	0·34 (0·17–0·57)	0·17 (-0·18–0·65)
Tunisia	Diarrheal diseases	9·3 (-6·1–26·3)	4·2 (-0·91–11·0)	2·0 (-1·2–5·6)	3·7 (-3·8–12·3)
Tunisia	Lower respiratory infections	5·0 (-9·1–21·5)	1·7 (-2·0–6·8)	0·51 (>-0.1–1·2)	3·0 (-6·9–14·7)
Tunisia	Malaria	0 (0–0)	0 (0–0)	--	--
Tunisia	Measles	3·3 (-1·2–9·2)	--	2·6 (-0·84–7·0)	0·69 (-0·32–2·4)
Türkiye	All causes	0·23 (-0·13–0·67)	0·11 (>-0.1–0·30)	<0.1 (>-0.1–0·13)	<0.1 (>-0.1–0·31)
Türkiye	Diarrheal diseases	8·5 (-5·3–24·5)	4·2 (-0·88–11·2)	1·4 (-0·85–4·0)	3·4 (-3·5–11·4)
Türkiye	Lower respiratory infections	4·5 (-8·7–19·6)	1·7 (-2·1–6·7)	0·29 (>-0.1–0·67)	2·7 (-6·4–13·1)
Türkiye	Malaria	0 (0–0)	0 (0–0)	--	--
Türkiye	Measles	2·4 (-0·82–6·7)	--	2·0 (-0·65–5·2)	0·44 (-0·20–1·5)
United Arab Emirates	All causes	2·5 (1·5–3·8)	2·3 (1·5–3·4)	2·2 (1·5–3·2)	<0.1 (-0·10–0·36)
United Arab Emirates	Diarrheal diseases	17·6 (-10·7–44·7)	10·2 (-2·4–25·0)	5·0 (-3·3–14·3)	4·3 (-4·7–14·3)
United Arab Emirates	Lower respiratory infections	9·7 (-15·5–36·7)	5·0 (-6·3–19·5)	2·1 (-0·40–5·0)	3·5 (-8·3–17·3)
United Arab Emirates	Malaria	0 (0–0)	0 (0–0)	--	--
United Arab Emirates	Measles	8·6 (-3·1–23·0)	--	7·8 (-2·7–20·5)	0·98 (-0·44–3·4)
Yemen	All causes	5·1 (3·7–6·6)	5·0 (3·7–6·4)	4·6 (3·5–5·6)	<0.1 (-0·12–0·31)
Yemen	Diarrheal diseases	33·4 (-27·6–71·7)	23·8 (-7·5–50·3)	8·1 (-5·8–22·3)	9·3 (-11·5–29·4)
Yemen	Lower respiratory infections	18·2 (-55·1–67·2)	12·6 (-23·4–44·9)	2·6 (-0·52–5·9)	7·7 (-24·6–37·7)
Yemen	Malaria	34·4 (-42·6–80·2)	34·4 (-42·6–80·2)	--	--
Yemen	Measles	15·6 (-6·8–40·0)	--	11·8 (-4·6–28·5)	4·7 (-2·5–16·1)
**South Asia**	**All causes**	**9·1 (7·1–11·5)**	**8·8 (7·0–11·0)**	**8·3 (6·8–10·2)**	**0·26 (-0·31–0·89)**
**South Asia**	**Diarrheal diseases**	**31·3 (-24·3–68·6)**	**21·8 (-6·6–47·0)**	**8·0 (-5·8–21·9)**	**8·2 (-10·0–25·9)**
**South Asia**	**Lower respiratory infections**	**16·7 (-42·3–60·2)**	**10·8 (-18·3–38·7)**	**3·1 (-0·61–7·1)**	**6·6 (-19·3–31·6)**
**South Asia**	**Malaria**	**32·4 (-36·1–78·0)**	**32·4 (-36·1–78·0)**	**--**	**--**
**South Asia**	**Measles**	**16·2 (-7·0–40·7)**	**--**	**12·6 (-4·9–30·9)**	**4·5 (-2·4–14·5)**
South Asia	All causes	9·1 (7·1–11·5)	8·8 (7·0–11·0)	8·3 (6·8–10·2)	0·26 (-0·31–0·89)
South Asia	Diarrheal diseases	31·3 (-24·3–68·6)	21·8 (-6·6–47·0)	8·0 (-5·8–21·9)	8·2 (-10·0–25·9)

South Asia	Lower respiratory infections	16·7 (-42·3–60·2)	10·8 (-18·3–38·7)	3·1 (-0·61–7·1)	6·6 (-19·3–31·6)
South Asia	Malaria	32·4 (-36·1–78·0)	32·4 (-36·1–78·0)	--	--
South Asia	Measles	16·2 (-7·0–40·7)	--	12·6 (-4·9–30·9)	4·5 (-2·4–14·5)
Bangladesh	All causes	6·3 (4·6–8·3)	6·1 (4·7–7·9)	5·9 (4·5–7·6)	0·16 (-0·22–0·54)
Bangladesh	Diarrheal diseases	29·2 (-21·5–66·0)	20·0 (-5·8–44·3)	7·1 (-4·9–19·7)	7·9 (-9·4–25·4)
Bangladesh	Lower respiratory infections	14·9 (-35·9–55·3)	9·6 (-15·3–34·7)	2·1 (-0·42–4·7)	6·0 (-17·3–29·1)
Bangladesh	Malaria	30·6 (-29·9–75·0)	30·6 (-29·9–75·0)	--	--
Bangladesh	Measles	11·8 (-4·6–29·6)	--	10·0 (-3·7–24·5)	2·3 (-1·0–7·6)
Bhutan	All causes	1·5 (0·33–2·7)	1·3 (0·65–2·0)	0·96 (0·66–1·4)	0·24 (-0·29–0·81)
Bhutan	Diarrheal diseases	20·1 (-13·9–49·9)	12·4 (-3·0–29·2)	3·4 (-2·1–9·7)	6·9 (-8·0–22·2)
Bhutan	Lower respiratory infections	10·1 (-23·6–40·8)	5·3 (-7·4–19·7)	0·95 (-0·18–2·2)	5·2 (-14·9–24·9)
Bhutan	Malaria	22·7 (-15·5–62·4)	22·7 (-15·5–62·4)	--	--
Bhutan	Measles	6·2 (-2·4–16·4)	--	5·0 (-1·7–13·0)	1·3 (-0·52–4·4)
India	All causes	9·8 (7·6–12·4)	9·5 (7·7–11·9)	9·1 (7·4–11·2)	0·26 (-0·31–0·87)
India	Diarrheal diseases	32·3 (-25·4–70·0)	22·7 (-7·0–48·4)	8·6 (-6·3–23·5)	8·2 (-10·2–26·1)
India	Lower respiratory infections	17·7 (-45·2–62·9)	11·5 (-20·1–41·2)	3·7 (-0·73–8·4)	6·8 (-19·7–32·4)
India	Malaria	33·3 (-38·4–78·8)	33·3 (-38·4–78·8)	--	--
India	Measles	16·6 (-7·2–41·7)	--	12·9 (-5·0–31·7)	4·6 (-2·5–14·8)
Nepal	All causes	3·6 (2·2–5·1)	3·4 (2·3–4·7)	3·2 (2·2–4·4)	0·15 (-0·20–0·50)
Nepal	Diarrheal diseases	27·9 (-20·5–63·6)	19·0 (-5·3–42·1)	5·8 (-3·9–16·4)	8·1 (-9·9–26·0)
Nepal	Lower respiratory infections	14·5 (-35·7–54·0)	9·1 (-14·1–32·7)	1·9 (-0·37–4·3)	6·2 (-18·0–29·4)
Nepal	Malaria	29·6 (-27·5–73·9)	29·6 (-27·5–73·9)	--	--
Nepal	Measles	11·2 (-4·4–28·5)	--	8·9 (-3·2–22·6)	2·7 (-1·2–8·6)
Pakistan	All causes	7·9 (5·4–10·4)	7·5 (5·6–9·5)	6·9 (5·3–8·6)	0·33 (-0·38–1·1)
Pakistan	Diarrheal diseases	29·0 (-21·7–65·1)	19·9 (-5·8–43·6)	6·6 (-4·6–18·3)	8·1 (-9·8–25·7)
Pakistan	Lower respiratory infections	15·4 (-38·9–56·8)	9·8 (-15·8–35·2)	2·4 (-0·46–5·4)	6·4 (-19·0–30·9)
Pakistan	Malaria	31·5 (-33·5–76·5)	31·5 (-33·5–76·5)	--	--
Pakistan	Measles	13·6 (-5·8–35·0)	--	10·2 (-3·7–25·2)	4·1 (-2·3–13·3)
**Southeast Asia, East Asia, and Oceania**	**All causes**	**3·6 (2·2–5·2)**	**3·3 (2·4–4·5)**	**3·0 (2·3–3·8)**	**0·22 (-0·26–0·76)**

**Southeast Asia, East Asia, and Oceania**	**Diarrheal diseases**	**23·4 (-16·6–55·2)**	**15·4 (-4·1–34·9)**	**4·7 (-3·1–13·3)**	**7·1 (-8·3–22·6)**
**Southeast Asia, East Asia, and Oceania**	**Lower respiratory infections**	**10·8 (-25·1–42·1)**	**6·2 (-9·2–23·0)**	**1·2 (-0·23–2·7)**	**5·0 (-13·9–24·0)**
**Southeast Asia, East Asia, and Oceania**	**Malaria**	**28·9 (-26·5–72·8)**	**28·9 (-26·5–72·8)**	**--**	**--**
**Southeast Asia, East Asia, and Oceania**	**Measles**	**9·5 (-3·8–25·0)**	**--**	**7·4 (-2·6–18·9)**	**2·4 (-1·1–8·2)**
East Asia	All causes	0·27 (>-0.1–0·65)	0·19 (<0.1–0·40)	<0.1 (<0.1–0·17)	<0.1 (>-0.1–0·25)
East Asia	Diarrheal diseases	16·3 (-10·8–41·4)	10·4 (-2·7–24·8)	2·8 (-1·7–8·1)	5·1 (-5·7–16·7)
East Asia	Lower respiratory infections	7·5 (-15·9–30·6)	3·9 (-5·5–15·0)	0·59 (-0·12–1·3)	3·7 (-9·4–17·8)
East Asia	Malaria	34·1 (-56·2–82·3)	34·1 (-56·2–82·3)	--	--
East Asia	Measles	3·8 (-1·3–9·9)	--	3·7 (-1·3–9·7)	<0.1 (>-0.1–0·36)
China	All causes	0·22 (>-0.1–0·51)	0·15 (<0.1–0·31)	<0.1 (<0.1–0·15)	<0.1 (>-0.1–0·19)
China	Diarrheal diseases	14·5 (-8·7–38·3)	8·6 (-2·0–21·7)	2·5 (-1·5–7·3)	4·6 (-5·0–15·2)
China	Lower respiratory infections	7·2 (-13·9–29·4)	3·6 (-4·7–13·9)	0·57 (-0·11–1·3)	3·5 (-8·6–17·1)
China	Malaria	0 (0–0)	0 (0–0)	--	--
China	Measles	4·3 (-1·5–11·5)	--	3·8 (-1·3–10·0)	0·54 (-0·23–1·9)
Democratic People's Republic of Korea	All causes	1·8 (-1·2–4·1)	1·4 (-0·32–3·0)	0·34 (>-0.1–0·85)	0·52 (-0·74–1·7)
Democratic People's Republic of Korea	Diarrheal diseases	35·6 (-31·7–74·6)	27·5 (-9·8–55·6)	5·1 (-3·4–14·6)	11·0 (-15·1–33·7)
Democratic People's Republic of Korea	Lower respiratory infections	15·6 (-58·2–62·0)	11·1 (-24·1–39·5)	0·97 (-0·20–2·2)	7·3 (-27·0–36·3)
Democratic People's Republic of Korea	Malaria	34·1 (-56·2–82·3)	34·1 (-56·2–82·3)	--	--
Democratic People's Republic of Korea	Measles	0 (0–0)	--	0 (0–0)	0 (0–0)
Taiwan	All causes	1·1 (-0·51–2·9)	0·62 (-0·12–1·6)	0·24 (-0·13–0·70)	0·27 (-0·28–0·92)
Taiwan	Diarrheal diseases	11·5 (-6·2–31·1)	6·7 (-1·5–17·2)	2·6 (-1·6–7·5)	3·0 (-3·0–10·1)
Taiwan	Lower respiratory infections	5·5 (-9·6–22·5)	2·9 (-3·7–11·2)	0·52 (-0·10–1·2)	2·4 (-5·6–11·8)
Taiwan	Malaria	0 (0–0)	0 (0–0)	--	--
Taiwan	Measles	3·8 (-1·3–9·8)	--	3·7 (-1·3–9·6)	<0.1 (>-0.1–0·25)
Oceania	All causes	8·5 (4·9–12·2)	8·1 (5·2–11·2)	6·8 (5·1–8·8)	0·40 (-0·49–1·4)
Oceania	Diarrheal diseases	26·8 (-20·6–61·9)	17·8 (-4·9–39·8)	5·1 (-3·4–14·3)	8·7 (-10·9–27·5)
Oceania	Lower respiratory infections	14·6 (-37·5–55·8)	8·6 (-12·8–31·2)	1·9 (-0·38–4·4)	7·1 (-21·3–34·1)
Oceania	Malaria	29·0 (-26·6–72·9)	29·0 (-26·6–72·9)	--	--
Oceania	Measles	12·4 (-5·5–33·7)	--	8·0 (-2·7–20·3)	5·2 (-2·8–17·1)

American Samoa	All causes	0·46 (>-0.1–1·1)	0·30 (<0.1–0·62)	0·22 (<0.1–0·44)	<0.1 (>-0.1–0·34)
American Samoa	Diarrheal diseases	13·3 (-7·8–35·5)	7·7 (-1·7–19·5)	2·5 (-1·5–7·0)	4·1 (-4·3–13·9)
American Samoa	Lower respiratory infections	6·3 (-11·8–25·9)	3·2 (-4·0–12·3)	0·63 (-0·12–1·5)	2·9 (-7·2–14·2)
American Samoa	Malaria	0 (0–0)	0 (0–0)	--	--
American Samoa	Measles	4·3 (-1·5–11·4)	--	3·9 (-1·3–10·2)	0·42 (-0·18–1·4)
Cook Islands	All causes	0·37 (-0·20–1·1)	0·19 (>-0.1–0·54)	<0.1 (>-0.1–0·28)	0·11 (-0·11–0·40)
Cook Islands	Diarrheal diseases	9·2 (-5·1–25·3)	4·9 (-1·0–12·6)	2·0 (-1·2–5·8)	2·8 (-2·8–9·2)
Cook Islands	Lower respiratory infections	4·3 (-7·1–17·7)	2·0 (-2·4–7·7)	0·47 (>-0.1–1·1)	2·0 (-4·5–9·9)
Cook Islands	Malaria	0 (0–0)	0 (0–0)	--	--
Cook Islands	Measles	3·5 (-1·2–9·5)	--	3·3 (-1·1–8·8)	0·24 (-0·11–0·85)
Fiji	All causes	1·5 (0·89–2·3)	1·4 (0·99–2·0)	1·3 (0·95–1·8)	<0.1 (>-0.1–0·27)
Fiji	Diarrheal diseases	13·8 (-7·6–35·9)	8·3 (-1·9–20·6)	3·2 (-2·0–9·2)	3·4 (-3·5–11·1)
Fiji	Lower respiratory infections	6·6 (-10·9–25·9)	3·5 (-4·4–13·8)	0·93 (-0·18–2·2)	2·6 (-6·0–12·3)
Fiji	Malaria	0 (0–0)	0 (0–0)	--	--
Fiji	Measles	5·3 (-1·8–14·0)	--	5·1 (-1·7–13·2)	0·25 (-0·11–0·90)
Guam	All causes	0·34 (-0·19–1·0)	0·18 (>-0.1–0·49)	<0.1 (>-0.1–0·26)	<0.1 (-0·10–0·36)
Guam	Diarrheal diseases	9·3 (-5·2–26·0)	5·0 (-1·1–13·0)	2·1 (-1·3–6·1)	2·7 (-2·8–9·5)
Guam	Lower respiratory infections	4·4 (-7·3–18·2)	2·0 (-2·4–7·9)	0·50 (>-0.1–1·1)	2·0 (-4·6–10·1)
Guam	Malaria	0 (0–0)	0 (0–0)	--	--
Guam	Measles	3·6 (-1·2–9·7)	--	3·3 (-1·1–9·0)	0·25 (-0·11–0·86)
Kiribati	All causes	1·2 (0·47–2·0)	1·0 (0·58–1·5)	0·84 (0·55–1·2)	0·11 (-0·13–0·40)
Kiribati	Diarrheal diseases	18·9 (-11·5–46·8)	12·2 (-3·0–29·0)	3·4 (-2·2–9·8)	5·3 (-6·0–17·3)
Kiribati	Lower respiratory infections	9·5 (-19·3–37·4)	5·5 (-7·4–20·5)	0·81 (-0·16–1·9)	4·2 (-11·1–20·0)
Kiribati	Malaria	0 (0–0)	0 (0–0)	--	--
Kiribati	Measles	5·8 (-2·1–15·6)	--	5·0 (-1·8–13·3)	0·88 (-0·38–3·0)
Marshall Islands	All causes	0·33 (-0·18–0·98)	0·24 (>-0.1–0·67)	<0.1 (>-0.1–<0.1)	<0.1 (-0·12–0·37)
Marshall Islands	Diarrheal diseases	16·7 (-8·9–42·0)	12·3 (-3·0–29·4)	0·84 (-0·50–2·5)	4·8 (-5·2–15·9)
Marshall Islands	Lower respiratory infections	8·9 (-17·4–36·6)	5·9 (-7·7–22·6)	0·12 (>-0.1–0·27)	3·7 (-9·2–18·0)
Marshall Islands	Malaria	0 (0–0)	0 (0–0)	--	--

Marshall Islands	Measles	1·8 (-0·68–5·1)	--	1·2 (-0·41–3·4)	0·56 (-0·25–2·0)
Micronesia (Federated States of)	All causes	1·5 (0·86–2·4)	1·3 (0·85–2·1)	1·2 (0·79–1·9)	<0.1 (-0·10–0·32)
Micronesia (Federated States of)	Diarrheal diseases	17·2 (-10·6–43·8)	10·8 (-2·6–26·1)	3·0 (-1·9–8·5)	5·3 (-5·8–17·1)
Micronesia (Federated States of)	Lower respiratory infections	8·7 (-17·5–34·7)	4·7 (-6·2–17·9)	0·76 (-0·14–1·8)	4·0 (-10·3–19·2)
Micronesia (Federated States of)	Malaria	0 (0–0)	0 (0–0)	--	--
Micronesia (Federated States of)	Measles	5·2 (-1·9–13·5)	--	4·5 (-1·5–11·5)	0·71 (-0·30–2·4)
Nauru	All causes	0·35 (-0·20–0·97)	0·21 (>-0.1–0·53)	<0.1 (>-0.1–0·12)	0·13 (-0·14–0·45)
Nauru	Diarrheal diseases	13·9 (-8·7–37·1)	8·3 (-1·9–20·8)	1·5 (-0·91–4·3)	5·2 (-5·7–17·1)
Nauru	Lower respiratory infections	7·3 (-15·1–30·5)	3·4 (-4·3–13·0)	0·27 (>-0.1–0·62)	4·2 (-10·3–19·7)
Nauru	Malaria	0 (0–0)	0 (0–0)	--	--
Nauru	Measles	3·5 (-1·4–9·8)	--	2·2 (-0·73–5·6)	1·3 (-0·58–4·6)
Niue	All causes	0·38 (-0·12–0·97)	0·25 (<0.1–0·56)	0·13 (<0.1–0·28)	<0.1 (-0·10–0·33)
Niue	Diarrheal diseases	12·4 (-7·0–33·2)	7·2 (-1·6–18·3)	2·5 (-1·5–7·0)	3·6 (-3·7–12·1)
Niue	Lower respiratory infections	6·0 (-10·5–24·1)	3·0 (-3·7–11·6)	0·60 (-0·11–1·4)	2·7 (-6·4–13·0)
Niue	Malaria	0 (0–0)	0 (0–0)	--	--
Niue	Measles	4·2 (-1·4–11·2)	--	3·8 (-1·3–10·1)	0·39 (-0·16–1·3)
Northern Mariana Islands	All causes	0·28 (-0·15–0·82)	0·15 (>-0.1–0·41)	<0.1 (>-0.1–0·22)	<0.1 (>-0.1–0·29)
Northern Mariana Islands	Diarrheal diseases	10·1 (-5·6–27·8)	5·6 (-1·2–14·3)	2·2 (-1·4–6·4)	3·0 (-3·0–10·1)
Northern Mariana Islands	Lower respiratory infections	4·9 (-8·0–19·8)	2·3 (-2·8–8·9)	0·52 (-0·10–1·2)	2·3 (-5·0–10·8)
Northern Mariana Islands	Malaria	0 (0–0)	0 (0–0)	--	--
Northern Mariana Islands	Measles	3·7 (-1·3–9·9)	--	3·5 (-1·1–9·1)	0·28 (-0·12–0·98)
Palau	All causes	0·46 (-0·15–1·2)	0·29 (<0.1–0·68)	0·16 (>-0.1–0·36)	0·11 (-0·12–0·37)
Palau	Diarrheal diseases	11·7 (-6·5–31·4)	6·8 (-1·5–17·1)	2·6 (-1·6–7·3)	3·2 (-3·3–10·7)
Palau	Lower respiratory infections	5·8 (-9·9–23·4)	2·9 (-3·6–11·2)	0·60 (-0·12–1·4)	2·6 (-6·0–12·5)
Palau	Malaria	0 (0–0)	0 (0–0)	--	--
Palau	Measles	4·1 (-1·4–11·0)	--	3·8 (-1·3–9·9)	0·37 (-0·16–1·3)
Papua New Guinea	All causes	9·4 (5·4–13·4)	8·9 (5·8–12·3)	7·4 (5·6–9·7)	0·44 (-0·54–1·5)
Papua New Guinea	Diarrheal diseases	27·5 (-21·3–63·3)	18·3 (-5·1–40·8)	5·3 (-3·5–14·7)	9·0 (-11·3–28·3)
Papua New Guinea	Lower respiratory infections	15·6 (-40·8–59·2)	9·2 (-13·8–33·3)	2·1 (-0·41–4·8)	7·6 (-23·2–36·5)

Papua New Guinea	Malaria	29·4 (-27·3–73·4)	29·4 (-27·3–73·4)	--	--
Papua New Guinea	Measles	13·1 (-5·7–35·5)	--	8·2 (-2·8–21·0)	5·6 (-3·0–18·4)
Samoa	All causes	2·0 (1·3–2·8)	1·9 (1·3–2·6)	1·8 (1·2–2·5)	<0.1 (>-0.1–0·30)
Samoa	Diarrheal diseases	13·0 (-7·7–34·8)	7·3 (-1·6–18·4)	2·8 (-1·8–8·0)	4·0 (-4·1–13·2)
Samoa	Lower respiratory infections	6·0 (-10·4–24·1)	2·9 (-3·6–11·0)	0·82 (-0·16–2·0)	2·7 (-6·4–13·0)
Samoa	Malaria	0 (0–0)	0 (0–0)	--	--
Samoa	Measles	4·6 (-1·6–11·9)	--	4·5 (-1·6–11·4)	0·16 (>-0.1–0·57)
Solomon Islands	All causes	5·9 (3·1–9·2)	5·7 (3·2–8·9)	4·5 (3·2–6·0)	0·11 (-0·14–0·39)
Solomon Islands	Diarrheal diseases	21·2 (-13·6–51·4)	13·5 (-3·3–31·5)	4·4 (-2·9–12·3)	6·2 (-6·9–19·7)
Solomon Islands	Lower respiratory infections	11·9 (-24·2–46·3)	6·7 (-8·9–25·0)	1·5 (-0·28–3·5)	5·5 (-13·7–26·1)
Solomon Islands	Malaria	24·2 (-17·3–65·5)	24·2 (-17·3–65·5)	--	--
Solomon Islands	Measles	9·7 (-3·9–26·1)	--	6·8 (-2·3–17·3)	3·3 (-1·7–11·0)
Tokelau	All causes	0·35 (>-0.1–0·78)	0·25 (<0.1–0·51)	0·17 (<0.1–0·31)	<0.1 (>-0.1–0·24)
Tokelau	Diarrheal diseases	13·4 (-7·8–35·6)	8·0 (-1·8–19·9)	2·5 (-1·5–7·0)	4·1 (-4·3–13·4)
Tokelau	Lower respiratory infections	6·4 (-11·4–25·3)	3·3 (-4·1–12·5)	0·62 (-0·12–1·5)	2·9 (-6·9–13·6)
Tokelau	Malaria	0 (0–0)	0 (0–0)	--	--
Tokelau	Measles	4·4 (-1·6–11·5)	--	3·9 (-1·4–10·1)	0·45 (-0·19–1·6)
Tonga	All causes	1·5 (0·96–2·2)	1·5 (0·92–2·2)	1·5 (0·90–2·2)	<0.1 (>-0.1–<0.1)
Tonga	Diarrheal diseases	6·3 (-3·4–17·7)	3·2 (-0·65–8·3)	1·7 (-1·0–4·8)	1·7 (-1·7–5·8)
Tonga	Lower respiratory infections	2·9 (-4·2–11·8)	1·3 (-1·5–5·2)	0·47 (>-0.1–1·1)	1·2 (-2·6–6·0)
Tonga	Malaria	0 (0–0)	0 (0–0)	--	--
Tonga	Measles	2·9 (-0·90–7·9)	--	2·7 (-0·85–7·5)	0·14 (>-0.1–0·49)
Tuvalu	All causes	0·32 (-0·16–0·91)	0·18 (>-0.1–0·51)	<0.1 (>-0.1–0·23)	<0.1 (>-0.1–0·32)
Tuvalu	Diarrheal diseases	12·3 (-7·2–32·9)	7·0 (-1·5–17·6)	2·3 (-1·4–6·7)	3·9 (-4·0–12·8)
Tuvalu	Lower respiratory infections	6·0 (-10·9–25·0)	2·8 (-3·6–11·1)	0·58 (-0·11–1·3)	3·0 (-6·9–14·6)
Tuvalu	Malaria	0 (0–0)	0 (0–0)	--	--
Tuvalu	Measles	3·9 (-1·4–10·5)	--	3·4 (-1·1–9·0)	0·51 (-0·22–1·7)
Vanuatu	All causes	1·8 (0·17–3·4)	1·4 (0·57–2·4)	1·1 (0·56–1·7)	0·27 (-0·31–0·95)
Vanuatu	Diarrheal diseases	20·4 (-13·5–50·3)	13·0 (-3·3–30·7)	3·9 (-2·5–11·0)	6·2 (-7·3–20·1)

Vanuatu	Lower respiratory infections	10·7 (-23·1–42·2)	5·8 (-8·0–22·0)	0·99 (-0·19–2·3)	5·2 (-13·7–24·6)
Vanuatu	Malaria	23·8 (-16·9–64·9)	23·8 (-16·9–64·9)	--	--
Vanuatu	Measles	7·4 (-2·8–19·4)	--	5·8 (-2·0–14·6)	1·8 (-0·77–6·0)
Southeast Asia	All causes	6·0 (4·0–8·5)	5·6 (4·2–7·5)	5·1 (4·0–6·5)	0·34 (-0·40–1·1)
Southeast Asia	Diarrheal diseases	24·8 (-17·7–58·3)	16·4 (-4·4–37·2)	5·1 (-3·4–14·5)	7·4 (-8·7–23·7)
Southeast Asia	Lower respiratory infections	13·0 (-31·3–49·7)	7·8 (-11·8–28·5)	1·5 (-0·30–3·5)	5·8 (-16·8–28·0)
Southeast Asia	Malaria	28·6 (-25·9–72·3)	28·6 (-25·9–72·3)	--	--
Southeast Asia	Measles	9·2 (-3·7–24·3)	--	7·4 (-2·6–18·9)	2·1 (-0·95–7·3)
Cambodia	All causes	3·7 (2·2–5·3)	3·5 (2·5–5·0)	3·2 (2·3–4·6)	0·16 (-0·23–0·61)
Cambodia	Diarrheal diseases	26·7 (-19·7–61·4)	18·0 (-5·1–40·3)	5·7 (-3·8–16·0)	7·8 (-9·3–24·9)
Cambodia	Lower respiratory infections	14·4 (-37·7–55·1)	9·0 (-14·4–32·7)	1·8 (-0·35–4·0)	6·4 (-19·6–31·3)
Cambodia	Malaria	29·9 (-28·6–75·4)	29·9 (-28·6–75·4)	--	--
Cambodia	Measles	10·6 (-4·3–27·3)	--	8·4 (-3·0–21·1)	2·6 (-1·1–8·8)
Indonesia	All causes	9·1 (6·2–12·3)	8·6 (6·7–11·2)	8·0 (6·2–10·2)	0·43 (-0·52–1·4)
Indonesia	Diarrheal diseases	26·0 (-19·4–60·5)	17·2 (-4·7–38·6)	5·2 (-3·5–14·7)	8·2 (-9·8–26·1)
Indonesia	Lower respiratory infections	13·9 (-34·9–53·6)	8·3 (-12·6–30·5)	1·7 (-0·33–3·9)	6·4 (-19·5–31·0)
Indonesia	Malaria	29·3 (-26·6–73·0)	29·3 (-26·6–73·0)	--	--
Indonesia	Measles	9·9 (-4·0–26·0)	--	7·7 (-2·7–19·7)	2·5 (-1·2–8·5)
Lao People's Democratic Republic	All causes	3·2 (1·1–5·3)	2·8 (1·6–4·3)	2·3 (1·5–3·3)	0·30 (-0·37–1·1)
Lao People's Democratic Republic	Diarrheal diseases	24·8 (-17·2–57·7)	16·5 (-4·5–37·2)	5·4 (-3·6–15·4)	6·9 (-8·2–22·0)
Lao People's Democratic Republic	Lower respiratory infections	13·9 (-35·7–53·2)	8·6 (-13·8–31·4)	1·5 (-0·30–3·5)	6·2 (-18·4–30·1)
Lao People's Democratic Republic	Malaria	28·9 (-27·8–72·8)	28·9 (-27·8–72·8)	--	--
Lao People's Democratic Republic	Measles	10·6 (-4·3–27·6)	--	8·1 (-2·9–20·6)	2·9 (-1·3–9·5)
Malaysia	All causes	6·8 (4·6–9·2)	6·5 (4·7–8·6)	6·2 (4·6–8·1)	0·21 (-0·21–0·72)
Malaysia	Diarrheal diseases	20·7 (-12·5–50·8)	13·3 (-3·3–31·7)	4·8 (-3·1–13·5)	5·2 (-5·7–17·0)
Malaysia	Lower respiratory infections	10·9 (-20·1–41·1)	6·2 (-8·5–23·8)	1·6 (-0·31–3·8)	4·2 (-10·4–19·8)
Malaysia	Malaria	23·3 (-15·7–64·3)	23·3 (-15·7–64·3)	--	--
Malaysia	Measles	8·3 (-3·0–21·7)	--	7·2 (-2·5–18·5)	1·3 (-0·55–4·5)
Maldives	All causes	4·3 (1·7–7·0)	3·8 (2·3–5·6)	3·2 (2·2–4·4)	0·38 (-0·40–1·3)

Maldives	Diarrheal diseases	23·4 (-15·1–55·6)	15·4 (-4·0–35·5)	5·4 (-3·6–15·2)	6·1 (-6·9–19·6)
Maldives	Lower respiratory infections	12·0 (-24·5–45·8)	7·3 (-10·6–27·3)	1·7 (-0·34–3·8)	4·7 (-12·1–22·6)
Maldives	Malaria	0 (0–0)	0 (0–0)	--	--
Maldives	Measles	8·6 (-3·2–22·4)	--	7·5 (-2·7–19·3)	1·2 (-0·53–4·3)
Mauritius	All causes	5·0 (3·1–7·6)	4·6 (3·1–6·8)	4·4 (3·0–6·2)	0·22 (-0·25–0·81)
Mauritius	Diarrheal diseases	20·3 (-12·7–50·0)	12·5 (-3·1–29·7)	5·8 (-3·9–16·3)	4·8 (-5·2–15·8)
Mauritius	Lower respiratory infections	10·9 (-20·0–40·1)	6·0 (-8·4–22·2)	2·2 (-0·42–5·1)	4·0 (-10·0–19·1)
Mauritius	Malaria	0 (0–0)	0 (0–0)	--	--
Mauritius	Measles	9·3 (-3·4–24·3)	--	8·8 (-3·1–22·9)	0·60 (-0·24–2·1)
Myanmar	All causes	2·4 (0·41–4·5)	2·1 (0·96–3·5)	1·5 (0·92–2·3)	0·31 (-0·36–1·1)
Myanmar	Diarrheal diseases	24·0 (-16·2–56·8)	16·0 (-4·3–36·7)	5·2 (-3·4–14·7)	6·6 (-7·7–21·5)
Myanmar	Lower respiratory infections	12·8 (-30·7–48·8)	7·9 (-12·3–28·6)	1·3 (-0·26–3·0)	5·6 (-16·9–26·9)
Myanmar	Malaria	28·2 (-24·1–71·1)	28·2 (-24·1–71·1)	--	--
Myanmar	Measles	9·1 (-3·5–22·9)	--	7·5 (-2·7–18·9)	1·8 (-0·73–6·3)
Philippines	All causes	4·3 (2·5–6·3)	4·0 (2·8–5·5)	3·5 (2·6–4·6)	0·29 (-0·35–0·97)
Philippines	Diarrheal diseases	25·9 (-18·3–60·4)	17·5 (-4·8–39·4)	5·2 (-3·5–14·9)	7·5 (-8·8–24·2)
Philippines	Lower respiratory infections	13·1 (-31·6–50·7)	8·1 (-12·4–29·7)	1·5 (-0·29–3·4)	5·8 (-16·5–28·0)
Philippines	Malaria	30·0 (-28·7–74·8)	30·0 (-28·7–74·8)	--	--
Philippines	Measles	9·6 (-3·8–25·9)	--	7·7 (-2·8–19·8)	2·2 (-1·0–7·9)
Seychelles	All causes	0·83 (-0·31–2·0)	0·57 (<0.1–1·2)	0·30 (<0.1–0·67)	0·18 (-0·22–0·63)
Seychelles	Diarrheal diseases	17·5 (-10·4–44·3)	10·9 (-2·6–26·5)	4·0 (-2·6–11·6)	4·6 (-4·9–15·2)
Seychelles	Lower respiratory infections	8·4 (-17·3–33·0)	4·6 (-6·7–17·2)	0·88 (-0·18–2·0)	3·7 (-9·6–17·7)
Seychelles	Malaria	0 (0–0)	0 (0–0)	--	--
Seychelles	Measles	6·0 (-2·1–15·6)	--	5·8 (-2·0–15·0)	0·24 (>-0.1–0·92)
Sri Lanka	All causes	10·9 (8·0–14·3)	10·5 (8·0–13·5)	10·1 (7·6–12·9)	0·29 (-0·36–0·97)
Sri Lanka	Diarrheal diseases	28·1 (-20·7–64·2)	18·8 (-5·3–42·1)	7·1 (-4·9–19·8)	7·5 (-8·7–24·3)
Sri Lanka	Lower respiratory infections	14·7 (-34·2–54·3)	9·2 (-14·8–33·5)	2·5 (-0·49–5·6)	5·7 (-18·1–28·0)
Sri Lanka	Malaria	0 (0–0)	0 (0–0)	--	--
Sri Lanka	Measles	10·4 (-3·9–25·8)	--	10·0 (-3·7–24·9)	0·54 (-0·18–2·1)

Thailand	All causes	2·9 (1·3–4·8)	2·5 (1·6–3·8)	2·2 (1·5–3·1)	0·26 (-0·27–0·89)
Thailand	Diarrheal diseases	17·4 (-10·6–44·2)	10·6 (-2·5–25·7)	3·8 (-2·4–10·9)	4·9 (-5·4–16·1)
Thailand	Lower respiratory infections	8·8 (-16·3–35·0)	4·6 (-6·0–17·7)	1·1 (-0·21–2·5)	3·9 (-9·5–19·1)
Thailand	Malaria	19·3 (-11·2–56·2)	19·3 (-11·2–56·2)	--	--
Thailand	Measles	6·2 (-2·2–16·5)	--	5·4 (-1·8–14·0)	0·95 (-0·43–3·2)
Timor-Leste	All causes	16·8 (12·9–21·0)	16·5 (12·9–20·8)	16·2 (12·8–20·3)	0·23 (-0·30–0·75)
Timor-Leste	Diarrheal diseases	30·2 (-22·9–67·1)	20·7 (-6·1–45·3)	7·9 (-5·6–21·7)	7·9 (-9·6–25·0)
Timor-Leste	Lower respiratory infections	18·4 (-46·4–65·2)	12·1 (-19·9–42·9)	3·4 (-0·66–7·7)	7·4 (-21·1–35·1)
Timor-Leste	Malaria	32·7 (-35·8–78·2)	32·7 (-35·8–78·2)	--	--
Timor-Leste	Measles	17·0 (-7·6–43·2)	--	12·2 (-4·5–30·1)	6·0 (-3·3–19·1)
Viet Nam	All causes	2·8 (1·1–4·6)	2·4 (1·5–3·7)	2·0 (1·4–2·9)	0·28 (-0·30–1·0)
Viet Nam	Diarrheal diseases	22·7 (-15·3–54·9)	14·7 (-3·8–34·3)	4·6 (-3·0–13·3)	6·7 (-7·8–21·6)
Viet Nam	Lower respiratory infections	11·1 (-23·8–42·6)	6·3 (-9·0–23·2)	1·2 (-0·24–2·9)	5·0 (-13·2–23·6)
Viet Nam	Malaria	24·5 (-18·1–65·6)	24·5 (-18·1–65·6)	--	--
Viet Nam	Measles	8·0 (-3·0–21·6)	--	6·5 (-2·3–16·9)	1·6 (-0·76–5·7)
**Sub-Saharan Africa**	**All causes**	**5·9 (1·6–9·7)**	**5·6 (1·9–9·0)**	**3·6 (2·8–4·6)**	**0·24 (-0·29–0·79)**
**Sub-Saharan Africa**	**Diarrheal diseases**	**25·0 (-18·0–58·3)**	**16·6 (-4·6–37·4)**	**5·3 (-3·6–14·8)**	**7·4 (-8·8–23·5)**
**Sub-Saharan Africa**	**Lower respiratory infections**	**13·6 (-33·8–51·9)**	**8·2 (-12·7–30·1)**	**1·7 (-0·33–3·9)**	**6·2 (-18·1–29·6)**
**Sub-Saharan Africa**	**Malaria**	**27·5 (-24·1–70·6)**	**27·5 (-24·1–70·6)**	**--**	**--**
**Sub-Saharan Africa**	**Measles**	**10·9 (-4·6–28·5)**	**--**	**8·1 (-2·9–20·3)**	**3·4 (-1·7–10·9)**
Central Sub-Saharan Africa	All causes	5·9 (0·30–10·6)	5·6 (0·65–10·2)	2·9 (2·2–3·9)	0·22 (-0·27–0·73)
Central Sub-Saharan Africa	Diarrheal diseases	25·6 (-18·6–59·8)	17·1 (-4·6–38·6)	4·8 (-3·1–13·5)	8·0 (-9·8–25·4)
Central Sub-Saharan Africa	Lower respiratory infections	14·0 (-35·4–53·6)	8·3 (-12·6–30·3)	1·6 (-0·30–3·6)	6·7 (-19·8–31·8)
Central Sub-Saharan Africa	Malaria	28·1 (-24·7–71·8)	28·1 (-24·7–71·8)	--	--
Central Sub-Saharan Africa	Measles	11·0 (-4·6–29·3)	--	7·5 (-2·6–19·2)	4·0 (-2·0–12·8)
Angola	All causes	3·7 (-0·82–8·0)	3·4 (-0·28–7·3)	1·3 (0·86–1·9)	0·28 (-0·35–0·96)
Angola	Diarrheal diseases	25·6 (-18·6–60·0)	17·2 (-4·7–39·0)	4·2 (-2·7–12·0)	8·3 (-10·0–26·3)
Angola	Lower respiratory infections	13·6 (-35·8–53·1)	8·1 (-12·5–29·9)	1·1 (-0·23–2·6)	6·6 (-19·9–31·8)
Angola	Malaria	28·2 (-24·1–71·8)	28·2 (-24·1–71·8)	--	--

Angola	Measles	9·4 (-4·0–25·6)	--	6·4 (-2·2–16·4)	3·4 (-1·6–11·3)
Central African Republic	All causes	7·4 (-0·15–13·9)	6·9 (0·71–12·8)	3·6 (2·7–4·8)	0·51 (-0·64–1·6)
Central African Republic	Diarrheal diseases	26·7 (-19·8–61·4)	18·1 (-5·1–40·2)	5·0 (-3·3–14·1)	8·3 (-10·3–25·9)
Central African Republic	Lower respiratory infections	14·6 (-37·0–55·9)	9·0 (-13·4–32·6)	1·5 (-0·30–3·6)	6·9 (-20·2–33·0)
Central African Republic	Malaria	29·0 (-26·2–72·8)	29·0 (-26·2–72·8)	--	--
Central African Republic	Measles	11·5 (-4·9–30·7)	--	7·7 (-2·7–19·4)	4·3 (-2·3–14·2)
Congo	All causes	4·4 (0·58–8·7)	4·2 (0·92–8·1)	2·1 (1·6–2·9)	0·18 (-0·21–0·60)
Congo	Diarrheal diseases	20·8 (-13·3–50·9)	13·1 (-3·2–30·8)	4·6 (-3·0–12·8)	5·9 (-6·6–18·9)
Congo	Lower respiratory infections	10·8 (-21·6–41·4)	5·9 (-8·1–22·0)	1·3 (-0·26–3·1)	4·8 (-12·2–22·7)
Congo	Malaria	23·2 (-15·5–63·8)	23·2 (-15·5–63·8)	--	--
Congo	Measles	8·6 (-3·2–22·9)	--	6·8 (-2·3–18·0)	2·0 (-0·84–6·7)
Democratic Republic of the Congo	All causes	6·6 (0·76–12·2)	6·4 (1·1–11·8)	3·5 (2·6–4·8)	0·17 (-0·21–0·60)
Democratic Republic of the Congo	Diarrheal diseases	25·9 (-18·8–60·4)	17·2 (-4·7–38·9)	5·2 (-3·4–14·5)	7·9 (-9·8–25·4)
Democratic Republic of the Congo	Lower respiratory infections	14·2 (-36·1–54·2)	8·5 (-12·8–30·7)	1·7 (-0·32–3·9)	6·8 (-20·0–32·2)
Democratic Republic of the Congo	Malaria	28·4 (-25·4–72·2)	28·4 (-25·4–72·2)	--	--
Democratic Republic of the Congo	Measles	11·4 (-4·8–30·3)	--	7·8 (-2·7–19·9)	4·2 (-2·2–13·3)
Equatorial Guinea	All causes	1·8 (-0·76–5·2)	1·6 (-0·55–4·7)	0·23 (<0.1–0·41)	0·12 (-0·15–0·42)
Equatorial Guinea	Diarrheal diseases	16·3 (-11·1–42·5)	8·9 (-2·0–22·0)	3·0 (-1·9–8·5)	6·2 (-7·0–19·7)
Equatorial Guinea	Lower respiratory infections	8·8 (-19·5–36·2)	3·9 (-4·9–15·0)	0·91 (-0·18–2·1)	4·9 (-13·6–23·5)
Equatorial Guinea	Malaria	17·6 (-9·8–53·5)	17·6 (-9·8–53·5)	--	--
Equatorial Guinea	Measles	6·2 (-2·5–17·3)	--	4·6 (-1·5–12·0)	1·8 (-0·78–6·2)
Gabon	All causes	1·7 (-0·79–5·1)	1·5 (-0·50–4·5)	0·29 (0·10–0·54)	0·17 (-0·19–0·59)
Gabon	Diarrheal diseases	15·0 (-9·4–39·1)	8·6 (-2·0–21·3)	2·7 (-1·7–7·8)	5·1 (-5·5–16·4)
Gabon	Lower respiratory infections	7·7 (-14·7–31·2)	3·6 (-4·5–13·9)	0·70 (-0·14–1·6)	4·0 (-9·6–18·9)
Gabon	Malaria	17·0 (-9·2–52·0)	17·0 (-9·2–52·0)	--	--
Gabon	Measles	5·0 (-1·9–13·5)	--	3·9 (-1·3–10·1)	1·2 (-0·51–4·0)
Eastern Sub-Saharan Africa	All causes	4·6 (1·6–7·3)	4·3 (2·0–6·6)	3·0 (2·4–3·9)	0·23 (-0·28–0·79)
Eastern Sub-Saharan Africa	Diarrheal diseases	24·4 (-17·5–57·5)	16·0 (-4·3–36·4)	4·8 (-3·2–13·5)	7·6 (-9·0–24·1)
Eastern Sub-Saharan Africa	Lower respiratory infections	13·2 (-33·1–51·2)	7·8 (-11·8–28·7)	1·5 (-0·29–3·4)	6·3 (-18·5–30·1)

Eastern Sub-Saharan Africa	Malaria	25·8 (-20·5–67·8)	25·8 (-20·5–67·8)	--	--
Eastern Sub-Saharan Africa	Measles	10·5 (-4·4–27·5)	--	7·7 (-2·8–19·4)	3·3 (-1·5–10·9)
Burundi	All causes	4·3 (-1·6–9·2)	4·1 (-1·1–8·7)	1·5 (1·0–2·0)	0·26 (-0·35–0·87)
Burundi	Diarrheal diseases	30·3 (-24·5–67·4)	21·7 (-6·5–46·7)	4·6 (-3·0–12·9)	10·0 (-12·8–31·2)
Burundi	Lower respiratory infections	16·3 (-51·6–63·6)	10·8 (-18·6–38·4)	1·2 (-0·23–2·7)	8·3 (-27·2–40·2)
Burundi	Malaria	32·5 (-35·0–77·9)	32·5 (-35·0–77·9)	--	--
Burundi	Measles	12·5 (-5·5–34·1)	--	7·1 (-2·6–17·9)	6·1 (-3·1–19·5)
Comoros	All causes	5·7 (3·8–8·1)	5·5 (3·8–7·6)	4·9 (3·6–6·7)	0·18 (-0·22–0·61)
Comoros	Diarrheal diseases	24·1 (-17·1–57·4)	15·3 (-4·0–35·3)	5·5 (-3·7–15·3)	7·3 (-8·6–23·3)
Comoros	Lower respiratory infections	13·7 (-30·8–52·1)	7·6 (-10·8–28·2)	2·1 (-0·42–4·9)	6·3 (-17·5–30·4)
Comoros	Malaria	26·4 (-21·3–69·6)	26·4 (-21·3–69·6)	--	--
Comoros	Measles	11·4 (-4·7–30·4)	--	8·3 (-2·9–21·3)	3·6 (-1·7–12·0)
Djibouti	All causes	12·1 (8·9–17·1)	11·8 (8·8–16·4)	11·1 (8·5–14·7)	0·21 (-0·25–0·70)
Djibouti	Diarrheal diseases	27·6 (-19·5–62·7)	17·9 (-4·9–40·1)	8·6 (-6·2–23·3)	6·3 (-7·2–20·2)
Djibouti	Lower respiratory infections	16·7 (-33·9–57·8)	9·8 (-15·1–35·9)	4·4 (-0·85–10·0)	5·8 (-15·5–27·8)
Djibouti	Malaria	29·2 (-26·5–73·0)	29·2 (-26·5–73·0)	--	--
Djibouti	Measles	16·5 (-7·0–41·3)	--	13·5 (-5·1–33·0)	4·0 (-1·9–13·1)
Eritrea	All causes	6·2 (4·1–8·6)	5·9 (4·1–7·9)	5·5 (3·9–7·2)	0·22 (-0·28–0·75)
Eritrea	Diarrheal diseases	30·8 (-23·8–67·9)	21·3 (-6·3–46·0)	8·0 (-5·8–22·1)	8·0 (-9·7–25·4)
Eritrea	Lower respiratory infections	17·0 (-45·0–62·3)	11·3 (-19·0–40·6)	2·7 (-0·55–6·3)	6·9 (-20·9–33·4)
Eritrea	Malaria	32·4 (-35·4–77·4)	32·4 (-35·4–77·4)	--	--
Eritrea	Measles	15·0 (-6·4–38·0)	--	12·1 (-4·6–29·2)	3·5 (-1·8–12·6)
Ethiopia	All causes	4·9 (3·1–6·7)	4·6 (3·2–6·0)	4·0 (3·1–5·1)	0·22 (-0·27–0·76)
Ethiopia	Diarrheal diseases	26·5 (-19·1–61·2)	17·7 (-4·8–39·6)	5·8 (-4·0–16·3)	7·7 (-9·3–24·6)
Ethiopia	Lower respiratory infections	14·6 (-36·6–54·9)	8·9 (-14·0–32·3)	2·1 (-0·40–4·8)	6·5 (-19·1–30·9)
Ethiopia	Malaria	29·1 (-26·3–72·8)	29·1 (-26·3–72·8)	--	--
Ethiopia	Measles	12·0 (-5·1–31·6)	--	8·8 (-3·2–22·1)	3·7 (-1·9–12·3)
Kenya	All causes	2·7 (0·18–5·1)	2·4 (0·71–4·0)	1·5 (1·1–2·0)	0·34 (-0·40–1·2)
Kenya	Diarrheal diseases	23·2 (-16·1–55·2)	15·3 (-4·1–34·9)	4·4 (-2·9–12·5)	7·1 (-8·3–22·7)

Kenya	Lower respiratory infections	11·9 (-28·0–46·4)	7·0 (-10·5–25·5)	1·2 (-0·23–2·6)	5·5 (-15·6–26·6)
Kenya	Malaria	26·8 (-22·9–69·4)	26·8 (-22·9–69·4)	--	--
Kenya	Measles	8·3 (-3·3–21·9)	--	6·5 (-2·3–16·7)	2·0 (-0·90–6·8)
Madagascar	All causes	8·1 (3·3–12·6)	7·6 (4·2–11·2)	5·9 (4·5–7·6)	0·51 (-0·61–1·6)
Madagascar	Diarrheal diseases	28·0 (-20·9–63·7)	19·4 (-5·6–42·9)	5·1 (-3·4–14·5)	8·6 (-10·4–27·1)
Madagascar	Lower respiratory infections	15·0 (-40·6–58·0)	9·6 (-15·5–35·3)	1·5 (-0·29–3·4)	6·9 (-21·2–33·6)
Madagascar	Malaria	30·6 (-29·7–75·1)	30·6 (-29·7–75·1)	--	--
Madagascar	Measles	11·1 (-4·7–29·9)	--	7·7 (-2·7–19·5)	4·0 (-2·0–13·7)
Malawi	All causes	2·9 (-0·77–7·5)	2·6 (-0·37–6·8)	0·83 (0·53–1·2)	0·29 (-0·32–0·97)
Malawi	Diarrheal diseases	19·9 (-14·0–49·9)	12·3 (-3·0–29·5)	2·7 (-1·7–7·8)	7·4 (-8·5–23·8)
Malawi	Lower respiratory infections	11·0 (-27·6–44·8)	5·7 (-8·0–21·4)	0·67 (-0·13–1·5)	6·1 (-18·0–29·0)
Malawi	Malaria	23·4 (-16·3–64·3)	23·4 (-16·3–64·3)	--	--
Malawi	Measles	6·4 (-2·7–18·0)	--	4·0 (-1·3–10·4)	2·6 (-1·2–8·6)
Mozambique	All causes	3·0 (-1·1–7·2)	2·9 (-0·88–6·9)	0·68 (0·47–0·91)	0·12 (-0·14–0·38)
Mozambique	Diarrheal diseases	21·5 (-15·1–52·0)	13·6 (-3·4–31·4)	3·5 (-2·3–10·1)	7·4 (-8·8–23·4)
Mozambique	Lower respiratory infections	12·0 (-29·6–46·9)	6·4 (-9·0–23·6)	1·0 (-0·20–2·4)	6·3 (-18·8–29·3)
Mozambique	Malaria	24·4 (-17·8–65·8)	24·4 (-17·8–65·8)	--	--
Mozambique	Measles	8·0 (-3·3–22·1)	--	5·3 (-1·8–14·1)	3·0 (-1·2–10·1)
Rwanda	All causes	1·3 (-0·59–3·0)	0·95 (-0·15–2·1)	0·34 (0·13–0·58)	0·33 (-0·45–1·1)
Rwanda	Diarrheal diseases	20·8 (-15·4–51·8)	13·0 (-3·2–30·6)	2·4 (-1·5–7·0)	8·1 (-9·8–25·8)
Rwanda	Lower respiratory infections	11·6 (-30·6–47·4)	5·9 (-8·2–21·9)	0·59 (-0·11–1·4)	6·8 (-20·5–32·1)
Rwanda	Malaria	23·8 (-16·7–64·8)	23·8 (-16·7–64·8)	--	--
Rwanda	Measles	7·1 (-3·2–20·4)	--	3·7 (-1·3–9·8)	3·6 (-1·7–12·3)
Somalia	All causes	9·4 (6·6–12·6)	9·1 (6·8–11·9)	8·6 (6·6–11·1)	0·22 (-0·24–0·76)
Somalia	Diarrheal diseases	26·3 (-19·1–61·0)	16·6 (-4·4–37·5)	7·4 (-5·3–20·5)	7·1 (-8·4–22·9)
Somalia	Lower respiratory infections	14·7 (-32·1–54·4)	9·0 (-12·7–33·2)	2·4 (-0·47–5·4)	6·0 (-16·6–28·6)
Somalia	Malaria	26·9 (-21·5–69·7)	26·9 (-21·5–69·7)	--	--
Somalia	Measles	13·1 (-5·4–33·1)	--	10·7 (-4·0–26·3)	3·0 (-1·3–10·0)
South Sudan	All causes	17·1 (11·7–22·8)	16·7 (12·0–22·1)	14·5 (11·1–18·1)	0·26 (-0·31–0·85)

South Sudan	Diarrheal diseases	28·1 (-20·0–63·1)	18·6 (-5·2–41·0)	8·2 (-5·8–22·4)	6·7 (-7·7–21·0)
South Sudan	Lower respiratory infections	16·9 (-36·7–58·0)	10·1 (-16·0–36·2)	4·2 (-0·81–9·5)	6·1 (-16·8–28·2)
South Sudan	Malaria	29·9 (-28·4–74·1)	29·9 (-28·4–74·1)	--	--
South Sudan	Measles	16·2 (-6·8–40·6)	--	13·2 (-4·9–32·3)	3·9 (-1·8–12·9)
Uganda	All causes	3·3 (-0·59–8·1)	3·1 (-0·29–7·5)	1·1 (0·83–1·5)	0·17 (-0·20–0·59)
Uganda	Diarrheal diseases	21·0 (-14·3–51·8)	13·3 (-3·3–31·5)	3·5 (-2·2–10·0)	6·9 (-8·1–22·3)
Uganda	Lower respiratory infections	11·2 (-26·2–44·9)	6·1 (-8·5–22·9)	0·91 (-0·17–2·1)	5·7 (-16·1–27·1)
Uganda	Malaria	23·7 (-16·4–64·8)	23·7 (-16·4–64·8)	--	--
Uganda	Measles	7·3 (-3·0–19·7)	--	5·2 (-1·8–13·4)	2·3 (-0·98–7·9)
United Republic of Tanzania	All causes	2·0 (-0·87–4·6)	1·8 (-0·53–4·1)	0·60 (0·38–0·86)	0·18 (-0·22–0·63)
United Republic of Tanzania	Diarrheal diseases	21·4 (-15·0–52·2)	13·6 (-3·4–31·8)	3·3 (-2·1–9·6)	7·4 (-8·6–23·4)
United Republic of Tanzania	Lower respiratory infections	11·8 (-29·5–47·6)	6·4 (-9·3–24·3)	0·86 (-0·16–2·0)	6·2 (-18·2–29·8)
United Republic of Tanzania	Malaria	25·0 (-18·5–67·1)	25·0 (-18·5–67·1)	--	--
United Republic of Tanzania	Measles	7·6 (-3·2–20·7)	--	4·9 (-1·6–12·4)	3·0 (-1·3–10·2)
Zambia	All causes	2·5 (-0·29–5·5)	2·3 (<0.1–5·0)	0·89 (0·61–1·2)	0·18 (-0·23–0·58)
Zambia	Diarrheal diseases	20·3 (-14·3–50·7)	12·4 (-3·0–29·8)	3·4 (-2·2–9·7)	7·2 (-8·6–23·1)
Zambia	Lower respiratory infections	11·6 (-28·5–46·6)	5·9 (-8·4–22·6)	1·1 (-0·21–2·5)	6·3 (-18·2–29·9)
Zambia	Malaria	24·0 (-17·1–65·4)	24·0 (-17·1–65·4)	--	--
Zambia	Measles	8·5 (-3·6–24·0)	--	5·2 (-1·8–13·4)	3·7 (-1·9–12·2)
Southern Sub-Saharan Africa	All causes	1·4 (0·29–2·6)	1·1 (0·58–1·8)	0·81 (0·59–1·1)	0·24 (-0·30–0·85)
Southern Sub-Saharan Africa	Diarrheal diseases	18·2 (-12·9–46·3)	10·7 (-2·6–25·7)	2·4 (-1·5–7·0)	7·1 (-8·3–22·8)
Southern Sub-Saharan Africa	Lower respiratory infections	10·0 (-23·3–41·2)	4·8 (-6·5–18·3)	0·68 (-0·13–1·6)	5·6 (-15·7–27·1)
Southern Sub-Saharan Africa	Malaria	23·8 (-16·5–65·1)	23·8 (-16·5–65·1)	--	--
Southern Sub-Saharan Africa	Measles	5·4 (-2·2–15·4)	--	3·6 (-1·2–9·4)	2·0 (-0·87–6·6)
Botswana	All causes	2·4 (1·5–3·7)	2·2 (1·6–3·2)	2·1 (1·5–2·9)	0·14 (-0·18–0·51)
Botswana	Diarrheal diseases	19·8 (-13·6–49·5)	11·6 (-2·8–27·8)	4·2 (-2·7–11·8)	6·7 (-7·7–21·4)
Botswana	Lower respiratory infections	11·3 (-23·9–43·7)	5·5 (-7·4–20·7)	1·6 (-0·30–3·7)	5·7 (-15·1–26·6)
Botswana	Malaria	22·8 (-15·4–64·0)	22·8 (-15·4–64·0)	--	--
Botswana	Measles	8·9 (-3·6–24·2)	--	6·3 (-2·1–16·2)	3·0 (-1·3–10·2)

Eswatini	All causes	0·21 (-0·16–0·64)	0·12 (>-0.1–0·33)	<0.1 (>-0.1–<0.1)	<0.1 (-0·11–0·33)
Eswatini	Diarrheal diseases	15·5 (-10·7–40·5)	8·8 (-2·0–21·4)	1·6 (-1·0–4·6)	6·6 (-7·5–21·0)
Eswatini	Lower respiratory infections	8·7 (-19·9–37·0)	3·9 (-4·9–14·8)	0·41 (>-0.1–0·96)	5·3 (-14·2–25·5)
Eswatini	Malaria	18·1 (-10·1–54·6)	18·1 (-10·1–54·6)	--	--
Eswatini	Measles	4·4 (-1·9–12·7)	--	2·4 (-0·79–6·2)	2·1 (-0·97–7·1)
Lesotho	All causes	1·0 (-0·12–2·1)	0·77 (0·23–1·4)	0·45 (0·27–0·69)	0·26 (-0·34–0·89)
Lesotho	Diarrheal diseases	21·4 (-15·6–52·8)	13·4 (-3·3–31·5)	2·7 (-1·7–7·8)	8·2 (-9·8–25·9)
Lesotho	Lower respiratory infections	11·8 (-30·7–47·8)	6·2 (-8·6–22·9)	0·73 (-0·14–1·7)	6·6 (-20·1–31·4)
Lesotho	Malaria	0 (0–0)	0 (0–0)	--	--
Lesotho	Measles	6·8 (-3·0–19·1)	--	4·0 (-1·3–10·6)	3·0 (-1·4–9·8)
Namibia	All causes	2·6 (1·7–3·8)	2·5 (1·7–3·6)	2·3 (1·6–3·3)	<0.1 (-0·11–0·32)
Namibia	Diarrheal diseases	21·1 (-13·6–51·1)	13·3 (-3·3–31·1)	4·9 (-3·2–13·8)	5·8 (-6·6–18·6)
Namibia	Lower respiratory infections	11·3 (-23·2–43·4)	6·4 (-8·9–23·7)	1·5 (-0·29–3·4)	4·9 (-12·7–23·5)
Namibia	Malaria	25·4 (-20·0–67·2)	25·4 (-20·0–67·2)	--	--
Namibia	Measles	8·9 (-3·4–23·3)	--	7·3 (-2·5–18·5)	1·9 (-0·76–6·6)
South Africa	All causes	1·2 (0·17–2·4)	0·95 (0·46–1·6)	0·67 (0·48–0·92)	0·27 (-0·31–0·93)
South Africa	Diarrheal diseases	17·3 (-12·4–44·8)	9·9 (-2·3–24·1)	2·2 (-1·3–6·3)	7·1 (-8·3–22·8)
South Africa	Lower respiratory infections	9·3 (-21·8–39·1)	4·2 (-5·6–16·1)	0·57 (-0·11–1·3)	5·5 (-15·5–26·7)
South Africa	Malaria	18·9 (-11·0–55·0)	18·9 (-11·0–55·0)	--	--
South Africa	Measles	4·8 (-2·0–13·7)	--	3·0 (-0·98–7·9)	1·9 (-0·83–6·3)
Zimbabwe	All causes	1·6 (0·32–3·0)	1·3 (0·60–2·3)	0·92 (0·66–1·3)	0·22 (-0·29–0·76)
Zimbabwe	Diarrheal diseases	20·8 (-14·1–51·2)	13·3 (-3·2–31·1)	3·1 (-2·0–8·8)	7·2 (-8·4–23·0)
Zimbabwe	Lower respiratory infections	11·0 (-25·9–44·2)	5·9 (-8·2–21·9)	0·78 (-0·15–1·8)	5·8 (-16·2–27·6)
Zimbabwe	Malaria	24·1 (-16·8–65·9)	24·1 (-16·8–65·9)	--	--
Zimbabwe	Measles	6·7 (-2·7–18·5)	--	4·7 (-1·7–12·4)	2·1 (-0·89–7·2)
Western Sub-Saharan Africa	All causes	7·3 (1·9–12·0)	7·0 (2·4–11·2)	4·5 (3·4–5·7)	0·24 (-0·29–0·81)
Western Sub-Saharan Africa	Diarrheal diseases	25·9 (-18·7–59·6)	17·4 (-4·9–38·9)	6·0 (-4·1–16·6)	7·1 (-8·5–22·7)
Western Sub-Saharan Africa	Lower respiratory infections	14·2 (-35·0–53·1)	8·9 (-14·0–32·2)	2·1 (-0·40–4·7)	6·1 (-17·6–29·0)
Western Sub-Saharan Africa	Malaria	28·0 (-25·1–71·3)	28·0 (-25·1–71·3)	--	--

Western Sub-Saharan Africa	Measles	11·6 (-4·8–29·9)	--	8·9 (-3·3–22·3)	3·2 (-1·7–10·4)
Benin	All causes	6·0 (-0·74–12·1)	5·8 (-0·42–11·6)	2·5 (1·8–3·2)	0·20 (-0·25–0·68)
Benin	Diarrheal diseases	24·4 (-17·6–57·5)	16·1 (-4·3–36·5)	4·5 (-2·9–12·5)	7·7 (-9·2–24·4)
Benin	Lower respiratory infections	13·0 (-32·2–49·8)	7·6 (-11·1–27·4)	1·3 (-0·25–3·0)	6·2 (-18·6–29·1)
Benin	Malaria	27·2 (-22·4–70·2)	27·2 (-22·4–70·2)	--	--
Benin	Measles	9·2 (-3·7–24·1)	--	6·9 (-2·4–17·1)	2·6 (-1·1–8·9)
Burkina Faso	All causes	7·2 (3·0–11·5)	7·0 (3·1–11·1)	4·8 (3·6–6·2)	0·12 (-0·15–0·39)
Burkina Faso	Diarrheal diseases	25·8 (-17·9–59·5)	17·4 (-4·8–38·9)	6·1 (-4·1–17·1)	6·7 (-7·8–21·4)
Burkina Faso	Lower respiratory infections	13·9 (-32·2–52·3)	8·7 (-13·3–31·7)	2·0 (-0·37–4·7)	5·6 (-15·8–27·1)
Burkina Faso	Malaria	29·0 (-25·8–72·8)	29·0 (-25·8–72·8)	--	--
Burkina Faso	Measles	11·3 (-4·4–29·4)	--	9·4 (-3·5–23·4)	2·2 (-1·1–7·6)
Cabo Verde	All causes	0·43 (-0·25–1·1)	0·28 (>-0.1–0·70)	<0.1 (>-0.1–0·29)	0·11 (-0·13–0·36)
Cabo Verde	Diarrheal diseases	19·0 (-12·2–47·3)	11·9 (-3·0–28·5)	4·2 (-2·7–11·9)	5·2 (-5·7–16·9)
Cabo Verde	Lower respiratory infections	9·4 (-19·7–36·3)	5·2 (-7·2–19·2)	0·99 (-0·20–2·4)	4·2 (-11·5–19·6)
Cabo Verde	Malaria	24·6 (-17·1–66·5)	24·6 (-17·1–66·5)	--	--
Cabo Verde	Measles	6·7 (-2·3–17·8)	--	6·3 (-2·2–16·7)	0·43 (-0·15–1·7)
Cameroon	All causes	4·0 (-0·90–9·1)	3·6 (-0·36–7·9)	1·3 (0·91–1·8)	0·32 (-0·36–1·1)
Cameroon	Diarrheal diseases	20·5 (-14·0–50·5)	12·7 (-3·1–29·9)	3·6 (-2·3–10·1)	6·8 (-8·0–21·8)
Cameroon	Lower respiratory infections	11·4 (-25·9–44·6)	6·2 (-8·2–22·7)	1·1 (-0·21–2·6)	5·7 (-16·0–26·8)
Cameroon	Malaria	23·4 (-16·0–64·0)	23·4 (-16·0–64·0)	--	--
Cameroon	Measles	7·9 (-3·2–21·5)	--	5·6 (-1·9–14·5)	2·6 (-1·0–8·6)
Chad	All causes	12·5 (5·6–19·0)	11·8 (6·7–17·4)	9·4 (6·9–12·4)	0·58 (-0·73–1·9)
Chad	Diarrheal diseases	27·5 (-19·9–62·6)	18·5 (-5·2–40·9)	6·9 (-4·8–19·1)	7·2 (-8·7–22·7)
Chad	Lower respiratory infections	15·1 (-34·1–55·2)	9·4 (-14·4–34·4)	2·7 (-0·52–6·2)	5·9 (-16·4–28·1)
Chad	Malaria	29·7 (-27·5–73·9)	29·7 (-27·5–73·9)	--	--
Chad	Measles	13·8 (-5·6–35·8)	--	11·1 (-4·0–27·7)	3·3 (-1·7–11·2)
Côte d'Ivoire	All causes	4·0 (-0·68–9·3)	3·8 (-0·40–9·0)	1·4 (0·95–1·9)	0·13 (-0·15–0·42)
Côte d'Ivoire	Diarrheal diseases	21·8 (-14·2–52·6)	14·0 (-3·5–32·6)	4·7 (-3·1–13·2)	6·1 (-7·0–19·4)
Côte d'Ivoire	Lower respiratory infections	11·4 (-23·8–43·6)	6·5 (-9·1–24·0)	1·4 (-0·27–3·2)	5·0 (-13·1–23·8)

Côte d'Ivoire	Malaria	24·7 (-18·0–66·4)	24·7 (-18·0–66·4)	--	--
Côte d'Ivoire	Measles	8·8 (-3·4–23·2)	--	7·2 (-2·5–18·3)	1·8 (-0·80–6·4)
Gambia	All causes	3·5 (1·7–5·5)	3·3 (1·9–5·2)	2·7 (1·9–3·7)	0·11 (-0·13–0·39)
Gambia	Diarrheal diseases	23·0 (-14·9–55·0)	15·3 (-4·0–35·4)	5·2 (-3·5–14·9)	5·9 (-6·7–19·4)
Gambia	Lower respiratory infections	11·8 (-25·4–45·8)	7·2 (-10·7–27·0)	1·4 (-0·27–3·1)	4·9 (-12·8–23·9)
Gambia	Malaria	27·0 (-22·2–70·0)	27·0 (-22·2–70·0)	--	--
Gambia	Measles	8·8 (-3·3–22·3)	--	7·8 (-2·8–19·6)	1·2 (-0·51–3·9)
Ghana	All causes	3·4 (0·64–6·6)	3·2 (0·81–6·3)	1·9 (1·3–2·5)	<0.1 (>-0.1–0·27)
Ghana	Diarrheal diseases	21·0 (-13·2–51·4)	13·5 (-3·4–31·9)	5·0 (-3·3–14·1)	5·3 (-6·0–17·6)
Ghana	Lower respiratory infections	10·9 (-21·4–42·4)	6·3 (-8·8–23·9)	1·4 (-0·29–3·3)	4·5 (-11·4–22·1)
Ghana	Malaria	24·6 (-17·7–66·3)	24·6 (-17·7–66·3)	--	--
Ghana	Measles	8·7 (-3·4–23·0)	--	7·6 (-2·8–19·4)	1·3 (-0·56–4·6)
Guinea	All causes	5·4 (0·78–10·0)	5·2 (0·99–9·4)	3·1 (2·4–4·0)	0·15 (-0·18–0·52)
Guinea	Diarrheal diseases	24·3 (-16·5–57·4)	15·9 (-4·2–36·4)	5·4 (-3·6–14·9)	6·9 (-8·0–21·9)
Guinea	Lower respiratory infections	13·2 (-29·3–49·6)	7·7 (-11·2–28·2)	1·7 (-0·32–3·9)	5·8 (-15·8–27·5)
Guinea	Malaria	27·1 (-22·2–70·0)	27·1 (-22·2–70·0)	--	--
Guinea	Measles	10·9 (-4·4–28·2)	--	8·3 (-2·9–20·6)	3·1 (-1·5–10·7)
Guinea-Bissau	All causes	2·7 (0·77–5·4)	2·5 (0·95–5·1)	1·6 (1·1–2·3)	0·15 (-0·18–0·51)
Guinea-Bissau	Diarrheal diseases	24·5 (-17·1–58·3)	16·3 (-4·3–37·5)	4·4 (-2·8–12·4)	7·7 (-9·1–24·7)
Guinea-Bissau	Lower respiratory infections	13·0 (-31·5–50·5)	7·7 (-11·5–28·3)	1·2 (-0·23–2·7)	6·2 (-17·8–29·5)
Guinea-Bissau	Malaria	27·6 (-23·1–70·9)	27·6 (-23·1–70·9)	--	--
Guinea-Bissau	Measles	8·6 (-3·4–22·7)	--	6·6 (-2·3–16·7)	2·3 (-1·0–7·7)
Liberia	All causes	4·5 (-1·5–11·0)	4·3 (-1·2–10·6)	1·1 (0·77–1·5)	0·17 (-0·20–0·58)
Liberia	Diarrheal diseases	21·4 (-14·3–52·4)	13·9 (-3·5–32·6)	3·8 (-2·4–10·6)	6·7 (-7·8–21·6)
Liberia	Lower respiratory infections	11·4 (-26·3–45·0)	6·5 (-9·1–23·8)	0·98 (-0·19–2·3)	5·5 (-15·5–26·5)
Liberia	Malaria	25·1 (-18·7–67·2)	25·1 (-18·7–67·2)	--	--
Liberia	Measles	7·8 (-3·0–20·5)	--	5·9 (-2·1–15·1)	2·0 (-0·80–6·9)
Mali	All causes	4·6 (0·70–8·5)	4·3 (1·0–7·9)	2·5 (1·8–3·4)	0·19 (-0·22–0·65)
Mali	Diarrheal diseases	24·7 (-16·6–57·7)	16·4 (-4·4–37·1)	6·2 (-4·2–17·2)	6·2 (-7·2–19·8)

Mali	Lower respiratory infections	13·3 (-27·8–49·7)	8·2 (-12·0–30·1)	1·9 (-0·38–4·4)	5·2 (-13·6–25·4)
Mali	Malaria	27·7 (-23·5–71·1)	27·7 (-23·5–71·1)	--	--
Mali	Measles	11·2 (-4·3–28·4)	--	9·4 (-3·4–23·0)	2·1 (-0·94–7·1)
Mauritania	All causes	5·4 (3·3–7·7)	5·1 (3·4–7·0)	4·6 (3·3–6·2)	0·18 (-0·21–0·61)
Mauritania	Diarrheal diseases	23·9 (-15·5–56·1)	15·8 (-4·2–36·2)	6·2 (-4·2–17·4)	5·6 (-6·3–18·1)
Mauritania	Lower respiratory infections	12·6 (-26·7–46·9)	7·8 (-11·8–28·7)	1·8 (-0·36–4·1)	4·8 (-12·8–23·1)
Mauritania	Malaria	27·9 (-23·8–71·1)	27·9 (-23·8–71·1)	--	--
Mauritania	Measles	10·7 (-4·2–27·4)	--	9·2 (-3·4–23·0)	1·8 (-0·79–6·3)
Niger	All causes	9·2 (3·2–14·2)	8·9 (3·7–13·5)	6·5 (5·0–8·5)	0·28 (-0·36–0·96)
Niger	Diarrheal diseases	32·3 (-25·2–69·8)	23·2 (-7·2–49·2)	7·5 (-5·3–20·7)	8·5 (-10·4–27·0)
Niger	Lower respiratory infections	17·9 (-52·6–65·6)	12·5 (-22·8–44·2)	2·4 (-0·46–5·5)	7·5 (-23·5–35·7)
Niger	Malaria	33·9 (-40·3–79·6)	33·9 (-40·3–79·6)	--	--
Niger	Measles	15·9 (-7·0–40·7)	--	11·7 (-4·6–28·1)	5·3 (-2·8–17·4)
Nigeria	All causes	8·8 (2·6–14·2)	8·5 (3·2–13·4)	5·6 (4·2–7·3)	0·27 (-0·32–0·89)
Nigeria	Diarrheal diseases	26·8 (-19·7–61·0)	18·1 (-5·2–40·2)	6·3 (-4·4–17·3)	7·3 (-8·9–23·5)
Nigeria	Lower respiratory infections	14·7 (-37·3–54·6)	9·3 (-14·8–33·4)	2·2 (-0·43–5·1)	6·3 (-18·6–30·1)
Nigeria	Malaria	28·7 (-26·9–72·0)	28·7 (-26·9–72·0)	--	--
Nigeria	Measles	12·9 (-5·5–33·2)	--	9·6 (-3·6–24·1)	3·9 (-2·1–12·8)
Sao Tome and Principe	All causes	1·5 (0·73–2·4)	1·4 (0·85–2·0)	1·2 (0·78–1·7)	<0.1 (-0·11–0·29)
Sao Tome and Principe	Diarrheal diseases	18·0 (-11·4–45·3)	11·0 (-2·6–26·3)	3·5 (-2·2–10·0)	5·6 (-6·2–17·8)
Sao Tome and Principe	Lower respiratory infections	9·3 (-18·3–36·8)	4·8 (-6·4–18·1)	1·0 (-0·20–2·3)	4·5 (-11·1–21·3)
Sao Tome and Principe	Malaria	20·8 (-12·9–59·8)	20·8 (-12·9–59·8)	--	--
Sao Tome and Principe	Measles	6·6 (-2·4–17·5)	--	5·6 (-1·9–14·5)	1·2 (-0·49–4·1)
Senegal	All causes	2·0 (0·62–3·7)	1·8 (0·89–3·1)	1·4 (0·92–2·0)	0·13 (-0·15–0·46)
Senegal	Diarrheal diseases	21·1 (-13·0–51·5)	13·6 (-3·4–32·2)	5·3 (-3·5–15·2)	5·1 (-5·5–16·6)
Senegal	Lower respiratory infections	11·0 (-20·8–41·7)	6·5 (-9·1–24·3)	1·6 (-0·30–3·5)	4·2 (-10·3–20·5)
Senegal	Malaria	25·1 (-18·4–66·8)	25·1 (-18·4–66·8)	--	--
Senegal	Measles	8·8 (-3·3–22·9)	--	7·8 (-2·8–19·9)	1·2 (-0·54–3·9)
Sierra Leone	All causes	5·4 (<0.1–11·9)	5·1 (0·44–11·0)	2·4 (1·8–3·2)	0·27 (-0·30–0·89)

Sierra Leone	Diarrheal diseases	21·8 (-14·4–52·5)	13·9 (-3·5–32·3)	4·4 (-2·9–12·4)	6·5 (-7·5–20·6)
Sierra Leone	Lower respiratory infections	12·0 (-25·6–46·0)	6·7 (-9·2–24·6)	1·5 (-0·28–3·4)	5·5 (-14·6–26·3)
Sierra Leone	Malaria	24·7 (-18·2–66·4)	24·7 (-18·2–66·4)	--	--
Sierra Leone	Measles	9·4 (-3·6–24·7)	--	7·0 (-2·4–17·3)	2·7 (-1·3–8·9)
Togo	All causes	3·7 (-1·6–8·6)	3·4 (-1·1–7·9)	1·1 (0·68–1·6)	0·27 (-0·30–0·87)
Togo	Diarrheal diseases	23·7 (-15·7–56·4)	15·7 (-4·1–36·1)	5·0 (-3·3–14·2)	6·5 (-7·4–21·2)
Togo	Lower respiratory infections	12·3 (-27·6–46·6)	7·4 (-10·9–26·9)	1·4 (-0·27–3·1)	5·3 (-14·8–24·9)
Togo	Malaria	26·9 (-21·8–69·8)	26·9 (-21·8–69·8)	--	--
Togo	Measles	9·1 (-3·5–23·6)	--	7·8 (-2·8–19·4)	1·6 (-0·67–5·8)

Table S7. Interventions and strategies to reduce the burden of childhood growth failure

Socio-economic factors	During Pregnancy	Postpartum and infancy	Acute malnutrition treatment
Gender equality and programs to give women economic and educational opportunities and social autonomy<br><br>Expanded access to educational opportunities for adolescent girls and prevention of adolescent pregnancy[1]<br><br>Family planning and reproductive health services give women greater decision making over number and timing of children. Birth spacing may result in 10-50% reduction in stunting prevalence[2]<br><br>Poverty alleviation programs including conditional cash transfers can improve linear growth and decrease stunting prevalence by 10%[3,4]<br><br>Prevention of infectious disease episodes, like diarrhea and enteric infections (reduction in childhood growth failure burden of 39%[5]) and malaria (Insecticide treated bed nets reduce low birthweight by up to 23%.[6] Malaria chemoprevention reduces moderate/severe anemia by 40% and 27% reduction in low birthweight)[7]	Supplementation of single and multiple micronutrients to mothers may reduce the prevalence of small-for-gestational age (SGA) and stunting at birth.[8]<br><br>Multiple micronutrient supplementation can reduce low birthweight by 15%, and babies born SGA by 7%.[9]<br><br>Iron supplementation in pregnancy to prevent or treat maternal anemia can reduce the risk of low birthweight by 12%.[9]<br><br>Balanced energy protein supplementation in pregnancy reduces risk of low birthweight and SGA.[10]	Kangaroo mother care, especially for preterm and small for gestational age, can increase weight gain by 40% in the first two weeks of life.[11]<br><br>Prompt initiation of exclusive breastfeeding is important to optimize macronutrient intake for infants and can protect against infections.[12]<br><br>Complementary feeding practices improves WAZ, HAZ, and reduces stunting prevalence.[13]<br><br>Small-quantity lipid-based nutrient supplementation during complementary feeding reduces severe and moderate stunting, moderate wasting, and moderate underweight.[14]<br><br>Supplementary feeding programs reduce wasting prevalence and reduce risk of infant mortality.[13]<br><br>Maternal mental health support[15]	Facility- and community-based strategies to manage severe acute malnutrition (including ready-to-use therapeutic food, supplementary food, antibiotics, and vitamin A) can result in recovery rates of up to 83%.[16]<br><br>Ready to use therapeutic food (RUTF) is extremely effective in treating uncomplicated severe acute malnutrition with recovery proportions of around 90%.[17]

Table S8. Comparison of all-cause population attributable fractions for stunting, underweight, and wasting deaths among children younger than 5 years between current and previous iterations of the Global Burden of Disease study[18] and with the Maternal and Child Nutrition Study Group[19] estimates published in 2013 Current and previous GBD estimates combine burden associated with mild, moderate, and severe forms of CGF: stunting was defined as height-for-age z-score (HAZ) < –1; underweight as weight-for-age z-score (WAZ) < –1; wasting as weight-for-height z-score (WHZ) < –1, according to WHO Child Growth Standards. Estimates from the Maternal and Child Nutrition Study Group focus only on moderate and severe forms, with each indicator defined as z-scores < -2.

CGF Indicator	GBD 2023	GBD 2019	MCNS: UN prevalence	MCNH: NIMS prevalence
**Stunting**	**8.0%**	**4.4%**	**14.7%**	**17.0%**
**Underweight**	**12.3%**	**6.5%**	**14.4%**	**17.0%**
**Wasting**	**9.2%**	**20.1%**	**12.6%**	**11.5%**

References

1 Welch C, Wong CK, Lelijveld N, Kerac M, Wrottesley SV. Adolescent pregnancy is associated with child undernutrition: Systematic review and meta-analysis. *Matern Child Nutr* 2024; **20**: e13569.

2 Dewey KG, Cohen RJ. Does birth spacing affect maternal or child nutritional status? A systematic literature review. *Matern Child Nutr* 2007; **3**: 151–73.

3 Bhutta ZA, Ahmed T, Black RE, *et al*. What works? Interventions for maternal and child undernutrition and survival. *Lancet Lond Engl* 2008; **371**: 417–40.

4 Owusu-Addo E, Cross R. The impact of conditional cash transfers on child health in low- and middle-income countries: a systematic review. *Int J Public Health* 2014; **59**: 609–18.

5 Troeger C, Colombara DV, Rao PC, *et al*. Global disability-adjusted life-year estimates of long-term health burden and undernutrition attributable to diarrhoeal diseases in children younger than 5 years. *Lancet Glob Health* 2018; **6**: e255–69.

6 Gamble C, Ekwaru JP, ter Kuile FO. Insecticide-treated nets for preventing malaria in pregnancy. *Cochrane Database Syst Rev* 2006; **2006**: CD003755.

7 Radeva-Petrova D, Kayentao K, ter Kuile FO, Sinclair D, Garner P. Drugs for preventing malaria in pregnant women in endemic areas: any drug regimen versus placebo or no treatment. *Cochrane Database Syst Rev* 2014; **2014**: CD000169.

8 Lassi ZS, Kedzior SGE, Tariq W, Jadoon Y, Das JK, Bhutta ZA. Effects of preconception care and periconception interventions on maternal nutritional status and birth outcomes in low- and middle-income countries: A systematic review. *Campbell Syst Rev* 2021; **17**: e1156.

9 Oh C, Keats EC, Bhutta ZA. Vitamin and Mineral Supplementation During Pregnancy on Maternal, Birth, Child Health and Development Outcomes in Low- and Middle-Income Countries: A Systematic Review and Meta-Analysis. *Nutrients* 2020; **12**: 491.

10 Lassi ZS, Padhani ZA, Rabbani A, *et al*. Impact of Dietary Interventions during Pregnancy on Maternal, Neonatal, and Child Outcomes in Low- and Middle-Income Countries. *Nutrients* 2020; **12**: 531.

11 Keats EC, Das JK, Salam RA, *et al*. Effective interventions to address maternal and child malnutrition: an update of the evidence. *Lancet Child Adolesc Health* 2021; **5**: 367–84.

12 Kramer MS, Kakuma R. Optimal duration of exclusive breastfeeding. *Cochrane Database Syst Rev* 2012; **2012**: CD003517.

13 Lassi ZS, Rind F, Irfan O, Hadi R, Das JK, Bhutta ZA. Impact of Infant and Young Child Feeding (IYCF) Nutrition Interventions on Breastfeeding Practices, Growth and Mortality in Low- and Middle-Income Countries: Systematic Review. *Nutrients* 2020; **12**: 722.

14 Das JK, Salam RA, Hadi YB, *et al.* Preventive lipid-based nutrient supplements given with complementary foods to infants and young children 6 to 23 months of age for health, nutrition, and developmental outcomes. *Cochrane Database Syst Rev* 2019; **5**: CD012611.

15 Patel V, Rahman A, Jacob KS, Hughes M. Effect of maternal mental health on infant growth in low income countries: new evidence from South Asia. *BMJ* 2004; **328**: 820–3.

16 Das JK, Salam RA, Saeed M, Kazmi FA, Bhutta ZA. Effectiveness of Interventions for Managing Acute Malnutrition in Children under Five Years of Age in Low-Income and Middle-Income Countries: A Systematic Review and Meta-Analysis. *Nutrients* 2020; **12**: 116.

17 Trehan I, Manary MJ. Management of severe acute malnutrition in low-income and middle-income countries. *Arch Dis Child* 2015; **100**: 283–7.

18 GBD 2019 Risk Factors Collaborators. Global burden of 87 risk factors in 204 countries and territories, 1990–2019: a systematic analysis for the Global Burden of Disease Study 2019. *The Lancet* 2020; **396**: 1223–49.

19 Black RE, Victora CG, Walker SP, *et al.* Maternal and child undernutrition and overweight in low-income and middle-income countries. *Lancet Lond Engl* 2013; **382**: 427–51.
