# Supplementary material for: Quantifying the fatal and non-fatal burden of disease associated with child growth failure, 2000–2023: a systematic analysis from the Global Burden of Disease Study 2023
Source: Lancet Child Adolesc Health. 2026 Jan;10(1):22–38. doi: 10.1016/S2352-4642(25)00303-7 (PMC12674951; doi:10.1016/S2352-4642(25)00303-7)
Supplement: Supplementary appendix 2 [file mmc2.pdf]

THE LANCET
Child & Adolescent Health

Supplementary appendix 2

This appendix formed part of the original submission and has been peer reviewed. We post it as supplied by the authors.

Supplement to: GBD 2023 Child Growth Failure Collaborators. Quantifying the fatal and non-fatal burden of disease associated with child growth failure, 2000–2023: a systematic analysis from the Global Burden of Disease Study 2023. *Lancet Child Adolesc Health* 2026; **10:** 22–38.

Appendix 2: Authorship appendix to "Quantifying the fatal and non-fatal burden of disease associated with child growth failure, 2000–2023: a systematic analysis from the Global Burden of Disease Study 2023"

This appendix provides further authorship detail for "Quantifying the fatal and non-fatal burden of disease associated with child growth failure, 2000–2023: a systematic analysis from the Global Burden of Disease Study 2023"

Table of Contents

GBD 2023 Global Child Growth Failure Collaborators **2**

Affiliations **4**

Authors' Contributions **17**

- Managing the overall research enterprise 17
- Writing the first draft of the manuscript 17
- Primary responsibility for applying analytical methods to produce estimates 17
- Primary responsibility for seeking, cataloguing, extracting, or cleaning data; designing or coding figures and tables 17
- Providing data or critical feedback on data sources 18
- Developing methods or computational machinery 19
- Providing critical feedback on methods or results 20
- Drafting the work or revising it critically for important intellectual content 23
- Managing the estimation or publications process 24

GBD 2023 Global Child Growth Failure Collaborators

Christopher E Troeger*, Michael Benjamin Arndt*, Hasan Aalruz, Meriem Abdoun, Auwal Abdullahi, Mesfin Abebe, Armita Abedi, Alemwork Abie, Richard Gyan Aboagye, Hassan Abolhassani, Yonas Derso Abtew, Ahmed Abu-Zaid, Lawan Hassan Adamu, Mesafint Molla Adane, Isaac Yeboah Addo, Oyelola A Adegboye, Victor Adekanmbi, Juliana Bunmi Adetunji, Qorinah Estiningtyas Sakilah Adnani, Leticia Akua Adzigbli, Muhammad Sohail Afzal, Saira Afzal, Navidha Aggarwal, Aqeel Ahmad, Muayyad M Ahmad, Sajjad Ahmad, Elham Ahmadi, Ayman Ahmed, Haroon Ahmed, Mehrunnisha Sharif Ahmed, Mushood Ahmed, Marjan Ajami, Budi Aji, Syed Mahfuz Al Hasan, Omar Al Omari, Mohammad Khursheed Alam, Mohammed Albashtawy, Fentahun Alemnew, Ayman Al-Eyadhy, Mohammed Usman Ali, Rafat Ali, Syed Shujait Ali, Waad Ali, Joseph Uy Almazan, Hesham M Al-Mekhlafi, Mohammed A Alsabri, Najim Z Alshahrani, Awais Altaf, Nelson Alvis-Guzman, Mohammad Al-Wardat, Hany Aly, Dickson A Amugsi, Abhishek Anil, Zelalem Alamrew Anteneh, Boluwatife Stephen Anuoluwa, Saeid Anvari, Anayochukwu Edward Anyasodor, Jalal Arabloo, Aleksandr Y Aravkin, Demelash Areda, Mahwish Arooj, Anton A Artamonov, Ashokan Arumugam, Nurila Aryntayeva, Bernard Kwadwo Yeboah Asiamah-Asare, Seyyed Shamsadin Athari, Maha Moh'd Wahbi Atout, Amlaku Mulat Aweke, Adedapo Wasiu Awotidebe, Asteray Assmie Ayenew, Melkalem Mamuye Azanaw, Shahkaar Aziz, Giridhara Rathnaiah Babu, Ruhai Bai, Jennifer L Baker, Wondu Feyisa Balcha, Palash Chandra Banik, Mainak Bardhan, Amadou Barrow, Shahid Bashir, Afisu Basiru, Quique Bassat, Mohammad-Mahdi Bastan, Priyamadhaba Behera, Michelle L Bell, Maryam Bemanalizadeh, Ajeet Singh Bhadoria, Sonu Bhaskar, Priyadarshini Bhattacharjee, Jasvinder Singh Bhatti, Catherine Bisignano, Bijit Biswas, Trupti Bodhare, Srinivasa Rao Bolla, Sri Harsha Boppana, Angelo Capodici, Rama Mohan Chandika, Vijay Kumar Chattu, Anis Ahmad Chaudhary, Moges Sisay Chekole, Hana Chen, Daniel Youngwhan Cho, Sonali Gajanan Choudhari, Isaac Sunday Chukwu, Erin Chung, Natalia Cruz-Martins, Alanna Gomes da Silva, Tukur Dahiru, Xiaochen Dai, Lalit Dandona, Rakhi Dandona, Samuel Demissie Darcho, Amira Hamed Darwish, Fernando Pio De la Hoz, Edgar Denova-Gutiérrez, Vinoth Gnana Chellaiyan Devanbu, Devananda Devegowda, Adriana Dima, Thanh Chi Do, Robert Kokou Dowou, Angel Belle Cheng Dy, Ibrahim Farahat El Bayoumy, Marwa Eldegwi, Muhammed Elhadi, Legesse Tesfaye Elilo, Iman El Sayed, Abidemi Omolara Fasanmi, Marta Figueiredo, Florian Fischer, Artem Alekseevich Fomenkov, Amin Fraij, Amanuel Tesfay Gebremedhin, Lemma Getacher, Genanew K Getahun, Maryam Gholamalizadeh, Nora M Gilbertson, Alem Abera Girmay, Mahaveer Golechha, Dinorah Gonzalez-Castell, Michal Grivna, Shi-Yang Guan, Mohammed Ibrahim Mohialdeen Gubari, Damitha Asanga Gunawardane, Zhifeng Guo, Bhawna Gupta, Rajat Das Gupta, Demewoz Haile, Nadia M Hamdy, Alexis J Handal, Nasrin Hanifi, Habtamu Endashaw Hareru, Eka Mishbahatul Marah Has, Ahmed I Hasaballah, Ikrama Hassan, Simon I Hay, Khezar Hayat, Jiawei He, Behzad Heibati, Austin Heuer, Kamal Hezam, Ramesh Holla, Md Sabbir Hossain, Hassan Hosseinzadeh, Sorin Hostiuc, Tanvir M Huda, Javid Hussain, Dursa Hussein, Hong-Han Huynh, Bing-Fang Hwang, Segun Emmanuel Ibitoye, Mustapha Immurana, Teresa R Iskander, Md Rabiul Islam, Md Sahidul Islam, Sheikh Mohammed Shariful Islam, Louis Jacob, Mihajlo Jakovljevic, Shubha Jayaram, Achala Upendra Jayatilleke, Wenyi Jin, Alex Joseph, Nitin Joseph, Ali Kabir, Vidya Kadashetti, Dler H Hussein Kadir, Sanjay Kalra, Arun Kamireddy, Kehinde Kazeem Kanmodi, Rami S Kantar, Faizan Zaffar Kashoo, Gbenga A Kayode, Shemsu Kedir, Tibebeselassie S Keflie, Ajmal Khan, Maseer Khan, Vishnu Khanal, Shaghayegh Khanmohammadi, Khaled Khatab, Moawiah Mohammad Khatatbeh, Mahalaqua Nazli Khatib, Feriha Fatima Khidri, Kwanghyun Kim, Min Seo Kim, Adnan Kisa, Farzad Kompani, Isaac Koomson, Kewal Krishan, Mukhtar Kulimbet, Dewesh Kumar, G Anil Kumar, Nithin Kumar, Vijay Kumar, Almagul Kurmanova, Maria Dyah Kurniasari, Dian Kusuma, Chandrakant Lahariya, Kamaluddin Latief, Minh Huu Nhat Le, Nhi Huu Hanh Le, Sang-woong

Lee, Seung Won Lee, Yo Han Lee, Virendra S Ligade, Stephen S Lim, Jialing Lin, Jue Liu, Xuefeng Liu, Rakesh Lodha, José Francisco López-Gil, Surbala Devi Lourembam, Zheng Feei Ma, Mahmoud Mabrok, Kashish Malhotra, Ahmad Azam Malik, Vahid Mansouri, Emmanuel Manu, Melvin Barrientos Marzan, Roy Rillera Marzo, Sammer Marzouk, Medha Mathur, Rita Mattiello, Rishi P Mediratta, Riffat Mehboob, Kala M Mehta, Tesfahun Mekene Meto, Tomislav Mestrovic, Sachith Mettananda, Tomasz Miazgowski, Giuseppe Minervini, Mojgan Mirghafourvand, Andreea Mirica, Jama Mohamed, Nouh Saad Mohamed, Sakineh Mohammad-Alizadeh-Charandabi, Abdollah Mohammadian-Hafshejani, Shafiu Mohammed, Ali H Mokdad, Lorenzo Monasta, Mohammad Ali Moni, Sumaira Mubarik, Sumoni Mukherjee, Sileshi Mulatu, Francesk Mulita, Christopher J L Murray, Ghulam Mustafa, Ayoub Nafei, Ganesh R Naik, Zuhair S Natto, Javaid Nauman, Samidi Nirasha Kumari Navaratna, Biswa Prakash Nayak, Amanuel Tebabal Nega, Samata Nepal, Henok Biresaw Netsere, Georges Nguefack-Tsague, Dang Nguyen, The Phuong Nguyen, Robina Khan Niazi, Ali Nikoobar, Lawrence Achilles Nnyanzi, Shuhei Nomura, Mehran Nouri, Chisom Adaobi Nri-Ezedi, Dieta Nurrika, Sylvester Dodzi Nyadanu, Chimezie Igwegbe Nzoputam, Ogochukwu Janet Nzoputam, Ismail A Odetokun, Hassan Okati-Aliabad, Akinkunmi Paul Okekunle, Osaretin Christabel Okonji, Bolajoko Olubukunola Olusanya, Jacob Olusegun Olusanya, Uchechukwu Levi Osuagwu, Amel Ouyahia, Mahesh P A, Jagadish Rao Padubidri, Anca Pantea Stoian, Romil R Parikh, Jay Patel, Shankargouda Patil, Shrikant Pawar, Gavin Pereira, Arokiasamy Perianayagam, Fanny Emily Petermann-Rocha, Hoang Nhat Pham, Hoang Tran Pham, My Kieu Phan, Jalandhar Pradhan, Pranil Man Singh Pradhan, Akila Prashant, Jagadeesh Puvvula, Ibrahim Qattea, Pankaja Raghav, Md. Mosfequr Rahman, Mosiur Rahman, Muhammad Aziz Rahman, Amir Masoud Rahmani, Masoud Rahmati, Rajesh Kumar Rai, Ivano Raimondo, Sathish Rajaa, Rayan Rajabi, Mahmoud Mohammed Ramadan, Chitra Ramasamy, Shakthi Kumaran Ramasamy, Chhabi Lal Ranabhat, Chythra R Rao, Sowmya J Rao, Davide Rasella, Mamunur Rashid, Devarajan Rathish, Santosh Kumar Rauniyar, David Laith Rawaf, Salman Rawaf, Nazila Rezaei, Mohsen Rezaeian, Thales Philipe Rodrigues da Silva, Jefferson Antonio Buendia Rodriguez, Peter Rohloff, Debby Syahru Romadlon, Bedanta Roy, Shubhanjali Roy, Cameron John Sabet, Kabir P Sadarangani, Basema Ahmad Saddik, Umar Saeed, Amene Saghazadeh, Dominic Sagoe, Narjes Saheb Sharif-Askari, Amirhossein Sahebkar, Pragyan Monalisa Sahoo, Yoseph Leonardo Samodra, Abdallah M Samy, Rama Krishna Sanjeev, Senthilkumar Sankararaman, Milena M Santric-Milicevic, Jacob Owusu Sarfo, Yaser Sarikhani, Tanmay Sarkar, Gargi Sachin Sarode, Sachin C Sarode, Benn Sartorius, Jennifer Saulam, Monika Sawhney, Ganesh Kumar Saya, Christophe Schinckus, Art Schuermans, Ashenafi Kibret Sendekie, Subramanian Senthilkumaran, Yashendra Sethi, Allen Seylani, Shazlin Shaharudin, Samiah Shahid, Masood Ali Shaikh, Sunder Sham, Muhammad Aaqib Shamim, Mohd Shanawaz, Mohammed Shannawaz, Nigussie Tadesse Sharew, Vishal Sharma, Pavanchand H Shetty, Aminu Shittu, Ivy Shiue, Seyed Afshin Shorofi, Emmanuel Edwar Siddig, Mithun Sikdar, Luís Manuel Lopes Rodrigues Silva, Harmanjit Singh, Jasvinder A Singh, Kalpana Singh, Surjit Singh, Shipra Solanki, Mansi Soni, Reed J D Sorensen, Muhammad Suleman, Desy Sulistiyorini, Chandan Kumar Swain, Seyyed Mohammad Tabatabaei, Seyed-Amir Tabatabaeizadeh, Mohammad Tabish, Jacques Lukenze Tamuzi, Birhan Tsegaw Taye, Wegayehu Zeneb Teklehaimanot, Abainash Tekola, Mohamad-Hani Temsah, Rekha Thapar, Jansje Henny Vera Ticoalu, Tenaw Yimer Tiruye, Mariya Vladimirovna Titova, Sojit Tomo, Marcos Roberto Tovani-Palone, Quynh Thuy Huong Tran, Thang Huu Tran, Nguyen Tran Minh Duc, Aristidis Tsatsakis, Abdul Rohim Tualeka, Saeed Ullah, Shahid Ullah, Muhammad Umair, Bhaskaran Unnikrishnan, Era Upadhyay, Jibrin Sammani Usman, Jef Van den Eynde, Siavash Vaziri, Balachandar Vellingiri, Vasily Vlassov, Gebeyaw Biset Wagaw, Yanzhong Wang, Felicia Wu, Hong Xiao, Vikas Yadav, Galal Yahya, Dong Keon Yon, Naohiro Yonemoto, Chuanhua Yu, Sojib Bin Zaman, Iman Zare, Michael Zastrozhin,

Mohammed G M Zeariya, Salih M Mustafa Salih Zebari, Claire Chenwen Zhong, Nicholas J Kassebaum, Robert C Reiner Jr.^

*Lead authors

^Senior author

Affiliations

Institute for Health Metrics and Evaluation (C E Troeger PhD, M B Arndt PhD, A Y Aravkin PhD, C Bisignano MPH, E Chung MD, X Dai PhD, Prof L Dandona MD, Prof R Dandona PhD, N M Gilbertson PhD, D Haile PhD, Prof S I Hay FMedSci, J He MSc, A Heuer MSc, Prof S S Lim PhD, T Mestrovic PhD, Prof A H Mokdad PhD, Prof C J L Murray Dphil, R J D Sorensen PhD, N J Kassebaum MD, R C Reiner Jr. PhD), Department of Health Metrics Sciences, School of Medicine (C E Troeger PhD, A Y Aravkin PhD, X Dai PhD, Prof R Dandona PhD, N M Gilbertson PhD, Prof S I Hay FMedSci, Prof S S Lim PhD, Prof A H Mokdad PhD, Prof C J L Murray DPhil, R C Reiner Jr. PhD, B Sartorius PhD, N J Kassebaum MD), Department of Global Health (M B Arndt PhD, R J D Sorensen PhD), Department of Applied Mathematics (A Y Aravkin PhD), Department of Pediatrics (E Chung MD), Department of Anesthesiology & Pain Medicine (N J Kassebaum MD), University of Washington, Seattle, WA, USA; Department of Nursing (H Aalruz PhD), Al Zaytoonah University of Jordan, Amman, Jordan; Department of Medicine (Prof M Abdoun PhD), Department of Health (Prof M Abdoun PhD), University of Setif Algeria, Sétif, Algeria; Department of Physiotherapy (A Abdullahi PhD, A W Awotidebe PhD, J S Usman PhD), Department of Anatomy (L H Adamu PhD), Bayero University Kano, Kano, Nigeria; Department of Physiotherapy (A Abdullahi PhD), Federal University Wukari, Wukari, Nigeria; Department of Midwifery (M Abebe MSc), School of Public Health (H Hareru MSc), Dilla University, Dilla, Ethiopia; Department of Emergency Medicine (A Abedi MD), Department of Immunology (S Athari PhD), Department of Critical Care and Emergency Nursing (N Hanifi PhD), Zanjan University of Medical Sciences, Zanjan, Iran; Department of Midwifery (A Abie MSc, F Alemnew MSc, A M Aweke MSc, A A Ayenew MSc, W F Balcha MSc, A T Nega MSc), College of Medicine and Health Sciences (M M Adane PhD, H B Netsere MSc), Department of Epidemiology (Z A Anteneh MPH), Department of Pediatrics and Child Health Nursing (S Mulatu MSc), Bahir Dar University, Bahir Dar, Ethiopia; Department of Family and Community Health (R G Aboagye MPH), Department of Epidemiology and Biostatistics (L A Adzigbli BSc, R K Dowou MPhil), Institute of Health Research (M Immurana PhD), Department of Population and Behavioural Sciences (E Manu PhD), University of Health and Allied Sciences, Ho, Ghana; School of Population Health (R G Aboagye MPH, Prof B A Saddik PhD), Centre for Social Research in Health (I Y Addo PhD), International Centre for Future Health Systems (J Lin PhD), University of New South Wales, Sydney, NSW, Australia; Research Center for Immunodeficiencies (H Abolhassani PhD, A Saghazadeh MD), School of Medicine (E Ahmadi MD, S Khanmohammadi MD), Non-communicable Diseases Research Center (M Bastan MD, N Rezaei MD), Department of Pediatric Neurology (M Bemanalizadeh MD), Children's Medical Center (Prof F Kompani MD), Digestive Diseases Research Institute (V Mansouri MD), Tehran University of Medical Sciences, Tehran, Iran; Department of Medical Biochemistry and Biophysics (H Abolhassani PhD), Karolinska Institute, Stockholm, Sweden; Department of Biomedical Science (Y D Abtew MSc), Department of Public Health (T Mekene Meto MPH), Arba Minch University, Arba Minch, Ethiopia; Department of Biochemistry and Molecular Medicine (A Abu-Zaid PhD), Alfaisal University, Riyadh, Saudi Arabia; College of Graduate Health Sciences (A Abu-Zaid PhD), University of Tennessee, Memphis, TN, USA; Department of Human Anatomy

(L H Adamu PhD), Federal University Dutse, Dutse, Nigeria; School of Medicine (I Y Addo PhD), School of Public Health (T M Huda PhD), University of Sydney, Sydney, NSW, Australia; Menzies School of Health Research (Prof O A Adegboye PhD), Charles Darwin University, Darwin, NT, Australia; Department of Obstetrics and Gynecology (V Adekanmbi PhD), University of Texas Medical Branch, Galveston, TX, USA; Department of Biochemistry (J B Adetunji PhD), Osun State University, Osogbo, Nigeria; Department of Public Health (Q Adnani PhD), Universitas Padjadjaran (Padjadjaran University), Bandung, Indonesia; Department of Life Sciences (M S Afzal PhD, Prof M Umair PhD), University of Management and Technology, Lahore, Pakistan; Department of Community Medicine (Prof S Afzal PhD), King Edward Memorial Hospital, Lahore, Pakistan; Department of Public Health (Prof S Afzal PhD), Public Health Institute, Lahore, Pakistan; MM College of Pharmacy (N Aggarwal PhD), Maharishi Markandeshwar (Deemed to be University), Ambala, India; College of Medicine (A Ahmad PhD, Prof G Mustafa MD, M Tabish MPharm), Shaqra University, Shaqra, Saudi Arabia; School of Nursing (Prof M M Ahmad PhD), University of Jordan, Amman, Jordan; Department of Health and Biological Sciences (S Ahmad PhD), Abasyn University, Peshawar, Pakistan; Department of Natural Sciences (S Ahmad PhD), Lebanese American University, Beirut, Lebanon; Institute of Endemic Diseases (A Ahmed MSc), Unit of Basic Medical Sciences (E E Siddig MD), University of Khartoum, Khartoum, Sudan; Swiss Tropical and Public Health Institute (A Ahmed MSc), University of Basel, Basel, Switzerland; Department of Biosciences (H Ahmed PhD), COMSATS Institute of Information Technology, Islamabad, Pakistan; College of Nursing (M S Ahmed MSc), Majmaah University, Al Majmaah, Saudi Arabia; Department of Medicine (M Ahmed MBBS), Rawalpindi Medical University, Rawalpindi, Pakistan; National Nutrition and Food Technology Research Institute (M Ajami PhD), Cancer Research Center (M Gholamalizadeh PhD), Social Determinants of Health Research Center (A Nikoobar BSc), Shahid Beheshti University of Medical Sciences, Tehran, Iran; Faculty of Medicine and Public Health (B Aji DrPH), Jenderal Soedirman University, Purwokerto, Indonesia; Division of Public Health Sciences (S Al Hasan PhD), Washington University in St. Louis, St. Louis, MO, USA; Fundamentals and Administration Department (Prof O Al Omari PhD), Department of Geography (W Ali PhD), Sultan Qaboos University, Muscat, Oman; Preventive Dentistry Department (Prof M K Alam PhD), Jouf University, Sakaka, Saudi Arabia; Department of Community and Mental Health (Prof M Albashtawy PhD), Al al-Bayt University, Mafraq, Jordan; Pediatric Intensive Care Unit (A Al-Eyadhy MD, Prof M Temsah MD), Research Chair for Evidence-Based Health Care and Knowledge Translation (Prof M Temsah MD), King Saud University, Riyadh, Saudi Arabia; Department of Medical Rehabilitation (Physiotherapy) (M U Ali PhD), University of Maiduguri, Maiduguri, Nigeria; Nethersole School of Nursing (M U Ali PhD), Jockey Club School of Public Health and Primary Care (C Zhong PhD), The Chinese University of Hong Kong, Hong Kong, China; Department of Biosciences (R Ali PhD), Jamia Millia Islamia, New Delhi, India; Center for Biotechnology and Microbiology (S S Ali PhD, M Suleman PhD), University of Swat, Swat, Pakistan; Department of Medicine (J U Almazan PhD), Department of Biomedical Sciences (S Bolla PhD), Nazarbayev University, Astana, Kazakhstan; Department of Parasitology (Prof H M Al-Mekhlafi PhD), University of Malaya, Kuala Lumpur, Malaysia; Department of Parasitology (Prof H M Al-Mekhlafi PhD), Sana'a University, Sana'a, Yemen; Department of Emergency Medicine (M A Alsabri MD), Sana'a University, Sanaa, Yemen; Pediatric Emergency Medicine Department (M A Alsabri MD), Drexel University, Philadelphia, PA, USA; Department of Family and Community Medicine (N Z Alshahrani MD), University of Jeddah, Jeddah, Saudi Arabia; Institute of Molecular Biology and Biotechnology (A Altaf PhD, S Shahid PhD), University College of Medicine & Dentistry (Prof M Arooj PhD), University Institute of Food Science and Technology (S Bashir PhD), Research Centre for Health Sciences (RCHS) (S Shahid PhD), The University of Lahore,

Lahore, Pakistan; Faculty of Health Sciences (A Altaf PhD), Equator University of Science and Technology, Uganda, Masaka, Uganda; Research Group in Health Economics (Prof N Alvis-Guzman PhD), Universidad de Cartagena (University of Cartagena), Cartagena, Colombia; Research Group in Hospital Management and Health Policies (Prof N Alvis-Guzman PhD), Universidad de la Costa (University of the Coast), Barranquilla, Colombia; Department of Rehabilitation Sciences (M Al-Wardat PhD), Jordan University of Science and Technology, Irbid, Jordan; Department of Pediatrics (Prof H Aly MD), Lerner Research Institute (Prof X Liu PhD), Cleveland Clinic, Cleveland, OH, USA; Department of Health and Wellbeing (D A Amugsi PhD), African Population and Health Research Center, Nairobi, Kenya; Department of Pharmacology (A Anil MD), Department of Community Medicine and Family Medicine (P Behera MD), All India Institute of Medical Sciences, Bhubaneswar, India; Department of Environmental and Occupational Health (B S Anuoluwa MPH), University of Medical Sciences, Ondo, Ondo, Nigeria; Regenerative Medicine, Organ Procurement and Transplantation Multi-disciplinary Center (S Anvari MD), Guilan University of Medical Sciences, Rasht, Iran; Rural Health Research Institute (A E Anyasodor PhD), Charles Sturt University, Orange, NSW, Australia; Health Management and Economics Research Center (J Arabloo PhD), School of Medicine (M Bastan MD), Minimally Invasive Surgery Research Center (A Kabir MD), Department of Medicine (R Rajabi MD), Iran University of Medical Sciences, Tehran, Iran; College of Art and Science (D Areda PhD), Ottawa University, Surprise, AZ, USA; School of Life Sciences (D Areda PhD), Arizona State University, Tempe, AZ, USA; Institute for Biomedical Problems (A A Artamonov PhD), K.A. Timiryazev Institute of Plant Physiology (M V Titova PhD), Russian Academy of Sciences, Moscow, Russia; Department of Physiotherapy (A Arumugam PhD), MBBS (A Fraij BSc), Department of Clinical Sciences (Prof M M Ramadan PhD), Clinical Sciences Department (N Saheb Sharif-Askari PhD), University of Sharjah, Sharjah, United Arab Emirates; Department of Physiotherapy (A Arumugam PhD), Kasturba Medical College, Mangalore (R Holla MD), Department of Pharmaceutical Regulatory Affairs and Management (V S Ligade PhD), Department of Community Medicine (C R Rao MD), Kasturba Medical College Mangalore (Prof B Unnikrishnan MD), Manipal Academy of Higher Education, Manipal, India; Department of Public Health (N Aryntayeva MSPH), Research and Publication Activity Division (M Kulimbet MSc), Kazakh National Medical University, Almaty, Kazakhstan; Department of Clinical Disciplines (N Aryntayeva MSPH), Department of Clinical Subjects (A Kurmanova MD), Al Farabi Kazakh National University, Almaty, Kazakhstan; School of Health and Social Development (B K Y Asiamah-Asare PhD), Deakin University, Melbourne, VIC, Australia; Faculty of Nursing (M M W Atout PhD), Philadelphia University, Amman, Jordan; School of Nursing and Public Health (A W Awotidebe PhD), University of KwaZulu-Natal, Durban, South Africa; College of Medicine and Public Health (G R Naik PhD, S Ullah PhD), Department of Nursing and Health Sciences (S Shorofi PhD), Flinders University, Adelaide, SA, Australia (M M Azanaw MPH); Institute of Biotechnology and Genetic Engineering (S Aziz MS), The University of Agriculture, Peshawar, Pakistan; Department of Population Medicine (Prof G Babu PhD), Social and Economic Survey Research Institute (SESRI) (Prof A Perianayagam PhD), Qatar University, Doha, Qatar; Clinical Research Center (Prof R Bai MD), Nanjing Children's Hospital, Nanjing, China; Center for Clinical Research and Prevention (J L Baker PhD), Bispebjerg University Hospital, Frederiksberg, Denmark; Department of Non-communicable Diseases (P C Banik MPhil), Bangladesh University of Health Sciences, Dhaka, Bangladesh; Miller School of Medicine (M Bardhan MD), University of Miami, Miami, FL, USA; Department of Public and Environmental Health (A Barrow MPH), University of The Gambia, Banjul, The Gambia; Department of Epidemiology (A Barrow MPH), University of Florida, Gainesville, FL, USA; Department of Veterinary Physiology and Biochemistry (A Basiru PhD), Department of Veterinary Public Health and Preventive Medicine (I A Odetokun PhD), University of Ilorin, Ilorin, Nigeria; Barcelona

Institute for Global Health (Prof Q Bassat MD), ISGlobal Instituto de Salud Global de Barcelona, Barcelona, Spain; Catalan Institution for Research and Advanced Studies (ICREA), Barcelona, Spain (Prof Q Bassat MD); School of the Environment (Prof M L Bell PhD), Department of Genetics (S Pawar PhD), Yale University, New Haven, CT, USA; School of Health Policy and Management (Prof M L Bell PhD), College of Medicine (M Elhadi MD), Department of Preventive Medicine (Prof Y Lee PhD), Korea University, Seoul, South Korea; Department of Pediatrics (M Bemanalizadeh MD), Isfahan University of Medical Sciences, Isfahan, Iran; Department of Community and Family Medicine (A S Bhadoria MD), All India Institute of Medical Sciences, Rishikesh, India; Community Health Department (A S Bhadoria MD), University of South Wales, South Wales, UK; Global Health Neurology Lab (S Bhaskar MD), NSW Brain Clot Bank, Sydney, NSW, Australia; Division of Cerebrovascular Medicine and Neurology (S Bhaskar MD), National Cerebral and Cardiovascular Center, Suita, Japan; Translational and Clinical Research Institute (P Bhattacharjee MD), Newcastle University, Newcastle upon Tyne, UK; Laboratory of Translational Medicine and Nanotherapeutics (Prof J S Bhatti PhD), Department of Zoology (B Vellingiri PhD), Central University of Punjab, Bathinda, India; Department of Community and Family Medicine (B Biswas MD), All India Institute of Medical Sciences, Deoghar, India; Department of Community and Family Medicine (Prof T Bodhare MD), All India Institute of Medical Sciences, Ramanathapuram, India; Department of Anesthesia and Critical Care Medicine (S Boppana MD), Russell H. Morgan Department of Radiology and Radiological Science (A Kamireddy MD), Johns Hopkins University, Baltimore, MD, USA; Unit of Hygiene and Public Health (A Capodici MD), Romagna Local Health Authority, Forlì-Cesena, Italy; Interdisciplinary Research Center for Health Science (A Capodici MD), Sant'Anna School of Advanced Studies, Pisa, Italy; Clinical Nutrition Department (R M Chandika PhD), Epidemiology Program (M Khan MD), College of Nursing and Health Sciences (M Shanawaz MD), Jazan University, Jazan, Saudi Arabia; Department of Epidemiology and Biostatistics (V Chattu PhD), Semey Medical University (SMU), Semey, Kazakhstan; Department of Community Medicine (V Chattu PhD), Datta Meghe Institute of Medical Sciences, Sawangi, India; Department of Biology (A A Chaudhary PhD), Imam Mohammad Ibn Saud Islamic University, Riyadh, Saudi Arabia; Midwifery Department (M S Chekole MSc), Department of Public Health (L Getacher PhD), Department of Nursing (N T Sharew MSc), School of Nursing and Midwifery (B T Taye MSc), Department of Pediatrics and Child Health Nursing (W Z Teklehaimanot MSc), Debre Berhan University, Debre Berhan, Ethiopia; Faculty of Humanities and Health Sciences (H Chen MSc), Curtin University, Miri, Malaysia; Division of Plastic Surgery (D Y Cho MD), University of Wisconsin, Madison, WI, USA; Department of Community Medicine (Prof S G Choudhari MD), Jawaharlal Nehru Medical College, Wardha, India; Department of Paediatric Surgery (I S Chukwu BMedSc), Federal Medical Centre, Umuahia, Nigeria; Life and Health Sciences Research Institute (ICVS) (Prof N Cruz-Martins PhD), University of Minho, Braga, Portugal; Institute for Research and Innovation in Health (i3S) (Prof N Cruz-Martins PhD), University of Porto, Porto, Portugal; School of Nursing (A da Silva PhD), Vaccination Research Observatory (T Rodrigues da Silva PhD), Federal University of Minas Gerais, Belo Horizonte, Brazil; Department of Community Medicine (Prof T Dahiru MA), Health Systems and Policy Research Unit (Prof S Mohammed PhD), Ahmadu Bello University, Zaria, Nigeria; Public Health Foundation of India, Gurugram, India (Prof L Dandona MD, Prof R Dandona PhD, G Kumar PhD); Department of Public Health (S D Darcho MPH), School of Public Health (A Tekola MPH), Haramaya University, Harar, Ethiopia; Department of Pediatrics (A H Darwish MD), Tanta University, Tanta, Egypt; Department of Public Health (Prof F P De la Hoz PhD), National University of Colombia, Bogota, Colombia; Dirección de Nutrición (E Denova-Gutiérrez DSc), Salvador Zubiran National Institute of Medical Sciences and Nutrition, Mexico City, Mexico; Chettinad Hospital & Research Institute (Prof V

Devanbu MD), Chettinad Academy of Research and Education, Chennai, India; JSS Medical College Department of Biochemistry (D Devegowda PhD), Jagadguru Sri Shivarathreeswara Academy of Health Education and Research, Mysuru, India; Faculty of Management (A Dima PhD), Department of Statistics and Econometrics (A Mirica PhD), Bucharest University of Economic Studies, Bucharest, Romania; Department of Medicine (T C Do MD), Pham Ngoc Thach University of Medicine, Ho Chi Minh City, Viet Nam; Ateneo Center for Research and Innovation (A C Dy MD), Ateneo De Manila University, Pasig City, Philippines; Demography and Health (A C Dy MD), London School of Hygiene & Tropical Medicine, London, UK; Department of Public Health and Community Medicine (Prof I F El Bayoumy DrPH), Tanta University, Tanta city, Egypt; School of Public Health (Prof I F El Bayoumy DrPH), Texila American University, Guyana, Guyana; Pediatrics and Neonatology Department (M Eldegwi MD), Kafr Elshiekh University, Kafr Elshiekh, Egypt; Houston Methodist Hospital, Houston, TX, USA (M Elhadi MD); Department of Public Health (L T Elilo MPH), Wachemo University, Hossana, Ethiopia; Biomedical Informatics and Medical Statistics Department (Prof I El Sayed PhD), Alexandria University, Alexandria, Egypt; Satcher Health Leadership Institute (A O Fasanmi PhD), Morehouse School of Medicine, Atlanta, GA, USA; School of Medicine (A O Fasanmi PhD), Emory University, Atlanta, GA, USA; Department of Social Policy and Action (M Figueiredo OTD), Escola Superior de Saúde do Alcoitão, Alcabideche, Portugal; European Network of Occupational Therapy in Higher Education, Vienna, Austria (M Figueiredo OTD); Institute of Public Health (F Fischer PhD), Charité Universitätsmedizin Berlin (Charité Medical University Berlin), Berlin, Germany; Department of Cell Biology and Biotechnology (A A Fomenkov PhD), K.A. Timiryazev Institute of Plant Physiology, Moscow, Russia; School of Nursing and Midwifery (A T Gebremedhin MPH), Edith Cowan University, Perth, WA, Australia; School of Population Health (A T Gebremedhin MPH, S Nyadanu PhD), School of Pharmacy (A K Sendekie MSc), Curtin University, Perth, WA, Australia; Department of Public Health (G K Getahun MPH), Menelik II Medical and Health Science College, Addis Ababa, Ethiopia; Department of Nursing (A A Girmay MSc), Aksum University, Aksum, Ethiopia; Department of Health Systems and Policy Research (Prof M Golechha PhD), Indian Institute of Public Health, Gandhinagar, India; Maternal, Child and Adolescent Nutrition Department (D Gonzalez-Castell MSc), National Institute of Public Health, Cuernavaca, Mexico; Institute of Public Health (Prof M Grivna PhD), College of Medicine and Health Sciences (J Nauman PhD), United Arab Emirates University, Al Ain, United Arab Emirates; Department of Public Health and Preventive Medicine (Prof M Grivna PhD), Charles University, Prague, Czech Republic; Department of Epidemiology and Biostatistics (S Guan MD), Anhui Medical University, Hefei, China; Department of Clinical Science (M I M Gubari PhD), University Of Sulaimani, Sulaimani, Iraq; Department of Community Medicine (D A Gunawardane MD, Prof S N K Navaratna MD), University of Peradeniya, Kandy, Sri Lanka; Nanyang Maternal and Child Health Care Hospital (Z Guo MPH), Nanyang Central Hospital, Nanyang, China; Department of Public Health (B Gupta PhD), Torrens University Australia, Melbourne, VIC, Australia; Department of Epidemiology and Biostatistics (R Gupta MPH), University of South Carolina, Columbia, SC, USA; Centre for Noncommunicable Diseases and Nutrition (R Gupta MPH), School of Pharmacy (M Islam PhD), BRAC University, Dhaka, Bangladesh; Biochemistry Department (Prof N M Hamdy PhD), Department of Entomology (A M Samy PhD), Medical Ain Shams Research Institute (MASRI) (A M Samy PhD), Ain Shams University, Cairo, Egypt; Department of Epidemiology (A J Handal PhD), University of Michigan School of Public Health, Ann Arbor, MI, USA; Department of Advanced Nursing (E M M Has PhD), Universitas Airlangga (Airlangga University), Surabaya, Indonesia; School of Nursing and Midwivery (E M M Has PhD), La Trobe University, Bundoora, VIC, Australia; Department of Zoology and Entomology (A I Hasaballah PhD, M G M Zeariya PhD), Al-Azhar University, Cairo, Egypt; Department of Community

Medicine (I Hassan PhD), Federal University Teaching Hospital, Lafia, Nigeria; Department of Epidemiology and Community Medicine (I Hassan PhD), Federal University of Lafia, Lafia, Nigeria; Institute of Pharmaceutical Sciences (K Hayat MS), University of Veterinary and Animal Sciences, Lahore, Pakistan; Department of Pharmacy Administration and Clinical Pharmacy (K Hayat MS), Xian Jiaotong University, Xian, China; Department of Medicine (B Heibati PhD), University of Alberta, Edmonton, AB, Canada; Department of Microbiology (K Hezam PhD), Taiz University, Taiz, Yemen; School of Medicine (K Hezam PhD), Nankai University, Tianjin, China; Department of Statistics (M Hossain MSc), Shahjalal University of Science and Technology, Sylhet, Bangladesh; School of Health and Society (Prof H Hosseinzadeh PhD), University of Wollongong, Wollongong, NSW, Australia; Department of Legal Medicine and Bioethics (Prof S Hostiuc PhD), Department of Diabetes, Nutrition and Metabolic Diseases (Prof A Pantea Stoian PhD), Carol Davila University of Medicine and Pharmacy, Bucharest, Romania; Department of Clinical Legal Medicine (Prof S Hostiuc PhD), National Institute of Legal Medicine Mina Minovici, Bucharest, Romania; Maternal and Child Health Division (T M Huda PhD), International Centre for Diarrhoeal Disease Research, Bangladesh, Dhaka, Bangladesh; Department of Biological Sciences and Chemistry (DBSC) (Prof J Hussain PhD), Natural and Medical Sciences Research Center (A Khan PhD), University of Nizwa, Nizwa, Oman; Clinical Governance and Quality Improvement Head (D Hussein MPH), Salale University, Gerba Guracha, Ethiopia; International Master Program for Translational Science (H Huynh BS), School of Nursing (M Kurniasari PhD), Department of Global Health and Health Security (K Latief PhD), International PhD Program in Medicine (M H N Le MD), Research Center for Artificial Intelligence in Medicine (M H N Le MD), Taipei Medical University, Taipei, Taiwan; Department of Occupational Safety and Health (Prof B Hwang PhD), China Medical University, Taiwan, Taichung, Taiwan; Department of Occupational Therapy (Prof B Hwang PhD), Asia University, Taiwan, Taichung, Taiwan; Department of Health Promotion and Education (S Ibitoye PhD), College of Medicine (A P Okekunle PhD), University of Ibadan, Ibadan, Nigeria; Independent Researcher, Cairo, Egypt (T R Iskander BSc); Research and Publication Department (M Islam MSc), World Health Organization (WHO), Dhaka, Bangladesh; Institute for Physical Activity and Nutrition (Prof S Islam PhD), Deakin University, Burwood, VIC, Australia; Department of Physical Medicine and Rehabilitation (L Jacob MD), Université Paris Cité, Paris, France; Research and Development Unit (L Jacob MD), Biomedical Research Networking Center for Mental Health Network (CiberSAM), Barcelona, Spain; UNESCO-TWAS Section of Economic & Social Sciences, Humanities & Arts (Prof M Jakovljevic PhD), The World Academy of Sciences UNESCO-TWAS, Trieste, Italy; Shaanxi University of Technology, Hanzhong, China (Prof M Jakovljevic PhD); Department of Biochemistry (Prof S Jayaram MD), Government Medical College, Mysuru, India; Postgraduate Institute of Medicine (A U Jayatilleke PhD, Prof S N K Navaratna MD), University of Colombo, Colombo, Sri Lanka; Faculty of Graduate Studies (A U Jayatilleke PhD), Institute for Violence and Injury Prevention, Colombo, Sri Lanka; Department of Orthopedics (W Jin MD), Department of Epidemiology and Biostatistics (Prof S Mubarik PhD, Prof C Yu PhD), Wuhan University, Wuhan, China; Department of Biomedical Sciences (W Jin MD), City University of Hong Kong, Hong Kong, China; School of Public Health, SRMIST (Prof A Joseph PhD), Sri Ramaswamy Memorial Institute of Science and Technology, Chennai, India; Department of Community Medicine (N Joseph MD, N Kumar MD, R Thapar MD), Department of Forensic Medicine and Toxicology (Prof J Padubidri MD, P H Shetty MD), Manipal Academy of Higher Education, Mangalore, India; Department of Oral and Maxillofacial Pathology (V Kadashetti MDS), Krishna Vishwa Vidyapeeth Deemed to be University, Karad, India; Department of Statistics (Prof D H Kadir PhD), Salahaddin University, Erbil, Iraq; Department of Business Administrations (Prof D H Kadir PhD), Department of Nutrition and Dietetics (S M S Zebari PhD), Cihan

University-Erbil, Erbil, Iraq; Department of Endocrinology (S Kalra DM), Bharti Hospital Karnal, Karnal, India; University Centre for Research and Development (S Kalra DM), Chandigarh University, Mohali, India; Office of the Executive Director (Prof K K Kanmodi MPH), Cephas Health Research Initiative Inc, Ibadan, Nigeria; The Hansjörg Wyss Department of Plastic and Reconstructive Surgery (R S Kantar MD), NYU Langone Health, New York, NY, USA; Cleft Lip and Palate Surgery Division (R S Kantar MD), Global Smile Foundation, Norwood, MA, USA; Department of Physical Therapy and Health Rehabilitation (Prof F Z Kashoo PhD), Majmaah University, Majmaah, Saudi Arabia; International Research Center of Excellence (G A Kayode PhD), Institute of Human Virology Nigeria, Abuja, Nigeria; Julius Centre for Health Sciences and Primary Care (G A Kayode PhD), Utrecht University, Utrecht, Netherlands; Department of Public Health (S Kedir MSc), Werabe University, Werabe, Ethiopia; Institute of Biological Chemistry and Nutrition (T S Keflie PhD), University Hohenheim, Stuttgart, Germany; Department of Health (V Khanal PhD), Nepal Development Society, Chitwan, Nepal; Department of Preventable Non Communicable Disease (V Khanal PhD), Menzies School of Health Research, Alice Springs, NT, Australia; Department of Epidemiology (S Khanmohammadi MD), Non-Communicable Diseases Research Center (NCDRC), Tehran, Iran; College of Health, Wellbeing and Life Sciences (Prof K Khatab PhD), Sheffield Hallam University, Sheffield, UK; College of Arts and Sciences (Prof K Khatab PhD), Ohio University, Zanesville, OH, USA; Department of Basic Medical Sciences (Prof M M Khatatbeh PhD), Yarmouk University, Irbid, Jordan; Global Consortium for Public Health Research (Prof M Khatib PhD), Datta Meghe Institute of Higher Education and Research, Wardha, India; Department of Biochemistry (F Khidri PhD), Liaquat University Of Medical and Health Sciences, Jamshoro, Pakistan; School of Medicine (Prof K Kim PhD), Creighton University, Omaha, NE, USA; Cardiovascular Disease Initiative (M Kim MD), Broad Institute of MIT and Harvard, Cambridge, MA, USA; Massachusetts General Hospital, Boston, MA, USA (M Kim MD); School of Health Sciences (Prof A Kisa PhD), Kristiania University College, Oslo, Norway; Department of International Health and Sustainable Development (Prof A Kisa PhD), Tulane University, New Orleans, LA, USA; Centre for the Business and Economics of Health (I Koomson PhD), Faculty of Medicine (B Sartorius PhD), The University of Queensland, Brisbane, QLD, Australia (M Moni PhD); Department of Anthropology (Prof K Krishan PhD), Institute of Forensic Science & Criminology (V Sharma PhD), Panjab University, Chandigarh, India; Center of Medicine and Public Health (M Kulimbet MSc), Asfendiyarov Kazakh National Medical University, Almaty, Kazakhstan; Department of Community Medicine (D Kumar MD), Rajendra Institute of Medical Sciences, Ranchi, India; Department of Economics (V Kumar PhD), Manipal University, Jaipur, Jaipur, India; Faculty of Medicine and Health Science (M Kurniasari PhD), Universitas Kristen Satya Wacana (Satya Wacana Christian University), Salatiga, Indonesia; Department of Public Health and Epidemiology (D Kusuma DSc), Khalifa University of Science and Technology, Abu Dhabi, United Arab Emirates; Faculty of Public Health (D Kusuma DSc), Centre for Family Welfare (K Latief PhD), University of Indonesia, Depok, Indonesia; Division of Evidence Synthesis (C Lahariya MD), Foundation for People-centric Health Systems, New Delhi, India; Division of Lifestyle Medicine (C Lahariya MD), Centre for Health: The Specialty Practice, New Delhi, India; Faculty of Medicine (N Le MD), Department of Internal Medicine (T H Tran MD), University of Medicine and Pharmacy at Ho Chi Minh City, Ho Chi Minh City, Viet Nam; Department of Cardiovascular Research (N Le MD), Methodist Hospital, Merrillville, IN, USA; Pattern Recognition and Machine Learning Lab (Prof S Lee PhD), Gachon University, Seongnam, South Korea; Department of Precision Medicine (Prof S Lee MD), Sungkyunkwan University, Suwon-si, South Korea; Department of Epidemiology and Biostatistics (Prof J Liu PhD), Peking University, Beijing, China; Department of Quantitative Health Science (Prof X Liu PhD), Department of Pediatrics (S Sankararaman MD), Case Western Reserve University, Cleveland, OH,

USA; Department of Paediatrics (Prof R Lodha MD), All India Institute of Medical Sciences, New Delhi, India; School of Medicine (J López-Gil PhD), Universidad Espíritu Santo, Samborondón, Ecuador; Vicerrectoría de Investigación y Postgrado (J López-Gil PhD), Universidad de Los Lagos, Osorno, Chile; Ashok & Rita Patel Institute of Physiotherapy (S D Lourembam PhD), Department of Neuro Physiotherapy (M Soni PhD), Charotar University of Science and Technology, Anand, India; Centre for Public Health and Wellbeing (Z Ma PhD), University of the West of England, Bristol, UK; Faculty of Veterinary Medicine (M Mabrok PhD), Suez Canal University, Ismailia, Egypt; Department of Microbiology and Parasitology (M Mabrok PhD), King Salman International University, South of Sinai, Egypt; Rama Medical College Hospital and Research Centre, Uttar Pradesh, India (K Malhotra MBBS); Institute of Applied Health Research (K Malhotra MBBS), University of Birmingham, Birmingham, UK; Rabigh Faculty of Medicine (Prof A Malik PhD), Department of Dental Public Health (Z S Natto DrPH), King Abdulaziz University, Jeddah, Saudi Arabia; Centre for Alcohol Policy Research (CAPR) (M B Marzan MSc), School of Nursing and Midwifery (Prof M Rahman PhD), La Trobe University, Melbourne, VIC, Australia; Faculty of Humanities and Health Sciences (Prof R R Marzo MD), Curtin University, Sarawak, Malaysia; Jeffrey Cheah School of Medicine and Health Sciences (Prof R R Marzo MD), Monash University, Subang Jaya, Malaysia; Medical Scientist Training Program (S Marzouk MA), Northwestern University, Chicago, IL, USA; Department of Community Medicine (M Mathur MD), Geetanjali Medical College and Hospital in Udaipur India, Udaipur, India; Department of Social Medicine (R Mattiello PhD), Federal University of Rio Grande do Sul, Porto Alegre, Brazil; Division of Pediatric Hospital Medicine (R P Mediratta MD), Stanford University, Palo Alto, CA, USA; National Heart, Lung and Blood Institute (Prof R Mehboob PhD), National Heart, Lung, and Blood Institute, Bethesda, MD, USA; Research and Development Department (Prof R Mehboob PhD), Lahore Medical Research Center, Lahore, Pakistan; Department of Epidemiology and Biostatistics (Prof K M Mehta DSc), Department of Bioengineering and Therapeutical Sciences (Prof M Zastrozhin PhD), University of California San Francisco, San Francisco, CA, USA; University Centre Varazdin (T Mestrovic PhD), University North, Varazdin, Croatia; Department of Paediatrics (Prof S Mettananda DPhil), University of Kelaniya, Ragama, Sri Lanka; University Paediatrics Unit (Prof S Mettananda DPhil), Colombo North Teaching Hospital, Ragama, Sri Lanka; Department of Propedeutics of Internal Diseases & Arterial Hypertension (Prof T Miazgowski MD), Pomeranian Medical University, Szczecin, Poland; Multidisciplinary Department of Medical-Surgical and Dental Specialties (G Minervini PhD), University of Campania Luigi Vanvitelli, Naples, Italy; Saveetha Dental College and Hospitals (G Minervini PhD, M Tovani-Palone PhD), Center for Global Health Research (Prof A Sahebkar PhD), Saveetha University, Chennai, India; Faculty of Nursing and Midwifery (Prof M Mirghafourvand PhD), Social Determinants of Health Research Center (Prof S Mohammad-Alizadeh-Charandabi PhD), Midwifery Department (Prof S Mohammad-Alizadeh-Charandabi PhD), Tabriz University of Medical Sciences, Tabriz, Iran; College of Applied and Natural Science (J Mohamed MSc), University of Hargeisa, Hargeisa, Somalia; Molecular Biology Unit (N S Mohamed MSc), Bio-Statistical and Molecular Biology Department (N S Mohamed MSc), Sirius Training and Research Centre, Khartoum, Sudan; Modeling in Health Research Center (A Mohammadian-Hafshejani PhD), Shahrekord University of Medical Sciences, Shahrekord, Iran; Heidelberg Institute of Global Health (HIGH) (Prof S Mohammed PhD), Heidelberg University, Heidelberg, Germany; Clinical Epidemiology and Public Health Research Unit (L Monasta DSc), Burlo Garofolo Institute for Maternal and Child Health, Trieste, Italy; AI & Cyber Futures Institute (M Moni PhD), Charles Sturt University, Bathurst, NSW, Australia; Unit of Pharmacotherapy, Epidemiology and Economics (Prof S Mubarik PhD), University of Groningen (Rijksuniversiteit Groningen), Groningen, Netherlands; Knowledge Management Department (S Mukherjee PhD), Prahlad

Omkarwati Foundation (POF), Mumbai, India; Changescape Consulting (S Mukherjee PhD), Independent Consultant, New Delhi, India; Department of Surgery (F Mulita PhD), General University Hospital of Patras, Patras, Greece; Faculty of Medicine (F Mulita PhD), University of Thessaly, Larissa, Greece; Department of Pediatrics & Pediatric Pulmonology (Prof G Mustafa MD), Institute of Mother & Child Care, Multan, Pakistan; Elderly Health Research Center (A Nafei PhD), Research and Academic Institution, Tehran, Iran; Department of Computer Science and IT (G R Naik PhD), Torrens University, Adelaide, SA, Australia; Department of Health Policy and Oral Epidemiology (Z S Natto DrPH), T.H. Chan School of Public Health (P M S Pradhan MD), Division of Global Health Equity (P Rohloff MD), Harvard University, Boston, MA, USA; Department of Circulation and Medical Imaging (J Nauman PhD), Norwegian University of Science and Technology, Trondheim, Norway; Amity Institute of Forensic Sciences (B P Nayak PhD), Amity Institute of Public Health (M Shannawaz PhD), Amity University, Noida, India; Department of Community Medicine (S Nepal MD), Lumbini Medical College, Palpa, Nepal; School of Nursing (H B Netsere MSc), Department of Clinical Pharmacy (A K Sendekie MSc), University of Gondar, Gondar, Ethiopia; Department of Public Health (G Nguefack-Tsague PhD), University of Yaoundé I, Yaoundé, Cameroon; Harvard T.H. Chan School of Public Health (D Nguyen BS), Harvard University, Cambridge, MA, USA; Department of Medical Engineering (D Nguyen BS), University of South Florida, Tampa, FL, USA; Hitotsubashi Institute for Advanced Study (HIAS) (T Nguyen DrPH), Hitotsubashi University, Tokyo, Japan; Institute for Cancer Control (T Nguyen DrPH), National Cancer Center, Chuo-ku, Japan; International Islamic University Islamabad, Islamabad, Pakistan (R K Niazi PhD); Center for Public Health (L A Nnyanzi PhD), Teesside University, Middlesbrough, UK; International Research Institute of Disaster Science (IRIDeS) (Prof S Nomura PhD), Tohoku University, Miyagi, Japan; Global Research Institute (Prof S Nomura PhD), Keio University, Tokyo, Japan; Health Policy Research Center (M Nouri PhD, Y Sarikhani PhD), Shiraz University of Medical Sciences, Shiraz, Iran; Health Research Institute (M Nouri PhD), Babol University of Medical Sciences, Babol, Iran; Department of Paediatrics (C A Nri-Ezedi PhD), Nnamdi Azikiwe University, Awka, Nigeria; Department of Public Health (D Nurrika PhD), Banten School of Health Science, South Tangerang, Indonesia; Ministry of Research, Technology and Higher Education (D Nurrika PhD), Higher Education Service Institutions (LL-DIKTI) Region IV, Bandung, Indonesia; Center of Excellence in Reproductive Health Innovation (CERHI) (C I Nzoputam MPH), University of Benin, Benin City, Nigeria; Department of Physiology (O J Nzoputam PhD), University of Benin, Edo, Nigeria; Department of Physiology (O J Nzoputam PhD), Benson Idahosa University, Benin City, Nigeria; Health Promotion Research Center (H Okati-Aliabad PhD), Zahedan University of Medical Sciences, Zahedan, Iran; Department of Food and Nutrition (A P Okekunle PhD), Seoul National University, Seoul, South Korea; School of Pharmacy (O C Okonji MSc), University of the Western Cape, Cape Town, South Africa; Research Policy & Administration (J O Olusanya MBA), Centre for Healthy Start Initiative, Lagos, Nigeria (B O Olusanya PhD); School of Medicine (U L Osuagwu PhD), Western Sydney University, Bathurst, NSW, Australia; Department of Optometry and Vision Science (U L Osuagwu PhD), University of KwaZulu-Natal, KwaZulu-Natal, South Africa; Faculty of Medicine (Prof A Ouyahia PhD), University Ferhat Abbas of Setif, Sétif, Algeria; Division of Infectious Diseases (Prof A Ouyahia PhD), University Hospital of Setif, Sétif, Algeria; Department of Respiratory Medicine (Prof M P A DNB), Jagadguru Sri Shivarathreeswara University, Mysore, India; Division of Health Policy and Management (R R Parikh MD), University of Minnesota, Minneapolis, MN, USA; Faculty of Medicine and Health (J Patel MChD), University of Leeds, Leeds, UK; College of Dental Medicine (Prof S Patil PhD), Roseman University of Health Sciences, South Jordan, UT, USA; School of Population Health (Prof G Pereira PhD), Curtin University, Bentley, WA, Australia; Centre for Fertility and Health (Prof G Pereira PhD), Norwegian

Institute of Public Health, Oslo, Norway; Facultad de Medicina (Faculty of Medicine) (F E Petermann-Rocha PhD), Universidad Diego Portales (Diego Portales University), Santiago, Chile; School of Cardiovascular and Metabolic Health (F E Petermann-Rocha PhD), University of Glasgow, Glasgow, UK; Department of Internal Medicine (H Pham MD), University of Arizona, Tucson, AZ, USA; Department of Cardiovascular Medicine (H Pham MD), Mayo Clinic, Rochester, MN, USA; Department of Internal Medicine (H Pham MD), Weiss Memorial Hospital, Chicago, IL, USA; Faculty of Medicine of Nam Can Tho University (M K Phan MD), University of Medicine, Nam Can Tho University, Viet Nam; Department of Humanities and Social Sciences (Prof J Pradhan PhD), National Institute of Technology Rourkela, Rourkela, India; Department of Community Medicine and Public Health (P M S Pradhan MD), Tribhuvan University, Kathmandu, Nepal; Department of Biochemistry (Prof A Prashant PhD), JSS Academy of Higher Education and Research, Mysuru, India; Department of Biostatistics, Epidemiology, and Informatics (J Puvvula PhD), University of Pennsylvania, Philadelphia, PA, USA; Department of Neonatology (I Qattea MD), Case Western Reserve University, Akron, OH, USA; Department of Community Medicine and Family Medicine (Prof P Raghav MD), Department of Pharmacology (M Shamim MBBS, S Singh MD), Department of Biochemistry (S Tomo MD), All India Institute of Medical Sciences, Jodhpur, India; Department of Population Science and Human Resource Development (Prof M Rahman PhD, Prof M Rahman DrPH), University of Rajshahi, Rajshahi, Bangladesh; Institute of Health and Wellbeing (Prof M Rahman PhD), Federation University Australia, Berwick, VIC, Australia; Future Technology Research Center (A Rahmani PhD), National Yunlin University of Science and Technology, Yunlin, Taiwan; Health Service Research and Quality of Life Center (CEReSS) (Prof M Rahmati PhD), Aix-Marseille University, Marseille, France; Society for Health and Demographic Surveillance, Suri, India (R Rai PhD); Institute of Nutrition (R Rai PhD), Mahidol University, Salaya, Thailand; Department of Medical, Surgical and Experimental Sciences (I Raimondo MD), University of Sassari, Sassari, Italy; Gynecology and Breast Care Center (I Raimondo MD), Mater Olbia Hospital, Olbia, Italy; Department of Community Medicine (S Rajaa MD), Employees' State Insurance Model Hospital, Chennai, India; Department of Cardiology (Prof M M Ramadan PhD), Mansoura University, Mansoura, Egypt; Department of Anatomy (C Ramasamy MD), Govt. Siddhartha Medical College, Vijayawada, India; Department of Radiology (S Ramasamy MD), Stanford University, Stanford, CA, USA; Department of Research (C L Ranabhat PhD), Eastern Scientific LLC, Richmond, KY, USA; Planetary Health Research Centre (PHRC), Kathmandu, Nepal (C L Ranabhat PhD); Department of Oral Pathology, Microbiology and Forensic Odontology (S J Rao MDS), Sharavathi Dental College and Hospital, Shimogga, India; Institute of Collective Health (Prof D Rasella PhD), Federal University of Bahia, Salvador, Brazil; Barcelona Institute for Global Health, Barcelona, Spain (Prof D Rasella PhD); Unit for Public Health Science (M Rashid PhD), University of Gävle, Sweden, Stockholm, Sweden; Department of Family Medicine (Prof D Rathish PhD), Rajarata University of Sri Lanka, Anuradhapura, Sri Lanka; Department of Global Health Policy (S K Rauniyar PhD), University of Tokyo, Tokyo, Japan; WHO Collaborating Centre for Public Health Education and Training (D L Rawaf MD), Department of Primary Care and Public Health (Prof S Rawaf MD), Imperial College London, London, UK; Inovus Medical, St Helens, UK (D L Rawaf MD); Academic Public Health England (Prof S Rawaf MD), Public Health England, London, UK; Department of Epidemiology and Biostatistics (Prof M Rezaeian PhD), Rafsanjan University of Medical Sciences, Rafsanjan, Iran; Department of Nursing in Women's Health (T Rodrigues da Silva PhD), Federal University of São Paulo, São Paulo, Brazil; Department of Pharmacology and Toxicology (Prof J A B Rodriguez PhD), University of Antioquia, Medellin, Colombia; Warwick Medical School (Prof J A B Rodriguez PhD), University of Warwick, Coventry, UK; Center for Indigenous Health Research (P Rohloff MD), Wuqu' Kawoq Maya Health

Alliance, Tecpan, Guatemala; Faculty of Nursing (D S Romadlon PhD), Chulalongkorn University, Bangkok, Thailand; Faculty of Medicine (B Roy PhD), Quest International University Perak, Ipoh, Malaysia; Research Department (S Roy MSc), Indian Institute of Public Health, Delhi, India; Department of Medicine (C J Sabet MA), Georgetown University, Washington, DC, USA; Escuela de Kinesiología (Prof K P Sadarangani PhD), Diego Portales University, Santiago de Chile, Chile; Universidad Autónoma de Chile, Santiago de Chile, Chile (Prof K P Sadarangani PhD); Department of Public Health and Epidemiology (Prof B A Saddik PhD), Khalifa University, Abu Dhabi, United Arab Emirates; Operational Research Center in Healthcare (Prof U Saeed PhD), Near East University, Nicosia, Turkiye; International Center of Medical Sciences Research, Islamabad, Pakistan (Prof U Saeed PhD); Department of Psychosocial Science (Prof D Sagoe PhD), University of Bergen, Bergen, Norway; Biotechnology Research Center (Prof A Sahebkar PhD), Department of Medical Informatics (S Tabatabaei PhD), Applied Biomedical Research Center (S Tabatabaei PhD), Mashhad University of Medical Sciences, Mashhad, Iran; Department of Analytical and Applied Economics (P Sahoo MA, C Swain MPhil), Utkal University, Bhubaneswar, India; Institute of Epidemiology and Preventive Medicine (Y L Samodra PhD), National Taiwan University, Taipei, Taiwan; Benang Merah Research Center (Y L Samodra PhD), Benang Merah Research Center (BMRC), Minahasa Utara, Indonesia; Department of Pediatrics (Prof R Sanjeev MD), SRM Medical College Hospital And Research Centre, Kattankulathur, India; Department of Pediatrics (S Sankararaman MD), University Hospitals Rainbow Babies & Children's Hospital, Cleveland, OH, USA; Faculty of Medicine (Prof M M Santric-Milicevic PhD), School of Public Health and Health Management (Prof M M Santric-Milicevic PhD), University of Belgrade, Belgrade, Serbia; Department of Health, Physical Education and Recreation (J Sarfo PhD), University of Cape Coast, Cape Coast, Ghana; Department of Public Health (Y Sarikhani PhD), Jahrom University of Medical Sciences, Jahrom, Iran; Department of Food Processing Technology (T Sarkar PhD), West Bengal State Council of Technical Education, Malda, India; Department of Oral Pathology and Microbiology (Prof G S Sarode PhD, Prof S C Sarode PhD), Dr. D. Y. Patil Vidyapeeth, Pune (Deemed to be University), Pune, India; Nuffield Department of Medicine (B Sartorius PhD), University of Oxford, Oxford, UK; Department of Medical Informatics (J Saulam MSc), Kagawa University, Miki-cho, Japan; Food Processing and Nutrition (J Saulam MSc), Karnataka State Akkamahadevi Women's University, Vijayapura, India; Department of Public Health Sciences (M Sawhney PhD), University of North Carolina at Charlotte, Charlotte, NC, USA; Department of Preventive and Social Medicine (G Saya MD), Jawaharlal Institute of Postgraduate Medical Education and Research, Puducherry, India; Faculty of Business and Computing (Prof C Schinckus PhD), University of the Fraser Valley, Abbotsford, BC, Canada; Graduate School of Business (Prof C Schinckus PhD), ESAN University, Lima, Peru; Faculty of Medicine (A Schuermans BSc), Department of Cardiovascular Sciences (A Schuermans BSc, J Van den Eynde BSc), Katholieke Universiteit Leuven, Leuven, Belgium; Emergency Department (S Senthilkumaran PhD), Manian Medical Centre, Erode, India; Department of Medicine (Y Sethi MD), Swami Vivekanand Subharti University, Meerut, India; National Heart, Lung, and Blood Institute (A Seylani MD), National Institutes of Health, Rockville, MD, USA; School of Health Sciences (S Shaharudin PhD), Universiti Sains Malaysia, Kota Bharu, Malaysia; Independent Consultant, Karachi, Pakistan (M A Shaikh MD); Department of Pathology and Laboratory Medicine (S Sham MD), Northwell Health, New York, NY, USA; Interdisciplinary Center Psychopathology and Emotion Regulation (ICPE) (N T Sharew MSc), University of Groningen, Groningen, Netherlands; Department of Veterinary Public Health and Preventive Medicine (A Shittu MSc), Usmanu Danfodiyo University, Sokoto, Sokoto, Nigeria; Oulu Business School (I Shiue PhD), Martti Ahtisaari Institute (I Shiue PhD), University of Oulu, Oulu, Finland; Department of Medical-Surgical Nursing (S

Shorofi PhD), Mazandaran University of Medical Sciences, Sari, Iran; Department of Medical Microbiology and Infectious Diseases (E E Siddig MD), Erasmus University, Rotterdam, Netherlands; Anthropological Survey of India (M Sikdar PhD), Anthropological Survey of India, Mysore, India; Sport Physical Activity and Health Research & Innovation Center (SPRINT) (Prof L M L R Silva PhD), Polytechnic Institute of Guarda, Guarda, Portugal; RISE Health (Prof L M L R Silva PhD), University of Beira Interior, Covilhã, Portugal; Department of Pharmacology (H Singh DM), Government Medical College and Hospital, Chandigarh, India; School of Medicine (Prof J A Singh MD), Baylor College of Medicine, Houston, TX, USA; Department of Medicine Service (Prof J A Singh MD), US Department of Veterans Affairs (VA), Houston, TX, USA; Research Department (K Singh PhD), Hamad Medical Corporation, Doha, Qatar; Department of Biochemistry (S Solanki MD), American University of Integrative Sciences, Bridgetown, Barbados; School of Life Sciences (M Suleman PhD), Xiamen University, Xiamen, China; Faculty of Health Science (D Sulistiyorini MSc), Universitas Indonesia Maju, Jakarta, Indonesia; Department of Basic Medical Sciences (S Tabatabaeizadeh PhD), Department of Internal Medicine (S Tabatabaeizadeh PhD), Islamic Azad University, Mashhad, Iran; Saveetha Medical College and Hospital (M Tabish MPharm), Saveetha Institute of Medical and Technical Sciences, Chennai, India; Department of Epidemiology (J L Tamuzi MSc), Stellenbosch University, Cape Town, South Africa; Department of Medicine (J L Tamuzi MSc), Northlands Medical Group, Omuthiya, Namibia; Faculty of Public Health (J H V Ticoalu MPH), Universitas Sam Ratulangi (Sam Ratulangi University), Manado, Indonesia; Department of Allied Health and Human Performance (T Y Tiruye PhD), University of South Australia, Adelaide, SA, Australia; Public Health Department (T Y Tiruye PhD), Debre Markos University, Debre Markos, Ethiopia; Second Department of Internal Medicine (Q T H Tran MD), Kansai Medical University, Hirakata, Japan; Department of Business Analytics (T H Tran MD), University of Massachusetts Dartmouth, Dartmouth, MA, USA; Research and Advocacy Initiative (N Tran Minh Duc MD), ALS Vietnam, Quang Ngai, Viet Nam; Department of Medicine (Prof A Tsatsakis DSc), University of Crete, Heraklion, Greece; Department of Occupational Health and Safety (A R Tualeka PhD), University of Development, Surabaya, Indonesia; International Center for Chemical and Biological Sciences (S Ullah MSc), University of Karachi, Karachi, Pakistan; Department of Biology and Biochemistry (S Ullah MSc), University of Houston, Houston, TX, USA; Medical Genomics Research Department (Prof M Umair PhD), King Abdullah International Medical Research Center, Riyadh, Saudi Arabia; Amity Institute of Biotechnology (E Upadhyay PhD), Amity University Rajasthan, Jaipur, India; Department of Rehabilitation Sciences (J S Usman PhD), Hong Kong Polytechnic University, Hong Kong, China; Department of Infectious Disease (Prof S Vaziri MD), Kermanshah University of Medical Sciences, Kermanshah, Iran; Department of Human Genetics & Molecular Biology (B Vellingiri PhD), Bharathiar University, Coimbatore, India; Department of Health Care Administration and Economics (Prof V Vlassov MD), National Research University Higher School of Economics, Moscow, Russia; College of Medicine and Health Sciences (G Wagaw MSc), Wollo University, Dessie, Ethiopia; School of Life Course and Population Sciences (Prof Y Wang PhD), King's College London, London, UK; Department of Food Science and Human Nutrition (Prof F Wu PhD), Michigan State University, East Lansing, MI, USA; School of Public Health (H Xiao PhD), Zhejiang University, Zhejiang, China; Department of Public Health Science (H Xiao PhD), Fred Hutchinson Cancer Research Center, Seattle, WA, USA; Department of Environmental Health and Epidemiology (V Yadav MD), National Institute for Research in Environmental Health, Bhopal, India; Department of Microbiology and Immunology (G Yahya PhD), Zagazig University, Zagazig, Egypt; Department of Cells and Tissues (G Yahya PhD), Molecular Biology Institute of Barcelona, Barcelona, Spain; Department of Pediatrics (Prof D Yon MD), Kyung Hee University, Seoul, South Korea; Department of Biostatistics (Prof N Yonemoto PhD),

University of Toyama, Toyama, Japan; Department of Public Health (Prof N Yonemoto PhD), Juntendo University, Tokyo, Japan; Department of Health Sciences (S Zaman PhD), James Madison University, Harrisonburg, VA, USA; Research and Development Department (I Zare BSc), Sina Medical Biochemistry Technologies, Shiraz, Iran; Department of Administration (Prof M Zastrozhin PhD), PGxAI, San Francisco, CA, USA; Department of Public Health (M G M Zeariya PhD), University of Hail, Hail, Saudi Arabia; Department of Animal Resources (S M S Zebari PhD), Salahaddin University-Erbil, Erbil, Iraq

Authors' Contributions

Managing the overall research enterprise

Nicholas J Kassebaum, Robert C Reiner Jr.

Writing the first draft of the manuscript

Christopher E Troeger

Primary responsibility for applying analytical methods to produce estimates

Christopher E Troeger, Michael Benjamin Arndt

Primary responsibility for seeking, cataloguing, extracting, or cleaning data; designing or coding figures and tables

Michael Benjamin Arndt, Robert C Reiner Jr.

Providing data or critical feedback on data sources

Auwal Abdullahi, Armita Abedi, Richard Gyan Aboagye, Hassan Abolhassani, Yonas Derso Abtew, Ahmed Abu-Zaid, Mesafint Molla Adane, Victor Adekanmbi, Qorinah Estiningtyas Sakilah Adnani, Leticia Akua Adzigbli, Saira Afzal, Muhammad Sohail Afzal, Sajjad Ahmad, Muayyad M Ahmad, Haroon Ahmed, Ayman Ahmed, Mehrunnisha Sharif Ahmed, Budi Aji, Mohammed Albashtawy, Syed Shujait Ali, Joseph Uy Almazan, Hesham M Al-Mekhlafi, Omar Al Omari, Najim Z. Alshahrani, Awais Altaf, Nelson Alvis-Guzman, Mohammad Al-Wardat, Hany Aly, Dickson A Amugsi, Saeid Anvari, Jalal Arabloo, Mahwish Arooj, Anton A Artamonov, Nurila Aryntayeva, Seyyed Shamsadin Athari, Maha Moh'd Wahbi Atout, Asteray Assmie Ayenew, Giridhara Rathnaiah Babu, Palash Chandra Banik, Mainak Bardhan, Amadou Barrow, Shahid Bashir, Mohammad-Mahdi Bastan, Michelle L Bell, Sonu Bhaskar, Priyadarshini Bhattacharjee, Jasvinder Singh Bhatti, Bijit Biswas, Sri Harsha Boppana, Vijay Kumar Chattu, Moges Sisay Chekole, Natalia Cruz-Martins, Xiaochen Dai, Lalit Dandona, Rakhi Dandona, Samuel Demissie Darcho, Fernando Pio De la Hoz, Vinoth Gnana Chellaiyan Devanbu, Devananda Devegowda, Thanh Chi Do, Robert Kokou Dowou, Ibrahim Farahat El Bayoumy, Marwa Eldegwi, Legesse Tesfaye Elilo, Abidemi Omolara Fasanmi, Marta Figueiredo, Artem Alekseevich Fomenkov, Amin Fraij, Amanuel Tesfay Gebremedhin, Lemma Getacher, Maryam Gholamalizadeh, Alem Abera Girmay, Mahaveer Golechha, Shi-Yang Guan, Damitha Asanga Gunawardane, Rajat Das Gupta, Demewoz Haile, Nadia M Hamdy, Nasrin Hanifi, Habtamu Endashaw Hareru, Eka Mishbahatul Marah Has, Simon I Hay, Dursa Hussein, Hong-Han Huynh, Segun Emmanuel Ibitoye, Teresa R Iskander, Sheikh Mohammed Shariful Islam, Md Sahidul Islam, Mihajlo Jakovljevic, Shubha Jayaram, Achala Upendra Jayatilleke, Wenyi Jin, Vidya Kadashetti, Dler H. Hussein Kadir, Rami S Kantar, Faizan Zaffar Kashoo, Nicholas J Kassebaum, Gbenga A Kayode, Shemsu Kedir, Maseer Khan, Ajmal Khan, Khaled Khatab, Mahalaqua Nazli Khatib, Feriha Fatima Khidri, Kwanghyun Kim, Adnan Kisa, Kewal Krishan, Vijay Kumar, Dewesh Kumar, G Anil Kumar, Maria Dyah Kurniasari, Dian Kusuma, Chandrakant Lahariya, Kamaluddin Latief, Nhi Huu Hanh Le, Minh Huu Nhat Le, Sang-woong Lee, Seung Won Lee, Yo Han Lee, Virendra S Ligade, Stephen S Lim, Jue Liu, Xuefeng Liu, Zheng Feei Ma, Kashish Malhotra, Ahmad Azam Malik, Roy Rillera Marzo, Sammer Marzouk, Medha Mathur, Riffat Mehboob, Tesfahun Mekene Meto, Giuseppe Minervini, Nouh Saad Mohamed, Abdollah Mohammadian-Hafshejani, Shafiu Mohammed, Ali H Mokdad, Sumaira Mubarik, Sumoni Mukherjee, Francesk Mulita, Christopher J L Murray, Ghulam Mustafa, Ganesh R Naik, Zuhair S Natto, Biswa Prakash Nayak, Henok Biresaw Netsere, Dang Nguyen, Robina Khan Niazi, Lawrence Achilles Nnyanzi, Shuhei Nomura, Mehran Nouri, Dieta Nurrika, Chimezie Igwegbe Nzoputam, Ogochukwu Janet Nzoputam, Ismail A Odetokun, Akinkunmi Paul Okekunle, Bolajoko Olubukunola Olusanya, Jacob Olusegun Olusanya, Uchechukwu Levi Osuagwu, Amel Ouyahia, Mahesh P A, Jagadish Rao Padubidri, Anca Pantea Stoian, Romil R Parikh, Shankargouda Patil, Shrikant Pawar, Gavin Pereira, Arokiasamy Perianayagam, Hoang Nhat Pham, Hoang Tran Pham, My Kieu Phan, Jalandhar Pradhan, Jagadeesh Puvvula, Ibrahim Qattea, Pankaja Raghav, Amir Masoud Rahmani, Masoud Rahmati, Sathish Rajaa, Mahmoud Mohammed Ramadan, Shakthi Kumaran Ramasamy, Chitra Ramasamy, Chhabi Lal Ranabhat, Sowmya J Rao, Chythra R Rao, Santosh Kumar Rauniyar, David Laith Rawaf, Salman Rawaf, Robert C Reiner Jr., Thales Philipe Rodrigues da Silva, Jefferson Antonio Buendia Rodriguez,

Peter Rohloff, Debby Syahru Romadlon, Shubhanjali Roy, Cameron John Sabet, Kabir P Sadarangani, Basema Ahmad Saddik, Umar Saeed, Narjes Saheb Sharif-Askari, Pragyan Monalisa Sahoo, Abdallah M Samy, Rama Krishna Sanjeev, Milena M Santric-Milicevic, Tanmay Sarkar, Monika Sawhney, Christophe Schinckus, Subramanian Senthilkumaran, Yashendra Sethi, Allen Seylani, Samiah Shahid, Masood Ali Shaikh, Sunder Sham, Muhammad Aaqib Shamim, Mohammed Shannawaz, Vishal Sharma, Aminu Shittu, Ivy Shiue, Mithun Sikdar, Luís Manuel Lopes Rodrigues Silva, Jasvinder A Singh, Kalpana Singh, Harmanjit Singh, Reed J D Sorensen, Muhammad Suleman, Chandan Kumar Swain, Seyyed Mohammad Tabatabaei, Birhan Tsegaw Taye, Mariya Vladimirovna Titova, Marcos Roberto Tovani-Palone, Quynh Thuy Huong Tran, Nguyen Tran Minh Duc, Christopher E Troeger, Abdul Rohim Tualeka, Muhammad Umair, Bhaskaran Unnikrishnan, Era Upadhyay, Jef Van den Eynde, Balachandar Vellingiri, Vasily Vlassov, Gebeyaw Biset Wagaw, Felicia Wu, Hong Xiao, Naohiro Yonemoto, Chuanhua Yu, Iman Zare, Michael Zastrozhin, Mohammed G M Zeariya, Salih M. Mustafa Salih Zebari

Developing methods or computational machinery

Aleksandr Y Aravkin, Michael Benjamin Arndt, Xiaochen Dai, Nora M Gilbertson, Demewoz Haile, Simon I Hay, Jiawei He, Nicholas J Kassebaum, Ali H Mokdad, Christopher J L Murray, Robert C Reiner Jr., Reed J D Sorensen, Christopher E Troeger

Providing critical feedback on methods or results

Meriem Abdoun, Auwal Abdullahi, Mesfin Abebe, Armita Abedi, Alemwork Abie, Richard Gyan Aboagye, Hassan Abolhassani, Ahmed Abu-Zaid, Lawan Hassan Adamu, Mesafint Molla Adane, Isaac Yeboah Addo, Oyelola A Adegboye, Victor Adekanmbi, Qorinah Estiningtyas Sakilah Adnani, Leticia Akua Adzigbli, Saira Afzal, Muhammad Sohail Afzal, Navidha Aggarwal, Aqeel Ahmad, Sajjad Ahmad, Muayyad M Ahmad, Elham Ahmadi, Haroon Ahmed, Ayman Ahmed, Mehrunnisha Sharif Ahmed, Mushood Ahmed, Budi Aji, Mohammad Khursheed Alam, Mohammed Albashtawy, Fentahun Alemnew, Syed Mahfuz Al Hasan, Syed Shujait Ali, Mohammed Usman Ali, Rafat Ali, Waad Ali, Joseph Uy Almazan, Hesham M Al-Mekhlafi, Omar Al Omari, Mohammed A Alsabri, Najim Z. Alshahrani, Awais Altaf, Nelson Alvis-Guzman, Mohammad Al-Wardat, Hany Aly, Dickson A Amugsi, Zelalem Alamrew Anteneh, Boluwatife Stephen Anuoluwa, Saeid Anvari, Anayochukwu Edward Anyasodor, Jalal Arabloo, Demelash Areda, Michael Benjamin Arndt, Mahwish Arooj, Anton A Artamonov, Ashokan Arumugam, Bernard Kwadwo Yeboah Asiamah-Asare, Seyyed Shamsadin Athari, Maha Moh'd Wahbi Atout, Amlaku Mulat Aweke, Adedapo Wasiu Awotidebe, Asteray Assmie Ayenew, Melkalem Mamuye Azanaw, Shahkaar Aziz, Giridhara Rathnaiah Babu, Ruhai Bai, Jennifer L Baker, Wondu Feyisa Balcha, Palash Chandra Banik, Mainak Bardhan, Amadou Barrow, Shahid Bashir, Quique Bassat, Mohammad-Mahdi Bastan, Priyamadhaba Behera, Michelle L Bell, Maryam Bemanalizadeh, Ajeet Singh Bhadoria, Sonu Bhaskar, Priyadarshini Bhattacharjee, Jasvinder Singh Bhatti, Bijit Biswas, Trupti Bodhare, Sri Harsha Boppana, Angelo Capodici, Rama Mohan Chandika, Vijay Kumar Chattu, Moges Sisay Chekole, Hana Chen, Sonali Gajanan Choudhari, Isaac Sunday Chukwu, Erin Chung, Natalia Cruz-Martins, Tukur Dahiru, Xiaochen Dai, Lalit Dandona, Rakhi Dandona, Samuel Demissie Darcho, Amira Hamed Darwish, Alanna Gomes da Silva, Fernando Pio De la Hoz, Vinoth Gnana Chellaiyan Devanbu, Devananda Devegowda, Adriana Dima, Thanh Chi Do, Robert Kokou Dowou, Angel Belle Cheng Dy, Ibrahim Farahat El Bayoumy, Marwa Eldegwi, Muhammed Elhadi, Legesse Tesfaye Elilo, Abidemi Omolara Fasanmi, Marta Figueiredo, Florian Fischer, Artem Alekseevich Fomenkov, Amin Fraij, Amanuel Tesfay Gebremedhin, Lemma Getacher, Genanew K Getahun, Alem Abera Girmay, Mahaveer Golechha, Dinorah Gonzalez-Castell, Michal Grivna, Shi-Yang Guan, Mohammed Ibrahim Mohialdeen Gubari, Damitha Asanga Gunawardane, Zhifeng Guo, Rajat Das Gupta, Bhawna Gupta, Demewoz Haile, Nadia M Hamdy, Nasrin Hanifi, Habtamu Endashaw Hareru, Eka Mishbahatul Marah Has, Ahmed I Hasaballah, Ikrama Hassan, Simon I Hay, Khezar Hayat, Behzad Heibati, Austin Heuer, Kamal Hezam, Ramesh Holla, Md Sabbir Hossain, Hassan Hosseinzadeh, Tanvir M Huda, Javid Hussain, Dursa Hussein, Hong-Han Huynh, Bing-Fang Hwang, Segun Emmanuel Ibitoye, Mustapha Immurana, Teresa R Iskander, Md Rabiul Islam, Sheikh Mohammed Shariful Islam, Md Sahidul Islam, Louis Jacob, Mihajlo Jakovljevic, Shubha Jayaram, Achala Upendra Jayatilleke, Wenyi Jin, Nitin Joseph, Alex Joseph, Ali Kabir, Vidya Kadashetti, Dler H. Hussein Kadir, Kehinde Kazeem Kanmodi, Rami S Kantar, Faizan Zaffar Kashoo, Nicholas J Kassebaum, Gbenga A Kayode, Shemsu Kedir, Tibebeselassie S Keflie, Maseer Khan, Ajmal Khan, Vishnu Khanal, Shaghayegh Khanmohammadi, Khaled Khatab, Moawiah Mohammad Khatatbeh, Mahalaqua Nazli Khatib, Feriha Fatima Khidri, Kwanghyun Kim, Min Seo Kim, Adnan Kisa, Farzad Kompani, Isaac Koomson, Kewal Krishan, Vijay Kumar, Dewesh Kumar, Nithin Kumar, G Anil Kumar, Maria Dyah Kurniasari, Dian Kusuma,

Chandrakant Lahariya, Kamaluddin Latief, Nhi Huu Hanh Le, Minh Huu Nhat Le, Sang-woong Lee, Seung Won Lee, Yo Han Lee, Virendra S Ligade, Stephen S Lim, Jialing Lin, Jue Liu, Xuefeng Liu, Rakesh Lodha, José Francisco López-Gil, Surbala Devi Lourembam, Zheng Feei Ma, Mahmoud Mabrok, Kashish Malhotra, Ahmad Azam Malik, Vahid Mansouri, Emmanuel Manu, Melvin Barrientos Marzan, Roy Rillera Marzo, Sammer Marzouk, Medha Mathur, Rita Mattiello, Rishi P Mediratta, Riffat Mehboob, Kala M Mehta, Tesfahun Mekene Meto, Tomislav Mestrovic, Sachith Mettananda, Tomasz Miazgowski, Andreea Mirica, Jama Mohamed, Nouh Saad Mohamed, Sakineh Mohammad-Alizadeh-Charandabi, Abdollah Mohammadian-Hafshejani, Shafiu Mohammed, Ali H Mokdad, Mohammad Ali Moni, Sumaira Mubarik, Sumoni Mukherjee, Sileshi Mulatu, Francesk Mulita, Christopher J L Murray, Ghulam Mustafa, Ayoub Nafei, Ganesh R Naik, Zuhair S Natto, Javaid Nauman, Samidi Nirasha Kumari Navaratna, Biswa Prakash Nayak, Amanuel Tebabal Nega, Henok Biresaw Netsere, Georges Nguefack-Tsague, Dang Nguyen, The Phuong Nguyen, Robina Khan Niazi, Ali Nikoobar, Lawrence Achilles Nnyanzi, Shuhei Nomura, Mehran Nouri, Chisom Adaobi Nri-Ezedi, Dieta Nurrika, Sylvester Dodzi Nyadanu, Chimezie Igwegbe Nzoputam, Ogochukwu Janet Nzoputam, Ismail A Odetokun, Hassan Okati-Aliabad, Akinkunmi Paul Okekunle, Osaretin Christabel Okonji, Bolajoko Olubukunola Olusanya, Jacob Olusegun Olusanya, Uchechukwu Levi Osuagwu, Amel Ouyahia, Mahesh P A, Jagadish Rao Padubidri, Anca Pantea Stoian, Romil R Parikh, Shankargouda Patil, Shrikant Pawar, Gavin Pereira, Arokiasamy Perianayagam, Fanny Emily Petermann-Rocha, Hoang Nhat Pham, Hoang Tran Pham, My Kieu Phan, Jalandhar Pradhan, Pranil Man Singh Pradhan, Akila Prashant, Jagadeesh Puvvula, Pankaja Raghav, Md. Mosfequr Rahman, Muhammad Aziz Rahman, Mosiur Rahman, Amir Masoud Rahmani, Masoud Rahmati, Rajesh Kumar Rai, Sathish Rajaa, Rayan Rajabi, Mahmoud Mohammed Ramadan, Shakthi Kumaran Ramasamy, Chitra Ramasamy, Chhabi Lal Ranabhat, Sowmya J Rao, Chythra R Rao, Devarajan Rathish, Santosh Kumar Rauniyar, David Laith Rawaf, Salman Rawaf, Robert C Reiner Jr., Nazila Rezaei, Mohsen Rezaeian, Thales Philipe Rodrigues da Silva, Jefferson Antonio Buendia Rodriguez, Peter Rohloff, Debby Syahru Romadlon, Shubhanjali Roy, Cameron John Sabet, Kabir P Sadarangani, Basema Ahmad Saddik, Umar Saeed, Amene Saghazadeh, Dominic Sagoe, Narjes Saheb Sharif-Askari, Pragyan Monalisa Sahoo, Yoseph Leonardo Samodra, Abdallah M Samy, Rama Krishna Sanjeev, Milena M Santric-Milicevic, Jacob Owusu Sarfo, Yaser Sarikhani, Tanmay Sarkar, Sachin C Sarode, Gargi Sachin Sarode, Benn Sartorius, Jennifer Saulam, Monika Sawhney, Ganesh Kumar Saya, Christophe Schinckus, Art Schuermans, Ashenafi Kibret Sendekie, Subramanian Senthilkumaran, Yashendra Sethi, Shazlin Shaharudin, Samiah Shahid, Masood Ali Shaikh, Muhammad Aaqib Shamim, Mohd Shanawaz, Mohammed Shannawaz, Nigussie Tadesse Sharew, Vishal Sharma, Aminu Shittu, Ivy Shiue, Seyed Afshin Shorofi, Emmanuel Edwar Siddig, Mithun Sikdar, Luís Manuel Lopes Rodrigues Silva, Jasvinder A Singh, Kalpana Singh, Harmanjit Singh, Mansi Soni, Reed J D Sorensen, Muhammad Suleman, Desy Sulistiyorini, Chandan Kumar Swain, Seyyed Mohammad Tabatabaei, Mohammad Tabish, Jacques Lukenze Tamuzi, Birhan Tsegaw Taye, Abainash Tekola, Mohamad-Hani Temsah, Rekha Thapar, Jansje Henny Vera Ticoalu, Tenaw Yimer Tiruye, Mariya Vladimirovna Titova, Marcos Roberto Tovani-Palone, Quynh Thuy Huong Tran, Nguyen Tran Minh Duc, Christopher E Troeger, Shahid Ullah, Saeed Ullah, Muhammad Umair, Bhaskaran Unnikrishnan, Era Upadhyay, Jibrin

Sammani Usman, Jef Van den Eynde, Siavash Vaziri, Balachandar Vellingiri, Vasily Vlassov, Gebeyaw Biset Wagaw, Yanzhong Wang, Felicia Wu, Hong Xiao, Vikas Yadav, Galal Yahya, Dong Keon Yon, Naohiro Yonemoto, Chuanhua Yu, Sojib Bin Zaman, Michael Zastrozhin, Mohammed G M Zeariya, Salih M. Mustafa Salih Zebari, Claire Chenwen Zhong

Drafting the work or revising it critically for important intellectual content

Hasan Aalruz, Auwal Abdullahi, Armita Abedi, Hassan Abolhassani, Ahmed Abu-Zaid, Lawan Hassan Adamu, Mesafint Molla Adane, Isaac Yeboah Addo, Victor Adekanmbi, Juliana Bunmi Adetunji, Qorinah Estiningtyas Sakilah Adnani, Saira Afzal, Muhammad Sohail Afzal, Muayyad M Ahmad, Elham Ahmadi, Haroon Ahmed, Ayman Ahmed, Mehrunnisha Sharif Ahmed, Mushood Ahmed, Marjan Ajami, Mohammed Albashtawy, Fentahun Alemnew, Ayman Al-Eyadhy, Syed Shujait Ali, Mohammed Usman Ali, Waad Ali, Hesham M Al-Mekhlafi, Omar Al Omari, Najim Z. Alshahrani, Awais Altaf, Nelson Alvis-Guzman, Mohammad Al-Wardat, Hany Aly, Dickson A Amugsi, Abhishek Anil, Zelalem Alamrew Anteneh, Boluwatife Stephen Anuoluwa, Saeid Anvari, Anayochukwu Edward Anyasodor, Jalal Arabloo, Michael Benjamin Arndt, Mahwish Arooj, Ashokan Arumugam, Bernard Kwadwo Yeboah Asiamah-Asare, Seyyed Shamsadin Athari, Maha Moh'd Wahbi Atout, Adedapo Wasiu Awotidebe, Asteray Assmie Ayenew, Giridhara Rathnaiah Babu, Ruhai Bai, Jennifer L Baker, Palash Chandra Banik, Mainak Bardhan, Amadou Barrow, Shahid Bashir, Afisu Basiru, Quique Bassat, Mohammad-Mahdi Bastan, Priyamadhaba Behera, Michelle L Bell, Maryam Bemanalizadeh, Ajeet Singh Bhadoria, Sonu Bhaskar, Priyadarshini Bhattacharjee, Jasvinder Singh Bhatti, Catherine Bisignano, Bijit Biswas, Srinivasa Rao Bolla, Sri Harsha Boppana, Angelo Capodici, Rama Mohan Chandika, Vijay Kumar Chattu, Anis Ahmad Chaudhary, Hana Chen, Daniel Youngwhan Cho, Natalia Cruz-Martins, Samuel Demissie Darcho, Amira Hamed Darwish, Alanna Gomes da Silva, Fernando Pio De la Hoz, Edgar Denova-Gutiérrez, Devananda Devegowda, Adriana Dima, Thanh Chi Do, Ibrahim Farahat El Bayoumy, Marwa Eldegwi, Muhammed Elhadi, Iman El Sayed, Marta Figueiredo, Florian Fischer, Amin Fraij, Amanuel Tesfay Gebremedhin, Lemma Getacher, Alem Abera Girmay, Michal Grivna, Shi-Yang Guan, Damitha Asanga Gunawardane, Rajat Das Gupta, Bhawna Gupta, Nadia M Hamdy, Alexis J Handal, Nasrin Hanifi, Habtamu Endashaw Hareru, Eka Mishbahatul Marah Has, Ahmed I Hasaballah, Simon I Hay, Behzad Heibati, Kamal Hezam, Ramesh Holla, Md Sabbir Hossain, Sorin Hostiuc, Tanvir M Huda, Hong-Han Huynh, Segun Emmanuel Ibitoye, Mustapha Immurana, Teresa R Iskander, Md Rabiul Islam, Sheikh Mohammed Shariful Islam, Md Sahidul Islam, Louis Jacob, Mihajlo Jakovljevic, Shubha Jayaram, Achala Upendra Jayatilleke, Wenyi Jin, Nitin Joseph, Ali Kabir, Vidya Kadashetti, Arun Kamireddy, Kehinde Kazeem Kanmodi, Rami S Kantar, Faizan Zaffar Kashoo, Nicholas J Kassebaum, Gbenga A Kayode, Shemsu Kedir, Tibebeselassie S Keflie, Maseer Khan, Ajmal Khan, Vishnu Khanal, Shaghayegh Khanmohammadi, Khaled Khatab, Moawiah Mohammad Khatatbeh, Mahalaqua Nazli Khatib, Feriha Fatima Khidri, Kwanghyun Kim, Min Seo Kim, Adnan Kisa, Isaac Koomson, Kewal Krishan, Mukhtar Kulimbet, Dewesh Kumar, Almagul Kurmanova, Maria Dyah Kurniasari, Dian Kusuma, Chandrakant Lahariya, Kamaluddin Latief, Nhi Huu Hanh Le, Minh Huu Nhat Le, Sang-woong Lee, Jue Liu, Rakesh Lodha, José Francisco López-Gil, Zheng Feei Ma, Mahmoud Mabrok, Kashish Malhotra, Ahmad Azam Malik, Vahid Mansouri, Emmanuel Manu, Melvin Barrientos Marzan, Roy Rillera Marzo, Sammer Marzouk, Medha Mathur, Rita Mattiello, Rishi P Mediratta, Riffat Mehboob, Kala M Mehta, Tomislav Mestrovic, Sachith Mettananda, Tomasz Miazgowski, Mojgan Mirghafourvand, Nouh Saad Mohamed, Sakineh Mohammad-Alizadeh-Charandabi, Abdollah Mohammadian-Hafshejani, Shafiu Mohammed, Ali H Mokdad, Lorenzo Monasta, Mohammad Ali Moni, Francesk Mulita, Christopher J L Murray, Ghulam Mustafa, Ayoub

Nafei, Zuhair S Natto, Javaid Nauman, Samidi Nirasha Kumari Navaratna, Biswa Prakash Nayak, Samata Nepal, Georges Nguefack-Tsague, Dang Nguyen, Robina Khan Niazi, Lawrence Achilles Nnyanzi, Chisom Adaobi Nri-Ezedi, Sylvester Dodzi Nyadanu, Chimezie Igwegbe Nzoputam, Ogochukwu Janet Nzoputam, Ismail A Odetokun, Osaretin Christabel Okonji, Bolajoko Olubukunola Olusanya, Jacob Olusegun Olusanya, Uchechukwu Levi Osuagwu, Amel Ouyahia, Mahesh P A, Jagadish Rao Padubidri, Anca Pantea Stoian, Romil R Parikh, Jay Patel, Shankargouda Patil, Shrikant Pawar, Gavin Pereira, Arokiasamy Perianayagam, Fanny Emily Petermann-Rocha, Hoang Nhat Pham, Hoang Tran Pham, My Kieu Phan, Jalandhar Pradhan, Pranil Man Singh Pradhan, Akila Prashant, Jagadeesh Puvvula, Ibrahim Qattea, Pankaja Raghav, Amir Masoud Rahmani, Masoud Rahmati, Ivano Raimondo, Sathish Rajaa, Mahmoud Mohammed Ramadan, Shakthi Kumaran Ramasamy, Chitra Ramasamy, Chhabi Lal Ranabhat, Sowmya J Rao, Chythra R Rao, Davide Rasella, Mamunur Rashid, Devarajan Rathish, Santosh Kumar Rauniyar, David Laith Rawaf, Salman Rawaf, Robert C Reiner Jr., Jefferson Antonio Buendia Rodriguez, Peter Rohloff, Debby Syahru Romadlon, Shubhanjali Roy, Bedanta Roy, Cameron John Sabet, Kabir P Sadarangani, Basema Ahmad Saddik, Umar Saeed, Dominic Sagoe, Amirhossein Sahebkar, Narjes Saheb Sharif-Askari, Abdallah M Samy, Senthilkumar Sankararaman, Milena M Santric-Milicevic, Jacob Owusu Sarfo, Yaser Sarikhani, Sachin C Sarode, Gargi Sachin Sarode, Ganesh Kumar Saya, Art Schuermans, Ashenafi Kibret Sendekie, Yashendra Sethi, Allen Seylani, Shazlin Shaharudin, Samiah Shahid, Muhammad Aaqib Shamim, Mohd Shanawaz, Mohammed Shannawaz, Vishal Sharma, Pavanchand H Shetty, Aminu Shittu, Seyed Afshin Shorofi, Emmanuel Edwar Siddig, Mithun Sikdar, Luís Manuel Lopes Rodrigues Silva, Jasvinder A Singh, Surjit Singh, Harmanjit Singh, Shipra Solanki, Mansi Soni, Muhammad Suleman, Desy Sulistiyorini, Chandan Kumar Swain, Seyed-Amir Tabatabaeizadeh, Jacques Lukenze Tamuzi, Birhan Tsegaw Taye, Wegayehu Zeneb Teklehaimanot, Mohamad-Hani Temsah, Tenaw Yimer Tiruye, Sojit Tomo, Marcos Roberto Tovani-Palone, Thang Huu Tran, Nguyen Tran Minh Duc, Christopher E Troeger, Aristidis Tsatsakis, Muhammad Umair, Bhaskaran Unnikrishnan, Era Upadhyay, Jibrin Sammani Usman, Jef Van den Eynde, Balachandar Vellingiri, Vasily Vlassov, Gebeyaw Biset Wagaw, Yanzhong Wang, Vikas Yadav, Galal Yahya, Dong Keon Yon, Naohiro Yonemoto, Sojib Bin Zaman, Iman Zare, Michael Zastrozhin, Mohammed G M Zeariya, Salih M. Mustafa Salih Zebari, Claire Chenwen Zhong

Managing the estimation or publications process

Simon I Hay, Nicholas J Kassebaum, Stephen S Lim, Ali H Mokdad, Christopher J L Murray, Robert C Reiner Jr.
